# TagC-RED: An Infrared-Triggered Retro-Ene Reaction for Deep-Tissue Bioconjugation

**DOI:** 10.1021/jacs.6c01581

**Published:** 2026-05-11

**Authors:** Sang Mi Suh, Benjamin Ben-Zvi, John M. Talbott, Niket Manoj, Brock M. Nelson, Riley R. Hughes, Graham C. Haug, Shohei Koide, Robert S. Paton, Monika Raj, Tianning Diao

**Affiliations:** † Department of Chemistry, 5894 New York University, 100 Washington Square East, New York, New York 10003, United States; ‡ Department of Chemistry, 1371 Emory University, 1515 Dickey Dr, Atlanta, Georgia 30322, United States; § Department of Chemistry, 224023 Colorado State University, 1301 Center Avenue, Ft. Collins, Colorado 80523-1872, United States; ∥ Department of Biochemistry and Molecular Pharmacology, 12296 New York University Grossman School of Medicine, and Perlmutter Cancer Center, New York University Langone Health, 522 first Ave, Smilow Research Center, New York, New York 10016, United States

## Abstract

Bioconjugation reactions are indispensable for probing biomolecules in their native environments. Photoactivatable bioconjugation offers spatiotemporal control; however, current methods face significant limitations, including the requirement for noncanonical functional groups, cytotoxic heavy-metal catalysts, and high-energy UV or visible light (λ < 800 nm), which restrict tissue penetration and increase phototoxicity risks. In response, we introduce TagC-RED, a Retro-Ene type sigmatropic rearrangement of Diazonium compounds for Cysteine-specific bioconjugation activated by infrared light (λ > 1000 nm). TagC-RED is quantitative, rapid, and catalyst-free, with deep tissue penetration, enabling robust labeling intracellularly and *in vivo*. Mechanistic studies and DFT calculations show that TagC-RED activates through the formation of an electron donor–acceptor (EDA) complex that enables IR irradiation and undergoes a stepwise retro-ene reaction. TagC-RED holds significant potential as a platform for *in vivo* chemical biology and diagnostic innovation.

## Graphical abstract

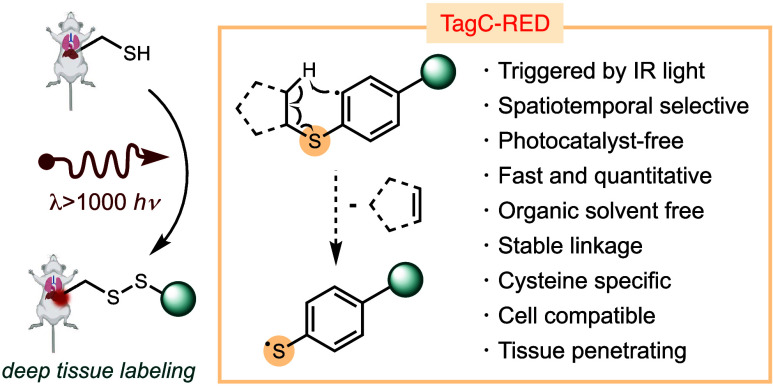

## Introduction

Bioconjugation chemistry has profoundly advanced the study of functional biomolecules within their native environment.^1–4^ These reactions allow the attachment of probes, inhibitors, or other functional groups to specific sites within biomolecules, thereby facilitating the controlled and targeted investigation of complex biological processes.^5–8^ For instance, bioconjugation enables protein profiling through the attachment of various probes to endogenous proteins. This approach enhances our understanding of disease-relevant targets and biological pathways, thereby accelerating modern drug discovery.^9^ Given the sensitivity of biological systems, the chemical reactions employed in bioconjugation must meet several criteria: (1) rapid, to ensure reactivity at low reagent concentrations; (2) specific, to avoid off-target activity; (3) efficient, to achieve sufficient sensitivity; and (4) compatible with the physiological conditions of the intracellular environment.

Photoactivatable bioconjugation chemistry offers spatiotemporal control over the labeling of biological molecules, presenting significant potential for studying dynamic processes such as protein interactions, signaling pathways, and intracellular transport.^10–14^ Additionally, photobioconjugation holds promise for light-mediated therapeutics, enabling the precise activation of therapeutic reagents and imaging probes.^15^ However, the application of photobioconjugation in cells and tissues remains limited. Conventional and recently developed methodologies rely on high-energy UV (λ = 200–400 nm) or visible light (λ = 400–800 nm),^16–19^ which can be phototoxic and suffer from limited tissue penetration (Figure 1A). An ideal photobioconjugation method would be triggered by longer wavelength irradiation, such as the infrared (λ = 1000–2000 nm) region. This approach would enhance tissue penetration, improve spatiotemporal resolution at fixed tissue depths, and mitigate light absorption, scattering, and phototoxicity to healthy tissues.^20,21^ However, such a method is currently unavailable.

**Figure 1:**
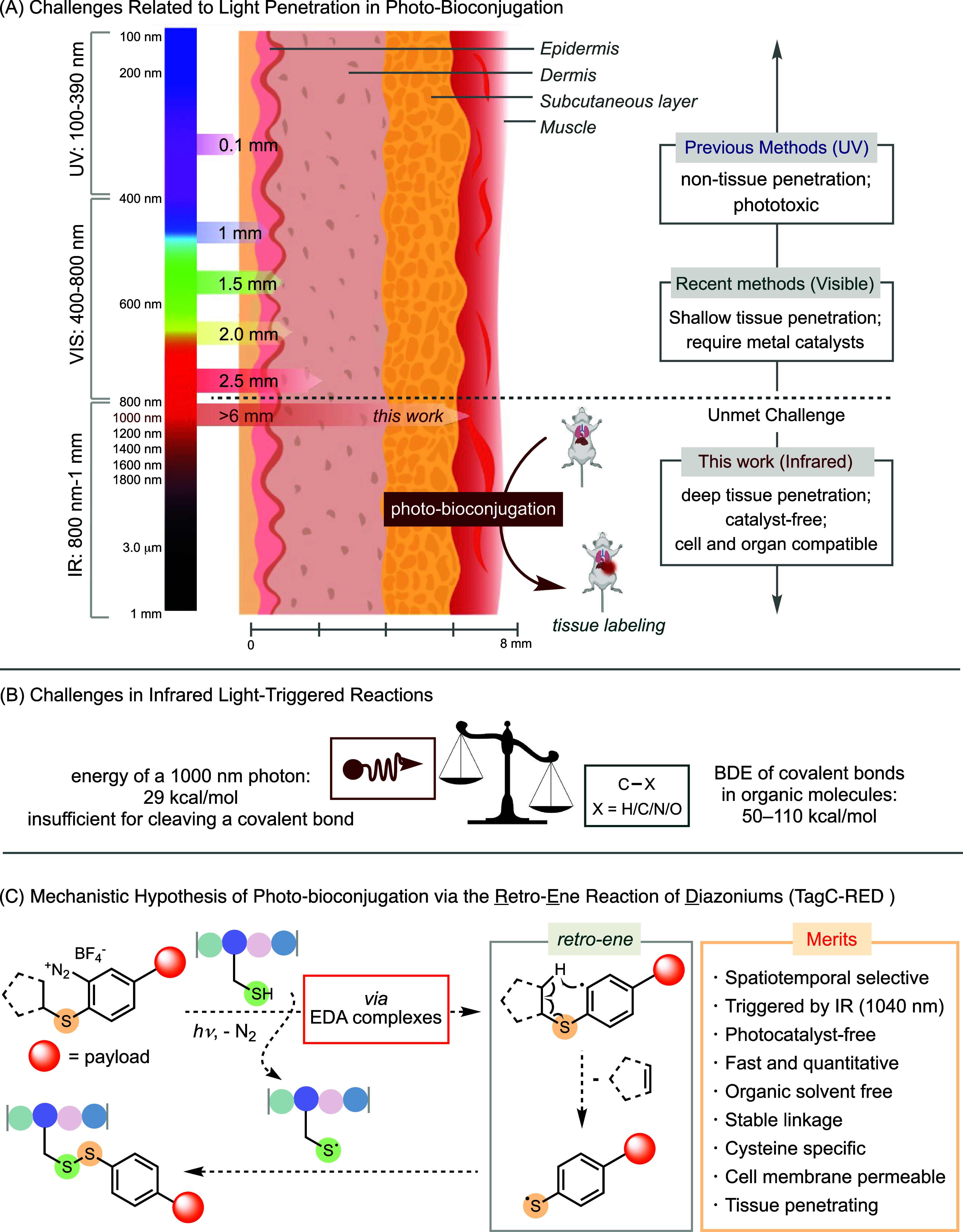
Challenges and solutions to photobioconjugation. (A) Existing challenges in photobioconjugation associated with the necessity of high-energy irradiation. (B) Chemical challenges of using infrared light for triggering reactions involving covalent bond cleavage. (C) Mechanistic hypothesis of the retro-ene reaction for catalyst-free photobioconjugation triggered by infrared light.

The need for high-energy light in conventional photobioconjugation arises from the strength of covalent bonds, which are typically greater than 50 kcal/mol, corresponding to photons with wavelengths shorter than 572 nm. Recent efforts to address this challenge include biorthogonal reactions using highly reactive noncanonical functional groups, but their direct application to native biomolecules remains less practical.^22–26^ Another approach applies photocatalysts activated by red (λ = 660 nm) or near-infrared (λ = 700–800 nm) light.^27–31^ Despite their potential, heavy-metal photocatalysts raise concerns about metal toxicity, uncertain biostability, and unintended off-target photoactivation. Additionally, the use of organic cosolvents can denature proteins and compromise their structural integrity and functional efficacy. These challenges, coupled with insufficient tissue penetration of UV or visible light, prevent current methods from operating effectively in cells and tissues.

A fundamental challenge in developing chemical reactions triggered by low-energy, long-wavelength light, whether for bioconjugation or in any other context, is the mismatch between photon energy and the energy required to cleave covalent bonds.^32^ A photon at 1000 nm carries only ∼29 kcal/mol, whereas typical covalent bond dissociation energies range from 50 to 110 kcal/mol (Figure 1B). As a result, there are virtually no examples of chemical reactions involving covalent bond cleavage or formation that proceed via direct activation by such low-energy irradiation.

While the photons lack the energy to directly cleave typical covalent bonds, they do carry sufficient energy (1.24 eV) to induce electron transfer, especially within electron–donor–acceptor (EDA) complexes.^33^ We hypothesize that a 1000 nm photon can promote the reduction of aryl diazonium compounds (*E*_p_ = −0.16 V vs SCE)^34–37^ by cysteine (Cys) (*E* = −0.22 V vs SCE),^38,39^ upon the formation of an EDA adduct. This redox process generates an aryl radical via nitrogen extrusion and a Cys radical (Figure 1C). The subsequent 1,5-hydrogen atom abstraction, driven by the high bond strength of the Csp^2^–H bond (110 kcal/mol),^40^ can initiate a retro-ene-type sigmatropic rearrangement. This reaction releases a thiophenol radical, which then selectively conjugates to Cys. The low abundance of Cys in proteins can further enhance the specificity.^41–45^ Based on this design, we introduce TagC-RED (Tag Cysteine via Retro-Ene of Diazoniums), a rearrangement triggered by infrared irradiation (λ > 1000 nm) without a photocatalyst. Compared to conventional^46–48^ and recent Cys ligation techniques,^49,50^ TagC-RED stands out as the only current method enabling cysteine-specific bioconjugation under infrared irradiation (λ > 1000 nm), without requiring a photocatalyst, while achieving highly selective and efficient labeling *in vivo* with deep tissue penetration.

## Results and Discussion

We tested our hypothesis by first synthesizing diazonium compound **1**, which features an adjacent thiol ether and a tethered alkyne (Figure 2A). We then developed photoredox conditions for TagC-RED with **1**, using the 8-mer peptide AcNH-Gln-Lys-His-Asp-Cys-Met-Tyr-Ser-CONH_2_ **2** as a model substrate, which contains a variety of nucleophilic side chains. Our initial examination of photoconditions involved green light (525 nm) irradiation at ambient temperature. Since diazonium compound **1** is water-soluble, we carried out our studies in an aqueous solution without the need for organic cosolvents. We exposed an aqueous solution of peptide **2** with three equivalents of **1** to green light in the presence of catalytic Eosin Y and observed the quantitative conversion of peptide **2** into **3** within 30 min (Figure 2A, entry 1). Using new methylene blue as the photocatalyst resulted in a lower conversion (entry 2). We attribute this to the competing light absorption by new methylene blue. Remarkably, even without a photocatalyst, we observed a complete conversion of **2** to **3** upon irradiation with 525 nm light (entry 3).

**Figure 2:**
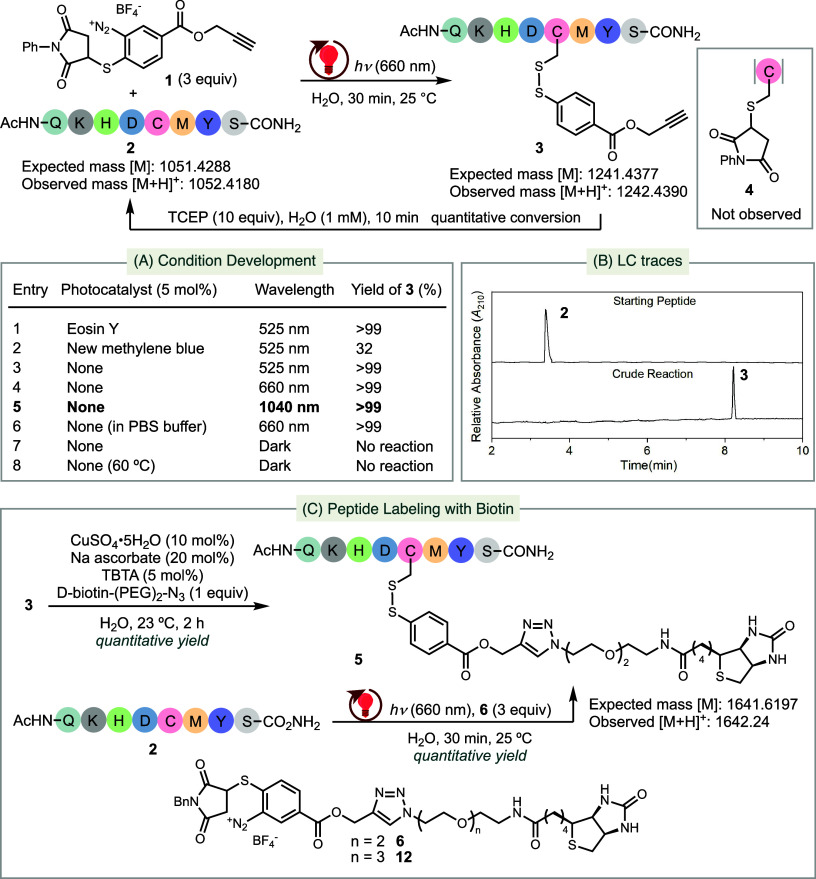
Bioconjugation of peptides with infrared irradiation. (A) Development of conditions for the bioconjugation of Cys-containing peptides. (B) HPLC trace of the crude reaction mixture. (C) Application of TagC-RED to label peptides with d-biotin.

Exploration into the effect of wavelength revealed that irradiation with 660 nm red LED lamps or a 1040 nm infrared laser pulsed at 50 Hz also resulted in a quantitative conversion of **2** to **3** (entries 4–5). The pulsed infrared laser beam passed through a filter to eliminate any residual guide beam shorter than 750 nm. Performing the reaction in PBS (phosphate-buffered saline) buffer solutions generated the same results (entry 6). Changing the pH to 6.2 or 7.8 did not affect the reaction outcome (cf. SI). However, more basic conditions are expected to lead to the decomposition of **1**. Control experiments confirmed no reaction between **1** and **2** in the absence of light even at elevated temperatures (entries 7–8). In all reactions, the potential byproduct **4**, which could derive from the reaction between Cys and maleimide, was not observed. The absence of **4** highlights the fast reactivity of TagC-RED relative to the traditional conjugate addition. Relevant to potential chemical biology applications, the maleimide byproduct exhibits no cytotoxicity at concentrations up to 10 μM.^51^ As the probe is not used in large excess and maleimide only forms upon each productive conjugation, significant cytotoxicity arising from maleimide is not anticipated.

We confirmed the identity of **3** using LC-MS (Figure 2B), ^1^H NMR, and HRMS (Figure S20). Treating **3** with excess tris­(2-carboxyethyl)­phosphine (TCEP) resulted in its complete reversion to **2** (Figure S18).^52^ These data verify the specificity of our approach to Cys, indicating the absence of off-target labeling of other nucleophilic residues within the peptide.

To further assess the selectivity of TagC-RED toward various nucleophilic and photoexcitable residues, we conducted bioconjugation reactions with individual amino acids. Under 660 nm light irradiation, applying **1** to Cys resulted in quantitative conjugation (Figure S21), while no reaction occurred with l-lysine, l-serine, l-tyrosine, l-tryptophan, l-threonine, and l-aspartic acid under identical conditions (Figures S22–S27). Moreover, we tested the reactivity of **1** with disulfides by irradiating a mixture of **1** and oxytocin with 660 nm light, which resulted in no reaction ().

Subsequently, we explored the application of TagC-RED for labeling peptides with biotin (Figure 2C). We functionalized TagC-RED-derived peptide **3** via “Click” chemistry, attaching a biotin-tethered azide to generate biotin-labeled peptide **5** in quantitative yield (Figure 2C). Alternatively, by conducting TagC-RED on peptide **2** with a biotin-attached diazonium reagent **6**, we directly obtained **5** in quantitative yield (Figure 2C).

TagC-RED proved to be compatible and effective in labeling proteins with various functional payloads (Figure 3). We expressed and purified ubiquitin with a Cys mutation at Lys 63 (Ub-K63C).^53^ Exposing Ub-K63C to TagC-RED conditions with an alkyne-tethered diazonium reagent **1** resulted in its complete transformation to **7** within 30 min, as evidenced by the disappearance of the Ub-K63C peak and the emergence of the peak corresponding to **7** by MALDI-TOF analysis (Figure 3A).

**Figure 3:**
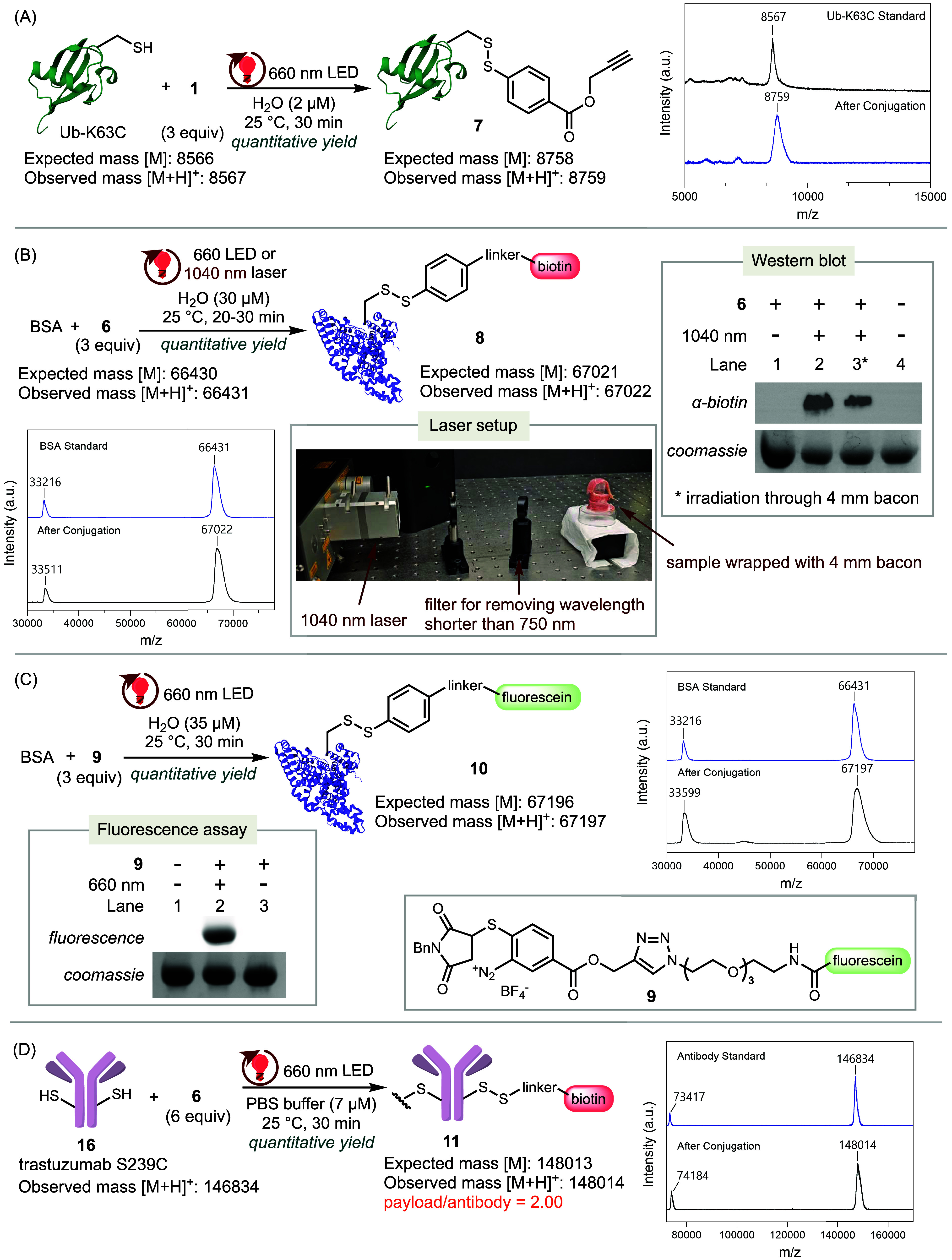
Application of TagC-RED for protein labeling. (A) Conjugation of Ub-K63C with a “Click” handle under 660 nm irradiation. (B) Conjugation of BSA with Biotin under 660 or 1040 nm irradiation. Western blot: Lane 1: BSA in the presence of **6** under ambient light; Lane 2: BSA in the presence of **6** exposed to pulsed 1040 nm IR irradiation; Lane 3: BSA in the presence of diazonium **6**, wrapped with 4 mm-thick bacon, exposed to pulsed 1040 nm IR laser irradiation; Lane 4: BSA alone in the absence of **6**. (C) Conjugation of BSA with fluorescein under 660 nm irradiation. SDS-PAGE: Lane 1: BSA alone in the absence of **9**; Lane 2: BSA in the presence of **9** with 660 nm red light irradiation; Lane 3: BSA in the presence of **9** in the dark. (D) Conjugation of trastuzumab S239C with d-biotin under 660 nm red light irradiation.

Furthermore, the bioconjugation of bovine serum albumin (BSA) with the biotin-labeling reagent **6,** using either 660 nm light or 1040 nm pulsed laser irradiation, resulted in complete conversion as evidenced by MALDI-TOF peaks corresponding to masses of 67022 and 33511, which are assigned to biotin-labeled BSA **8** (Figure 3B). Even when the reaction vessel was wrapped with 4 mm-thick bacon, to imitate the tissue layer, we still observed the quantitative conversion of BSA to **8**. SDS-PAGE (sodium dodecyl sulfate–polyacrylamide gel electrophoresis) followed by Western blot analysis of protein **8** confirmed efficient biotinylation upon irradiation at either 660 or 1040 nm, with or without tissue shielding (bacon wrapping), as evidenced by distinct bands (Figures 3B, lanes 2 and 3, S35, and S42). In contrast, control experiments in the absence of **6** (lane 4) or lack of light exposure (lane 1) produced no detectable signal, underscoring the light- and probe-dependent specificity of the reaction.

Next, we prepared fluorescent-labeling agent **9** with a tethered fluorescein. Under our standard conditions, irradiating a mixture of BSA and **9** with 660 nm light resulted in the quantitative formation of fluorescein-labeled BSA **10**, as confirmed by MALDI-TOF analysis (Figure 3C). SDS-PAGE fluorescence imaging of **10** revealed strong fluorescent bands (Figures 3C, lane 2, and S33), whereas control reactions in the absence of **9** (lane 1) or lack of light irradiation (lane 3) showed no signal. Notably, the presence of the fluorescent dye did not interfere with the efficiency or specificity of the bioconjugation reaction.

The specificity of TagC-RED allowed for uniformity in the drug-to-antibody ratio (DAR) when applied to modified antibody-drug conjugates (ADCs). We produced a humanized monoclonal anti-HER2 antibody, trastuzumab, in the IgG format, containing the Fc-silencing “LALAPG” mutations as well as a Ser-to-Cys mutation at position 239 of the heavy chains.^54,55^ By applying TagC-RED with biotin-labeling reagent **6**, we created conjugates with a precise 2:1 payload-to-antibody ratio (Figure 3D).

We evaluated the compatibility of TagC-RED within cells. We treated HeLa cells with a 200 μM solution of biotin-labeling reagent **12**, a derivative of **6** with a tetra-ethylene glycol linker for improved solubility, in 1× PBS (Figure 4A). After the irradiation of the cells with a 1040 nm IR laser for 20 min, we performed three rounds of PBS washes on the irradiated cells and then isolated the cellular proteins. Western blot analysis of the cell lysate with an antibiotin antibody conjugated with HRP revealed that proteins from the irradiated cells showed bright bands with intense chemiluminescence, indicating biotinylation of cellular proteins (Figure 4B, Lane 3). When the cell suspension was wrapped with 4 mm-thick bacon, to imitate a tissue layer, we still observed the same intense chemiluminescence (Lane 4). In contrast, control groups—HeLa cells incubated in the absence of **12** (Lane 1) or HeLa cells incubated with **12** in the dark for 20 min (Lane 2)—showed limited or no biotinylation. These results demonstrate that light-controlled labeling by TagC-RED is compatible with the complex intracellular environment and that temporal photolabeling of cellular proteins using this protocol is feasible.

**Figure 4:**
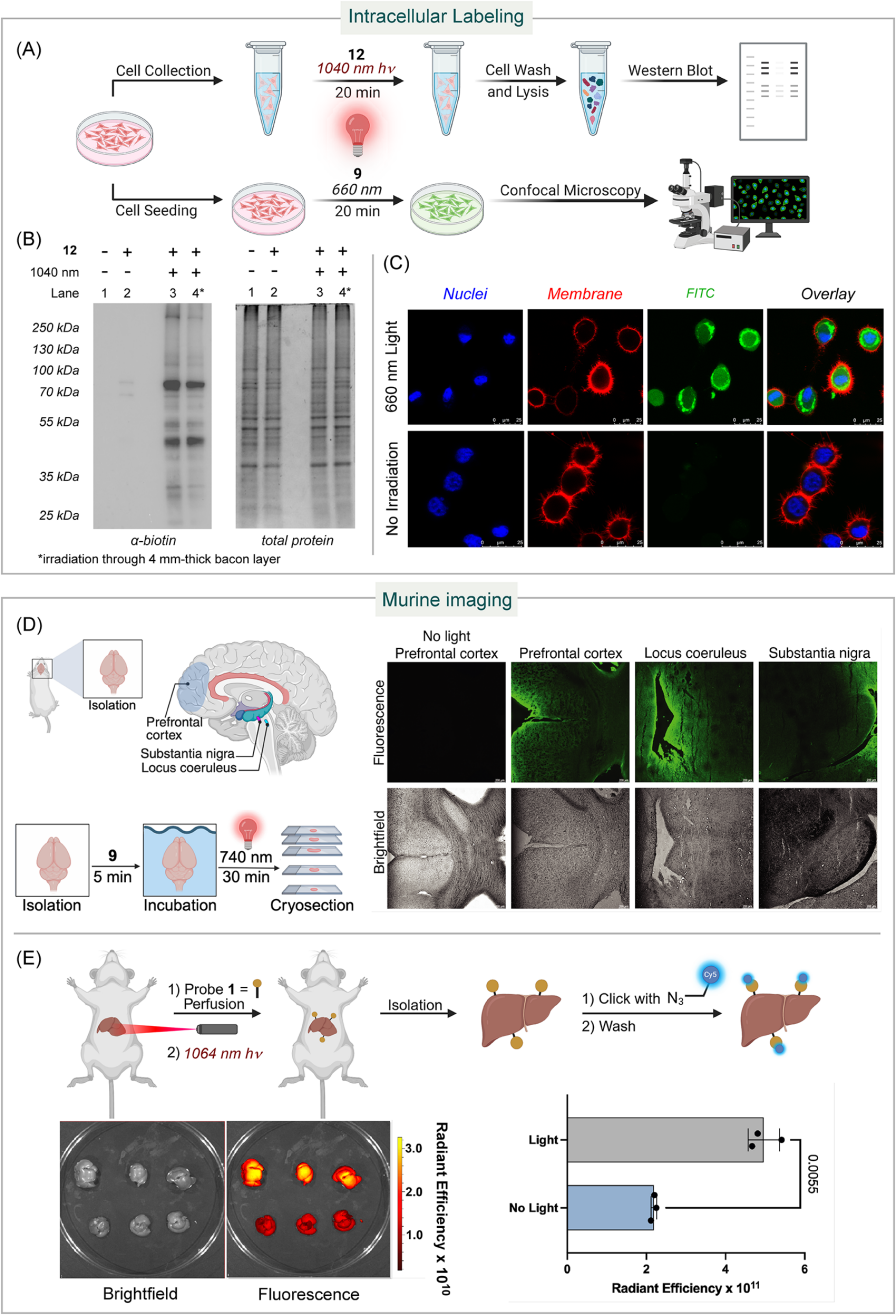
Application of TagC-RED to intracellular bioconjugation and tissue imaging. (A) Schematic of the intracellular protein labeling protocol with TagC-RED. (B) Western blot analysis of HeLa cells treated with TagC-RED biotin-labeling with or without 1040 nm IR irradiation. (C) Confocal images of HeLa cells treated with TagC-RED fluorescent labeling probe **9** with and without 660 nm red light irradiation. Depicted scale bar is 10 μm. (D) Tissue imaging of mice organs via TagC-RED. Fluorescence and brightfield imaging of mice brain tissue after the whole brain was treated with TagC-RED with and without 740 nm IR irradiation. No fluorescence observed without light treatment. Scale bar represents 200 μm. Experiments were repeated in triplicate with similar imaging results. (E) *in vivo* labeling of mice with probe **1** followed by *ex vivo* labeling with Cy5 azide via Click chemistry. IVIS Imaging and quantification of livers exposed *in vivo* to probe **1** with or without light. Top row = 1064 nm light exposure, bottom = no light exposure. Experiments were repeated in triplicate.

TagC-RED was also applicable to intracellular fluorescence labeling. We subjected HeLa cells to a 150 μM solution of fluorescein-labeling reagent **9** in 1× MEM (minimum essential media) buffer. After irradiating the cells with 660 nm light for 20 min, we washed them with 1× PBS and stained them with a membrane dye (ab219942) and nuclear stain (Hoechst 33342). Under a confocal super-resolution microscope, we observed robust endosome fluorescence from the fluorescein labeling (Figure 4C). In contrast, the control group, where HeLa cells were incubated with **9** in the dark for 20 min, showed no fluorescence signal. These results suggest that reagent **9** is cell-membrane permeable and enables light-controlled fluorescence labeling within cells, highlighting the capability of TagC-RED for intracellular labeling. In addition, the relative TagC-RED-mediated intracellular fluorescence was able to differentiate two different cell lines, MDA-MB-231 and MCF 10A, by their respective differences in cellular cysteine levels (Figure S51). Both cell lines exhibited light-controlled intracellular fluorescence; however, MDA-MB-231, known to have elevated intracellular cysteine expression relative to MCF 10A,^56^ exhibited a greater fluorescence intensity, demonstrating the potential for quantitative fluorescence analysis.

To showcase the versatility of TagC-RED, we demonstrated its capability to perform bioconjugation labeling within whole organs. Following the sacrifice of mice, we treated whole brain organs with 150 μM of fluorescent-labeling agent **9** for 5 min, then illuminated them with two 740 nm Kessil lamps for 30 min (Figure 4D). After washing the whole brain organs with PBS, we cryosectioned them to reveal slices containing the prefrontal cortex (PFC), locus coeruleus (LC), and substantia nigra (SN). All slices displayed significant fluorescence throughout the sagittal sections, underscoring the remarkable tissue penetration of the TagC-RED probe. Notably, the fluorescence of LC and SN, deeply embedded within the brain, highlights the ability of TagC-RED to label proteins in regions that current probes struggle to access. Importantly, control samples of brains that were not exposed to light exhibited no fluorescence, further affirming the high photoselectivity of TagC-RED.

To demonstrate the translational potential of TagC-RED, we investigated its efficacy in *in vivo* bioconjugation applications by using murine models. Initial experiments employed alkyne probe **1**, which was administered via transcardial perfusion to ensure a systemic distribution (Figure 4E). Mice were subsequently subjected to targeted IR irradiation at 1064 nm for 15 min, with the laser precisely focused on the anatomical region corresponding to the liver to achieve optimal photochemical activation. Following treatment, hepatic tissues were surgically isolated, fixed in 4% paraformaldehyde (PFA) to preserve the cellular architecture, and subjected to click chemistry with Cy5 azide overnight under standard conditions. Unreacted probe and excess Cy5 fluorophore were removed through extensive phosphate-buffered saline (PBS) washing protocols to minimize the background signal (Figure 4E).

Quantitative fluorescence imaging using an IVIS Spectrum imaging system revealed that hepatic tissues from IR-irradiated animals exhibited a statistically significant increase in radiant efficiency [(p/sec/cm^2^/sr)/(μW/cm^2^)] compared to nonirradiated control animals (Figure 4E). This robust signal enhancement demonstrates TagC-RED’s capability for precise spatiotemporal control of bioconjugation within complex *in vivo* environments, expanding the potential therapeutic and diagnostic applications.

To gauge the stability of the disulfide linkage formed through TagC-RED, we dissolved the TagC-RED-labeled Cys **14** in water, 1× PBS buffer, and 1× MEM, all with added glutathione (30 mM), a reducing agent commonly existing in cells. Compound **14** demonstrated excellent stability in all three solutions over a day, as observed by LCMS with internal standards (Figure 5A). We attribute this stability to the absence of excess anionic sulfide reagents, such as 2-mercaptopyridine (p*K*_a_ = −1.38),^57^ which is present in traditional disulfide ligation methods and commonly destabilizes disulfide linkages formed via disulfide and selenylsulfide exchange.^44,45^

**Figure 5:**
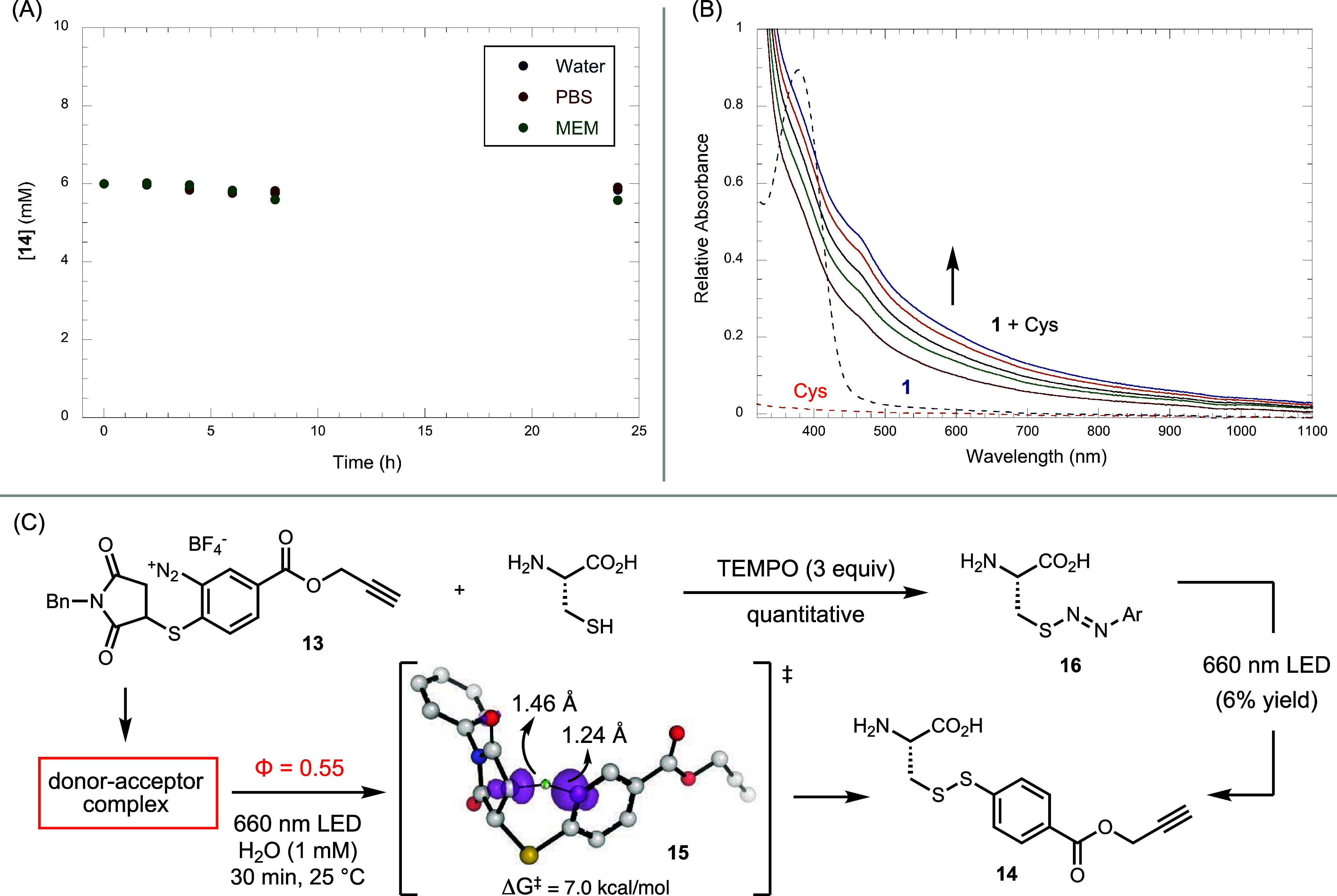
Studies of the stability of the disulfide linkage and the mechanism. (A) Stability test of **14** (6 mM) in H_2_O, 1× PBS, or 1× MEM, all three with added glutathione (30 mM). (B) UV–vis studies of the interaction between **1** and Cys: Cys = 1, 2, 3, 4, and 5 equiv relative to [**1**]. (C) Quantum yield and control experiments.

To probe the mechanism of TagC-RED, we performed UV–vis spectroscopic studies on the interaction between Cys and diazonium compound **1** (Figure 5B). While Cys showed no significant absorption above 240 nm, compound **1** exhibited a maximum absorption at 400 nm and no absorption above 500 nm. Upon mixing **1** and Cys, the spectrum displayed a notable red shift, indicating an interaction between Cys and **1**, potentially forming an EDA complex.

In a control experiment, we tested the possibility of nucleophilic addition product **16** as a potential intermediate in the reaction.^58^ We added 3 equiv of TEMPO in the reaction of **13** with Cys, which led to the complete conversion of Cys to the nucleophilic addition product diazosulfide **16**. When we isolated **16** and subjected it to the standard photolysis conditions, only 6% of **16** converted into disulfide **14**, indicating that **16** is not an intermediate in this reaction (Figure 5C). Furthermore, we determined the quantum yield for the reaction between **13** and Cys to form **14** under 660 nm irradiation to be 0.55 (Figure 5C). This value, being less than 1, suggests that a chain mechanism is unlikely.^59^

Overall, the experimental data support a mechanism consistent with our initial hypothesis (Figures 1C and 5C): an EDA complex is formed between **13** and cysteine, as evidenced by the UV–vis studies. The absorption spectrum of the mixture extends beyond 1100 nm, providing the basis for photoexcitation by 1040 nm IR light. Upon irradiation, aryl radical formation triggers a retro-ene reaction to generate a thio-phenol radical intermediate, which then combines with a cysteine-derived radical to afford product **14**. Density functional theory (DFT) calculations in SMD water at the ωB97M-V/def2-TZVPD//ωB97X-D/6-31+G­(d) level of theory identified TS^‡^ **15** for the hydrogen atom transfer (HAT) step, with a kinetic barrier of 7.0 kcal/mol (ΔG^‡^) and a driving force of −20.9 kcal/mol (ΔG) (Figure S57). The low activation energy and strong driving force reflect the difference in bond strengths between the Csp^2^ and Csp^3^ C–H bonds, for which the predicted bond dissociation energy (BDE) difference is −18.1 kcal/mol.^60^ Contrary to our initial hypothesis of a concerted fragmentation process, the computations revealed a stepwise HAT mechanism with the formation of a stable maleimide-centered radical, followed by fast fragmentation with an activation barrier of 11.9 kcal/mol.

A key distinction of this reaction is the formation of an EDA complex that enables IR-triggered reactivity. To further investigate the nature of this EDA interaction, we conducted computational studies. DFT calculations revealed that deprotonation of the cysteine residue **17** precedes association with the diazonium compound **18** to form an EDA complex **19**, favoring the branched regioisomer (Figure 6a). Using an implicit solvation model, the calculations predicted the formation of an S–N covalent adduct **19**. However, upon inclusion of several explicit water molecules, better representing the strong, directional hydrogen bonding in protic solvents, the charge-separated EDA complex **20** became the preferred structure (by 3.2 kcal·mol^–1^), featuring nonbonding S–N distances of 3.18–3.70 Å. These results highlight the critical role of protic solvent stabilization in preventing ion-pair collapse and in promoting the formation of the EDA complex. To experimentally test this hypothesis, we performed the reaction between **1** and Boc-Cys-OMe in the polar aprotic solvent chloroform and observed no reactivity, aligning with the computational results (Figure S48).

**Figure 6:**
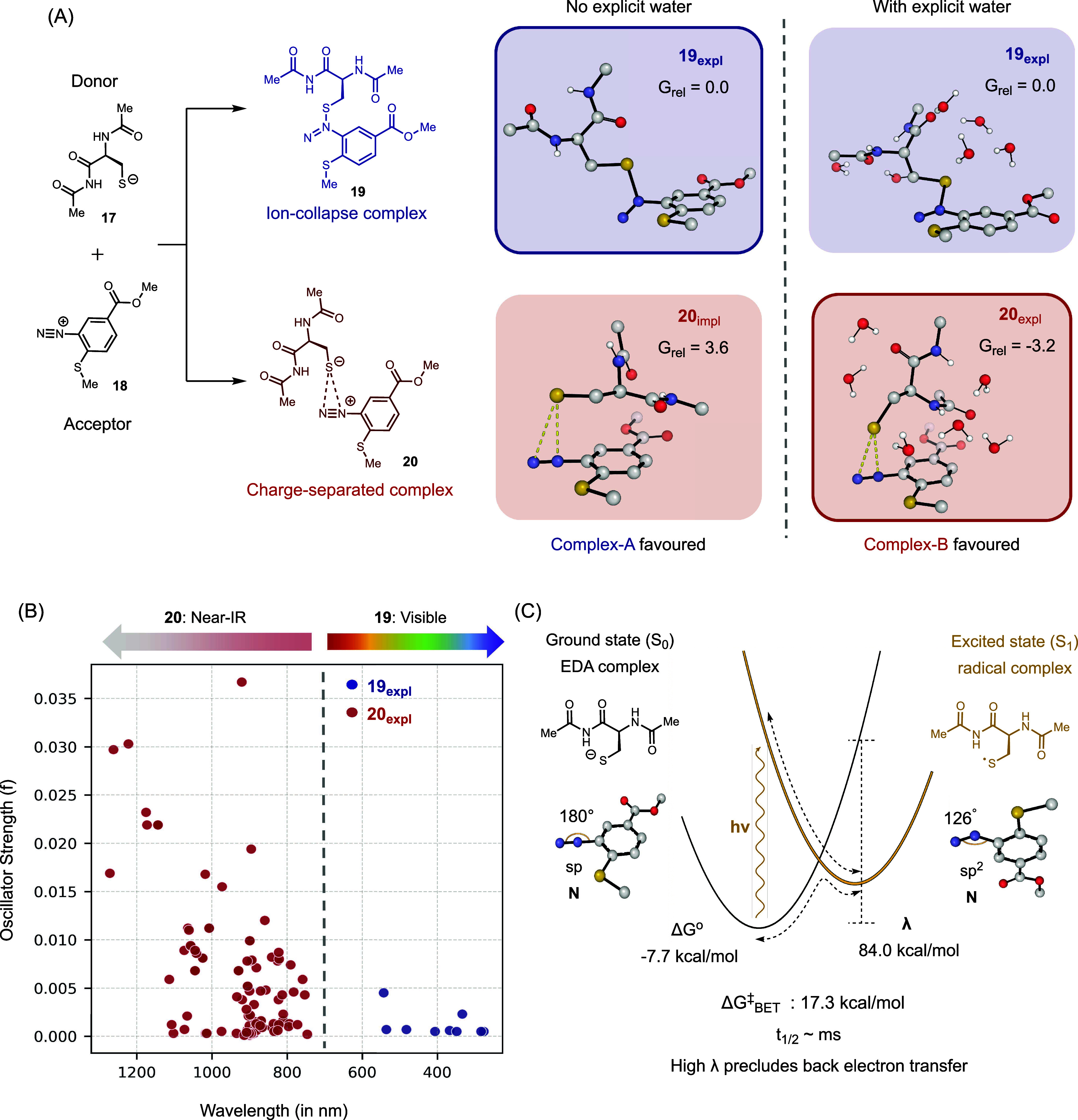
Computational modeling of donor–acceptor complex formation and photochemistry. (A) Two classes of donor–acceptor complexes are observed: ion-collapsed **19** and charge-separated **20**. Complex **20** is favored upon inclusion of explicit water solvent molecules, highlighting the role of hydrogen bonding in stabilizing charge separation. (B) Lowest-energy absorption wavelengths and oscillator strengths for various conformers of complexes **19** and **20**. Complex **19** absorbs predominantly in the visible region below 550 nm, while complex **20** exhibits near-IR absorption across its conformers. (C) Thermodynamic driving force (ΔG°) and reorganization energy (λ) associated with back electron transfer (BET) from the excited singlet state (S_1_) to the ground state (S_0_). The large λ leads to high activation barriers and a long half-life for the BET process.

Electronic S_0_ to S_1_ excitation in complex **20** is dominated by a (cysteine-localized) HOMO to (diazonium-localized) LUMO transition, with computationally predicted absorption features (from ΔSCF, see SI) extending deep into the near-IR region, reaching wavelengths of up to ∼1300 nm (Figure 6b). These data strongly implicate complex **20** as the putative EDA complex responsible for the observed near-IR reactivity, and its low vertical S_0_–S_1_ energy gap is responsible for efficient photoexcitation at wavelengths as long as 1064 nm. Consistent with this picture of photoinduced donor–acceptor charge transfer, spin- and charge-density analysis of the S_1_ state indicates that the donor and acceptor become neutral biradicaloid species with opposite spins in the excited state.

There is appreciable angle bending of the diazonium upon reduction, changing from 180° to 126°. This creates a large geometric reorganization energy (λ = 84.0 kcal·mol^–1^) that suppresses thermal back electron transfer: despite being exergonic, the computed Marcus barrier is around 17 kcal·mol^–1^. This corresponds to a BET lifetime on the order of milliseconds—a very slow decay relative to typical excited-state relaxations. Such a long lifetime allows the charge-separated radical pair to diffuse apart well before recombination can occur, consistent with the high quantum yield observed experimentally. This insight is particularly significant because high quantum yields are essential in biological environments, where photon flux is inherently limited. The long lifetime against back electron transfer therefore suggests that this system, and the underlying photochemistry, may be especially well suited for similar applications.

## Conclusions

We have developed TagC-RED, a photoactivated retro-ene reaction that achieves rapid, quantitative, and cysteine-specific bioconjugation in aqueous solutions within minutes without the need for a catalyst. Uniquely, TagC-RED is activated by 1040 nm infrared light, enabling deep tissue penetration and effective labeling in cells, *ex vivo* organs, and whole organisms *in vivo*. Mechanistic studies and DFT calculations revealed the formation of an EDA complex that enables IR excitation and the generation of radical species for highly selective cysteine bioconjugation. As a versatile platform for use in living systems, TagC-RED opens opportunities for precise protein labeling, real-time monitoring of dynamic processes, mapping of biomolecular interactions, and the development of next-generation diagnostic and therapeutic strategies.

## Supplementary Material





## Notes

Computational data: Thermochemical data and cartesian coordinates (PDF)
All experimental data, full Western blot images, detailed cell experiments, DFT calculations, and copies of spectra (PDF)
