# Supplementary material for: TagC-RED: An Infrared-Triggered Retro-Ene Reaction for Deep-Tissue Bioconjugation
Source: J Am Chem Soc. 2026 May 11;148(19):19738–49. doi: 10.1021/jacs.6c01581 (PMC13195677; doi:10.1021/jacs.6c01581)
Supplement: Supplementary file 1 [file ja6c01581_si_001.pdf]

Computational Data for

TagC-RED: An Infrared-Triggered Retro-Ene Reaction for Deep Tissue Bioconjugation

Sang Mi Suh,[1,§] Benjamin Ben-zvi,[1,§] John M. Talbott,[2] Niket Manoj,[3] Brock M. Nelson,[1] Riley R. Hughes,[2] Graham Haug,[3] Shohei Koide,[4] Robert S. Paton,[3] Monika Raj,[2,*] and Tianning Diao[1,*]

[1]Department of Chemistry, New York University, 100 Washington Square East, New York, NY 10003, United States. [2]Department of Chemistry, Emory University, 1515 Dickey Dr, Atlanta, GA 30322, United States. [3]Department of Chemistry, Colorado State University, 1301 Center Avenue Ft. Collins, CO 80523-1872, United States. [4]Department of Biochemistry and Molecular Pharmacology, New York University Grossman School of Medicine, and Perlmutter Cancer Center, New York University Langone Health, 522 1st Ave, Smilow Research Center, New York, NY 10016, United States.

[§]Authors contributed equally

*e-mail: diao@nyu.edu, monika.raj@emory.edu, and Robert.Paton@colstate.edu

1.0. Thermochemical data

The thermochemical information for all the reported structures and their conformers are given in table below

Legend:

E = energy obtained in the geometry optimizations

ZPE = zero-point energy

T·S = temperature times entropy with no correction

T.qh-S = temperature times entropy with quasi-harmonic S correction

G(T) = Gibbs free energy corrected only with ESPC

qh-G(T) = Gibbs free energy with ESPC and quasi-harmonic S correction

SPC = Single Point Correction

Naming Convention:

Anionic and neutral cysteine peptides are denoted cysteine_pep_anion and cysteine_pep_neu, respectively. Solvated structures include a suffix indicating the number of explicit water molecules (e.g., cysteine_pep_anion_6sol refers to the anionic cysteine peptide with six explicit waters). For Marcus calculations, the post-SET species structures are additionally labeled _ox and _red to indicate the oxidized and reduced electronic states as relevant.

Structure	E_SPC	E	ZPE	H_SPC	T.S	T.qh-S	G(T)_SPC	qh-G(T)_SPC
A_conf10	-1633.8849	-1633.4406	0.3070	-1633.5518	0.0853	0.0774	-1633.6371	-1633.6292
A_conf12	-1633.8870	-1633.4416	0.3074	-1633.5539	0.0828	0.0762	-1633.6367	-1633.6301
A_conf2	-1633.8840	-1633.4399	0.3067	-1633.5511	0.0871	0.0783	-1633.6382	-1633.6295
A_conf3	-1633.8871	-1633.4418	0.3070	-1633.5541	0.0839	0.0769	-1633.6380	-1633.6310
A_conf4	-1633.8868	-1633.4431	0.3072	-1633.5540	0.0809	0.0750	-1633.6349	-1633.6290
A_conf5	-1633.8890	-1633.4453	0.3076	-1633.5558	0.0814	0.0752	-1633.6371	-1633.6309
A_conf6	-1633.8893	-1633.4458	0.3078	-1633.5559	0.0805	0.0747	-1633.6364	-1633.6306
A_conf7	-1633.8869	-1633.4417	0.3071	-1633.5539	0.0837	0.0768	-1633.6376	-1633.6307
A_conf9	-1633.8897	-1633.4463	0.3073	-1633.5567	0.0821	0.0755	-1633.6388	-1633.6322
B_conf1	-1524.3560	-1523.9485	0.2970	-1524.0350	0.0793	0.0725	-1524.1143	-1524.1075
B_conf10	-1524.3585	-1523.9522	0.2976	-1524.0372	0.0776	0.0715	-1524.1148	-1524.1087
B_conf11	-1524.3548	-1523.9472	0.2974	-1524.0335	0.0788	0.0724	-1524.1123	-1524.1059
B_conf12	-1524.3561	-1523.9487	0.2970	-1524.0351	0.0797	0.0728	-1524.1148	-1524.1079
B_conf13	-1524.3561	-1523.9487	0.2970	-1524.0351	0.0798	0.0729	-1524.1149	-1524.1080
B_conf3	-1524.3592	-1523.9529	0.2973	-1524.0382	0.0775	0.0714	-1524.1157	-1524.1096
B_conf6	-1524.3549	-1523.9471	0.2972	-1524.0337	0.0798	0.0728	-1524.1135	-1524.1066
B_conf7	-1524.3547	-1523.9470	0.2973	-1524.0335	0.0789	0.0725	-1524.1124	-1524.1060
B_conf9	-1524.3584	-1523.9522	0.2972	-1524.0374	0.0788	0.0720	-1524.1162	-1524.1094
C_conf1	-1524.3851	-1523.9766	0.2967	-1524.0645	0.0792	0.0725	-1524.1436	-1524.1370
C_conf10	-1524.3911	-1523.9840	0.2967	-1524.0706	0.0793	0.0724	-1524.1500	-1524.1430
C_conf11	-1524.3851	-1523.9766	0.2968	-1524.0645	0.0786	0.0723	-1524.1431	-1524.1368
C_conf12	-1524.3848	-1523.9767	0.2966	-1524.0644	0.0789	0.0723	-1524.1432	-1524.1367
C_conf13	-1524.3848	-1523.9767	0.2966	-1524.0643	0.0789	0.0723	-1524.1432	-1524.1367
C_conf14	-1524.3850	-1523.9767	0.2967	-1524.0644	0.0788	0.0723	-1524.1433	-1524.1368
C_conf2	-1524.3851	-1523.9766	0.2967	-1524.0645	0.0792	0.0725	-1524.1436	-1524.1370
C_conf3	-1524.3911	-1523.9841	0.2967	-1524.0707	0.0777	0.0715	-1524.1484	-1524.1422
C_conf7	-1524.3848	-1523.9767	0.2965	-1524.0644	0.0793	0.0726	-1524.1437	-1524.1370
D_conf1	-933.8539	-933.6441	0.1442	-933.6969	0.0504	0.0485	-933.7473	-933.7454
D_conf2	-933.8539	-933.6441	0.1443	-933.6969	0.0505	0.0483	-933.7473	-933.7452
TS_1_conf1	-1633.8670	-1633.4202	0.3038	-1633.5367	0.0857	0.0780	-1633.6224	-1633.6147
TS_1_conf10	-1633.8720	-1633.4258	0.3047	-1633.5413	0.0825	0.0761	-1633.6239	-1633.6174
TS_1_conf12	-1633.8682	-1633.4213	0.3040	-1633.5378	0.0853	0.0778	-1633.6231	-1633.6156
TS_1_conf3	-1633.8732	-1633.4270	0.3042	-1633.5428	0.0834	0.0765	-1633.6262	-1633.6193
TS_1_conf7	-1633.8688	-1633.4208	0.3042	-1633.5382	0.0850	0.0778	-1633.6232	-1633.6160
TS_1_conf9	-1633.8722	-1633.4260	0.3045	-1633.5416	0.0834	0.0764	-1633.6249	-1633.6180
TS_2_conf1	-1524.3450	-1523.9369	0.2921	-1524.0298	0.0760	0.0704	-1524.1058	-1524.1002
TS_2_conf2	-1524.3451	-1523.9371	0.2924	-1524.0297	0.0755	0.0700	-1524.1052	-1524.0997
TS_2_conf3	-1524.3450	-1523.9370	0.2923	-1524.0297	0.0759	0.0702	-1524.1056	-1524.0999
TS_3_conf1	-1524.3698	-1523.9627	0.2959	-1524.0504	0.0766	0.0710	-1524.1270	-1524.1214
TS_3_conf11	-1524.3626	-1523.9551	0.2951	-1524.0436	0.0808	0.0733	-1524.1244	-1524.1168
TS_3_conf12	-1524.3629	-1523.9554	0.2953	-1524.0437	0.0799	0.0728	-1524.1236	-1524.1165
TS_3_conf13	-1524.3718	-1523.9648	0.2956	-1524.0527	0.0768	0.0711	-1524.1295	-1524.1238

TS_3_conf8	-1524.3626	-1523.9551	0.2949	-1524.0437	0.0828	0.0743	-1524.1264	-1524.1180
complexA_6sol_conf_10	-2358.7756	-2358.1025	0.5018	-2358.2265	0.1294	0.1174	-2358.3560	-2358.3439
complexA_6sol_conf_15	-2358.7828	-2358.1104	0.5033	-2358.2333	0.1262	0.1148	-2358.3596	-2358.3482
complexA_6sol_conf_21	-2358.7805	-2358.1074	0.5028	-2358.2310	0.1295	0.1166	-2358.3605	-2358.3476
complexA_6sol_conf_25	-2358.7799	-2358.1074	0.5019	-2358.2311	0.1298	0.1169	-2358.3609	-2358.3480
complexA_6sol_conf_26	-2358.7837	-2358.1117	0.5044	-2358.2337	0.1238	0.1134	-2358.3574	-2358.3471
complexA_6sol_conf_28	-2358.7837	-2358.1119	0.5044	-2358.2337	0.1236	0.1133	-2358.3573	-2358.3470
complexA_6sol_conf_3	-2358.7853	-2358.1139	0.5041	-2358.2358	0.1217	0.1120	-2358.3575	-2358.3478
complexA_6sol_conf_31	-2358.7816	-2358.1097	0.5024	-2358.2325	0.1281	0.1159	-2358.3607	-2358.3484
complexA_6sol_conf_36	-2358.7840	-2358.1126	0.5041	-2358.2342	0.1231	0.1132	-2358.3573	-2358.3474
complexA_6sol_conf_38	-2358.7846	-2358.1134	0.5050	-2358.2341	0.1214	0.1122	-2358.3555	-2358.3464
complexA_6sol_conf_44	-2358.7796	-2358.1070	0.5032	-2358.2302	0.1289	0.1157	-2358.3591	-2358.3459
complexA_6sol_conf_46	-2358.7735	-2358.1000	0.5013	-2358.2246	0.1329	0.1189	-2358.3576	-2358.3435
complexA_6sol_conf_51	-2358.7812	-2358.1084	0.5034	-2358.2317	0.1280	0.1151	-2358.3597	-2358.3468
complexA_6sol_conf_55	-2358.7829	-2358.1107	0.5044	-2358.2329	0.1247	0.1135	-2358.3576	-2358.3465
complexA_6sol_conf_57	-2358.7801	-2358.1089	0.5053	-2358.2299	0.1218	0.1118	-2358.3517	-2358.3417
complexA_6sol_conf_59	-2358.7825	-2358.1096	0.5039	-2358.2331	0.1239	0.1131	-2358.3570	-2358.3462
complexA_6sol_conf_6	-2358.7865	-2358.1154	0.5055	-2358.2361	0.1198	0.1108	-2358.3559	-2358.3470
complexA_6sol_conf_60	-2358.7699	-2358.0989	0.5027	-2358.2208	0.1251	0.1145	-2358.3459	-2358.3353
complexA_6sol_conf_7	-2358.7849	-2358.1128	0.5039	-2358.2354	0.1250	0.1134	-2358.3604	-2358.3489
complexA_6sol_conf_9	-2358.7878	-2358.1166	0.5056	-2358.2378	0.1211	0.1106	-2358.3589	-2358.3484
complexA_conf_1	-1900.0215	-1899.5782	0.3547	-1899.6387	0.0857	0.0796	-1899.7244	-1899.7183
complexA_conf_10	-1900.0126	-1899.5703	0.3549	-1899.6296	0.0848	0.0792	-1899.7144	-1899.7088
complexA_conf_11	-1900.0186	-1899.5752	0.3545	-1899.6357	0.0864	0.0801	-1899.7222	-1899.7158
complexA_conf_12	-1900.0215	-1899.5782	0.3538	-1899.6390	0.0867	0.0804	-1899.7258	-1899.7195
complexA_conf_13	-1900.0175	-1899.5744	0.3546	-1899.6347	0.0854	0.0795	-1899.7201	-1899.7142
complexA_conf_14	-1900.0176	-1899.5744	0.3548	-1899.6347	0.0850	0.0792	-1899.7196	-1899.7139
complexA_conf_15	-1900.0188	-1899.5764	0.3539	-1899.6362	0.0868	0.0804	-1899.7230	-1899.7166
complexA_conf_16	-1900.0132	-1899.5707	0.3549	-1899.6298	0.0858	0.0797	-1899.7156	-1899.7095
complexA_conf_17	-1900.0221	-1899.5793	0.3541	-1899.6395	0.0867	0.0802	-1899.7262	-1899.7197
complexA_conf_18	-1900.0227	-1899.5795	0.3537	-1899.6412	0.0845	0.0786	-1899.7257	-1899.7198
complexA_conf_2	-1900.0137	-1899.5711	0.3547	-1899.6305	0.0876	0.0805	-1899.7181	-1899.7110
complexA_conf_20	-1900.0127	-1899.5700	0.3538	-1899.6310	0.0859	0.0793	-1899.7169	-1899.7103

complexA_conf_22	-1900.0137	-1899.5711	0.3548	-1899.6305	0.0871	0.0803	-1899.7176	-1899.7107
complexA_conf_23	-1900.0184	-1899.5760	0.3542	-1899.6366	0.0838	0.0781	-1899.7204	-1899.7146
complexA_conf_24	-1900.0130	-1899.5706	0.3547	-1899.6301	0.0858	0.0796	-1899.7159	-1899.7097
complexA_conf_25	-1900.0172	-1899.5743	0.3542	-1899.6346	0.0862	0.0801	-1899.7208	-1899.7147
complexA_conf_26	-1900.0130	-1899.5702	0.3539	-1899.6303	0.0892	0.0814	-1899.7194	-1899.7117
complexA_conf_3	-1900.0179	-1899.5752	0.3538	-1899.6354	0.0878	0.0807	-1899.7232	-1899.7161
complexA_conf_4	-1900.0209	-1899.5776	0.3545	-1899.6381	0.0862	0.0799	-1899.7243	-1899.7180
complexA_conf_5	-1900.0179	-1899.5752	0.3547	-1899.6350	0.0861	0.0798	-1899.7211	-1899.7148
complexA_conf_6	-1900.0137	-1899.5712	0.3547	-1899.6307	0.0863	0.0798	-1899.7170	-1899.7105
complexA_conf_7	-1900.0165	-1899.5733	0.3544	-1899.6336	0.0875	0.0807	-1899.7212	-1899.7143
complexA_conf_8	-1900.0179	-1899.5755	0.3544	-1899.6351	0.0852	0.0793	-1899.7204	-1899.7144
complexA_conf_9	-1900.0175	-1899.5752	0.3535	-1899.6353	0.0881	0.0810	-1899.7234	-1899.7163
complexB_6sol_conf_1	-2358.7798	-2358.1025	0.5020	-2358.2306	0.1305	0.1177	-2358.3611	-2358.3483
complexB_6sol_conf_10	-2358.7791	-2358.1024	0.4999	-2358.2320	0.1308	0.1174	-2358.3628	-2358.3494
complexB_6sol_conf_11	-2358.7847	-2358.1085	0.4999	-2358.2411	0.1210	0.1100	-2358.3621	-2358.3511
complexB_6sol_conf_12	-2358.7827	-2358.1059	0.5008	-2358.2355	0.1279	0.1159	-2358.3633	-2358.3514
complexB_6sol_conf_14	-2358.7791	-2358.1023	0.4991	-2358.2325	0.1318	0.1182	-2358.3643	-2358.3507
complexB_6sol_conf_15	-2358.7784	-2358.1016	0.5002	-2358.2340	0.1212	0.1106	-2358.3553	-2358.3447
complexB_6sol_conf_16	-2358.7793	-2358.1025	0.4997	-2358.2339	0.1285	0.1152	-2358.3623	-2358.3490
complexB_6sol_conf_17	-2358.7819	-2358.1049	0.5013	-2358.2366	0.1218	0.1106	-2358.3585	-2358.3473
complexB_6sol_conf_18	-2358.7833	-2358.1074	0.5026	-2358.2351	0.1262	0.1144	-2358.3614	-2358.3495
complexB_6sol_conf_19	-2358.7753	-2358.0974	0.4990	-2358.2328	0.1246	0.1113	-2358.3574	-2358.3441
complexB_6sol_conf_2	-2358.7761	-2358.0985	0.4974	-2358.2344	0.1244	0.1120	-2358.3588	-2358.3464
complexB_6sol_conf_20	-2358.7828	-2358.1065	0.5008	-2358.2371	0.1260	0.1136	-2358.3630	-2358.3506
complexB_6sol_conf_21	-2358.7825	-2358.1060	0.5021	-2358.2337	0.1277	0.1158	-2358.3614	-2358.3494
complexB_6sol_conf_22	-2358.7796	-2358.1027	0.4989	-2358.2365	0.1230	0.1114	-2358.3595	-2358.3478
complexB_6sol_conf_23	-2358.7822	-2358.1065	0.5018	-2358.2371	0.1171	0.1081	-2358.3542	-2358.3452
complexB_6sol_conf_24	-2358.7896	-2358.1131	0.5024	-2358.2411	0.1251	0.1142	-2358.3662	-2358.3552
complexB_6sol_conf_25	-2358.7791	-2358.1024	0.5003	-2358.2328	0.1279	0.1154	-2358.3607	-2358.3481
complexB_6sol_conf_26	-2358.7822	-2358.1061	0.5030	-2358.2338	0.1220	0.1123	-2358.3558	-2358.3461
complexB_6sol_conf_27	-2358.7814	-2358.1039	0.5020	-2358.2333	0.1274	0.1152	-2358.3607	-2358.3485
complexB_6sol_conf_28	-2358.7765	-2358.0991	0.4992	-2358.2329	0.1234	0.1119	-2358.3563	-2358.3448

complexB_6sol_conf_29	-2358.7801	-2358.1033	0.4984	-2358.2383	0.1216	0.1100	-2358.3599	-2358.3483
complexB_6sol_conf_3	-2358.7774	-2358.1004	0.5019	-2358.2287	0.1297	0.1170	-2358.3584	-2358.3457
complexB_6sol_conf_30	-2358.7795	-2358.1025	0.4978	-2358.2380	0.1219	0.1105	-2358.3599	-2358.3484
complexB_6sol_conf_31	-2358.7801	-2358.1032	0.5010	-2358.2314	0.1319	0.1185	-2358.3633	-2358.3499
complexB_6sol_conf_32	-2358.7801	-2358.1033	0.5014	-2358.2313	0.1306	0.1177	-2358.3619	-2358.3490
complexB_6sol_conf_33	-2358.7792	-2358.1024	0.5002	-2358.2329	0.1259	0.1144	-2358.3588	-2358.3473
complexB_6sol_conf_34	-2358.7727	-2358.0962	0.4977	-2358.2322	0.1194	0.1085	-2358.3516	-2358.3407
complexB_6sol_conf_35	-2358.7823	-2358.1060	0.5037	-2358.2328	0.1243	0.1137	-2358.3571	-2358.3465
complexB_6sol_conf_36	-2358.7880	-2358.1125	0.5038	-2358.2391	0.1213	0.1120	-2358.3604	-2358.3510
complexB_6sol_conf_37	-2358.7754	-2358.0981	0.4994	-2358.2310	0.1261	0.1133	-2358.3571	-2358.3442
complexB_6sol_conf_38	-2358.7827	-2358.1066	0.5011	-2358.2371	0.1214	0.1112	-2358.3585	-2358.3483
complexB_6sol_conf_39	-2358.7760	-2358.0994	0.4987	-2358.2329	0.1242	0.1122	-2358.3571	-2358.3451
complexB_6sol_conf_4	-2358.7819	-2358.1061	0.5023	-2358.2338	0.1260	0.1143	-2358.3599	-2358.3482
complexB_6sol_conf_40	-2358.7794	-2358.1025	0.5007	-2358.2329	0.1266	0.1143	-2358.3596	-2358.3473
complexB_6sol_conf_41	-2358.7835	-2358.1072	0.5011	-2358.2361	0.1268	0.1153	-2358.3628	-2358.3514
complexB_6sol_conf_42	-2358.7803	-2358.1026	0.5016	-2358.2314	0.1323	0.1185	-2358.3637	-2358.3499
complexB_6sol_conf_44	-2358.7775	-2358.1004	0.4985	-2358.2336	0.1299	0.1152	-2358.3635	-2358.3489
complexB_6sol_conf_45	-2358.7749	-2358.0966	0.5004	-2358.2286	0.1267	0.1150	-2358.3553	-2358.3435
complexB_6sol_conf_46	-2358.7769	-2358.0997	0.4998	-2358.2334	0.1204	0.1100	-2358.3538	-2358.3434
complexB_6sol_conf_47	-2358.7810	-2358.1054	0.5049	-2358.2306	0.1227	0.1127	-2358.3533	-2358.3433
complexB_6sol_conf_48	-2358.7823	-2358.1073	0.5034	-2358.2341	0.1220	0.1114	-2358.3562	-2358.3455
complexB_6sol_conf_49	-2358.7833	-2358.1087	0.5033	-2358.2339	0.1250	0.1142	-2358.3589	-2358.3481
complexB_6sol_conf_5	-2358.7779	-2358.1014	0.5003	-2358.2335	0.1205	0.1104	-2358.3540	-2358.3439
complexB_6sol_conf_50	-2358.7837	-2358.1088	0.5039	-2358.2351	0.1212	0.1112	-2358.3563	-2358.3462
complexB_6sol_conf_51	-2358.7791	-2358.1036	0.5024	-2358.2313	0.1239	0.1130	-2358.3553	-2358.3443
complexB_6sol_conf_52	-2358.7754	-2358.0999	0.4995	-2358.2303	0.1252	0.1136	-2358.3555	-2358.3439
complexB_6sol_conf_53	-2358.7822	-2358.1067	0.5014	-2358.2352	0.1272	0.1145	-2358.3623	-2358.3496
complexB_6sol_conf_54	-2358.7750	-2358.0978	0.4990	-2358.2301	0.1281	0.1154	-2358.3582	-2358.3454
complexB_6sol_conf_55	-2358.7821	-2358.1081	0.5048	-2358.2321	0.1239	0.1126	-2358.3561	-2358.3447
complexB_6sol_conf_56	-2358.7793	-2358.1030	0.4993	-2358.2357	0.1214	0.1109	-2358.3571	-2358.3466
complexB_6sol_conf_57	-2358.7849	-2358.1111	0.5056	-2358.2346	0.1229	0.1119	-2358.3575	-2358.3465
complexB_6sol_conf_58	-2358.7774	-2358.1034	0.5024	-2358.2286	0.1268	0.1152	-2358.3554	-2358.3438

complexB_6sol_conf_59	-2358.7796	-2358.1034	0.5003	-2358.2328	0.1273	0.1156	-2358.3601	-2358.3484
complexB_6sol_conf_6	-2358.7880	-2358.1121	0.5024	-2358.2401	0.1266	0.1143	-2358.3666	-2358.3543
complexB_6sol_conf_60	-2358.7791	-2358.1035	0.5013	-2358.2333	0.1226	0.1114	-2358.3559	-2358.3447
complexB_6sol_conf_61	-2358.7792	-2358.1024	0.4991	-2358.2334	0.1318	0.1174	-2358.3652	-2358.3508
complexB_6sol_conf_62	-2358.7821	-2358.1058	0.5005	-2358.2343	0.1305	0.1176	-2358.3648	-2358.3519
complexB_6sol_conf_63	-2358.7838	-2358.1077	0.5006	-2358.2378	0.1241	0.1133	-2358.3619	-2358.3511
complexB_6sol_conf_64	-2358.7858	-2358.1109	0.5041	-2358.2362	0.1236	0.1128	-2358.3598	-2358.3490
complexB_6sol_conf_65	-2358.7816	-2358.1052	0.4984	-2358.2391	0.1233	0.1112	-2358.3624	-2358.3503
complexB_6sol_conf_66	-2358.7833	-2358.1072	0.5007	-2358.2354	0.1293	0.1171	-2358.3647	-2358.3525
complexB_6sol_conf_67	-2358.7783	-2358.1015	0.5029	-2358.2291	0.1271	0.1153	-2358.3562	-2358.3444
complexB_6sol_conf_68	-2358.7828	-2358.1074	0.5003	-2358.2377	0.1217	0.1114	-2358.3594	-2358.3491
complexB_6sol_conf_69	-2358.7885	-2358.1134	0.5052	-2358.2384	0.1194	0.1109	-2358.3579	-2358.3494
complexB_6sol_conf_7	-2358.7884	-2358.1119	0.5015	-2358.2403	0.1284	0.1162	-2358.3687	-2358.3564
complexB_6sol_conf_70	-2358.7766	-2358.1009	0.5008	-2358.2296	0.1296	0.1161	-2358.3591	-2358.3457
complexB_6sol_conf_71	-2358.7837	-2358.1089	0.5044	-2358.2340	0.1230	0.1125	-2358.3569	-2358.3465
complexB_6sol_conf_72	-2358.7844	-2358.1091	0.5047	-2358.2345	0.1224	0.1125	-2358.3568	-2358.3469
complexB_6sol_conf_73	-2358.7785	-2358.1016	0.5008	-2358.2313	0.1271	0.1157	-2358.3584	-2358.3470
complexB_6sol_conf_74	-2358.7797	-2358.1036	0.5021	-2358.2313	0.1277	0.1156	-2358.3590	-2358.3469
complexB_6sol_conf_75	-2358.7881	-2358.1141	0.5061	-2358.2386	0.1169	0.1082	-2358.3555	-2358.3468
complexB_6sol_conf_76	-2358.7878	-2358.1126	0.5035	-2358.2392	0.1218	0.1118	-2358.3610	-2358.3509
complexB_6sol_conf_77	-2358.7804	-2358.1042	0.4991	-2358.2347	0.1331	0.1178	-2358.3678	-2358.3525
complexB_6sol_conf_78	-2358.7805	-2358.1051	0.5039	-2358.2310	0.1232	0.1130	-2358.3542	-2358.3440
complexB_6sol_conf_79	-2358.7890	-2358.1140	0.5039	-2358.2401	0.1230	0.1119	-2358.3631	-2358.3521
complexB_6sol_conf_8	-2358.7796	-2358.1024	0.5006	-2358.2323	0.1295	0.1168	-2358.3618	-2358.3490
complexB_6sol_conf_80	-2358.7789	-2358.1020	0.5006	-2358.2339	0.1259	0.1128	-2358.3597	-2358.3466
complexB_6sol_conf_81	-2358.7712	-2358.0944	0.5004	-2358.2230	0.1315	0.1186	-2358.3545	-2358.3416
complexB_6sol_conf_82	-2358.7821	-2358.1060	0.5010	-2358.2339	0.1296	0.1171	-2358.3635	-2358.3511
complexB_6sol_conf_83	-2358.7830	-2358.1079	0.5044	-2358.2332	0.1268	0.1144	-2358.3600	-2358.3476
complexB_6sol_conf_84	-2358.7842	-2358.1088	0.5048	-2358.2350	0.1200	0.1103	-2358.3549	-2358.3453
complexB_6sol_conf_85	-2358.7823	-2358.1060	0.5003	-2358.2345	0.1316	0.1185	-2358.3661	-2358.3530
complexB_6sol_conf_86	-2358.7848	-2358.1106	0.5042	-2358.2362	0.1203	0.1104	-2358.3565	-2358.3465
complexB_6sol_conf_87	-2358.7833	-2358.1087	0.5039	-2358.2345	0.1206	0.1113	-2358.3551	-2358.3458

complexB_6sol_conf_88	-2358.7837	-2358.1088	0.5042	-2358.2341	0.1235	0.1129	-2358.3576	-2358.3470
complexB_6sol_conf_9	-2358.7889	-2358.1125	0.5043	-2358.2401	0.1206	0.1106	-2358.3607	-2358.3507
complexB_conf_1	-1900.0126	-1899.5663	0.3540	-1899.6299	0.0856	0.0801	-1899.7155	-1899.7100
complexB_conf_10	-1900.0045	-1899.5587	0.3539	-1899.6217	0.0870	0.0807	-1899.7087	-1899.7025
complexB_conf_12	-1900.0109	-1899.5644	0.3543	-1899.6281	0.0849	0.0797	-1899.7130	-1899.7078
complexB_conf_13	-1900.0157	-1899.5693	0.3543	-1899.6328	0.0848	0.0798	-1899.7176	-1899.7125
complexB_conf_15	-1900.0156	-1899.5691	0.3540	-1899.6325	0.0864	0.0809	-1899.7189	-1899.7134
complexB_conf_2	-1900.0100	-1899.5636	0.3540	-1899.6274	0.0855	0.0799	-1899.7129	-1899.7073
complexB_conf_20	-1900.0114	-1899.5648	0.3544	-1899.6286	0.0841	0.0794	-1899.7127	-1899.7079
complexB_conf_21	-1900.0151	-1899.5688	0.3531	-1899.6325	0.0881	0.0819	-1899.7206	-1899.7144
complexB_conf_22	-1900.0078	-1899.5613	0.3526	-1899.6256	0.0925	0.0838	-1899.7181	-1899.7093
complexB_conf_23	-1900.0009	-1899.5554	0.3533	-1899.6180	0.0915	0.0833	-1899.7094	-1899.7013
complexB_conf_25	-1900.0057	-1899.5594	0.3537	-1899.6230	0.0877	0.0810	-1899.7107	-1899.7041
complexB_conf_26	-1900.0061	-1899.5613	0.3535	-1899.6237	0.0875	0.0810	-1899.7113	-1899.7047
complexB_conf_27	-1900.0061	-1899.5612	0.3536	-1899.6237	0.0873	0.0808	-1899.7110	-1899.7045
complexB_conf_28	-1900.0118	-1899.5652	0.3534	-1899.6292	0.0884	0.0816	-1899.7176	-1899.7108
complexB_conf_29	-1900.0119	-1899.5654	0.3532	-1899.6293	0.0910	0.0829	-1899.7203	-1899.7122
complexB_conf_3	-1900.0060	-1899.5604	0.3528	-1899.6238	0.0910	0.0830	-1899.7147	-1899.7067
complexB_conf_30	-1900.0110	-1899.5648	0.3535	-1899.6282	0.0892	0.0820	-1899.7174	-1899.7102
complexB_conf_31	-1900.0116	-1899.5648	0.3533	-1899.6290	0.0891	0.0820	-1899.7181	-1899.7110
complexB_conf_32	-1900.0054	-1899.5586	0.3529	-1899.6229	0.0902	0.0828	-1899.7131	-1899.7057
complexB_conf_33	-1900.0130	-1899.5669	0.3539	-1899.6303	0.0861	0.0805	-1899.7164	-1899.7107
complexB_conf_34	-1900.0109	-1899.5644	0.3525	-1899.6305	0.0854	0.0792	-1899.7159	-1899.7097
complexB_conf_36	-1900.0033	-1899.5568	0.3532	-1899.6205	0.0901	0.0825	-1899.7106	-1899.7030
complexB_conf_39	-1900.0134	-1899.5668	0.3529	-1899.6312	0.0883	0.0817	-1899.7195	-1899.7130
complexB_conf_4	-1900.0078	-1899.5613	0.3525	-1899.6257	0.0919	0.0835	-1899.7176	-1899.7092
complexB_conf_41	-1900.0126	-1899.5660	0.3531	-1899.6303	0.0880	0.0815	-1899.7183	-1899.7118
complexB_conf_42	-1900.0102	-1899.5640	0.3534	-1899.6276	0.0884	0.0819	-1899.7160	-1899.7094
complexB_conf_44	-1900.0101	-1899.5640	0.3532	-1899.6277	0.0877	0.0813	-1899.7154	-1899.7090
complexB_conf_45	-1900.0102	-1899.5640	0.3534	-1899.6276	0.0884	0.0819	-1899.7160	-1899.7094
complexB_conf_46	-1900.0110	-1899.5649	0.3545	-1899.6280	0.0847	0.0796	-1899.7127	-1899.7076
complexB_conf_47	-1900.0121	-1899.5659	0.3543	-1899.6292	0.0857	0.0802	-1899.7150	-1899.7094

complexB_conf_48	-1900.0052	-1899.5593	0.3538	-1899.6220	0.0899	0.0826	-1899.7119	-1899.7045
complexB_conf_49	-1900.0125	-1899.5659	0.3531	-1899.6311	0.0853	0.0795	-1899.7163	-1899.7106
complexB_conf_5	-1900.0104	-1899.5647	0.3537	-1899.6275	0.0874	0.0814	-1899.7149	-1899.7089
complexB_conf_50	-1900.0112	-1899.5653	0.3530	-1899.6289	0.0892	0.0821	-1899.7181	-1899.7110
complexB_conf_51	-1900.0157	-1899.5693	0.3540	-1899.6329	0.0862	0.0804	-1899.7191	-1899.7133
complexB_conf_52	-1900.0130	-1899.5675	0.3548	-1899.6294	0.0856	0.0801	-1899.7150	-1899.7096
complexB_conf_53	-1900.0122	-1899.5657	0.3539	-1899.6292	0.0868	0.0809	-1899.7160	-1899.7101
complexB_conf_54	-1900.0096	-1899.5632	0.3532	-1899.6280	0.0862	0.0802	-1899.7142	-1899.7082
complexB_conf_55	-1900.0074	-1899.5611	0.3537	-1899.6244	0.0892	0.0820	-1899.7136	-1899.7064
complexB_conf_56	-1900.0015	-1899.5550	0.3546	-1899.6179	0.0883	0.0817	-1899.7062	-1899.6995
complexB_conf_57	-1900.0089	-1899.5629	0.3530	-1899.6263	0.0909	0.0830	-1899.7171	-1899.7093
complexB_conf_58	-1900.0108	-1899.5642	0.3538	-1899.6288	0.0846	0.0792	-1899.7133	-1899.7079
complexB_conf_59	-1900.0131	-1899.5675	0.3549	-1899.6295	0.0855	0.0800	-1899.7149	-1899.7095
complexB_conf_6	-1900.0111	-1899.5646	0.3539	-1899.6284	0.0875	0.0812	-1899.7158	-1899.7096
complexB_conf_60	-1900.0078	-1899.5613	0.3526	-1899.6256	0.0921	0.0835	-1899.7177	-1899.7092
complexB_conf_61	-1900.0126	-1899.5658	0.3535	-1899.6299	0.0879	0.0815	-1899.7177	-1899.7114
complexB_conf_62	-1900.0100	-1899.5641	0.3532	-1899.6275	0.0888	0.0819	-1899.7163	-1899.7094
complexB_conf_63	-1900.0111	-1899.5654	0.3532	-1899.6294	0.0857	0.0798	-1899.7151	-1899.7091
complexB_conf_64	-1900.0134	-1899.5671	0.3537	-1899.6307	0.0872	0.0811	-1899.7179	-1899.7119
complexB_conf_65	-1900.0086	-1899.5621	0.3527	-1899.6281	0.0842	0.0785	-1899.7124	-1899.7066
complexB_conf_66	-1900.0122	-1899.5665	0.3545	-1899.6288	0.0856	0.0802	-1899.7144	-1899.7090
complexB_conf_67	-1900.0098	-1899.5635	0.3530	-1899.6277	0.0878	0.0813	-1899.7155	-1899.7090
complexB_conf_68	-1900.0155	-1899.5690	0.3536	-1899.6326	0.0878	0.0817	-1899.7205	-1899.7143
complexB_conf_69	-1900.0110	-1899.5649	0.3546	-1899.6279	0.0847	0.0796	-1899.7126	-1899.7076
complexB_conf_7	-1900.0109	-1899.5652	0.3540	-1899.6277	0.0883	0.0815	-1899.7160	-1899.7092
complexB_conf_8	-1900.0063	-1899.5603	0.3543	-1899.6235	0.0855	0.0799	-1899.7090	-1899.7034
complexB_conf_9	-1900.0063	-1899.5603	0.3541	-1899.6236	0.0861	0.0803	-1899.7097	-1899.7039
complexC_conf_1	-1900.0083	-1899.5616	0.3536	-1899.6257	0.0870	0.0809	-1899.7127	-1899.7067
complexC_conf_10	-1900.0017	-1899.5553	0.3535	-1899.6188	0.0893	0.0823	-1899.7082	-1899.7011
complexC_conf_14	-1900.0044	-1899.5583	0.3536	-1899.6217	0.0877	0.0814	-1899.7093	-1899.7030
complexC_conf_18	-1900.0095	-1899.5631	0.3528	-1899.6273	0.0892	0.0822	-1899.7165	-1899.7095
complexC_conf_19	-1900.0044	-1899.5583	0.3536	-1899.6217	0.0877	0.0814	-1899.7093	-1899.7030

complexC_conf_2	-1900.0017	-1899.5552	0.3537	-1899.6188	0.0891	0.0821	-1899.7079	-1899.7009
complexC_conf_20	-1900.0060	-1899.5589	0.3537	-1899.6229	0.0896	0.0826	-1899.7126	-1899.7055
complexC_conf_21	-1900.0054	-1899.5607	0.3531	-1899.6228	0.0884	0.0821	-1899.7112	-1899.7049
complexC_conf_22	-1900.0058	-1899.5595	0.3533	-1899.6230	0.0905	0.0828	-1899.7136	-1899.7059
complexC_conf_23	-1900.0028	-1899.5565	0.3536	-1899.6199	0.0887	0.0818	-1899.7086	-1899.7018
complexC_conf_24	-1900.0026	-1899.5562	0.3540	-1899.6200	0.0886	0.0811	-1899.7086	-1899.7011
complexC_conf_25	-1900.0073	-1899.5608	0.3537	-1899.6242	0.0890	0.0821	-1899.7132	-1899.7063
complexC_conf_26	-1900.0032	-1899.5573	0.3540	-1899.6200	0.0888	0.0819	-1899.7088	-1899.7019
complexC_conf_27	-1900.0121	-1899.5658	0.3538	-1899.6291	0.0888	0.0818	-1899.7179	-1899.7110
complexC_conf_28	-1900.0085	-1899.5621	0.3534	-1899.6260	0.0870	0.0810	-1899.7131	-1899.7070
complexC_conf_29	-1900.0127	-1899.5665	0.3534	-1899.6300	0.0876	0.0815	-1899.7176	-1899.7115
complexC_conf_30	-1900.0112	-1899.5643	0.3538	-1899.6282	0.0876	0.0815	-1899.7158	-1899.7097
complexC_conf_4	-1900.0058	-1899.5595	0.3532	-1899.6231	0.0912	0.0832	-1899.7143	-1899.7062
complexC_conf_5	-1900.0099	-1899.5635	0.3535	-1899.6272	0.0891	0.0819	-1899.7163	-1899.7091
complexC_conf_6	-1900.0083	-1899.5618	0.3528	-1899.6268	0.0880	0.0811	-1899.7148	-1899.7079
complexC_conf_7	-1900.0094	-1899.5630	0.3532	-1899.6269	0.0892	0.0822	-1899.7161	-1899.7091
complexC_conf_9	-1900.0070	-1899.5608	0.3537	-1899.6240	0.0884	0.0819	-1899.7123	-1899.7058
cysteine_pep_anion_6sol_conf_10	-1352.4232	-1351.9899	0.3306	-1352.0634	0.0841	0.0797	-1352.1475	-1352.1431
cysteine_pep_anion_6sol_conf_12	-1352.4198	-1351.9856	0.3281	-1352.0612	0.0890	0.0827	-1352.1502	-1352.1439
cysteine_pep_anion_6sol_conf_15	-1352.4180	-1351.9838	0.3282	-1352.0592	0.0916	0.0839	-1352.1508	-1352.1431
cysteine_pep_anion_6sol_conf_17	-1352.4169	-1351.9819	0.3274	-1352.0581	0.0938	0.0857	-1352.1518	-1352.1438
cysteine_pep_anion_6sol_conf_18	-1352.4182	-1351.9828	0.3260	-1352.0603	0.0958	0.0871	-1352.1560	-1352.1474
cysteine_pep_anion_6sol_conf_21	-1352.4228	-1351.9883	0.3290	-1352.0636	0.0887	0.0826	-1352.1524	-1352.1462
cysteine_pep_anion_6sol_conf_22	-1352.4164	-1351.9813	0.3272	-1352.0578	0.0934	0.0854	-1352.1513	-1352.1432
cysteine_pep_anion_6sol_conf_25	-1352.4168	-1351.9816	0.3271	-1352.0582	0.0933	0.0857	-1352.1515	-1352.1439
cysteine_pep_anion_6sol_conf_26	-1352.4202	-1351.9849	0.3277	-1352.0614	0.0922	0.0850	-1352.1536	-1352.1464
cysteine_pep_anion_6sol_conf_28	-1352.4204	-1351.9857	0.3296	-1352.0610	0.0866	0.0811	-1352.1476	-1352.1421

cysteine_pep_anion_6sol_conf_5	-1352.4202	-1351.9865	0.3287	-1352.0610	0.0900	0.0830	-1352.1510	-1352.1441
cysteine_pep_anion_6sol_conf_6	-1352.4230	-1351.9900	0.3303	-1352.0633	0.0849	0.0801	-1352.1483	-1352.1434
cysteine_pep_anion_6sol_conf_7	-1352.4199	-1351.9850	0.3277	-1352.0610	0.0936	0.0856	-1352.1547	-1352.1467
cysteine_pep_anion_6sol_conf_8	-1352.4155	-1351.9802	0.3269	-1352.0573	0.0926	0.0851	-1352.1499	-1352.1424
cysteine_pep_anion_6sol_conf_9	-1352.4146	-1351.9795	0.3267	-1352.0564	0.0928	0.0853	-1352.1492	-1352.1417
cysteine_pep_anion_6sol_ox_conf_25	-1352.2354	-1351.8001	0.3274	-1351.8757	0.0958	0.0880	-1351.9715	-1351.9637
cysteine_pep_anion_6sol_ox_conf_7	-1352.2356	-1351.8007	0.3266	-1351.8766	0.0968	0.0882	-1351.9735	-1351.9648
cysteine_pep_anion_7sol_conf_1	-1428.8795	-1428.4076	0.3531	-1428.4929	0.0982	0.0896	-1428.5911	-1428.5825
cysteine_pep_anion_7sol_conf_10	-1428.8803	-1428.4079	0.3552	-1428.4923	0.0943	0.0875	-1428.5866	-1428.5798
cysteine_pep_anion_7sol_conf_13	-1428.8832	-1428.4119	0.3541	-1428.4962	0.0950	0.0878	-1428.5912	-1428.5840
cysteine_pep_anion_7sol_conf_17	-1428.8800	-1428.4082	0.3540	-1428.4932	0.0960	0.0881	-1428.5892	-1428.5813
cysteine_pep_anion_7sol_conf_20	-1428.8804	-1428.4075	0.3538	-1428.4933	0.0960	0.0887	-1428.5894	-1428.5820
cysteine_pep_anion_7sol_conf_21	-1428.8773	-1428.4047	0.3523	-1428.4911	0.0972	0.0898	-1428.5883	-1428.5808
cysteine_pep_anion_7sol_conf_26	-1428.8894	-1428.4177	0.3563	-1428.5016	0.0894	0.0841	-1428.5910	-1428.5857
cysteine_pep_anion_7sol_conf_27	-1428.8839	-1428.4123	0.3550	-1428.4963	0.0938	0.0869	-1428.5901	-1428.5832
cysteine_pep_anion_7sol_conf_30	-1428.8773	-1428.4037	0.3515	-1428.4913	0.1011	0.0918	-1428.5924	-1428.5831
cysteine_pep_anion_7sol_conf_5	-1428.8825	-1428.4113	0.3543	-1428.4953	0.0953	0.0877	-1428.5906	-1428.5830
cysteine_pep_anion_7sol_conf_6	-1428.8817	-1428.4103	0.3549	-1428.4944	0.0932	0.0865	-1428.5876	-1428.5809
cysteine_pep_anion_7sol_conf_7	-1428.8812	-1428.4083	0.3544	-1428.4940	0.0931	0.0870	-1428.5871	-1428.5810
cysteine_pep_anion_7sol_conf_8	-1428.8839	-1428.4125	0.3549	-1428.4966	0.0928	0.0864	-1428.5895	-1428.5830
cysteine_pep_anion_7sol_conf_9	-1428.8791	-1428.4070	0.3534	-1428.4925	0.0973	0.0890	-1428.5898	-1428.5815
cysteine_pep_anion_7sol_ox_conf_17	-1428.7017	-1428.2297	0.3542	-1428.3142	0.0955	0.0888	-1428.4097	-1428.4030

cysteine_pep_anion_7sol_ox_conf_21	-1428.6997	-1428.2268	0.3554	-1428.3108	0.0958	0.0890	-1428.4066	-1428.3998
cysteine_pep_anion_7sol_ox_conf_26	-1428.7014	-1428.2289	0.3550	-1428.3132	0.0960	0.0887	-1428.4092	-1428.4019
cysteine_pep_anion_7sol_ox_conf_30	-1428.6950	-1428.2215	0.3528	-1428.3075	0.1004	0.0921	-1428.4080	-1428.3997
cysteine_pep_anion_7sol_ox_conf_6	-1428.7027	-1428.2320	0.3560	-1428.3140	0.0964	0.0885	-1428.4103	-1428.4024
cysteine_pep_anion_7sol_ox_conf_8	-1428.7015	-1428.2299	0.3547	-1428.3135	0.0955	0.0888	-1428.4091	-1428.4023
cysteine_pep_anion_7sol_ox_conf_9	-1428.6972	-1428.2246	0.3529	-1428.3097	0.1004	0.0922	-1428.4101	-1428.4019
cysteine_pep_anion_8sol_conf_10	-1505.3429	-1504.8335	0.3785	-1504.9287	0.1015	0.0932	-1505.0302	-1505.0219
cysteine_pep_anion_8sol_conf_12	-1505.3449	-1504.8360	0.3799	-1504.9299	0.0987	0.0915	-1505.0286	-1505.0214
cysteine_pep_anion_8sol_conf_13	-1505.3455	-1504.8359	0.3816	-1504.9294	0.0971	0.0903	-1505.0266	-1505.0198
cysteine_pep_anion_8sol_conf_17	-1505.3430	-1504.8334	0.3795	-1504.9280	0.1002	0.0926	-1505.0282	-1505.0205
cysteine_pep_anion_8sol_conf_20	-1505.3457	-1504.8365	0.3813	-1504.9302	0.0971	0.0903	-1505.0272	-1505.0205
cysteine_pep_anion_8sol_conf_21	-1505.3398	-1504.8299	0.3792	-1504.9247	0.1035	0.0943	-1505.0282	-1505.0190
cysteine_pep_anion_8sol_conf_23	-1505.3395	-1504.8294	0.3794	-1504.9241	0.1040	0.0948	-1505.0281	-1505.0189
cysteine_pep_anion_8sol_conf_24	-1505.3404	-1504.8295	0.3793	-1504.9253	0.1010	0.0933	-1505.0263	-1505.0186
cysteine_pep_anion_8sol_conf_26	-1505.3489	-1504.8397	0.3830	-1504.9324	0.0940	0.0885	-1505.0264	-1505.0208
cysteine_pep_anion_8sol_conf_27	-1505.3409	-1504.8298	0.3772	-1504.9270	0.1050	0.0959	-1505.0319	-1505.0228
cysteine_pep_anion_8sol_conf_30	-1505.3375	-1504.8269	0.3786	-1504.9227	0.1021	0.0939	-1505.0248	-1505.0166
cysteine_pep_anion_8sol_conf_4	-1505.3476	-1504.8390	0.3811	-1504.9322	0.0958	0.0896	-1505.0280	-1505.0218
cysteine_pep_anion_8sol_conf_6	-1505.3471	-1504.8384	0.3808	-1504.9318	0.0973	0.0905	-1505.0291	-1505.0223
cysteine_pep_anion_8sol_conf_7	-1505.3422	-1504.8329	0.3795	-1504.9275	0.1002	0.0923	-1505.0277	-1505.0198
cysteine_pep_anion_8sol_conf_8	-1505.3444	-1504.8353	0.3806	-1504.9294	0.0962	0.0898	-1505.0256	-1505.0192
cysteine_pep_anion_8sol_ox_conf_26	-1505.1675	-1504.6577	0.3850	-1504.7487	0.0962	0.0899	-1504.8449	-1504.8385

cysteine_pep_anion_8sol_ox_conf_6	-1505.1668	-1504.6580	0.3802	-1504.7509	0.1008	0.0935	-1504.8517	-1504.8444
cysteine_pep_anion_8sol_ox_conf_7	-1505.1602	-1504.6503	0.3799	-1504.7443	0.1022	0.0945	-1504.8465	-1504.8388
cysteine_pep_anion_conf_10	-893.6482	-893.4442	0.1791	-893.4557	0.0503	0.0489	-893.5060	-893.5046
cysteine_pep_anion_conf_11	-893.6425	-893.4390	0.1794	-893.4498	0.0502	0.0486	-893.5000	-893.4983
cysteine_pep_anion_conf_2	-893.6454	-893.4417	0.1792	-893.4529	0.0499	0.0485	-893.5028	-893.5013
cysteine_pep_anion_conf_3	-893.6459	-893.4420	0.1788	-893.4535	0.0514	0.0493	-893.5048	-893.5028
cysteine_pep_anion_conf_5	-893.6452	-893.4412	0.1788	-893.4529	0.0502	0.0488	-893.5031	-893.5017
cysteine_pep_anion_conf_6	-893.6486	-893.4446	0.1789	-893.4560	0.0514	0.0494	-893.5074	-893.5054
cysteine_pep_anion_conf_8	-893.6450	-893.4415	0.1789	-893.4526	0.0509	0.0490	-893.5035	-893.5016
cysteine_pep_anion_ox_conf_1	-893.4781	-893.2739	0.1791	-893.2853	0.0517	0.0497	-893.3370	-893.3350
cysteine_pep_anion_ox_conf_10	-893.4799	-893.2755	0.1787	-893.2871	0.0535	0.0510	-893.3406	-893.3381
cysteine_pep_anion_ox_conf_11	-893.4704	-893.2661	0.1791	-893.2776	0.0521	0.0501	-893.3297	-893.3276
cysteine_pep_anion_ox_conf_2	-893.4773	-893.2731	0.1789	-893.2846	0.0520	0.0500	-893.3367	-893.3346
cysteine_pep_anion_ox_conf_6	-893.4804	-893.2761	0.1788	-893.2877	0.0537	0.0508	-893.3414	-893.3385
cysteine_pep_anion_ox_conf_8	-893.4772	-893.2730	0.1791	-893.2844	0.0524	0.0502	-893.3368	-893.3345
cysteine_pep_neu_conf_1	-894.1280	-893.9181	0.1888	-893.9249	0.0525	0.0503	-893.9774	-893.9753
cysteine_pep_neu_conf_10	-894.1235	-893.9136	0.1891	-893.9202	0.0519	0.0500	-893.9721	-893.9702
cysteine_pep_neu_conf_12	-894.1256	-893.9156	0.1889	-893.9223	0.0529	0.0507	-893.9752	-893.9730
cysteine_pep_neu_conf_13	-894.1234	-893.9134	0.1891	-893.9200	0.0523	0.0503	-893.9723	-893.9704
cysteine_pep_neu_conf_14	-894.1248	-893.9147	0.1882	-893.9218	0.0543	0.0517	-893.9761	-893.9735
cysteine_pep_neu_conf_15	-894.1244	-893.9146	0.1892	-893.9210	0.0518	0.0501	-893.9728	-893.9710
cysteine_pep_neu_conf_16	-894.1229	-893.9129	0.1891	-893.9196	0.0519	0.0502	-893.9715	-893.9697
cysteine_pep_neu_conf_17	-894.1270	-893.9170	0.1889	-893.9236	0.0531	0.0508	-893.9767	-893.9744
cysteine_pep_neu_conf_18	-894.1267	-893.9167	0.1888	-893.9235	0.0532	0.0508	-893.9766	-893.9743
cysteine_pep_neu_conf_19	-894.1273	-893.9173	0.1888	-893.9241	0.0527	0.0506	-893.9768	-893.9747
cysteine_pep_neu_conf_2	-894.1269	-893.9169	0.1886	-893.9237	0.0536	0.0510	-893.9773	-893.9748
cysteine_pep_neu_conf_20	-894.1213	-893.9114	0.1891	-893.9179	0.0522	0.0504	-893.9701	-893.9684
cysteine_pep_neu_conf_21	-894.1230	-893.9130	0.1891	-893.9197	0.0517	0.0501	-893.9714	-893.9698

cysteine_pep_neu_conf_23	-894.1225	-893.9128	0.1888	-893.9193	0.0530	0.0507	-893.9723	-893.9700
cysteine_pep_neu_conf_24	-894.1238	-893.9140	0.1892	-893.9204	0.0523	0.0502	-893.9727	-893.9706
cysteine_pep_neu_conf_25	-894.1225	-893.9126	0.1890	-893.9192	0.0532	0.0507	-893.9724	-893.9698
cysteine_pep_neu_conf_26	-894.1256	-893.9158	0.1890	-893.9225	0.0514	0.0497	-893.9739	-893.9722
cysteine_pep_neu_conf_3	-894.1269	-893.9168	0.1883	-893.9239	0.0541	0.0514	-893.9779	-893.9753
cysteine_pep_neu_conf_4	-894.1267	-893.9169	0.1885	-893.9237	0.0533	0.0509	-893.9770	-893.9746
cysteine_pep_neu_conf_5	-894.1268	-893.9168	0.1889	-893.9235	0.0529	0.0508	-893.9764	-893.9743
cysteine_pep_neu_conf_6	-894.1269	-893.9168	0.1883	-893.9239	0.0541	0.0515	-893.9780	-893.9753
cysteine_pep_neu_conf_7	-894.1267	-893.9169	0.1885	-893.9237	0.0533	0.0509	-893.9770	-893.9746
cysteine_pep_neu_conf_8	-894.1236	-893.9139	0.1891	-893.9202	0.0525	0.0503	-893.9727	-893.9705
cysteine_pep_neu_ox_conf_1	-893.8953	-893.6835	0.1896	-893.6917	0.0527	0.0505	-893.7445	-893.7422
cysteine_pep_neu_ox_conf_10	-893.8926	-893.6809	0.1899	-893.6889	0.0518	0.0498	-893.7408	-893.7388
cysteine_pep_neu_ox_conf_12	-893.8960	-893.6842	0.1897	-893.6925	0.0513	0.0499	-893.7438	-893.7424
cysteine_pep_neu_ox_conf_13	-893.8728	-893.6623	0.1889	-893.6699	0.0527	0.0506	-893.7226	-893.7205
cysteine_pep_neu_ox_conf_14	-893.8975	-893.6858	0.1898	-893.6940	0.0511	0.0498	-893.7451	-893.7438
cysteine_pep_neu_ox_conf_15	-893.8739	-893.6623	0.1875	-893.6721	0.0529	0.0512	-893.7249	-893.7233
cysteine_pep_neu_ox_conf_16	-893.8981	-893.6863	0.1896	-893.6946	0.0517	0.0499	-893.7463	-893.7445
cysteine_pep_neu_ox_conf_17	-893.8763	-893.6643	0.1870	-893.6747	0.0552	0.0522	-893.7298	-893.7268
cysteine_pep_neu_ox_conf_18	-893.9007	-893.6889	0.1894	-893.6975	0.0519	0.0501	-893.7494	-893.7475
cysteine_pep_neu_ox_conf_19	-893.8977	-893.6857	0.1898	-893.6941	0.0521	0.0501	-893.7461	-893.7441
cysteine_pep_neu_ox_conf_2	-893.8986	-893.6870	0.1894	-893.6953	0.0523	0.0503	-893.7476	-893.7456
cysteine_pep_neu_ox_conf_20	-893.8726	-893.6611	0.1873	-893.6708	0.0536	0.0513	-893.7244	-893.7222
cysteine_pep_neu_ox_conf_21	-893.9002	-893.6882	0.1895	-893.6969	0.0514	0.0498	-893.7484	-893.7468
cysteine_pep_neu_ox_conf_23	-893.8945	-893.6829	0.1901	-893.6909	0.0503	0.0488	-893.7412	-893.7397
cysteine_pep_neu_ox_conf_24	-893.8924	-893.6808	0.1902	-893.6887	0.0503	0.0492	-893.7389	-893.7378

cysteine_pep_neu_ox_conf_26	-893.8922	-893.6805	0.1900	-893.6885	0.0513	0.0496	-893.7398	-893.7381
cysteine_pep_neu_ox_conf_3	-893.8810	-893.6705	0.1891	-893.6779	0.0532	0.0508	-893.7311	-893.7286
cysteine_pep_neu_ox_conf_4	-893.8960	-893.6842	0.1897	-893.6925	0.0513	0.0499	-893.7438	-893.7424
cysteine_pep_neu_ox_conf_5	-893.8847	-893.6737	0.1892	-893.6818	0.0513	0.0497	-893.7331	-893.7315
cysteine_pep_neu_ox_conf_6	-893.8810	-893.6705	0.1893	-893.6778	0.0523	0.0504	-893.7301	-893.7282
maleimide	-590.5144	-590.3160	0.1506	-590.3530	0.0445	0.0433	-590.3974	-590.3962
n2	-109.5330	-109.4899	0.0057	-109.5240	0.0187	0.0187	-109.5427	-109.5427
s2_cation_trunc_conf_1	-1006.3483	-1006.1041	0.1730	-1006.1606	0.0533	0.0516	-1006.2139	-1006.2122
s2_cation_trunc_conf_2	-1006.3483	-1006.1043	0.1733	-1006.1604	0.0535	0.0516	-1006.2139	-1006.2120
s2_cation_trunc_conf_3	-1006.3422	-1006.0982	0.1734	-1006.1544	0.0522	0.0508	-1006.2066	-1006.2052
s2_cation_trunc_conf_4	-1006.3421	-1006.0981	0.1733	-1006.1542	0.0539	0.0516	-1006.2080	-1006.2057
s2_reduced_trunc_conf_1	-1006.5132	-1006.2757	0.1714	-1006.3269	0.0543	0.0525	-1006.3812	-1006.3794
s2_reduced_trunc_conf_2	-1006.5131	-1006.2757	0.1712	-1006.3270	0.0544	0.0526	-1006.3814	-1006.3796
s2_reduced_trunc_conf_3	-1006.5072	-1006.2707	0.1708	-1006.3213	0.0553	0.0533	-1006.3765	-1006.3745
s2_reduced_trunc_conf_4	-1006.5072	-1006.2706	0.1707	-1006.3213	0.0556	0.0534	-1006.3769	-1006.3747

2.0. Cartesian coordinates

42
A_conf10 Eopt -1633.440620
C -0.754505 -1.464736 -0.828369
C -1.503896 -2.141818 0.140994
C -2.726693 -1.654090 0.581305
C -3.228019 -0.474829 0.034746
C -2.499186 0.207782 -0.942579
C -1.267612 -0.281627 -1.362010
H -3.290795 -2.198287 1.331935
H -2.881945 1.126289 -1.372455
H -0.695120 0.264350 -2.104264
C -4.547259 0.016326 0.523289
O -5.205737 -0.539806 1.384928
O -4.936018 1.137979 -0.085021
C -6.202581 1.681706 0.338524
H -6.154060 1.917049 1.405224
H -6.987386 0.938453 0.174181
C -6.453024 2.884167 -0.447857

C -6.683983 3.882624 -1.084605
H -6.888502 4.769294 -1.651220
N -1.351957 -3.939020 1.627510
N -0.983709 -3.396459 0.647492
S 0.797420 -2.084833 -1.443115
C 1.893576 -1.938903 0.025120
C 3.309111 -2.381851 -0.352959
C 2.045011 -0.480060 0.415462
H 1.475382 -2.505973 0.857179
C 4.119490 -1.111166 -0.384027
H 3.392160 -2.897826 -1.313274
H 3.732968 -3.037891 0.413081
O 5.279435 -0.984266 -0.729425
O 1.198172 0.234921 0.916841
N 3.328213 -0.070016 0.088406
C 3.795501 1.272946 0.251866
C 3.973488 1.782805 1.535084
C 4.062894 2.040939 -0.878001
C 4.423557 3.092521 1.686460
H 3.760368 1.162115 2.400087
C 4.522819 3.345935 -0.716260
H 3.913406 1.619584 -1.867537
C 4.700511 3.872072 0.563362
H 4.561512 3.500751 2.683023
H 4.738260 3.951555 -1.591268
H 5.055194 4.891242 0.685459
42
A_conf12 Eopt -
1633.441587
C 1.174227 1.140014 -0.273748
C 2.096963 2.126687 0.135209
C 3.443826 1.837973 0.306198
C 3.921457 0.555197 0.062072
C 3.024936 -0.432779 -0.354337
C 1.677730 -0.144729 -0.517665
H 4.111716 2.628581 0.632332
H 3.370962 -1.440144 -0.556524
H 1.027585 -0.943653 -0.849520
C 5.369127 0.289120 0.254954
O 6.178528 1.127562 0.615068
O 5.700237 -0.977793 -0.009135
C 7.091512 -1.315940 0.154960
H 7.388633 -1.130324 1.190735
H 7.696144 -0.688603 -0.505685
C 7.247047 -2.726234 -0.183673
C 7.403041 -3.890840 -0.457712
H 7.539154 -4.925501 -0.702472
N 0.677548 3.985686 0.229209
N 1.734197 3.489122 0.420369
S -0.526961 1.570520 -0.464401
C -1.323426 -0.066105 -0.645560
C -1.267891 -0.996461 0.569522
C -2.807499 0.196324 -0.865423
H -0.959210 -0.552468 -1.555561
C -2.707390 -1.308719 0.886081
H -0.810752 -0.533211 1.448985
H -0.738579 -1.929649 0.363806
O -3.125277 -2.065992 1.741285
O -3.299900 0.946404 -1.685842
N -3.524143 -0.579975 0.026292
C -4.954784 -0.626772 0.055952
C -5.638586 -1.203683 -1.010717
C -5.631051 -0.095790 1.150738
C -7.030612 -1.244073 -0.979402
H -5.086589 -1.612912 -1.851511
C -7.022815 -0.150295 1.177216
H -5.073804 0.351823 1.968068
C -7.721801 -0.720961 0.113519
H -7.573304 -1.689090 -1.807774
H -7.559638 0.258014 2.028032
H -8.806930 -0.758053 0.136302
42
A_conf2 Eopt -
1633.439946
C 1.151031 0.764215 0.982041
C 1.729613 1.700041 0.116047
C 3.012272 1.527171 -0.386280
C 3.749052 0.407633 -0.006481
C 3.192624 -0.531320 0.865546
C 1.899101 -0.357089 1.344993
H 3.439977 2.269083 -1.053024
H 3.757825 -1.406475 1.165065
H 1.464913 -1.102204 2.003165
C 5.125989 0.259179 -0.555592
O 5.630743 1.034403 -1.348971
O 5.755621 -0.817949 -0.083595
C 7.096537 -1.030943 -0.569741
H 7.714483 -0.166413 -0.312363
H 7.072107 -1.139323 -1.657458
C 7.609126 -2.241808 0.061257
C 8.058050 -3.240073 0.568799
H 8.456880 -4.126306 1.020814
N 1.185675 3.612067 -1.113164
N 0.965283 2.887848 -0.209093
S -0.482450 0.966157 1.662935
C -1.477042 0.859272 0.126795
C -1.213776 -0.350677 -0.769806
C -2.940939 0.745208 0.511241
H -1.371778 1.794253 -0.431281
C -2.578744 -0.927818 -1.041500
H -0.617732 -1.125764 -0.276441
H -0.722030 -0.089565 -1.708774
O -2.860127 -1.828490 -1.809404
O -3.538048 1.424697 1.324669
N -3.508733 -0.267000 -0.243075
C -4.900913 -0.596497 -0.201295
C -5.832142 0.323541 -0.675468
C -5.294817 -1.828933 0.312673
C -7.186535 0.001433 -0.628290
H -5.498450 1.276921 -1.073914

C -6.650914 -2.146007 0.345566
H -4.549099 -2.527287 0.680324
C -7.595607 -1.232063 -0.121398
H -7.920528 0.713529 -0.992916
H -6.968131 -3.106084 0.741049
H -8.652127 -1.481367 -0.090124
42
A_conf3 Eopt -1633.441817
C 1.149670 1.045166 -0.264585
C 2.068569 2.052197 0.070094
C 3.423758 1.794251 0.213181
C 3.897321 0.499756 0.020906
C 2.998122 -0.515397 -0.322095
C 1.643480 -0.248507 -0.465402
H 4.101808 2.600041 0.475961
H 3.350611 -1.528144 -0.481671
H 0.987401 -1.064767 -0.739486
C 5.351801 0.249356 0.186368
O 6.166292 1.108953 0.478756
O 5.682784 -1.028214 -0.020195
C 7.080679 -1.349959 0.118802
H 7.410418 -1.099234 1.130722
H 7.659250 -0.762128 -0.599077
C 7.234017 -2.777905 -0.136658
C 7.389206 -3.956653 -0.342216
H 7.525196 -5.003894 -0.526058
N 2.153248 4.352489 0.506274
N 1.531296 3.380769 0.261045
S -0.546852 1.516214 -0.422376
C -1.379292 -0.096489 -0.615039
C -1.360604 -1.029210 0.600195
C -2.855146 0.193515 -0.857906
H -1.008117 -0.590286 -1.518391
C -2.810418 -1.304339 0.902718
H -0.899069 -0.579685 1.484332
H -0.854405 -1.975988 0.397406
O -3.256467 -2.045563 1.757821
O -3.321798 0.947366 -1.689795
N -3.599570 -0.561891 0.029023
C -5.031181 -0.569597 0.047064
C -5.722858 -1.137750 -1.019130
C -5.700524 -0.008187 1.130920
C -7.115727 -1.137818 -0.998617
H -5.176450 -1.571339 -1.851294
C -7.093392 -0.022314 1.146723
H -5.136954 0.431986 1.947958
C -7.800140 -0.583707 0.083205
H -7.664538 -1.575636 -1.826795
H -7.624821 0.410452 1.988778
H -8.886048 -0.589153 0.097369
42
A_conf4 Eopt -1633.443145
C -1.747759 -1.333893 -0.958407
C -1.485557 -2.233268 0.090381
C -0.183399 -2.593280 0.413057
C 0.885342 -2.041698 -0.288424
C 0.641662 -1.208328 -1.379114
C -0.665292 -0.882408 -1.718945
H -0.010645 -3.285133 1.231235
H 1.461656 -0.800287 -1.958847
H -0.847251 -0.233401 -2.568475
C 2.267119 -2.325647 0.188599
O 2.556284 -3.209897 0.975369
O 3.153536 -1.467841 -0.314814
C 4.510593 -1.599164 0.151475
H 4.914231 -2.561028 -0.176323
H 4.520754 -1.562549 1.244272
C 5.273584 -0.489777 -0.409674
C 5.915267 0.428374 -0.857669
H 6.484842 1.243297 -1.258197
N -3.616222 -2.600399 1.053332
N -2.498680 -2.926170 0.862633
S -3.374928 -0.716740 -1.369758
C -3.382544 0.858629 -0.412135
C -3.345191 0.729726 1.109714
C -2.147613 1.685072 -0.745704
H -4.272755 1.377889 -0.774307
C -1.966272 1.183015 1.504942
H -3.535384 -0.275180 1.487148
H -4.067746 1.406945 1.577020
O -1.453579 1.114185 2.606433
O -1.865525 2.180074 -1.819027
N -1.352335 1.753469 0.390914
C -0.014087 2.260107 0.392279
C 0.232643 3.577486 0.016490
C 1.025391 1.405965 0.755870
C 1.547728 4.038746 -0.010095
H -0.591091 4.230852 -0.252687
C 2.334504 1.878697 0.735566
H 0.809150 0.383945 1.048856
C 2.597218 3.192563 0.347290
H 1.749106 5.063332 -0.307700
H 3.148814 1.216319 1.014768
H 3.620104 3.556864 0.324531
42
A_conf5 Eopt -1633.445268
C -0.970489 -1.968487 0.526836
C -0.449666 -2.285411 -0.733441
C 0.871716 -2.008541 -1.063622
C 1.694471 -1.390451 -0.126931
C 1.199155 -1.084916 1.143342
C -0.115543 -1.391764 1.470554
H 1.245097 -2.247547 -2.054177
H 1.835382 -0.614376 1.884081
H -0.473654 -1.181476 2.472567
C 3.089732 -1.058976 -0.532546
O 3.558700 -1.312175 -1.628242

O 3.773950 -0.446212 0.433905
C 5.133310 -0.085928 0.115072
H 5.134881 0.582956 -0.749883
H 5.696293 -0.989769 -0.133415
C 5.701432 0.575758 1.284005
C 6.195085 1.126844 2.237034
H 6.633235 1.615412 3.084461
N -0.987322 -3.457794 -2.679300
N -1.338012 -2.869684 -1.719940
S -2.677301 -2.322089 0.923633
C -3.384402 -0.625300 1.016022
C -3.032599 0.202076 2.253831
C -2.891767 0.216606 -0.151520
H -4.459505 -0.787201 0.911368
C -2.095401 1.267531 1.750664
H -2.576908 -0.359454 3.070482
H -3.923025 0.704367 2.647532
O -1.432424 2.047151 2.408870
O -3.100714 0.030057 -1.334553
N -2.118445 1.248023 0.357967
C -1.354146 2.142091 -0.456009
C -1.666457 3.498157 -0.470352
C -0.297397 1.633160 -1.206164
C -0.898488 4.360377 -1.250408
H -2.496480 3.870943 0.122053
C 0.459682 2.500814 -1.988656
H -0.075452 0.571340 -1.176514
C 0.161534 3.863533 -2.008908
H -1.131775 5.420604 -1.266342
H 1.284493 2.110891 -2.577480
H 0.756017 4.539318 -2.616594
42
A_conf6 Eopt -1633.445813
C -0.717534 -2.095982 -0.249070
C -0.412824 -1.477159 -1.468720
C 0.833640 -0.912304 -1.703199
C 1.802111 -0.954672 -0.703884
C 1.522336 -1.575128 0.515153
C 0.273666 -2.146311 0.733928
H 1.045854 -0.441826 -2.657810
H 2.273336 -1.621241 1.295493
H 0.071159 -2.638485 1.678974
C 3.114702 -0.303743 -0.977443
O 3.400521 0.235071 -2.032229
O 3.945600 -0.365531 0.063385
C 5.236433 0.250894 -0.119429
H 5.099467 1.308205 -0.362169
H 5.755356 -0.239662 -0.947318
C 5.980251 0.096693 1.125539
C 6.614495 -0.017206 2.145483
H 7.177258 -0.118619 3.052036
N -1.439016 -0.728034 -3.425919
N -1.413000 -1.482245 -2.521052
S -2.331221 -2.805830 0.058714
C -3.134371 -1.443228 1.000910
C -2.532697 -1.129274 2.370628
C -3.008861 -0.137583 0.231606
H -4.184047 -1.739929 1.051248
C -1.790637 0.166627 2.175304
H -1.871164 -1.897720 2.772773
H -3.324388 -0.949083 3.105964
O -1.025167 0.708965 2.950451
O -3.517111 0.139833 -0.836597
N -2.157099 0.697674 0.940536
C -1.632069 1.915996 0.405582
C -2.486241 2.981829 0.139674
C -0.267169 1.996089 0.140410
C -1.959604 4.148253 -0.412020
H -3.546152 2.897282 0.358637
C 0.250901 3.168510 -0.402910
H 0.377116 1.150433 0.359101
C -0.594554 4.242564 -0.682449
H -2.618216 4.983960 -0.627921
H 1.314094 3.238320 -0.612486
H -0.188907 5.153990 -1.111540
42
A_conf7 Eopt -1633.441696
C 1.066735 1.467809 0.065946
C 2.148044 2.198097 -0.451912
C 3.418799 1.653710 -0.565306
C 3.639184 0.342712 -0.154822
C 2.573303 -0.401975 0.361735
C 1.303485 0.148562 0.467193
H 4.228839 2.252260 -0.969982
H 2.726260 -1.426674 0.681300
H 0.507661 -0.474044 0.855546
C 5.008125 -0.216701 -0.289323
O 5.962087 0.404522 -0.727414
O 5.091643 -1.484942 0.121610
C 6.391924 -2.097820 0.017476
H 7.107114 -1.532839 0.621554
H 6.716976 -2.079697 -1.026253
C 6.275351 -3.469457 0.499991
C 6.206855 -4.608289 0.892565
H 6.144740 -5.620134 1.240620
N 2.647982 4.310351 -1.335804
N 1.874120 3.557860 -0.860364
S -0.489987 2.306159 0.158125
C -1.583965 1.140084 1.017916
C -2.779658 1.862692 1.648295
C -2.214019 0.094110 0.107816
H -1.025136 0.612636 1.796938
C -3.955080 0.967245 1.353434
H -2.988469 2.832190 1.180545
H -2.671742 2.017070 2.722590
O -5.079232 1.052236 1.810887
O -1.661139 -0.578118 -0.740090
N -3.560519 0.008561 0.425771

C -4.450546 -0.947235 -0.160240
C -4.265994 -2.301714 0.103984
C -5.481305 -0.502677 -0.983887
C -5.130744 -3.228317 -0.473975
H -3.455926 -2.622954 0.751849
C -6.347457 -1.436295 -1.548306
H -5.601558 0.558864 -1.178248
C -6.171603 -2.796924 -1.296326
H -4.992631 -4.286995 -0.276574
H -7.156439 -1.098910 -2.189044
H -6.846462 -3.521994 -1.741525
42
A_conf9 Eopt -1633.446277
C -1.691012 -1.332411 -1.069431
C -1.421739 -2.139731 0.042404
C -0.117279 -2.451059 0.409639
C 0.940580 -1.947351 -0.339949
C 0.690196 -1.189119 -1.485809
C -0.617198 -0.900034 -1.852856
H 0.069926 -3.051947 1.293927
H 1.510015 -0.807250 -2.083424
H -0.806843 -0.303071 -2.738013
C 2.324310 -2.162966 0.168935
O 2.622552 -2.974383 1.027404
O 3.197969 -1.327813 -0.391229
C 4.546248 -1.377826 0.114779
H 4.987643 -2.348768 -0.126495
H 4.526956 -1.255623 1.201231
C 5.289825 -0.292025 -0.514173
C 5.920171 0.604010 -1.019466
H 6.478471 1.400037 -1.470593
N -2.458980 -3.331840 1.765800
N -2.543123 -2.634749 0.818843
S -3.344715 -0.802297 -1.503164
C -3.445646 0.777522 -0.557593
C -3.513520 0.646770 0.963590
C -2.202384 1.619302 -0.805399
H -4.316531 1.281782 -0.981915
C -2.170258 1.118856 1.453777
H -3.716258 -0.362527 1.325326
H -4.276110 1.313268 1.380050
O -1.731497 1.054233 2.586848
O -1.850640 2.116547 -1.856931
N -1.488651 1.696573 0.383532
C -0.152877 2.203171 0.468221
C 0.115409 3.524597 0.123602
C 0.864390 1.341266 0.873154
C 1.431339 3.982124 0.171399
H -0.691844 4.183202 -0.180620
C 2.174167 1.809434 0.926152
H 0.630051 0.316166 1.140299
C 2.459247 3.127799 0.569966
H 1.650531 5.009797 -0.101923
H 2.971044 1.139866 1.237136
H 3.482794 3.489280 0.604024
40
B_conf1 Eopt -1523.948545
C -1.518654 0.497731 -0.500563
C -2.742400 1.108381 -0.555418
C -3.856154 0.323061 -0.215426
C -3.672849 -1.014424 0.160851
C -2.404814 -1.579398 0.203215
C -1.277752 -0.809918 -0.138428
H -2.859526 2.147976 -0.847277
H -4.528255 -1.625421 0.426312
H -2.287559 -2.617996 0.501297
C -5.199235 0.951631 -0.272199
O -5.402642 2.110306 -0.597425
O -6.181589 0.111919 0.071693
C -7.516264 0.653387 0.035363
H -7.740713 0.993075 -0.979514
H -7.579541 1.506348 0.716678
S 0.325592 -1.551984 -0.092145
C 1.356040 -0.114523 -0.536685
C 1.433189 1.018442 0.491049
H 1.028633 0.252724 -1.515243
H 0.943030 0.777378 1.439182
H 1.010425 1.953992 0.117328
N 3.612933 0.244734 0.035286
C 2.792182 -0.588677 -0.702829
O 3.179529 -1.520573 -1.381413
C 2.905333 1.205888 0.752013
O 3.427037 2.037523 1.470429
C 5.038996 0.120507 0.067595
C 5.820575 1.107952 -0.525751
C 5.613226 -0.982765 0.693021
C 7.207829 0.986695 -0.487234
H 5.347168 1.957138 -1.009072
C 7.001147 -1.099751 0.717752
H 4.980391 -1.736703 1.151477
C 7.797330 -0.116047 0.130963
H 7.826528 1.752708 -0.944686
H 7.459091 -1.957963 1.199903
H 8.879043 -0.209089 0.155597
C -8.435134 -0.403670 0.443211
C -9.217143 -1.259209 0.778337
H -9.910074 -2.020722 1.076056
40
B_conf10 Eopt -1523.952229
C -0.288393 -2.203850 -1.465247
C 0.987639 -1.779374 -1.732361
C 1.759755 -1.397373 -0.623698
C 1.228787 -1.481980 0.668529
C -0.067617 -1.943380 0.876225
C -0.868958 -2.297866 -0.220744
H 1.386943 -1.718940 -2.740129
H 1.829268 -1.194229 1.523820

H -0.454223 -2.022957 1.888033
C 3.137028 -0.888906 -0.877630
O 3.651555 -0.834818 -1.981493
O 3.753701 -0.487000 0.235696
C 5.091249 0.024544 0.068586
H 5.068468 0.877247 -0.615619
H 5.724920 -0.757194 -0.359232
S -2.560896 -2.859029 -0.066666
C -3.418374 -1.298098 0.392183
C -3.211962 -0.802552 1.825911
H -4.468642 -1.495585 0.165087
H -2.720968 -1.516335 2.489579
H -4.165735 -0.518969 2.283176
N -2.305597 0.777091 0.339264
C -2.932157 -0.149339 -0.478751
O -3.037830 -0.042603 -1.685511
C -2.377911 0.443124 1.688703
O -1.854390 1.089827 2.577118
C -1.593742 1.915695 -0.154468
C -2.087308 3.194430 0.085375
C -0.407292 1.714913 -0.854417
C -1.372426 4.293871 -0.385804
H -3.015011 3.324423 0.634353
C 0.296830 2.818905 -1.327644
H -0.044937 0.706116 -1.024141
C -0.184039 4.107176 -1.092076
H -1.747066 5.296297 -0.202518
H 1.222486 2.670384 -1.875539
H 0.368510 4.967200 -1.458789
C 5.578414 0.427725 1.382824
C 6.004429 0.770436 2.458349
H 6.381495 1.075517 3.414175
40
B_conf11 Eopt -
1523.947157
C -2.088056 -2.255660 0.363870
C -3.426162 -2.093837 0.585678
C -3.983917 -0.863039 0.194432
C -3.167955 0.110516 -0.390004
C -1.809457 -0.114130 -0.596034
C -1.231720 -1.334056 -0.207615
H -4.038625 -2.864644 1.044136
H -3.590318 1.060945 -0.696343
H -1.216207 0.665740 -1.061815
C -5.435577 -0.649962 0.420556
O -6.178468 -1.473650 0.929243
O -5.857276 0.548823 0.004842
C -7.260432 0.822878 0.186055
H -7.508345 0.754245 1.248816
H -7.847459 0.081525 -0.363035
S 0.462446 -1.801684 -0.425279
C 1.272038 -0.186268 -0.687763
C 1.188990 0.812637 0.469752
H 0.916793 0.247487 -1.627156
H 0.705981 0.405280 1.362715
H 0.668166 1.732427 0.193118
N 3.456685 0.369768 0.000487
C 2.759949 -0.447743 -0.870685
O 3.271864 -1.221368 -1.656988
C 2.620732 1.136227 0.807855
O 3.019913 1.921157 1.647118
C 4.886101 0.420436 0.061444
C 5.542477 1.580372 -0.341055
C 5.589028 -0.689249 0.522566
C 6.933062 1.628638 -0.274399
H 4.970658 2.431016 -0.699524
C 6.980034 -0.634793 0.575216
H 5.052704 -1.580743 0.833395
C 7.651115 0.522496 0.180187
H 7.454362 2.529875 -0.582338
H 7.537751 -1.496344 0.929475
H 8.735373 0.562549 0.226790
C -7.517760 2.166678 -0.319925
C -7.755773 3.275827 -0.730981
H -7.965350 4.261707 -1.095782
40
B_conf12 Eopt -
1523.948677
C -1.206722 -0.385564 0.756913
C -2.308420 0.336511 1.126418
C -3.490759 0.101764 0.404113
C -3.490127 -0.832235 -0.640024
C -2.337409 -1.532261 -0.974726
C -1.145614 -1.310718 -0.262294
H -2.282905 1.058351 1.937572
H -4.397360 -1.018136 -1.203758
H -2.361583 -2.247736 -1.792764
C -4.704696 0.867934 0.780278
O -4.747904 1.687109 1.684156
O -5.763703 0.572176 0.018917
C -6.977263 1.288376 0.319093
H -6.799618 2.361826 0.209830
H -7.274153 1.081782 1.351001
S 0.303726 -2.227275 -0.699028
C 1.534687 -1.562625 0.460180
C 2.714778 -2.514291 0.672819
H 1.033996 -1.349428 1.411848
H 2.800894 -3.279304 -0.107037
H 2.686578 -3.019069 1.639851
N 3.541205 -0.371964 0.147833
C 2.168784 -0.260948 -0.008051
O 1.599251 0.731586 -0.417973
C 3.934829 -1.635345 0.575563
O 5.089223 -1.949767 0.797783
C 4.454270 0.699768 -0.111331
C 5.111628 1.306083 0.955616
C 4.664577 1.113642 -1.423755
C 6.001708 2.346562 0.698928
H 4.927558 0.965475 1.970077
C 5.549297 2.161394 -1.669345

H 4.140977 0.621855 -2.237943
C 6.218385 2.775581 -0.610613
H 6.521576 2.824153 1.523801
H 5.717339 2.494067 -2.689112
H 6.909843 3.589808 -0.806272
C -8.001641 0.835481 -0.615580
C -8.865851 0.478691 -1.378263
H -9.632783 0.160879 -2.056189
40
B_conf13 Eopt -1523.948684
C -1.204159 -0.348897 0.738143
C -2.303650 0.383321 1.094323
C -3.489465 0.131532 0.383646
C -3.494262 -0.828204 -0.636953
C -2.343509 -1.536876 -0.959701
C -1.148291 -1.298782 -0.258332
H -2.273809 1.125226 1.887042
H -4.404165 -1.027196 -1.191857
H -2.371873 -2.272447 -1.759622
C -4.701308 0.907812 0.745564
O -4.735787 1.762653 1.616227
O -5.769604 0.576389 0.012376
C -6.983058 1.297575 0.300856
H -6.818494 2.364989 0.129497
H -7.255313 1.144814 1.348818
S 0.298246 -2.226064 -0.681424
C 1.530557 -1.554959 0.472508
C 2.705774 -2.510374 0.695862
H 1.030041 -1.330333 1.421613
H 2.790208 -3.282064 -0.077600
H 2.672921 -3.007088 1.666900
N 3.543354 -0.376628 0.154209
C 2.171934 -0.260904 -0.006928
O 1.608341 0.730166 -0.428426
C 3.930087 -1.637827 0.594309
O 5.082410 -1.955380 0.822729
C 4.462103 0.688551 -0.111798
C 5.117171 1.302902 0.951952
C 4.680375 1.087960 -1.427402
C 6.013165 2.336666 0.688805
H 4.926855 0.973568 1.968977
C 5.571002 2.129136 -1.679563
H 4.158364 0.590170 -2.238956
C 6.237938 2.751157 -0.624046
H 6.531376 2.820331 1.511178
H 5.745377 2.450501 -2.701888
H 6.934028 3.560186 -0.824777
C -8.023581 0.785150 -0.583903
C -8.902407 0.377851 -1.303369
H -9.682312 0.015401 -1.943097
40
B_conf3 Eopt -1523.952946
C -0.515937 -1.795124 1.073241
C 0.645991 -1.243468 1.541620
C 1.711329 -1.166019 0.628708
C 1.552668 -1.637703 -0.678547
C 0.335067 -2.164884 -1.101830
C -0.743125 -2.247780 -0.208181
H 0.752780 -0.880239 2.559196
H 2.374205 -1.574909 -1.383024
H 0.214081 -2.499688 -2.128040
C 2.982631 -0.546510 1.094805
O 3.170476 -0.144944 2.230445
O 3.903744 -0.466727 0.131666
C 5.159578 0.132279 0.508315
H 5.611439 -0.452987 1.313805
H 4.980653 1.149918 0.866124
S -2.318079 -2.900004 -0.741665
C -3.396163 -1.437991 -0.471880
C -3.673243 -1.056778 0.984509
H -4.308140 -1.670445 -1.026746
H -3.346500 -1.796825 1.717802
H -4.739659 -0.867733 1.146413
N -2.476248 0.688393 -0.043689
C -2.747223 -0.199515 -1.072447
O -2.474450 -0.004408 -2.241458
C -2.933509 0.238236 1.192446
O -2.756835 0.832437 2.239800
C -1.690213 1.872143 -0.207532
C -2.266353 3.120200 0.007922
C -0.349099 1.742125 -0.560198
C -1.477836 4.261034 -0.129566
H -3.314306 3.194975 0.281740
C 0.428633 2.887970 -0.703026
H 0.075299 0.755572 -0.720222
C -0.134046 4.146086 -0.485485
H -1.916437 5.239755 0.039409
H 1.474879 2.795425 -0.978607
H 0.475871 5.038222 -0.592638
C 6.017920 0.141196 -0.670891
C 6.750662 0.161378 -1.629255
H 7.398964 0.178176 -2.482750
40
B_conf6 Eopt -1523.947143
C 2.101143 -2.263757 -0.335921
C 3.447082 -2.122935 -0.517226
C 3.999952 -0.873481 -0.180401
C 3.169960 0.137794 0.311762
C 1.802481 -0.065785 0.478333
C 1.230206 -1.304677 0.146588
H 4.070282 -2.924396 -0.902904
H 3.587287 1.103544 0.573968
H 1.198757 0.746858 0.867646
C 5.460211 -0.681570 -0.363710
O 6.214577 -1.534537 -0.802598
O 5.876380 0.534115 0.006542
C 7.287418 0.789718 -0.136208

H 7.573848 0.665396 -1.184102
H 7.847560 0.073864 0.471700
S -0.473030 -1.752583 0.319649
C -1.263362 -0.141601 0.659331
C -1.215842 0.888580 -0.473117
H -0.870729 0.257969 1.599008
H -0.708686 0.529881 -1.373192
H -0.737044 1.821594 -0.164687
N -3.468310 0.389209 0.011668
C -2.746123 -0.408643 0.880097
O -3.234356 -1.176215 1.687079
C -2.657041 1.167955 -0.809162
O -3.081201 1.934328 -1.653226
C -4.898983 0.405337 -0.039116
C -5.581044 1.555375 0.348447
C -5.577453 -0.728204 -0.478646
C -6.972759 1.568994 0.288787
H -5.028398 2.425509 0.689692
C -6.969553 -0.708253 -0.524209
H -5.021422 -1.611383 -0.778579
C -7.666319 0.438790 -0.143985
H -7.513941 2.462433 0.584930
H -7.508120 -1.588565 -0.861576
H -8.751454 0.452018 -0.185268
C 7.536899 2.156038 0.309852
C 7.768662 3.282940 0.673470
H 7.972291 4.284930 0.995148
40
B_conf7 Eopt -
1523.946986
C 2.198532 -2.397799 0.842562
C 3.494735 -1.982429 0.949663
C 3.801819 -0.728927 0.388816
C 2.792376 0.013931 -0.230459
C 1.484420 -0.458867 -0.301123
C 1.158632 -1.711183 0.243559
H 4.259627 -2.580477 1.436184
H 3.017265 0.982981 -0.661647
H 0.731396 0.161282 -0.774687
C 5.200972 -0.243187 0.482227
O 6.102182 -0.848664 1.039779
O 5.383190 0.939230 -0.115382
C 6.721452 1.471200 -0.067956
H 7.408135 0.766814 -0.545396
H 7.020583 1.605746 0.975163
S -0.429173 -2.496963 0.241630
C -1.472426 -1.428168 -0.792695
C -2.673295 -2.190506 -1.363058
H -0.871398 -1.014974 -1.608428
H -2.890119 -3.119668 -0.823801
H -2.562793 -2.430551 -2.421686
N -3.435954 -0.204084 -0.354699
C -2.087585 -0.256850 -0.039063
O -1.519547 0.530235 0.692339
C -3.845435 -1.268142 -1.150632
O -4.980716 -1.412794 -1.563869
C -4.315518 0.826289 0.108334
C -4.820572 1.749211 -0.803325
C -4.647350 0.883487 1.459190
C -5.680396 2.747233 -0.350115
H -4.543882 1.683197 -1.851185
C -5.499812 1.891427 1.904352
H -4.241586 0.149646 2.149006
C -6.017575 2.820160 1.001468
H -6.082680 3.469636 -1.053761
H -5.761791 1.947105 2.956526
H -6.684799 3.602121 1.351841
C 6.715922 2.747796 -0.773918
C 6.738329 3.806450 -1.352353
H 6.756286 4.746324 -1.867071
40
B_conf9 Eopt -
1523.952220
C -0.246441 -2.108149 -1.534072
C 1.013637 -1.635674 -1.796578
C 1.799342 -1.309570 -0.679687
C 1.296393 -1.490252 0.613840
C 0.015280 -1.994938 0.815102
C -0.799005 -2.298046 -0.287699
H 1.391607 -1.500606 -2.805328
H 1.907518 -1.244393 1.474622
H -0.349889 -2.148690 1.826317
C 3.161272 -0.756982 -0.924602
O 3.649201 -0.610961 -2.032159
O 3.796347 -0.428233 0.202234
C 5.120097 0.120873 0.045852
H 5.065637 1.024199 -0.567833
H 5.757172 -0.612044 -0.456787
S -2.470837 -2.917583 -0.136893
C -3.364757 -1.404826 0.403355
C -3.125389 -0.950205 1.845606
H -4.414774 -1.632484 0.205149
H -2.591703 -1.668733 2.469812
H -4.072226 -0.712137 2.341984
N -2.329139 0.709854 0.385085
C -2.948489 -0.211371 -0.443414
O -3.097176 -0.068797 -1.642038
C -2.338325 0.327088 1.723265
O -1.804687 0.962147 2.614103
C -1.678907 1.891459 -0.093405
C -2.252915 3.137135 0.141194
C -0.472586 1.764221 -0.776185
C -1.600279 4.278971 -0.319545
H -3.194096 3.208812 0.677792
C 0.169676 2.910016 -1.238186
H -0.047141 0.779263 -0.940804
C -0.392852 4.165992 -1.009058
H -2.037944 5.256405 -0.140985
H 1.110228 2.819667 -1.773163
H 0.111024 5.058596 -1.367938

C 5.631605 0.427901 1.376825
C 6.076442 0.691045 2.467042
H 6.469668 0.923109 3.436834
40
C_conf1 Eopt -
1523.976642
C -1.608673 0.756476 -1.121267
C -2.921368 1.181932 -0.961017
C -3.874794 0.345991 -0.374904
C -3.498207 -0.929165 0.059888
C -2.184377 -1.353481 -0.080200
C -1.233520 -0.514564 -0.674946
H -3.205398 2.171799 -1.303148
H -4.222802 -1.590809 0.521357
H -1.900009 -2.337957 0.280258
C -5.263834 0.846840 -0.230058
O -5.638411 1.955176 -0.579525
O -6.084071 -0.049323 0.330680
C -7.451166 0.367431 0.508721
H -7.883619 0.618610 -0.463730
H -7.479018 1.254044 1.148215
S 0.394996 -1.200288 -0.869033
C 1.463390 0.298338 -0.953795
C 1.459918 1.087111 0.296976
H 1.215378 0.878639 -1.848665
H 0.613261 1.602222 0.730892
N 3.598091 0.298254 0.033292
C 2.911079 -0.179009 -1.063507
O 3.384763 -0.854882 -1.957380
C 2.774885 1.062111 0.887862
O 3.163416 1.583354 1.926584
C 4.981195 0.039544 0.282480
C 5.879338 1.103413 0.317516
C 5.403239 -1.271725 0.489541
C 7.224943 0.846048 0.569455
H 5.527904 2.116858 0.149735
C 6.752915 -1.519584 0.728224
H 4.683454 -2.084382 0.463309
C 7.662460 -0.462892 0.771551
H 7.931276 1.670014 0.602237
H 7.091047 -2.539183 0.886121
H 8.713163 -0.659640 0.963088
C -8.174604 -0.739083 1.125881
C -8.795169 -1.639662 1.635489
H -9.344038 -2.440941 2.088825
H -0.898643 1.426548 -1.593214
40
C_conf10 Eopt -
1523.984001
C -0.328358 -2.125661 -1.562074
C 0.955286 -1.630775 -1.762001
C 1.757280 -1.302038 -0.666985
C 1.280735 -1.495716 0.633030
C 0.008675 -2.018141 0.835004
C -0.805591 -2.314850 -0.262290
H 1.325071 -1.479498 -2.770745
H 1.898569 -1.250630 1.489539
H -0.345996 -2.189335 1.846868
C 3.108105 -0.735192 -0.928782
O 3.580643 -0.574135 -2.041758
O 3.760270 -0.410095 0.190843
C 5.077028 0.148561 0.017426
H 5.007535 1.057849 -0.585851
H 5.710561 -0.574833 -0.503301
S -2.467501 -2.937096 -0.034362
C -3.318549 -1.395552 0.539442
C -2.891721 -0.955164 1.882391
H -4.386299 -1.629341 0.468699
H -3.013058 -1.505581 2.805431
N -2.310294 0.721709 0.433046
C -2.965724 -0.207264 -0.351669
O -3.191748 -0.086626 -1.541102
C -2.233699 0.324499 1.783569
O -1.696984 0.989941 2.662427
C -1.698349 1.907995 -0.075896
C -2.210016 3.154523 0.273376
C -0.585033 1.787395 -0.904187
C -1.590938 4.301854 -0.218822
H -3.078672 3.222440 0.921178
C 0.021589 2.939099 -1.398302
H -0.202710 0.802480 -1.154817
C -0.479342 4.195118 -1.054801
H -1.980784 5.279036 0.049478
H 0.888310 2.853435 -2.046791
H -0.002135 5.092047 -1.438468
C 5.609822 0.445140 1.342656
C 6.071229 0.699070 2.428188
H 6.480392 0.924612 3.392926
H -0.960733 -2.351207 -2.414595
40
C_conf11 Eopt -
1523.976582
C -1.979414 -2.222976 0.453747
C -3.336126 -2.015901 0.651759
C -3.942317 -0.834839 0.211864
C -3.171581 0.132383 -0.438323
C -1.814319 -0.075670 -0.654499
C -1.211260 -1.254013 -0.205643
H -3.925390 -2.771159 1.161522
H -3.625128 1.053224 -0.788042
H -1.249163 0.690377 -1.172810
C -5.393861 -0.650555 0.454309
O -6.112832 -1.475600 0.995679
O -5.850841 0.527852 0.014600
C -7.256145 0.774579 0.211135
H -7.487400 0.725125 1.278736
H -7.835161 0.008511 -0.311958
S 0.497973 -1.664235 -0.462548
C 1.272963 -0.023975 -0.777049
C 1.258237 0.860214 0.408504

H	0.837817	0.424188	-1.676729
H	0.383211	1.265887	0.898605
N	3.463455	0.417254	-0.043603
C	2.763651	-0.261375	-1.019336
O	3.255571	-0.923867	-1.913232
C	2.610280	1.104085	0.845985
O	3.005077	1.770167	1.795772
C	4.888570	0.417358	0.061914
C	5.584100	1.614271	-0.090938
C	5.553405	-0.779039	0.320417
C	6.972489	1.610155	0.022489
H	5.044003	2.533631	-0.295868
C	6.942685	-0.774371	0.420253
H	4.988721	-1.698412	0.442244
C	7.651569	0.418115	0.274792
H	7.522597	2.539182	-0.091736
H	7.469331	-1.702995	0.618245
H	8.734306	0.418527	0.358667
C	-7.551505	2.100548	-0.321135
C	-7.819681	3.194826	-0.753074
H	-8.056792	4.166972	-1.137391
H	-1.518956	-3.137402	0.816923

40

C_conf12 Eopt -1523.976746

C	1.285384	0.476079	-0.221720
C	2.516406	1.057760	-0.496248
C	3.703073	0.411928	-0.139095
C	3.647666	-0.834844	0.492024
C	2.420457	-1.432523	0.747674
C	1.234878	-0.778998	0.392415
H	2.552905	2.029319	-0.978275
H	4.558326	-1.352957	0.771331
H	2.390310	-2.412510	1.214861
C	4.991557	1.082111	-0.444276
O	5.091128	2.160216	-1.008472
O	6.054396	0.381360	-0.032698
C	7.341370	0.975195	-0.289224
H	7.397731	1.946634	0.209562
H	7.464779	1.122445	-1.365744
S	-0.284608	-1.597740	0.826558
C	-1.430266	-1.100849	-0.518828
C	-2.360863	-2.193960	-0.876873
H	-0.821675	-0.790472	-1.378124
H	-2.084732	-3.182471	-1.218426
N	-3.665020	-0.407555	-0.272155
C	-2.366705	0.040537	-0.121321
O	-2.047450	1.152266	0.254548
C	-3.721955	-1.741559	-0.723554
O	-4.764818	-2.352109	-0.928952
C	-4.819370	0.384593	0.014106
C	-5.685034	0.731319	-1.020251
C	-5.057015	0.792408	1.324616
C	-6.812355	1.497454	-0.732360
H	-5.477064	0.405452	-2.034893
C	-6.181109	1.567339	1.600333
H	-4.370127	0.504744	2.114831
C	-7.059280	1.917497	0.574653
H	-7.494222	1.769608	-1.532222
H	-6.372112	1.891946	2.618678
H	-7.937230	2.517736	0.794478
C	8.359675	0.068137	0.229040
C	9.222131	-0.663527	0.649398
H	9.987089	-1.314673	1.023176
H	0.376962	1.011481	-0.477537

40

C_conf13 Eopt -1523.976746

C	-1.285541	0.476765	0.220210
C	-2.516531	1.058411	0.494911
C	-3.703231	0.412244	0.138461
C	-3.647862	-0.834779	-0.492150
C	-2.420648	-1.432413	-0.747951
C	-1.235052	-0.778585	-0.393363
H	-2.552996	2.030178	0.976526
H	-4.558543	-1.353141	-0.770929
H	-2.390530	-2.412605	-1.214707
C	-4.991700	1.082349	0.443896
O	-5.091209	2.160691	1.007646
O	-6.054595	0.381274	0.033018
C	-7.341562	0.974978	0.289856
H	-7.398346	1.946195	-0.209319
H	-7.464518	1.122681	1.366367
S	0.284507	-1.597251	-0.827483
C	1.429948	-1.100165	0.518009
C	2.360101	-2.193288	0.877188
H	0.821153	-0.789109	1.376930
H	2.083544	-3.181543	1.219134
N	3.665014	-0.407445	0.272400
C	2.366871	0.040772	0.120318
O	2.048089	1.152305	-0.256516
C	3.721383	-1.741210	0.724571
O	4.763991	-2.351849	0.931013
C	4.819703	0.384271	-0.013670
C	5.685031	0.731167	1.020913
C	5.058027	0.791504	-1.324245
C	6.812694	1.496867	0.733190
H	5.476533	0.405775	2.035597
C	6.182449	1.566013	-1.599794
H	4.371405	0.503708	-2.114643
C	7.060292	1.916322	-0.573882
H	7.494302	1.769142	1.533232
H	6.373976	1.890158	-2.618187
H	7.938502	2.516229	-0.793574
C	-8.359945	0.067502	-0.227532
C	-9.222343	-0.664592	-0.647257
H	-9.987274	-1.316145	-1.020373
H	-0.377136	1.012423	0.475505

40

C_conf14 Eopt -1523.976659
C -1.606633 0.716457 -1.159854
C -2.916725 1.151115 -1.004675
C -3.872139 0.331538 -0.399064
C -3.500475 -0.937159 0.058576
C -2.188973 -1.370271 -0.076957
C -1.235684 -0.546811 -0.688893
H -3.197280 2.135215 -1.366020
H -4.226847 -1.586601 0.534333
H -1.907930 -2.349212 0.300886
C -5.257752 0.842750 -0.257636
O -5.630959 1.941409 -0.637862
O -6.076647 -0.032373 0.337173
C -7.440413 0.395429 0.514403
H -7.881948 0.614003 -0.461836
H -7.458783 1.303975 1.122625
S 0.391252 -1.239917 -0.872193
C 1.461786 0.255852 -0.961724
C 1.445484 1.063011 0.277157
H 1.224622 0.825666 -1.866484
H 0.593963 1.579945 0.699114
N 3.590824 0.286320 0.037170
C 2.911637 -0.219686 -1.051601
O 3.393023 -0.914179 -1.926881
C 2.758444 1.060702 0.872758
O 3.138763 1.606007 1.902126
C 4.977333 0.052079 0.292347
C 5.861041 1.128570 0.293307
C 5.417590 -1.246323 0.538072
C 7.210492 0.897471 0.549916
H 5.495484 2.131636 0.095821
C 6.771056 -1.468372 0.781167
H 4.709252 -2.069313 0.538181
C 7.666213 -0.398676 0.790495
H 7.905404 1.731699 0.556187
H 7.123292 -2.478080 0.968987
H 8.719844 -0.575308 0.985552
C -8.160959 -0.686056 1.177498
C -8.779327 -1.565282 1.725426
H -9.325948 -2.347637 2.213115
H -0.894364 1.373508 -1.646299
40
C_conf2 Eopt -1523.976643
C -1.608620 0.755461 -1.121806
C -2.921271 1.181162 -0.961825
C -3.874781 0.345727 -0.375108
C -3.498325 -0.929156 0.060567
C -2.184531 -1.353707 -0.079226
C -1.233602 -0.515315 -0.674583
H -3.205206 2.170823 -1.304624
H -4.222973 -1.590401 0.522521
H -1.900267 -2.337944 0.281958
C -5.263774 0.846826 -0.230614
O -5.638249 1.954919 -0.580955
O -6.084103 -0.048797 0.330841
C -7.451179 0.368214 0.508413
H -7.883437 0.618928 -0.464245
H -7.479022 1.255177 1.147423
S 0.394848 -1.201336 -0.868193
C 1.463325 0.297146 -0.953610
C 1.459798 1.086628 0.296722
H 1.215352 0.877066 -1.848745
H 0.612997 1.601732 0.730375
N 3.598053 0.297909 0.033388
C 2.911039 -0.180146 -1.063068
O 3.384786 -0.856482 -1.956562
C 2.774789 1.062154 0.887565
O 3.163341 1.584129 1.925912
C 4.981295 0.039827 0.282471
C 5.879121 1.103999 0.316415
C 5.403802 -1.271142 0.490487
C 7.224871 0.847254 0.568199
H 5.527333 2.117202 0.147917
C 6.753620 -1.518394 0.729012
H 4.684264 -2.084043 0.465117
C 7.662849 -0.461388 0.771234
H 7.930960 1.671463 0.600116
H 7.092107 -2.537761 0.887643
H 8.713667 -0.657652 0.962642
C -8.174875 -0.737865 1.126050
C -8.795663 -1.638084 1.636028
H -9.344717 -2.439044 2.089707
H -0.898519 1.425112 -1.594270
40
C_conf3 Eopt -1523.984054
C -0.613143 -2.134534 1.078716
C 0.638261 -1.698576 1.496741
C 1.636579 -1.427860 0.557444
C 1.381793 -1.603024 -0.805193
C 0.124425 -2.021346 -1.226782
C -0.877721 -2.277187 -0.287050
H 0.838651 -1.568235 2.555078
H 2.151356 -1.398566 -1.540987
H -0.080620 -2.131200 -2.286714
C 2.951936 -0.935805 1.049392
O 3.262905 -0.865857 2.226692
O 3.767326 -0.562076 0.059325
C 5.061325 -0.068638 0.456946
H 5.597462 -0.854797 0.995630
H 4.932683 0.791346 1.119832
S -2.496467 -2.773898 -0.866120
C -3.433950 -1.197565 -0.600022
C -3.648129 -0.882738 0.826199
H -4.359499 -1.326793 -1.171168
H -4.207728 -1.476310 1.536292
N -2.373602 0.818061 -0.030193
C -2.647136 0.005797 -1.113843

O -2.298146 0.221714 -2.259167
C -2.947120 0.331763 1.162552
O -2.837513 0.887026 2.250262
C -1.536026 1.973888 -0.086472
C -2.082767 3.234606 0.135838
C -0.177353 1.808409 -0.344479
C -1.248098 4.349874 0.099560
H -3.144896 3.338240 0.335861
C 0.647404 2.929338 -0.387341
H 0.224053 0.812812 -0.506751
C 0.113526 4.198674 -0.163319
H -1.664343 5.337324 0.274243
H 1.707438 2.808032 -0.589587
H 0.759601 5.071120 -0.192140
C 5.779030 0.314548 -0.753836
C 6.392347 0.639500 -1.740762
H 6.936295 0.926820 -2.618571
H -1.379442 -2.355066 1.815621
40
C_conf7 Eopt -1523.976728
C 2.257276 -2.479649 0.636823
C 3.579882 -2.063371 0.571762
C 3.887561 -0.737233 0.254669
C 2.853475 0.173212 0.016598
C 1.527155 -0.232379 0.102052
C 1.225125 -1.563812 0.402036
H 4.376528 -2.777451 0.752876
H 3.074819 1.207400 -0.222622
H 0.739771 0.497163 -0.055414
C 5.315782 -0.339888 0.183250
O 6.252961 -1.097314 0.379246
O 5.483358 0.952265 -0.120636
C 6.846400 1.410460 -0.203740
H 7.371447 0.846416 -0.979621
H 7.344823 1.241937 0.754842
S -0.437251 -2.175422 0.575940
C -1.377077 -1.199435 -0.661796
C -2.435631 -2.005279 -1.309867
H -0.660378 -0.812067 -1.397501
H -2.291945 -2.927084 -1.857286
N -3.484616 -0.193049 -0.375162
C -2.150941 -0.030048 -0.051951
O -1.693417 0.893244 0.593974
C -3.714113 -1.363509 -1.126182
O -4.818569 -1.718980 -1.521631
C -4.517079 0.713087 0.017919
C -5.222855 1.410141 -0.959803
C -4.798687 0.880232 1.371744
C -6.232836 2.287170 -0.570987
H -4.982557 1.266590 -2.008877
C -5.802651 1.768332 1.750743
H -4.238690 0.321365 2.115383
C -6.521025 2.468752 0.781705
H -6.790372 2.831931 -1.326778
H -6.026313 1.907194 2.804115
H -7.306561 3.156299 1.080979
C 6.816635 2.832461 -0.528696
C 6.818841 4.008955 -0.796543
H 6.819181 5.053916 -1.035056
H 2.032477 -3.518409 0.860001
20
D_conf1 Eopt -933.644129
C 2.457836 1.089491 -0.000008
C 1.130094 1.477954 -0.000002
C 0.115977 0.510941 0.000009
C 0.437955 -0.853495 0.000015
C 1.764569 -1.248507 0.000008
C 2.802641 -0.284572 -0.000003
H 0.871773 2.531546 -0.000006
H -0.345689 -1.602627 0.000023
H 2.015937 -2.304226 0.000011
C -1.299010 0.979568 0.000014
O -1.628758 2.153095 -0.000052
O -2.177267 -0.025015 0.000069
C -3.568268 0.353401 0.000031
H -3.778586 0.952930 -0.889901
H -3.778636 0.952900 0.889971
S 4.452067 -0.772772 -0.000009
H 3.244244 1.837392 -0.000015
C -4.361111 -0.870724 -0.000011
C -5.042097 -1.866662 -0.000049
H -5.645442 -2.752568 -0.000100
20
D_conf2 Eopt -933.644133
C -2.229345 1.208420 0.084168
C -0.864159 1.359776 -0.080763
C -0.045512 0.231577 -0.225895
C -0.601631 -1.055113 -0.203894
C -1.966678 -1.213099 -0.037253
C -2.809103 -0.083988 0.109872
H -0.425089 2.351609 -0.098882
H 0.031132 -1.928218 -0.314501
H -2.399785 -2.207881 -0.018672
C 1.419172 0.445630 -0.398654
O 1.954095 1.539876 -0.378361
O 2.090229 -0.696620 -0.581747
C 3.515206 -0.597303 -0.765025
H 3.737509 0.183451 -1.496239
H 3.805312 -1.564812 -1.176036
S -4.505900 -0.279130 0.315368
H -2.864644 2.081129 0.195506
C 4.208772 -0.346638 0.498013
C 4.796452 -0.152518 1.534258
H 5.316333 0.024286 2.454846
42
TS_1_conf1 Eopt -1633.420237

C 1.768045 1.629571 0.118101
C 3.034695 1.515808 -0.392257
C 3.775178 0.385040 -0.022375
C 3.213997 -0.568498 0.831395
C 1.915045 -0.412015 1.308951
C 1.159655 0.712909 0.954175
H 3.457440 2.273141 -1.047707
H 3.781838 -1.445711 1.119737
H 1.482547 -1.168470 1.956768
C 5.156301 0.247706 -0.562933
O 5.655842 1.021958 -1.361002
O 5.799299 -0.816174 -0.077260
C 7.145667 -1.013055 -0.554397
H 7.748361 -0.135496 -0.304907
H 7.128728 -1.135319 -1.640801
S -0.492236 0.921778 1.595432
C -1.422877 0.674723 0.035999
C -1.229766 -0.675273 -0.656008
H -1.197548 1.505111 -0.639207
H -0.671629 -1.394478 -0.048038
H -0.729494 -0.588271 -1.622835
N -3.525618 -0.349379 -0.234342
C -2.908906 0.748112 0.341167
O -3.485774 1.621666 0.960966
C -2.623504 -1.213336 -0.849335
O -2.946772 -2.236977 -1.422331
C -4.938186 -0.576273 -0.188602
C -5.677029 -0.506002 -1.366431
C -5.543055 -0.864873 1.031777
C -7.050333 -0.734846 -1.318672
H -5.181190 -0.276682 -2.304825
C -6.918658 -1.081394 1.070773
H -4.943426 -0.917562 1.935442
C -7.670932 -1.019440 -0.102272
H -7.634752 -0.686421 -2.232377
H -7.400548 -1.303428 2.018070
H -8.742314 -1.193725 -0.068497
N 1.294837 3.971676 -1.006765
N 0.809590 3.229187 -0.311171
C 7.675260 -2.207445 0.093862
C 8.138617 -3.191417 0.616167
H 8.550038 -4.065313 1.080732
42
TS_1_conf10 Eopt -1633.425846
C 0.058956 1.592331 -1.297089
C -1.069353 0.873134 -1.599356
C -2.052048 0.774152 -0.608021
C -1.872528 1.415791 0.620826
C -0.712298 2.141438 0.873889
C 0.300113 2.226165 -0.093204
H -1.195666 0.388134 -2.563883
H -2.636494 1.354174 1.387418
H -0.590166 2.642676 1.828940
C -3.271855 -0.026178 -0.912563
O -3.455737 -0.611777 -1.965535
O -4.146980 -0.039283 0.093962
C -5.350854 -0.801564 -0.126607
H -5.085878 -1.844752 -0.319135
H -5.876190 -0.401634 -0.998135
S 1.821309 3.107994 0.238904
C 2.788206 1.802324 1.097796
C 2.206305 1.283448 2.413690
H 3.777834 2.248579 1.221843
H 1.387101 1.874700 2.824350
H 2.986531 1.215472 3.179776
N 2.248338 -0.480264 0.859013
C 2.920019 0.558321 0.231147
O 3.498296 0.461563 -0.833842
C 1.750239 -0.118378 2.109049
O 1.085158 -0.853157 2.815447
C 2.100270 -1.786085 0.293068
C 2.681673 -2.876657 0.934836
C 1.377073 -1.942706 -0.886233
C 2.527451 -4.147692 0.385984
H 3.245498 -2.730042 1.851132
C 1.236835 -3.215842 -1.432969
H 0.931626 -1.078871 -1.368623
C 1.807926 -4.317898 -0.796868
H 2.974754 -5.003818 0.881713
H 0.676573 -3.344606 -2.354082
H 1.692807 -5.309982 -1.223378
N 1.271662 1.045574 -3.571799
N 1.332278 1.767751 -2.708324
C -6.173636 -0.687923 1.072203
C -6.876242 -0.610510 2.049957
H -7.498644 -0.542148 2.919915
42
TS_1_conf11 Eopt -1633.421100
C 2.063131 1.960593 0.067611
C 3.409767 1.776991 0.216051
C 3.907238 0.481498 0.014780
C 3.023477 -0.544993 -0.329996
C 1.660186 -0.304592 -0.473773
C 1.141600 0.983162 -0.269026
H 4.069379 2.599170 0.482510
H 3.393201 -1.551107 -0.492372
H 1.017859 -1.133089 -0.748770
C 5.364858 0.253406 0.177195
O 6.167571 1.126964 0.462550
O 5.717009 -1.020659 -0.021700
C 7.120150 -1.317728 0.117371
H 7.448009 -1.052284 1.126170
H 7.687870 -0.727347 -0.607087
S -0.560352 1.447453 -0.426060
C -1.377762 -0.167439 -0.661292
C -1.364562 -1.118472 0.539808
H -0.987444 -0.638198 -1.568249
H -0.849138 -0.714147 1.415224

H -0.913515 -2.084667 0.298464
N -3.600579 -0.600148 0.001151
C -2.852900 0.116223 -0.915412
O -3.316365 0.833396 -1.780943
C -2.814555 -1.333606 0.885750
O -3.263217 -2.022537 1.782156
C -5.031451 -0.576569 0.041617
C -5.740409 -1.736355 -0.258840
C -5.683160 0.604915 0.384850
C -7.132348 -1.710283 -0.208906
H -5.208213 -2.644261 -0.526092
C -7.075521 0.623545 0.421176
H -5.106374 1.494672 0.618987
C -7.799211 -0.532276 0.127601
H -7.694417 -2.610525 -0.437980
H -7.593448 1.540914 0.683880
H -8.884549 -0.514916 0.161426
N 2.089829 4.549919 0.625926
N 1.369204 3.732159 0.341257
C 7.296692 -2.745369 -0.125035
C 7.470461 -3.923365 -0.319916
H 7.623172 -4.970011 -0.493679
42
TS_1_conf12 Eopt -1633.421322
C 1.453028 -2.125803 -0.047605
C 2.664790 -1.700641 -0.525516
C 3.156704 -0.484403 -0.032640
C 2.416250 0.239820 0.905987
C 1.181706 -0.229715 1.345811
C 0.673267 -1.443094 0.865559
H 3.230042 -2.282388 -1.249330
H 2.791485 1.181711 1.289279
H 0.603468 0.351904 2.057387
C 4.473743 -0.006500 -0.538467
O 5.131232 -0.588127 -1.384326
O 4.865186 1.133618 0.034338
C 6.131374 1.661646 -0.408714
H 6.083465 1.858303 -1.483253
H 6.916510 0.925270 -0.217253
S -0.889430 -2.052322 1.468649
C -1.937860 -1.948008 -0.039339
C -3.373469 -2.333570 0.322386
H -1.505706 -2.557410 -0.833020
H -3.486610 -2.887935 1.257294
H -3.825384 -2.930363 -0.476248
N -3.292552 -0.022562 -0.107020
C -2.043804 -0.500780 -0.478931
O -1.193929 0.157323 -1.048412
C -4.114708 -1.022791 0.401664
O -5.244758 -0.836474 0.813043
C -3.684188 1.345616 -0.246209
C -4.725710 1.677480 -1.108379
C -3.009017 2.320156 0.484485
C -5.101305 3.013146 -1.233706
H -5.232936 0.900205 -1.671786
C -3.384261 3.654007 0.343429
H -2.200888 2.034441 1.151644
C -4.430627 4.000349 -0.511775
H -5.915172 3.281188 -1.900475
H -2.861232 4.421305 0.906007
H -4.723333 5.040946 -0.615941
N 1.467078 -4.401150 -1.391319
N 0.831011 -3.832011 -0.655252
C 6.381694 2.892455 0.332763
C 6.612398 3.913741 0.932379
H 6.816532 4.820663 1.466173
42
TS_1_conf3 Eopt -1633.427024
C -1.465911 -2.056572 -0.066355
C -0.214125 -2.446477 0.340943
C 0.875263 -1.962358 -0.390951
C 0.664587 -1.169924 -1.522776
C -0.625221 -0.818362 -1.903018
C -1.729626 -1.230470 -1.141459
H -0.067894 -3.078090 1.213708
H 1.506609 -0.807777 -2.101493
H -0.778973 -0.191147 -2.775415
C 2.244775 -2.245882 0.121400
O 2.505763 -3.099759 0.950779
O 3.155066 -1.422108 -0.397686
C 4.496530 -1.546952 0.113184
H 4.899423 -2.525594 -0.161861
H 4.475169 -1.464632 1.203348
S -3.364325 -0.624760 -1.547209
C -3.397134 0.901389 -0.516121
C -3.419336 0.684529 0.996408
H -4.267173 1.452364 -0.880445
H -3.625274 -0.340933 1.306775
H -4.158428 1.334713 1.475988
N -1.394222 1.731902 0.413242
C -2.140736 1.725490 -0.758068
O -1.805139 2.263872 -1.794683
C -2.054952 1.108593 1.470453
O -1.586656 0.978364 2.586100
C -0.054199 2.227816 0.491320
C 0.211230 3.562936 0.200876
C 0.968428 1.347160 0.837888
C 1.528703 4.016001 0.243746
H -0.599953 4.236059 -0.057712
C 2.280096 1.810797 0.886670
H 0.737673 0.311506 1.063649
C 2.561874 3.143198 0.584482
H 1.744863 5.054579 0.012457
H 3.081279 1.126960 1.152174
H 3.586737 3.501201 0.615516
N -2.710264 -3.441124 1.802069
N -2.933452 -2.801982 0.900595
C 5.288520 -0.470082 -0.470708

C 5.957555 0.418309 -0.938389
H 6.550510 1.207610 -1.355914
42
TS_1_conf7 Eopt -1633.420794
C 2.105426 2.078364 -0.401440
C 3.377410 1.608935 -0.577182
C 3.646392 0.305438 -0.137461
C 2.621716 -0.443216 0.449189
C 1.341218 0.080229 0.601942
C 1.052989 1.384428 0.172181
H 4.149801 2.219313 -1.038654
H 2.812549 -1.454339 0.790806
H 0.579103 -0.547741 1.048319
C 5.018867 -0.228607 -0.319849
O 5.938178 0.401229 -0.817248
O 5.152260 -1.484465 0.118020
C 6.460851 -2.068918 -0.032247
H 7.190098 -1.470550 0.520748
H 6.735517 -2.072933 -1.090597
S -0.506454 2.218105 0.307620
C -1.593090 1.038980 1.163950
C -2.797148 1.759422 1.782463
H -1.029773 0.511521 1.938733
H -2.931308 2.782059 1.412752
H -2.751260 1.799145 2.872169
N -3.574372 -0.013708 0.440919
C -2.204443 -0.000617 0.234002
O -1.619487 -0.725172 -0.547078
C -3.994446 0.958665 1.341099
O -5.152678 1.122167 1.676322
C -4.463049 -0.922285 -0.218310
C -5.086863 -1.921454 0.523714
C -4.684077 -0.788105 -1.586232
C -5.953955 -2.801223 -0.120332
H -4.894490 -2.005808 1.589030
C -5.545270 -1.678956 -2.222988
H -4.187108 0.002022 -2.140888
C -6.181052 -2.682204 -1.491495
H -6.447889 -3.582057 0.449856
H -5.721945 -1.584972 -3.290126
H -6.854625 -3.372487 -1.990691
N 2.569792 4.456596 -1.469835
N 1.735054 3.869159 -0.992906
C 6.402201 -3.428899 0.492095
C 6.381821 -4.557750 0.917582
H 6.362105 -5.560622 1.295169
42
TS_1_conf8 Eopt -1633.426764
C -0.428425 1.506119 1.365400
C 0.787819 0.950401 1.670980
C 1.777028 0.989029 0.681896
C 1.511634 1.593942 -0.549906
C 0.263226 2.153365 -0.806060
C -0.750604 2.104775 0.161919
H 0.976968 0.485316 2.635079
H 2.276960 1.632613 -1.316644
H 0.073539 2.627760 -1.763888
C 3.088005 0.347450 0.980947
O 3.358122 -0.186435 2.042770
O 3.939228 0.410209 -0.044161
C 5.228948 -0.199233 0.165850
H 5.092838 -1.257029 0.407068
H 5.728684 0.294814 1.003458
S -2.376890 2.778575 -0.165397
C -3.143307 1.371085 -1.069162
C -2.531482 1.040209 -2.430576
H -4.201606 1.634227 -1.127170
H -1.880434 1.812938 -2.842393
H -3.315012 0.832340 -3.167162
N -2.115385 -0.744706 -0.958827
C -2.980136 0.090489 -0.265345
O -3.473868 -0.170497 0.813914
C -1.765368 -0.236759 -2.207677
O -0.993864 -0.783333 -2.974129
C -1.568137 -1.943293 -0.402277
C -2.404761 -3.014976 -0.105258
C -0.199441 -2.000196 -0.151403
C -1.856258 -4.162921 0.463596
H -3.468011 -2.949258 -0.314297
C 0.340536 -3.154380 0.409314
H 0.430760 -1.150320 -0.393669
C -0.487065 -4.233771 0.719968
H -2.500983 -5.002959 0.703385
H 1.406860 -3.205733 0.608000
H -0.064331 -5.131032 1.162227
N -1.540168 0.829651 3.656649
N -1.713671 1.512833 2.777039
C 5.997356 -0.042504 -1.063855
C 6.651938 0.074087 -2.070567
H 7.232603 0.177641 -2.965520
42
TS_1_conf9 Eopt -1633.426005
C 0.317173 -2.153349 0.781401
C -0.946771 -1.805728 1.187218
C -1.807464 -1.274838 0.220211
C -1.372870 -1.139022 -1.101835
C -0.088439 -1.529588 -1.466501
C 0.802102 -2.034780 -0.506847
H -1.265276 -1.919787 2.220391
H -2.037820 -0.737030 -1.857533
H 0.219433 -1.444570 -2.503944
C -3.171683 -0.857637 0.650246
O -3.589840 -0.967500 1.789744
O -3.892561 -0.343029 -0.347307
C -5.226470 0.085656 -0.007391
H -5.171766 0.849949 0.772755
H -5.793619 -0.768999 0.371440

S 2.471197 -2.518534 -0.934751
C 3.272912 -0.877755 -1.150117
C 2.888411 -0.086201 -2.402702
H 4.341665 -1.099989 -1.106412
H 2.330782 -0.651173 -3.150968
H 3.778498 0.327284 -2.888862
N 2.177687 1.103807 -0.505611
C 2.910476 0.044614 0.004380
O 3.178248 -0.104040 1.181191
C 2.069295 1.069356 -1.892491
O 1.428712 1.874384 -2.543163
C 1.539265 2.089602 0.312075
C 2.063034 3.377073 0.376680
C 0.393871 1.734910 1.018834
C 1.421553 4.328006 1.168099
H 2.957125 3.628077 -0.186046
C -0.237735 2.689718 1.811327
H 0.006798 0.723692 0.944656
C 0.274983 3.985137 1.885009
H 1.819884 5.336395 1.225074
H -1.131164 2.420962 2.366895
H -0.220400 4.729394 2.501552
N 1.080234 -2.885640 3.199057
N 1.474688 -2.831813 2.144802
C -5.840701 0.622247 -1.216532
C -6.370672 1.073274 -2.202117
H -6.839652 1.475162 -3.078114
40
TS_2_conf1 Eopt -
1523.936926
C 0.637544 -1.240141 0.815321
C 1.597893 -0.346704 1.221157
C 2.750862 -0.234883 0.428958
C 2.877499 -1.008779 -0.730927
C 1.869584 -1.884532 -1.120401
C 0.717030 -2.007696 -0.332910
H 1.481348 0.247116 2.122548
H 3.764259 -0.921497 -1.348594
H 1.977671 -2.461377 -2.034349
C 3.804730 0.719391 0.858820
O 3.733567 1.415385 1.858526
O 4.855062 0.742598 0.031999
C 5.918317 1.648302 0.385936
H 6.308357 1.380984 1.371888
H 5.526662 2.668437 0.424625
S -0.555699 -3.163914 -0.776222
C -2.003809 -2.380389 0.003122
C -1.824730 -1.863333 1.417186
H -2.783792 -3.150942 -0.030930
H -0.651670 -1.463499 1.459437
H -1.950017 -2.560954 2.242363
N -2.934854 -0.230824 0.183896
C -2.548016 -1.171689 -0.755690
O -2.673597 -1.045041 -1.957807
C -2.621709 -0.613715 1.490502
O -2.922203 0.030838 2.481085
C -3.572376 1.006346 -0.149298
C -4.907083 1.202017 0.194613
C -2.840298 1.990982 -0.807722
C -5.517699 2.412932 -0.124614
H -5.456044 0.417035 0.705738
C -3.462923 3.194107 -1.132290
H -1.799192 1.813836 -1.060610
C -4.798308 3.406276 -0.789034
H -6.557138 2.576861 0.142910
H -2.901817 3.966998 -1.648626
H -5.279133 4.347362 -1.039331
C 6.957447 1.530278 -0.631102
C 7.833182 1.449875 -1.457179
H 8.610200 1.375924 -2.191913
40
TS_2_conf2 Eopt -
1523.937077
C 0.715104 -1.456759 0.732453
C 1.762296 -0.639495 1.078899
C 2.871823 -0.612903 0.219784
C 2.871195 -1.391858 -0.943626
C 1.779207 -2.189194 -1.270547
C 0.668243 -2.225030 -0.417204
H 1.743508 -0.038957 1.983096
H 3.723954 -1.369813 -1.612699
H 1.789971 -2.771795 -2.187332
C 4.014608 0.262621 0.583321
O 4.060664 0.943372 1.593906
O 5.003573 0.231709 -0.320192
C 6.156398 1.051160 -0.053984
H 6.921588 0.675375 -0.734164
H 6.487481 0.898228 0.976020
S -0.714652 -3.277284 -0.783347
C -2.053654 -2.377353 0.063683
C -1.758025 -1.863066 1.459200
H -2.890518 -3.085929 0.081407
H -0.554441 -1.567330 1.440396
H -1.901853 -2.538814 2.299448
N -2.793284 -0.155686 0.261908
C -2.544466 -1.137926 -0.681655
O -2.733786 -1.016504 -1.876017
C -2.440076 -0.548255 1.555250
O -2.630047 0.130866 2.550049
C -3.361075 1.119410 -0.054253
C -4.664956 1.402554 0.342921
C -2.596195 2.050279 -0.752116
C -5.210581 2.647338 0.036887
H -5.240571 0.657531 0.883772
C -3.154130 3.288573 -1.062090
H -1.580333 1.804744 -1.046945
C -4.457895 3.587861 -0.666443
H -6.225601 2.879120 0.344869
H -2.567265 4.020242 -1.608981
H -4.888110 4.555708 -0.906537

C 5.890070 2.466202 -0.313507
C 5.688745 3.635590 -0.534668
H 5.506562 4.673986 -0.727941
40
TS_2_conf3 Eopt -1523.937023
C -0.760765 1.113106 0.663156
C -1.739201 0.167181 0.843873
C -2.782653 0.130394 -0.094120
C -2.786733 1.029327 -1.167602
C -1.763117 1.957407 -1.327766
C -0.719833 2.005264 -0.393397
H -1.718508 -0.523441 1.681359
H -3.588271 1.001407 -1.897091
H -1.773726 2.633689 -2.177833
C -3.854650 -0.879836 0.092704
O -3.893796 -1.674120 1.017074
O -4.786635 -0.833680 -0.868871
C -5.867057 -1.779986 -0.777972
H -5.467682 -2.778121 -0.582717
H -6.325394 -1.767060 -1.767461
S 0.565107 3.221425 -0.541242
C 1.928637 2.373490 0.318246
C 1.593899 1.691151 1.630447
H 2.683637 3.154472 0.469030
H 0.435537 1.274806 1.481691
H 1.599439 2.288468 2.539539
N 2.894655 0.232273 0.365830
C 2.595147 1.270047 -0.500657
O 2.866980 1.285916 -1.685108
C 2.410882 0.452206 1.657788
O 2.606040 -0.300771 2.596782
C 3.598509 -0.949769 -0.028778
C 4.896958 -1.157989 0.427887
C 2.965393 -1.868672 -0.861679
C 5.572025 -2.314540 0.043453
H 5.368601 -0.424643 1.074923
C 3.652385 -3.016978 -1.248750
H 1.950460 -1.682830 -1.200267
C 4.952511 -3.241032 -0.795299
H 6.583950 -2.488004 0.396705
H 3.168984 -3.737844 -1.901072
H 5.483697 -4.139365 -1.095772
C -6.841864 -1.396244 0.243176
C -7.660718 -1.083317 1.072904
H -8.384280 -0.806263 1.813335
40
TS_3_conf1 Eopt -1523.962706
C -0.984171 -1.658925 1.368804
C 0.294321 -1.237121 1.703790
C 1.341689 -1.381136 0.789434
C 1.108299 -1.963479 -0.461068
C -0.176886 -2.347421 -0.816151
C -1.237489 -2.193869 0.094155
H 0.478751 -0.784615 2.672532
H 1.917411 -2.078539 -1.173525
H -0.365852 -2.757209 -1.803218
C 2.670894 -0.822184 1.153198
O 2.947052 -0.364041 2.249475
O 3.536081 -0.844741 0.136147
C 4.827688 -0.260695 0.392079
H 5.321852 -0.811341 1.197141
H 4.697035 0.779972 0.702012
S -2.859949 -2.660248 -0.374148
C -3.819778 -0.505881 -0.850322
C -4.098941 -0.059399 0.416275
H -4.511381 -0.959602 -1.548885
H -1.794100 -1.555999 2.084565
H -4.925433 -0.330232 1.058832
N -2.203453 1.065420 -0.284673
C -2.637586 0.280424 -1.348763
O -2.148630 0.258644 -2.460569
C -3.036260 0.874895 0.830476
O -2.886562 1.409585 1.914393
C -1.049286 1.903613 -0.289121
C -1.155279 3.222202 0.151367
C 0.172983 1.388866 -0.714005
C -0.014071 4.020395 0.188719
H -2.116242 3.618414 0.462989
C 1.307184 2.195551 -0.679464
H 0.236516 0.366213 -1.064425
C 1.217447 3.509686 -0.221931
H -0.091316 5.045847 0.537246
H 2.261775 1.788800 -1.001358
H 2.104008 4.136024 -0.188189
C 5.600737 -0.339264 -0.842419
C 6.259082 -0.391324 -1.852161
H 6.843349 -0.436886 -2.749556
40
TS_3_conf11 Eopt -1523.955070
C -1.843096 -2.218855 0.370519
C -3.177391 -2.037813 0.036705
C -3.769179 -0.777328 0.171084
C -3.017890 0.304999 0.642244
C -1.682503 0.126626 0.977139
C -1.083968 -1.137054 0.847295
H -3.762982 -2.874284 -0.330192
H -3.468963 1.285430 0.745410
H -1.097594 0.966964 1.337152
C -5.202636 -0.631086 -0.203036
O -5.895286 -1.539731 -0.629808
O -5.662713 0.610109 -0.024001
C -7.045112 0.826632 -0.368094
H -7.675475 0.171603 0.239519
H -7.196626 0.584789 -1.423705
S 0.601821 -1.358499 1.271729
C 1.464611 -1.195510 -0.960788
C 1.166067 0.105133 -1.272770

H	0.945406	-2.083801	-1.296709
H	-1.384364	-3.196695	0.262415
H	0.238522	0.510879	-1.653257
N	3.363973	0.075840	-0.559029
C	2.922993	-1.241203	-0.588884
O	3.598920	-2.220721	-0.344693
C	2.341252	0.943504	-0.963048
O	2.451260	2.153461	-1.052657
C	4.671404	0.489824	-0.163471
C	4.820399	1.283374	0.971929
C	5.773413	0.094501	-0.918206
C	6.096475	1.693211	1.351241
H	3.946857	1.573834	1.547920
C	7.046994	0.497113	-0.522130
H	5.632723	-0.518575	-1.803142
C	7.209018	1.298324	0.608033
H	6.220318	2.315853	2.232095
H	7.911570	0.189547	-1.102526
H	8.202979	1.615029	0.910032
C	-7.352034	2.228914	-0.108402
C	-7.630490	3.384658	0.097628
H	-7.876714	4.411808	0.279865

40

TS_3_conf12 Eopt -1523.955355

C	-1.503723	1.032912	1.276452
C	-2.807711	1.425909	1.010431
C	-3.737548	0.495373	0.535644
C	-3.357372	-0.835984	0.329917
C	-2.054063	-1.231902	0.595235
C	-1.115802	-0.301476	1.072689
H	-3.105430	2.457533	1.166431
H	-4.073199	-1.561982	-0.038828
H	-1.758045	-2.263356	0.431575
C	-5.121813	0.965890	0.255409
O	-5.499713	2.115028	0.411402
O	-5.920521	-0.006697	-0.192615
C	-7.276290	0.377298	-0.494911
H	-7.268255	1.148495	-1.270339
H	-7.746258	0.784057	0.404659
S	0.532038	-0.798625	1.402007
C	1.260924	-0.739958	-0.882834
C	1.285648	0.610301	-1.114656
H	0.508339	-1.446465	-1.208145
H	-0.781115	1.757004	1.638844
H	0.467435	1.260750	-1.392808
N	3.446470	-0.019098	-0.587807
C	2.680792	-1.177096	-0.642270
O	3.100723	-2.310726	-0.521319
C	2.655057	1.103451	-0.866194
O	3.061824	2.251284	-0.894872
C	4.841650	0.018508	-0.290736
C	5.287385	-0.443592	0.945826
C	5.732523	0.515566	-1.239561
C	6.650357	-0.413596	1.231234
H	4.573943	-0.820685	1.672636
C	7.092099	0.556062	-0.938387
H	5.364214	0.864460	-2.199436
C	7.551899	0.088942	0.292825
H	7.005594	-0.776577	2.190865
H	7.792053	0.946660	-1.670801
H	8.613243	0.116648	0.521229
C	-7.981902	-0.813528	-0.955220
C	-8.589097	-1.783137	-1.338461
H	-9.126994	-2.645617	-1.678706

40

TS_3_conf13 Eopt -1523.964806

C	-1.223427	-2.366862	0.677425
C	0.115589	-2.332697	1.038014
C	1.070262	-1.842964	0.141368
C	0.686681	-1.422084	-1.136185
C	-0.648827	-1.467888	-1.505785
C	-1.621214	-1.923746	-0.596604
H	0.418040	-2.652706	2.029642
H	1.423366	-1.042282	-1.834998
H	-0.947843	-1.134678	-2.494216
C	2.471250	-1.694164	0.619488
O	2.878209	-2.101458	1.694911
O	3.239917	-1.027007	-0.244130
C	4.587206	-0.753990	0.184454
H	5.122643	-1.697287	0.323076
H	4.560768	-0.217910	1.137402
S	-3.311540	-1.920322	-1.047137
C	-3.904127	0.339301	-0.507372
C	-3.943463	0.326060	0.863496
H	-4.750397	0.290149	-1.180867
H	-1.967599	-2.722190	1.382917
H	-4.738391	-0.017550	1.511105
N	-1.935104	1.306123	0.261854
C	-2.644536	1.054407	-0.910804
O	-2.292576	1.353847	-2.033696
C	-2.679942	0.883665	1.376474
O	-2.334053	1.014441	2.537393
C	-0.619459	1.855253	0.318391
C	0.352044	1.226071	1.095533
C	-0.315078	3.004147	-0.410854
C	1.645287	1.740123	1.125186
H	0.104114	0.336227	1.662262
C	0.985547	3.503580	-0.383672
H	-1.082905	3.502057	-0.993178
C	1.967816	2.873229	0.379561
H	2.403051	1.242592	1.724049
H	1.226969	4.392841	-0.958164
H	2.980278	3.265746	0.396067
C	5.221024	0.059721	-0.847015
C	5.764183	0.735971	-1.685668
H	6.245401	1.335900	-2.432302

40

TS_3_conf8 Eopt -1523.955053
C -1.819887 -2.120498 0.382063
C -3.158101 -1.999593 0.038042
C -3.807343 -0.767208 0.169176
C -3.109696 0.347214 0.647791
C -1.769910 0.229407 0.992172
C -1.113873 -1.005823 0.865390
H -3.702519 -2.861074 -0.334417
H -3.606601 1.305485 0.749103
H -1.226634 1.094759 1.358117
C -5.242907 -0.685348 -0.215652
O -5.891632 -1.624598 -0.645215
O -5.758654 0.534735 -0.042642
C -7.147238 0.689278 -0.395259
H -7.751381 0.007309 0.209397
H -7.281541 0.439905 -1.451444
S 0.576511 -1.152994 1.302950
C 1.423386 -1.012078 -0.936320
C 1.206401 0.314078 -1.203856
H 0.838699 -1.854617 -1.282720
H -1.316438 -3.076407 0.277037
H 0.300612 0.791948 -1.551914
N 3.410263 0.116282 -0.534857
C 2.882516 -1.167070 -0.597907
O 3.496091 -2.197197 -0.402230
C 2.441188 1.062063 -0.895313
O 2.631539 2.263883 -0.954288
C 4.753538 0.429585 -0.167771
C 4.988363 1.182366 0.980817
C 5.804226 -0.021388 -0.963459
C 6.299859 1.493496 1.332190
H 4.153426 1.517892 1.588648
C 7.113104 0.282501 -0.595436
H 5.597034 -0.600867 -1.857961
C 7.361425 1.041797 0.547951
H 6.491170 2.083368 2.223486
H 7.937851 -0.069175 -1.207845
H 8.382873 1.281603 0.828166
C -7.518150 2.076581 -0.138942
C -7.851264 3.218287 0.064346
H -8.145122 4.233331 0.243994
63
complexA_6sol_conf_10
Eopt -2358.102508
C 2.254543 -1.263396 -0.933186
C 1.934696 0.081834 -1.176019
C 2.805232 1.124534 -0.910674
C 4.059949 0.837447 -0.379306
C 4.402899 -0.490821 -0.111315
C 3.518136 -1.522156 -0.385132
H 2.509314 2.146228 -1.123465
H 5.375891 -0.720716 0.310388
H 3.818917 -2.541897 -0.173377
C 5.044322 1.911969 -0.083368
O 6.140133 1.706605 0.415925
O 4.609049 3.122796 -0.418983
C 5.498424 4.224105 -0.168490
H 4.961351 5.107837 -0.509207
H 6.422519 4.095620 -0.735922
N 0.010463 -0.119417 -2.528740
N 0.612802 0.398815 -1.672947
S 1.089390 -2.546766 -1.281102
C 1.530845 -3.800639 -0.045423
H 0.692577 -4.502022 -0.029651
H 2.438587 -4.340301 -0.322226
H 1.639453 -3.348660 0.942896
H 5.714178 4.296121 0.899568
C -1.885991 1.333536 -0.852940
H -2.492854 2.217053 -1.057776
H -1.810493 0.769965 -1.802936
S -0.218913 1.825888 -0.439368
C -2.577773 0.468332 0.220127
H -2.750525 1.086086 1.103329
C -3.963777 0.069991 -0.289098
O -4.806145 0.975090 -0.486443
N -1.763125 -0.665200 0.595968
N -4.217196 -1.210238 -0.518551
H -3.507378 -1.920486 -0.328038
C -5.507850 -1.662919 -1.010494
H -5.463076 -2.745830 -1.127734
H -6.302665 -1.412205 -0.302561
H -5.735106 -1.206659 -1.977593
C -1.690534 -1.232215 1.814312
O -1.024292 -2.279206 1.965110
C -2.407077 -0.581832 2.961356
H -2.004637 0.421297 3.141562
H -3.477004 -0.484174 2.749494
H -2.268299 -1.190185 3.855242
H -1.273611 -1.166025 -0.141977
H -1.869720 2.715456 2.478168
H -6.571051 1.039823 -0.957261
H -4.514697 2.605743 0.192691
H -1.903722 -3.427982 0.570692
H 0.778510 -1.495343 2.265040
O -7.512815 1.198217 -1.174056
H -7.506133 1.997050 -1.718583
O -4.393553 3.504161 0.573623
H -5.141833 3.618161 1.176164
O -2.539258 -3.646100 -0.135857
H -3.205696 -4.196321 0.298759
O 1.540360 -0.943812 2.518495
H 1.713565 -0.399159 1.737423
O -2.222275 3.602623 2.325577
H -2.961084 3.479403 1.688611
O 6.257054 -3.356322 0.911316
H 6.176085 -3.000044 0.015238
H 5.608069 -2.850552 1.420692
63

complexA_6sol_conf_15
Eopt -2358.110446

C	3.412783	1.652825	-0.556119
C	2.771316	0.451625	-0.911344
C	3.366910	-0.788982	-0.767554
C	4.652124	-0.873667	-0.243325
C	5.307538	0.298599	0.145031
C	4.699390	1.534908	-0.010121
H	2.841787	-1.687621	-1.073625
H	6.306455	0.254037	0.564042
H	5.244881	2.420528	0.291507
C	5.271649	-2.218610	-0.115535
O	4.733693	-3.250117	-0.489000
O	6.474336	-2.185054	0.450296
C	7.147768	-3.444960	0.612991
H	6.557393	-4.105892	1.250980
H	8.096115	-3.202247	1.089725
N	0.894033	1.259093	-2.116480
N	1.417573	0.496477	-1.414877
S	2.613057	3.206724	-0.785480
C	3.564187	4.302938	0.300229
H	4.562917	4.500445	-0.094060
H	3.002897	5.240668	0.319366
H	3.620096	3.901118	1.314600
H	7.316863	-3.907861	-0.361489
C	-1.237803	-0.386989	-1.059999
H	-0.943994	0.304541	-1.875208
H	-1.819900	-1.197400	-1.505346
S	0.309394	-1.033182	-0.445048
C	-2.040071	0.393814	-0.012592
H	-1.408248	1.207027	0.362329
C	-3.300205	1.101088	-0.535951
O	-4.052698	1.679647	0.276351
N	-2.379772	-0.407942	1.151764
N	-3.527532	1.137770	-1.844637
H	-2.979335	0.553763	-2.461538
C	-4.713187	1.770051	-2.400887
H	-4.657669	1.698661	-3.486931
H	-5.621846	1.270751	-2.051873
H	-4.752111	2.823162	-2.112119
C	-3.280803	-1.388763	1.132011
O	-3.895540	-1.696459	0.082224
C	-3.552098	-2.116419	2.414435
H	-3.314466	-3.175309	2.272545
H	-2.973907	-1.725615	3.254217
H	-4.620567	-2.039444	2.638244
H	-1.967072	-0.127761	2.047183
H	0.915847	0.336898	1.446992
H	-5.776107	0.661431	0.340780
H	-3.335388	2.391425	1.782367
H	-4.919278	-3.182778	0.054781
H	-4.254292	-1.597726	-1.741574
O	-6.331339	-0.138184	0.368419
H	-5.679097	-0.846854	0.226764
O	-2.947353	2.845789	2.562307
H	-3.659090	2.859383	3.217584
O	-5.466554	-3.994695	-0.011488
H	-5.468643	-4.207611	-0.954770
O	-4.556974	-1.735487	-2.659015
H	-5.154917	-2.493405	-2.599190
O	1.197868	0.921879	2.176169
H	1.826905	0.386162	2.680560
O	-1.259870	0.832144	3.512516
H	-1.781663	1.612322	3.221342
H	-0.375436	0.940283	3.102024

63
complexA_6sol_conf_21
Eopt -2358.107378

C	3.180653	1.732734	-0.609015
C	2.696721	0.453666	-0.941342
C	3.433714	-0.701896	-0.753520
C	4.710794	-0.616088	-0.209602
C	5.211549	0.636785	0.157238
C	4.460942	1.785893	-0.038572
H	3.026484	-1.664869	-1.043685
H	6.200542	0.723898	0.592830
H	4.888433	2.737971	0.251428
C	5.490568	-1.869791	-0.038196
O	5.080793	-2.973863	-0.364439
O	6.684681	-1.665560	0.509485
C	7.511220	-2.823963	0.713751
H	7.723040	-3.306719	-0.242572
H	7.016425	-3.522721	1.391492
N	0.759455	1.002043	-2.193535
N	1.356360	0.323831	-1.464518
S	2.201106	3.169734	-0.894901
C	3.010023	4.412354	0.146297
H	3.100443	4.065253	1.178177
H	3.984083	4.704089	-0.251655
H	2.346129	5.280389	0.119512
H	8.428641	-2.444515	1.160697
C	-1.196837	-0.784840	-1.054085
H	-0.971117	-0.112043	-1.905239
H	-1.736100	-1.648268	-1.450688
S	0.409718	-1.297936	-0.464091
C	-2.018270	-0.018280	-0.011296
H	-1.409676	0.817683	0.352514
C	-3.300442	0.649248	-0.530135
O	-4.058933	1.202884	0.293319
N	-2.330591	-0.818860	1.160144
N	-3.541363	0.681685	-1.835491
H	-2.976533	0.120542	-2.458524
C	-4.758357	1.266632	-2.376440
H	-4.767666	1.094730	-3.452394
H	-5.641749	0.802561	-1.928306
H	-4.784126	2.342378	-2.186005
C	-3.245445	-1.792160	1.153195
O	-3.868139	-2.100442	0.112791
C	-3.506037	-2.509654	2.445115
H	-4.567457	-2.411785	2.692292

H -3.290902 -3.573396 2.304134
H -2.903469 -2.126110 3.271109
H -1.931084 -0.515368 2.053184
H 0.926385 0.132768 1.428894
H -5.208102 2.621518 0.222520
H -3.355451 1.990184 1.762143
H -5.024419 -3.474584 0.103321
H -4.092609 -2.096548 -1.723797
O -5.775772 3.405249 0.367864
H -5.527977 3.714544 1.250046
O -2.990725 2.485606 2.527718
H -3.724969 2.538659 3.155605
O -5.653833 -4.224758 0.025164
H -5.939708 -4.201646 -0.898603
O -4.337654 -2.263862 -2.654168
H -4.619312 -3.189211 -2.662228
O 1.146610 0.767107 2.137139
H 1.868348 0.344281 2.624052
O -1.273764 0.509833 3.511570
H -1.829178 1.266812 3.221538
H -0.398444 0.658479 3.093965
63
complexA_6sol_conf_25
Eopt -2358.107422
C 2.830272 1.709649 0.279183
C 2.609948 0.444575 0.840849
C 3.546818 -0.570095 0.827134
C 4.787968 -0.325712 0.236754
C 5.038277 0.923117 -0.335762
C 4.078606 1.926428 -0.316587
H 3.308659 -1.531424 1.269641
H 5.998327 1.117827 -0.802178
H 4.313163 2.882328 -0.769889
C 5.848071 -1.365812 0.191662
O 6.937725 -1.201077 -0.336475
O 5.490570 -2.500906 0.786559
C 6.457247 -3.564891 0.785863
H 5.973983 -4.389841 1.306804
H 7.358546 -3.252748 1.317619
N 0.843458 0.524435 2.429995
N 1.304783 0.160901 1.418536
S 1.546933 2.918477 0.364292
C 2.087164 4.162798 -0.833846
H 1.252739 4.863578 -0.918944
H 2.968327 4.703635 -0.482640
H 2.274598 3.710584 -1.810331
H 6.700004 -3.850374 -0.239798
C -1.285598 -0.687022 0.828022
H -1.845957 -1.623637 0.841797
H -1.057239 -0.423533 1.875652
S 0.306237 -0.939181 0.049492
C -2.091475 0.463333 0.222501
H -1.429184 1.331930 0.130814
C -2.600456 0.160597 -1.193656
O -2.349407 -0.916809 -1.768931
N -3.164145 0.892469 1.106755
N -3.272732 1.136558 -1.795617
H -3.441504 1.996220 -1.289115
C -3.796875 1.004096 -3.144626
H -2.983026 0.911435 -3.869145
H -4.442657 0.125029 -3.219459
H -4.378690 1.896723 -3.373220
C -4.317300 0.232292 1.259782
O -4.581727 -0.784083 0.579424
C -5.296073 0.775062 2.259884
H -4.918413 1.652248 2.789439
H -6.220566 1.039224 1.736735
H -5.531582 -0.012383 2.982179
H -2.951517 1.697881 1.700557
H 0.369493 0.977124 -1.798267
H -2.106005 -2.640255 -1.323617
H -1.419131 -0.935331 -3.327642
H -6.238966 -1.465870 0.828144
H -4.037906 -2.538161 1.021968
O -2.033575 -3.598191 -1.127693
H -2.580739 -4.027717 -1.799999
O -0.779649 -0.834395 -4.063204
H -0.123892 -1.530298 -3.915173
O -7.142589 -1.824550 0.966670
H -7.528014 -1.241940 1.635731
O -3.737275 -3.460683 1.118394
H -3.128579 -3.582668 0.358981
O 0.375018 1.350250 -2.695120
H 0.001601 0.628684 -3.245592
O -1.966235 3.122156 2.506006
H -1.708961 3.656996 1.741303
H -1.159599 2.641285 2.741437
63
complexA_6sol_conf_26
Eopt -2358.111672
C 2.915262 0.943491 1.502193
C 2.567853 -0.278352 0.909143
C 3.457440 -1.013611 0.142126
C 4.760388 -0.555038 -0.027585
C 5.145472 0.649094 0.566979
C 4.232497 1.383938 1.308054
H 3.147247 -1.956229 -0.296677
H 6.153091 1.028165 0.440453
H 4.537459 2.333929 1.735513
C 5.694054 -1.378927 -0.842574
O 5.389900 -2.450088 -1.344555
O 6.896086 -0.826424 -0.969527
C 7.863994 -1.553275 -1.745843
H 7.504171 -1.680628 -2.768895
H 8.059000 -2.524669 -1.286705
N 0.674161 -1.128100 2.076325
N 1.252307 -0.866738 1.103973
S 1.896953 1.969828 2.522949
C 0.252066 1.944499 1.748558
H 0.331300 1.839561 0.664015

H -0.378759 1.159947 2.167482
H -0.189670 2.916895 1.976144
H 8.761753 -0.937457 -1.731260
C -1.088115 -1.881588 -0.186858
H -1.226235 -2.889381 -0.587103
H -0.975594 -1.981995 0.910022
S 0.464390 -1.228632 -0.791552
C -2.318778 -1.025371 -0.516958
H -2.273496 -0.705460 -1.557339
C -3.583196 -1.858457 -0.283086
O -3.768444 -2.422101 0.820524
N -2.394695 0.160814 0.319660
N -4.441015 -1.942233 -1.289767
H -4.272554 -1.376435 -2.126096
C -5.690159 -2.678363 -1.185974
H -5.500511 -3.706139 -0.867092
H -6.367813 -2.203963 -0.470373
H -6.160206 -2.689966 -2.169298
C -2.795433 1.364107 -0.100865
O -3.021571 1.615883 -1.309526
C -2.954027 2.435458 0.936883
H -2.776926 2.074121 1.951864
H -2.257700 3.249284 0.713835
H -3.969595 2.836087 0.864057
H -2.287916 0.011597 1.324865
H -0.007176 0.929535 -1.729335
H -3.048104 -1.636351 2.368153
H -5.325562 -2.017977 1.739381
H -2.956569 3.430681 -1.760807
H -3.647450 0.637464 -2.715878
O -2.673143 -1.013888 3.024628
H -3.435892 -0.509526 3.340746
O -6.057952 -1.746605 2.328270
H -5.617349 -1.480569 3.147137
O -2.730258 4.362457 -1.954189
H -1.804520 4.430887 -1.642657
O -3.985679 0.043627 -3.417704
H -4.878967 0.364230 -3.603630
O -0.337774 1.671236 -2.270235
H -1.304579 1.634009 -2.136326
O -0.022129 4.150468 -1.067301
H 0.503613 4.685847 -1.677751
H -0.019476 3.247010 -1.458373
63
complexA_6sol_conf_28
Eopt -2358.111949
C 2.851113 0.957200 1.487216
C 2.494630 -0.200854 0.782954
C 3.361807 -0.843950 -0.084853
C 4.655887 -0.356937 -0.246124
C 5.051359 0.783297 0.457116
C 4.157130 1.430111 1.297566
H 3.042347 -1.738405 -0.609281
H 6.052058 1.183059 0.339648
H 4.469293 2.333969 1.810955
C 5.568963 -1.084702 -1.169127
O 5.245509 -2.084578 -1.791650
O 6.775470 -0.532893 -1.246450
C 7.724048 -1.170131 -2.119420
H 8.631560 -0.574132 -2.038237
H 7.351921 -1.162352 -3.145951
N 0.676125 -1.230741 1.920299
N 1.193396 -0.824000 0.963488
S 1.850445 1.852763 2.641782
C 0.197658 1.921396 1.883873
H 0.270292 1.963011 0.794841
H -0.425463 1.084214 2.198674
H -0.248331 2.851614 2.242494
H 7.908002 -2.194396 -1.788643
C -1.177216 -1.737622 -0.314695
H -1.282883 -2.706931 -0.808715
H -0.996965 -1.936775 0.760196
S 0.300343 -0.931496 -0.915822
C -2.472500 -0.933315 -0.482773
H -2.524215 -0.524838 -1.491793
C -3.668734 -1.859501 -0.234319
O -3.737300 -2.541112 0.814560
N -2.544210 0.170092 0.459778
N -4.609271 -1.884617 -1.167268
H -4.540668 -1.233823 -1.954465
C -5.812198 -2.688067 -1.026679
H -6.380805 -2.386404 -0.142152
H -6.425807 -2.540422 -1.915255
H -5.557922 -3.747537 -0.938783
C -3.013166 1.388135 0.176077
O -3.347833 1.728459 -0.984817
C -3.106324 2.365752 1.309891
H -2.857202 1.920812 2.275299
H -2.427852 3.200766 1.109861
H -4.124862 2.763545 1.341578
H -2.349767 -0.057323 1.436601
H -0.325536 1.313616 -1.620856
H -2.971010 -1.835316 2.377715
H -2.503742 -3.874099 1.246954
H -3.446648 3.574588 -1.279009
H -4.067388 0.847084 -2.414483
O -2.567756 -1.258706 3.058583
H -3.319820 -0.821861 3.482089
O -1.900324 -4.592078 1.520819
H -1.021986 -4.188374 1.484797
O -3.292897 4.533774 -1.393965
H -2.357501 4.637816 -1.123123
O -4.439516 0.298304 -3.135432
H -5.363406 0.575292 -3.206976
O -0.732095 2.067731 -2.086335
H -1.686414 1.950674 -1.914198
O -0.535245 4.443437 -0.665111
H -0.070458 5.063353 -1.244258
H -0.492562 3.580597 -1.136805
63

complexA_6sol_conf_3 Eopt
-2358.113883

C	-1.972699	2.070325	0.036947
C	-2.035421	0.935890	-0.795973
C	-3.118191	0.071505	-0.799626
C	-4.184634	0.303797	0.061371
C	-4.133954	1.400613	0.926713
C	-3.053165	2.265715	0.912524
H	-3.142088	-0.772750	-1.478423
H	-4.954251	1.585039	1.612351
H	-3.059817	3.112847	1.587339
C	-5.367748	-0.594434	0.096069
O	-6.326698	-0.421791	0.833118
O	-5.274913	-1.611405	-0.756301
C	-6.376554	-2.534285	-0.787078
H	-7.293382	-2.014044	-1.072134
H	-6.495631	-3.008040	0.189592
N	-0.124786	1.369009	-2.109614
N	-0.907799	0.637318	-1.650540
S	-0.603600	3.178162	-0.030237
C	-0.736274	4.053634	1.551219
H	-1.569948	4.759268	1.558999
H	-0.815726	3.353742	2.386557
H	0.198273	4.613258	1.644117
H	-6.108240	-3.274136	-1.539402
C	1.114288	-1.291407	-1.905809
H	1.476074	-1.995725	-2.660170
H	1.341028	-0.274343	-2.267901
S	-0.673297	-1.426769	-1.842259
C	1.768707	-1.575538	-0.546794
H	1.480610	-2.588944	-0.246941
C	3.302411	-1.617974	-0.530042
O	3.887069	-1.926604	0.530894
N	1.283287	-0.708518	0.515504
N	3.970232	-1.407399	-1.657942
H	3.478171	-1.040199	-2.461796
C	5.423809	-1.426463	-1.702533
H	5.842688	-0.637106	-1.071041
H	5.800866	-2.393378	-1.361137
H	5.734307	-1.265089	-2.734550
C	1.806637	0.474488	0.844247
O	2.724903	1.001670	0.174454
C	1.307217	1.100738	2.113806
H	0.256534	0.866301	2.303398
H	1.905662	0.700580	2.941680
H	1.450841	2.181049	2.078274
H	0.516496	-1.070472	1.087743
H	-1.512201	-1.860034	0.868208
H	4.863044	-0.505411	1.377717
H	2.997213	-2.233680	2.041206
H	3.097798	2.769508	0.397497
H	2.925390	1.451545	-1.609061
O	5.091201	0.379879	1.717876
H	4.403714	0.931744	1.307204
O	2.546948	-2.482586	2.880510
H	2.051487	-1.690341	3.134124
O	3.332534	3.720673	0.431427
H	3.373190	3.985764	-0.497759
O	3.026127	1.837070	-2.501626
H	2.119470	1.862462	-2.840466
O	-1.110967	-1.860430	1.750678
H	-0.640512	-2.723865	1.812067
O	0.505507	-4.095989	1.936057
H	1.262719	-3.572042	2.289567
H	0.219773	-4.652175	2.674262

63
complexA_6sol_conf_31
Eopt -2358.109697

C	-3.980119	-1.423218	-0.272333
C	-2.800140	-0.702784	-0.043922
C	-2.741227	0.677344	-0.031940
C	-3.911824	1.403920	-0.244791
C	-5.104194	0.717076	-0.485594
C	-5.140314	-0.670676	-0.503079
H	-1.793373	1.172964	0.149379
H	-6.019720	1.272429	-0.660797
H	-6.085745	-1.164765	-0.692765
C	-3.915645	2.888881	-0.224643
O	-4.905463	3.569463	-0.449852
O	-2.725967	3.406110	0.068475
C	-2.633377	4.839417	0.112281
H	-3.309521	5.235624	0.872775
H	-2.872843	5.260367	-0.866494
N	-1.254358	-2.028924	1.168914
N	-1.554990	-1.414003	0.216911
S	-3.932325	-3.186158	-0.249762
C	-5.581441	-3.644500	-0.837021
H	-5.564471	-4.734430	-0.916973
H	-6.356968	-3.355477	-0.124478
H	-5.783836	-3.219478	-1.822722
H	-1.598380	5.051758	0.375061
C	1.124706	-1.579515	-0.447095
H	0.802146	-2.331263	0.291087
H	1.831249	-2.055895	-1.132157
S	-0.354200	-1.145922	-1.363705
C	1.733187	-0.367715	0.268751
H	0.963413	0.064109	0.918664
C	2.903347	-0.661031	1.218382
O	3.491205	0.294442	1.767485
N	2.116231	0.694455	-0.643080
N	3.220901	-1.921762	1.489657
H	2.810870	-2.663393	0.937894
C	4.361536	-2.257916	2.327370
H	4.231182	-1.849211	3.332285
H	4.429387	-3.343911	2.389327
H	5.286771	-1.859968	1.898902
C	3.259183	0.680311	-1.333149
O	3.999447	-0.328256	-1.349030
C	3.647278	1.934242	-2.058448
H	2.841650	2.670757	-2.088535

H 4.508756 2.370436 -1.540075
H 3.957879 1.682844 -3.075928
H 1.573673 1.559615 -0.599960
H -0.108664 2.898364 0.450983
H 5.243801 0.782594 1.236962
H 3.129831 2.049388 1.519666
H 5.738979 -0.102622 -1.613014
H 4.340037 -2.144220 -1.220557
O 6.133587 1.133613 1.046081
H 6.363505 0.770460 0.166003
O 3.024879 3.024162 1.448264
H 3.614064 3.268670 0.719610
O 6.705138 0.061127 -1.528045
H 7.085279 -0.811638 -1.355246
O 4.653517 -3.066381 -1.283651
H 5.011127 -3.262483 -0.406724
O 0.682369 3.152391 -0.044758
H 1.409275 3.199168 0.616321
O -4.084920 -0.519516 3.151057
H -3.445839 -1.145336 2.779994
H -4.459671 -0.080772 2.373331
63
complexA_6sol_conf_36
Eopt -2358.112647
C 2.354251 -2.117395 0.661571
C 2.049649 -1.304843 -0.442582
C 2.972700 -0.444927 -1.006752
C 4.270583 -0.414335 -0.496939
C 4.618043 -1.237449 0.572130
C 3.660379 -2.063992 1.151305
H 2.699303 0.179670 -1.851095
H 5.622500 -1.214191 0.979973
H 3.925421 -2.669201 2.011770
C 5.236005 0.546081 -1.095955
O 4.890349 1.554839 -1.698229
O 6.499961 0.205726 -0.904960
C 7.501114 1.110863 -1.407851
H 7.387552 2.088975 -0.936470
H 7.414026 1.195923 -2.492695
N 0.068837 -2.236504 -1.362365
N 0.719746 -1.334055 -1.028391
S 1.174509 -3.186044 1.460632
C 0.080956 -1.924592 2.185902
H 0.618545 -1.342719 2.937198
H -0.320743 -1.263400 1.415309
H -0.744057 -2.460210 2.661168
H 8.453727 0.660795 -1.134306
C -1.604871 0.145998 -1.664652
H -1.795473 0.468671 -2.692122
H -1.599064 -0.961810 -1.668362
S 0.031874 0.638269 -1.146400
C -2.724519 0.677390 -0.761233
H -2.718207 1.766010 -0.793970
C -4.081111 0.165236 -1.251233
O -4.223116 -1.031818 -1.588331
N -2.565588 0.281000 0.626231
N -5.071857 1.046700 -1.270852
H -4.898579 1.982053 -0.891947
C -6.429612 0.681653 -1.638343
H -7.019338 1.595092 -1.715729
H -6.437179 0.167976 -2.602570
H -6.879186 0.028299 -0.884707
C -2.288750 1.141376 1.614999
O -2.253041 2.379858 1.434780
C -2.018685 0.559719 2.972710
H -0.951737 0.673652 3.193214
H -2.578503 1.130017 3.718536
H -2.284888 -0.497141 3.040916
H -2.530361 -0.722418 0.814585
H 1.324205 0.740284 1.595700
H -3.302519 -2.268285 -0.477868
H -5.611290 -2.111404 -1.039138
H -3.699441 3.202730 0.605348
H -0.761136 3.140916 2.285020
O -2.864289 -2.668253 0.300046
H -3.590947 -2.868476 0.906640
O -6.215232 -2.798134 -0.690762
H -5.622670 -3.452640 -0.295597
O -4.464753 3.573952 0.120156
H -5.172875 3.621298 0.777384
O 0.086445 3.455932 2.655104
H 0.725480 2.748744 2.425105
O 1.925799 1.427626 1.917911
H 2.263248 1.873398 1.107809
O 2.824880 2.944584 -0.237756
H 2.039738 3.083442 -0.786005
H 3.451541 2.469160 -0.815264
63
complexA_6sol_conf_38
Eopt -2358.113431
C 2.347805 -1.840499 1.137171
C 1.988811 -1.308374 -0.111622
C 2.857496 -0.548867 -0.871911
C 4.157516 -0.342551 -0.411180
C 4.561072 -0.893499 0.803373
C 3.655620 -1.618350 1.571783
H 2.540641 -0.138586 -1.825389
H 5.567602 -0.732080 1.173092
H 3.962537 -2.005705 2.537483
C 5.061168 0.513713 -1.225280
O 4.652806 1.336845 -2.034927
O 6.343955 0.304660 -0.978686
C 7.286689 1.134877 -1.683185
H 7.107995 2.185078 -1.444493
H 7.199730 0.965896 -2.758190
N 0.040273 -2.507970 -0.749893
N 0.653826 -1.527680 -0.642768
S 1.232265 -2.754599 2.182320
C 0.092364 -1.409988 2.637116
H 0.613618 -0.653624 3.226923

H	-0.350693	-0.953604	1.749490
H	-0.700094	-1.860924	3.239148
H	8.265102	0.820684	-1.324265
C	-1.763633	-0.320212	-1.473383
H	-2.006290	-0.279737	-2.538812
H	-1.707893	-1.388888	-1.191091
S	-0.130532	0.339105	-1.174642
C	-2.868287	0.387459	-0.680386
H	-2.876978	1.443052	-0.948317
C	-4.232223	-0.224233	-1.015681
O	-4.387496	-1.465011	-1.079217
N	-2.667303	0.302860	0.754651
N	-5.229135	0.631286	-1.198168
H	-5.053124	1.628451	-1.044886
C	-6.590075	0.186081	-1.444771
H	-6.638182	-0.414517	-2.356886
H	-6.962616	-0.412354	-0.608008
H	-7.220479	1.067149	-1.564122
C	-2.408632	1.362334	1.532435
O	-2.415742	2.534517	1.093129
C	-2.107140	1.089225	2.977474
H	-1.050702	1.311658	3.161350
H	-2.704843	1.763995	3.596082
H	-2.307400	0.054340	3.261659
H	-2.605176	-0.635844	1.152934
H	1.028338	1.166546	1.300494
H	-3.423034	-2.420522	0.246577
H	-3.448032	-2.673270	-2.142857
H	-3.850110	3.160129	0.097746
H	-0.988545	3.538886	1.774807
O	-2.890469	-2.652273	1.033996
H	-3.542798	-2.806082	1.731392
O	-3.028886	-3.363200	-2.693622
H	-2.095271	-3.332187	-2.442073
O	-4.614033	3.415394	-0.459369
H	-5.318986	3.620627	0.170123
O	-0.147525	3.942785	2.066269
H	0.514121	3.235543	1.912843
O	1.678278	1.852578	1.517464
H	1.999045	2.179829	0.646314
O	2.524946	2.927102	-0.922250
H	1.727086	2.912747	-1.469934
H	3.156107	2.341878	-1.382753

63
complexA_6sol_conf_44
Eopt -2358.106971

C	3.431658	1.974604	0.168108
C	2.635555	0.949213	-0.363930
C	3.109604	-0.324589	-0.613971
C	4.446600	-0.614637	-0.338497
C	5.263154	0.383577	0.196067
C	4.767007	1.655794	0.446564
H	2.443102	-1.076484	-1.022464
H	6.302796	0.167409	0.418773
H	5.433376	2.401757	0.862480
C	5.019079	-1.961561	-0.591338
O	6.175684	-2.265995	-0.339689
O	4.140917	-2.808848	-1.122117
C	4.607567	-4.140472	-1.395342
H	4.923684	-4.624546	-0.468903
H	5.433254	-4.109666	-2.109413
N	0.789347	1.946147	-1.459630
N	1.228157	1.211736	-0.648247
S	2.720128	3.562958	0.460229
C	3.986871	4.396379	1.448882
H	3.538922	5.348887	1.743527
H	4.228466	3.824171	2.347584
H	4.887680	4.597142	0.865058
H	3.752274	-4.660286	-1.824045
C	-1.420695	0.730950	0.030482
H	-2.085618	0.808530	0.893407
H	-1.260129	1.747045	-0.358137
S	0.172741	0.149279	0.602891
C	-2.035072	-0.107621	-1.091761
H	-1.279382	-0.254167	-1.870388
C	-2.465949	-1.507340	-0.636006
O	-2.303589	-1.901200	0.535161
N	-3.123128	0.615214	-1.735432
N	-2.964747	-2.302147	-1.577789
H	-3.089163	-1.932982	-2.511592
C	-3.420855	-3.651037	-1.286897
H	-2.594690	-4.265577	-0.919069
H	-4.214071	-3.635289	-0.533942
H	-3.808801	-4.086615	-2.207279
C	-4.331451	0.776254	-1.177319
O	-4.659023	0.150788	-0.145525
C	-5.285333	1.713392	-1.857020
H	-6.168209	1.148820	-2.172616
H	-5.610055	2.465126	-1.131383
H	-4.844618	2.210639	-2.723680
H	-2.827486	1.260685	-2.470687
H	0.495326	-0.830449	3.689850
H	-2.603183	-1.158935	2.179703
H	-0.925614	-2.852440	1.271526
H	-6.351390	0.352922	0.469827
H	-4.376482	1.122362	1.519892
O	-2.707198	-0.860335	3.105641
H	-1.809936	-0.972431	3.486749
O	-0.213112	-3.302777	1.768662
H	-0.633133	-4.094528	2.133415
O	-7.243499	0.445968	0.868495
H	-7.070378	0.770876	1.762896
O	-4.147335	1.434645	2.413432
H	-3.579769	0.713563	2.760396
O	-0.109952	-1.545768	3.930199
H	-0.060920	-2.184239	3.182254
O	-1.561368	2.499471	-3.299063
H	-0.867570	2.371289	-2.626935
H	-1.990196	3.329961	-3.048733

63

complexA_6sol_conf_46
Eopt -2358.099980

C	-3.893166	-1.567842	-0.273370
C	-2.851250	-0.632761	-0.417553
C	-3.065038	0.733905	-0.437195
C	-4.360828	1.223796	-0.307208
C	-5.415910	0.322999	-0.137107
C	-5.185660	-1.044712	-0.122351
H	-2.231917	1.416413	-0.566701
H	-6.431648	0.684343	-0.023188
H	-6.031105	-1.709970	0.003361
C	-4.564473	2.695416	-0.354440
O	-3.660067	3.499442	-0.523502
O	-5.836463	3.048384	-0.194698
C	-6.128320	4.455698	-0.230577
H	-5.597982	4.969129	0.574085
H	-7.204793	4.527753	-0.083890
N	-1.041633	-2.015304	-1.078648
N	-1.487771	-1.107607	-0.504996
S	-3.553384	-3.297847	-0.264181
C	-5.057985	-3.992378	0.469042
H	-4.828476	-5.046834	0.643216
H	-5.287842	-3.515450	1.424633
H	-5.909508	-3.927051	-0.211328
H	-5.845646	4.872389	-1.199552
C	1.139908	-0.936762	0.469356
H	1.613634	-1.275524	1.392709
H	0.769832	-1.844964	-0.045295
S	-0.323199	0.022376	0.829649
C	2.106697	-0.254509	-0.504157
H	1.515701	0.108035	-1.350513
C	2.865979	0.987213	-0.019579
O	3.463003	1.675579	-0.877897
N	3.050240	-1.200034	-1.086332
N	2.852718	1.313518	1.265084
H	2.505549	0.642829	1.943215
C	3.630608	2.427193	1.783758
H	3.265293	3.372034	1.373920
H	3.517152	2.442936	2.867790
H	4.689278	2.312341	1.531809
C	4.037415	-1.802010	-0.414822
O	4.232368	-1.606759	0.804600
C	4.934785	-2.718112	-1.194665
H	4.615503	-2.843765	-2.231305
H	5.946952	-2.300500	-1.177635
H	4.964364	-3.692514	-0.698817
H	2.892080	-1.429177	-2.069091
H	-2.095896	-0.703034	2.570661
H	4.197141	3.370283	-0.881521
H	5.262916	1.314675	-1.096475
H	5.809083	-0.709024	1.268696
H	3.684028	-1.078978	2.469496
O	4.633698	4.232718	-1.033865
H	5.363204	4.026899	-1.634283
O	6.224684	1.155833	-1.178010
H	6.619538	2.036550	-1.105615
O	6.563034	-0.091931	1.321903
H	6.510759	0.385443	0.466565
O	3.375703	-0.780847	3.347425
H	4.048216	-0.147519	3.635409
O	-2.928241	-0.803509	3.062292
H	-3.613687	-0.591703	2.411764
O	1.886179	-1.502184	-3.718068
H	0.999078	-1.610480	-3.345222
H	1.911914	-0.578667	-4.006382

63

complexA_6sol_conf_51
Eopt -2358.108354

C	3.032063	-2.276323	-0.206272
C	2.250596	-1.236616	-0.753216
C	2.777577	0.005260	-1.067516
C	4.125324	0.256219	-0.842451
C	4.925570	-0.753282	-0.301782
C	4.389488	-1.991124	0.010207
H	2.148748	0.770841	-1.504838
H	5.978369	-0.567680	-0.116308
H	5.043108	-2.745022	0.430363
C	4.722538	1.584444	-1.132007
O	5.921905	1.808659	-1.126668
O	3.803745	2.518128	-1.377910
C	4.282817	3.848235	-1.645749
H	3.387529	4.442603	-1.819861
H	4.919638	3.844933	-2.532496
N	0.224143	-2.425746	-1.059775
N	0.837724	-1.450338	-0.947448
S	2.321735	-3.836222	0.193635
C	3.614305	-4.622666	1.190049
H	3.158930	-5.545457	1.559108
H	4.490156	-4.880152	0.590689
H	3.895406	-4.000221	2.042653
H	4.831831	4.226055	-0.780873
C	-1.784658	-0.393963	-0.682483
H	-2.466233	-0.007874	-1.439749
H	-1.582226	-1.454065	-0.942898
S	-0.218575	0.449596	-0.797076
C	-2.400482	-0.365181	0.727840
H	-1.647309	-0.719058	1.433067
C	-2.746145	1.059228	1.183297
O	-2.086468	1.605830	2.094084
N	-3.518512	-1.300701	0.865763
N	-3.735025	1.690128	0.562323
H	-4.246294	1.183478	-0.155768
C	-4.132692	3.043958	0.909303
H	-4.437649	3.100699	1.958035
H	-3.309176	3.743328	0.741320
H	-4.974370	3.322802	0.275592
C	-4.606384	-1.392130	0.086763
O	-4.841240	-0.578102	-0.832814
C	-5.560465	-2.515373	0.370233
H	-5.249844	-3.141724	1.208641

H -6.546534 -2.090091 0.579232
H -5.644254 -3.131931 -0.529957
H -3.418391 -1.994721 1.595729
H 0.849772 0.221938 1.355992
H -0.875834 2.980565 1.571904
H -1.081010 0.906558 3.372763
H -4.495089 0.532550 -2.357289
H -6.387276 -0.785885 -1.795293
O -0.298975 3.729194 1.332396
H 0.609390 3.359459 1.299917
O -0.459709 0.593015 4.067609
H -0.239734 1.386097 4.576179
O -4.349976 1.146034 -3.098256
H -3.537632 1.614749 -2.860446
O -7.190443 -0.840228 -2.354216
H -7.006731 -0.243715 -3.092934
O 1.469210 0.242469 2.109926
H 0.879074 0.374657 2.886532
O 2.348838 2.759618 1.319391
H 2.135834 1.845823 1.616706
H 2.637134 2.662023 0.397668
63
complexA_6sol_conf_55
Eopt -2358.110653
C -3.123812 1.168848 1.157741
C -2.936864 -0.193626 0.884590
C -3.873845 -0.966740 0.228170
C -5.072599 -0.378639 -0.174729
C -5.286852 0.978083 0.077022
C -4.327916 1.740664 0.732354
H -3.678891 -2.017418 0.037034
H -6.210099 1.452981 -0.235180
H -4.530838 2.789911 0.910602
C -6.076466 -1.229965 -0.865941
O -5.918730 -2.421943 -1.083709
O -7.167407 -0.560048 -1.225071
C -8.192283 -1.307135 -1.902347
H -8.558543 -2.109542 -1.258553
H -7.802057 -1.716634 -2.836357
N -1.245163 -1.003861 2.340230
N -1.684280 -0.827685 1.269378
S -1.854291 2.065613 2.003054
C -2.367622 3.789751 1.794684
H -2.500342 4.038476 0.739342
H -3.275061 4.007581 2.361544
H -1.544680 4.383053 2.201232
H -8.984495 -0.587651 -2.102760
C 0.846686 -1.581617 0.407929
H 0.560496 -1.946033 1.407874
H 1.369758 -2.390742 -0.108674
S -0.707395 -1.279587 -0.431244
C 1.720497 -0.319605 0.539589
H 1.185677 0.394152 1.177743
C 3.062685 -0.536161 1.253975
O 4.001495 0.268410 1.097298
N 1.908546 0.357411 -0.727217
N 3.148289 -1.545966 2.118430
H 2.403891 -2.227586 2.166963
C 4.359187 -1.825905 2.874322
H 4.702004 -0.924749 3.387705
H 4.127309 -2.592866 3.613174
H 5.156159 -2.189173 2.218664
C 2.641943 -0.127835 -1.732841
O 3.347583 -1.153706 -1.610047
C 2.602218 0.618406 -3.034788
H 1.897548 1.452347 -3.022375
H 3.605745 0.992298 -3.260770
H 2.319446 -0.082374 -3.826029
H 1.290413 1.151249 -0.908656
H -0.477987 2.295104 -0.061211
H 5.778840 -0.241847 0.919992
H 4.511159 1.503585 -0.176956
H 5.125104 -0.728882 -2.025404
H 3.682906 -2.697220 -0.695448
O 6.704671 -0.439029 0.667044
H 7.158828 0.412672 0.738858
O 5.005609 2.035681 -0.832490
H 5.368848 1.357902 -1.438507
O 6.022495 -0.341620 -2.061010
H 6.367622 -0.476299 -1.151957
O 3.953675 -3.593714 -0.414103
H 3.848994 -3.590496 0.546849
O -0.127197 2.436856 -0.957778
H -0.729525 1.933874 -1.525373
O 3.334166 3.919194 -1.958120
H 2.920778 3.550804 -2.750191
H 3.905604 3.196020 -1.612354
63
complexA_6sol_conf_57
Eopt -2358.108933
C 3.077549 1.546859 0.398307
C 2.748660 0.190168 0.309040
C 3.705617 -0.802319 0.153029
C 5.047987 -0.451718 0.051792
C 5.408852 0.897474 0.111798
C 4.439614 1.870595 0.294668
H 3.398030 -1.840960 0.088552
H 6.448913 1.192894 0.034994
H 4.740448 2.910581 0.376959
C 6.048027 -1.539198 -0.124304
O 5.757505 -2.724479 -0.179048
O 7.294248 -1.085910 -0.217119
C 8.330991 -2.067261 -0.390153
H 9.257465 -1.497540 -0.439356
H 8.171843 -2.620043 -1.318355
N 0.824533 -0.976709 1.081683
N 1.371331 -0.290803 0.318458
S 1.976927 2.920274 0.591082
C 0.637537 2.320420 1.657157
H 1.037457 1.754087 2.501084

H 0.143845 3.219593 2.034390
H -0.083193 1.730078 1.095923
H 8.346402 -2.749964 0.461973
C -0.930085 -0.786280 -1.284901
H -1.041267 -1.390307 -2.188690
H -0.711401 -1.468771 -0.446704
S 0.547469 0.210613 -1.506737
C -2.210897 0.010738 -0.999811
H -2.287613 0.825616 -1.726543
C -3.530651 -0.757485 -1.172032
O -4.598508 -0.113828 -1.073340
N -2.198466 0.650161 0.305674
N -3.507759 -2.049189 -1.475737
H -2.642019 -2.561192 -1.342258
C -4.720058 -2.846907 -1.570470
H -5.443961 -2.353742 -2.221979
H -4.457211 -3.815363 -1.996698
H -5.168107 -2.997636 -0.583375
C -2.344704 -0.007461 1.461884
O -2.490483 -1.247796 1.510229
C -2.318908 0.808469 2.721944
H -1.416377 0.544466 3.283670
H -2.319984 1.884061 2.534206
H -3.186331 0.540402 3.332066
H -2.184474 1.675155 0.321705
H -0.138865 2.515074 -1.733920
H -6.046034 -0.645626 -0.154612
H -4.705114 1.681302 -1.063713
H -4.155864 -1.947123 1.966785
H -2.081162 -2.891410 0.790017
O -6.767799 -0.917128 0.452636
H -6.985049 -0.115902 0.949584
O -4.823738 2.656049 -1.115753
H -5.502229 2.857910 -0.456454
O -5.033172 -2.309840 2.192997
H -5.653637 -1.835269 1.600178
O -1.863398 -3.763289 0.406270
H -2.700543 -4.249031 0.424035
O -0.528135 3.370222 -1.997679
H 0.158400 4.024193 -1.801770
O -2.432284 3.515679 0.034265
H -3.286373 3.300718 -0.402971
H -1.774881 3.541342 -0.694104
63
complexA_6sol_conf_59
Eopt -2358.109629
C 2.793036 1.782299 0.214560
C 2.533843 0.482736 0.682977
C 3.340750 -0.598615 0.380871
C 4.455819 -0.414315 -0.430121
C 4.723428 0.859108 -0.940735
C 3.908254 1.935312 -0.621813
H 3.119322 -1.578948 0.786864
H 5.581224 1.023021 -1.583230
H 4.155313 2.910007 -1.025094
C 5.313425 -1.593741 -0.716641
O 5.123053 -2.701203 -0.236119
O 6.310822 -1.320589 -1.552121
C 7.191559 -2.406606 -1.886888
H 7.682052 -2.782562 -0.986573
H 7.923961 -1.979603 -2.570074
N 0.949370 0.844431 2.410670
N 1.366443 0.243341 1.506684
S 1.749771 3.125402 0.678900
C 2.115487 4.370102 -0.585905
H 3.107145 4.806792 -0.450081
H 2.010739 3.954172 -1.590332
H 1.364789 5.151397 -0.442734
H 6.631581 -3.205539 -2.377292
C -1.042953 -1.200611 1.814851
H -1.294763 -2.179042 2.232303
H -0.693691 -0.569248 2.653107
S 0.341473 -1.385109 0.696723
C -2.319432 -0.575456 1.216895
H -3.085550 -0.629504 1.993283
C -2.126168 0.893208 0.844307
O -1.729693 1.212531 -0.295043
N -2.792363 -1.282018 0.042108
N -2.395658 1.785758 1.790079
H -2.709393 1.454628 2.693810
C -2.214403 3.213599 1.588466
H -1.176846 3.434658 1.325252
H -2.869765 3.573258 0.790513
H -2.466330 3.723787 2.517826
C -3.731804 -2.237844 0.012168
O -4.080107 -2.742073 -1.079909
C -4.366296 -2.675767 1.301126
H -4.986197 -3.551893 1.108924
H -3.615084 -2.917352 2.058716
H -5.000762 -1.873202 1.694661
H -2.401168 -0.994473 -0.850430
H -3.132159 1.299380 -1.548294
H -1.329816 2.810867 -1.156737
H -6.012342 -2.262497 -1.273304
H -3.996961 -1.761345 -2.572366
H 1.664500 -3.118271 1.852252
O 2.249280 -3.786469 2.253043
H 3.055302 -3.746400 1.718210
O -3.958207 1.288535 -2.066963
H -3.941139 0.430071 -2.548216
O -1.177168 3.556295 -1.771080
H -1.941929 3.532450 -2.362443
O -6.807326 -1.703637 -1.222364
H -6.473332 -0.878198 -0.808006
O -4.030585 -1.175936 -3.362113
H -4.955414 -1.207717 -3.645934
O -5.740375 0.622547 -0.051489
H -5.210277 0.277521 0.681145
H -5.082266 0.868069 -0.743928
63

complexA_6sol_conf_6 Eopt
-2358.115442

C	-1.468826	2.314227	-0.203923
C	-1.613618	1.141143	-0.976576
C	-2.681764	0.274313	-0.814541
C	-3.641155	0.532047	0.156573
C	-3.506263	1.668708	0.958746
C	-2.447445	2.540786	0.778147
H	-2.776387	-0.596365	-1.448866
H	-4.239864	1.876325	1.730543
H	-2.387702	3.414718	1.414147
C	-4.796289	-0.374845	0.373680
O	-5.702757	-0.142680	1.156628
O	-4.745800	-1.479624	-0.373461
C	-5.841676	-2.404277	-0.238032
H	-5.618768	-3.211658	-0.933499
H	-6.778235	-1.912720	-0.507589
N	0.239281	1.490664	-2.402825
N	-0.587343	0.818310	-1.943472
S	-0.122255	3.419742	-0.455079
C	-0.217901	4.500218	0.995664
H	-0.204313	3.927615	1.926188
H	0.685467	5.113744	0.944523
H	-1.091252	5.155085	0.958701
H	-5.886384	-2.780778	0.785658
C	1.209171	-1.367974	-2.015929
H	1.631053	-2.142826	-2.663215
H	1.676065	-0.414529	-2.300469
S	-0.545586	-1.269609	-2.417788
C	1.455261	-1.715564	-0.543940
H	0.914459	-2.643832	-0.328454
C	2.903389	-2.043954	-0.153930
O	3.138871	-2.407621	1.019066
N	0.911809	-0.731705	0.381472
N	3.857228	-2.004310	-1.076235
H	3.657809	-1.596700	-1.979970
C	5.243571	-2.310631	-0.760325
H	5.642975	-1.598973	-0.031634
H	5.325559	-3.321192	-0.353153
H	5.823380	-2.247308	-1.680795
C	1.572437	0.348614	0.810788
O	2.656762	0.706750	0.298258
C	0.994844	1.065499	1.996941
H	-0.096623	1.018544	2.020005
H	1.377499	0.575581	2.900642
H	1.328654	2.103731	2.001816
H	0.066911	-0.993086	0.895649
H	-1.675267	-2.634732	-0.716451
H	4.087195	-1.114405	2.047923
H	1.886000	-2.474352	2.328890
H	3.323897	2.355227	0.684339
H	3.222648	1.122222	-1.418027
O	4.439511	-0.299239	2.452994
H	4.057731	0.392696	1.888887
O	1.295486	-2.544454	3.111461
H	1.454491	-1.723333	3.599039
O	3.712087	3.246060	0.815577
H	3.985040	3.517338	-0.071640
O	3.614881	1.474382	-2.240646
H	2.865165	1.552103	-2.847257
O	-2.248103	-3.089116	-0.070054
H	-3.096300	-2.617804	-0.139415
O	-1.212672	-1.719777	2.134322
H	-0.420221	-2.170519	2.496359
H	-1.570589	-2.309962	1.437195

63
complexA_6sol_conf_60
Eopt -2358.098944

C	2.711637	0.229500	0.830907
C	2.655490	-0.240847	-0.485451
C	2.310571	-1.544833	-0.802881
C	1.961948	-2.424768	0.217792
C	2.007360	-1.990574	1.543409
C	2.402599	-0.694430	1.839173
H	2.277441	-1.856205	-1.842157
H	1.746440	-2.664128	2.352056
H	2.463792	-0.380588	2.876496
C	1.516735	-3.792371	-0.158607
O	1.768470	-4.317683	-1.231917
O	0.797746	-4.376161	0.794926
C	0.290601	-5.690309	0.507848
H	1.116090	-6.380978	0.323617
H	-0.374385	-5.650471	-0.357823
N	3.906743	0.680072	-2.286361
N	2.953924	0.622570	-1.620625
S	3.047129	1.879969	1.375475
C	4.363859	2.521915	0.300938
H	4.805211	3.349893	0.862222
H	5.132743	1.765453	0.131395
H	3.981890	2.910471	-0.643880
H	-0.261536	-5.984380	1.398981
C	-0.143632	0.816166	-1.825027
H	-0.596751	0.706940	-2.813233
H	0.166621	-0.177013	-1.492258
S	1.347334	1.838659	-2.055811
C	-1.156938	1.436425	-0.856378
H	-1.234523	2.504728	-1.077402
C	-2.605874	0.937085	-0.999355
O	-3.504256	1.612159	-0.446999
N	-0.771136	1.356788	0.543963
N	-2.869536	-0.156823	-1.700391
H	-2.105795	-0.780956	-1.952028
C	-4.212654	-0.709045	-1.780913
H	-4.636098	-0.854641	-0.782627
H	-4.866827	-0.045413	-2.352044
H	-4.151162	-1.671443	-2.289629
C	-0.793417	0.223442	1.258681
O	-1.003494	-0.889145	0.733343
C	-0.624563	0.361632	2.745615
H	0.027345	1.196810	3.015002

H -1.614253 0.546272 3.180298
H -0.232496 -0.567918 3.160511
H -0.634348 2.239169 1.045017
H 1.422957 3.743929 -0.507627
H -4.182516 0.795981 1.086159
H -5.329763 1.752751 -0.710551
H -2.454719 -1.871352 1.351163
H -0.948738 -2.039186 -0.713938
O -4.587090 0.360596 1.862823
H -5.500715 0.194299 1.589480
O -6.290864 1.936245 -0.715291
H -6.543538 1.894669 0.217356
O -3.340930 -2.161000 1.640180
H -3.825589 -1.313630 1.735531
O -1.086640 -2.524038 -1.550543
H -1.775940 -3.169555 -1.341408
O 1.385345 4.614698 -0.069102
H 0.873458 5.168638 -0.676254
O -0.398919 3.893717 1.929433
H 0.117018 3.586807 2.688271
H 0.270025 4.205729 1.279265
63
complexA_6sol_conf_7 Eopt
-2358.112754
C 3.317647 1.707239 -0.470049
C 2.806352 0.468400 -0.899421
C 3.500139 -0.720404 -0.760391
C 4.759042 -0.711026 -0.169262
C 5.284796 0.499143 0.293644
C 4.577511 1.682296 0.145676
H 3.072852 -1.649790 -1.122435
H 6.259382 0.526857 0.767647
H 5.021681 2.600321 0.510854
C 5.491561 -1.999130 -0.052314
O 5.067123 -3.061457 -0.481490
O 6.660223 -1.876777 0.569847
C 7.438698 -3.075217 0.727363
H 6.883771 -3.808224 1.316561
H 8.338420 -2.763819 1.255581
N 0.930991 1.154772 -2.178197
N 1.481309 0.416569 -1.471839
S 2.394006 3.191329 -0.692936
C 3.196526 4.332728 0.463121
H 3.239556 3.910081 1.469654
H 4.191993 4.623985 0.121692
H 2.557099 5.219295 0.476333
H 7.693274 -3.487729 -0.251165
C -1.120279 -0.623708 -1.204825
H -0.849040 0.072830 -2.022870
H -1.682237 -1.449942 -1.647542
S 0.447135 -1.225573 -0.594168
C -1.938288 0.134374 -0.154011
H -1.304662 0.921905 0.268927
C -3.170736 0.882854 -0.683732
O -3.893614 1.502614 0.125314
N -2.333570 -0.702680 0.967190
N -3.410803 0.911005 -1.989733
H -2.891091 0.294664 -2.600327
C -4.612266 1.528735 -2.529152
H -4.636051 2.593683 -2.286235
H -4.598853 1.409524 -3.612362
H -5.508762 1.050860 -2.121609
C -3.323059 -1.596878 0.891756
O -3.920125 -1.826545 -0.184449
C -3.719910 -2.297180 2.158925
H -4.666190 -1.867688 2.506547
H -3.886110 -3.356310 1.947033
H -2.973900 -2.190225 2.949214
H -1.943262 -0.472746 1.886352
H 0.948617 0.064581 1.380629
H -5.418020 0.710381 0.881128
H -3.289395 2.043881 1.735583
H -5.660139 -2.208712 0.023274
H -4.054693 -1.913219 -2.026779
O -6.239118 0.415244 1.317853
H -6.405010 -0.479851 0.957221
O -2.969900 2.424233 2.583441
H -3.723451 2.350077 3.185559
O -6.621279 -2.197943 0.227150
H -6.705379 -2.751767 1.016176
O -4.232706 -2.080511 -2.971946
H -4.844915 -1.377429 -3.229929
O 1.190653 0.622889 2.144214
H 1.850890 0.100236 2.621550
O -1.275674 0.378382 3.439324
H -1.812782 1.164793 3.196174
H -0.388408 0.535376 3.051233
63
complexA_6sol_conf_9 Eopt
-2358.116576
C -1.894061 2.257946 -0.018605
C -1.820570 1.169367 -0.916082
C -2.735738 0.131255 -0.889679
C -3.752653 0.122849 0.055354
C -3.838334 1.175205 0.971690
C -2.929082 2.218165 0.930431
H -2.671875 -0.674941 -1.607680
H -4.616927 1.184522 1.726418
H -3.033277 3.017217 1.653261
C -4.693747 -1.024317 0.068144
O -4.519065 -2.055997 -0.569825
O -5.749002 -0.824380 0.844567
C -6.699343 -1.900475 0.942103
H -7.465727 -1.541207 1.626743
H -6.213541 -2.792060 1.343463
N 0.030574 1.942009 -2.170324
N -0.725623 1.120463 -1.859978
S -0.737931 3.583426 -0.082620
C -1.043558 4.452140 1.477141
H -0.247232 5.198642 1.539765

H -2.007280 4.966242 1.479200
H -0.962411 3.776478 2.332204
H -7.129591 -2.108426 -0.039528
C 1.334562 -0.791632 -2.222245
H 1.858551 -1.409883 -2.957389
H 1.678361 0.244313 -2.361701
S -0.412681 -0.861167 -2.656097
C 1.609753 -1.299969 -0.802077
H 1.208399 -2.316693 -0.725360
C 3.086488 -1.448738 -0.406600
O 3.350593 -1.865674 0.741244
N 0.924171 -0.530847 0.225582
N 4.033472 -1.198787 -1.304113
H 3.776588 -0.724191 -2.159992
C 5.447598 -1.214639 -0.964211
H 6.019858 -1.097613 -1.884507
H 5.691229 -0.394977 -0.280455
H 5.713356 -2.164754 -0.496198
C 1.422225 0.589304 0.762670
O 2.424969 1.159481 0.282247
C 0.760794 1.087409 2.016102
H 0.965763 2.150759 2.146396
H -0.317611 0.907690 2.012872
H 1.193851 0.541722 2.863063
H 0.143254 -0.984100 0.709337
H -1.379805 -2.626774 -1.258153
H 4.726396 -1.434849 1.805578
H 2.103131 -2.697007 1.766791
H 3.834858 1.487604 1.437483
H 3.346006 1.616689 -1.241872
O 5.377344 -1.104549 2.461841
H 6.229411 -1.161179 2.006873
O 1.493247 -3.200937 2.349154
H 1.505187 -4.100866 1.994819
O 4.606882 1.584741 2.028313
H 4.902618 0.664563 2.191866
O 3.880951 1.998662 -1.965365
H 4.766903 1.635334 -1.828399
O -1.894404 -3.229900 -0.689376
H -2.796995 -2.856118 -0.702245
O -1.004276 -2.108610 1.713992
H -0.185394 -2.583621 1.976972
H -1.328333 -2.574457 0.912525
45
complexA_conf_1 Eopt -1899.578150
C 2.056112 1.638508 0.374760
C 1.517936 0.410125 0.795914
C 2.186323 -0.792073 0.642946
C 3.438472 -0.804213 0.035935
C 3.986070 0.396891 -0.425806
C 3.307281 1.594086 -0.256491
H 1.742531 -1.714715 1.001886
H 4.956090 0.406527 -0.909764
H 3.768200 2.508711 -0.610864
C 4.141145 -2.107379 -0.099808
O 3.700774 -3.163059 0.330016
O 5.302013 -2.007425 -0.740571
C 6.050654 -3.221990 -0.917295
H 5.470955 -3.937847 -1.503762
H 6.309505 -3.647455 0.054531
N -0.286269 1.124772 2.145117
N 0.193581 0.389040 1.377905
S 1.181164 3.148070 0.640614
C 1.786717 4.187680 -0.716575
H 1.114251 5.049285 -0.741901
H 2.804299 4.542063 -0.540239
H 1.723768 3.658096 -1.669907
H 6.949770 -2.927369 -1.456117
C -2.438593 -0.574913 1.168992
H -2.866203 -1.364903 1.790555
H -2.171227 0.254436 1.854939
S -0.891293 -1.114049 0.452451
C -3.507547 -0.095839 0.167985
H -4.289369 0.402873 0.751106
C -2.958296 0.901228 -0.857181
O -2.884961 0.610095 -2.062370
N -4.110702 -1.190509 -0.562582
N -2.577563 2.088824 -0.381580
H -2.628370 2.259238 0.614714
C -1.979615 3.101202 -1.235962
H -1.055596 2.732780 -1.691310
H -2.674513 3.389981 -2.029806
H -1.753702 3.975587 -0.626446
C -5.066265 -1.983919 -0.049959
O -5.488681 -1.843059 1.113187
C -5.605872 -3.060840 -0.951837
H -6.687543 -2.927247 -1.049600
H -5.151142 -3.054923 -1.945264
H -5.428807 -4.032976 -0.481465
H -3.824458 -1.305116 -1.528135
45
complexA_conf_10 Eopt -1899.570283
C -2.175819 2.061750 -0.076871
C -1.364243 1.022423 0.386874
C -1.819683 -0.280730 0.514518
C -3.117880 -0.594853 0.124588
C -3.944736 0.414649 -0.377387
C -3.482230 1.719498 -0.457566
H -1.152000 -1.047918 0.893145
H -4.959533 0.192261 -0.686834
H -4.150371 2.500128 -0.808144
C -3.565371 -2.007711 0.252648
O -2.861442 -2.904209 0.692004
O -4.814362 -2.196127 -0.162638
C -5.332118 -3.534343 -0.071971
H -4.734648 -4.209926 -0.687737
H -6.350617 -3.472645 -0.451857
N 0.578931 1.065475 1.743203

N 0.039907 1.216135 0.719822
S -1.719897 3.763288 -0.267981
C -0.652141 4.128907 1.156138
H 0.385095 3.843987 0.973032
H -0.703404 5.213321 1.285398
H -1.035465 3.649870 2.059551
H -5.331480 -3.866540 0.968212
C 2.622768 1.063215 -0.364618
H 3.348500 1.878779 -0.426252
H 2.444685 0.876539 0.709910
S 1.047085 1.595826 -1.022033
C 3.235375 -0.171265 -1.065639
H 3.523196 0.127694 -2.073721
C 4.560964 -0.514516 -0.371328
O 5.626616 -0.067149 -0.834661
N 2.329874 -1.297717 -1.256232
N 4.507789 -1.253715 0.737392
H 3.588520 -1.516968 1.087476
C 5.697616 -1.589810 1.497958
H 5.398578 -2.204568 2.347082
H 6.405001 -2.153021 0.882552
H 6.194721 -0.687519 1.867434
C 1.535156 -1.876697 -0.332022
O 1.671276 -1.668970 0.886374
C 0.471980 -2.796907 -0.869647
H 0.142450 -3.475947 -0.081971
H -0.383798 -2.195828 -1.197991
H 0.831520 -3.370219 -1.728879
H 2.114001 -1.508573 -2.222134
45
complexA_conf_11 Eopt
-1899.575194
C -1.559750 1.489006 -0.490419
C -1.445305 0.423722 0.413544
C -2.487532 -0.430899 0.712874
C -3.724723 -0.228791 0.100715
C -3.872059 0.822006 -0.808228
C -2.809115 1.667118 -1.098839
H -2.339532 -1.244970 1.415229
H -4.824298 0.991492 -1.298150
H -2.963837 2.472689 -1.806614
C -4.837945 -1.151368 0.441351
O -4.738430 -2.067932 1.243998
O -5.961251 -0.878659 -0.217212
C -7.092587 -1.722050 0.056511
H -6.862628 -2.756200 -0.208313
H -7.894221 -1.337248 -0.571921
N 0.316186 0.680270 1.991664
N -0.163737 0.163626 1.059115
S -0.158894 2.516487 -0.785868
C -0.704920 3.571583 -2.150467
H -1.517481 4.235019 -1.847114
H 0.167236 4.177000 -2.410252
H -0.995165 2.976639 -3.019480
H -7.366614 -1.651412 1.111231
C 2.169327 -1.404948 1.053501
H 1.908408 -0.859792 1.977523
H 2.366570 -2.442598 1.335885
S 0.700728 -1.341819 0.029525
C 3.437453 -0.754287 0.463327
H 4.252157 -0.956491 1.164531
C 3.813606 -1.376274 -0.884812
O 3.689735 -0.749616 -1.948769
N 3.308091 0.675799 0.290401
N 4.253474 -2.636686 -0.834900
H 4.339171 -3.088579 0.066574
C 4.581558 -3.391322 -2.032119
H 5.400802 -2.915383 -2.578532
H 4.888906 -4.392686 -1.731171
H 3.712980 -3.466828 -2.692624
C 3.547481 1.622976 1.219934
O 3.324603 2.821606 0.966198
C 4.121682 1.197626 2.545183
H 5.111606 0.749743 2.404344
H 4.216598 2.075231 3.185664
H 3.485190 0.458735 3.041179
H 2.906420 0.992125 -0.586507
45
complexA_conf_12 Eopt
-1899.578166
C 1.764406 1.565807 -0.554064
C 1.542587 0.193952 -0.760922
C 2.506755 -0.767975 -0.518420
C 3.754885 -0.376201 -0.042352
C 3.998141 0.978559 0.201960
C 3.020460 1.930051 -0.049192
H 2.296229 -1.815140 -0.709909
H 4.960570 1.302107 0.581861
H 3.248984 2.972201 0.140309
C 4.780233 -1.426861 0.190150
O 4.591311 -2.616258 -0.017085
O 5.929789 -0.938858 0.647099
C 6.981978 -1.886410 0.896409
H 7.821879 -1.292889 1.253919
H 7.245921 -2.404976 -0.027686
N -0.422122 0.170281 -2.083480
N 0.243203 -0.249789 -1.223719
S 0.504104 2.744327 -0.926883
C 0.953777 4.143708 0.134717
H 0.093075 4.816923 0.102321
H 1.112573 3.817870 1.165396
H 1.830526 4.675821 -0.239869
H 6.668053 -2.602800 1.658389
C -2.049103 -1.769429 -0.636360
H -1.993301 -1.196608 -1.583923
H -2.321836 -2.796527 -0.893614
S -0.400198 -1.750334 0.047197
C -3.141720 -1.166703 0.262912
H -3.152127 -1.688436 1.224318
C -2.913347 0.334209 0.501671

O -3.189198 1.170999 -0.374236
N -4.419097 -1.381965 -0.390777
N -2.397058 0.673610 1.684842
H -2.189861 -0.057072 2.352108
C -2.093066 2.049509 2.035835
H -1.364658 2.475801 1.339534
H -2.999266 2.662147 2.017264
H -1.674805 2.062080 3.042227
C -5.590729 -1.082359 0.201349
O -5.634605 -0.657445 1.369342
C -6.839121 -1.283260 -0.612366
H -7.315791 -0.309403 -0.763299
H -6.645848 -1.740733 -1.585478
H -7.532042 -1.912868 -0.046818
H -4.420432 -1.643420 -1.369068
45
complexA_conf_13 Eopt
-1899.574358
C -1.732282 1.516319 -0.324893
C -1.559908 0.154536 -0.611908
C -2.578796 -0.772914 -0.513313
C -3.846366 -0.344559 -0.119570
C -4.048977 1.004575 0.183678
C -3.009594 1.919851 0.083014
H -2.392286 -1.817433 -0.741777
H -5.026345 1.353142 0.497781
H -3.203190 2.959231 0.321589
C -4.933506 -1.353699 -0.030845
O -4.783591 -2.537524 -0.295284
O -6.092205 -0.836899 0.369020
C -7.200621 -1.744767 0.485403
H -8.037969 -1.132224 0.815900
H -6.976870 -2.516849 1.224531
N 0.411075 -0.055902 -1.919357
N -0.236815 -0.322183 -0.983337
S -0.361392 2.614142 -0.486995
C -0.850833 4.022586 0.540372
H 0.041719 4.649756 0.611817
H -1.653021 4.603349 0.080559
H -1.137985 3.694784 1.542248
H -7.417882 -2.197998 -0.484092
C 2.074820 -1.646935 -0.069934
H 2.310970 -2.693778 -0.280204
H 2.102213 -1.106205 -1.033020
S 0.387569 -1.521197 0.516294
C 3.124450 -1.094490 0.915682
H 2.975649 -1.585077 1.877982
C 4.519542 -1.567074 0.473614
O 5.018539 -2.568721 1.019806
N 3.010080 0.331253 1.204096
N 5.123168 -0.910623 -0.516997
H 4.631658 -0.122140 -0.933593
C 6.423307 -1.309234 -1.025017
H 7.179463 -1.267582 -0.235553
H 6.390410 -2.326624 -1.426307
H 6.703927 -0.620934 -1.822362
C 3.060343 1.368173 0.344086
O 3.288890 1.223264 -0.870752
C 2.897490 2.733112 0.959814
H 3.860433 3.037911 1.386189
H 2.621109 3.450561 0.185836
H 2.150681 2.740620 1.758410
H 2.818465 0.558703 2.171040
45
complexA_conf_14 Eopt
-1899.574430
C -1.742897 1.667673 -0.305627
C -1.626380 0.293864 -0.561408
C -2.674594 -0.595698 -0.419107
C -3.916535 -0.110664 -0.007850
C -4.061819 1.251186 0.266706
C -2.995216 2.127196 0.121165
H -2.522074 -1.648944 -0.627936
H -5.022665 1.634330 0.593915
H -3.146622 3.178181 0.338727
C -5.088655 -1.009090 0.154942
O -6.186694 -0.629274 0.533701
O -4.822106 -2.275581 -0.153543
C -5.900895 -3.215903 -0.018176
H -5.482357 -4.175485 -0.317470
H -6.726423 -2.937086 -0.676382
N 0.314732 -0.008859 -1.894876
N -0.329837 -0.239703 -0.947519
S -0.338079 2.711001 -0.526962
C -0.752148 4.159577 0.477184
H -1.544647 4.757473 0.022620
H -1.025263 3.865920 1.493421
H 0.163102 4.756348 0.511504
H -6.237750 -3.253151 1.019905
C 1.941475 -1.645324 -0.056392
H 2.132030 -2.701480 -0.266019
H 1.974457 -1.109575 -1.022271
S 0.270878 -1.448378 0.557123
C 3.029199 -1.131685 0.908799
H 2.881143 -1.617329 1.873674
C 4.396984 -1.655308 0.440171
O 4.860404 -2.684780 0.965644
N 2.970434 0.296946 1.199112
N 5.015467 -1.009916 -0.548458
H 4.549478 -0.199374 -0.952098
C 6.290966 -1.456057 -1.078919
H 6.208459 -2.462704 -1.499848
H 6.594345 -0.764949 -1.865423
H 7.055989 -1.464373 -0.297019
C 3.037534 1.332407 0.338329
O 3.247947 1.181513 -0.879027
C 2.916290 2.700520 0.957055
H 2.678523 3.430504 0.182107
H 2.156759 2.731164 1.743126
H 3.881890 2.968282 1.401834

H 2.795012 0.529836 2.167826
45
complexA_conf_15 Eopt
-1899.576380
C -0.709872 -1.925965 0.654883
C 0.103677 -1.875028 -0.486659
C 1.439199 -1.525422 -0.455674
C 2.017452 -1.193784 0.770063
C 1.237327 -1.241764 1.927524
C -0.100553 -1.610083 1.876076
H 2.006635 -1.486954 -1.379000
H 1.677508 -0.979753 2.883912
H -0.671786 -1.630222 2.796408
C 3.430550 -0.746861 0.865853
O 3.973743 -0.432120 1.914202
O 4.046919 -0.716345 -0.312945
C 5.415828 -0.278609 -0.317034
H 5.482361 0.745731 0.055496
H 5.727406 -0.325709 -1.359275
N -0.771750 -3.159250 -2.286146
N -0.494232 -2.135547 -1.789042
S -2.412042 -2.347841 0.474738
C -3.091868 -2.028599 2.120660
H -2.690263 -2.722405 2.862512
H -2.919444 -0.993710 2.425008
H -4.167107 -2.198787 2.023590
H 6.025779 -0.946810 0.294529
C -0.055875 0.963203 -1.987430
H 1.016255 0.759069 -1.985673
H -0.225391 1.784435 -2.697118
S -0.957250 -0.429417 -2.738898
C -0.482818 1.403627 -0.578865
H -0.228315 0.629568 0.146855
C 0.380427 2.605131 -0.180685
O 1.596075 2.436170 0.021790
N -1.907327 1.638247 -0.498639
N -0.213872 3.794642 -0.089693
H -1.215012 3.844549 -0.226929
C 0.510969 4.994204 0.290669
H 0.943854 4.886036 1.289563
H 1.314486 5.201277 -0.421439
H -0.187475 5.830849 0.293204
C -2.584901 1.506337 0.664316
O -1.990939 1.356595 1.743562
C -4.085292 1.548926 0.588729
H -4.458068 2.291290 1.299930
H -4.457601 1.782310 -0.411474
H -4.470220 0.569118 0.892762
H -2.436547 1.703332 -1.359092
45
complexA_conf_16 Eopt
-1899.570696
C -0.290479 2.253359 0.595561
C -0.583990 1.832598 -0.709155
C -1.680227 1.054353 -1.023455
C -2.533629 0.645216 0.000383
C -2.268861 1.045675 1.312366
C -1.172385 1.846815 1.604572
H -1.851942 0.745152 -2.049576
H -2.919442 0.732540 2.121066
H -1.003323 2.144179 2.632815
C -3.667762 -0.249089 -0.347293
O -3.920354 -0.611389 -1.486980
O -4.379066 -0.624747 0.711634
C -5.486263 -1.507955 0.465853
H -5.129195 -2.442700 0.028606
H -6.208946 -1.028662 -0.197857
N 0.447653 3.176128 -2.376058
N 0.342666 2.168352 -1.783080
S 1.143548 3.240973 0.880050
C 1.370833 3.101820 2.670899
H 2.332896 3.577657 2.877551
H 0.591210 3.630538 3.222875
H 1.418458 2.053838 2.977092
H -5.929135 -1.689571 1.443849
C 0.928312 -0.860637 -1.471836
H -0.107383 -0.980673 -1.800429
H 1.511921 -1.639581 -1.973355
S 1.620522 0.681265 -2.138555
C 0.982673 -1.044825 0.056810
H 0.330052 -0.308444 0.521985
C 0.330037 -2.392122 0.400644
O -0.880030 -2.423521 0.688852
N 2.290484 -0.813085 0.656023
N 1.089635 -3.486761 0.337647
H 2.044223 -3.384108 0.001143
C 0.554392 -4.811967 0.593742
H 0.144763 -4.873789 1.605826
H -0.235978 -5.061080 -0.121095
H 1.365701 -5.533198 0.494658
C 3.468015 -1.325398 0.249322
O 3.536533 -2.241253 -0.591837
C 4.707449 -0.742593 0.868445
H 5.261943 -0.212410 0.086908
H 4.494557 -0.048067 1.684046
H 5.338141 -1.556611 1.235874
H 2.327917 -0.057481 1.327867
45
complexA_conf_17 Eopt
-1899.579272
C -2.796324 1.264281 0.018249
C -1.503774 0.719685 -0.092935
C -1.268017 -0.642567 -0.136187
C -2.339437 -1.527169 -0.067375
C -3.638248 -1.017764 0.012967
C -3.860195 0.351057 0.052029
H -0.254162 -1.016813 -0.213682
H -4.488893 -1.688806 0.055289
H -4.880788 0.706956 0.122237
C -2.044929 -2.983238 -0.080066

O -0.913225 -3.444830 -0.097932
O -3.140706 -3.736219 -0.067551
C -2.947263 -5.160896 -0.071778
H -3.949979 -5.585051 -0.059778
H -2.392178 -5.466099 0.817707
N -0.132975 2.565474 0.474294
N -0.357901 1.606631 -0.147661
S -3.031729 3.010523 0.103129
C -4.790368 3.191554 -0.294775
H -4.946886 4.267258 -0.409958
H -5.036830 2.693491 -1.235395
H -5.429151 2.829481 0.513464
H -2.414625 -5.463976 -0.975583
C 2.287566 1.931996 -1.063330
H 2.834850 2.357546 -1.908846
H 1.875348 2.780292 -0.490375
S 0.887917 1.003719 -1.697971
C 3.257403 1.151305 -0.159785
H 4.078624 1.828267 0.092030
C 2.578655 0.804878 1.170454
O 2.500576 1.668256 2.061094
N 3.798045 0.001085 -0.853807
N 2.044020 -0.412196 1.293471
H 2.103960 -1.047163 0.507671
C 1.200350 -0.777507 2.417799
H 0.354399 -0.087610 2.505646
H 0.823377 -1.786576 2.248955
H 1.768937 -0.759816 3.351908
C 4.872459 -0.676650 -0.396615
O 5.477155 -0.313207 0.625830
C 5.299338 -1.887169 -1.179061
H 6.344266 -1.761743 -1.478072
H 5.240275 -2.762272 -0.524417
H 4.688338 -2.062291 -2.067750
H 3.290088 -0.372674 -1.646729
45
complexA_conf_18 Eopt -1899.579540
C -3.175639 1.008755 0.007837
C -1.851148 0.568393 0.151140
C -1.476447 -0.757602 0.044371
C -2.453272 -1.718726 -0.207206
C -3.779579 -1.312819 -0.372045
C -4.135387 0.024741 -0.270027
H -0.434609 -1.030499 0.167300
H -4.547593 -2.051175 -0.577663
H -5.175713 0.297577 -0.401085
C -2.111409 -3.160811 -0.303066
O -2.927472 -4.038906 -0.540610
O -0.819440 -3.399892 -0.098716
C -0.393942 -4.771099 -0.148619
H -0.579880 -5.185809 -1.141605
H -0.917851 -5.353952 0.611832
N -0.675109 2.272463 1.316243
N -0.794623 1.525994 0.430059
S -3.552038 2.723979 0.172909
C -5.246003 2.828119 -0.457253
H -5.477305 3.896168 -0.477799
H -5.317119 2.429472 -1.471894
H -5.954896 2.327679 0.205545
H 0.674468 -4.746975 0.060438
C 1.856270 2.237131 -0.196098
H 2.283344 3.053724 -0.783622
H 1.354748 2.700108 0.673747
S 0.555508 1.423978 -1.124908
C 2.978972 1.319197 0.303879
H 3.615348 1.915989 0.964856
C 2.415159 0.193716 1.181046
O 1.840876 0.485439 2.244890
N 3.798804 0.853708 -0.797016
N 2.566883 -1.063862 0.765051
H 3.039194 -1.231668 -0.113101
C 2.058620 -2.195482 1.519650
H 2.234913 -3.100845 0.939018
H 2.573280 -2.282146 2.481550
H 0.986129 -2.089316 1.704700
C 5.102790 0.527511 -0.654316
O 5.674288 0.597040 0.445875
C 5.827791 0.073642 -1.890934
H 5.191886 0.054348 -2.778772
H 6.672676 0.746133 -2.067811
H 6.227862 -0.929052 -1.712670
H 3.377235 0.757378 -1.713004
45
complexA_conf_2 Eopt -1899.571124
C -1.471388 1.729789 -0.178979
C -1.360118 0.982231 1.002883
C -1.791242 -0.323495 1.116651
C -2.357589 -0.948008 0.005199
C -2.481651 -0.232644 -1.188178
C -2.052937 1.085628 -1.277460
H -1.686868 -0.851383 2.059410
H -2.916623 -0.701799 -2.063438
H -2.164637 1.605612 -2.221019
C -2.798642 -2.359317 0.140747
O -2.697035 -3.006103 1.173170
O -3.314802 -2.853980 -0.981145
C -3.760918 -4.219824 -0.941263
H -4.139154 -4.425858 -1.941300
H -4.555940 -4.333386 -0.201213
N -1.204967 2.428050 2.879796
N -0.762603 1.605757 2.174570
S -0.876482 3.387877 -0.203543
C -1.190186 3.914093 -1.904996
H -0.816171 4.939710 -1.959670
H -0.641202 3.296160 -2.618520
H -2.258758 3.915024 -2.131748
H -2.924307 -4.880807 -0.705353
C 1.515091 -0.546858 1.630145

H 2.143638 -1.121997 2.319480
H 0.606310 -1.135432 1.474290
S 1.129060 0.992160 2.517204
C 2.264886 -0.361619 0.306194
H 3.092000 0.330986 0.461832
C 2.812689 -1.727916 -0.123944
O 2.044581 -2.618397 -0.526120
N 1.403390 0.158265 -0.736366
N 4.132119 -1.899808 -0.016471
H 4.701246 -1.124834 0.297612
C 4.779457 -3.158961 -0.343135
H 4.386899 -3.967661 0.279709
H 4.621421 -3.414514 -1.394780
H 5.848149 -3.052345 -0.158324
C 1.730374 1.077979 -1.671469
O 0.985844 1.267158 -2.648745
C 3.000363 1.866239 -1.489170
H 3.874450 1.209332 -1.555269
H 3.061971 2.618455 -2.276456
H 3.020781 2.361061 -0.512870
H 0.552130 -0.366097 -0.918287
45
complexA_conf_20 Eopt
-1899.570019
C 2.563050 0.371705 -0.300900
C 1.747473 0.108465 0.808314
C 1.043068 -1.067671 0.968137
C 1.123690 -2.044674 -0.023621
C 1.920335 -1.810758 -1.147394
C 2.635572 -0.628088 -1.280125
H 0.419372 -1.210786 1.845368
H 1.991696 -2.555887 -1.931673
H 3.248186 -0.488170 -2.162571
C 0.333711 -3.290534 0.150892
O -0.317590 -3.545537 1.152603
O 0.402571 -4.102311 -0.901055
C -0.336195 -5.332552 -0.817239
H 0.024906 -5.930765 0.021976
H -0.146535 -5.844279 -1.759489
N 2.158503 1.271119 2.844877
N 1.582800 1.126958 1.837054
S 3.423126 1.906923 -0.387053
C 4.289805 1.801314 -1.971587
H 3.588008 1.707142 -2.803354
H 4.827384 2.748064 -2.067226
H 5.011320 0.981300 -1.979580
H -1.401907 -5.122630 -0.704156
C -0.620527 1.920247 -0.217970
H -0.660172 2.808002 -0.857411
H 0.049074 1.204958 -0.707209
S 0.104091 2.423794 1.368036
C -2.030766 1.308948 -0.108865
H -2.708454 2.035842 0.340106
C -2.498037 0.980703 -1.529758
O -2.051859 -0.016135 -2.122434
N -2.054109 0.091558 0.673271
N -3.363993 1.830473 -2.085092
H -3.692859 2.617506 -1.541474
C -3.833665 1.676046 -3.451472
H -4.369837 0.730517 -3.571765
H -4.509611 2.500691 -3.676102
H -2.994860 1.698391 -4.152962
C -2.526023 -0.074190 1.927299
O -2.359730 -1.159541 2.513144
C -3.280328 1.057230 2.573581
H -2.638266 1.934440 2.699530
H -4.140980 1.350564 1.963460
H -3.630931 0.729014 3.552799
H -1.537517 -0.695088 0.291423
45
complexA_conf_22 Eopt
-1899.571124
C 1.470858 -1.729397 -0.179034
C 1.359618 -0.982087 1.002986
C 1.790761 0.323614 1.116985
C 2.357075 0.948325 0.005615
C 2.481213 0.233151 -1.187867
C 2.052493 -1.085105 -1.277384
H 1.686421 0.851320 2.059845
H 2.916243 0.702452 -2.063019
H 2.164258 -1.604989 -2.220990
C 2.798145 2.359596 0.141436
O 2.696258 3.006282 1.173898
O 3.314664 2.854360 -0.980249
C 3.760930 4.220151 -0.940050
H 4.555718 4.333519 -0.199719
H 2.924326 4.881209 -0.704331
N 1.206296 -2.427001 2.880711
N 0.762477 -1.606100 2.174680
S 0.875995 -3.387483 -0.203776
C 1.191584 -3.914293 -1.904678
H 0.818256 -4.940164 -1.959236
H 0.642932 -3.297102 -2.619082
H 2.260367 -3.914651 -2.130444
H 4.139524 4.426258 -1.939934
C -1.516387 0.543845 1.630617
H -2.145426 1.117768 2.320505
H -0.607447 1.132593 1.476467
S -1.130690 -0.996760 2.515011
C -2.265173 0.361092 0.305749
H -3.092519 -0.331666 0.459386
C -2.812485 1.728252 -0.122234
O -2.043826 2.619854 -0.520838
N -1.403069 -0.156863 -0.737267
N -4.132177 1.899514 -0.017021
H -4.701705 1.123682 0.294213
C -4.779218 3.159133 -0.342448
H -5.848298 3.051710 -0.160384
H -4.388443 3.966627 0.283081
H -4.618803 3.417043 -1.393156

C -1.728977 -1.075951 -1.673354
O -0.984083 -1.263337 -2.650699
C -2.998226 -1.865658 -1.492146
H -3.872947 -1.209589 -1.558220
H -3.058700 -2.617385 -2.279984
H -3.018586 -2.361156 -0.516189
H -0.552113 0.368286 -0.918348
45
complexA_conf_23 Eopt
-1899.576018
C 2.468398 -0.695651 -0.140465
C 1.486846 -0.521183 -1.124580
C 0.303682 -1.232435 -1.147648
C 0.056939 -2.166398 -0.140887
C 1.017540 -2.366747 0.853297
C 2.206223 -1.649084 0.852730
H -0.427342 -1.034416 -1.924399
H 0.832413 -3.085437 1.644885
H 2.925985 -1.832407 1.641453
C -1.240646 -2.885166 -0.058753
O -1.593456 -3.537852 0.912497
O -1.988163 -2.726129 -1.144809
C -3.313333 -3.279699 -1.112215
H -3.871958 -2.864978 -0.270312
H -3.263870 -4.368039 -1.036316
N 2.323846 0.350751 -3.165538
N 1.692898 0.468154 -2.181885
S 3.932309 0.284542 -0.212361
C 4.738146 -0.077845 1.366713
H 5.074507 -1.115459 1.416942
H 5.611346 0.578435 1.400872
H 4.082711 0.158331 2.208104
H -3.770041 -2.983654 -2.055247
C 0.371119 2.191545 -0.054062
H 1.212276 1.729396 0.471159
H 0.379466 3.252509 0.222276
S 0.687293 2.149226 -1.842659
C -0.946465 1.547157 0.396206
H -0.993401 0.529535 0.012347
C -0.954833 1.503255 1.928387
O -1.243054 2.508978 2.597578
N -2.089284 2.280748 -0.101654
N -0.599931 0.339490 2.478597
H -0.367952 -0.435201 1.869635
C -0.456737 0.162635 3.912855
H 0.289588 0.853165 4.316362
H -1.409553 0.334823 4.421171
H -0.133761 -0.861724 4.098664
C -3.245033 1.752451 -0.561990
O -4.196270 2.498396 -0.856049
C -3.333076 0.258399 -0.730672
H -4.333491 0.000042 -1.079616
H -2.597607 -0.086424 -1.464789
H -3.135395 -0.260467 0.213260
H -2.095334 3.283149 0.059992
45
complexA_conf_24 Eopt
-1899.570574
C 1.303854 1.852593 0.019162
C 0.608025 1.344886 1.126403
C -0.768631 1.368024 1.225360
C -1.517197 1.873103 0.161964
C -0.855050 2.379915 -0.957871
C 0.532839 2.387429 -1.020118
H -1.257763 0.993123 2.118447
H -1.419322 2.778171 -1.793636
H 1.011332 2.802493 -1.899239
C -2.997636 1.826476 0.263424
O -3.597912 1.395383 1.237243
O -3.609137 2.297887 -0.820081
C -5.046586 2.278602 -0.811969
H -5.342943 2.697471 -1.772366
H -5.425737 2.894160 0.006474
N 1.882979 1.309535 3.129605
N 1.363982 0.765800 2.231378
S 3.064110 1.793089 0.015386
C 3.500320 2.681080 -1.498907
H 4.593013 2.685052 -1.523691
H 3.129430 2.171553 -2.390557
H 3.142443 3.712716 -1.468558
H -5.406892 1.252275 -0.715193
C -0.008942 -1.847700 1.384829
H -0.307851 -2.679549 2.031025
H -0.764456 -1.069484 1.512356
S 1.579637 -1.233741 2.014112
C -0.001805 -2.333147 -0.078001
H 0.619312 -3.225169 -0.157136
C -1.455739 -2.638839 -0.450447
O -2.193610 -1.720675 -0.852418
N 0.524672 -1.371410 -1.016283
N -1.898301 -3.888909 -0.281028
H -2.876252 -4.037834 -0.502122
C -1.116837 -5.042771 0.149399
H -0.356414 -5.307309 -0.590416
H -0.637727 -4.860585 1.114587
H -1.802180 -5.882926 0.259560
C 1.676986 -1.553388 -1.691067
O 2.405544 -2.545694 -1.512519
C 2.027481 -0.514762 -2.720881
H 3.073316 -0.223688 -2.593891
H 1.923833 -0.972174 -3.710916
H 1.388262 0.369365 -2.676280
H -0.022244 -0.536567 -1.191597
45
complexA_conf_25 Eopt
-1899.574261
C -0.997619 2.199804 -0.165516
C -0.764629 1.330811 0.909074
C -1.542295 0.217900 1.163598
C -2.611780 -0.067443 0.316613

C -2.850237 0.758934 -0.784347
C -2.058669 1.874521 -1.021279
H -1.318335 -0.417576 2.014416
H -3.668410 0.543563 -1.462310
H -2.283237 2.498774 -1.877555
C -3.460377 -1.245269 0.628810
O -3.372681 -1.889905 1.663344
O -4.328326 -1.531731 -0.337376
C -5.172284 -2.675816 -0.127507
H -4.562320 -3.576278 -0.026189
H -5.796997 -2.736993 -1.017190
N 0.253354 2.128298 2.897803
N 0.316478 1.611920 1.848146
S 0.022834 3.625995 -0.348788
C -0.609779 4.391943 -1.860900
H -0.502480 3.725739 -2.719908
H -1.648028 4.710370 -1.744386
H 0.016123 5.274204 -2.016856
H -5.787980 -2.531178 0.762714
C 1.997157 0.450547 -0.514901
H 1.013328 0.724770 -0.904630
H 2.731657 1.050512 -1.059700
S 2.096749 0.910877 1.242502
C 2.219135 -1.044135 -0.813297
H 2.310493 -1.126771 -1.902812
C 3.509791 -1.575520 -0.184873
O 3.486700 -2.425797 0.719671
N 1.127056 -1.881237 -0.378471
N 4.644608 -1.057404 -0.660823
H 4.598174 -0.386043 -1.416653
C 5.946143 -1.429491 -0.132927
H 6.706415 -0.860753 -0.667834
H 6.011035 -1.198388 0.934074
H 6.132213 -2.497884 -0.275620
C -0.014773 -2.015955 -1.078265
O -0.242376 -1.345189 -2.100374
C -0.971226 -3.064508 -0.576647
H -1.967802 -2.878504 -0.980720
H -0.632813 -4.044275 -0.933252
H -1.007027 -3.094874 0.515627
H 1.294302 -2.476385 0.424831
45
complexA_conf_26 Eopt -1899.570197
C -2.745693 -0.703660 0.276030
C -1.911272 -0.445521 -0.820135
C -1.395034 0.802829 -1.103270
C -1.705417 1.868322 -0.258921
C -2.533717 1.645192 0.842158
C -3.051652 0.384063 1.104562
H -0.745213 0.931941 -1.962241
H -2.772815 2.465595 1.510695
H -3.689784 0.252736 1.970141
C -1.128320 3.219793 -0.471198
O -1.380192 4.184797 0.235168
O -0.294648 3.271714 -1.506689
C 0.337342 4.535870 -1.766422
H 0.967539 4.366417 -2.638033
H 0.943908 4.833257 -0.908338
N -2.095265 -2.031379 -2.584413
N -1.513431 -1.546477 -1.690512
S -3.334734 -2.345068 0.534768
C -4.236265 -2.212483 2.097763
H -5.100363 -1.550837 2.006986
H -4.587552 -3.224787 2.312858
H -3.581388 -1.884432 2.908247
H -0.416281 5.296272 -1.981740
C 0.866094 -1.329975 0.261625
H 0.003131 -0.979804 0.837301
H 1.393845 -2.046831 0.896064
S 0.287507 -2.269133 -1.182166
C 1.763984 -0.127871 -0.075194
H 1.218759 0.539369 -0.742408
C 1.997536 0.707029 1.193691
O 1.320695 1.733994 1.381749
N 2.975511 -0.483612 -0.808384
N 2.901525 0.266136 2.069337
H 3.378741 -0.608513 1.859576
C 3.172931 0.962534 3.313682
H 3.505417 1.986506 3.120389
H 2.280222 0.996701 3.945706
H 3.962282 0.426340 3.840596
C 3.879225 -1.427322 -0.479244
O 3.864130 -2.010548 0.621317
C 4.924414 -1.748359 -1.510907
H 4.903833 -1.073853 -2.369619
H 5.910435 -1.710445 -1.040070
H 4.756558 -2.772699 -1.860020
H 3.061166 -0.070689 -1.727590
45
complexA_conf_3 Eopt -1899.575216
C -1.297666 1.638869 -0.232874
C -1.296588 0.363142 -0.816036
C -2.408986 -0.458112 -0.835498
C -3.592761 -0.009878 -0.255492
C -3.622601 1.248342 0.354022
C -2.495219 2.056847 0.363381
H -2.358634 -1.437373 -1.301459
H -4.533252 1.609977 0.817924
H -2.554795 3.031792 0.833471
C -4.781369 -0.902129 -0.302793
O -4.784998 -1.997685 -0.844101
O -5.843931 -0.385546 0.307074
C -7.039699 -1.183878 0.310613
H -7.370568 -1.367260 -0.713769
H -7.777245 -0.590940 0.849102
N 0.660284 0.336396 -2.152822
N -0.062450 -0.152035 -1.377510
S 0.156103 2.645346 -0.260887

C -0.024689 3.599676 1.272267
H 0.949173 4.065599 1.443456
H -0.778537 4.384049 1.180459
H -0.258476 2.940578 2.112152
H -6.857732 -2.128744 0.826936
C 2.129605 -1.732956 -0.744362
H 2.573726 -2.732022 -0.784877
H 2.222258 -1.286684 -1.746974
S 0.378655 -1.894489 -0.391759
C 2.882762 -0.836159 0.253649
H 2.445829 0.162216 0.237231
C 2.847367 -1.432451 1.659368
O 3.452307 -2.498515 1.880864
N 4.275086 -0.705973 -0.136154
N 2.175012 -0.786092 2.616924
H 2.171488 -1.243106 3.521847
C 1.412380 0.450642 2.486817
H 1.025793 0.702220 3.474431
H 2.044156 1.272984 2.142156
H 0.567664 0.324750 1.803906
C 4.722377 0.275945 -0.945521
O 3.986248 1.198309 -1.335980
C 6.171255 0.210713 -1.345978
H 6.228546 0.154109 -2.437358
H 6.663950 1.135679 -1.031482
H 6.698887 -0.642916 -0.914566
H 4.892100 -1.478877 0.084430
45
complexA_conf_4 Eopt -1899.577586
C 2.056106 1.767454 0.204977
C 1.698180 0.498219 0.691070
C 2.468071 -0.633692 0.482186
C 3.648759 -0.524513 -0.247350
C 4.016918 0.718474 -0.769984
C 3.236206 1.841956 -0.548666
H 2.154010 -1.586006 0.894328
H 4.930569 0.811552 -1.347415
H 3.559561 2.792081 -0.957594
C 4.525682 -1.700321 -0.489217
O 5.572767 -1.646744 -1.116512
O 4.052324 -2.821457 0.047477
C 4.836018 -4.011641 -0.141467
H 4.928177 -4.234505 -1.206536
H 4.284032 -4.800706 0.366621
N -0.020407 1.039500 2.223300
N 0.452776 0.352364 1.410927
S 1.053460 3.180330 0.541620
C 1.393107 4.241976 -0.889678
H 0.626901 5.021343 -0.859995
H 2.376254 4.712859 -0.827133
H 1.294477 3.680197 -1.821340
H 5.823209 -3.885423 0.307984
C -2.072373 -0.876201 1.497607
H -2.344697 -1.690599 2.173981
H -1.795239 -0.015984 2.141643
S -0.576951 -1.270801 0.599060
C -3.300030 -0.487757 0.660023
H -4.011046 -0.010827 1.342613
C -2.968425 0.526630 -0.450736
O -2.982941 0.215889 -1.651027
N -3.939103 -1.662651 0.110157
N -2.678769 1.763732 -0.033654
H -2.644653 1.950875 0.960442
C -2.286441 2.815521 -0.956121
H -1.379862 2.539855 -1.503259
H -3.087186 3.010704 -1.675544
H -2.095422 3.722661 -0.383295
C -5.195894 -1.630614 -0.376164
O -5.902637 -0.611251 -0.287293
C -5.708611 -2.888679 -1.020649
H -5.918463 -2.678303 -2.074169
H -5.002553 -3.719701 -0.956536
H -6.649237 -3.173892 -0.540590
H -3.374201 -2.481623 -0.076338
45
complexA_conf_5 Eopt -1899.575225
C -2.432086 1.770472 0.162194
C -1.380416 0.859890 -0.030550
C -1.579723 -0.503202 -0.147536
C -2.872376 -1.014091 -0.066654
C -3.943474 -0.132197 0.102469
C -3.726813 1.234286 0.212215
H -0.732757 -1.165024 -0.292449
H -4.959090 -0.507909 0.157130
H -4.582241 1.885200 0.347740
C -3.050004 -2.485164 -0.166845
O -2.121567 -3.274472 -0.263540
O -4.325998 -2.859676 -0.139505
C -4.588325 -4.270255 -0.228052
H -4.209073 -4.663426 -1.173587
H -5.672376 -4.362524 -0.184555
N 0.561745 2.024912 0.662404
N -0.010697 1.343314 -0.096658
S -2.090798 3.494610 0.321490
C -3.722488 4.246805 0.093315
H -4.178565 3.929027 -0.847176
H -4.385712 4.036568 0.934825
H -3.537436 5.323371 0.052892
H -4.126376 -4.791111 0.613223
C 2.500572 0.777414 -1.224301
H 3.136897 1.253957 -1.975354
H 2.488178 1.441941 -0.343828
S 0.817062 0.710723 -1.838620
C 3.072868 -0.614007 -0.880565
H 3.000111 -1.233498 -1.774778
C 4.583927 -0.502903 -0.631395
O 5.359020 -0.679587 -1.590713
N 2.297813 -1.332287 0.129789

N 5.007797 -0.178940 0.589117
H 4.302350 0.016453 1.298147
C 6.416345 -0.007794 0.895417
H 6.850160 0.805982 0.306112
H 6.508927 0.231104 1.954999
H 6.972623 -0.926282 0.687562
C 1.985182 -0.924205 1.376666
O 2.541150 0.046120 1.923144
C 0.928333 -1.710989 2.101942
H 1.338157 -2.056612 3.055439
H 0.093075 -1.037104 2.320087
H 0.555179 -2.565977 1.534078
H 1.751150 -2.101920 -0.234000
45
complexA_conf_6 Eopt -1899.571162
C -1.968955 1.219667 -0.163265
C -1.673716 0.343278 0.891424
C -1.670748 -1.030868 0.759782
C -1.967586 -1.588515 -0.484843
C -2.268322 -0.743456 -1.554601
C -2.275818 0.635947 -1.398231
H -1.441134 -1.653484 1.617800
H -2.497633 -1.167119 -2.526814
H -2.510416 1.256637 -2.254399
C -1.960293 -3.057207 -0.701313
O -2.248144 -3.588945 -1.763404
O -1.599672 -3.741452 0.382006
C -1.557955 -5.173001 0.259972
H -1.244152 -5.533947 1.238142
H -0.834068 -5.463198 -0.504363
N -2.061919 1.418059 2.972474
N -1.354626 0.899223 2.198045
S -1.923072 2.956981 0.125499
C -2.313516 3.654311 -1.496715
H -1.567899 3.367276 -2.240993
H -3.317490 3.371336 -1.821427
H -2.279785 4.737867 -1.357025
H -2.549202 -5.557598 0.010813
C 1.508830 -0.336207 1.551965
H 2.259160 -0.804702 2.199167
H 0.845195 -1.137949 1.215007
S 0.613365 0.816712 2.635050
C 2.213767 0.312429 0.354186
H 2.759294 1.191118 0.699758
C 3.197599 -0.712530 -0.224824
O 2.785540 -1.679545 -0.887872
N 1.273577 0.713114 -0.672243
N 4.489772 -0.519463 0.050696
H 4.760024 0.294072 0.587655
C 5.521017 -1.448990 -0.378091
H 6.483455 -1.078916 -0.025552
H 5.344008 -2.443593 0.041099
H 5.544419 -1.524450 -1.468854
C 1.326792 1.835207 -1.424107
O 0.598308 1.952198 -2.424368
C 2.273502 2.930813 -1.011803
H 3.312178 2.591571 -1.088382
H 2.129440 3.785572 -1.673608
H 2.092851 3.239701 0.022752
H 0.643597 -0.009137 -1.010476
45
complexA_conf_7 Eopt -1899.573276
C -1.997366 1.781477 -0.230921
C -1.770730 0.454202 -0.624998
C -2.714754 -0.546788 -0.502196
C -3.966408 -0.230566 0.028176
C -4.222142 1.080397 0.434832
C -3.257025 2.070391 0.309884
H -2.476124 -1.556029 -0.819966
H -5.189850 1.334179 0.854748
H -3.495813 3.074551 0.639061
C -5.031095 -1.255096 0.179066
O -6.131656 -1.024391 0.657586
O -4.664730 -2.456625 -0.260364
C -5.633828 -3.511937 -0.144763
H -5.892737 -3.669668 0.904351
H -5.144733 -4.394731 -0.553433
N 0.043781 0.416974 -2.157925
N -0.466196 0.087458 -1.158098
S -0.710715 2.972336 -0.425510
C -1.357847 4.416149 0.452264
H -2.234332 4.832360 -0.048976
H -1.584213 4.179054 1.494377
H -0.551307 5.153342 0.422523
H -6.526386 -3.266980 -0.724280
C 2.013138 -1.056772 -0.607805
H 2.487582 -2.030412 -0.475996
H 1.807707 -0.929610 -1.683034
S 0.424148 -1.088496 0.218935
C 2.911694 0.106158 -0.155296
H 2.373160 1.036878 -0.339212
C 3.197031 0.107460 1.355222
O 2.794370 1.046477 2.065618
N 4.128525 0.204858 -0.961528
N 3.870173 -0.933917 1.849871
H 4.240204 -1.612544 1.187930
C 4.228673 -1.023666 3.253634
H 4.867473 -0.185959 3.550835
H 3.333112 -1.021784 3.881407
H 4.769550 -1.957155 3.410012
C 5.032847 -0.769060 -1.183973
O 4.966950 -1.884040 -0.633483
C 6.147786 -0.443339 -2.139592
H 6.120077 0.587666 -2.499006
H 7.102873 -0.627583 -1.639212
H 6.079459 -1.123026 -2.994914
H 4.266069 1.082981 -1.444320
45

complexA_conf_8 Eopt -1899.575450

C	-2.599830	-0.669168	0.040147
C	-1.803222	-0.101932	1.043850
C	-0.610151	-0.653896	1.462071
C	-0.146302	-1.814049	0.843814
C	-0.907111	-2.395910	-0.172954
C	-2.121577	-1.842273	-0.558270
H	-0.025646	-0.162537	2.233699
H	-0.553563	-3.287719	-0.678271
H	-2.685971	-2.323909	-1.347887
C	1.192169	-2.323046	1.237006
O	1.885041	-1.806460	2.101585
O	1.573088	-3.385141	0.535556
C	2.889913	-3.895000	0.800870
H	2.944981	-4.274794	1.823414
H	3.634457	-3.110918	0.647411
N	-2.912066	1.411044	2.515056
N	-2.185937	1.180970	1.626730
S	-4.104373	0.133506	-0.404871
C	-4.657023	-0.806991	-1.848111
H	-5.562301	-0.300096	-2.192022
H	-3.910783	-0.782207	-2.645621
H	-4.907207	-1.836817	-1.584138
H	3.033222	-4.703270	0.085600
C	-0.474796	1.941710	-0.788527
H	-1.142933	1.165311	-1.165669
H	-0.463624	2.722130	-1.558242
S	-1.226509	2.693086	0.687879
C	0.925339	1.370157	-0.574425
H	0.995743	0.919278	0.417590
C	1.236797	0.260301	-1.595727
O	0.569718	0.104354	-2.631163
N	1.930580	2.419718	-0.616922
N	2.276592	-0.519942	-1.282098
H	2.751050	-0.353776	-0.400346
C	2.663117	-1.656608	-2.097511
H	2.956141	-1.333569	-3.101085
H	3.511554	-2.148789	-1.622196
H	1.836814	-2.369416	-2.184711
C	3.061120	2.381661	0.115815
O	3.330661	1.414461	0.851282
C	3.983227	3.562775	0.000966
H	4.962270	3.212114	-0.339010
H	3.614334	4.326424	-0.687388
H	4.110134	4.003919	0.994347
H	1.773158	3.206529	-1.235138

45

complexA_conf_9 Eopt -1899.575217

C	2.931080	-1.570201	0.082816
C	1.707657	-0.899106	0.201517
C	1.589680	0.472918	0.072879
C	2.735382	1.237127	-0.137109
C	3.975379	0.603708	-0.237745
C	4.063884	-0.780302	-0.141690
H	0.616312	0.943586	0.161068
H	4.875768	1.182038	-0.410977
H	5.029471	-1.262396	-0.254167
C	2.577875	2.714329	-0.248343
O	1.498282	3.282945	-0.200650
O	3.734653	3.348601	-0.403936
C	3.677070	4.780489	-0.524097
H	3.091751	5.058787	-1.402927
H	4.711937	5.098942	-0.638191
N	0.211713	-2.257955	1.445354
N	0.484990	-1.620789	0.511985
S	3.147488	-3.326837	0.248116
C	2.010441	-3.984137	-1.012645
H	2.302414	-5.027468	-1.159657
H	0.968528	-3.954710	-0.689853
H	2.133323	-3.443835	-1.953539
H	3.240289	5.214438	0.377710
C	-2.249096	-1.867781	-0.119942
H	-2.820036	-2.613741	-0.678388
H	-1.838503	-2.381112	0.769758
S	-0.824371	-1.342486	-1.072963
C	-3.184764	-0.743865	0.344130
H	-3.936477	-1.198590	0.996895
C	-2.431888	0.263321	1.222826
O	-1.958547	-0.118108	2.307721
N	-3.878274	-0.147002	-0.779578
N	-2.311051	1.517470	0.786198
H	-2.717305	1.764822	-0.106093
C	-1.590735	2.529375	1.538476
H	-2.100532	2.747699	2.481963
H	-0.573064	2.195432	1.759467
H	-1.547195	3.437757	0.938050
C	-5.095921	0.429307	-0.670095
O	-5.694447	0.486569	0.416408
C	-5.685794	1.007225	-1.926516
H	-5.806676	2.086409	-1.789125
H	-5.070988	0.825252	-2.810893
H	-6.678771	0.574819	-2.080183
H	-3.422790	-0.135541	-1.684173

63

complexB_6sol_conf_1 Eopt -2358.102484

C	-3.484742	0.596719	-0.028659
C	-2.884515	0.043118	1.128600
C	-1.778741	0.586868	1.789994
C	-1.235121	1.749658	1.284333
C	-1.816235	2.345577	0.152762
C	-2.913102	1.790059	-0.484865
H	-1.367865	0.093824	2.663776
H	-1.393894	3.260608	-0.246932
H	-3.313957	2.285604	-1.359902
C	-0.010872	2.293049	1.935183
O	0.528491	1.766743	2.895100
O	0.431461	3.395830	1.350249

C 1.646652 3.962003 1.871416
H 1.497422 4.272406 2.907727
H 1.854744 4.823837 1.240109
N -3.814271 -2.057024 2.057976
N -3.395425 -1.121134 1.641732
S -4.824706 -0.221388 -0.786845
C -5.113171 0.755747 -2.281983
H -5.440981 1.767319 -2.035881
H -4.223797 0.770675 -2.916053
H -5.918859 0.235471 -2.805354
H 2.456049 3.232640 1.804312
C 0.925638 -1.556217 0.906237
H 0.683558 -0.558816 1.291789
H 1.779946 -1.922604 1.484885
S -0.491041 -2.691861 1.118251
C 1.424394 -1.402279 -0.544058
H 1.608871 -2.388957 -0.972075
C 0.424470 -0.653417 -1.421349
O 0.362425 0.594696 -1.377142
N 2.670134 -0.645533 -0.564588
N -0.355343 -1.382939 -2.210025
H -0.293561 -2.400306 -2.151708
C -1.413595 -0.798943 -3.015204
H -2.058405 -1.603324 -3.369929
H -2.005069 -0.100702 -2.418730
H -1.002210 -0.265070 -3.876814
C 3.905454 -1.146801 -0.431683
O 4.881397 -0.371562 -0.295756
C 4.096133 -2.635251 -0.474823
H 5.150360 -2.866954 -0.321195
H 3.501410 -3.127433 0.301216
H 3.778050 -3.031497 -1.444836
H 2.583762 0.358865 -0.444297
H 1.504256 2.134906 -1.317816
H -0.482043 1.792435 -2.530549
H 6.590769 -0.945524 -0.216270
H 4.683548 1.419682 -0.247361
H -0.857974 -2.255440 3.232292
H -0.370268 -3.915121 -0.700347
O -0.292288 -4.295703 -1.612756
H -1.187304 -4.591015 -1.831942
O -1.024942 -2.058060 4.188702
H -0.666611 -2.823535 4.658948
O 2.072143 2.921816 -1.398945
H 2.903639 2.691058 -0.932534
O -0.793326 2.516681 -3.111197
H -0.689659 3.311379 -2.570026
O 7.541201 -1.187756 -0.173414
H 8.009558 -0.344370 -0.237880
O 4.570171 2.395047 -0.195741
H 5.153045 2.747172 -0.882979
63
complexB_6sol_conf_10
Eopt -2358.102371
C 2.889415 -0.291756 1.498422
C 2.904526 -0.183713 0.087741
C 2.436309 0.920277 -0.633012
C 1.920061 1.979797 0.085585
C 1.867606 1.904208 1.487145
C 2.334719 0.797062 2.177776
H 2.482125 0.925047 -1.716133
H 1.450544 2.729293 2.053402
H 2.274218 0.789337 3.259077
C 1.403524 3.155963 -0.669240
O 1.230140 3.153317 -1.876621
O 1.166290 4.199901 0.111908
C 0.612113 5.366693 -0.520192
H 0.475838 6.088652 0.283145
H -0.346610 5.119728 -0.980956
N 3.738265 -2.097746 -1.247413
N 3.380148 -1.241088 -0.646305
S 3.556888 -1.708519 2.273218
C 2.753913 -1.679864 3.897436
H 1.671700 -1.584049 3.783053
H 2.986513 -2.647158 4.349218
H 3.155461 -0.884165 4.527083
H 1.307441 5.752762 -1.268299
C -0.625109 -0.253388 -2.266332
H -0.216878 0.648919 -1.794398
H -1.214332 0.065802 -3.131525
S 0.732033 -1.361318 -2.799319
C -1.588665 -0.931399 -1.287129
H -1.987335 -1.832807 -1.753068
C -0.880971 -1.288065 0.020920
O -0.469459 -0.380498 0.776098
N -2.705546 -0.040409 -0.987739
N -0.757136 -2.575502 0.322435
H -0.975954 -3.256455 -0.404782
C -0.040867 -3.022833 1.503880
H -0.143779 -4.105700 1.578384
H 1.022009 -2.770040 1.435232
H -0.459903 -2.561969 2.401366
C -3.966487 -0.405917 -0.720451
O -4.795662 0.451441 -0.330307
C -4.373425 -1.837599 -0.917252
H -3.782258 -2.499439 -0.276810
H -5.429720 -1.944361 -0.668830
H -4.214779 -2.144990 -1.955962
H -2.465064 0.925003 -0.766046
H -0.739596 -0.127755 2.560599
H -0.956893 1.403806 0.674511
H -6.066919 -0.082945 0.855425
H -4.258207 1.571331 0.992767
H -0.242925 -3.343004 -2.481723
H 1.886228 0.104982 -3.935239
O 2.434586 0.783673 -4.405195
H 2.130381 0.750231 -5.322575
O -0.732636 -4.162007 -2.216764
H -0.039419 -4.795251 -1.982827
O -0.992476 0.206115 3.445154

H -1.476301 1.021418 3.251054
O -1.354026 2.294534 0.587802
H -2.202480 2.234285 1.076128
O -6.705803 -0.302409 1.565945
H -6.448405 0.270655 2.301011
O -3.904864 2.084824 1.751287
H -4.276275 2.971522 1.642244
63
complexB_6sol_conf_11
Eopt -2358.108502
C -3.791400 -0.126047 0.133887
C -2.620990 -0.253739 0.922889
C -1.670119 0.755337 1.107153
C -1.867552 1.953491 0.449390
C -3.008871 2.116822 -0.350032
C -3.950788 1.112088 -0.500322
H -0.806555 0.570500 1.734152
H -3.158890 3.056548 -0.871246
H -4.814386 1.296102 -1.126572
C -0.854542 3.042982 0.512271
O -0.973590 4.107336 -0.072160
O 0.194832 2.712011 1.248638
C 1.283395 3.647853 1.308903
H 2.029987 3.173480 1.943574
H 0.945693 4.587855 1.750446
N -2.086513 -2.398661 2.042327
N -2.352721 -1.450071 1.538524
S -4.912719 -1.453604 0.028599
C -6.185988 -0.840311 -1.100890
H -6.694939 0.031813 -0.685934
H -5.764595 -0.620693 -2.084036
H -6.899693 -1.662787 -1.191806
H 1.682542 3.818064 0.306495
C 2.156894 -0.107863 1.622362
H 1.502074 0.638432 2.081665
H 3.168402 0.082139 1.992035
S 1.603855 -1.789368 2.112462
C 2.165092 0.174225 0.105052
H 2.313296 1.250754 -0.022018
C 3.370219 -0.483810 -0.563490
O 4.494203 0.049711 -0.414213
N 0.895805 -0.205485 -0.478626
N 3.193833 -1.590950 -1.271590
H 2.286836 -2.056173 -1.245062
C 4.302182 -2.304875 -1.882996
H 4.830064 -1.657795 -2.588193
H 5.007742 -2.658922 -1.125186
H 3.897357 -3.161859 -2.421509
C 0.313994 0.320901 -1.563814
O -0.782509 -0.130666 -1.969194
C 0.990236 1.463278 -2.263897
H 2.006888 1.190644 -2.565709
H 0.412948 1.737550 -3.147364
H 1.063482 2.330598 -1.598318
H 0.404730 -0.959620 0.000717
H 6.197510 -0.438611 -0.810937
H 4.663808 1.638856 0.429932
H -1.352669 -1.725218 -1.401324
H -1.833586 0.859426 -3.054514
H 1.231635 -3.107553 0.277090
H 0.068838 -1.008876 3.469104
O -0.604548 -0.584178 4.060364
H -0.506938 -1.044952 4.905193
O 1.043026 -3.542857 -0.585933
H 0.125292 -3.258049 -0.792091
O 7.152058 -0.599359 -0.965129
H 7.185466 -1.481917 -1.358782
O 4.792547 2.515516 0.853070
H 5.749765 2.597149 0.960586
O -1.552334 -2.620281 -1.046508
H -1.974041 -3.093263 -1.776939
O -2.442813 1.361760 -3.637135
H -1.936626 1.506151 -4.448047
63
complexB_6sol_conf_12
Eopt -2358.105921
C -3.116680 0.483694 -0.199256
C -2.389367 1.315110 0.686606
C -1.358358 2.182187 0.305755
C -1.002942 2.202206 -1.027801
C -1.701706 1.394350 -1.940275
C -2.739026 0.568237 -1.544909
H -0.861060 2.787625 1.055700
H -1.420588 1.417059 -2.988079
H -3.246457 -0.029848 -2.291278
C 0.144427 3.017542 -1.513248
O 0.597121 2.927076 -2.642386
O 0.620511 3.835793 -0.585614
C 1.764427 4.630492 -0.945526
H 1.511653 5.293251 -1.775564
H 2.598787 3.980137 -1.214691
N -2.878375 1.203070 3.113799
N -2.672063 1.254734 2.027674
S -4.385108 -0.536512 0.420335
C -4.723440 -1.661461 -0.956901
H -3.809243 -2.169769 -1.267285
H -5.426365 -2.395166 -0.554866
H -5.190811 -1.138447 -1.792770
H 2.001652 5.207653 -0.053545
C 1.572879 0.692045 1.283505
H 1.137342 1.640812 0.964082
H 2.613946 0.904663 1.543575
S 0.661779 0.074297 2.749837
C 1.597632 -0.242395 0.048916
H 1.813172 0.386484 -0.820312
C 2.775243 -1.210654 0.146496
O 3.907350 -0.798524 -0.195605
N 0.322893 -0.902100 -0.144371
N 2.579377 -2.440239 0.601845
H 1.670097 -2.698082 0.989045

C 3.679702 -3.374439 0.773189
H 4.166973 -3.572451 -0.185148
H 4.422342 -2.981941 1.474334
H 3.274823 -4.307273 1.165953
C -0.108588 -1.458150 -1.283772
O -1.172123 -2.119230 -1.303916
C 0.699637 -1.247979 -2.531783
H 0.215844 -1.754573 -3.367149
H 0.780377 -0.178212 -2.755410
H 1.714924 -1.641628 -2.414046
H -0.254998 -1.011988 0.689437
H 5.613161 -1.386108 -0.314104
H 4.259033 0.850786 -0.794267
H -1.769777 -2.972154 0.138116
H -2.031439 -2.359337 -2.882558
H 0.392159 -2.169902 2.597784
H 0.030527 2.110540 3.277886
O -0.251817 3.049133 3.418144
H 0.227140 3.331135 4.209867
O 0.336471 -3.132675 2.399230
H -0.501452 -3.237207 1.897023
O 6.572306 -1.524206 -0.457578
H 6.881799 -0.678267 -0.809860
O 4.562779 1.719005 -1.138095
H 5.497588 1.580509 -1.343449
O -2.022536 -3.489517 0.935484
H -1.952132 -4.414465 0.661210
O -2.521089 -2.485423 -3.723731
H -1.843394 -2.402824 -4.408453
63
complexB_6sol_conf_14
Eopt -2358.102260
C 2.963660 -0.233041 1.422836
C 2.914314 -0.165546 0.009543
C 2.395205 0.909924 -0.719774
C 1.893485 1.981510 -0.009293
C 1.901006 1.943373 1.394932
C 2.415800 0.863784 2.095261
H 2.395367 0.885158 -1.803441
H 1.492874 2.776681 1.955805
H 2.399020 0.885387 3.177781
C 1.331738 3.130370 -0.773495
O 1.098788 3.089209 -1.970132
O 1.128252 4.197339 -0.014182
C 0.534368 5.340658 -0.653201
H 1.189727 5.708760 -1.445202
H 0.430563 6.085414 0.134029
N 3.738629 -2.097448 -1.304292
N 3.382837 -1.231901 -0.714375
S 3.698594 -1.613485 2.198168
C 3.083716 -1.500635 3.897303
H 3.519101 -0.649848 4.423932
H 1.992312 -1.456652 3.908025
H 3.412116 -2.424809 4.379193
H -0.443223 5.073396 -1.059707
C -0.696062 -0.338431 -2.250424
H -0.287393 0.587725 -1.827481
H -1.311626 -0.062865 -3.112225
S 0.660816 -1.451639 -2.774366
C -1.624976 -0.985421 -1.218630
H -2.017683 -1.914663 -1.632222
C -0.882995 -1.264904 0.089142
O -0.473110 -0.313322 0.789030
N -2.750791 -0.101017 -0.934823
N -0.725936 -2.532424 0.451655
H -0.949961 -3.254501 -0.232492
C 0.021415 -2.903221 1.640364
H 1.065323 -2.584638 1.559242
H -0.421511 -2.447906 2.529307
H -0.011568 -3.988207 1.741562
C -4.004754 -0.473148 -0.644342
O -4.841650 0.386654 -0.277272
C -4.393822 -1.916431 -0.786820
H -5.445853 -2.028428 -0.523246
H -4.241830 -2.257256 -1.816069
H -3.787077 -2.547783 -0.130295
H -2.523626 0.875368 -0.755536
H -0.974506 1.476031 0.662790
H -0.648556 -0.000241 2.561375
H -4.291602 1.506490 1.044817
H -6.114367 -0.111138 0.922180
H 1.755800 0.015632 -3.983691
H -0.265447 -3.431193 -2.326936
O -0.741580 -4.239295 -2.009137
H -0.037962 -4.861046 -1.775680
O 2.250764 0.677538 -4.529641
H 1.904274 0.559077 -5.424766
O -1.397672 2.356955 0.618624
H -2.248966 2.242703 1.092473
O -0.816734 0.330490 3.467619
H -1.343933 1.129506 3.327846
O -3.926860 2.017546 1.799143
H -4.329126 2.893835 1.719264
O -6.752322 -0.312042 1.638922
H -6.459970 0.239885 2.377156
63
complexB_6sol_conf_15
Eopt -2358.101569
C 3.265906 0.013371 -0.093180
C 2.895918 -0.454713 1.192757
C 1.981407 0.181371 2.040857
C 1.413174 1.360170 1.601149
C 1.766814 1.866419 0.340476
C 2.662134 1.210928 -0.488864
H 1.736422 -0.250730 3.003872
H 1.320430 2.790099 -0.009807
H 2.868678 1.631504 -1.465076
C 0.407199 2.027363 2.471609
O 0.087134 1.612423 3.572815
O -0.099034 3.119442 1.913857

C -1.083594 3.848657 2.667180
H -1.965569 3.230285 2.848139
H -1.343411 4.703266 2.044131
N 3.896137 -2.559524 2.038063
N 3.449290 -1.619201 1.660421
S 4.416975 -0.876336 -1.051932
C 4.374459 -0.014050 -2.643286
H 5.028462 -0.597209 -3.296095
H 3.362470 -0.016696 -3.053043
H 4.765790 1.001384 -2.558665
H -0.650234 4.180957 3.612569
C -1.581909 -0.944620 1.333085
H -2.629051 -1.081519 1.621318
H -1.293546 0.044259 1.703905
S -0.562708 -2.227686 2.151394
C -1.480971 -0.918617 -0.219231
H -0.842786 -1.734732 -0.560077
C -2.875499 -1.087992 -0.828760
O -3.578729 -0.092325 -1.110027
N -0.906253 0.338066 -0.665281
N -3.297308 -2.335566 -0.995751
H -2.681185 -3.101271 -0.709635
C -4.639294 -2.646140 -1.457021
H -5.388782 -2.347396 -0.717979
H -4.848980 -2.133540 -2.399503
H -4.705215 -3.722840 -1.614934
C -0.275574 0.559874 -1.826697
O -0.079736 1.732632 -2.217876
C 0.232545 -0.612466 -2.610127
H 0.927282 -1.198971 -1.998615
H -0.593995 -1.268795 -2.903363
H 0.743644 -0.255433 -3.504653
H -1.282249 1.161134 -0.202182
H -3.419240 1.501176 -0.080749
H -5.354970 0.159039 -0.737897
H -0.914261 3.120108 -1.446287
H 1.224568 2.187403 -3.379579
H -1.257493 -3.820281 0.833377
H 0.920708 -2.770379 0.639611
O 1.602104 -3.107969 0.001093
H 1.220760 -3.932484 -0.332903
O -1.563250 -4.446081 0.122060
H -2.263013 -4.969519 0.538235
O -3.188664 2.256783 0.493440
H -2.655302 2.866807 -0.061629
O -6.269527 0.407122 -0.493440
H -6.169416 1.234531 -0.003448
O -1.448233 3.845865 -1.049279
H -0.889712 4.195049 -0.339473
O 1.961164 2.485408 -3.954218
H 1.605739 3.248996 -4.430230
63
complexB_6sol_conf_16
Eopt -2358.102463
C -2.949310 0.924561 1.232853
C -2.890253 0.177672 0.032391
C -2.394463 -1.126717 -0.074185
C -1.929914 -1.726774 1.079567
C -1.950133 -1.011691 2.287098
C -2.438658 0.282581 2.365651
H -2.389846 -1.626298 -1.034923
H -1.572025 -1.484394 3.187547
H -2.437093 0.786787 3.324063
C -1.396404 -3.118232 1.066199
O -1.229489 -3.781859 2.075442
O -1.129249 -3.546889 -0.158342
C -0.599735 -4.877708 -0.281359
H -1.309578 -5.602275 0.122797
H 0.356319 -4.945274 0.241860
N -3.684012 1.238703 -2.062168
N -3.333462 0.766872 -1.125026
S -3.654762 2.522817 1.225570
C -2.983947 3.263847 2.736880
H -3.444580 2.836078 3.628538
H -1.898034 3.152650 2.762685
H -3.239185 4.324196 2.673497
H -0.462614 -5.031481 -1.350322
C 0.700496 -0.800325 -2.049427
H 0.287814 -1.401877 -1.230468
H 1.329097 -1.458728 -2.656939
S -0.651816 -0.101609 -3.068284
C 1.613096 0.278484 -1.459781
H 2.004294 0.891070 -2.272519
C 0.859141 1.158287 -0.461518
O 0.429802 0.669506 0.606313
N 2.740828 -0.343400 -0.772521
N 0.721282 2.444761 -0.759914
H 0.965014 2.741365 -1.703787
C -0.013130 3.360916 0.094818
H 0.074295 4.364186 -0.322829
H -1.071759 3.087355 0.142500
H 0.402404 3.356741 1.105207
C 3.980234 0.154471 -0.660737
O 4.811616 -0.409028 0.090761
C 4.358410 1.368274 -1.459674
H 4.227587 1.182993 -2.530626
H 3.729111 2.220914 -1.186351
H 5.402219 1.614144 -1.262892
H 2.516605 -1.125284 -0.158671
H 0.927711 -0.958620 1.309096
H 0.702435 1.215007 2.316771
H 4.232492 -0.944428 1.717078
H 6.245278 0.487575 0.724459
H 0.270232 1.857462 -3.635464
H -1.795350 -1.960564 -3.311399
O -2.311493 -2.799231 -3.421138
H -1.971493 -3.184803 -4.240101
O 0.730272 2.724701 -3.762050
H 0.013409 3.370435 -3.834644
O 1.318180 -1.796483 1.632276

H 2.161960 -1.530487 2.056676
O 0.929844 1.307042 3.264566
H 1.304244 0.443933 3.491362
O 3.866884 -1.140490 2.607495
H 4.225887 -2.009840 2.834790
O 7.001622 0.929497 1.166554
H 6.869440 0.743572 2.106144
63
complexB_6sol_conf_17
Eopt -2358.104863
C -2.854978 -1.136420 0.488080
C -2.055970 -1.820649 -0.459290
C -0.802146 -2.381205 -0.198450
C -0.310858 -2.271179 1.087816
C -1.070838 -1.597996 2.059368
C -2.303926 -1.037070 1.771961
H -0.256251 -2.874999 -0.994151
H -0.678184 -1.507839 3.066920
H -2.838763 -0.514323 2.555269
C 1.004072 -2.859159 1.469182
O 1.448620 -2.828552 2.604772
O 1.628118 -3.424568 0.445352
C 2.897410 -4.041524 0.720470
H 2.751034 -4.909172 1.367145
H 3.569617 -3.326566 1.195321
N -2.925336 -2.060038 -2.769464
N -2.537570 -1.953724 -1.739473
S -4.401660 -0.501069 0.003210
C -5.103315 0.133887 1.545670
H -6.114378 0.452979 1.280684
H -5.164390 -0.651189 2.301912
H -4.531890 0.994780 1.897250
H 3.283467 -4.349404 -0.249068
C 0.747103 1.343206 -2.151580
H 1.472111 0.936885 -2.864213
H 0.786461 2.434636 -2.237876
S -0.939885 0.776227 -2.582298
C 1.248574 0.953115 -0.745469
H 1.385252 -0.132122 -0.693297
C 2.626534 1.598750 -0.562148
O 3.660598 0.939849 -0.803870
N 0.302257 1.388126 0.265475
N 2.645718 2.881143 -0.206957
H 1.759263 3.344707 -0.004050
C 3.859581 3.645821 0.015707
H 4.033352 3.784321 1.087188
H 4.708700 3.119080 -0.419133
H 3.762945 4.624569 -0.459072
C 0.447290 1.311038 1.596204
O -0.370471 1.879192 2.353920
C 1.624755 0.567565 2.156618
H 1.346869 0.143374 3.123412
H 1.982823 -0.226686 1.499369
H 2.445550 1.276088 2.320380
H -0.495498 1.918666 -0.070688
H 3.514493 -0.781534 -1.476570
H 4.418277 -0.137486 0.550068
H -1.956263 2.442082 1.777573
H -0.088220 3.986290 1.264047
H -1.805430 2.611332 -1.590632
H -0.100745 -1.153747 -3.079267
O 0.284834 -2.040554 -3.307611
H 0.326395 -2.040405 -4.273850
O -1.980979 3.442786 -1.088398
H -2.420325 3.158729 -0.255869
O 3.465001 -1.692343 -1.831574
H 4.185559 -2.163461 -1.392853
O 4.930350 -0.680068 1.178879
H 4.981055 -1.547965 0.755440
O -2.848138 2.709340 1.458197
H -3.026336 3.553724 1.895254
O 0.112619 4.529904 0.485634
H -0.531446 4.214602 -0.185528
63
complexB_6sol_conf_18
Eopt -2358.107403
C -3.169566 0.086968 -1.102555
C -2.727126 0.695898 0.094922
C -1.752160 1.697757 0.171503
C -1.178261 2.117392 -1.012852
C -1.578230 1.527171 -2.221691
C -2.541708 0.532110 -2.269876
H -1.469133 2.098797 1.138157
H -1.117488 1.856083 -3.147240
H -2.815177 0.110824 -3.229464
C -0.109267 3.154718 -1.032513
O 0.287469 3.689359 -2.054295
O 0.363140 3.418039 0.176059
C 1.442414 4.363755 0.263399
H 1.117340 5.338557 -0.105764
H 2.297639 4.001528 -0.311210
N -3.584228 -0.111644 2.275308
N -3.235628 0.249920 1.289572
S -4.441583 -1.110431 -1.052993
C -4.182836 -2.025180 -2.595733
H -4.839008 -2.896053 -2.525155
H -4.468771 -1.431894 -3.465785
H -3.145076 -2.358325 -2.667825
H 1.688389 4.417955 1.322349
C 1.110333 0.401293 1.972168
H 0.878006 1.108818 1.167198
H 1.992890 0.782846 2.495180
S -0.300827 0.276176 3.131206
C 1.516320 -0.935149 1.336334
H 1.788634 -1.639774 2.122879
C 0.388633 -1.509973 0.478186
O 0.091524 -0.971338 -0.611367
N 2.673571 -0.717988 0.479187
N -0.222215 -2.603775 0.919273
H -0.036527 -2.899974 1.878351

C -1.364220 -3.178199 0.229637
H -1.626919 -4.114142 0.723509
H -2.225498 -2.503983 0.265770
H -1.118130 -3.382668 -0.814630
C 3.767075 -1.480194 0.353283
O 4.626949 -1.196723 -0.517101
C 3.948351 -2.654743 1.270228
H 4.894664 -3.144273 1.038193
H 3.954807 -2.332650 2.316458
H 3.130469 -3.371094 1.142978
H 2.574448 0.026338 -0.206976
H 0.469765 -2.059740 -2.093016
H 1.076057 0.283714 -1.617580
H 4.633001 0.669527 -1.098948
H 3.917685 -1.302438 -2.219862
H -0.067229 -1.838055 3.761887
H -0.394029 2.445808 3.423332
O -0.422411 3.433215 3.497531
H -0.089402 3.619023 4.386313
O 0.074275 -2.812583 3.870222
H -0.796524 -3.161207 4.107533
O 0.824394 -2.557971 -2.854256
H 1.718340 -2.187272 -2.980481
O 1.743837 0.763682 -2.148276
H 2.256209 0.046888 -2.578737
O 4.444853 1.588370 -1.372983
H 3.499901 1.558247 -1.612114
O 3.388968 -1.335206 -3.044367
H 3.976229 -1.021429 -3.746062
63
complexB_6sol_conf_19
Eopt -2358.097430
C -3.870445 -1.167916 -0.351271
C -3.696843 -0.371668 0.808799
C -3.274102 0.963318 0.802341
C -3.031549 1.554830 -0.419087
C -3.191780 0.799565 -1.594416
C -3.601985 -0.521930 -1.564483
H -3.146418 1.501844 1.734690
H -2.985422 1.256491 -2.555630
H -3.705563 -1.057490 -2.499284
C -2.579160 2.973520 -0.439626
O -2.309890 3.607945 0.566731
O -2.494043 3.462164 -1.668179
C -1.957962 4.789300 -1.802546
H -1.962547 4.992030 -2.871974
H -0.938330 4.816434 -1.411103
N -4.062664 -1.395554 3.031511
N -3.903989 -0.941943 2.034049
S -4.384803 -2.822653 -0.193393
C -4.423434 -3.416856 -1.901294
H -3.429544 -3.378133 -2.350377
H -4.744352 -4.458269 -1.828635
H -5.149005 -2.857406 -2.494169
H -2.589204 5.506169 -1.273891
C 1.220326 -1.548138 1.385916
H 1.508921 -0.924827 2.240566
H 1.572697 -2.564630 1.586078
S -0.597197 -1.528417 1.180315
C 1.964490 -1.049463 0.142533
H 1.709914 -1.689399 -0.701267
C 1.598772 0.399543 -0.177454
O 1.980924 1.320431 0.578308
N 3.403273 -1.134508 0.361327
N 0.900130 0.633224 -1.281050
H 0.492289 -0.163360 -1.769299
C 0.450166 1.968221 -1.635776
H -0.310636 1.879531 -2.411005
H 0.029369 2.471583 -0.761697
H 1.280601 2.569125 -2.017389
C 4.336133 -1.421559 -0.557337
O 5.550305 -1.320895 -0.264466
C 3.900821 -1.878789 -1.919663
H 4.782630 -2.064385 -2.533039
H 3.312918 -2.799494 -1.848062
H 3.280710 -1.117557 -2.402090
H 3.746923 -0.768333 1.246408
H 3.148651 1.223518 1.963222
H 2.422576 3.034989 0.242714
H 6.063119 -0.006930 0.876589
H 6.864832 -1.720899 -1.431663
H -0.698903 -1.836796 -1.039332
H -1.028822 -0.321556 2.963039
O -1.223297 0.222571 3.766879
H -1.200903 -0.411790 4.496089
O -0.611747 -1.881377 -2.025841
H 0.073068 -2.547893 -2.175919
O 3.786090 1.148878 2.703362
H 4.662956 1.055112 2.276060
O 2.777000 3.947197 0.184868
H 3.316813 4.039138 0.980303
O 6.294185 0.773919 1.425369
H 6.789389 0.411989 2.173505
O 7.590851 -1.935179 -2.056890
H 8.402318 -1.777313 -1.556012
63
complexB_6sol_conf_2 Eopt
-2358.098510
C 3.354757 0.503115 0.501133
C 2.881156 0.081034 -0.764540
C 1.915852 0.757033 -1.521324
C 1.387083 1.918328 -0.997804
C 1.818752 2.364070 0.262635
C 2.769884 1.674641 0.996809
H 1.612079 0.372003 -2.487757
H 1.395223 3.268568 0.684219
H 3.058756 2.059894 1.966767
C 0.354850 2.641780 -1.791455
O -0.157879 2.185692 -2.800035
O 0.059157 3.827953 -1.280865

C	-0.960546	4.586899	-1.954238
H	-1.888817	4.013918	-1.993255
H	-0.627132	4.843553	-2.961943
N	3.825691	-1.983392	-1.757364
N	3.405469	-1.059618	-1.317088
S	4.594903	-0.413817	1.316807
C	4.669778	0.356446	2.948068
H	5.415657	-0.221790	3.499792
H	5.004470	1.393727	2.888524
H	3.709253	0.274013	3.462468
H	-1.090358	5.485488	-1.353652
C	-1.077681	-1.366304	-1.374348
H	-0.796256	-0.320041	-1.544218
H	-1.914530	-1.593664	-2.042374
S	0.317232	-2.487858	-1.744804
C	-1.617935	-1.500200	0.058070
H	-1.824233	-2.550845	0.266657
C	-0.636944	-0.959527	1.093955
O	-0.450981	0.269790	1.204970
N	-2.854248	-0.743788	0.214028
N	-0.017685	-1.843077	1.871734
H	-0.142965	-2.827361	1.673447
C	0.991777	-1.459430	2.843584
H	0.531192	-0.974133	3.708107
H	1.515780	-2.357401	3.169714
H	1.711552	-0.770900	2.395335
C	-4.104965	-1.213693	0.134320
O	-5.073018	-0.420681	0.223926
C	-4.318947	-2.687112	-0.051247
H	-5.385456	-2.883701	-0.163681
H	-3.787420	-3.047929	-0.937631
H	-3.942185	-3.238797	0.816415
H	-2.762967	0.265888	0.257823
H	-1.433436	1.863309	0.904150
H	0.110749	1.324443	2.636337
H	-6.776655	-0.971189	0.405926
H	-4.786014	1.156067	1.082850
H	1.614839	-3.196563	-0.056871
H	0.912029	-1.608342	-3.645586
O	1.183658	-1.206994	-4.512705
H	0.988717	-1.890226	-5.168644
O	2.250662	-3.597514	0.581915
H	2.664468	-4.325565	0.098220
O	-1.995811	2.641530	0.731392
H	-2.877581	2.407504	1.096334
O	0.226456	1.980284	3.353105
H	-0.647909	2.385455	3.435897
O	-7.717747	-1.231268	0.510048
H	-8.108955	-0.530377	1.048685
O	-4.571054	1.951332	1.619173
H	-5.149400	2.647463	1.277634

63
complexB_6sol_conf_20
Eopt -2358.106486

C	-3.238877	0.426101	-0.100040
C	-2.486122	1.175921	0.836472
C	-1.516795	2.129314	0.506570
C	-1.294362	2.375088	-0.834242
C	-2.033300	1.666262	-1.795202
C	-2.974981	0.713075	-1.444010
H	-0.980185	2.645519	1.293996
H	-1.850675	1.857978	-2.847485
H	-3.499037	0.181240	-2.228399
C	-0.292419	3.380958	-1.285950
O	-0.170716	3.727159	-2.449398
O	0.444537	3.850541	-0.290651
C	1.451246	4.821300	-0.631468
H	2.187399	4.368745	-1.298304
H	1.909396	5.101224	0.315012
N	-2.924102	0.838945	3.253287
N	-2.728563	0.989374	2.174514
S	-4.384605	-0.758944	0.466322
C	-5.272171	-1.229145	-1.039479
H	-6.046741	-1.923392	-0.704768
H	-5.741318	-0.357601	-1.500430
H	-4.613463	-1.740195	-1.742889
H	0.989200	5.689501	-1.105238
C	1.238559	-1.540984	1.817925
H	1.990640	-1.263496	2.562604
H	1.399330	-2.597843	1.576056
S	-0.441055	-1.329379	2.512169
C	1.531423	-0.705557	0.556570
H	1.474310	0.358754	0.804221
C	2.970824	-0.999584	0.131759
O	3.884300	-0.193810	0.421002
N	0.574701	-1.027704	-0.488310
N	3.198520	-2.147681	-0.492824
H	2.415864	-2.788327	-0.662153
C	4.532322	-2.554784	-0.898814
H	4.980821	-1.803545	-1.555084
H	5.180278	-2.699091	-0.029183
H	4.450150	-3.496802	-1.441522
C	0.538175	-0.537587	-1.735662
O	-0.273654	-0.993199	-2.570973
C	1.500472	0.552396	-2.113691
H	2.435276	0.099405	-2.465429
H	1.072864	1.131210	-2.934309
H	1.741856	1.217092	-1.280198
H	-0.092056	-1.761985	-0.280881
H	5.697402	-0.237384	0.416811
H	3.554820	1.528575	0.791863
H	-1.470370	-2.254463	-2.119443
H	0.693838	-3.738865	-1.811213
H	-1.102474	-3.075341	1.243427
H	0.053412	0.748025	3.086174
O	0.311248	1.677832	3.301341
H	0.744241	1.615659	4.163883
O	-1.186437	-3.840290	0.624409
H	-1.632674	-3.481038	-0.173157
O	6.672471	-0.143653	0.451460

H 6.823551 0.606668 1.041946
O 3.429370 2.490650 0.944876
H 4.313700 2.869596 0.851789
O -2.111157 -2.964764 -1.885872
H -1.820039 -3.733629 -2.395848
O 1.098590 -4.135453 -1.025659
H 0.394959 -4.060312 -0.343072
63
complexB_6sol_conf_21
Eopt -2358.106016
C -2.954365 -0.575278 -1.332109
C -2.770774 -0.417079 0.063092
C -2.329172 0.757360 0.683572
C -2.033522 1.836440 -0.124725
C -2.155236 1.704668 -1.518570
C -2.601779 0.533685 -2.109165
H -2.217286 0.785915 1.761867
H -1.900664 2.541257 -2.159230
H -2.690668 0.493380 -3.187505
C -1.559831 3.097277 0.509242
O -1.318211 3.210437 1.701709
O -1.437219 4.089273 -0.357039
C -0.937047 5.339018 0.151036
H -0.900506 6.001917 -0.711625
H 0.061622 5.195826 0.568512
N -3.095020 -2.345107 1.584019
N -2.988502 -1.491450 0.887651
S -3.613713 -2.061797 -1.960438
C -3.266627 -1.945386 -3.733119
H -3.548034 -2.918825 -4.141878
H -3.871763 -1.170013 -4.205958
H -2.201736 -1.775485 -3.905385
H -1.616641 5.732732 0.909366
C 0.869669 0.023000 2.326945
H 0.394450 0.864938 1.807168
H 1.559836 0.442852 3.065262
S -0.395773 -1.000983 3.169982
C 1.715260 -0.761701 1.317271
H 2.162053 -1.619494 1.821178
C 0.865979 -1.230395 0.133709
O 0.418305 -0.390465 -0.676422
N 2.792398 0.086562 0.817821
N 0.668050 -2.538758 0.015524
H 0.971171 -3.129088 0.790456
C -0.173918 -3.145332 -0.999314
H 0.326556 -4.027227 -1.405591
H -1.131959 -3.452352 -0.569508
H -0.349228 -2.433046 -1.805617
C 3.998874 -0.320177 0.399837
O 4.779853 0.499120 -0.141001
C 4.402359 -1.750922 0.608572
H 3.720294 -2.423189 0.078660
H 5.415337 -1.894594 0.231873
H 4.372081 -2.010481 1.671694
H 2.540136 1.043372 0.578185
H 0.577856 -0.326682 -2.470128
H 0.829024 1.410221 -0.781149
H 4.086860 1.560934 -1.431850
H 5.957860 -0.094285 -1.378012
H -1.239875 0.891722 3.985023
H 0.513614 -3.021370 2.929533
O 0.957168 -3.872081 2.683771
H 0.233469 -4.509604 2.605088
O -1.540836 1.803455 4.214424
H -1.486084 2.283237 3.368625
O 0.749283 -0.144336 -3.416908
H 1.300299 0.650915 -3.402409
O 1.177065 2.322018 -0.855031
H 2.007273 2.226478 -1.368393
O 3.656874 2.051271 -2.165498
H 4.027890 2.943336 -2.114326
O 6.559363 -0.343259 -2.111853
H 6.177665 0.087914 -2.888590
63
complexB_6sol_conf_22
Eopt -2358.102725
C 3.120199 0.664365 0.856193
C 2.727215 0.499210 -0.493571
C 1.763897 1.277087 -1.145841
C 1.145890 2.273666 -0.417615
C 1.487770 2.453342 0.932593
C 2.445660 1.669205 1.556938
H 1.524573 1.090223 -2.186848
H 0.991324 3.223120 1.512671
H 2.673965 1.854892 2.599048
C 0.106449 3.099385 -1.093727
O -0.376630 2.814352 -2.176698
O -0.226654 4.174173 -0.392432
C -1.271852 5.000583 -0.934047
H -2.189916 4.418369 -1.037430
H -0.965734 5.405657 -1.900676
N 3.665237 -1.321682 -1.884924
N 3.290549 -0.505480 -1.238493
S 4.379861 -0.346375 1.517232
C 4.228565 -0.074895 3.301666
H 4.553860 0.930100 3.575887
H 3.206094 -0.259105 3.638935
H 4.898369 -0.807225 3.758093
H -1.406438 5.801476 -0.209114
C -1.017492 -0.988176 -1.915371
H -0.803249 0.065772 -1.700108
H -1.851283 -1.015006 -2.623614
S 0.452072 -1.803169 -2.643118
C -1.499882 -1.667590 -0.628974
H -1.693813 -2.720204 -0.838082
C -0.462976 -1.541877 0.487134
O -0.222709 -0.425342 0.996497
N -2.745919 -1.050794 -0.186755
N 0.139217 -2.651943 0.896233
H 0.002196 -3.500794 0.347826

C	1.174340	-2.635111	1.915611
H	1.997198	-1.973575	1.629011
H	0.768066	-2.295842	2.871722
H	1.556909	-3.649257	2.032279
C	-3.758857	-1.655916	0.449435
O	-4.724822	-0.979607	0.879276
C	-3.729273	-3.146922	0.618775
H	-4.623582	-3.459275	1.158584
H	-3.703995	-3.644728	-0.355924
H	-2.841523	-3.456512	1.178769
H	-2.777083	-0.032457	-0.196313
H	-1.258892	1.104704	0.898095
H	-0.180384	-0.017126	2.748084
H	-4.232469	0.272180	2.141890
H	-5.118739	0.595314	0.005693
H	0.978325	-0.253440	-4.112145
H	0.207805	-3.866840	-1.786010
O	0.043654	-4.703973	-1.283892
H	0.917271	-5.110341	-1.195162
O	1.215972	0.443545	-4.773143
H	1.029975	0.039247	-5.631507
O	-1.922421	1.823330	0.884932
H	-2.586807	1.551830	1.551583
O	-0.256290	0.312946	3.667192
H	-0.963186	0.972385	3.623572
O	-3.908307	0.985376	2.729376
H	-4.569668	1.686222	2.639241
O	-5.318319	1.441347	-0.445231
H	-4.479389	1.704909	-0.848516

63

complexB_6sol_conf_23

Eopt -2358.106507

C	-2.658357	-1.574779	0.749208
C	-2.396015	-0.915731	-0.474922
C	-1.326480	-1.211133	-1.326010
C	-0.457162	-2.212713	-0.942635
C	-0.669365	-2.877340	0.275480
C	-1.736375	-2.565628	1.103811
H	-1.196083	-0.660321	-2.250861
H	0.016979	-3.656152	0.587425
H	-1.854005	-3.114033	2.029955
C	0.709512	-2.508742	-1.819469
O	1.040527	-1.801553	-2.756936
O	1.341201	-3.620760	-1.472005
C	2.520628	-3.955674	-2.223978
H	3.256263	-3.153354	-2.140011
H	2.260407	-4.127015	-3.270488
N	-3.712427	1.040048	-1.227966
N	-3.195582	0.130659	-0.862027
S	-4.066632	-1.141602	1.683949
C	-3.753800	-1.937897	3.279552
H	-3.807245	-3.024902	3.197508
H	-2.792448	-1.619211	3.688302
H	-4.556944	-1.588727	3.932800
H	2.898317	-4.869908	-1.769537
C	0.586459	1.926457	-1.807306
H	0.633397	0.839994	-1.946509
H	1.323036	2.370055	-2.485130
S	-1.079275	2.547537	-2.232520
C	1.066282	2.243805	-0.378137
H	1.045893	3.327302	-0.229714
C	0.179057	1.560400	0.665964
O	0.319588	0.344613	0.913297
N	2.442800	1.772901	-0.275537
N	-0.719957	2.330230	1.270225
H	-0.814357	3.292120	0.943242
C	-1.761111	1.836513	2.153149
H	-2.723398	1.823717	1.632771
H	-1.520420	0.829177	2.491098
H	-1.841962	2.493179	3.022233
C	3.280284	1.922444	0.760147
O	4.416248	1.391381	0.735331
C	2.828569	2.707876	1.955042
H	2.216602	2.058001	2.592394
H	3.703315	3.024173	2.524955
H	2.231127	3.581093	1.681943
H	2.760434	1.143085	-1.006262
H	1.702727	-0.849154	0.673388
H	0.363898	-0.417754	2.571756
H	4.439944	0.096608	2.096546
H	4.636938	-0.062909	-0.468440
H	-1.753517	1.129466	-3.794618
H	-1.261335	4.266567	-0.866344
O	-1.270493	4.922105	-0.123890
H	-2.204770	5.010070	0.112485
O	-2.098429	0.496889	-4.470913
H	-2.403099	1.063425	-5.193324
O	2.367322	-1.565262	0.737065
H	2.951600	-1.294553	1.476966
O	0.515199	-0.875771	3.423480
H	1.275618	-1.447328	3.247007
O	4.209172	-0.655436	2.677913
H	4.943519	-1.277567	2.578992
O	4.478964	-0.884451	-0.975140
H	3.673095	-1.238149	-0.541262

63

complexB_6sol_conf_24

Eopt -2358.113111

C	-2.803235	-1.697563	-0.456521
C	-1.661408	-1.654821	-1.293933
C	-1.092616	-0.485419	-1.809330
C	-1.700510	0.713211	-1.499022
C	-2.849481	0.714439	-0.690059
C	-3.384015	-0.454957	-0.175886
H	-0.200593	-0.536909	-2.424744
H	-3.324739	1.655932	-0.438205
H	-4.257722	-0.393423	0.460344
C	-1.061784	1.972288	-1.969358
O	0.101950	2.027204	-2.337573
O	-1.877252	3.012554	-1.939756

C	-1.300988	4.288597	-2.273788
H	-0.969865	4.285520	-3.314228
H	-0.462710	4.502623	-1.607220
N	-0.522230	-3.787705	-1.833967
N	-1.029287	-2.832345	-1.596663
S	-3.377149	-3.228084	0.141959
C	-4.779395	-2.768952	1.187810
H	-5.147448	-3.713366	1.595762
H	-4.458646	-2.123327	2.007674
H	-5.570162	-2.299547	0.599669
H	-2.102172	5.010826	-2.129536
C	1.858740	-0.833473	2.089533
H	1.436585	-0.559289	3.061948
H	2.844798	-1.265720	2.273939
S	0.772904	-2.059768	1.268937
C	2.059410	0.472870	1.299094
H	2.655613	1.145918	1.923678
C	2.895215	0.215017	0.047841
O	4.051737	-0.247158	0.192349
N	0.771647	1.084829	1.039059
N	2.384296	0.501194	-1.139994
H	1.445856	0.885342	-1.195602
C	3.087902	0.273243	-2.389622
H	3.106120	-0.791591	-2.639297
H	2.564817	0.815068	-3.178117
H	4.114338	0.642133	-2.321740
C	0.487997	2.395242	0.989685
O	-0.698729	2.766922	0.857263
C	1.605375	3.389940	1.107971
H	2.452126	3.133341	0.465635
H	1.230929	4.378889	0.840954
H	1.963129	3.414519	2.143787
H	-0.008329	0.436724	0.928586
H	4.814692	-1.477996	-0.847956
H	4.894144	-0.106081	1.786470
H	-1.251660	4.466855	0.990633
H	-1.927166	1.890992	1.931702
H	2.004300	-2.836503	-0.470114
H	-1.024728	-1.487663	2.338197
O	-1.784253	-1.198946	2.911007
H	-1.416945	-1.181298	3.806290
O	2.430702	-3.258367	-1.252245
H	3.356269	-2.939176	-1.247963
O	5.113939	-2.285596	-1.319812
H	5.172703	-2.016484	-2.247322
O	5.396375	-0.009882	2.624241
H	6.142247	0.561805	2.397655
O	-1.621911	5.371577	1.082541
H	-2.361873	5.269300	1.696130
O	-2.513858	1.483578	2.598859
H	-2.233817	0.543339	2.638131

63

complexB_6sol_conf_25

Eopt -2358.102388

C	2.911795	-0.193597	1.473215
C	2.927929	-0.115045	0.059674
C	2.427946	0.959131	-0.684222
C	1.884024	2.020411	0.010593
C	1.834003	1.974891	1.413587
C	2.328049	0.895390	2.128777
H	2.473097	0.943899	-1.767163
H	1.395193	2.801507	1.961032
H	2.264385	0.910186	3.209612
C	1.335575	3.164130	-0.770903
O	1.156491	3.126656	-1.977032
O	1.076097	4.221443	-0.015044
C	0.487417	5.357062	-0.672218
H	-0.468808	5.074418	-1.117472
H	1.166048	5.740938	-1.436591
N	3.848634	-2.015629	-1.236886
N	3.444980	-1.167742	-0.651749
S	3.611739	-1.578265	2.276048
C	2.881390	-1.487835	3.931058
H	1.793880	-1.404049	3.863804
H	3.142682	-2.433513	4.411801
H	3.304850	-0.663455	4.507434
H	0.339868	6.096225	0.113300
C	-0.608436	-0.403438	-2.255384
H	-0.211294	0.531711	-1.841276
H	-1.187405	-0.148106	-3.148176
S	0.760140	-1.539481	-2.690019
C	-1.579720	-1.019438	-1.244201
H	-1.965399	-1.954156	-1.653107
C	-0.881857	-1.280453	0.091216
O	-0.474665	-0.320584	0.781916
N	-2.706321	-0.119919	-1.015049
N	-0.761139	-2.541629	0.487951
H	-0.976192	-3.274444	-0.186828
C	-0.050476	-2.900904	1.702667
H	-0.484321	-2.390229	2.565251
H	-0.137393	-3.978293	1.845854
H	1.009133	-2.637037	1.625396
C	-3.968908	-0.479882	-0.743497
O	-4.803786	0.389060	-0.395239
C	-4.370356	-1.919382	-0.888684
H	-5.429827	-2.018539	-0.650703
H	-4.196759	-2.266715	-1.912338
H	-3.787963	-2.555290	-0.214776
H	-2.471488	0.853322	-0.820336
H	-0.787512	0.025966	2.538777
H	-0.986243	1.438345	0.573538
H	-6.074661	-0.095346	0.821263
H	-4.287657	1.545635	0.917806
H	1.967931	-0.131393	-3.860824
H	-0.195656	-3.506519	-2.234108
O	-0.679235	-4.316377	-1.932029
H	0.018135	-4.919706	-1.638821
O	2.513114	0.504202	-4.389312
H	2.020034	0.613945	-5.214560
O	-1.057626	0.376170	3.412080

```
H -1.651676 1.107485 3.190476
O -1.419323 2.305620 0.434470
H -2.253757 2.248714 0.946573
O -6.713363 -0.284229 1.539819
H -6.381326 0.224550 2.292035
O -3.937455 2.077572 1.664481
H -4.325265 2.955814 1.543834
63
complexB_6sol_conf_26
Eopt -2358.106053
C 3.181938 -0.221350 0.207237
C 2.966855 0.740500 -0.813034
C 2.129594 0.555479 -1.919749
C 1.467332 -0.652665 -2.026135
C 1.679679 -1.641889 -1.055481
C 2.517795 -1.437508 0.029975
H 2.004031 1.343892 -2.651285
H 1.172219 -2.595711 -1.143668
H 2.639570 -2.236949 0.749631
C 0.508645 -0.832673 -3.148971
O 0.421731 -0.061646 -4.090986
O -0.253875 -1.906072 -2.996973
C -1.239594 -2.150467 -4.015061
H -0.748920 -2.302037 -4.978876
H -1.935044 -1.310582 -4.068845
N 4.072631 2.950170 -0.639247
N 3.584513 1.959255 -0.713531
S 4.220035 0.151131 1.558975
C 3.869366 -1.197554 2.714668
H 4.239017 -2.152762 2.338418
H 4.411760 -0.938041 3.627027
H 2.799943 -1.242701 2.930996
H -1.755676 -3.056383 -3.702943
C -1.639422 1.552247 -1.117329
H -2.631694 1.972317 -1.306649
H -1.524473 0.720499 -1.821861
S -0.387311 2.846877 -1.459279
C -1.604839 0.977927 0.324353
H -1.007688 1.632033 0.961917
C -3.028204 0.924456 0.892449
O -3.732497 -0.106755 0.817358
N -1.008282 -0.345320 0.331334
N -3.469909 2.053683 1.434742
H -2.870466 2.883050 1.382738
C -4.837803 2.204519 1.900078
H -4.958058 3.219072 2.280578
H -5.550164 2.040246 1.085734
H -5.051718 1.495227 2.704084
C -0.468730 -0.978252 1.382138
O -0.268304 -2.214698 1.321644
C -0.086802 -0.188253 2.597050
H 0.311512 -0.864612 3.353832
H 0.674831 0.554144 2.334126
H -0.951401 0.343221 3.008310
H -1.253905 -0.945241 -0.450997
H -3.588017 -1.259680 -0.650882
H -3.369018 -1.711868 1.699537
H -1.041988 -3.215612 0.017910
H 0.763076 -3.103302 2.502563
H -1.297643 4.016182 0.162151
H 0.906995 2.845675 0.293817
O 1.506830 2.925854 1.080475
H 0.914607 2.820836 1.838460
O -1.751673 4.400779 0.958187
H -2.408626 5.009800 0.591829
O -3.540884 -1.870335 -1.412768
H -2.843700 -2.515064 -1.168671
O -3.215932 -2.548098 2.181574
H -2.286908 -2.756157 1.996861
O -1.548637 -3.749100 -0.632883
H -2.100879 -4.327715 -0.088356
O 1.326758 -3.655317 3.086293
H 0.719176 -4.298417 3.475821
63
complexB_6sol_conf_27
Eopt -2358.103943
C 3.409218 -0.132375 0.686543
C 3.066068 -0.288366 -0.679005
C 2.389740 0.666805 -1.445114
C 2.038389 1.851909 -0.831353
C 2.352450 2.043971 0.524613
C 3.015239 1.079493 1.266641
H 2.162070 0.466323 -2.485839
H 2.067278 2.966926 1.016721
H 3.225732 1.277164 2.310427
C 1.335402 2.882038 -1.645997
O 0.873336 2.660334 -2.753204
O 1.290113 4.062726 -1.046634
C 0.608635 5.117772 -1.747168
H -0.421929 4.825022 -1.955981
H 1.135970 5.347093 -2.675442
N 3.730968 -2.385476 -1.818116
N 3.426675 -1.448217 -1.314830
S 4.261404 -1.409421 1.511822
C 4.360718 -0.813019 3.216085
H 3.364315 -0.661688 3.637264
H 4.864432 -1.613301 3.763594
H 4.959752 0.097157 3.279987
H 0.634235 5.971575 -1.072418
C -0.867200 -0.694188 -1.828484
H -0.368908 0.279052 -1.740609
H -1.640493 -0.588114 -2.595635
S 0.320664 -1.988401 -2.348623
C -1.593088 -1.005638 -0.509207
H -1.893315 -2.053403 -0.511111
C -0.714904 -0.719901 0.711497
O -0.526807 0.460598 1.079655
N -2.799256 -0.194068 -0.417022
N -0.195402 -1.758294 1.353635
H -0.344807 -2.693366 0.969296
```

C 0.612495 -1.610062 2.551280
H 1.305153 -0.775116 2.440699
H -0.019590 -1.420203 3.424530
H 1.173450 -2.531375 2.709581
C -3.955566 -0.547619 0.159642
O -4.887701 0.285902 0.250273
C -4.111841 -1.953008 0.665280
H -5.099594 -2.068336 1.111603
H -4.004893 -2.669031 -0.157010
H -3.347286 -2.182266 1.413948
H -2.725287 0.774537 -0.715406
H -1.086540 2.057449 0.394568
H 0.029383 1.275089 2.600032
H -4.445839 2.019207 0.483549
H -6.295986 -0.022836 1.344715
H -0.112128 -3.611746 -0.912223
H -1.530593 -3.155441 -2.805937
O -2.414678 -3.582342 -2.931580
H -2.790861 -3.611293 -2.039330
O -0.347589 -4.249106 -0.189204
H -1.286963 -4.433628 -0.334467
O -1.396062 2.942504 0.110260
H -2.358965 2.944051 0.295302
O 0.262543 1.810764 3.386864
H -0.522392 2.348778 3.554713
O -4.167000 2.946949 0.642630
H -4.201495 3.052708 1.603270
O -7.043631 -0.154267 1.966226
H -7.692624 0.517182 1.716956
63
complexB_6sol_conf_28
Eopt -2358.099084
C 3.328482 0.459939 0.556443
C 2.707704 -0.039485 -0.613341
C 1.792928 0.674067 -1.392400
C 1.473539 1.957789 -0.996342
C 2.044455 2.480501 0.174053
C 2.943998 1.750333 0.934604
H 1.350271 0.227959 -2.274363
H 1.785050 3.482256 0.497643
H 3.361542 2.203229 1.824421
C 0.500875 2.724601 -1.824631
O -0.157800 2.223486 -2.721123
O 0.445875 4.005571 -1.491048
C -0.494591 4.821602 -2.211362
H -1.509538 4.457496 -2.038563
H -0.260307 4.809925 -3.277641
N 3.151655 -2.370523 -1.333619
N 2.970754 -1.332219 -0.999598
S 4.504828 -0.511603 1.406959
C 4.544165 0.276130 3.037405
H 5.124773 -0.399411 3.669737
H 5.040512 1.247465 2.999730
H 3.530465 0.369838 3.434634
H -0.373219 5.823872 -1.804151
C -1.324274 -1.179342 -1.439637
H -0.921268 -0.163849 -1.534548
H -2.270029 -1.200358 -1.990847
S -0.167669 -2.410299 -2.145557
C -1.679074 -1.419892 0.039526
H -2.005318 -2.452291 0.174630
C -0.502112 -1.133945 0.970139
O -0.178497 0.048278 1.216552
N -2.760670 -0.538103 0.459361
N 0.115609 -2.189007 1.490060
H -0.180449 -3.116205 1.182039
C 1.291855 -2.112837 2.335560
H 1.682922 -1.096855 2.333939
H 1.043922 -2.393619 3.363523
H 2.056002 -2.794596 1.955854
C -4.074289 -0.784901 0.382471
O -4.894431 0.115141 0.682961
C -4.533832 -2.152489 -0.031306
H -5.620596 -2.156874 -0.118605
H -4.092125 -2.446377 -0.988126
H -4.232605 -2.890936 0.719554
H -2.498807 0.414279 0.685256
H 0.563581 0.838643 2.730030
H -0.864041 1.837807 1.020591
H -6.682081 -0.141282 0.586986
H -4.294126 1.698333 1.363052
H 1.373959 -1.652648 -3.644464
H -0.368961 -4.060673 -0.729437
O -0.477147 -4.705210 0.016942
H 0.380062 -5.147437 0.093237
O 2.183991 -1.460511 -4.169550
H 2.254568 -2.202181 -4.786863
O 0.829127 1.330764 3.532855
H 0.005918 1.730078 3.845621
O -1.239391 2.729013 0.895582
H -2.170144 2.656038 1.195038
O -7.657862 -0.243224 0.557594
H -8.002280 0.620615 0.821421
O -3.925893 2.518373 1.759923
H -4.486366 3.231266 1.422226
63
complexB_6sol_conf_29
Eopt -2358.103346
C -3.426687 0.423697 -0.011818
C -2.515697 0.203968 1.051702
C -1.466522 1.068388 1.394039
C -1.286188 2.200459 0.628901
C -2.159822 2.446071 -0.446648
C -3.209271 1.594741 -0.750416
H -0.817528 0.828181 2.229261
H -2.019136 3.330385 -1.058838
H -3.858288 1.843417 -1.580473
C -0.137668 3.092509 0.951223
O 0.542712 2.975555 1.957076
O 0.056625 4.023211 0.026268

C 1.121205 4.962002 0.259755
H 1.116859 5.619283 -0.608446
H 2.076357 4.438342 0.338163
N -2.593266 -1.884524 2.375628
N -2.604103 -0.950885 1.778991
S -4.692301 -0.732682 -0.308610
C -5.592000 -0.038796 -1.716780
H -6.062831 0.910697 -1.453972
H -4.930235 0.067699 -2.579293
H -6.369247 -0.772856 -1.943881
H 0.922607 5.531052 1.170816
C 2.009478 -0.341349 1.725984
H 1.315871 0.354514 2.205947
H 3.019981 -0.013849 1.987619
S 1.698334 -2.042711 2.342832
C 1.860056 -0.146877 0.205410
H 1.861474 0.931484 0.018762
C 3.078907 -0.699322 -0.533185
O 4.136054 -0.027450 -0.528242
N 0.603590 -0.705161 -0.248534
N 2.986169 -1.870761 -1.149741
H 2.157475 -2.443950 -0.992733
C 4.129195 -2.481134 -1.810008
H 4.903944 -2.757269 -1.086994
H 3.786266 -3.377168 -2.328269
H 4.557027 -1.789073 -2.538906
C -0.064247 -0.361878 -1.358579
O -1.127069 -0.955123 -1.660578
C 0.487878 0.746433 -2.207599
H 0.564322 1.672774 -1.628526
H 1.492711 0.496546 -2.565800
H -0.166664 0.910447 -3.063845
H 0.215188 -1.454142 0.323720
H 4.087832 1.757330 -0.324669
H 5.821044 0.021012 -1.175363
H -1.507045 -2.506224 -0.923457
H -2.145108 -0.328173 -3.004567
H 1.304084 -3.498099 0.614856
H 0.107627 -1.255087 3.680595
O -0.600871 -0.904384 4.276228
H -0.655551 -1.556700 4.988644
O 1.089527 -4.011453 -0.199163
H 0.140399 -3.814332 -0.358089
O 3.994146 2.732960 -0.260498
H 4.775646 3.085761 -0.707554
O 6.737463 0.172949 -1.485812
H 7.250504 0.295606 -0.675397
O -1.642073 -3.404011 -0.539176
H -2.020200 -3.927505 -1.259648
O -2.727752 -0.068309 -3.753223
H -2.410443 -0.599531 -4.496574
63
complexB_6sol_conf_3 Eopt
-2358.100430
C -3.394774 -0.064477 -1.106169
C -2.834794 -0.504066 0.113919
C -2.099673 0.311656 0.990594
C -1.883982 1.624513 0.627307
C -2.395296 2.091844 -0.594108
C -3.130423 1.274057 -1.431709
H -1.715674 -0.102815 1.914704
H -2.228025 3.123029 -0.883523
H -3.532263 1.681885 -2.353350
C -1.095207 2.542514 1.495503
O -0.822111 3.693168 1.190596
O -0.720152 1.972766 2.628660
C 0.097992 2.751135 3.518584
H -0.448280 3.637120 3.848356
H 1.024953 3.037973 3.018218
N -2.890360 -2.852817 0.892367
N -2.922695 -1.815979 0.507636
S -4.323583 -0.976074 -2.272813
C -5.159759 -2.299144 -1.352310
H -6.021989 -2.561154 -1.970953
H -4.536337 -3.187082 -1.240111
H -5.528403 -1.936409 -0.390145
H 0.306066 2.093736 4.360874
C 1.378476 -0.866184 2.107615
H 0.731600 -0.094073 2.535107
H 2.060778 -1.188392 2.900877
S 0.352014 -2.297103 1.575968
C 2.225355 -0.172930 1.034420
H 2.718043 0.669194 1.524818
C 3.367704 -0.988189 0.408637
O 4.334000 -0.375826 -0.103716
N 1.392951 0.355495 -0.038063
N 3.285420 -2.310514 0.417517
H 2.426097 -2.724888 0.789574
C 4.292701 -3.163017 -0.188827
H 4.004230 -4.200988 -0.022419
H 4.362308 -2.978562 -1.264709
H 5.270968 -2.987074 0.266403
C 1.445244 1.580265 -0.581836
O 0.669818 1.879213 -1.519463
C 2.413556 2.589555 -0.032922
H 3.434005 2.199991 -0.018029
H 2.379188 3.486822 -0.650705
H 2.135750 2.851451 0.994063
H 0.672847 -0.266823 -0.395729
H 5.799594 -0.917866 -1.078965
H 3.995394 0.516447 -1.718879
H 0.222291 3.683653 -1.661092
H -0.128649 0.601142 -2.474423
H -0.315383 -2.314036 -0.616670
H -1.077520 -2.016967 3.247143
O -1.725288 -1.806251 3.962490
H -1.331997 -2.176102 4.764905
O -0.690076 -2.462684 -1.512507
H -0.625139 -1.596967 -1.970695
O 6.596835 -1.039720 -1.635085

H 6.560720 -0.306273 -2.264442
O 3.893536 0.898042 -2.612533
H 4.413653 0.312881 -3.180212
O -0.101469 4.594302 -1.505144
H -0.432762 4.538801 -0.592980
O -0.518053 -0.107150 -3.034830
H 0.192801 -0.352189 -3.643610
63
complexB_6sol_conf_30
Eopt -2358.102452
C -3.571177 0.253496 0.022303
C -2.612437 -0.050724 1.021514
C -1.578106 0.804576 1.421344
C -1.464568 2.020805 0.780024
C -2.393507 2.359564 -0.219142
C -3.428554 1.511385 -0.577307
H -0.892726 0.495703 2.202478
H -2.308722 3.314395 -0.725173
H -4.119494 1.829911 -1.346779
C -0.329360 2.910298 1.150781
O 0.400725 2.705096 2.105703
O -0.185412 3.927412 0.312172
C 0.934662 4.801276 0.540440
H 0.819790 5.311633 1.498882
H 0.909079 5.516628 -0.279633
N -2.685219 -2.218186 2.217386
N -2.676937 -1.259141 1.664411
S -4.803749 -0.910869 -0.374196
C -5.816399 -0.055409 -1.604495
H -6.599984 -0.768004 -1.871564
H -6.274476 0.838556 -1.177002
H -5.225056 0.185579 -2.489187
H 1.863528 4.228125 0.521518
C 2.085138 -0.362113 1.656276
H 1.395804 0.307721 2.175890
H 3.094275 -0.099440 1.985997
S 1.703817 -2.099511 2.107217
C 1.996564 -0.023869 0.154006
H 2.065540 1.065024 0.068006
C 3.210144 -0.571458 -0.591017
O 4.280181 0.074118 -0.537248
N 0.728059 -0.468112 -0.384226
N 3.098196 -1.712251 -1.263286
H 2.275273 -2.289765 -1.107077
C 4.243230 -2.338464 -1.903588
H 3.883688 -3.168846 -2.511540
H 4.751410 -1.619573 -2.548861
H 4.954575 -2.715148 -1.161789
C 0.079251 0.046376 -1.438476
O -1.000774 -0.455106 -1.819750
C 0.670019 1.232041 -2.142909
H 1.672977 1.001127 -2.517655
H 0.025340 1.506569 -2.978416
H 0.759063 2.083772 -1.461073
H 0.293078 -1.257432 0.091118
H 6.028368 -0.088411 -0.959871
H 4.411209 1.830723 -0.227154
H -2.236821 0.089049 -3.011053
H -1.478163 -2.118816 -1.249906
H 1.217201 -3.368420 0.363226
H 0.095720 -1.550968 3.522126
O -0.626335 -1.306378 4.153408
H -0.626849 -2.022503 4.802980
O 1.009047 -3.828028 -0.490197
H 1.232681 -4.757339 -0.340195
O 6.987962 -0.057297 -1.152435
H 7.405053 0.088270 -0.292941
O 4.558551 2.792355 -0.098991
H 5.448363 2.948819 -0.442379
O -2.948749 0.289157 -3.654087
H -2.611794 -0.041804 -4.497745
O -1.636804 -2.998934 -0.850419
H -0.734243 -3.371062 -0.767333
63
complexB_6sol_conf_31
Eopt -2358.103238
C -3.269800 -0.228151 -0.910065
C -2.825238 -0.692406 0.352223
C -2.340559 0.125751 1.379018
C -2.271650 1.482887 1.138075
C -2.668129 1.981059 -0.114281
C -3.153839 1.151608 -1.112492
H -2.009173 -0.317500 2.313259
H -2.599807 3.044520 -0.314044
H -3.458761 1.591475 -2.053870
C -1.733481 2.361740 2.213333
O -1.281010 1.936717 3.263744
O -1.804346 3.648607 1.904704
C -1.261821 4.573902 2.862811
H -1.801632 4.493355 3.808449
H -1.407316 5.558650 2.422057
N -2.749715 -3.127122 0.804799
N -2.813149 -2.042127 0.595314
S -3.936041 -1.351840 -2.064753
C -3.933592 -0.400764 -3.604947
H -4.676974 0.397924 -3.579544
H -2.936536 -0.004192 -3.807385
H -4.203041 -1.118168 -4.383563
H -0.198636 4.373311 3.010677
C 1.088807 -0.634685 1.947404
H 0.426346 0.235990 1.876967
H 1.870010 -0.385013 2.672683
S 0.160322 -2.109501 2.518486
C 1.782047 -0.838888 0.595668
H 2.369290 -1.756648 0.632635
C 0.764870 -0.908275 -0.546516
O 0.110745 0.112321 -0.850419
N 2.691030 0.271459 0.331984
N 0.652607 -2.066351 -1.188580
H 1.124477 -2.875207 -0.782321

C -0.313797 -2.321656 -2.241047
H -0.668641 -1.377994 -2.655439
H 0.162183 -2.902479 -3.034138
H -1.163902 -2.889370 -1.851589
C 3.882145 0.203133 -0.276697
O 4.491148 1.259035 -0.574728
C 4.479176 -1.143322 -0.569066
H 3.834280 -1.713595 -1.245055
H 5.455675 -1.005749 -1.034293
H 4.596948 -1.722296 0.353302
H 2.310130 1.206450 0.461851
H 0.359114 1.859079 -0.258597
H -0.132421 0.932592 -2.453235
H 5.406488 1.261143 -2.146837
H 3.471645 2.599015 -1.268964
H 2.242704 -2.756280 3.124425
H 1.022587 -3.710192 1.210805
O 1.435622 -4.330094 0.557627
H 0.722510 -4.936108 0.311582
O 3.207266 -2.884137 3.291388
H 3.634602 -2.435971 2.547149
O 0.646779 2.764232 -0.023968
H 1.374209 2.958227 -0.652625
O -0.166617 1.524779 -3.231822
H 0.320612 2.308664 -2.941173
O 5.828909 1.334051 -3.028323
H 5.267878 1.958687 -3.508006
O 2.867867 3.244088 -1.697125
H 3.189511 4.109742 -1.408676
63
complexB_6sol_conf_32
Eopt -2358.103302
C 3.262881 0.198409 -0.926632
C 2.822547 0.684591 0.328846
C 2.337134 -0.114962 1.369648
C 2.262074 -1.475453 1.150524
C 2.650506 -1.994812 -0.095704
C 3.137484 -1.183303 -1.108203
H 2.010636 0.344450 2.297808
H 2.574677 -3.060674 -0.279185
H 3.435620 -1.638715 -2.044404
C 1.725644 -2.334512 2.242429
O 1.274974 -1.890145 3.285584
O 1.797189 -3.626935 1.958217
C 1.255350 -4.533877 2.934028
H 1.797510 -4.437080 3.876800
H 1.398181 -5.526672 2.510851
N 2.757053 3.125841 0.745504
N 2.817051 2.037875 0.551142
S 3.936318 1.300758 -2.097882
C 3.937136 0.325301 -3.622673
H 4.226659 1.026631 -4.408692
H 4.668965 -0.482995 -3.576493
H 2.937014 -0.059697 -3.832547
H 0.192885 -4.328835 3.080815
C -1.091558 0.684339 1.952594
H -0.439093 -0.195366 1.903588
H -1.873777 0.463399 2.685983
S -0.143605 2.161359 2.483783
C -1.785630 0.861187 0.597250
H -2.364782 1.785051 0.614143
C -0.769225 0.892621 -0.547355
O -0.126349 -0.141957 -0.826799
N -2.704650 -0.248144 0.365183
N -0.644984 2.033955 -1.216013
H -1.111663 2.855619 -0.830430
C 0.322516 2.256622 -2.274819
H -0.158245 2.789021 -3.098634
H 1.158406 2.859075 -1.907527
H 0.697198 1.300618 -2.640218
C -3.875044 -0.192130 -0.284007
O -4.492703 -1.252033 -0.547395
C -4.439311 1.146948 -0.662067
H -4.583948 1.769865 0.227135
H -3.759996 1.676194 -1.337469
H -5.399166 1.000958 -1.158278
H -2.337997 -1.181525 0.541585
H 0.115412 -0.996216 -2.403887
H -0.392120 -1.864937 -0.187423
H -3.482865 -2.608783 -1.216045
H -5.424833 -1.313632 -2.102061
H -0.987250 3.743340 1.136134
H -2.190366 2.885493 3.077863
O -3.146620 3.067800 3.244329
H -3.586255 2.754520 2.440666
O -1.395110 4.352420 0.470124
H -0.675481 4.943272 0.206742
O 0.156900 -1.597696 -3.175068
H -0.362171 -2.364963 -2.895901
O -0.689150 -2.760841 0.070131
H -1.411096 -2.966568 -0.561167
O -2.881354 -3.265337 -1.629346
H -3.212001 -4.123505 -1.328863
O -5.863539 -1.428793 -2.971403
H -5.303669 -2.065582 -3.436155
63
complexB_6sol_conf_33
Eopt -2358.102449
C 2.900896 -0.253035 1.454338
C 2.923931 -0.156943 0.042327
C 2.445959 0.934779 -0.690901
C 1.915545 1.995328 0.015551
C 1.858418 1.933142 1.417573
C 2.332304 0.836972 2.120671
H 2.501187 0.936326 -1.774442
H 1.430588 2.759541 1.973929
H 2.265917 0.839040 3.201458
C 1.391992 3.159020 -0.753387
O 1.199080 3.133617 -1.957647
O 1.173788 4.219266 0.011056

C	0.611761	5.375567	-0.633178
H	0.482616	6.109576	0.160360
H	-0.351114	5.121155	-1.081023
N	3.807422	-2.063397	-1.270009
N	3.424934	-1.210490	-0.678870
S	3.577134	-1.657687	2.242027
C	2.834714	-1.584013	3.893191
H	1.748801	-1.496682	3.817236
H	3.090375	-2.536056	4.364135
H	3.254778	-0.767656	4.482906
H	1.299470	5.750754	-1.393832
C	-0.617746	-0.388263	-2.258926
H	-0.197206	0.535179	-1.841774
H	-1.188944	-0.115927	-3.151827
S	0.723904	-1.556869	-2.697058
C	-1.605241	-0.986183	-1.252811
H	-2.010648	-1.909359	-1.667383
C	-0.917186	-1.272750	0.081842
O	-0.488968	-0.328630	0.780726
N	-2.714458	-0.066626	-1.020485
N	-0.825304	-2.540500	0.466696
H	-1.049182	-3.261654	-0.218316
C	-0.118681	-2.925325	1.676055
H	0.951406	-2.710496	1.587558
H	-0.520248	-2.389263	2.538793
H	-0.254663	-3.996447	1.827495
C	-3.982505	-0.408522	-0.751272
O	-4.805932	0.470693	-0.400556
C	-4.404408	-1.842080	-0.899538
H	-5.466307	-1.925379	-0.666474
H	-4.231252	-2.192454	-1.922100
H	-3.834494	-2.485608	-0.222245
H	-2.464745	0.902777	-0.825910
H	-0.775048	0.032548	2.529135
H	-0.958404	1.457314	0.576764
H	-6.055051	-0.048278	0.834902
H	-4.266785	1.619897	0.901457
H	2.011378	-0.123971	-3.734570
H	-0.291930	-3.498033	-2.265653
O	-0.808088	-4.287390	-1.964088
H	-0.135709	-4.925741	-1.687440
O	2.594216	0.524640	-4.204233
H	2.053708	0.849417	-4.937859
O	-0.998882	0.391795	3.412217
H	-1.504742	1.192214	3.213588
O	-1.377126	2.331047	0.438047
H	-2.218765	2.285296	0.939214
O	-6.667711	-0.261710	1.568861
H	-6.394580	0.328820	2.284155
O	-3.910198	2.147428	1.648319
H	-4.285107	3.030933	1.526120

63

complexB_6sol_conf_34

Eopt -2358.096176

C	4.371254	0.630788	0.116538
C	3.495325	0.034177	1.050202
C	2.236791	0.542677	1.393502
C	1.838668	1.727747	0.807111
C	2.702089	2.383408	-0.082231
C	3.936162	1.845207	-0.419893
H	1.608921	0.016527	2.103762
H	2.395060	3.315488	-0.543567
H	4.561636	2.356565	-1.143601
C	0.467318	2.221910	1.118617
O	-0.261695	1.684555	1.934402
O	0.127055	3.280256	0.399551
C	-1.222422	3.758020	0.550637
H	-1.382108	4.109320	1.572124
H	-1.929757	2.962052	0.306745
N	4.128316	-2.014537	2.293717
N	3.872336	-1.102431	1.721888
S	5.947114	0.020861	-0.363314
C	5.681054	-1.766658	-0.601542
H	6.363370	-2.059152	-1.402780
H	5.923422	-2.343505	0.292019
H	4.657869	-1.957978	-0.933770
H	-1.314452	4.581265	-0.155620
C	-0.680523	-2.257141	-0.758491
H	-0.922545	-2.421165	-1.814977
H	-1.087797	-3.101888	-0.196679
S	1.137726	-2.228494	-0.512124
C	-1.434811	-1.000767	-0.261472
H	-1.004556	-0.692507	0.695514
C	-1.313664	0.189046	-1.225429
O	-2.319161	0.757338	-1.703148
N	-2.832108	-1.335340	-0.059814
N	-0.078773	0.576068	-1.526165
H	0.688298	-0.001816	-1.175867
C	0.199246	1.646428	-2.466308
H	-0.048702	1.345985	-3.489813
H	-0.378906	2.537339	-2.209569
H	1.262616	1.882983	-2.415378
C	-3.619626	-0.954556	0.953543
O	-4.827081	-1.295477	0.967779
C	-3.030921	-0.141469	2.068236
H	-3.817737	0.114735	2.778212
H	-2.248588	-0.707290	2.585537
H	-2.579538	0.775301	1.678951
H	-3.296223	-1.828088	-0.818082
H	-3.797052	1.382966	-0.849331
H	-3.520784	-0.251151	-2.692058
H	-5.992630	-0.474017	2.055881
H	-5.741629	-1.324081	-0.569170
H	0.820972	-2.183822	1.679588
H	2.331383	-1.214560	-2.127566
O	2.908500	-0.739244	-2.769809
H	2.646541	-1.085049	-3.633890
O	0.681433	-2.164359	2.658503
H	0.656753	-3.095355	2.918959
O	-4.549168	1.750501	-0.344248

H -5.324104 1.259182 -0.648508
O -4.153484 -0.782345 -3.212731
H -4.899898 -0.940640 -2.595150
O -6.668552 -0.039251 2.620422
H -6.337911 0.861320 2.741968
O -6.247676 -1.248175 -1.407960
H -6.554768 -2.146439 -1.594449
63
complexB_6sol_conf_35
Eopt -2358.106041
C 2.339327 -0.706622 1.434193
C 3.114194 -0.741130 0.248891
C 3.523831 0.386754 -0.469908
C 3.155972 1.625797 0.014455
C 2.396773 1.704936 1.194774
C 1.995485 0.573660 1.884996
H 4.105600 0.273335 -1.378015
H 2.108399 2.675283 1.581732
H 1.406096 0.693569 2.785170
C 3.571830 2.834387 -0.750551
O 4.232386 2.786273 -1.775210
O 3.140548 3.961150 -0.199982
C 3.479871 5.184796 -0.876381
H 3.058605 5.182821 -1.883631
H 4.564772 5.301208 -0.915925
N 3.824934 -2.944338 -0.635290
N 3.495028 -1.960656 -0.250572
S 1.901220 -2.193662 2.225937
C 0.825152 -1.651480 3.574755
H 1.375469 -1.047490 4.298764
H -0.038926 -1.115119 3.177584
H 0.490888 -2.573284 4.057023
H 3.032131 5.975181 -0.276441
C 0.057421 -0.109482 -1.656676
H 0.597714 0.335153 -0.812337
H 0.054524 0.627751 -2.465262
S 0.897765 -1.631127 -2.236551
C -1.405901 -0.329757 -1.245585
H -1.903649 -0.891370 -2.035154
C -1.491413 -1.089440 0.078346
O -1.220640 -0.511971 1.156270
N -2.096971 0.947790 -1.103832
N -1.880562 -2.357239 0.036933
H -1.992789 -2.797183 -0.878140
C -1.906461 -3.193215 1.224272
H -2.577587 -2.770357 1.976930
H -2.268323 -4.181345 0.938425
H -0.905942 -3.290315 1.657414
C -3.396869 1.161411 -1.354603
O -3.924072 2.261034 -1.060543
C -4.205750 0.079092 -2.011516
H -4.224132 -0.820560 -1.388280
H -5.225382 0.437892 -2.154785
H -3.779878 -0.191430 -2.983132
H -1.623939 1.664388 -0.552488
H -0.867003 1.280959 1.374905
H -2.849212 -0.005280 1.958141
H -5.624351 2.126191 -0.338201
H -3.659219 2.932309 0.579096
H -0.898387 -2.886579 -2.736361
H 0.682314 -3.541503 -1.034700
O 0.709475 -4.479246 -0.729070
H -0.220373 -4.740229 -0.671948
O -1.755017 -3.385256 -2.757724
H -1.498437 -4.311177 -2.641347
O -0.803764 2.258668 1.386629
H -1.701775 2.561287 1.610913
O -3.692049 0.365415 2.280068
H -3.646147 1.310804 2.031832
O -6.438876 2.059359 0.200858
H -6.108976 1.886267 1.093911
O -3.500433 3.119010 1.529892
H -4.344092 3.442812 1.877193
63
complexB_6sol_conf_36
Eopt -2358.112517
C -3.859249 0.036612 0.268918
C -2.674084 0.014165 1.044522
C -1.639366 0.952911 0.950354
C -1.758848 1.946348 0.000121
C -2.900673 1.980442 -0.817028
C -3.931032 1.065166 -0.679243
H -0.779255 0.869452 1.602768
H -2.986339 2.754980 -1.572309
H -4.795769 1.151068 -1.325020
C -0.684222 2.960107 -0.196634
O -0.629880 3.699215 -1.165301
O 0.197658 2.961102 0.791047
C 1.318688 3.853840 0.678255
H 1.922971 3.663837 1.563914
H 0.970821 4.888940 0.668190
N -2.291245 -1.811153 2.675470
N -2.489967 -0.996890 1.952283
S -5.092537 -1.160783 0.547588
C -6.325625 -0.798724 -0.725474
H -7.099691 -1.557963 -0.590690
H -6.764036 0.190337 -0.579373
H -5.895631 -0.896916 -1.724200
H 1.885634 3.634419 -0.228520
C 1.985414 0.162941 1.299837
H 1.383314 1.023724 1.600972
H 2.943363 0.249333 1.818858
S 1.157179 -1.395809 1.800076
C 2.269532 0.320515 -0.206324
H 2.622634 1.346000 -0.355039
C 3.432252 -0.577967 -0.618279
O 4.576934 -0.304591 -0.178440
N 1.061858 0.129311 -0.982266
N 3.212005 -1.614749 -1.410409
H 2.268292 -1.820331 -1.748045

C 4.282828 -2.513500 -1.806555
H 5.057586 -1.974021 -2.358873
H 4.737549 -2.985686 -0.930893
H 3.858305 -3.285335 -2.448860
C 0.776074 0.669733 -2.178895
O -0.302972 0.404316 -2.750253
C 1.774834 1.601367 -2.804643
H 2.755542 1.124605 -2.900102
H 1.417171 1.894474 -3.792137
H 1.897948 2.498692 -2.189724
H 0.380793 -0.523432 -0.601814
H 4.796205 1.294482 0.661807
H 5.062995 -1.222676 1.352235
H -1.555889 -0.758210 -2.238618
H 0.337075 -1.957692 -2.999458
H -0.218480 -2.423655 0.344504
H 0.157250 -0.381749 3.476153
O -0.313137 0.091620 4.208510
H 0.096074 -0.248744 5.016102
O -0.762800 -2.979951 -0.260754
H -1.384213 -2.361000 -0.702639
O 4.913396 2.156726 1.116745
H 4.009089 2.477116 1.244612
O 5.342817 -1.702366 2.156675
H 6.222370 -2.041805 1.941729
O -2.232904 -1.450996 -2.067434
H -2.069278 -2.113698 -2.753659
O 0.787006 -2.714215 -2.593682
H 0.274693 -2.879295 -1.770537
63
complexB_6sol_conf_37
Eopt -2358.098115
C 2.895508 -0.206050 -1.453374
C 2.734254 -0.438108 -0.065261
C 1.930089 -1.441730 0.485965
C 1.216140 -2.244565 -0.379026
C 1.332516 -2.034867 -1.763323
C 2.158533 -1.053857 -2.288740
H 1.876071 -1.565095 1.561292
H 0.771555 -2.658228 -2.450494
H 2.219761 -0.946315 -3.364256
C 0.344102 -3.300801 0.205819
O 0.291943 -3.540539 1.400623
O -0.361232 -3.949208 -0.711402
C -1.212611 -5.010862 -0.241728
H -0.610331 -5.779509 0.246786
H -1.690584 -5.406345 -1.136201
N 3.878812 0.973110 1.613483
N 3.398667 0.354810 0.831290
S 3.968193 1.052438 -1.997782
C 3.799405 0.998351 -3.795821
H 4.140534 0.041020 -4.194614
H 2.771650 1.205822 -4.099943
H 4.451686 1.793863 -4.163946
H -1.957199 -4.611933 0.448915
C -0.651603 0.022638 2.432717
H -0.373513 -0.870890 1.860942
H -1.348724 -0.300926 3.211832
S 0.825334 0.774776 3.206323
C -1.452193 0.951030 1.498377
H -1.775278 1.827671 2.064159
C -0.634589 1.388646 0.276062
O -0.543729 0.637836 -0.717407
N -2.628921 0.216812 1.052700
N -0.084615 2.598581 0.324892
H -0.147823 3.120644 1.200681
C 0.818833 3.092754 -0.696666
H 1.827431 2.688414 -0.560825
H 0.455480 2.819266 -1.688758
H 0.863850 4.180495 -0.623025
C -3.784163 0.730096 0.612489
O -4.661109 -0.028360 0.135380
C -4.015807 2.209459 0.721934
H -3.214633 2.776777 0.240015
H -4.967729 2.457972 0.251294
H -4.048132 2.503604 1.776829
H -2.506803 -0.779890 0.900359
H -2.170283 1.392348 -1.796454
H 0.034197 0.683691 -2.643172
H -5.207602 0.596858 -1.502938
H -4.425960 -1.826750 -0.018111
H 2.332977 -0.770681 3.795486
H 0.224134 2.888390 3.218183
O -0.102295 3.807543 3.033956
H 0.700071 4.344399 2.966676
O 3.025759 -1.430028 4.035405
H 3.332069 -1.153939 4.910025
O -2.600067 1.822819 -2.555205
H -3.524923 1.494718 -2.545050
O -0.176568 0.765204 -3.590995
H -1.095022 1.092566 -3.564191
O -5.289254 0.950845 -2.412699
H -5.747480 1.795823 -2.302258
O -4.299195 -2.795098 -0.100537
H -4.977988 -3.173390 0.474056
63
complexB_6sol_conf_38
Eopt -2358.106553
C 2.704948 -1.215056 0.785844
C 2.632884 -1.244215 -0.629039
C 2.456613 -0.117352 -1.442377
C 2.322690 1.106066 -0.819981
C 2.386975 1.180050 0.581757
C 2.584692 0.054094 1.364781
H 2.409983 -0.224017 -2.520025
H 2.287754 2.142080 1.071507
H 2.636976 0.169003 2.439574
C 2.075861 2.303447 -1.670700
O 2.429559 2.385534 -2.835668
O 1.406266 3.244738 -1.025321

C	1.033712	4.413856	-1.774390
H	0.496945	5.044328	-1.068073
H	0.383443	4.126650	-2.603452
N	2.799777	-3.426943	-1.788087
N	2.728744	-2.452041	-1.268977
S	2.933420	-2.698452	1.671413
C	2.974340	-2.157691	3.397645
H	2.022667	-1.710121	3.689746
H	3.131274	-3.072675	3.973351
H	3.808127	-1.475914	3.574909
H	1.925053	4.924204	-2.145191
C	-0.960167	-1.993112	-0.801114
H	-1.601300	-2.862144	-0.626972
H	-0.123761	-2.085597	-0.097507
S	-0.357718	-2.051393	-2.532650
C	-1.750979	-0.708509	-0.453601
H	-2.098214	-0.245758	-1.378467
C	-2.984976	-1.058838	0.384329
O	-2.976047	-1.000660	1.633880
N	-0.911849	0.245719	0.246153
N	-4.047053	-1.451568	-0.309319
H	-3.948831	-1.546860	-1.324689
C	-5.259202	-1.929766	0.332449
H	-5.051276	-2.796706	0.966963
H	-5.706845	-1.143105	0.946007
H	-5.965823	-2.218078	-0.445996
C	-1.113885	1.569894	0.319871
O	-0.503101	2.243829	1.182453
C	-2.080473	2.216150	-0.626535
H	-3.101886	1.900241	-0.382839
H	-2.011266	3.300013	-0.528876
H	-1.872161	1.924462	-1.658901
H	-0.285581	-0.133964	0.950987
H	-2.919926	0.628627	2.550496
H	-1.410136	-1.279422	2.603852
H	-0.794121	3.992620	1.421926
H	0.153452	1.541368	2.705045
H	-0.296334	0.086833	-3.048189
H	-2.491938	-1.963331	-3.084354
O	-3.477465	-1.880138	-3.173581
H	-3.789251	-2.791336	-3.269449
O	-0.303713	1.034439	-3.343264
H	0.547603	1.172011	-3.785224
O	-2.957114	1.484779	3.021614
H	-2.173137	1.961303	2.708779
O	-0.587870	-1.425264	3.112644
H	-0.251508	-0.522192	3.295064
O	-0.901554	4.948332	1.620449
H	-1.070400	4.981167	2.571640
O	0.387059	1.180970	3.588310
H	-0.304505	1.521077	4.173996

63

complexB_6sol_conf_39

Eopt -2358.099415

C	-3.166874	0.286956	-0.950221
C	-2.673225	0.390721	0.371867
C	-1.851676	1.426896	0.837275
C	-1.492148	2.410055	-0.061186
C	-1.947536	2.334580	-1.386273
C	-2.761095	1.302738	-1.823928
H	-1.515253	1.438858	1.867821
H	-1.649267	3.102838	-2.092303
H	-3.083164	1.291328	-2.857178
C	-0.594734	3.529062	0.339589
O	-0.402905	4.516470	-0.350481
O	-0.016272	3.319645	1.513855
C	0.891991	4.330878	1.983186
H	1.274508	3.952499	2.929515
H	0.357688	5.271315	2.134084
N	-2.979817	-1.415964	2.032519
N	-2.925538	-0.620245	1.262232
S	-4.214664	-1.039294	-1.384242
C	-4.178966	-1.024164	-3.194819
H	-4.709895	-0.158794	-3.595146
H	-3.150251	-1.059918	-3.563263
H	-4.702569	-1.934278	-3.497762
H	1.705279	4.463089	1.266792
C	1.268875	-0.424015	1.951185
H	0.886481	0.520087	1.545818
H	2.178314	-0.194387	2.514433
S	0.027807	-1.184838	3.068719
C	1.685199	-1.331820	0.784706
H	2.053522	-2.273904	1.192170
C	0.513601	-1.589394	-0.168644
O	0.089772	-0.662698	-0.893608
N	2.766649	-0.707035	0.030823
N	0.023793	-2.825652	-0.184899
H	0.363405	-3.455693	0.538588
C	-1.158171	-3.241386	-0.918129
H	-0.995871	-4.242500	-1.323247
H	-2.031360	-3.264912	-0.259051
H	-1.343900	-2.554069	-1.741990
C	3.783434	-1.335995	-0.571059
O	4.555296	-0.702805	-1.332480
C	3.997242	-2.798148	-0.306199
H	3.120595	-3.381953	-0.603998
H	4.864860	-3.136234	-0.873555
H	4.171162	-2.972780	0.760791
H	2.625205	0.269888	-0.226850
H	-0.328427	-0.746453	-2.650989
H	0.896032	1.008251	-1.161696
H	5.623574	0.686786	-0.781180
H	3.880395	0.537207	-2.442329
H	-0.988717	0.538843	3.908398
H	0.408969	-3.363284	2.700765
O	0.606763	-4.293198	2.434923
H	-0.208601	-4.779741	2.622213
O	-1.454072	1.327022	4.292182
H	-1.075269	1.427360	5.177633
O	-0.475623	-0.709065	-3.619619

H 0.345153 -0.337772 -3.971700
O 1.448214 1.815255 -1.192935
H 2.099535 1.651299 -1.906317
O 6.248959 1.397833 -0.536438
H 7.095589 1.111974 -0.907017
O 3.484138 1.189623 -3.062113
H 4.056725 1.966992 -2.990202
63
complexB_6sol_conf_4 Eopt
-2358.106106
C -3.155773 0.548990 0.256128
C -2.224322 1.057145 1.196915
C -1.203016 1.965506 0.897972
C -1.107770 2.415874 -0.403535
C -2.034828 1.964987 -1.358388
C -3.031457 1.056584 -1.041508
H -0.504330 2.272164 1.668816
H -1.968569 2.320004 -2.380651
H -3.708132 0.731331 -1.821741
C 0.008778 3.341832 -0.741760
O 0.803805 3.762436 0.081996
O 0.050043 3.644174 -2.031990
C 1.120089 4.505383 -2.459610
H 2.081967 4.029550 -2.257014
H 1.053223 5.467386 -1.947788
N -2.369356 0.284999 3.547217
N -2.309378 0.633918 2.498294
S -4.326574 -0.637807 0.756926
C -5.143086 -1.091278 -0.791867
H -5.700858 -0.249575 -1.206442
H -4.417880 -1.478374 -1.511039
H -5.841855 -1.886120 -0.520494
H 0.973397 4.631266 -3.530964
C 2.142732 -0.553427 2.059818
H 3.180670 -0.261254 2.240919
H 2.097680 -1.638560 2.206836
S 1.056782 0.318327 3.250397
C 1.821787 -0.248453 0.582580
H 1.651113 0.827610 0.475551
C 3.044236 -0.635112 -0.247092
O 3.196174 -1.782020 -0.717985
N 0.632265 -0.965298 0.162016
N 3.960395 0.322218 -0.374827
H 3.800029 1.195429 0.123343
C 5.253096 0.088707 -0.995244
H 5.128540 -0.207888 -2.040063
H 5.824996 1.015732 -0.955819
H 5.803663 -0.695914 -0.467645
C 0.026590 -0.875129 -1.025802
O -1.009491 -1.546915 -1.249829
C 0.625864 -0.009572 -2.096514
H 1.349233 -0.607949 -2.662593
H -0.158087 0.310973 -2.784453
H 1.145159 0.866012 -1.703386
H 0.171849 -1.562179 0.852914
H 2.203849 -2.753049 -1.961641
H 2.432779 -3.211757 0.357969
H -0.717270 -3.367410 -0.812006
H -1.903472 -1.323896 -2.795773
H -0.218329 -1.493545 2.941610
H 2.480457 2.019798 2.335605
O 3.223230 2.486921 1.894578
H 4.010177 2.134593 2.334242
O -0.683095 -2.260539 2.525682
H -0.085104 -3.010182 2.655563
O 1.792162 -3.359213 -2.610692
H 2.400137 -4.110876 -2.655168
O 2.015060 -3.972069 0.807830
H 1.160987 -4.071695 0.336758
O -0.243828 -4.218967 -0.891858
H 0.414059 -4.020661 -1.594684
O -2.405821 -1.252318 -3.637098
H -1.984325 -1.894273 -4.224470
63
complexB_6sol_conf_40
Eopt -2358.102478
C -3.050099 -0.142657 -0.191663
C -2.421952 -0.181341 -1.461846
C -1.634239 0.844920 -1.994550
C -1.475205 1.988883 -1.238526
C -2.116055 2.083814 0.008878
C -2.875704 1.046947 0.524119
H -1.167212 0.723990 -2.965621
H -2.005309 2.985899 0.599127
H -3.325796 1.164235 1.501712
C -0.596923 3.067469 -1.770570
O 0.009176 2.978727 -2.826092
O -0.542757 4.122289 -0.970739
C 0.316791 5.201535 -1.377117
H 1.341245 4.841627 -1.490230
H 0.254623 5.933082 -0.573274
N -2.754294 -2.178337 -2.889793
N -2.599262 -1.289182 -2.249362
S -3.959285 -1.521116 0.359652
C -4.587660 -1.007217 1.975338
H -3.779460 -0.910939 2.702534
H -5.174465 -0.089838 1.902128
H -5.245582 -1.823351 2.285530
H -0.042523 5.630753 -2.314690
C 1.603640 -0.593660 -1.507778
H 2.647096 -0.521059 -1.828215
H 1.195352 0.422470 -1.566697
S 0.718123 -1.710538 -2.659260
C 1.569621 -1.049738 -0.027200
H 1.345954 -2.116819 0.013923
C 2.947372 -0.831333 0.603435
O 3.214083 0.208121 1.245199
N 0.543783 -0.332127 0.706508
N 3.826670 -1.805728 0.396944
H 3.543384 -2.580269 -0.210190

C 5.219965 -1.701118 0.795062
H 5.737781 -0.926894 0.220659
H 5.296172 -1.464011 1.859006
H 5.700815 -2.662203 0.610890
C 0.026091 -0.687860 1.893538
O -0.633946 0.141536 2.558875
C 0.243031 -2.086555 2.389260
H -0.324797 -2.233113 3.308566
H -0.075380 -2.819046 1.641423
H 1.306150 -2.253946 2.595846
H 0.434688 0.649150 0.466948
H 4.744615 1.198502 1.105740
H 2.442409 1.822092 0.685493
H -0.290097 1.903111 2.412716
H -1.528167 -0.268811 4.046182
H 2.194491 -3.227928 -2.003026
H -0.329172 -3.096664 -1.315057
O -0.803221 -3.764087 -0.754864
H -0.097932 -4.183689 -0.242507
O 2.869434 -3.813427 -1.571276
H 3.614273 -3.826330 -2.189031
O 5.447809 1.873563 1.016035
H 4.961587 2.704148 0.917198
O 2.027884 2.680643 0.469464
H 1.319660 2.768829 1.144436
O -0.010781 2.845752 2.389881
H 0.514153 2.960296 3.194712
O -2.077089 -0.425073 4.845319
H -1.485039 -0.256070 5.590730
63
complexB_6sol_conf_41
Eopt -2358.107239
C -3.574164 0.244674 0.035941
C -2.623431 0.020291 1.063033
C -1.565192 0.881874 1.372231
C -1.421506 2.022987 0.608513
C -2.342655 2.281687 -0.420928
C -3.399056 1.427569 -0.691422
H -0.887724 0.635910 2.182905
H -2.232903 3.175372 -1.025026
H -4.083254 1.679922 -1.491332
C -0.263434 2.917865 0.885618
O 0.463438 2.798939 1.857552
O -0.106382 3.846538 -0.047633
C 1.014426 4.735217 0.104572
H 0.935665 5.280800 1.046912
H 0.952514 5.418967 -0.740270
N -2.790726 -2.021944 2.457829
N -2.729117 -1.115373 1.826591
S -4.836481 -0.921261 -0.245358
C -5.813172 -0.171326 -1.570505
H -5.221102 -0.064311 -2.480953
H -6.626863 -0.877515 -1.751995
H -6.232356 0.785904 -1.254796
H 1.945275 4.165623 0.068411
C 2.106628 -0.216743 1.607167
H 1.396423 0.454256 2.097330
H 3.108285 0.115999 1.891390
S 1.822163 -1.933928 2.188447
C 1.965375 0.008520 0.087668
H 1.961275 1.090657 -0.080492
C 3.199941 -0.514386 -0.644280
O 4.253989 0.160305 -0.581333
N 0.717374 -0.550904 -0.385710
N 3.123746 -1.659394 -1.308764
H 2.280362 -2.222421 -1.222452
C 4.274807 -2.250898 -1.969692
H 3.952067 -3.173913 -2.451553
H 4.669660 -1.571876 -2.729682
H 5.065984 -2.479714 -1.249529
C 0.025558 -0.170564 -1.470068
O -1.054160 -0.735107 -1.753009
C 0.567607 0.947602 -2.312660
H 0.557343 1.884278 -1.744299
H 1.601734 0.754095 -2.614557
H -0.052968 1.063841 -3.201679
H 0.305196 -1.274351 0.201163
H 4.222134 1.712673 0.379226
H 5.974384 -0.098053 -1.120001
H -2.122217 -0.117832 -3.058007
H -1.544807 -2.266057 -0.832076
H 1.391509 -3.253354 0.485088
H 0.148804 -1.311526 3.443542
O -0.583843 -0.926267 3.990275
H -0.569995 -1.440410 4.809425
O 1.134159 -3.718444 -0.353036
H 1.500550 -4.610529 -0.273983
O 4.207856 2.554186 0.884287
H 3.304220 2.606042 1.227640
O 6.922118 -0.136552 -1.366824
H 7.325403 0.608417 -0.901184
O -2.732725 0.163302 -3.774323
H -2.341917 -0.199131 -4.580903
O -1.619479 -3.120045 -0.359456
H -0.692679 -3.436488 -0.330441
63
complexB_6sol_conf_42
Eopt -2358.102617
C 2.187899 1.336593 1.033749
C 2.294439 1.951300 -0.237340
C 1.392467 2.891290 -0.747484
C 0.321492 3.251243 0.045329
C 0.184798 2.673702 1.320329
C 1.091431 1.746472 1.803891
H 1.539774 3.305751 -1.738580
H -0.653445 2.953327 1.948457
H 0.940422 1.335788 2.793625
C -0.657999 4.233813 -0.496721
O -0.557128 4.743656 -1.600635
O -1.646882 4.488271 0.349501

C -2.646025 5.429312 -0.083043
H -3.143734 5.057729 -0.980929
H -2.185072 6.400258 -0.275121
N 4.204269 1.349381 -1.697562
N 3.344836 1.606479 -1.049856
S 3.373244 0.166483 1.536812
C 2.748555 -0.393211 3.138533
H 1.731309 -0.775142 3.042440
H 3.412751 -1.208921 3.433606
H 2.802405 0.405404 3.881202
H -3.350101 5.496849 0.744464
C -0.299187 -0.219828 -1.465861
H -0.053365 0.408095 -0.602991
H -1.151695 0.248197 -1.968286
S 1.106871 -0.308005 -2.629550
C -0.797185 -1.575949 -0.929685
H -0.940808 -2.270792 -1.758287
C 0.184811 -2.175772 0.071026
O 0.215782 -1.751523 1.247419
N -2.064891 -1.416002 -0.228553
N 0.976197 -3.149217 -0.358875
H 0.954224 -3.390771 -1.351705
C 2.025743 -3.716845 0.469838
H 2.618804 -2.926634 0.939522
H 1.602603 -4.353573 1.252036
H 2.673279 -4.322586 -0.164812
C -3.288185 -1.442986 -0.770839
O -4.286714 -1.166423 -0.063903
C -3.437364 -1.835378 -2.211532
H -4.481308 -1.728623 -2.507269
H -2.811376 -1.213128 -2.857827
H -3.130912 -2.878049 -2.348785
H -2.010452 -1.093545 0.731865
H 0.518400 -2.763729 2.751502
H -0.767168 -0.700774 2.497821
H -5.985210 -1.318862 -0.656479
H -4.099727 -0.892500 1.711806
H 1.020645 -2.424544 -3.233609
H 2.890939 -1.443653 -1.936955
O 3.776481 -1.870084 -1.827122
H 3.594091 -2.818984 -1.879639
O 1.016922 -3.414289 -3.312460
H 1.944354 -3.644821 -3.466961
O 0.568044 -3.212816 3.620326
H 0.379407 -2.509651 4.257191
O -1.267895 -0.257079 3.207351
H -2.210954 -0.456184 3.020556
O -6.927266 -1.379833 -0.925552
H -7.378476 -1.752999 -0.156322
O -3.997847 -0.743177 2.678354
H -4.156439 -1.609230 3.078828
63
complexB_6sol_conf_44
Eopt -2358.100432
C -2.696145 1.598691 -0.315745
C -2.063591 0.872888 -1.355097
C -2.219349 -0.499882 -1.578777
C -3.058614 -1.197709 -0.733992
C -3.728012 -0.509774 0.292689
C -3.551776 0.848349 0.500102
H -1.685524 -0.985449 -2.388734
H -4.396230 -1.051248 0.952813
H -4.081362 1.324998 1.315651
C -3.192673 -2.667583 -0.935878
O -2.594839 -3.280718 -1.804985
O -4.023763 -3.229311 -0.070782
C -4.197551 -4.653990 -0.171504
H -4.614350 -4.911140 -1.147338
H -4.895277 -4.912434 0.623045
N -0.565576 2.074718 -2.926872
N -1.230665 1.542207 -2.219285
S -2.381869 3.300881 -0.136787
C -3.364579 3.751667 1.313068
H -4.430967 3.617859 1.122429
H -3.044609 3.186598 2.190516
H -3.158970 4.812696 1.473120
H -3.240219 -5.156706 -0.019777
C 2.618590 1.114678 -1.339606
H 1.847823 1.298350 -2.095991
H 3.539763 0.878031 -1.879792
S 2.851278 2.616643 -0.312878
C 2.205716 -0.156800 -0.571423
H 1.939619 -0.912816 -1.315334
C 3.397861 -0.736294 0.188777
O 4.279986 -1.346823 -0.461213
N 1.054215 0.110422 0.264221
N 3.472728 -0.572959 1.502167
H 2.799768 0.036367 1.965838
C 4.612675 -1.036303 2.276025
H 4.609003 -2.125884 2.359581
H 5.549773 -0.720116 1.811022
H 4.543903 -0.603599 3.273675
C 0.190178 -0.795258 0.747719
O -0.678945 -0.448983 1.577350
C 0.278870 -2.213360 0.260543
H 1.243475 -2.655363 0.533177
H -0.522941 -2.800146 0.710536
H 0.189379 -2.255031 -0.829778
H 0.974301 1.062312 0.618315
H 4.028371 -1.721654 -2.207432
H 4.636410 -3.101944 -0.076857
H -0.611910 1.089679 2.468043
H -1.793411 -1.627484 2.360200
H 2.523869 2.162730 1.906086
H 1.528454 3.924637 -1.444925
O 0.925813 4.520488 -1.960500
H 1.408175 4.702763 -2.778693
O 2.340177 1.854574 2.823906
H 1.362561 1.895992 2.900478
O 3.891699 -1.925898 -3.157947

H 4.441384 -2.705147 -3.320996
O 4.781963 -4.045333 0.138838
H 5.561759 -4.296422 -0.374384
O -0.479065 1.922140 2.975452
H -0.718402 1.701273 3.886217
O -2.400781 -2.230961 2.840494
H -1.886625 -2.542097 3.595978
63
complexB_6sol_conf_45
Eopt -2358.096640
C 2.032955 -1.293354 0.943034
C 2.709844 -1.273371 -0.299969
C 3.482551 -0.204114 -0.767881
C 3.612980 0.903999 0.043781
C 2.991943 0.906664 1.305567
C 2.228137 -0.160568 1.745302
H 3.961697 -0.263582 -1.739538
H 3.101953 1.766945 1.956720
H 1.769081 -0.102623 2.723955
C 4.425865 2.049632 -0.452400
O 4.957653 2.071379 -1.550599
O 4.495870 3.042930 0.423929
C 5.252127 4.201936 0.029263
H 6.293053 3.924225 -0.149011
H 5.178412 4.889510 0.870282
N 2.737311 -3.299595 -1.727083
N 2.674354 -2.385900 -1.105364
S 1.018154 -2.643981 1.346288
C 0.523746 -2.326464 3.057251
H -0.264603 -3.056381 3.256778
H 1.360117 -2.495455 3.739559
H 0.120577 -1.321649 3.194426
H 4.814702 4.644909 -0.867805
C -0.517500 0.148017 -1.206828
H 0.082992 0.145430 -0.288090
H -0.379513 1.129058 -1.670914
S 0.059338 -1.142849 -2.369171
C -2.008584 0.033858 -0.823837
H -2.596381 -0.197761 -1.713067
C -2.215217 -1.047105 0.233211
O -1.993835 -0.788904 1.436857
N -2.518412 1.274091 -0.251905
N -2.642984 -2.233543 -0.176921
H -2.720958 -2.396257 -1.181853
C -2.759690 -3.366622 0.723359
H -3.371356 -4.129190 0.238347
H -1.777294 -3.792419 0.955434
H -3.242981 -3.057139 1.653257
C -2.942975 2.352996 -0.921038
O -3.233374 3.398453 -0.289646
C -3.097408 2.269902 -2.412093
H -2.170346 1.939878 -2.890864
H -3.880717 1.546517 -2.665504
H -3.375792 3.248969 -2.803489
H -2.402746 1.386161 0.748708
H -2.453201 -1.279243 3.140498
H -1.018448 0.545604 2.326638
H -2.851222 3.450239 1.520648
H -3.980556 4.827109 -1.103801
H -0.299850 -3.337172 -1.831737
H -1.867099 -2.021997 -3.086245
O -2.752965 -2.455166 -3.191994
H -2.556029 -3.373090 -3.429975
O -0.390673 -4.300938 -1.635034
H -0.627085 -4.698557 -2.484434
O -2.727653 -1.361235 4.075740
H -2.565746 -0.478565 4.442772
O -0.660584 1.310636 2.819252
H -1.331361 2.015486 2.695457
O -2.589082 3.337466 2.460997
H -3.422778 3.254073 2.945426
O -4.395470 5.617781 -1.512878
H -4.510031 6.239232 -0.780861
63
complexB_6sol_conf_46
Eopt -2358.099691
C -0.180878 2.486965 1.143198
C -0.519641 2.694185 -0.215648
C -1.635857 2.137274 -0.853703
C -2.484197 1.356402 -0.096902
C -2.191940 1.137843 1.260344
C -1.068817 1.675567 1.863078
H -1.823861 2.342390 -1.902123
H -2.857553 0.526631 1.859093
H -0.890705 1.467416 2.910434
C -3.694350 0.782068 -0.749486
O -3.881680 0.797871 -1.954835
O -4.546215 0.270538 0.128766
C -5.779588 -0.260885 -0.390216
H -6.355587 0.541579 -0.856834
H -6.306338 -0.651269 0.478804
N 0.827147 4.295405 -1.546427
N 0.248962 3.553158 -0.963406
S 1.257054 3.221667 1.798570
C 1.207603 2.755181 3.546088
H 0.326768 3.171887 4.038941
H 1.245594 1.670320 3.663165
H 2.107512 3.197694 3.979616
H -5.584969 -1.058077 -1.109141
C 1.997282 0.004874 -0.336973
H 3.012508 -0.053819 0.069964
H 1.371719 0.441385 0.448117
S 2.009787 1.064294 -1.831040
C 1.465760 -1.428665 -0.610990
H 1.506111 -1.631828 -1.680688
C 2.361256 -2.434310 0.110655
O 2.117353 -2.790963 1.284701
N 0.099394 -1.626888 -0.166737
N 3.423897 -2.840051 -0.579291
H 3.578940 -2.413702 -1.496180

C 4.488610 -3.660981 -0.028746
H 5.428838 -3.102977 -0.030706
H 4.241597 -3.941607 0.994856
H 4.611184 -4.566844 -0.627700
C -1.028642 -1.594396 -0.887511
O -2.130177 -1.800529 -0.320371
C -0.942044 -1.312142 -2.358647
H -0.375324 -0.391654 -2.535005
H -0.425693 -2.130083 -2.873491
H -1.946141 -1.209255 -2.770362
H -0.023024 -1.770549 0.828556
H 1.404420 -1.575746 2.627990
H 0.661129 -3.753798 1.940874
H -3.393407 -2.506682 -1.453477
H -2.033302 -1.889475 1.602255
H 3.334674 2.706191 -1.286977
H 3.374406 -0.422037 -2.716964
O 3.909207 -1.193991 -3.040817
H 4.818941 -0.865346 -3.061999
O 3.938416 3.454473 -1.039029
H 4.381650 3.695118 -1.864340
O 0.886581 -0.969934 3.191080
H -0.039274 -1.252138 3.051997
O -0.093323 -4.234963 2.336345
H 0.228482 -4.518577 3.203419
O -4.029849 -2.978267 -2.031327
H -3.944603 -3.905511 -1.770528
O -1.768168 -2.000999 2.538504
H -1.284455 -2.856158 2.537587
63
complexB_6sol_conf_47
Eopt -2358.105432
C 2.637624 1.648896 0.636459
C 2.009914 0.754010 1.540582
C 2.125338 -0.640620 1.494948
C 2.921173 -1.185448 0.509190
C 3.601500 -0.330773 -0.376489
C 3.463980 1.045401 -0.320186
H 1.596598 -1.257568 2.213311
H 4.245812 -0.753088 -1.139205
H 3.996249 1.652618 -1.041441
C 2.986020 -2.669603 0.403457
O 2.455326 -3.423747 1.200053
O 3.666614 -3.077286 -0.661675
C 3.754225 -4.500204 -0.868278
H 4.257257 -4.969187 -0.020752
H 4.341519 -4.618672 -1.777062
N 0.612562 1.683867 3.362756
N 1.233522 1.270835 2.543873
S 2.347023 3.358169 0.774518
C 3.370098 4.066880 -0.537820
H 3.042938 3.718038 -1.519022
H 3.208478 5.145172 -0.467077
H 4.426636 3.849001 -0.370215
H 2.753474 -4.916288 -1.000081
C -2.505041 1.294073 1.399893
H -1.735918 1.316179 2.177841
H -3.444608 1.041310 1.900668
S -2.624116 2.938325 0.595237
C -2.168845 0.119115 0.461115
H -1.950723 -0.750469 1.085710
C -3.393519 -0.279122 -0.357533
O -4.299894 -0.937698 0.211371
N -1.001457 0.423447 -0.338241
N -3.471937 0.066997 -1.634026
H -2.784146 0.716147 -2.015909
C -4.637827 -0.245300 -2.444252
H -5.539340 0.212198 -2.025426
H -4.469178 0.146795 -3.447257
H -4.784500 -1.327101 -2.502847
C -0.211759 -0.466575 -0.956472
O 0.671813 -0.077112 -1.751950
C -0.401460 -1.927393 -0.658881
H -1.397647 -2.261298 -0.969685
H 0.351419 -2.505134 -1.196373
H -0.304621 -2.121782 0.414523
H -0.857793 1.409740 -0.550899
H -4.282135 -2.692054 -0.402097
H -3.951869 -1.604131 1.834729
H 1.752379 -1.247553 -2.623452
H 0.745606 1.602533 -2.337760
H -2.317050 2.755401 -1.633507
O -2.146056 2.569778 -2.589096
H -1.167565 2.573181 -2.662409
O -4.262496 -3.621571 -0.702639
H -4.289504 -3.557328 -1.667364
O -3.776750 -2.024616 2.707373
H -3.733770 -2.971835 2.514684
O 2.328648 -1.851702 -3.138844
H 2.848015 -2.315085 -2.464234
O 0.683006 2.514905 -2.700308
H 0.919090 2.425528 -3.634115
O -1.221515 -1.432424 3.686473
H -2.097910 -1.603189 3.274589
H -0.650645 -1.160265 2.955060
63
complexB_6sol_conf_48
Eopt -2358.107325
C 3.105908 -0.069342 1.259276
C 2.950390 -0.226520 -0.138605
C 2.454688 0.755033 -1.004096
C 2.075378 1.961871 -0.451025
C 2.165258 2.141203 0.938698
C 2.661835 1.152660 1.774924
H 2.356017 0.539681 -2.064406
H 1.845487 3.078439 1.380060
H 2.723671 1.350168 2.838025
C 1.552854 3.019255 -1.360639
O 1.303676 2.827447 -2.539568
O 1.407841 4.188942 -0.754345

C 0.862429 5.261181 -1.541885
H 0.811617 6.113637 -0.866658
H -0.135067 4.991950 -1.895506
N 3.412127 -2.404454 -1.224272
N 3.251922 -1.436562 -0.711802
S 3.843400 -1.339940 2.201921
C 3.354376 -0.918062 3.893907
H 2.270390 -0.802584 3.962278
H 3.666002 -1.770661 4.501836
H 3.868750 -0.022176 4.245937
H 1.520880 5.479163 -2.385145
C -0.592517 -0.468301 -2.617149
H -0.239108 0.502672 -2.246747
H -1.277066 -0.268432 -3.447797
S 0.817444 -1.480846 -3.203761
C -1.420438 -1.137986 -1.513020
H -1.722574 -2.130082 -1.851778
C -0.628028 -1.243193 -0.207698
O -0.370468 -0.208606 0.444753
N -2.626255 -0.353166 -1.262277
N -0.274500 -2.462317 0.183752
H -0.439173 -3.230054 -0.467733
C 0.519691 -2.744861 1.364904
H 1.421551 -3.293194 1.080607
H 0.799466 -1.810174 1.849580
H -0.054679 -3.350689 2.071525
C -3.785203 -0.813773 -0.772206
O -4.688253 -0.012678 -0.427948
C -3.991983 -2.296184 -0.658745
H -3.838918 -2.786941 -1.625000
H -3.282573 -2.727620 0.054386
H -5.008409 -2.487759 -0.313795
H -2.510322 0.656564 -1.250374
H -0.845833 1.541305 0.166794
H -0.651910 0.018017 2.236689
H -4.233588 1.627225 0.123480
H -5.314310 -0.470948 1.258742
H 0.209254 -3.469017 -2.528677
O -0.106457 -4.325287 -2.130436
H 0.703564 -4.779872 -1.859211
O -1.119591 2.476572 0.074878
H -2.082119 2.469415 0.251380
O -0.908593 0.223261 3.157437
H -1.833142 0.549833 3.087675
O -3.881824 2.433485 0.565641
H -4.491617 3.150821 0.344792
O -5.458163 -0.686488 2.203891
H -6.289488 -0.248076 2.433580
O -3.529169 1.190546 3.047558
H -4.159775 0.461634 2.865385
H -3.603357 1.750382 2.246977
63
complexB_6sol_conf_49
Eopt -2358.108686
C -0.939197 -2.726848 0.394282
C -0.352538 -2.698736 -0.896556
C 1.003691 -2.459460 -1.152998
C 1.827429 -2.213806 -0.075993
C 1.282984 -2.214950 1.221505
C -0.056806 -2.471384 1.453721
H 1.371309 -2.449679 -2.173210
H 1.923180 -2.004220 2.070721
H -0.423396 -2.459784 2.472684
C 3.253820 -1.880906 -0.349052
O 3.743312 -1.900574 -1.466392
O 3.921477 -1.559008 0.748444
C 5.286344 -1.141377 0.573526
H 5.874610 -1.955413 0.144963
H 5.641494 -0.898207 1.573208
N -1.800633 -3.073162 -2.871456
N -1.152731 -2.910856 -1.987830
S -2.632570 -3.072827 0.558760
C -2.928201 -2.931470 2.335680
H -2.736380 -1.914319 2.682542
H -3.990675 -3.153872 2.460346
H -2.340445 -3.665007 2.891104
H 5.323924 -0.261924 -0.072763
C -2.008338 0.758521 1.369744
H -1.662281 1.544851 2.048218
H -1.639507 -0.190123 1.774708
S -3.834876 0.761869 1.299949
C -1.307009 0.948500 0.013862
H -1.643544 0.164331 -0.670384
C -1.616324 2.326848 -0.575998
O -1.074506 3.356376 -0.108115
N 0.133213 0.824245 0.215876
N -2.462287 2.375251 -1.595861
H -2.952741 1.517159 -1.851666
C -2.902531 3.632116 -2.174912
H -3.590577 3.410033 -2.990821
H -3.415357 4.248927 -1.429919
H -2.050083 4.191447 -2.569530
C 1.063875 0.787843 -0.737162
O 2.282973 0.848614 -0.432350
C 0.663291 0.706502 -2.180183
H 0.549038 1.726660 -2.567163
H 1.464075 0.218490 -2.739060
H -0.275219 0.170743 -2.337698
H 0.473310 0.900650 1.180811
H -0.463835 3.501604 1.604911
H 0.794142 3.715297 -0.320453
H 3.106318 1.291636 1.066379
H 3.479370 1.311339 -1.680419
H -4.071075 0.079096 -0.742751
O -4.085847 -0.118618 -1.721875
H -4.944467 0.210269 -2.023628
O -0.116871 3.689709 2.504047
H 0.672310 4.227646 2.347971
O 1.745936 3.911056 -0.418462
H 2.177718 3.045265 -0.341376

O 3.528612 1.553978 1.914744
H 3.594179 2.518017 1.864325
O 4.107938 1.669487 -2.344301
H 3.668397 2.465458 -2.674122
O 0.996176 1.180486 3.058118
H 0.594893 2.071826 2.959774
H 1.930973 1.308391 2.790197
63
complexB_6sol_conf_5 Eopt
-2358.101355
C -3.439984 0.379851 0.006997
C -2.717076 0.108934 1.192622
C -1.952266 -1.040972 1.420541
C -1.884819 -1.978194 0.409303
C -2.549103 -1.728085 -0.803292
C -3.309248 -0.587819 -0.998974
H -1.441403 -1.180731 2.367441
H -2.479080 -2.447178 -1.612185
H -3.808130 -0.452586 -1.950199
C -1.133485 -3.237335 0.664118
O -0.618247 -3.501661 1.740739
O -1.119108 -4.047951 -0.380958
C -0.402195 -5.286677 -0.233183
H 0.648676 -5.084242 -0.017117
H -0.507486 -5.793914 -1.190376
N -2.888350 1.684878 3.102383
N -2.798239 1.000250 2.238490
S -4.400939 1.823340 -0.119714
C -5.249181 1.615588 -1.704485
H -4.546038 1.644636 -2.539693
H -5.837451 0.695760 -1.718168
H -5.924018 2.471910 -1.777843
H -0.848186 -5.882363 0.566010
C 0.688295 2.118994 1.124107
H 1.353113 2.836959 1.613301
H 0.416364 1.382635 1.889336
S -0.806976 2.999253 0.536069
C 1.475994 1.393421 0.005434
H 1.206148 1.828365 -0.958226
C 2.974344 1.611913 0.215444
O 3.676454 0.795181 0.853735
N 1.140330 -0.018399 -0.026449
N 3.460258 2.738758 -0.293622
H 2.803598 3.378714 -0.750021
C 4.823248 3.180645 -0.051453
H 5.033797 3.216433 1.021550
H 5.541981 2.511011 -0.530448
H 4.936904 4.180269 -0.471509
C 1.319763 -0.851162 -1.063105
O 1.174055 -2.083147 -0.900340
C 1.683964 -0.282653 -2.403215
H 1.719919 -1.090528 -3.135204
H 0.954780 0.469175 -2.719372
H 2.666792 0.199999 -2.353404
H 0.988058 -0.472172 0.870130
H 5.239277 0.168474 0.156934
H 2.946841 -0.276825 2.130029
H 1.403713 -3.298355 -2.186372
H 1.918615 -2.869454 0.567495
H -1.197354 2.100526 -1.444968
H 0.580623 4.128405 -0.766953
O 1.308364 4.502535 -1.325635
H 1.605073 5.288936 -0.846093
O -1.371176 1.760851 -2.359250
H -1.033567 2.455509 -2.941349
O 6.044721 -0.256442 -0.204027
H 5.815141 -1.193094 -0.273478
O 2.553541 -0.854542 2.817721
H 2.491294 -1.725564 2.371295
O 1.510667 -3.985343 -2.880376
H 1.570238 -3.484563 -3.705331
O 2.253686 -3.280148 1.392416
H 1.437250 -3.599385 1.810138
63
complexB_6sol_conf_50
Eopt -2358.108846
C 3.110679 1.021908 0.557432
C 2.663840 0.638351 -0.730805
C 1.614053 1.247596 -1.427841
C 0.954170 2.291156 -0.811239
C 1.346452 2.683903 0.479364
C 2.392454 2.067805 1.148243
H 1.336105 0.874786 -2.410095
H 0.824606 3.494712 0.974409
H 2.654388 2.417565 2.138741
C -0.164563 2.947579 -1.543485
O -0.547968 2.588625 -2.644675
O -0.684372 3.967580 -0.875029
C -1.774282 4.665987 -1.502194
H -2.054499 5.447235 -0.797738
H -2.608866 3.982557 -1.669374
N 3.672068 -1.295184 -1.904098
N 3.263649 -0.419744 -1.363215
S 4.479079 0.218745 1.278282
C 4.560922 0.944372 2.933451
H 3.637659 0.756549 3.484849
H 5.386587 0.426679 3.427368
H 4.785051 2.011714 2.885901
H -1.442668 5.102679 -2.446510
C -0.979881 -1.102723 -2.058647
H -0.862229 -0.045740 -1.790236
H -1.900799 -1.182850 -2.645438
S 0.443636 -1.667013 -3.061602
C -1.210531 -1.903127 -0.766902
H -1.278298 -2.963946 -1.019739
C -0.070958 -1.676471 0.229300
O 0.012621 -0.590034 0.842216
N -2.478611 -1.475237 -0.179182
N 0.781737 -2.679720 0.399307
H 0.683063 -3.490681 -0.213067

C 1.955698 -2.621503 1.249566
H 2.858283 -2.777537 0.652088
H 2.008294 -1.648345 1.736055
H 1.902223 -3.398925 2.016815
C -3.111085 -2.052145 0.847933
O -4.152433 -1.537638 1.329488
C -2.557375 -3.312041 1.446682
H -1.756418 -3.046781 2.147102
H -3.348251 -3.816197 2.003859
H -2.143881 -3.993116 0.699121
H -2.838665 -0.566162 -0.484651
H -1.077036 0.855631 0.942390
H 0.385812 -0.181612 2.563256
H -3.764245 -0.155759 2.500671
H -5.154715 -0.207818 0.665169
H 0.652759 -3.713517 -2.339219
O 0.722337 -4.595776 -1.882058
H 1.663935 -4.815901 -1.917523
O -1.652826 1.621815 1.150684
H -2.268059 1.277422 1.834323
O 0.427701 0.147877 3.484479
H -0.287786 0.797855 3.527324
O -3.466762 0.606183 3.037174
H -4.197432 1.238589 2.982176
O -5.675988 0.558406 0.336334
H -5.728273 1.159070 1.093308
O -3.349326 1.314677 -1.038637
H -2.733522 1.555463 -0.310815
H -4.209903 1.170258 -0.592038
63
complexB_6sol_conf_51
Eopt -2358.103569
C 3.372012 0.189006 0.698389
C 2.974061 -0.285307 -0.576145
C 2.296089 0.474710 -1.535365
C 1.991594 1.783096 -1.219773
C 2.359385 2.290365 0.038164
C 3.029164 1.518043 0.973367
H 2.007590 0.019170 -2.477954
H 2.111727 3.313779 0.296221
H 3.284224 1.960110 1.928166
C 1.241718 2.590747 -2.221053
O 0.849483 2.143145 -3.286293
O 1.049797 3.842824 -1.829215
C 0.309016 4.693860 -2.721505
H -0.692295 4.287333 -2.877323
H 0.836492 4.786925 -3.672946
N 3.367338 -2.652532 -1.187353
N 3.219985 -1.592169 -0.905748
S 4.230218 -0.865809 1.786197
C 4.419614 0.134203 3.281870
H 4.935781 -0.517472 3.990959
H 5.035073 1.015288 3.090886
H 3.446047 0.411107 3.690621
H 0.256169 5.657372 -2.217609
C -0.932741 -0.954181 -2.035276
H -0.448235 0.024640 -1.929457
H -1.764656 -0.827367 -2.736868
S 0.253859 -2.198163 -2.669150
C -1.552787 -1.331724 -0.683396
H -2.048993 -2.298156 -0.772152
C -0.494096 -1.414874 0.412877
O 0.113243 -0.388451 0.783035
N -2.550365 -0.348065 -0.267117
N -0.285769 -2.608936 0.961809
H -0.672309 -3.419678 0.477639
C 0.764801 -2.839831 1.937093
H 0.701686 -2.109021 2.745520
H 0.633756 -3.839921 2.351599
H 1.755458 -2.774837 1.476100
C -3.870893 -0.444999 -0.452002
O -4.617192 0.518850 -0.146438
C -4.449240 -1.715208 -1.007211
H -5.521409 -1.582143 -1.154945
H -3.982961 -1.976489 -1.962241
H -4.284133 -2.544594 -0.311221
H -2.204130 0.571784 0.016682
H -0.400213 1.395140 0.455602
H 0.388387 0.237570 2.493917
H -4.425541 1.361986 1.436675
H -6.246498 0.083485 0.535712
H -0.529338 -3.970261 -1.634303
O -0.908943 -4.696732 -1.069010
H -0.165759 -5.295118 -0.907784
O -0.981772 2.178739 0.404611
H -1.278992 2.307583 1.330669
O 0.374945 0.708076 3.350441
H -0.406340 1.290736 3.284996
O -4.464874 1.772927 2.328023
H -4.818636 1.077800 2.900660
O -7.081529 -0.109874 1.012919
H -6.993333 0.373973 1.845844
O -1.898380 2.382092 3.043864
H -2.839548 2.162470 2.837129
H -1.897046 3.312063 3.309637
63
complexB_6sol_conf_52
Eopt -2358.099899
C -3.140152 -0.708829 0.797190
C -2.581036 -0.567895 -0.500011
C -1.584844 -1.399486 -1.032831
C -1.114232 -2.425966 -0.239465
C -1.625900 -2.583827 1.059307
C -2.607523 -1.751577 1.567360
H -1.225481 -1.215361 -2.040452
H -1.244346 -3.386043 1.681430
H -2.969495 -1.924496 2.573087
C -0.083677 -3.383201 -0.731132
O 0.371402 -4.284473 -0.047830
O 0.257957 -3.161987 -1.992405

C 1.280601 -4.002478 -2.551701
H 2.163250 -4.002496 -1.910288
H 1.509971 -3.566553 -3.522062
N -3.463837 1.187332 -2.007048
N -3.067986 0.406675 -1.328431
S -4.394517 0.375311 1.320817
C -4.816286 -0.239785 2.969003
H -3.964044 -0.154683 3.645847
H -5.617840 0.415739 3.317073
H -5.185723 -1.265687 2.922924
H 0.895774 -5.018195 -2.667728
C -0.553617 1.983049 0.980188
H -0.116060 2.393122 1.897955
H -1.186361 1.142749 1.287361
S -1.549163 3.258597 0.128054
C 0.579207 1.392021 0.124900
H 0.176179 1.097415 -0.847412
C 1.712821 2.407532 -0.047070
O 2.499492 2.623107 0.904678
N 1.099991 0.202802 0.787350
N 1.822049 3.015774 -1.220106
H 1.117671 2.820476 -1.929741
C 2.824179 4.027937 -1.504420
H 3.067732 4.580971 -0.594870
H 3.738142 3.571665 -1.899002
H 2.421444 4.718676 -2.247457
C 1.867786 -0.747563 0.241028
O 2.303981 -1.682541 0.952672
C 2.206056 -0.661831 -1.218376
H 2.688174 -1.587827 -1.531065
H 1.317931 -0.484180 -1.831143
H 2.902667 0.167901 -1.382400
H 0.965090 0.142750 1.792374
H 2.277645 2.027452 2.661427
H 4.140520 1.882128 0.559663
H 2.720089 -1.276729 2.656487
H 3.322676 -3.004576 0.309636
H -1.029925 2.827593 -1.910964
O -0.654279 2.647444 -2.821218
H -0.970507 3.373127 -3.378603
O 2.161570 1.715819 3.580517
H 2.475621 0.789437 3.580539
O 4.981087 1.445206 0.316152
H 5.497909 1.430216 1.134959
O 3.011731 -0.977182 3.544449
H 2.506892 -1.518303 4.167744
O 3.895324 -3.740550 0.001205
H 4.632279 -3.759069 0.626534
O -1.201556 0.135707 -3.834699
H -0.992601 1.035857 -3.498199
H -0.861910 0.123293 -4.739928
63
complexB_6sol_conf_53
Eopt -2358.106698
C 3.606541 -0.499688 -0.386144
C 2.460598 -0.790415 -1.165380
C 1.519970 0.156752 -1.578841
C 1.683681 1.459953 -1.153632
C 2.798310 1.788951 -0.364438
C 3.742664 0.840815 -0.003341
H 0.681475 -0.148497 -2.195012
H 2.940900 2.812719 -0.036315
H 4.594977 1.146883 0.590791
C 0.633604 2.445578 -1.530293
O -0.199073 2.240086 -2.395919
O 0.688949 3.563113 -0.814312
C -0.321395 4.552775 -1.088521
H -0.113461 5.370150 -0.400437
H -1.311665 4.132313 -0.900394
N 1.884876 -3.086644 -1.889904
N 2.201964 -2.080664 -1.553429
S 4.729954 -1.768518 0.013261
C 6.025702 -0.905652 0.934591
H 5.632518 -0.469017 1.854291
H 6.751110 -1.682401 1.187913
H 6.511965 -0.149181 0.315340
H -0.238943 4.890303 -2.123155
C -2.110638 -0.676214 -1.818075
H -1.510725 0.013638 -2.417642
H -3.133168 -0.617617 -2.202157
S -1.451528 -2.379805 -2.007779
C -2.131238 -0.111292 -0.375972
H -2.207082 0.976599 -0.460176
C -3.396234 -0.549636 0.358253
O -4.467388 0.047473 0.098503
N -0.905054 -0.446881 0.313293
N -3.331536 -1.545512 1.230590
H -2.468436 -2.078996 1.313986
C -4.508750 -2.052508 1.915655
H -5.244009 -2.436337 1.202198
H -4.194236 -2.861877 2.574642
H -4.971086 -1.264477 2.515403
C -0.309159 0.254395 1.284886
O 0.773084 -0.147721 1.771561
C -0.958446 1.523467 1.755312
H -0.933939 2.275881 0.958445
H -2.007093 1.354418 2.020884
H -0.421814 1.904091 2.624684
H -0.428532 -1.281910 -0.030326
H -6.184460 -0.076457 0.685544
H -4.415276 1.617312 -0.805342
H 1.823966 0.979958 2.632783
H 1.263674 -1.901307 1.451252
H -1.381672 -3.378621 -0.062863
O -1.258591 -3.674172 0.878642
H -1.671497 -4.547087 0.936125
O -7.133575 -0.035730 0.926344
H -7.498821 0.654591 0.356501
O -4.393136 2.482715 -1.268065
H -4.015873 3.093423 -0.620332

O	2.438748	1.610668	3.072949
H	2.110304	1.674007	3.980996
O	1.402496	-2.858300	1.300424
H	0.498244	-3.211246	1.174072
O	1.489209	4.032186	2.036090
H	1.827255	3.178684	2.386895
H	1.171776	3.814904	1.145765

63
complexB_6sol_conf_54
Eopt -2358.097834

C	3.612298	0.073150	0.309699
C	2.824352	-0.130218	-0.849793
C	2.012327	0.841922	-1.448368
C	1.973996	2.090818	-0.861244
C	2.726541	2.328135	0.300636
C	3.520860	1.349605	0.876514
H	1.430405	0.577435	-2.327329
H	2.686341	3.307644	0.765670
H	4.079704	1.588775	1.772919
C	1.131839	3.184568	-1.420852
O	1.128029	4.322095	-0.980053
O	0.387587	2.783006	-2.442628
C	-0.506638	3.751108	-3.017796
H	0.063300	4.575907	-3.450856
H	-1.195656	4.120783	-2.255647
N	2.722430	-2.350210	-1.942341
N	2.810891	-1.365448	-1.444094
S	4.610635	-1.219849	0.916507
C	5.206973	-0.576174	2.497938
H	5.864377	0.283523	2.354613
H	4.368018	-0.333310	3.154171
H	5.780951	-1.396963	2.935708
H	-1.048056	3.212498	-3.793715
C	-1.395293	-0.213693	-1.579804
H	-0.822354	0.691686	-1.342457
H	-2.343932	0.112683	-2.018494
S	-0.485569	-1.257775	-2.780652
C	-1.740784	-0.916143	-0.254447
H	-2.274082	-1.847408	-0.456133
C	-0.488641	-1.201282	0.570190
O	0.108605	-0.253563	1.126971
N	-2.588987	-0.067995	0.578503
N	-0.107814	-2.468789	0.678536
H	-0.599940	-3.161053	0.116344
C	1.115994	-2.887527	1.338010
H	1.795699	-2.040259	1.427163
H	0.909255	-3.277606	2.339234
H	1.590644	-3.669882	0.741434
C	-3.922411	0.047059	0.525498
O	-4.492487	0.942507	1.195460
C	-4.703882	-0.925394	-0.308498
H	-5.759035	-0.651248	-0.289682
H	-4.349730	-0.934030	-1.343697
H	-4.588519	-1.937358	0.093969
H	-2.108497	0.639260	1.122073
H	-0.142760	1.654047	1.319145
H	1.186844	-0.111796	2.584011
H	-6.279216	1.145621	1.228286
H	-3.493676	2.107683	2.193315
H	-1.032838	-3.190803	-2.035716
O	-1.265712	-4.042159	-1.565427
H	-0.784006	-4.747306	-2.019805
O	-0.319428	2.595705	1.500934
H	-1.206060	2.623201	1.919203
O	1.714356	0.037746	3.396368
H	1.057246	0.272636	4.065728
O	-7.249134	1.296644	1.266386
H	-7.407244	1.657172	2.149405
O	-2.878944	2.675272	2.708592
H	-3.320287	3.533742	2.772406
O	-3.402773	-4.436726	0.158976
H	-2.653632	-4.313621	-0.463117
H	-3.305921	-5.342380	0.483225

63
complexB_6sol_conf_55
Eopt -2358.108124

C	3.137504	0.326869	1.117892
C	2.957813	0.079139	-0.263513
C	2.308442	0.942508	-1.152990
C	1.794366	2.117472	-0.642665
C	1.910107	2.380605	0.732052
C	2.562013	1.510691	1.592135
H	2.200577	0.661046	-2.196329
H	1.484443	3.290109	1.140763
H	2.635232	1.769428	2.641213
C	1.092794	3.042861	-1.575762
O	0.865852	2.773630	-2.743852
O	0.758802	4.190394	-1.001708
C	0.031125	5.129691	-1.811336
H	0.633483	5.428347	-2.671712
H	-0.155606	5.982528	-1.161015
N	3.647405	-2.082502	-1.257266
N	3.386451	-1.114497	-0.788058
S	4.055032	-0.793809	2.091392
C	3.568025	-0.354767	3.779384
H	3.999939	-1.133295	4.412621
H	3.977628	0.612960	4.073994
H	2.480749	-0.371351	3.879174
H	-0.910519	4.685095	-2.140371
C	-0.655174	-0.744998	-2.423391
H	-0.367900	0.270592	-2.126559
H	-1.409982	-0.650857	-3.210833
S	0.803339	-1.658092	-3.056369
C	-1.334060	-1.439708	-1.237270
H	-1.574487	-2.461327	-1.529175
C	-0.436088	-1.446172	0.001379
O	-0.179713	-0.370796	0.588494
N	-2.582145	-0.764293	-0.883118
N	-0.007593	-2.627352	0.432518
H	-0.156745	-3.429467	-0.180588

C	0.881359	-2.798995	1.567344
H	0.602800	-3.709252	2.102256
H	1.921127	-2.888792	1.238967
H	0.788690	-1.946962	2.241663
C	-3.689680	-1.361330	-0.427106
O	-4.648103	-0.674338	0.006318
C	-3.785402	-2.860781	-0.451121
H	-4.757018	-3.157859	-0.055622
H	-3.680153	-3.239267	-1.473096
H	-2.995268	-3.311552	0.157269
H	-2.526668	0.250260	-0.764454
H	-1.146942	-0.097463	2.139197
H	-0.867550	1.248625	-0.029010
H	-4.684576	1.089512	0.276200
H	-4.510161	-0.706282	1.864963
H	0.352108	-3.673342	-2.303040
O	0.101073	-4.532484	-1.867183
H	0.945889	-4.951741	-1.650894
O	-1.640459	0.130521	2.951674
H	-2.524423	-0.286151	2.853793
O	-1.455950	1.976802	-0.316140
H	-1.823896	2.321909	0.523479
O	-4.824674	2.034989	0.508347
H	-4.472953	2.525533	-0.247978
O	-4.311993	-0.679336	2.822098
H	-4.682961	0.163950	3.119555
O	-2.652308	2.622785	2.166878
H	-2.286417	1.779933	2.516302
H	-3.490271	2.370223	1.720775

63
complexB_6sol_conf_56
Eopt -2358.102968

C	-3.270159	-0.203826	-1.015496
C	-2.019789	-0.860841	-1.124972
C	-0.787517	-0.212991	-1.266944
C	-0.783174	1.166245	-1.283492
C	-2.000275	1.857689	-1.166517
C	-3.210611	1.194740	-1.046811
H	0.126756	-0.790976	-1.350596
H	-2.003511	2.941954	-1.171224
H	-4.118790	1.778540	-0.970727
C	0.529352	1.859629	-1.398397
O	1.584350	1.269444	-1.556955
O	0.425369	3.177130	-1.303509
C	1.656280	3.921648	-1.336816
H	1.362919	4.964599	-1.229667
H	2.163270	3.761429	-2.290854
N	-1.906831	-3.336180	-1.099166
N	-1.979191	-2.231563	-1.112919
S	-4.731291	-1.138416	-0.861954
C	-6.029825	0.120107	-0.817083
H	-5.916494	0.765091	0.056317
H	-6.044586	0.703809	-1.739485
H	-6.962591	-0.442385	-0.732280
H	2.298121	3.614489	-0.508027
C	2.001958	-2.669618	1.909610
H	1.901372	-2.516559	2.988542
H	2.833204	-3.365769	1.758511
S	0.453999	-3.413610	1.251404
C	2.424422	-1.318585	1.310521
H	3.275894	-0.956246	1.890274
C	2.911992	-1.376094	-0.144832
O	3.923905	-0.723107	-0.487249
N	1.336595	-0.356468	1.436703
N	2.214512	-2.111716	-1.000245
H	1.429987	-2.649490	-0.610689
C	2.505491	-2.166325	-2.420899
H	1.821865	-2.881320	-2.880254
H	2.361225	-1.185598	-2.883796
H	3.534978	-2.492392	-2.596510
C	1.428817	0.973348	1.584227
O	0.387297	1.668102	1.611688
C	2.780371	1.607248	1.731749
H	3.386795	1.430055	0.839863
H	2.655785	2.680305	1.879364
H	3.311510	1.184070	2.590919
H	0.402585	-0.737720	1.294323
H	5.173939	-0.171839	0.692768
H	4.369094	0.111323	-2.042059
H	0.393182	3.466515	1.710289
H	-1.241083	0.983233	1.949664
H	-1.370476	-2.411889	1.802854
O	-2.290673	-2.048441	1.902232
H	-2.581204	-2.349136	2.775212
O	5.909230	0.119187	1.274653
H	6.636243	-0.483190	1.067053
O	4.686906	0.635945	-2.806258
H	5.527333	0.226102	-3.051091
O	0.306905	4.441179	1.791335
H	-0.429937	4.568047	2.404004
O	-2.114279	0.687142	2.283518
H	-2.166545	-0.273870	2.082827
O	-4.349592	2.251755	1.880417
H	-3.547252	1.686603	1.959551
H	-4.381849	2.745184	2.711058

63
complexB_6sol_conf_57
Eopt -2358.111082

C	2.862529	-1.524773	-0.257115
C	2.988047	-0.190712	0.200452
C	2.303266	0.338321	1.298725
C	1.440195	-0.491937	1.984214
C	1.280414	-1.821568	1.558451
C	1.975940	-2.328002	0.473624
H	2.453368	1.371797	1.588031
H	0.593851	-2.475655	2.084283
H	1.812585	-3.358825	0.185306
C	0.702338	0.073552	3.147556
O	0.852966	1.217689	3.544885
O	-0.130078	-0.797764	3.698454

C	-0.880158	-0.346000	4.839789
H	-1.548195	-1.169563	5.085429
H	-1.450215	0.549805	4.586898
N	4.602712	1.334292	-0.902728
N	3.860697	0.655416	-0.441317
S	3.767966	-2.047607	-1.648341
C	3.289168	-3.782755	-1.828071
H	3.850350	-4.141025	-2.694400
H	3.574914	-4.364408	-0.949430
H	2.220145	-3.873553	-2.031104
H	-0.200086	-0.141847	5.669621
C	0.204524	0.046165	-1.916232
H	-0.439298	-0.308909	-2.730013
H	0.767596	-0.823161	-1.561272
S	1.364795	1.318055	-2.520743
C	-0.699186	0.486074	-0.748773
H	-0.089819	0.974317	0.017304
C	-1.780473	1.446415	-1.243154
O	-2.775540	1.013481	-1.868852
N	-1.308340	-0.710318	-0.184187
N	-1.610100	2.735841	-0.986424
H	-0.744298	3.027378	-0.534749
C	-2.522630	3.753123	-1.478793
H	-2.578927	3.730139	-2.571342
H	-3.524933	3.601276	-1.069464
H	-2.151481	4.727159	-1.159683
C	-2.099841	-0.796917	0.893018
O	-2.585395	-1.902693	1.231985
C	-2.431854	0.451759	1.655632
H	-2.809959	0.176185	2.640516
H	-1.576215	1.124305	1.760010
H	-3.223323	0.992581	1.123014
H	-1.167621	-1.576569	-0.695790
H	-4.406007	1.189402	-1.038369
H	-3.275577	-0.760948	-2.162251
H	-2.451752	-3.242602	-0.113164
H	-4.457252	-1.707831	0.619198
H	1.346163	2.731806	-0.917027
O	1.228816	3.396162	-0.176673
H	1.203448	4.254121	-0.626342
O	-5.239317	1.205388	-0.522769
H	-5.937646	1.262961	-1.190342
O	-3.768020	-1.595385	-2.309436
H	-4.390039	-1.625874	-1.551328
O	-2.362979	-3.663986	-0.991527
H	-2.802454	-3.015144	-1.581930
O	-5.261286	-1.521707	0.100647
H	-5.283787	-0.542738	0.011696
O	3.725310	3.675807	1.047670
H	2.840390	3.527989	0.645847
H	3.637618	3.361251	1.957764

63
complexB_6sol_conf_58
Eopt -2358.103384

C	-1.976127	1.899560	-0.634771
C	-1.748171	0.805271	-1.503477
C	-2.542293	-0.343984	-1.544548
C	-3.616871	-0.428119	-0.680204
C	-3.892599	0.648768	0.176848
C	-3.099763	1.783739	0.195192
H	-2.298029	-1.148419	-2.229640
H	-4.737294	0.595600	0.854269
H	-3.350323	2.580276	0.884087
C	-4.414293	-1.684035	-0.672766
O	-4.136516	-2.660674	-1.350656
O	-5.444828	-1.633209	0.160493
C	-6.238833	-2.826296	0.281538
H	-5.618589	-3.650795	0.639425
H	-6.688743	-3.076357	-0.681353
N	0.210511	0.826506	-3.042931
N	-0.680098	0.825174	-2.373102
S	-0.884562	3.258706	-0.640698
C	-1.675281	4.428807	0.490682
H	-1.705026	4.034917	1.508577
H	-1.033954	5.313386	0.470321
H	-2.674393	4.702141	0.144899
H	-7.009077	-2.583069	1.011331
C	3.216683	0.472244	-0.802117
H	4.032326	-0.038093	-1.324603
H	3.640636	0.880635	0.123229
S	2.564458	1.840926	-1.820903
C	2.197990	-0.618466	-0.425074
H	1.856323	-1.116880	-1.335474
C	2.947128	-1.658267	0.410580
O	3.449093	-2.663847	-0.146349
N	1.050139	-0.071193	0.281091
N	3.091379	-1.409127	1.705839
H	2.755767	-0.513943	2.079030
C	3.858288	-2.282851	2.577398
H	3.434834	-3.290913	2.578952
H	4.903102	-2.338920	2.257275
H	3.815632	-1.877519	3.588488
C	-0.024788	-0.777846	0.666359
O	-0.859027	-0.281566	1.454143
C	-0.211601	-2.166356	0.125053
H	0.443429	-2.862901	0.662026
H	-1.246690	-2.474482	0.282641
H	0.030201	-2.229680	-0.939217
H	1.150129	0.852993	0.691088
H	3.552895	-2.595554	-1.977457
H	5.306201	-2.386367	-0.288687
H	-0.507335	1.046599	2.562806
H	-2.216393	-1.284138	2.121880
H	2.527972	2.986245	0.020082
O	2.633635	3.375411	0.929704
H	3.546318	3.696791	0.950575
O	3.625359	-2.513068	-2.952203
H	3.413568	-1.584130	-3.122455
O	6.233736	-2.136825	-0.464626
H	6.175800	-1.560275	-1.239335

O -0.241833 1.715631 3.232969
H -0.469440 1.314420 4.083258
O -2.919985 -1.792388 2.579346
H -3.224278 -2.433698 1.922590
O 2.501264 1.265050 2.804219
H 1.555870 1.392718 3.036726
H 2.652704 1.946659 2.113481
63
complexB_6sol_conf_59
Eopt -2358.103405
C -3.267822 -0.282496 -1.056127
C -1.999897 -0.913124 -1.096664
C -0.780293 -0.242071 -1.234857
C -0.808388 1.134595 -1.316888
C -2.043731 1.800977 -1.267012
C -3.241100 1.113918 -1.152150
H 0.149938 -0.799320 -1.267397
H -2.072718 2.883293 -1.324410
H -4.164512 1.677881 -1.130213
C 0.490001 1.853675 -1.435432
O 1.557483 1.282754 -1.584513
O 0.358345 3.169556 -1.359600
C 1.571009 3.941280 -1.418577
H 2.228049 3.664863 -0.591324
H 1.254529 4.978591 -1.326941
N -1.827356 -3.379358 -0.930272
N -1.928985 -2.279824 -1.009221
S -4.710319 -1.245093 -0.902757
C -6.040204 -0.019111 -0.953515
H -5.972950 0.667171 -0.107206
H -6.041679 0.521031 -1.902273
H -6.961123 -0.601254 -0.870864
H 2.071863 3.776008 -2.374775
C 1.989657 -2.548850 2.130099
H 1.920382 -2.317716 3.197519
H 2.818438 -3.253070 2.005605
S 0.426528 -3.343110 1.574464
C 2.391937 -1.244224 1.423172
H 3.263625 -0.844743 1.945837
C 2.831791 -1.407217 -0.038806
O 3.831078 -0.784001 -0.459179
N 1.308986 -0.273148 1.514114
N 2.105731 -2.194681 -0.822475
H 1.336640 -2.709475 -0.375736
C 2.368203 -2.346247 -2.241954
H 3.355156 -2.786232 -2.414781
H 1.606741 -3.005695 -2.660122
H 2.319005 -1.377475 -2.748215
C 1.403547 1.062994 1.575666
O 0.361281 1.757875 1.577037
C 2.756340 1.707276 1.651795
H 2.630775 2.778895 1.810228
H 3.348490 1.286812 2.470294
H 3.304000 1.542960 0.719826
H 0.371815 -0.661460 1.418484
H 5.154721 0.060138 0.425619
H 4.662820 -0.501480 -2.042029
H 0.365567 3.554337 1.627611
H -1.274202 1.102068 1.921480
H -1.426819 -2.279473 1.914121
O -2.352798 -1.921044 1.887979
H -2.752996 -2.205901 2.721963
O 5.968242 0.491997 0.762745
H 6.578682 0.463013 0.013192
O 5.243009 -0.235529 -2.785521
H 5.864234 0.388712 -2.385706
O 0.291490 4.531203 1.696259
H 0.277248 4.705064 2.647107
O -2.155022 0.818776 2.247025
H -2.216469 -0.142240 2.048451
O -4.218506 2.540564 1.632572
H -3.479465 1.910021 1.795192
H -4.390883 2.939533 2.496192
63
complexB_6sol_conf_6 Eopt
-2358.112121
C 3.636731 -0.256197 -0.576067
C 2.489106 -0.364059 -1.402045
C 1.555176 0.657113 -1.607274
C 1.731940 1.839785 -0.918399
C 2.845174 1.980028 -0.072230
C 3.784578 0.973070 0.077574
H 0.714718 0.492337 -2.272244
H 2.987483 2.904372 0.476409
H 4.632264 1.144481 0.728241
C 0.697689 2.902422 -1.061500
O -0.154593 2.900563 -1.933827
O 0.797331 3.834604 -0.124936
C -0.202823 4.868450 -0.132787
H 0.050736 5.516190 0.704498
H -1.191535 4.426700 0.009740
N 2.001931 -2.497069 -2.564977
N 2.237753 -1.547477 -2.047890
S 4.735720 -1.598592 -0.441136
C 6.009279 -0.989829 0.690502
H 5.585611 -0.762465 1.670440
H 6.716558 -1.817057 0.787887
H 6.525384 -0.123715 0.271835
H -0.163056 5.422000 -1.072955
C -2.046622 0.102890 -1.327586
H -1.460736 0.819130 -1.907026
H -3.098959 0.337951 -1.513846
S -1.663959 -1.595239 -1.910405
C -1.768193 0.390034 0.171502
H -1.654714 1.473181 0.278480
C -2.992566 0.024057 1.004359
O -3.966992 0.816430 1.010855
N -0.549479 -0.259234 0.598728
N -3.004921 -1.118472 1.674697
H -2.244661 -1.783021 1.531748

C -4.180583 -1.577444 2.395534
H -5.013155 -1.764780 1.710154
H -3.924342 -2.504524 2.908786
H -4.488965 -0.833342 3.133978
C 0.268779 0.127032 1.587378
O 1.313740 -0.521115 1.821329
C -0.089944 1.347403 2.384035
H 0.604013 1.451882 3.218610
H -0.023831 2.239515 1.750597
H -1.113200 1.284864 2.767210
H -0.253492 -1.050643 0.026843
H -3.852080 2.294724 -0.068069
H -5.418182 0.154191 0.152073
H 2.618223 0.171909 2.846888
H 1.557143 -2.171031 0.994811
H -1.504167 -2.916811 -0.155616
H -3.763759 -2.271056 -1.469743
O -4.691268 -2.544312 -1.275725
H -5.145389 -1.724511 -0.987582
O -1.307674 -3.361677 0.709033
H -1.857561 -4.157945 0.719414
O -3.785266 3.097799 -0.628566
H -2.897780 3.044865 -1.012011
O -6.121980 -0.249488 -0.397363
H -6.712486 -0.675499 0.239747
O 3.370284 0.512595 3.378762
H 3.179257 0.227373 4.282436
O 1.507482 -3.068970 0.608480
H 0.550650 -3.272177 0.626709
63
complexB_6sol_conf_60
Eopt -2358.103490
C 3.244766 -0.690385 0.948407
C 2.785543 -0.938150 -0.368052
C 2.468944 0.052475 -1.303361
C 2.604881 1.369374 -0.913020
C 3.033234 1.659259 0.392959
C 3.347769 0.659628 1.300205
H 2.095036 -0.230745 -2.283031
H 3.124197 2.691792 0.710573
H 3.677588 0.939709 2.292586
C 2.233823 2.432209 -1.887764
O 1.787109 2.197574 -2.998827
O 2.440927 3.654042 -1.416678
C 2.067919 4.749529 -2.270687
H 2.648337 4.718035 -3.194980
H 2.303601 5.647386 -1.701940
N 2.314301 -3.257136 -1.097736
N 2.560467 -2.232194 -0.759608
S 3.646485 -2.027358 1.992712
C 3.878336 -1.238653 3.604894
H 2.978956 -0.693800 3.898791
H 4.044265 -2.063099 4.302262
H 4.754511 -0.587633 3.605239
H 0.998905 4.702972 -2.488438
C -1.046104 -0.182304 -2.132873
H -0.312474 0.604717 -1.918210
H -1.749553 0.222479 -2.867922
S -0.210161 -1.665114 -2.809061
C -1.845852 -0.468754 -0.857889
H -2.570263 -1.256863 -1.062873
C -0.933755 -0.891403 0.292862
O -0.044788 -0.113197 0.707221
N -2.572083 0.730503 -0.445207
N -1.152603 -2.079158 0.839802
H -1.892177 -2.674201 0.454777
C -0.354402 -2.582274 1.942398
H 0.694941 -2.683756 1.650509
H -0.414262 -1.913434 2.805606
H -0.739391 -3.563077 2.223233
C -3.763302 0.770813 0.167019
O -4.184055 1.855220 0.637903
C -4.582670 -0.484390 0.255316
H -4.060775 -1.241324 0.849051
H -5.538889 -0.256391 0.726855
H -4.760378 -0.903256 -0.740290
H -2.025383 1.590907 -0.414363
H -0.026964 1.731976 0.369258
H 0.285285 0.452685 2.398738
H -5.587813 1.915245 1.762868
H -2.959231 2.830191 1.584049
H -2.056110 -2.733399 -2.936364
O -2.962665 -3.156640 -2.928874
H -2.857525 -3.977154 -3.431612
O -0.231501 2.680209 0.239328
H -0.857708 2.898136 0.962232
O 0.398674 0.924100 3.249762
H -0.317401 1.574743 3.249952
O -6.335897 1.991103 2.394613
H -6.080110 2.707892 2.990719
O -2.250964 3.257023 2.112509
H -2.406126 4.206569 2.010937
O -3.172324 -3.871954 -0.262504
H -3.109161 -3.637294 -1.218393
H -2.706593 -4.716246 -0.187112
63
complexB_6sol_conf_61
Eopt -2358.102379
C 2.965847 -0.233357 1.402162
C 2.917560 -0.168490 -0.011468
C 2.404231 0.907135 -0.744003
C 1.909307 1.983926 -0.036255
C 1.921755 1.951833 1.368092
C 2.430721 0.871608 2.071539
H 2.406047 0.881692 -1.827830
H 1.522203 2.790789 1.926756
H 2.417677 0.898553 3.153899
C 1.352446 3.132843 -0.803839
O 1.118944 3.088445 -2.000450
O 1.151404 4.202481 -0.047857

C 0.560373 5.345668 -0.689766
H 1.214020 5.707246 -1.486107
H 0.463159 6.094423 0.094519
N 3.726948 -2.114529 -1.313418
N 3.379879 -1.241263 -0.730043
S 3.682101 -1.622631 2.180059
C 3.083405 -1.489041 3.883489
H 3.405803 -2.412412 4.370984
H 3.532896 -0.638394 4.398383
H 1.992566 -1.434828 3.901811
H -0.420184 5.080880 -1.090808
C -0.692434 -0.381811 -2.247772
H -0.270377 0.547364 -1.844836
H -1.303023 -0.116364 -3.116240
S 0.648138 -1.528071 -2.745446
C -1.630849 -0.994271 -1.203205
H -2.034515 -1.926608 -1.598701
C -0.893601 -1.257139 0.111076
O -0.465646 -0.296518 0.787817
N -2.747166 -0.091946 -0.936693
N -0.762248 -2.518486 0.505736
H -0.994511 -3.252764 -0.162424
C -0.017594 -2.872431 1.701625
H 1.033852 -2.583533 1.605941
H -0.444175 -2.379056 2.578109
H -0.078042 -3.952689 1.836395
C -4.007814 -0.445839 -0.649912
O -4.833781 0.427509 -0.289864
C -4.417584 -1.883487 -0.789841
H -5.474314 -1.977849 -0.538413
H -4.258861 -2.232748 -1.815144
H -3.829018 -2.520666 -0.122537
H -2.508057 0.882005 -0.757313
H -0.610060 0.052063 2.560469
H -0.967214 1.486540 0.643402
H -4.272970 1.526427 1.047203
H -6.123547 -0.062131 0.896317
H 1.779503 -0.082599 -3.940430
O 2.291190 0.571150 -4.480890
H 1.696510 0.808575 -5.205701
O -0.767403 0.399039 3.462830
H -1.279534 1.206362 3.314736
O -1.394193 2.365008 0.590315
H -2.236842 2.256944 1.081062
O -3.906534 2.031090 1.804692
H -4.305216 2.909428 1.729102
O -6.768874 -0.257403 1.608110
H -6.393268 0.167875 2.391131
O -0.802457 -4.276489 -1.921814
H -0.108512 -4.902822 -1.672169
H -0.313517 -3.484294 -2.259085
63
complexB_6sol_conf_62
Eopt -2358.105818
C -3.463018 -1.027466 -0.816554
C -2.099538 -1.293377 -1.097858
C -1.136493 -0.316518 -1.376125
C -1.541243 1.002809 -1.366698
C -2.886064 1.309666 -1.098826
C -3.824671 0.324801 -0.841959
H -0.110586 -0.604736 -1.581559
H -3.207754 2.344941 -1.091678
H -4.848548 0.616444 -0.646096
C -0.523176 2.057738 -1.622163
O 0.630076 1.814119 -1.942172
O -0.999069 3.281487 -1.470453
C -0.059150 4.360583 -1.619180
H 0.746290 4.252195 -0.889281
H -0.631423 5.266333 -1.427947
N -1.335183 -3.650615 -1.153311
N -1.675748 -2.597893 -1.126591
S -4.551350 -2.341677 -0.469668
C -6.154107 -1.520599 -0.295686
H -6.154463 -0.837542 0.555790
H -6.430573 -1.004922 -1.217577
H -6.864906 -2.329140 -0.108700
H 0.343436 4.367547 -2.634108
C 2.498054 -1.717377 1.663461
H 2.078831 -1.638314 2.672057
H 3.485938 -2.174618 1.758229
S 1.423448 -2.755409 0.601450
C 2.699329 -0.281195 1.145555
H 3.322701 0.252174 1.870398
C 3.493323 -0.296359 -0.157665
O 4.664179 -0.743358 -0.134597
N 1.409911 0.377007 1.052920
N 2.930903 0.163883 -1.264178
H 1.982188 0.525490 -1.225875
C 3.619113 0.182110 -2.542604
H 3.860480 -0.833834 -2.867909
H 2.958987 0.643702 -3.277305
H 4.543144 0.763413 -2.477061
C 1.135273 1.674045 1.251645
O -0.054058 2.064821 1.240205
C 2.265211 2.630164 1.495931
H 1.882678 3.650995 1.468782
H 2.699708 2.440410 2.484163
H 3.058801 2.515889 0.752093
H 0.620498 -0.240642 0.868364
H 5.553502 -0.951788 1.430324
H 5.904448 -1.182566 -1.389405
H -0.572859 3.757230 1.540596
H -1.337195 0.901968 1.775403
H -0.474382 -2.466035 1.499256
O -1.407960 -2.346328 1.840669
H -1.386145 -2.687460 2.746155
O 6.035278 -1.064702 2.278110
H 6.930493 -0.740817 2.098388
O 6.657559 -1.458280 -1.952975
H 6.871893 -2.351061 -1.649999

O -0.930283 4.654811 1.715663
H -1.686244 4.505725 2.299438
O -1.989214 0.333175 2.235794
H -1.753678 -0.595438 2.012518
O -4.640982 1.074955 2.228875
H -3.697771 0.799220 2.162143
H -4.811489 1.122204 3.179413
63
complexB_6sol_conf_63
Eopt -2358.107716
C -3.444308 -1.034257 -0.821276
C -2.084616 -1.291554 -1.128405
C -1.125705 -0.305900 -1.388803
C -1.529664 1.012559 -1.334862
C -2.869161 1.311150 -1.033287
C -3.803757 0.318543 -0.792741
H -0.102565 -0.587232 -1.614923
H -3.189373 2.345839 -0.985485
H -4.823799 0.604097 -0.570822
C -0.517060 2.072767 -1.586965
O 0.639449 1.835389 -1.899433
O -1.002509 3.295661 -1.451103
C -0.071332 4.378647 -1.628296
H 0.328454 4.364254 -2.644149
H 0.736827 4.295018 -0.897987
N -1.319786 -3.645249 -1.257187
N -1.660374 -2.593802 -1.197387
S -4.531305 -2.358209 -0.512876
C -6.125675 -1.540271 -0.262255
H -6.101785 -0.894935 0.617860
H -6.424123 -0.984273 -1.153233
H -6.834841 -2.353958 -0.092569
H -0.650774 5.284584 -1.458895
C 2.496254 -1.758010 1.643873
H 2.089269 -1.692820 2.658437
H 3.482476 -2.222045 1.718732
S 1.406132 -2.775599 0.579281
C 2.698827 -0.314525 1.145274
H 3.322812 0.208047 1.877810
C 3.492772 -0.312331 -0.157872
O 4.663987 -0.759330 -0.140644
N 1.410066 0.345878 1.065184
N 2.931210 0.160488 -1.259722
H 1.983594 0.525230 -1.221217
C 3.625372 0.199195 -2.534584
H 3.868370 -0.811395 -2.875074
H 2.968544 0.672422 -3.264737
H 4.548505 0.780084 -2.455136
C 1.135696 1.640078 1.282223
O -0.054171 2.028356 1.290937
C 2.266767 2.595168 1.523849
H 2.712728 2.395410 2.504965
H 3.052447 2.490389 0.770410
H 1.882225 3.615655 1.512079
H 0.618955 -0.270228 0.882703
H 5.528823 -0.961652 1.443447
H 5.915666 -1.110165 -1.409481
H -0.584775 3.721141 1.541184
H -1.338782 0.863338 1.837463
H -0.502709 -2.487910 1.460675
O -1.440892 -2.379180 1.790043
H -1.434832 -2.755954 2.681592
O 5.996177 -1.070948 2.299611
H 5.289885 -1.283188 2.926341
O 6.657653 -1.348128 -2.004141
H 7.271223 -1.838179 -1.440673
O -0.950787 4.613175 1.727623
H -1.383210 4.874819 0.903445
O -1.987286 0.291057 2.297752
H -1.773948 -0.632483 2.034656
O -4.582609 1.231152 2.247533
H -3.663004 0.881645 2.206567
H -4.695874 1.508996 3.166650
63
complexB_6sol_conf_64
Eopt -2358.110906
C 2.362964 1.842873 0.036033
C 2.737317 0.771766 0.881828
C 2.869816 -0.559479 0.473278
C 2.616442 -0.851289 -0.850850
C 2.272297 0.188417 -1.732698
C 2.157694 1.499705 -1.306221
H 3.149040 -1.325135 1.188392
H 2.087833 -0.033583 -2.777992
H 1.884266 2.260162 -2.026000
C 2.707755 -2.270851 -1.289507
O 3.244602 -3.146283 -0.631650
O 2.120591 -2.476608 -2.461066
C 2.143502 -3.822178 -2.971221
H 1.634384 -4.493512 -2.276724
H 3.175298 -4.143912 -3.126393
N 3.257868 1.266133 3.254394
N 3.002338 1.039372 2.201704
S 2.167894 3.441871 0.696378
C 1.723735 4.437525 -0.747589
H 2.518577 4.420208 -1.495918
H 0.773431 4.106160 -1.169906
H 1.612846 5.454645 -0.364343
H 1.610031 -3.775935 -3.918913
C -1.440843 -0.526858 2.541731
H -1.715822 -1.501927 2.957044
H -2.233943 0.174985 2.823395
S 0.163429 0.012676 3.230869
C -1.448076 -0.676798 1.011962
H -0.619194 -1.328441 0.723926
C -2.772829 -1.317578 0.600339
O -3.834167 -0.650288 0.586676
N -1.280820 0.617677 0.373273
N -2.740230 -2.612842 0.320500
H -1.831152 -3.086888 0.297436

C -3.937405 -3.356637 -0.029838
H -4.393333 -2.953566 -0.939210
H -3.655928 -4.395656 -0.201918
H -4.670589 -3.313841 0.780482
C -1.091941 0.844271 -0.931178
O -1.048311 2.021286 -1.362529
C -0.971760 -0.328151 -1.862780
H -1.977183 -0.706535 -2.084074
H -0.516557 -0.001797 -2.798621
H -0.386833 -1.148790 -1.439084
H -1.419795 1.440158 0.952094
H -3.947630 1.046698 1.351724
H -4.339771 0.188224 -0.968603
H -2.635912 2.946964 -0.984703
H -0.406548 2.348263 -3.011784
H 0.973237 -1.892801 2.629507
O 1.259883 -2.808893 2.353310
H 0.930768 -3.387370 3.056277
O -4.097400 1.973290 1.630471
H -3.898773 2.485166 0.817820
O -4.624048 0.693977 -1.757825
H -5.586768 0.749575 -1.676818
O -3.587911 3.145981 -0.888672
H -4.015164 2.353590 -1.283296
O -0.055284 2.554076 -3.906000
H 0.673276 1.929181 -4.024992
O -0.115075 -3.843042 0.173209
H 0.389567 -3.421452 0.907457
H 0.188028 -3.394371 -0.628429
63
complexB_6sol_conf_65
Eopt -2358.105194
C -3.411651 -1.136310 -0.796683
C -2.037642 -1.353750 -1.067050
C -1.111202 -0.349476 -1.362021
C -1.565751 0.953868 -1.379637
C -2.920570 1.216091 -1.118326
C -3.822846 0.201266 -0.845143
H -0.074369 -0.602890 -1.556784
H -3.279907 2.239036 -1.130384
H -4.856879 0.458882 -0.654510
C -0.581818 2.035156 -1.650964
O 0.575515 1.819405 -1.977029
O -1.092164 3.246883 -1.511592
C -0.183033 4.348295 -1.686172
H -0.783857 5.242573 -1.531654
H 0.233228 4.333876 -2.695492
N -1.179531 -3.682038 -1.039575
N -1.561982 -2.643794 -1.052987
S -4.450721 -2.486614 -0.435198
C -6.080116 -1.724323 -0.243012
H -6.094877 -1.036361 0.604400
H -6.389412 -1.223808 -1.162785
H -6.758324 -2.557306 -0.042576
H 0.616662 4.286339 -0.943386
C 2.583781 -1.580136 1.713954
H 2.200730 -1.475656 2.734593
H 3.584723 -2.011380 1.788192
S 1.494409 -2.676388 0.729931
C 2.734183 -0.157085 1.144244
H 3.354784 0.418922 1.838603
C 3.505687 -0.186399 -0.172529
O 4.682751 -0.617013 -0.170213
N 1.427385 0.466326 1.054684
N 2.921902 0.250334 -1.278678
H 1.978372 0.623711 -1.230398
C 3.599921 0.248173 -2.561905
H 4.531955 0.819170 -2.510511
H 3.828257 -0.774456 -2.877058
H 2.938614 0.707829 -3.297189
C 1.122609 1.762064 1.219723
O -0.073193 2.127099 1.190972
C 2.231408 2.747136 1.445629
H 3.016197 2.647441 0.689790
H 1.824285 3.758372 1.417944
H 2.686934 2.571358 2.426768
H 0.651389 -0.174362 0.891914
H 5.707562 -1.509508 -1.383438
H 5.610215 -0.754489 1.375620
H -0.639005 3.812918 1.467721
H -1.376537 0.984958 1.748172
H -0.431975 -2.370616 1.582841
O -1.376583 -2.267415 1.891044
H -1.379427 -2.597421 2.801054
O 6.304889 -2.062012 -1.928229
H 7.172103 -1.643305 -1.844222
O 6.113909 -0.828822 2.215594
H 6.744899 -1.544325 2.059333
O -1.028228 4.698722 1.633746
H -1.955291 4.518184 1.842736
O -2.002456 0.417124 2.243722
H -1.744801 -0.510638 2.045244
O -4.683058 1.092564 2.157901
H -3.732594 0.838932 2.145485
H -4.885896 1.236551 3.092154
63
complexB_6sol_conf_66
Eopt -2358.107181
C -3.556436 -0.719875 -0.803919
C -2.241854 -1.094127 -1.179993
C -1.196840 -0.196358 -1.426912
C -1.458356 1.150002 -1.276737
C -2.746119 1.563537 -0.896806
C -3.769670 0.657883 -0.675904
H -0.218729 -0.564530 -1.717612
H -2.955237 2.620201 -0.772749
H -4.745347 1.032656 -0.394602
C -0.347795 2.112587 -1.506767
O 0.764457 1.774309 -1.880888
O -0.690489 3.368147 -1.273955

C 0.350524 4.352010 -1.415403
H 0.715599 4.363922 -2.444200
H 1.165430 4.130119 -0.722572
N -1.725154 -3.502253 -1.455095
N -1.955953 -2.426562 -1.333168
S -4.768190 -1.942914 -0.544510
C -6.226394 -0.989441 -0.056685
H -6.036810 -0.429115 0.860837
H -6.552975 -0.329850 -0.862913
H -7.000282 -1.737357 0.131342
H -0.118810 5.302071 -1.165848
C 2.309704 -2.217718 1.398170
H 1.937946 -2.171529 2.427108
H 3.245260 -2.782264 1.416444
S 1.089719 -3.050696 0.313278
C 2.645614 -0.776081 0.973742
H 3.342896 -0.362645 1.709933
C 3.396384 -0.778356 -0.354282
O 4.508264 -1.358476 -0.413283
N 1.430828 0.015926 0.974745
N 2.867355 -0.166118 -1.401471
H 1.973458 0.307847 -1.304243
C 3.519296 -0.133639 -2.698971
H 3.711391 -1.148415 -3.058444
H 2.856065 0.374271 -3.399418
H 4.467303 0.409184 -2.647116
C 1.298942 1.313911 1.282741
O 0.156897 1.820970 1.355025
C 2.529651 2.130011 1.546077
H 2.967196 1.833506 2.506361
H 3.288023 1.983186 0.772109
H 2.255423 3.184521 1.595037
H 0.575986 -0.503947 0.780307
H 5.416012 -1.665915 1.130060
H 5.910926 -0.185873 -0.737870
H -0.200670 3.539853 1.712020
H -1.221651 0.760368 1.880350
H -0.739646 -2.620456 1.307589
O -1.640866 -2.436028 1.700430
H -1.620319 -2.854287 2.573010
O 5.985169 -1.774189 1.922156
H 6.740076 -1.194375 1.752419
O 6.681182 0.412425 -0.809367
H 6.628022 0.766165 -1.707782
O -0.482311 4.447980 1.957365
H -0.924131 4.785838 1.166472
O -1.928165 0.247374 2.324783
H -1.819539 -0.683135 2.024596
O -4.319313 1.625745 2.404337
H -3.484811 1.110029 2.322541
H -4.340549 1.908634 3.328553
63
complexB_6sol_conf_67
Eopt -2358.101541
C -4.362912 0.412789 -0.116939
C -3.416703 -0.160384 -1.003952
C -2.204605 0.434914 -1.370283
C -1.899330 1.661437 -0.818323
C -2.807505 2.263754 0.068331
C -4.010264 1.663472 0.404655
H -1.528075 -0.079852 -2.043656
H -2.566894 3.224041 0.511117
H -4.674768 2.172716 1.091376
C -0.574877 2.257619 -1.146281
O 0.141075 1.839131 -2.040257
O -0.254354 3.267976 -0.351203
C 1.037040 3.867426 -0.561665
H 1.094947 4.681541 0.158779
H 1.109303 4.252441 -1.580784
N -3.917654 -2.377699 -1.991158
N -3.688141 -1.387467 -1.552204
S -5.837485 -0.440605 0.242906
C -6.703624 0.681042 1.367116
H -6.135167 0.832130 2.286824
H -6.924580 1.631618 0.877769
H -7.641072 0.172697 1.604809
H 1.820960 3.129894 -0.375563
C 0.981878 -2.643182 -0.421146
H 1.123894 -3.045586 0.588687
H 1.642488 -3.205089 -1.086368
S -0.747604 -2.894877 -0.981294
C 1.479581 -1.185239 -0.451632
H 1.173568 -0.732957 -1.399621
C 0.928267 -0.279470 0.665397
O 1.615186 0.663438 1.116572
N 2.931129 -1.177608 -0.373395
N -0.297297 -0.535158 1.102589
H -0.819579 -1.268084 0.613830
C -0.954467 0.260623 2.121718
H -1.992573 -0.066304 2.192720
H -0.467772 0.124353 3.092513
H -0.930232 1.323044 1.866032
C 3.768891 -0.364700 -1.024921
O 4.981536 -0.325543 -0.691051
C 3.255696 0.486661 -2.147980
H 2.570102 1.245961 -1.758448
H 4.095854 0.979267 -2.638425
H 2.709412 -0.113238 -2.882074
H 3.321150 -1.644481 0.449048
H 3.346423 0.732172 1.691892
H 1.297356 2.078070 2.226361
H 6.061996 0.969183 -1.311599
H 5.803501 -1.671730 0.183623
H -2.245156 -2.953614 0.692017
O -2.950109 -2.906374 1.382653
H -3.735458 -3.272668 0.952679
O 4.262347 0.687241 2.037373
H 4.792984 0.484945 1.247345
O 1.261658 2.867148 2.805797
H 1.718265 3.555792 2.303672

O 6.675844 1.671013 -1.619228
H 6.233513 2.054649 -2.388746
O 6.188467 -2.430709 0.675274
H 6.929474 -2.055185 1.170869
O 3.961106 -2.135228 2.366612
H 4.777684 -2.385240 1.884808
H 4.039173 -1.162589 2.447011
63
complexB_6sol_conf_68
Eopt -2358.107433
C 3.410270 0.856022 -0.271178
C 2.357868 0.568385 -1.176459
C 1.162420 1.289618 -1.274333
C 0.993055 2.358756 -0.417609
C 2.016538 2.686318 0.487192
C 3.196937 1.965239 0.556958
H 0.398486 0.957606 -1.974604
H 1.875612 3.524091 1.162365
H 3.948951 2.258944 1.278279
C -0.286075 3.120047 -0.381912
O -0.523769 3.996032 0.433113
O -1.137489 2.724969 -1.317291
C -2.446606 3.320420 -1.301677
H -2.366306 4.403228 -1.415753
H -2.951553 3.069142 -0.366845
N 2.523063 -1.406549 -2.663293
N 2.471061 -0.521272 -2.000210
S 4.822468 -0.161942 -0.241944
C 5.856876 0.585934 1.040871
H 5.361699 0.548727 2.012864
H 6.756490 -0.033290 1.070848
H 6.134477 1.607481 0.774455
H -2.965064 2.882664 -2.152023
C -2.215022 -1.389132 -1.678522
H -3.185867 -1.057716 -2.056174
H -2.313878 -2.449271 -1.414218
S -0.935410 -1.186864 -2.973642
C -1.945573 -0.592912 -0.385219
H -1.978993 0.477491 -0.613255
C -3.085612 -0.870061 0.587382
O -4.154071 -0.222918 0.474294
N -0.643381 -0.956668 0.144001
N -2.934330 -1.826480 1.492398
H -2.044359 -2.324183 1.568203
C -3.975946 -2.143369 2.454245
H -4.178317 -1.285138 3.102610
H -4.901181 -2.428469 1.946043
H -3.631339 -2.978787 3.064340
C 0.007819 -0.408852 1.180685
O 1.170686 -0.787294 1.455071
C -0.679081 0.624282 2.026256
H -1.149273 0.122046 2.879257
H 0.069574 1.321095 2.409447
H -1.449595 1.182322 1.489276
H -0.094498 -1.578187 -0.441941
H -4.215109 1.326984 1.502472
H -4.645784 0.674389 -1.016856
H 1.672746 -2.460916 0.712266
H 2.114727 0.032471 2.751048
H 1.142091 -4.132342 -2.559686
O 0.455328 -3.852109 -1.937290
H 0.078582 -3.016246 -2.328779
O -4.246933 2.141178 2.042796
H -4.483586 2.839229 1.416742
O -5.077637 1.097097 -1.790503
H -5.857002 0.549636 -1.954597
O 1.648064 -3.423679 0.540497
H 1.255900 -3.522786 -0.357516
O 2.656929 0.424414 3.468874
H 2.198729 0.179374 4.283859
O -0.628203 -3.532423 2.212629
H 0.138318 -3.532309 1.598009
H -0.352940 -2.945913 2.931382
63
complexB_6sol_conf_69
Eopt -2358.113367
C 2.872654 1.051924 -0.387261
C 1.888617 1.226799 -1.390660
C 0.685446 1.919872 -1.219638
C 0.445607 2.492098 0.015050
C 1.409985 2.364312 1.028973
C 2.584919 1.658579 0.841655
H -0.007556 2.000672 -2.048996
H 1.225923 2.820852 1.995016
H 3.282453 1.576985 1.665813
C -0.811690 3.229500 0.309087
O -1.018206 3.803811 1.366938
O -1.691996 3.186326 -0.682047
C -2.965771 3.816556 -0.439067
H -3.541609 3.654891 -1.348464
H -2.823793 4.883477 -0.258355
N 2.348215 0.405936 -3.684279
N 2.138551 0.745018 -2.652442
S 4.311400 0.138417 -0.730392
C 5.360474 0.465745 0.708393
H 6.312677 -0.018210 0.477473
H 5.523392 1.537360 0.839215
H 4.944446 0.017029 1.612031
H -3.452202 3.343722 0.416958
C -0.218437 -2.569883 -1.567904
H -0.682502 -2.698982 -2.550867
H -0.411652 -3.485087 -0.997538
S 1.585282 -2.326373 -1.730988
C -0.965754 -1.405454 -0.881715
H -0.932300 -0.522102 -1.527872
C -2.433672 -1.806789 -0.741204
O -3.294717 -1.323929 -1.514747
N -0.340001 -1.104461 0.396246
N -2.733460 -2.705414 0.186832
H -1.989351 -3.057073 0.798607

C -4.099157 -3.132253 0.433359
H -4.529514 -3.596033 -0.458749
H -4.086923 -3.861342 1.243972
H -4.723806 -2.282372 0.725938
C -0.779980 -0.289762 1.360108
O -0.104281 -0.127907 2.405251
C -2.103872 0.399059 1.186441
H -2.153061 1.266316 1.845729
H -2.274205 0.712204 0.153079
H -2.909888 -0.285395 1.473876
H 0.598130 -1.481952 0.509356
H -4.560166 -0.158365 -0.708362
H -2.875772 0.238765 -2.359899
H -0.444449 1.379727 3.409025
H 1.484487 -0.936772 2.633128
H 1.901837 -3.286727 0.207258
O 1.906461 -3.645767 1.134233
H 2.139278 -2.866628 1.687046
O -5.176355 0.525510 -0.381779
H -4.611453 1.296139 -0.227718
O -2.683703 1.112025 -2.763849
H -2.284344 1.624499 -2.044287
O -0.638744 2.225510 3.864474
H -0.798335 2.843809 3.131635
O 2.356310 -1.381371 2.735393
H 2.351986 -1.732385 3.636759
O -0.725242 -3.762011 2.070242
H -0.737006 -2.991205 2.656067
H 0.172804 -3.735489 1.664112
63
complexB_6sol_conf_7 Eopt
-2358.111949
C 3.164529 -1.333932 -0.357085
C 2.288194 -1.223317 -1.465979
C 1.766564 -0.019244 -1.954069
C 2.113699 1.142582 -1.296976
C 2.984860 1.077422 -0.196008
C 3.507294 -0.123584 0.255260
H 1.096477 -0.021993 -2.806240
H 3.264293 1.989048 0.319309
H 4.174776 -0.113798 1.107457
C 1.497193 2.419764 -1.748863
O 0.933915 2.555515 -2.822632
O 1.609586 3.380342 -0.843299
C 1.017502 4.650511 -1.165721
H 1.527136 5.090125 -2.025753
H 1.161820 5.265033 -0.278958
N 1.539984 -3.281898 -2.620665
N 1.884753 -2.364014 -2.106759
S 3.734402 -2.902146 0.143786
C 4.808117 -2.526796 1.550255
H 4.242797 -2.064345 2.361551
H 5.185525 -3.496938 1.882372
H 5.648211 -1.899405 1.245234
H -0.046319 4.524843 -1.377291
C -1.778428 0.273708 -1.482844
H -1.040813 0.856531 -2.043736
H -2.748227 0.440357 -1.958428
S -1.359196 -1.506827 -1.553479
C -1.850247 0.887729 -0.075821
H -2.141440 1.934796 -0.197240
C -2.963854 0.234110 0.738292
O -4.148420 0.381925 0.361302
N -0.560267 0.836277 0.583889
N -2.637072 -0.458395 1.822158
H -1.652770 -0.600988 2.012648
C -3.598847 -1.213570 2.606906
H -3.931019 -2.104710 2.066014
H -3.117159 -1.517505 3.536429
H -4.465452 -0.591362 2.840665
C -0.060268 1.752778 1.430493
O 1.022330 1.533831 2.015734
C -0.820595 3.028539 1.649550
H -1.817968 2.815623 2.049403
H -0.273874 3.651485 2.357839
H -0.946338 3.573664 0.708748
H 0.000947 -0.000451 0.436536
H -5.431487 -0.865968 0.362161
H -4.583426 1.603585 -0.902636
H 1.296232 -0.236403 2.482344
H 2.098678 2.952483 2.392198
H -0.576652 -2.285734 0.316891
H -3.147757 -2.651867 -0.807720
O -3.851050 -3.282940 -0.521765
H -4.606714 -2.720755 -0.254347
O -0.214793 -2.760394 1.110521
H -0.994178 -2.958240 1.650223
O -6.044281 -1.624366 0.245914
H -6.479936 -1.460691 -0.602312
O -4.860246 2.273306 -1.564409
H -5.746752 2.540513 -1.286706
O 1.333813 -1.162407 2.793449
H 0.786582 -1.666401 2.152747
O 2.717352 3.690833 2.575382
H 3.229809 3.393915 3.339396
63
complexB_6sol_conf_70
Eopt -2358.100883
C -2.719748 -1.143516 0.808216
C -2.906397 -0.315476 -0.323510
C -2.544884 1.034121 -0.395741
C -1.965950 1.600507 0.722491
C -1.764965 0.815075 1.868710
C -2.133076 -0.517887 1.916240
H -2.715302 1.592039 -1.309401
H -1.300899 1.262550 2.741033
H -1.954254 -1.077744 2.825247
C -1.497894 3.013005 0.723442
O -0.760309 3.473288 1.578815
O -1.962761 3.708678 -0.306189

C -1.478346 5.054849 -0.450549
H -1.771124 5.653383 0.414425
H -1.951980 5.432928 -1.354876
N -4.153076 -1.210846 -2.270836
N -3.549726 -0.835443 -1.422340
S -3.215606 -2.812120 0.751331
C -2.950627 -3.382866 2.447920
H -3.524781 -2.785455 3.158993
H -3.322469 -4.410156 2.458938
H -1.888498 -3.385420 2.698852
H -0.391599 5.049000 -0.560859
C 0.416565 -1.985930 -1.004815
H -0.091818 -1.722760 -0.067223
H 0.787028 -3.010041 -0.893450
S -0.756487 -1.907046 -2.405812
C 1.654135 -1.083253 -1.160808
H 2.061934 -1.220543 -2.163549
C 1.291081 0.385928 -0.935323
O 1.105014 0.815583 0.225616
N 2.669991 -1.488904 -0.195307
N 1.200875 1.161776 -2.008753
H 1.283354 0.722794 -2.927429
C 0.783273 2.551668 -1.946858
H 1.213325 3.032443 -1.066264
H 1.141603 3.060930 -2.842640
H -0.306845 2.632330 -1.904721
C 3.990479 -1.285292 -0.290774
O 4.745683 -1.606161 0.659315
C 4.548499 -0.678772 -1.544830
H 4.233637 -1.235513 -2.432564
H 4.195816 0.352717 -1.649786
H 5.637232 -0.678714 -1.486069
H 2.336297 -1.813150 0.708877
H 1.048288 -0.201761 1.773372
H 2.660125 1.512925 0.999934
H 4.242460 -1.055820 2.290633
H 6.112718 -0.412322 0.968131
H 0.439381 -0.922649 -3.936349
O 1.042139 -0.396203 -4.529525
H 0.447007 0.156593 -5.055464
O 1.099385 -0.732202 2.595373
H 2.039643 -0.687469 2.853664
O 3.485511 1.775341 1.450115
H 3.668669 1.029550 2.057068
O 3.879468 -0.535340 3.040084
H 4.573464 -0.512910 3.713504
O 6.756193 0.281368 1.220851
H 6.199140 1.014642 1.517105
O -5.647043 0.515459 1.548996
H -6.233233 1.069409 2.082967
H -4.770408 0.907318 1.669776
63
complexB_6sol_conf_71
Eopt -2358.108851
C 3.118940 1.034709 0.552868
C 2.671947 0.650484 -0.735214
C 1.616006 1.252981 -1.428490
C 0.947861 2.288221 -0.806423
C 1.336967 2.677781 0.486055
C 2.390155 2.068993 1.150603
H 1.339307 0.881822 -2.411577
H 0.807468 3.480820 0.985662
H 2.650760 2.416595 2.142269
C -0.177403 2.938299 -1.534477
O -0.563694 2.576759 -2.633827
O -0.699114 3.956134 -0.864422
C -1.799582 4.644804 -1.483530
H -2.078978 5.426147 -0.778873
H -2.630672 3.954718 -1.640785
N 3.693197 -1.277404 -1.907053
N 3.278506 -0.403489 -1.368651
S 4.502968 0.248660 1.263315
C 4.541412 0.917417 2.944125
H 3.609655 0.697031 3.468683
H 5.364444 0.394405 3.436879
H 4.750575 1.988663 2.938860
H -1.480335 5.080957 -2.432367
C -0.965961 -1.119715 -2.061749
H -0.860660 -0.059276 -1.801870
H -1.882211 -1.214287 -2.653787
S 0.469565 -1.678888 -3.049697
C -1.196897 -1.911124 -0.764399
H -1.249638 -2.974966 -1.008027
C -0.066573 -1.660846 0.236939
O -0.000997 -0.568007 0.840543
N -2.473968 -1.492993 -0.189022
N 0.798380 -2.650952 0.420856
H 0.713364 -3.469288 -0.183400
C 1.966844 -2.569384 1.276742
H 2.005768 -1.589636 1.751358
H 1.917760 -3.337786 2.053325
H 2.874030 -2.723021 0.685716
C -3.102799 -2.063205 0.844062
O -4.152488 -1.555672 1.314661
C -2.534397 -3.306665 1.463445
H -1.743003 -3.019950 2.166235
H -3.321825 -3.815255 2.021563
H -2.105485 -3.991241 0.727956
H -2.844789 -0.593247 -0.508985
H 0.353754 -0.141321 2.563711
H -1.107196 0.861624 0.923382
H -5.158194 -0.235629 0.646734
H -3.763794 -0.184181 2.505405
H 0.697612 -3.716251 -2.306994
O 0.774790 -4.595096 -1.844876
H 1.719302 -4.803605 -1.872101
O 0.380131 0.193941 3.483370
H -0.383864 0.785779 3.531835
O -1.694083 1.622894 1.119349
H -2.308584 1.278750 1.803756

O -5.687274 0.518711 0.302320
H -5.751561 1.132332 1.047797
O -3.487537 0.600064 3.020259
H -4.243600 1.201729 2.967493
O -3.371019 1.278477 -1.079675
H -2.760446 1.533751 -0.352410
H -4.231147 1.129176 -0.633215
63
complexB_6sol_conf_72
Eopt -2358.109088
C 2.816499 -1.273544 -0.234001
C 2.538235 -0.514244 -1.396178
C 2.473655 0.883493 -1.442590
C 2.704852 1.572537 -0.268331
C 2.979235 0.856141 0.908735
C 3.030028 -0.526445 0.930872
H 2.243536 1.383225 -2.376711
H 3.157819 1.401376 1.829248
H 3.251433 -1.027098 1.864562
C 2.632058 3.058719 -0.207428
O 2.860941 3.698203 0.805644
O 2.278627 3.604360 -1.363371
C 2.115098 5.033242 -1.381286
H 1.331121 5.325148 -0.679111
H 1.822443 5.273542 -2.401884
N 2.138276 -1.698254 -3.536513
N 2.304512 -1.176536 -2.574636
S 2.886225 -3.008583 -0.325261
C 2.893465 -3.507647 1.413957
H 2.790801 -4.595039 1.396617
H 3.833282 -3.242698 1.901919
H 2.039920 -3.074922 1.940095
H 3.057582 5.522427 -1.127384
C -2.057798 -1.274312 -0.754188
H -3.033752 -1.377321 -1.237724
H -2.114860 -1.857812 0.172693
S -0.764474 -1.931123 -1.866931
C -1.873925 0.208951 -0.377759
H -1.505384 0.754479 -1.249753
C -3.261525 0.773020 -0.084062
O -3.981349 1.110809 -1.054848
N -0.931142 0.349771 0.716506
N -3.685653 0.814840 1.172266
H -3.056818 0.544486 1.931555
C -5.017409 1.281306 1.520567
H -5.160171 2.318811 1.204827
H -5.781996 0.659129 1.047129
H -5.126651 1.219540 2.603451
C -0.436980 1.516579 1.163068
O 0.185667 1.572471 2.247188
C -0.652729 2.752253 0.333920
H -0.303960 2.603267 -0.693222
H -1.717003 3.008675 0.288212
H -0.113203 3.584706 0.787266
H -0.801654 -0.464763 1.324097
H -5.257638 -0.180504 -1.435998
H -3.270248 0.502283 -2.820174
H 0.928323 0.226771 3.177675
H -1.398153 0.731375 3.509808
H -0.347310 -3.403949 -0.297224
O -0.351134 -3.976255 0.514306
H -1.246070 -4.344466 0.540491
O -5.844111 -0.915705 -1.702750
H -5.259769 -1.511995 -2.191525
O -2.859317 0.122053 -3.621131
H -2.172696 -0.485051 -3.276599
O 1.305242 -0.505578 3.715568
H 0.843976 -0.440236 4.563763
O -2.132077 0.108922 3.646723
H -1.766299 -0.739513 3.330351
O -0.664662 -2.055060 2.509370
H 0.191456 -1.735477 2.862919
H -0.467206 -2.725907 1.816988
63
complexB_6sol_conf_73
Eopt -2358.101642
C 3.676499 -0.620948 -0.320332
C 2.582274 -0.908247 -1.173019
C 1.637343 0.029828 -1.606280
C 1.786483 1.331910 -1.174373
C 2.859013 1.659707 -0.326931
C 3.781133 0.713905 0.088142
H 0.828409 -0.275383 -2.261269
H 2.971497 2.679943 0.022178
H 4.586310 1.020660 0.743536
C 0.786490 2.343965 -1.615210
O -0.187678 2.072757 -2.297988
O 1.073191 3.563345 -1.185917
C 0.141691 4.606687 -1.519935
H 0.061051 4.702786 -2.604618
H 0.561063 5.512725 -1.086435
N 2.304366 -3.208755 -2.049191
N 2.438740 -2.184864 -1.651188
S 4.765605 -1.897751 0.144600
C 6.078722 -1.028083 1.034866
H 5.701127 -0.583346 1.956944
H 6.810010 -1.801571 1.281378
H 6.549118 -0.277080 0.397183
H -0.834198 4.388584 -1.081899
C -2.086708 -1.111747 -1.839196
H -1.551412 -0.396424 -2.471640
H -2.972772 -1.432579 -2.396756
S -1.012805 -2.569983 -1.519799
C -2.584941 -0.320480 -0.620800
H -3.115064 0.552220 -1.006278
C -3.598377 -1.041350 0.276895
O -4.544693 -0.391219 0.780715
N -1.465186 0.146052 0.186701
N -3.411690 -2.330713 0.519729
H -2.630167 -2.784909 0.032165

C -4.294974 -3.107588 1.370915
H -4.405283 -2.629037 2.347538
H -5.283894 -3.216109 0.915945
H -3.855727 -4.096387 1.505320
C -1.274378 1.377774 0.679506
O -0.208363 1.645627 1.281353
C -2.342431 2.418531 0.507057
H -2.022118 3.338219 0.997544
H -2.518527 2.621689 -0.554175
H -3.284119 2.080172 0.948258
H -0.687984 -0.504594 0.280328
H -6.255608 -0.905732 1.137878
H -5.257561 1.045426 -0.062061
H 0.299176 3.335582 1.650811
H 0.845197 0.320243 1.905360
H 0.314251 -2.580721 0.217702
O 1.020987 -2.729322 0.897251
H 0.612790 -3.317406 1.548998
O -7.215700 -1.036266 1.283113
H -7.624765 -0.253758 0.888710
O -5.741510 1.775329 -0.504441
H -6.667606 1.498446 -0.481137
O 0.634091 4.229371 1.880518
H 0.387969 4.350296 2.807523
O 1.344601 -0.391609 2.358381
H 1.264218 -1.182347 1.780415
O 3.677417 0.579054 3.459286
H 3.481474 0.506087 4.403120
H 2.878969 0.216296 3.012267
63
complexB_6sol_conf_74
Eopt -2358.103563
C 1.903299 -1.670140 -0.670755
C 2.564780 -0.773659 -1.545399
C 3.826288 -0.217138 -1.312146
C 4.470813 -0.556786 -0.139824
C 3.843717 -1.434044 0.761335
C 2.596106 -1.979535 0.505071
H 4.265474 0.462913 -2.034029
H 4.344675 -1.703924 1.684134
H 2.162513 -2.658778 1.228169
C 5.808425 0.043870 0.124933
O 6.367563 0.802650 -0.649670
O 6.320103 -0.337331 1.287136
C 7.613647 0.192004 1.629513
H 8.351720 -0.119956 0.887933
H 7.846375 -0.234063 2.603813
N 1.422794 -0.114813 -3.643399
N 1.937505 -0.416808 -2.712133
S 0.348533 -2.314763 -1.116006
C -0.172672 -3.211563 0.366426
H -1.203972 -3.513515 0.166951
H 0.441631 -4.099977 0.524038
H -0.150476 -2.558133 1.240071
H 7.566777 1.281187 1.687987
C -1.380239 1.075130 -1.489025
H -2.218524 1.638788 -1.913584
H -1.198670 0.227404 -2.159265
S 0.103986 2.139411 -1.433659
C -1.813566 0.481594 -0.140341
H -0.971666 -0.069959 0.283489
C -2.205690 1.548838 0.888545
O -1.521074 1.720287 1.921613
N -2.919267 -0.440533 -0.394673
N -3.299986 2.259874 0.636253
H -3.859556 2.052584 -0.196618
C -3.800502 3.255043 1.568514
H -4.005197 2.804064 2.544209
H -3.078082 4.065948 1.700249
H -4.725845 3.665604 1.163961
C -3.466576 -1.314977 0.461433
O -4.461479 -1.995209 0.109111
C -2.915259 -1.440260 1.850141
H -3.482916 -0.762842 2.499901
H -3.063083 -2.461876 2.205068
H -1.860696 -1.174480 1.926975
H -3.407691 -0.328336 -1.277973
H -0.624938 3.611028 1.662748
H -0.222853 0.514196 2.405950
H -5.579510 -1.217944 -1.064087
H -5.210842 -3.103535 1.323888
H 1.465514 1.639165 0.187948
O 2.125704 1.586997 0.928210
H 1.911295 2.356301 1.476401
O -0.216809 4.200792 1.006521
H -0.154190 3.617303 0.211121
O 0.560759 -0.043370 2.598183
H 1.239166 0.350986 2.011070
O -6.192736 -0.699526 -1.631670
H -6.023708 -1.012431 -2.531283
O -5.637192 -3.699202 1.977587
H -5.257731 -3.428775 2.825913
O -5.073344 1.858104 -1.646409
H -5.468003 0.956310 -1.641280
H -4.462001 1.852552 -2.396969
63
complexB_6sol_conf_75
Eopt -2358.114132
C 3.544912 -0.397047 0.115572
C 3.148535 0.895422 -0.308106
C 2.272942 1.731333 0.391616
C 1.762312 1.270047 1.587533
C 2.159009 0.007487 2.062170
C 3.023089 -0.806206 1.348049
H 2.013142 2.702055 -0.013250
H 1.766038 -0.356092 3.004605
H 3.277827 -1.777134 1.753724
C 0.752287 2.099808 2.297022
O 0.216427 3.081975 1.803090
O 0.479979 1.649306 3.509642

C -0.558380 2.327552 4.239384
H -1.491947 2.294460 3.674043
H -0.263931 3.361223 4.431024
N 3.951510 1.741630 -2.493782
N 3.595980 1.363125 -1.516042
S 4.602675 -1.349638 -0.887796
C 4.684724 -2.932811 -0.016997
H 5.168379 -2.823239 0.955360
H 3.688988 -3.369287 0.080842
H 5.299130 -3.573858 -0.653601
H -0.655454 1.777028 5.173269
C -1.188128 0.555278 -2.468690
H -1.927012 1.215561 -2.933146
H -1.281227 -0.424091 -2.954413
S 0.492351 1.230389 -2.725678
C -1.600328 0.394799 -0.993865
H -1.513832 1.364205 -0.493748
C -3.057769 -0.068056 -0.957911
O -3.352540 -1.282906 -1.036092
N -0.742941 -0.573633 -0.335365
N -3.975233 0.886180 -0.879810
H -3.664380 1.863151 -0.905451
C -5.396725 0.594266 -0.944026
H -5.941723 1.536766 -0.888059
H -5.650766 0.087757 -1.880131
H -5.697238 -0.041402 -0.106568
C -0.823238 -0.967913 0.941595
O -0.141164 -1.934673 1.356401
C -1.754857 -0.243847 1.871768
H -2.758945 -0.675179 1.782466
H -1.412229 -0.389974 2.897354
H -1.824602 0.825458 1.656548
H -0.119252 -1.106173 -0.934181
H -2.209402 -2.707147 -1.371373
H -4.143508 -2.122446 0.370540
H -1.340233 -3.434933 1.493196
H 0.770626 -2.940688 0.048540
H -0.000196 2.951495 -1.506715
O -0.322091 3.725394 -0.965914
H -0.150775 3.469800 -0.040561
O -1.805886 -3.599600 -1.415636
H -1.910322 -3.930550 -0.498026
O -4.427558 -2.661430 1.138755
H -5.040750 -3.311109 0.766779
O -2.039304 -4.103648 1.364323
H -2.876753 -3.588280 1.377111
O 0.954252 -3.427427 -0.780567
H 0.060713 -3.492168 -1.178994
O -3.066773 3.623871 -1.267053
H -3.180381 3.636445 -2.227698
H -2.088666 3.637661 -1.135195
63
complexB_6sol_conf_76
Eopt -2358.112646
C 3.209767 0.974368 -0.200253
C 2.254319 0.592795 -1.173968
C 1.033452 1.239109 -1.393583
C 0.727399 2.319877 -0.591675
C 1.642771 2.727584 0.394092
C 2.855034 2.082737 0.579150
H 0.349600 0.851456 -2.147195
H 1.403570 3.571131 1.032177
H 3.524243 2.442336 1.350300
C -0.587481 2.985747 -0.793611
O -1.310097 2.754941 -1.752212
O -0.890084 3.837874 0.171441
C -2.134517 4.552150 0.043107
H -2.967184 3.849426 -0.019813
H -2.104704 5.186788 -0.844895
N 2.613633 -1.419459 -2.575628
N 2.491694 -0.514375 -1.949343
S 4.688266 0.069360 -0.039258
C 5.605315 0.983595 1.224437
H 5.087851 0.951712 2.184733
H 6.560610 0.460513 1.312202
H 5.786186 2.011845 0.904814
H -2.209323 5.156250 0.945367
C -2.095881 -1.541629 -1.825027
H -3.026598 -1.157103 -2.251004
H -2.270364 -2.590403 -1.554550
S -0.748471 -1.436214 -3.060724
C -1.839328 -0.746063 -0.528127
H -1.797032 0.321313 -0.769249
C -3.037540 -0.941917 0.393781
O -4.045398 -0.209020 0.246045
N -0.584849 -1.171973 0.064935
N -2.997717 -1.915334 1.292121
H -2.142813 -2.463167 1.419159
C -4.098080 -2.154636 2.209916
H -3.824053 -2.986574 2.859200
H -4.290739 -1.269182 2.823428
H -5.010247 -2.411753 1.664304
C 0.047740 -0.624448 1.113898
O 1.192355 -1.024116 1.427022
C -0.651899 0.429969 1.920366
H -1.312024 1.052604 1.312957
H -1.255872 -0.059215 2.693324
H 0.091625 1.060613 2.410402
H -0.028279 -1.804016 -0.504334
H -4.027219 1.084812 -1.043866
H -3.994081 1.216395 1.471779
H 1.574956 -2.768647 0.871452
H 2.123236 -0.191565 2.727429
H 0.144635 -3.265850 -2.292109
O 0.471555 -4.101952 -1.860386
H -0.332729 -4.606900 -1.672890
O -4.061046 1.802405 -1.711516
H -3.163169 2.177025 -1.709933
O -3.955829 1.965126 2.097926
H -3.125527 2.413218 1.884181

O 1.504242 -3.731730 0.710651
H 1.164988 -3.814985 -0.208980
O 2.658394 0.226021 3.437035
H 2.772091 -0.469987 4.098304
O -0.855909 -3.690709 2.232545
H -0.052241 -3.758556 1.669183
H -0.597343 -3.078011 2.935807
63
complexB_6sol_conf_77
Eopt -2358.104212
C -3.706004 -0.283211 -0.842930
C -2.366264 -0.665760 -1.104527
C -1.296727 0.225051 -1.248376
C -1.558667 1.571888 -1.102152
C -2.873584 1.994221 -0.844833
C -3.921340 1.095804 -0.730140
H -0.296942 -0.148871 -1.446009
H -3.084473 3.052115 -0.733576
H -4.916861 1.476772 -0.539173
C -0.420797 2.525595 -1.197090
O 0.692787 2.205434 -1.582719
O -0.743315 3.752397 -0.823664
C 0.316775 4.725730 -0.831392
H 0.697236 4.854426 -1.846659
H 1.118758 4.406011 -0.161922
N -1.798932 -3.071989 -1.298468
N -2.066851 -2.000957 -1.219290
S -4.941691 -1.499804 -0.685647
C -6.426577 -0.543097 -0.295923
H -6.299035 0.015375 0.633720
H -6.696894 0.118401 -1.121237
H -7.213869 -1.288816 -0.161157
H -0.138488 5.647141 -0.473491
C 2.393601 -2.028779 1.301728
H 2.040064 -2.086910 2.336768
H 3.339793 -2.573091 1.251817
S 1.166206 -2.780218 0.164343
C 2.693470 -0.546501 1.009285
H 3.398034 -0.186490 1.765809
C 3.411425 -0.410801 -0.330680
O 4.530730 -0.960257 -0.459118
N 1.463287 0.215233 1.103957
N 2.847102 0.285838 -1.305466
H 1.933449 0.705055 -1.157081
C 3.467971 0.447965 -2.608138
H 4.463426 0.889656 -2.508949
H 3.555309 -0.514728 -3.120244
H 2.841000 1.112765 -3.203091
C 1.307451 1.480240 1.516326
O 0.156182 1.961488 1.624537
C 2.523121 2.292271 1.852732
H 2.232198 3.334323 1.990263
H 2.963014 1.921749 2.785866
H 3.285960 2.224202 1.072062
H 0.618574 -0.302251 0.866361
H 5.510144 -1.416290 0.978678
H 5.582943 -1.394220 -1.870310
H -0.185685 3.640339 2.178715
H -1.299535 0.945449 1.871728
H -0.533719 -2.391296 1.418244
O -1.295728 -2.264550 2.045146
H -1.566817 -1.323383 1.963689
O 6.045573 -1.665592 1.763751
H 6.941011 -1.791459 1.421792
O 6.235787 -1.688118 -2.540256
H 6.379851 -2.623657 -2.343488
O -0.445228 4.531425 2.499092
H -0.866404 4.368286 3.354284
O -2.094767 0.414358 2.101202
H -2.177835 0.505841 3.060861
O -3.116239 -4.226344 1.405705
H -2.824370 -4.965611 1.956024
H -2.493683 -3.492994 1.622674
63
complexB_6sol_conf_78
Eopt -2358.105122
C -3.530959 0.493011 -0.186908
C -2.731326 0.942228 0.893747
C -1.676504 1.856881 0.783619
C -1.405720 2.370722 -0.468279
C -2.177047 1.953573 -1.565918
C -3.207925 1.038823 -1.435715
H -1.110379 2.133521 1.664473
H -1.953704 2.353647 -2.549411
H -3.762829 0.746226 -2.318010
C -0.306535 3.351206 -0.684787
O -0.125704 3.930789 -1.742690
O 0.452225 3.516042 0.390278
C 1.552412 4.434916 0.270353
H 2.043421 4.420101 1.241350
H 1.178655 5.435851 0.044503
N -3.256966 0.110863 3.167754
N -3.010795 0.478994 2.153160
S -4.812309 -0.650988 0.103120
C -5.594918 -0.824143 -1.518468
H -6.421449 -1.520609 -1.358587
H -5.991950 0.132125 -1.864584
H -4.901020 -1.250936 -2.244672
H 2.235414 4.097759 -0.512021
C 1.461338 -1.276855 2.007950
H 2.349952 -1.329723 2.643928
H 1.195586 -2.308736 1.744955
S 0.101765 -0.477658 2.933624
C 1.875879 -0.541622 0.720918
H 2.012878 0.515475 0.962582
C 3.246262 -1.065245 0.287879
O 4.272713 -0.549523 0.791060
N 0.860357 -0.683277 -0.308827
N 3.303081 -2.089789 -0.553091
H 2.447132 -2.447107 -0.984261

C 4.564197 -2.660760 -0.994994
H 4.345738 -3.558090 -1.574536
H 5.115014 -1.955617 -1.624706
H 5.185034 -2.931107 -0.137111
C 0.856176 -0.037229 -1.485543
O 0.029857 -0.331454 -2.378234
C 1.885218 1.031969 -1.730880
H 2.052449 1.667030 -0.857300
H 2.843114 0.562688 -1.986440
H 1.566461 1.644425 -2.575243
H 0.204148 -1.463849 -0.213076
H 5.918239 -0.290399 0.046420
H 4.269119 1.257020 1.042933
H -1.364934 -1.415386 -2.355210
H 0.831228 -2.611906 -2.591820
H -1.282368 -2.022527 2.615336
O -1.915659 -2.760056 2.367718
H -1.638801 -3.516101 2.904792
O 6.804285 -0.030275 -0.280953
H 6.899548 0.892342 -0.007406
O 4.346606 2.231900 1.111761
H 5.227501 2.426331 0.763608
O -2.128773 -2.037301 -2.349571
H -1.881984 -2.729245 -2.979397
O 1.137576 -3.382512 -2.088751
H 0.486971 -3.452962 -1.361993
O -0.881682 -3.174592 -0.155306
H -1.518446 -2.798214 -0.799191
H -1.275627 -3.050198 0.738878
63
complexB_6sol_conf_79
Eopt -2358.113998
C 3.224198 0.951083 -0.202879
C 2.248378 0.568887 -1.155483
C 1.037431 1.234504 -1.372136
C 0.762116 2.336292 -0.587660
C 1.697992 2.745750 0.377297
C 2.900941 2.082106 0.557439
H 0.336219 0.845770 -2.109880
H 1.483931 3.606166 1.001709
H 3.589656 2.446723 1.309401
C -0.544890 3.019314 -0.782776
O -1.281634 2.784214 -1.729567
O -0.822184 3.891205 0.171546
C -2.056444 4.624013 0.045253
H -2.902147 3.934347 0.025494
H -2.035294 5.226989 -0.864733
N 2.512605 -1.485413 -2.516386
N 2.445042 -0.562800 -1.907410
S 4.689338 0.024758 -0.043724
C 5.640039 0.951504 1.185524
H 5.144129 0.943723 2.157790
H 6.591382 0.419792 1.263899
H 5.825604 1.971872 0.843983
H -2.099168 5.260457 0.927097
C -2.157964 -1.463553 -1.811854
H -3.077567 -1.032739 -2.216949
H -2.371596 -2.509752 -1.560087
S -0.822985 -1.387346 -3.064285
C -1.850128 -0.706273 -0.503336
H -1.772236 0.363780 -0.723312
C -3.036795 -0.874666 0.438518
O -4.020925 -0.105209 0.319233
N -0.602750 -1.192544 0.056571
N -3.014238 -1.859394 1.324987
H -2.179872 -2.443436 1.426763
C -4.105582 -2.070165 2.260547
H -4.254978 -1.185486 2.887101
H -5.036341 -2.288407 1.729569
H -3.849375 -2.918600 2.895595
C 0.074704 -0.692976 1.100547
O 1.201795 -1.157620 1.387627
C -0.550742 0.389179 1.930922
H 0.236858 0.971885 2.411967
H -1.188572 1.055532 1.345667
H -1.164425 -0.075646 2.711343
H -0.092157 -1.850252 -0.526447
H -3.873371 1.314357 1.544594
H -4.008390 1.172305 -0.983687
H 2.186562 -0.426575 2.691344
H 1.494891 -2.905996 0.797566
H -0.025389 -3.273786 -2.348324
O 0.257984 -4.135114 -1.935647
H -0.572610 -4.585000 -1.724316
O -3.790560 2.059094 2.171126
H -2.954573 2.481949 1.930049
O -4.041165 1.885854 -1.655852
H -3.138335 2.248552 -1.665623
O 2.752066 -0.091351 3.421331
H 2.997256 0.802500 3.145974
O 1.377811 -3.860597 0.613816
H 1.015545 -3.902457 -0.299662
O -0.941001 -3.738256 2.188192
H -0.153137 -3.838056 1.607568
H -0.635769 -3.155827 2.898370
63
complexB_6sol_conf_8 Eopt
-2358.102408
C 2.506116 -0.889459 1.235356
C 3.141674 -0.870332 -0.029875
C 3.448401 0.287234 -0.752142
C 3.127034 1.502691 -0.182340
C 2.518330 1.529329 1.084752
C 2.211020 0.369046 1.774474
H 3.921072 0.214179 -1.725532
H 2.271286 2.481412 1.539697
H 1.724743 0.447751 2.738963
C 3.435334 2.742403 -0.947730
O 3.974300 2.738907 -2.042414
O 3.056244 3.843454 -0.312897

C 3.301598 5.093731 -0.981553
H 2.767103 5.118074 -1.933212
H 4.373416 5.227285 -1.141304
N 3.841888 -3.025333 -1.032948
N 3.505045 -2.065163 -0.598110
S 2.154350 -2.413093 1.997137
C 1.427084 -1.936357 3.582050
H 2.134901 -1.357976 4.179363
H 0.490762 -1.392978 3.440773
H 1.218225 -2.881053 4.090278
H 2.917055 5.856953 -0.307291
C -0.114499 -0.352121 -1.550313
H 0.532901 0.156885 -0.825664
H -0.224346 0.315504 -2.410779
S 0.639130 -1.933163 -2.086015
C -1.507751 -0.528686 -0.933389
H -2.097767 -1.182386 -1.575856
C -1.416602 -1.118177 0.475611
O -0.935960 -0.434501 1.404905
N -2.190317 0.758122 -0.848749
N -1.887783 -2.346121 0.661096
H -2.145221 -2.889878 -0.163692
C -1.810178 -3.026612 1.941928
H -2.196148 -2.385672 2.737332
H -2.418602 -3.929847 1.886406
H -0.779461 -3.307791 2.180127
C -3.493460 0.985149 -1.057638
O -3.974405 2.119362 -0.816977
C -4.352903 -0.119113 -1.602717
H -3.976408 -0.455332 -2.574694
H -4.356109 -0.979041 -0.925722
H -5.372132 0.249415 -1.721306
H -1.666975 1.523979 -0.428755
H -0.572954 1.398894 1.442751
H -1.574826 -0.087284 3.080966
H -5.688693 2.223966 -0.230766
H -3.546687 2.809495 0.814644
H -0.757147 -1.731252 -3.812739
H -1.094359 -3.347660 -1.982525
O -1.921659 -3.882645 -1.875458
H -1.621139 -4.727676 -1.512127
O -1.445261 -1.525833 -4.492600
H -2.246707 -1.371465 -3.972050
O -0.590707 2.376669 1.442229
H -1.530123 2.604272 1.611386
O -1.896477 0.335057 3.903385
H -1.959271 1.271215 3.666120
O -6.578482 2.329555 0.167659
H -6.401970 2.639206 1.066586
O -3.304049 3.066480 1.730013
H -3.297467 4.033959 1.720050
63
complexB_6sol_conf_80
Eopt -2358.101956
C 3.564064 -0.400895 0.038678
C 2.592751 -0.499481 -0.987796
C 1.821975 0.566953 -1.464954
C 1.994272 1.799154 -0.867064
C 2.913957 1.931324 0.187100
C 3.690399 0.868020 0.617692
H 1.100547 0.393238 -2.261261
H 3.039435 2.893707 0.671256
H 4.399906 1.030002 1.419270
C 1.189622 2.945659 -1.373388
O 0.667739 2.961100 -2.475400
O 1.096027 3.932892 -0.493815
C 0.339070 5.088198 -0.898989
H -0.680787 4.796441 -1.156210
H 0.823046 5.567662 -1.752339
N 2.190691 -2.677519 -2.101414
N 2.380914 -1.710688 -1.597376
S 4.500452 -1.797953 0.487470
C 5.709351 -1.136920 1.660299
H 5.224434 -0.782734 2.571344
H 6.352718 -1.985568 1.905035
H 6.311039 -0.350525 1.200313
H 0.342947 5.749466 -0.034452
C -1.779540 0.476801 -1.663613
H -0.999310 1.241088 -1.604072
H -2.658505 0.965018 -2.099837
S -1.215739 -0.891351 -2.756798
C -2.132642 0.119473 -0.214379
H -2.313120 1.055529 0.315623
C -3.419168 -0.685835 -0.014472
O -4.081691 -0.525269 1.038119
N -1.057858 -0.585887 0.472139
N -3.771105 -1.545169 -0.958343
H -3.142613 -1.631547 -1.764817
C -4.957344 -2.376747 -0.860958
H -5.857744 -1.759236 -0.803122
H -5.009663 -3.000650 -1.753433
H -4.910197 -3.018783 0.023370
C -0.156646 -0.070161 1.318568
O 0.786663 -0.785519 1.728300
C -0.314884 1.347705 1.784875
H -0.378085 2.032540 0.935626
H -1.231332 1.451448 2.375124
H 0.543565 1.620645 2.399534
H -0.854864 -1.520205 0.132780
H -3.945616 1.008671 1.959227
H -5.683406 -1.126382 1.654309
H 1.954742 -0.216358 2.979816
H 1.020683 -2.462064 1.117585
H -0.871109 -2.861819 -1.702297
O -0.753525 -3.751833 -1.296127
H -0.094770 -3.617804 -0.580285
O -3.914216 1.861301 2.448549
H -4.841022 2.118257 2.553686
O -6.528294 -1.361851 2.091221
H -6.448942 -2.307015 2.278519

O 2.600230 0.025557 3.678469
H 2.128275 -0.120864 4.509455
O 1.126288 -3.368702 0.750646
H 0.811227 -3.957708 1.450375
O -2.984102 3.659991 0.484583
H -2.068245 3.420278 0.287073
H -3.269510 3.018130 1.171043
63
complexB_6sol_conf_81
Eopt -2358.094439
C -4.017181 0.148653 -0.615550
C -2.898833 -0.448918 -1.251019
C -1.698902 0.209660 -1.537599
C -1.589936 1.534199 -1.166783
C -2.672176 2.163435 -0.531086
C -3.858524 1.495142 -0.271690
H -0.884284 -0.336582 -2.003230
H -2.589712 3.202976 -0.233968
H -4.662710 2.029725 0.217307
C -0.307204 2.235411 -1.449867
O 0.542756 1.799106 -2.207782
O -0.197956 3.381914 -0.794168
C 1.000981 4.144988 -1.016345
H 0.894175 5.031454 -0.393679
H 1.075185 4.422474 -2.069921
N -3.091888 -2.827193 -1.924461
N -2.987356 -1.765775 -1.625483
S -5.446596 -0.799895 -0.310264
C -6.556456 0.366184 0.515525
H -7.462050 -0.208631 0.723989
H -6.127536 0.714523 1.456944
H -6.805502 1.203054 -0.139775
H 1.875488 3.567047 -0.712433
C 1.861155 -2.621404 -0.876629
H 2.221772 -3.303761 -0.096877
H 2.437593 -2.838262 -1.781679
S 0.087065 -2.905385 -1.203155
C 2.204708 -1.182071 -0.470087
H 1.880444 -0.500806 -1.259045
C 1.472510 -0.742929 0.802922
O 0.635257 0.182175 0.756392
N 3.654650 -1.078115 -0.314402
N 1.796864 -1.365476 1.933642
H 2.458274 -2.130682 1.889270
C 1.202380 -1.056632 3.223190
H 1.129054 0.024929 3.352984
H 0.204846 -1.495188 3.313672
H 1.845170 -1.466421 4.002794
C 4.390992 0.043340 -0.353023
O 5.623491 -0.010824 -0.139030
C 3.710279 1.341483 -0.673496
H 3.274716 1.301947 -1.677182
H 2.899644 1.542301 0.033626
H 4.437209 2.152757 -0.628066
H 4.150603 -1.925750 -0.053356
H -0.984106 0.131603 1.646638
H 0.820310 1.761827 1.689279
H 6.694880 1.439045 -0.248879
H 6.486667 -1.547113 0.309584
H -1.098354 -2.819306 0.628445
O -1.713573 -2.814346 1.412319
H -2.551099 -3.160697 1.073117
O -1.844080 -0.120581 2.037241
H -1.912852 -1.078986 1.828431
O 0.922702 2.616322 2.153235
H 1.153850 2.368553 3.058895
O 7.324854 2.189908 -0.303247
H 8.193991 1.788516 -0.169058
O 6.952149 -2.376114 0.551781
H 7.322437 -2.701290 -0.279855
O -3.651332 1.742217 2.998707
H -3.028511 1.083947 2.620979
H -3.419889 1.785743 3.936306
63
complexB_6sol_conf_82
Eopt -2358.106011
C -3.661781 -0.253387 -0.679371
C -2.359086 -0.677786 -1.044363
C -1.276777 0.179246 -1.274123
C -1.488745 1.534276 -1.121555
C -2.765390 1.997142 -0.761276
C -3.825878 1.131517 -0.554162
H -0.307742 -0.229185 -1.545076
H -2.934695 3.061264 -0.640409
H -4.789624 1.543429 -0.282688
C -0.339804 2.455712 -1.329664
O 0.743439 2.089818 -1.758655
O -0.615874 3.709204 -1.012873
C 0.466775 4.650857 -1.122305
H 0.800374 4.716413 -2.159870
H 1.291350 4.342875 -0.475172
N -1.956883 -3.108772 -1.316635
N -2.128669 -2.021582 -1.197106
S -4.915315 -1.433435 -0.422182
C -6.344666 -0.432472 0.055125
H -6.141505 0.126104 0.970851
H -6.647746 0.233156 -0.755366
H -7.141780 -1.155422 0.244050
H 0.053006 5.601800 -0.792597
C 2.325108 -1.989447 1.125075
H 1.966837 -2.105210 2.153555
H 3.236674 -2.584530 1.029189
S 1.057354 -2.582138 -0.058184
C 2.718675 -0.507569 0.948179
H 3.390745 -0.239462 1.769560
C 3.529169 -0.334954 -0.331609
O 4.673797 -0.847402 -0.390087
N 1.527444 0.315382 1.019857
N 3.006076 0.332951 -1.346880
H 2.077993 0.736294 -1.253192

C 3.709946 0.515665 -2.604104
H 4.680440 0.990737 -2.436814
H 3.867001 -0.443854 -3.105657
H 3.103195 1.157152 -3.243478
C 1.431451 1.584342 1.438481
O 0.307675 2.133200 1.503058
C 2.678736 2.314829 1.838879
H 2.438500 3.362959 2.020813
H 3.083266 1.877334 2.758702
H 3.451339 2.244874 1.067549
H 0.666571 -0.141187 0.721523
H 5.400924 -1.541731 1.111921
H 4.790261 -2.297633 -1.517587
H 0.053485 3.851278 1.959448
H -1.151740 1.153285 1.884699
H -0.604315 -2.196537 1.268595
O -1.353566 -2.075876 1.910899
H -1.566508 -1.116966 1.908646
O 5.805978 -1.909495 1.927754
H 5.049257 -2.090777 2.502501
O 4.841090 -3.070082 -2.115514
H 4.250157 -2.840817 -2.846009
O -0.169188 4.775778 2.204243
H -0.135843 4.787932 3.170167
O -1.928911 0.646721 2.209053
H -1.849738 0.675636 3.172810
O -3.010117 -4.268509 1.602781
H -2.467312 -4.874921 1.080845
H -2.462200 -3.455645 1.685192
63
complexB_6sol_conf_83
Eopt -2358.107893
C 3.632805 -0.468598 -0.434901
C 2.471539 -0.493389 -1.247806
C 1.613913 0.592724 -1.452283
C 1.914187 1.775012 -0.808003
C 3.055131 1.842912 0.009270
C 3.896932 0.756989 0.187573
H 0.736046 0.466966 -2.082137
H 3.291693 2.768676 0.521955
H 4.762122 0.867574 0.828933
C 0.987742 2.927466 -0.984596
O 0.037787 2.913599 -1.749758
O 1.305416 3.962264 -0.218984
C 0.434422 5.104906 -0.287190
H -0.573412 4.821858 0.024470
H 0.422084 5.506939 -1.302211
N 1.885187 -2.606735 -2.402357
N 2.147033 -1.660790 -1.890147
S 4.599171 -1.907379 -0.267362
C 5.990325 -1.366263 0.755182
H 6.626490 -2.249211 0.852725
H 6.551562 -0.570535 0.261618
H 5.650067 -1.056944 1.744740
H 0.859280 5.828917 0.405953
C -2.346556 0.117656 -2.301417
H -1.851116 1.052645 -2.579629
H -3.245092 0.040309 -2.922789
S -1.225387 -1.301286 -2.649445
C -2.798572 0.288663 -0.845252
H -3.452743 1.164906 -0.809490
C -3.645258 -0.841882 -0.245346
O -4.124423 -0.703233 0.905761
N -1.663490 0.534950 0.027398
N -3.868656 -1.926140 -0.975947
H -3.262956 -2.066403 -1.787752
C -4.651650 -3.046446 -0.482790
H -4.165333 -3.523037 0.373960
H -5.646563 -2.709276 -0.182389
H -4.749548 -3.773878 -1.288946
C -1.431769 1.595495 0.816990
O -0.356226 1.682391 1.449130
C -2.475834 2.671212 0.919356
H -3.430760 2.260982 1.260370
H -2.133998 3.427510 1.626679
H -2.637768 3.140999 -0.056752
H -0.899163 -0.127170 -0.060479
H -3.009014 -1.950840 1.875928
H -5.259728 0.727896 1.087831
H -1.238685 0.061927 3.631059
H 0.727850 0.213391 1.536308
H -0.615698 -2.188315 -0.626210
O -0.278918 -2.531378 0.236644
H -1.054520 -2.528739 0.842164
O -2.295144 -2.521227 2.217515
H -1.801218 -1.938065 2.836570
O -5.936616 1.429973 1.175176
H -6.778653 0.965383 1.075772
O -0.795436 -0.782322 3.801806
H -0.044860 -0.780299 3.163476
O 1.100604 -0.662255 1.786045
H 0.689052 -1.307854 1.165934
O 3.406214 -0.647100 3.324127
H 2.630400 -0.657838 2.723987
H 3.032898 -0.803258 4.202220
63
complexB_6sol_conf_84
Eopt -2358.108817
C -2.680610 -1.354032 -0.354555
C -2.039120 -1.166715 0.895265
C -1.659013 0.073858 1.420975
C -1.914215 1.196743 0.660478
C -2.536632 1.053189 -0.591898
C -2.915073 -0.183598 -1.085860
H -1.184527 0.126000 2.394390
H -2.737834 1.931734 -1.193648
H -3.393768 -0.233182 -2.055404
C -1.515665 2.525752 1.201394
O -1.078533 2.691545 2.327952
O -1.687447 3.504731 0.323798

C -1.355611 4.835604 0.758044
H -2.007238 5.126958 1.584422
H -1.530858 5.469623 -0.109569
N -1.578604 -3.154357 2.299683
N -1.787789 -2.266361 1.673025
S -3.118582 -2.957734 -0.869875
C -3.886403 -2.682410 -2.484079
H -3.173355 -2.245754 -3.185944
H -4.171235 -3.677787 -2.833126
H -4.781917 -2.064927 -2.392027
H -0.308927 4.883520 1.063293
C 1.881079 0.301098 2.293776
H 1.012077 0.946466 2.458608
H 2.609569 0.561314 3.069716
S 1.398853 -1.461188 2.488560
C 2.479941 0.743553 0.952954
H 2.759369 1.792268 1.082812
C 3.794359 0.087892 0.499402
O 4.389753 0.546211 -0.500255
N 1.525721 0.681154 -0.143784
N 4.283917 -0.906768 1.231227
H 3.649713 -1.338447 1.907946
C 5.551000 -1.546901 0.927138
H 5.714078 -2.343129 1.653628
H 5.553829 -1.976101 -0.079122
H 6.369848 -0.825676 0.999001
C 1.134752 1.700788 -0.918218
O 0.419460 1.481854 -1.926066
C 1.562974 3.099582 -0.573834
H 1.028311 3.802634 -1.213195
H 1.357910 3.335991 0.474389
H 2.639002 3.212652 -0.745285
H 1.203221 -0.244412 -0.425744
H 3.566466 0.435498 -2.146281
H 3.881440 -2.509275 -1.484507
H -0.629292 2.810395 -2.562940
H -0.047070 -0.193562 -2.518216
H 1.010415 -2.139656 0.467444
O 0.819503 -2.410232 -0.469929
H 1.693637 -2.479097 -0.915192
O 3.168345 0.139177 -2.991994
H 2.249363 0.447732 -2.954571
O 3.097438 -2.489880 -2.050670
H 3.086679 -1.578464 -2.421880
O -1.239555 3.522589 -2.852124
H -1.632047 3.833480 -2.023868
O -0.212744 -1.126916 -2.762621
H 0.015387 -1.606090 -1.940728
O -5.137717 -0.211279 2.056057
H -4.710679 0.199720 1.290994
H -4.397214 -0.522165 2.595511
63
complexB_6sol_conf_85
Eopt -2358.106009
C -3.358940 -1.268013 -0.742286
C -2.006440 -1.505316 -1.094158
C -1.083578 -0.508540 -1.432277
C -1.515259 0.801683 -1.405370
C -2.841704 1.082178 -1.035587
C -3.741008 0.078506 -0.718317
H -0.067108 -0.775728 -1.699612
H -3.181055 2.111061 -0.997422
H -4.753146 0.351290 -0.449513
C -0.546605 1.872278 -1.765308
O 0.609078 1.647642 -2.091519
O -1.071511 3.084674 -1.714845
C -0.182244 4.181218 -1.992406
H 0.183875 4.112125 -3.018789
H 0.652772 4.169109 -1.288186
N -1.200280 -3.848496 -1.141813
N -1.554024 -2.799526 -1.122731
S -4.414048 -2.607792 -0.391079
C -5.966188 -1.804620 0.077435
H -5.825713 -1.165285 0.951327
H -6.387455 -1.243880 -0.759104
H -6.639606 -2.625318 0.335733
H -0.784395 5.078029 -1.860484
C 2.325851 -1.576385 1.793225
H 1.913595 -1.396942 2.791805
H 3.282983 -2.086670 1.925180
S 1.184265 -2.619648 0.810754
C 2.616092 -0.197567 1.171404
H 3.260012 0.352276 1.865568
C 3.422718 -0.353446 -0.114416
O 4.566001 -0.862152 -0.040582
N 1.368943 0.523652 1.008080
N 2.901695 0.054079 -1.261308
H 1.969165 0.458829 -1.271958
C 3.606947 -0.068025 -2.524820
H 4.556691 0.473098 -2.491812
H 3.802803 -1.118061 -2.760302
H 2.979697 0.361478 -3.306309
C 1.168199 1.845832 1.100437
O 0.005076 2.302477 1.029880
C 2.347632 2.751240 1.300101
H 3.145284 2.540375 0.582257
H 2.024780 3.787450 1.193663
H 2.755239 2.607561 2.307286
H 0.548866 -0.059902 0.846887
H 5.432325 -1.027512 1.534286
H 5.840864 -1.358800 -1.230776
H -0.435535 4.029233 1.223807
H -1.330051 1.310278 1.750149
H -0.670744 -2.104646 1.748959
O -1.543697 -1.887323 2.183398
H -1.408541 -2.093030 3.119577
O 5.966773 -1.108917 2.353718
H 6.556363 -0.343186 2.337688
O 6.597558 -1.662960 -1.774747
H 7.144827 -2.173976 -1.163334

O -0.752772 4.950735 1.343327
H -1.534529 4.866801 1.905783
O -2.000146 0.845560 2.293224
H -1.830248 -0.114808 2.164980
O -4.542747 1.858624 1.939287
H -4.641106 2.405550 2.730436
H -3.640451 1.471251 2.016763
63
complexB_6sol_conf_86
Eopt -2358.110554
C 3.049523 1.004986 0.857238
C 2.728740 0.862201 -0.514119
C 1.723179 1.573865 -1.178030
C 0.979610 2.472088 -0.439207
C 1.249729 2.626922 0.930620
C 2.253048 1.911374 1.565223
H 1.535123 1.387353 -2.231211
H 0.665868 3.328181 1.515559
H 2.425301 2.077754 2.621456
C -0.101510 3.226222 -1.133286
O -0.386965 3.060979 -2.307924
O -0.707226 4.098011 -0.339658
C -1.771525 4.872815 -0.919445
H -1.384271 5.483845 -1.737469
H -2.136942 5.503719 -0.110994
N 3.879011 -0.836686 -1.902047
N 3.404537 -0.066334 -1.264666
S 4.380092 0.105781 1.538881
C 4.068789 0.213203 3.319831
H 3.050976 -0.109088 3.549591
H 4.779733 -0.479530 3.776213
H 4.256877 1.219752 3.697283
H -2.562766 4.211690 -1.277770
C -0.779807 -0.722346 -2.220417
H -0.695062 0.278885 -1.780538
H -1.667348 -0.716637 -2.861660
S 0.704250 -1.107164 -3.220717
C -1.057711 -1.724918 -1.089746
H -1.084324 -2.731307 -1.514480
C 0.020583 -1.643112 -0.005585
O 0.059540 -0.662041 0.770151
N -2.366339 -1.424123 -0.511417
N 0.867362 -2.662697 0.073859
H 0.829425 -3.368106 -0.663111
C 1.973640 -2.730506 1.010194
H 1.810372 -2.026211 1.826284
H 2.035523 -3.741764 1.418716
H 2.918602 -2.495708 0.511688
C -3.040493 -2.176960 0.363419
O -4.128608 -1.774881 0.849700
C -2.479201 -3.502348 0.786049
H -3.288852 -4.121372 1.175394
H -1.972219 -4.027558 -0.026318
H -1.754024 -3.336649 1.591685
H -2.745207 -0.492292 -0.699558
H -0.429437 -1.013280 2.533590
H -1.095110 0.721981 0.985812
H -5.067888 -0.323883 0.390426
H -3.794200 -0.666735 2.284500
H 0.936467 -3.231916 -2.805358
O 1.010846 -4.174598 -2.492547
H 1.960929 -4.358301 -2.496479
O -0.809151 -1.146333 3.423678
H -1.700383 -0.757603 3.358164
O -1.725903 1.425041 1.251724
H -2.353847 0.960547 1.844858
O -5.577152 0.496161 0.201761
H -5.578330 0.976729 1.041821
O -3.424793 -0.047409 2.945936
H -4.163612 0.515258 3.218346
O -3.267175 1.460419 -1.058884
H -4.136987 1.233840 -0.667325
H -2.682744 1.585686 -0.277785
63
complexB_6sol_conf_87
Eopt -2358.108701
C -0.904906 -2.729480 0.398160
C -0.322275 -2.690702 -0.894103
C 1.030784 -2.437114 -1.153536
C 1.854844 -2.186222 -0.077962
C 1.313979 -2.196770 1.221013
C -0.022393 -2.468033 1.456040
H 1.395551 -2.421148 -2.174697
H 1.954151 -1.981784 2.069180
H -0.386355 -2.463846 2.476024
C 3.276885 -1.837864 -0.353962
O 3.763795 -1.851119 -1.472542
O 3.943365 -1.509051 0.742224
C 5.301842 -1.072303 0.564123
H 5.324509 -0.188154 -0.076532
H 5.899350 -1.875489 0.127980
N -1.771233 -3.074775 -2.866837
N -1.123096 -2.907772 -1.984217
S -2.593991 -3.093535 0.566187
C -2.885489 -2.960231 2.344310
H -2.695856 -1.944293 2.695975
H -3.946750 -3.187043 2.471015
H -2.293172 -3.693557 2.895223
H 5.657836 -0.830614 1.563879
C -2.022674 0.731309 1.355155
H -1.687603 1.515581 2.041258
H -1.650248 -0.217314 1.756495
S -3.848814 0.720522 1.273638
C -1.313296 0.935320 0.005334
H -1.635961 0.149800 -0.684048
C -1.634752 2.312550 -0.580850
O -1.105886 3.346244 -0.107286
N 0.127249 0.826756 0.215829
N -2.477175 2.354902 -1.603984
H -2.956278 1.491894 -1.864653

C -2.932229 3.608574 -2.178577
H -3.465960 4.209895 -1.435527
H -2.084892 4.186227 -2.557239
H -3.605263 3.380947 -3.005394
C 1.062925 0.805227 -0.733000
O 2.279657 0.884897 -0.423358
C 0.669361 0.720986 -2.178114
H 0.544777 1.740202 -2.564421
H 1.478244 0.243092 -2.734118
H -0.261952 0.174383 -2.340825
H 0.460168 0.903399 1.183033
H -0.514905 3.485962 1.613378
H 0.760726 3.719102 -0.304132
H 3.068499 1.303487 1.099495
H 3.471127 1.328361 -1.686776
H -4.062783 0.035727 -0.768988
O -4.064241 -0.163115 -1.748008
H -4.925133 0.151205 -2.058858
O -0.181741 3.672777 2.518070
H 0.602703 4.221156 2.375225
O 1.711795 3.932551 -0.367096
H 2.153183 3.069316 -0.321079
O 3.478747 1.558880 1.956160
H 3.541680 2.523551 1.915884
O 4.087350 1.668423 -2.371324
H 3.547364 2.288279 -2.880921
O 0.944306 1.169842 3.079912
H 0.535448 2.057127 2.976320
H 1.879681 1.304866 2.816924
63
complexB_6sol_conf_88
Eopt -2358.108835
C 3.112732 1.027338 0.543393
C 2.657683 0.629959 -0.737976
C 1.602703 1.230733 -1.434344
C 0.945037 2.279234 -0.823767
C 1.344282 2.684627 0.460735
C 2.395970 2.077198 1.128775
H 1.319814 0.848283 -2.411489
H 0.823484 3.498633 0.951673
H 2.663147 2.437016 2.114268
C -0.178370 2.927704 -1.556043
O -0.569014 2.556239 -2.650499
O -0.692005 3.956862 -0.896946
C -1.784806 4.649423 -1.525589
H -2.058870 5.440074 -0.829253
H -2.621775 3.965617 -1.678727
N 3.669016 -1.311048 -1.896550
N 3.257797 -0.432040 -1.363686
S 4.488847 0.234916 1.261783
C 4.584417 0.978215 2.908166
H 3.669425 0.788953 3.472811
H 5.419454 0.471249 3.397526
H 4.800274 2.046581 2.847248
H -1.458705 5.073395 -2.477613
C -0.983752 -1.124437 -2.051770
H -0.867104 -0.065220 -1.791814
H -1.904988 -1.209719 -2.637461
S 0.439463 -1.695948 -3.050199
C -1.214373 -1.913800 -0.752785
H -1.279998 -2.977081 -0.996099
C -0.075859 -1.675632 0.242025
O -0.000128 -0.586298 0.850913
N -2.483400 -1.482008 -0.169707
N 0.784414 -2.671600 0.415109
H 0.689044 -3.487565 -0.191287
C 1.956899 -2.601835 1.266440
H 2.857383 -2.793255 0.676254
H 2.024257 -1.612431 1.717123
H 1.890870 -3.349760 2.061712
C -3.110110 -2.044466 0.868799
O -4.150336 -1.525196 1.347598
C -2.551169 -3.294808 1.483150
H -2.147513 -3.988536 0.741701
H -1.742236 -3.020685 2.170757
H -3.337001 -3.788494 2.056645
H -2.847135 -0.578877 -0.488432
H 0.405046 -0.147998 2.555907
H -1.084446 0.861536 0.933684
H -3.735285 -0.156478 2.538022
H -5.145426 -0.194305 0.686415
H 0.651703 -3.734166 -2.309339
O 0.722968 -4.612010 -1.844279
H 1.664129 -4.833482 -1.882291
O 0.470602 0.205997 3.466656
H -0.270170 0.825837 3.524358
O -1.662333 1.629392 1.130156
H -2.274105 1.294925 1.821732
O -3.454957 0.630911 3.045812
H -4.206654 1.237247 2.982829
O -5.670270 0.564765 0.345815
H -5.714243 1.183289 1.088740
O -3.354950 1.296904 -1.057846
H -2.739735 1.547034 -0.332668
H -4.214736 1.154530 -0.608717
63
complexB_6sol_conf_9 Eopt
-2358.112501
C -2.770036 1.807458 -0.238245
C -1.907851 1.537431 -1.330470
C -1.641669 0.262119 -1.844114
C -2.254761 -0.813964 -1.236025
C -3.107918 -0.589869 -0.141118
C -3.364191 0.681100 0.343098
H -0.969276 0.147000 -2.686849
H -3.590873 -1.430096 0.343338
H -4.033468 0.791528 1.186740
C -1.980739 -2.178230 -1.767539
O -1.355116 -2.387696 -2.793483
O -2.488327 -3.135173 -1.002836

C -2.287421 -4.492071 -1.437485
H -2.784062 -4.650066 -2.397021
H -2.743388 -5.108972 -0.664775
N -0.771357 3.440871 -2.435109
N -1.282574 2.589940 -1.945474
S -3.026627 3.450067 0.278727
C -4.142476 3.274275 1.691523
H -5.096581 2.840032 1.386742
H -3.675806 2.689291 2.486153
H -4.309048 4.294216 2.046133
H -1.220549 -4.707634 -1.519480
C 1.922566 -0.645174 -1.710797
H 1.018923 -1.151241 -2.067087
H 2.738369 -1.009179 -2.345598
S 1.748232 1.169761 -1.900698
C 2.150110 -1.158910 -0.282732
H 2.332732 -2.236045 -0.354182
C 3.380559 -0.616821 0.455030
O 3.383510 -0.529689 1.702849
N 0.981970 -0.960024 0.554286
N 4.465207 -0.331161 -0.257138
H 4.398833 -0.241972 -1.265690
C 5.668474 0.197541 0.364305
H 6.426358 0.316045 -0.410319
H 5.475758 1.168525 0.831408
H 6.040993 -0.493321 1.124272
C 0.182367 -1.908472 1.053932
O -0.737244 -1.596309 1.849483
C 0.412175 -3.341120 0.663895
H 0.484743 -3.454955 -0.421777
H 1.347638 -3.699636 1.107955
H -0.410494 -3.951127 1.037746
H 0.786544 -0.006182 0.851126
H 3.204736 1.184554 2.343355
H 2.152293 -0.872828 3.010491
H -0.984954 0.159641 2.426959
H -2.136256 -2.736211 2.076227
H 1.057715 1.956943 0.022895
H 3.785843 1.305630 -2.638873
O 4.715552 1.120510 -2.934217
H 5.266207 1.689942 -2.378114
O 0.756716 2.314744 0.896615
H 1.551019 2.273989 1.473485
O 2.947922 2.083500 2.632611
H 2.488414 1.941470 3.472764
O 1.628955 -1.020600 3.825480
H 0.717805 -1.124985 3.511510
O -1.013195 1.098356 2.700481
H -0.462638 1.548180 2.025125
O -2.912626 -3.332041 2.147419
H -3.120223 -3.543748 1.226163
45
complexB_conf_1 Eopt -1899.566250
C -2.661196 0.832625 -0.300069
C -1.577405 0.244450 -0.994274
C -0.293171 0.788243 -1.076920
C -0.063816 1.992140 -0.439262
C -1.115277 2.617723 0.247249
C -2.380381 2.057797 0.317903
H 0.478070 0.269285 -1.632820
H -0.930648 3.561980 0.749116
H -3.152295 2.579850 0.869006
C 1.286198 2.618998 -0.427270
O 1.494209 3.762418 -0.055832
O 2.231654 1.786319 -0.834534
C 3.587880 2.257501 -0.786385
H 3.841540 2.557250 0.232693
H 3.716779 3.095448 -1.474828
N -1.945794 -1.867989 -2.239642
N -1.782511 -0.942208 -1.657634
S -4.199515 0.018411 -0.254014
C -5.258507 1.172731 0.650983
H -6.241741 0.696314 0.669388
H -4.907688 1.306972 1.676084
H -5.334075 2.130039 0.131601
H 4.196485 1.409944 -1.096918
C 0.260623 -3.102178 1.326583
H 0.487956 -3.460811 2.336079
H 0.288601 -3.973665 0.666108
S -1.414181 -2.341303 1.323507
C 1.421980 -2.178424 0.918539
H 2.349355 -2.586630 1.337059
C 1.647712 -2.085068 -0.603762
O 0.899484 -2.615607 -1.440419
N 1.221415 -0.860135 1.498494
N 2.751524 -1.413501 -0.964579
H 3.274862 -0.936928 -0.234280
C 3.070293 -1.137536 -2.352398
H 3.168386 -2.068897 -2.917610
H 2.298231 -0.522497 -2.827605
H 4.020302 -0.603728 -2.388203
C 2.186794 -0.014047 1.874039
O 3.399093 -0.253799 1.678944
C 1.746898 1.240040 2.578971
H 1.959691 1.126392 3.647873
H 2.329553 2.088637 2.211015
H 0.679517 1.441104 2.455377
H 0.236553 -0.621579 1.633668
45
complexB_conf_10 Eopt -1899.558673
C 3.112191 0.783946 -0.235469
C 2.061950 0.115962 -0.902102
C 0.761621 0.614075 -1.030563
C 0.500925 1.868033 -0.513408
C 1.536292 2.599740 0.089379
C 2.810007 2.068398 0.226094
H -0.000193 0.012574 -1.515719
H 1.337142 3.588893 0.486209

H	3.574885	2.638956	0.742056
C	-0.891702	2.386792	-0.620323
O	-1.757536	1.836435	-1.280921
O	-1.086006	3.494598	0.076440
C	-2.414275	4.049793	0.044459
H	-2.369447	4.931728	0.680858
H	-3.127647	3.324641	0.441158
N	2.586419	-1.952892	-2.167166
N	2.332506	-1.058730	-1.567496
S	4.732979	0.159578	0.016549
C	4.475298	-1.590475	0.462874
H	5.250239	-1.821844	1.197103
H	3.498888	-1.729377	0.933930
H	4.594502	-2.256554	-0.392819
H	-2.677367	4.325960	-0.978425
C	-1.593418	-2.882507	0.471947
H	-2.443609	-3.422803	0.042589
H	-1.291566	-3.442333	1.365223
S	-0.213154	-2.844770	-0.739864
C	-2.151195	-1.532321	0.950216
H	-2.932060	-1.763552	1.684441
C	-1.201633	-0.602369	1.715362
O	-1.647994	0.427704	2.257892
N	-2.798005	-0.773728	-0.106792
N	0.073952	-0.979195	1.826507
H	0.401530	-1.678077	1.151832
C	1.044811	-0.178656	2.548184
H	0.829709	-0.188750	3.621173
H	2.036027	-0.603332	2.384662
H	1.037156	0.859874	2.205677
C	-4.100711	-0.900681	-0.407933
O	-4.861218	-1.662248	0.222105
C	-4.620374	-0.056876	-1.541919
H	-5.276422	0.716050	-1.127440
H	-3.827360	0.424866	-2.117060
H	-5.219592	-0.686713	-2.205132
H	-2.232802	-0.144385	-0.667560

45

complexB_conf_12 Eopt -1899.564439

C	-2.231160	-1.158442	-0.327501
C	-1.332130	-0.644423	-1.292251
C	0.033989	-0.940960	-1.350638
C	0.542672	-1.798347	-0.394302
C	-0.320311	-2.347936	0.565471
C	-1.669210	-2.039509	0.603763
H	0.651105	-0.495660	-2.122897
H	0.080597	-3.035523	1.302555
H	-2.286281	-2.488763	1.371595
C	1.992068	-2.129096	-0.329672
O	2.503176	-2.705832	0.616211
O	2.661263	-1.734824	-1.403401
C	4.078053	-1.981824	-1.406335
H	4.433854	-1.599355	-2.361303
H	4.269649	-3.054063	-1.329520
N	-2.226902	0.745648	-3.140908
N	-1.826324	0.166178	-2.286799
S	-3.904987	-0.679212	-0.363664
C	-4.645338	-1.660172	0.964353
H	-5.706427	-1.399992	0.948375
H	-4.534299	-2.729312	0.772527
H	-4.225187	-1.387694	1.934556
H	4.550122	-1.449597	-0.577721
C	-0.415176	3.286294	0.728529
H	0.080270	4.241728	0.525768
H	-1.027993	3.427487	1.622397
S	-1.462563	2.836057	-0.711130
C	0.694806	2.281038	1.079159
H	1.445696	2.805689	1.685013
C	0.226600	1.112987	1.971675
O	-0.913316	1.057318	2.464155
N	1.347471	1.852677	-0.147054
N	1.173108	0.214073	2.270746
H	2.102371	0.350570	1.881232
C	0.962953	-0.844194	3.239593
H	1.766539	-1.574446	3.138986
H	0.967456	-0.447167	4.261483
H	0.004783	-1.339179	3.069738
C	2.640441	1.535058	-0.288636
O	3.396467	1.369099	0.692944
C	3.149308	1.442137	-1.701803
H	3.483979	2.440717	-2.006864
H	4.004272	0.766552	-1.747951
H	2.375315	1.114699	-2.400806
H	0.747397	1.931998	-0.970054

45

complexB_conf_13 Eopt -1899.569253

C	-1.740857	-1.433364	-0.387796
C	-1.043931	-1.707606	0.813491
C	0.337952	-1.899858	0.907891
C	1.082288	-1.785528	-0.249167
C	0.434145	-1.489931	-1.460082
C	-0.941157	-1.331316	-1.532681
H	0.797899	-2.105185	1.868517
H	1.017098	-1.383969	-2.368255
H	-1.391717	-1.110516	-2.492564
C	2.559477	-1.948041	-0.147076
O	3.111126	-2.477322	0.803062
O	3.200579	-1.450983	-1.195648
C	4.636180	-1.548329	-1.176035
H	5.032893	-1.026482	-0.302610
H	4.967904	-1.066723	-2.094170
N	-2.330291	-1.894959	2.924442
N	-1.758329	-1.800748	1.982355
S	-3.472208	-1.242978	-0.354582
C	-3.926737	-1.259176	-2.105812
H	-3.539885	-0.384484	-2.629944
H	-3.590204	-2.181838	-2.582927
H	-5.018873	-1.225177	-2.113168

H 4.937546 -2.597797 -1.162814
C -0.126472 2.413733 1.604726
H -0.356898 3.472413 1.432571
H 0.729174 2.377821 2.284925
S -1.551772 1.547605 2.380831
C 0.340797 1.784464 0.274706
H 0.317933 0.699010 0.400095
C -0.550126 2.128101 -0.922349
O -0.060853 2.503286 -2.007362
N 1.697842 2.166463 -0.072404
N -1.861092 1.997273 -0.733232
H -2.170988 1.709842 0.203489
C -2.821484 2.250202 -1.788286
H -2.571067 1.676709 -2.685966
H -3.809800 1.951240 -1.435538
H -2.848384 3.312425 -2.054602
C 2.786520 1.574835 0.448082
O 2.712450 0.727982 1.357711
C 4.107699 1.978147 -0.153325
H 4.098575 3.011674 -0.509581
H 4.901416 1.849504 0.585232
H 4.319826 1.325548 -1.007579
H 1.812145 2.764034 -0.883054
45
complexB_conf_15 Eopt -1899.569075
C 2.728538 0.745381 -0.062331
C 2.045201 0.244592 -1.197640
C 0.793631 0.690520 -1.634275
C 0.179384 1.692418 -0.909846
C 0.838177 2.239026 0.204139
C 2.081337 1.786256 0.613649
H 0.325739 0.237763 -2.500933
H 0.363030 3.030147 0.772846
H 2.539684 2.236263 1.484892
C -1.199132 2.097300 -1.302253
O -1.774101 1.652952 -2.282864
O -1.739524 2.963541 -0.457520
C -3.093104 3.371973 -0.726808
H -3.346654 4.061887 0.076029
H -3.755068 2.503330 -0.708143
N 3.084717 -1.576650 -2.516750
N 2.623177 -0.764041 -1.924423
S 4.255571 0.050464 0.407042
C 4.634518 0.893518 1.962645
H 4.802127 1.960246 1.802834
H 3.846566 0.722343 2.699055
H 5.561218 0.433297 2.313494
H -3.146930 3.872755 -1.695425
C -1.375488 -2.768505 -0.646144
H -2.126616 -2.754078 -1.442539
H -1.658686 -3.561082 0.056255
S 0.289417 -3.103345 -1.337488
C -1.472193 -1.417966 0.088428
H -1.339489 -0.616445 -0.645117
C -2.855574 -1.281999 0.718402
O -3.193078 -1.941056 1.717540
N -0.427945 -1.343636 1.089654
N -3.703467 -0.470567 0.073591
H -3.340624 0.116877 -0.666117
C -5.054901 -0.227562 0.543314
H -5.617630 -1.163297 0.598912
H -5.049175 0.239372 1.533698
H -5.549170 0.440233 -0.162564
C -0.314741 -0.357090 1.992826
O -1.182329 0.528321 2.117653
C 0.888425 -0.415018 2.896976
H 1.710186 -0.991071 2.462747
H 1.224120 0.599307 3.122422
H 0.591331 -0.892549 3.837749
H 0.378636 -1.932070 0.887571
45
complexB_conf_2 Eopt -1899.563593
C 1.582736 1.495119 -0.629493
C 0.934484 1.717449 0.607006
C -0.462149 1.821365 0.759215
C -1.259613 1.667937 -0.351786
C -0.654811 1.436647 -1.601558
C 0.716801 1.372132 -1.733180
H -0.886528 1.982879 1.744552
H -1.270423 1.304872 -2.484048
H 1.142680 1.194435 -2.715937
C -2.738036 1.702987 -0.169980
O -3.283289 2.118712 0.838599
O -3.387260 1.217232 -1.219551
C -4.824839 1.220397 -1.153393
H -5.163762 0.639495 -0.293946
H -5.153197 0.756712 -2.081896
N 2.075059 1.871043 2.799693
N 1.624190 1.801642 1.791800
S 3.272981 1.360484 -1.024520
C 4.234632 1.377972 0.506578
H 3.951281 0.547733 1.157062
H 5.261943 1.213809 0.169453
H 4.194502 2.344631 1.010812
H -5.189646 2.247502 -1.086882
C 0.654639 -2.544686 1.275677
H 0.819541 -3.562842 0.902801
H 0.032285 -2.626339 2.170785
S 2.249432 -1.737150 1.718187
C -0.157642 -1.748564 0.234518
H -0.039196 -0.691596 0.485828
C 0.325915 -1.919147 -1.207526
O -0.479483 -2.076278 -2.147206
N -1.577965 -2.047562 0.261571
N 1.644433 -1.869477 -1.386435
H 2.217587 -1.701302 -0.550334
C 2.251019 -1.876064 -2.702154
H 2.002538 -2.793490 -3.244522

H 1.915472 -1.018185 -3.295406
H 3.333485 -1.821476 -2.580323
C -2.407199 -1.515089 1.176581
O -1.986466 -0.852118 2.143271
C -3.876370 -1.791264 0.998321
H -4.136093 -2.043253 -0.032935
H -4.156819 -2.630370 1.644979
H -4.444211 -0.914337 1.317777
H -1.975479 -2.455276 -0.576754
45
complexB_conf_20 Eopt -1899.564793
C 1.588129 -1.605609 -0.321963
C 0.753934 -1.653509 0.820407
C -0.645124 -1.644883 0.790298
C -1.258784 -1.551474 -0.444058
C -0.467061 -1.467345 -1.600086
C 0.915428 -1.510041 -1.545775
H -1.206320 -1.699206 1.715860
H -0.950147 -1.380780 -2.567872
H 1.475470 -1.467568 -2.471048
C -2.741265 -1.543031 -0.580485
O -3.317832 -1.210311 -1.602974
O -3.359163 -1.951154 0.519884
C -4.796903 -1.973716 0.489489
H -5.139943 -2.696741 -0.253564
H -5.186480 -0.980025 0.260022
N 1.780817 -1.928376 3.061500
N 1.335329 -1.765738 2.061141
S 3.317672 -1.673789 -0.141357
C 3.915609 -1.571749 -1.845373
H 5.004498 -1.607847 -1.763072
H 3.572617 -2.422157 -2.437948
H 3.622691 -0.625462 -2.305313
H -5.098071 -2.281001 1.489445
C 1.415528 2.819162 1.386862
H 1.067474 3.610410 2.059279
H 2.319039 3.191207 0.896904
S 1.786944 1.314156 2.371898
C 0.334774 2.654974 0.305900
H -0.048514 3.652795 0.056082
C 0.856755 2.086076 -1.029360
O 2.062524 1.887530 -1.252373
N -0.771095 1.885616 0.850948
N -0.080671 1.902233 -1.969217
H -1.036840 2.164348 -1.745159
C 0.240520 1.501752 -3.325740
H -0.687275 1.250189 -3.840996
H 0.741539 2.311622 -3.868087
H 0.896695 0.628807 -3.329415
C -2.061523 2.023218 0.527956
O -2.438706 2.761465 -0.408872
C -3.052039 1.269540 1.371719
H -3.846939 0.877041 0.733900
H -2.591772 0.456941 1.939228
H -3.504413 1.975326 2.077685
H -0.490284 1.239657 1.590743
45
complexB_conf_21 Eopt -1899.568789
C -2.068472 -1.111967 -0.698399
C -1.270144 -1.727146 0.294893
C 0.095673 -2.000024 0.165421
C 0.708476 -1.660980 -1.025737
C -0.052889 -1.071658 -2.047599
C -1.402310 -0.802084 -1.892480
H 0.635514 -2.457043 0.986313
H 0.431791 -0.803670 -2.980561
H -1.937128 -0.328951 -2.706370
C 2.166924 -1.875796 -1.239633
O 2.734986 -1.617099 -2.287384
O 2.771372 -2.375290 -0.171249
C 4.197008 -2.541661 -0.250479
H 4.492393 -2.943551 0.717180
H 4.673353 -1.574463 -0.425427
N -2.357007 -2.592813 2.350156
N -1.869836 -2.148548 1.461574
S -3.746577 -0.776974 -0.378722
C -4.362344 -0.181812 -1.973140
H -3.898769 0.768847 -2.243837
H -4.217276 -0.928777 -2.756055
H -5.432957 -0.025355 -1.819592
H 4.449039 -3.242248 -1.049152
C -1.081365 1.996486 1.532523
H -1.918443 2.146207 0.842400
H -0.885272 2.958137 2.021636
S -1.508296 0.729529 2.783848
C 0.145266 1.633002 0.672609
H -0.112953 0.785647 0.032988
C 0.499321 2.818127 -0.221342
O 1.134171 3.800721 0.200702
N 1.254852 1.265526 1.525473
N 0.017912 2.759382 -1.469002
H -0.459446 1.918145 -1.765940
C 0.199270 3.836775 -2.424365
H -0.232396 4.767853 -2.045945
H 1.261260 4.000233 -2.631652
H -0.305165 3.562582 -3.350961
C 2.430456 0.830614 1.038914
O 2.708789 0.913504 -0.171682
C 3.387062 0.227538 2.031798
H 3.194806 0.567026 3.052694
H 3.274156 -0.861990 2.003049
H 4.412913 0.472281 1.747433
H 0.984937 1.002200 2.471380
45
complexB_conf_22 Eopt -1899.561303
C 2.865229 0.285397 -0.159567
C 1.670556 0.227899 -0.916097

C 0.827982 -0.883673 -0.990568
C 1.167464 -2.002601 -0.256079
C 2.340180 -1.985945 0.517478
C 3.170458 -0.877025 0.560433
H -0.067721 -0.839122 -1.602312
H 2.612998 -2.858667 1.100243
H 4.063629 -0.918284 1.171261
C 0.253797 -3.177016 -0.313160
O -0.793190 -3.181724 -0.940145
O 0.698747 -4.210266 0.389758
C -0.126690 -5.389002 0.398862
H -0.242133 -5.771269 -0.617286
H -1.101354 -5.158081 0.833487
N 0.987455 2.188536 -2.273358
N 1.290825 1.328278 -1.648103
S 3.830565 1.733754 -0.172538
C 5.280652 1.278630 0.809132
H 5.005311 1.067187 1.844255
H 5.810755 0.435522 0.361709
H 5.924108 2.161457 0.784612
H 0.408107 -6.106472 1.018844
C -1.576871 2.417918 0.241541
H -2.359398 3.068829 0.651551
H -1.433097 2.716711 -0.801983
S 0.002085 2.627644 1.155605
C -2.111750 0.966836 0.215071
H -1.266597 0.292945 0.046560
C -2.777071 0.545714 1.527768
O -3.934769 0.083963 1.553589
N -3.075376 0.765208 -0.851783
N -2.029661 0.692181 2.623019
H -1.155177 1.220296 2.508277
C -2.542632 0.408426 3.950187
H -2.830997 -0.642916 4.037271
H -1.752959 0.617188 4.672849
H -3.412896 1.030586 4.184267
C -2.728948 0.606312 -2.138173
O -1.541765 0.652047 -2.516427
C -3.845665 0.370188 -3.119104
H -4.832133 0.358311 -2.649854
H -3.818606 1.157732 -3.878534
H -3.674424 -0.586697 -3.621886
H -4.044870 0.648441 -0.581382
45
complexB_conf_23 Eopt -1899.555391
C -3.003837 0.980406 0.283542
C -2.057659 0.330126 -0.534306
C -0.832387 0.882477 -0.921364
C -0.492723 2.121244 -0.415146
C -1.380969 2.781400 0.449112
C -2.611009 2.231702 0.773220
H -0.166791 0.317504 -1.566109
H -1.117358 3.749991 0.858459
H -3.292371 2.786843 1.409231
C 0.843063 2.672644 -0.772536
O 1.622348 2.098811 -1.518976
O 1.104920 3.825925 -0.180203
C 2.402640 4.400192 -0.428013
H 2.420137 5.321719 0.150902
H 2.519293 4.611516 -1.492604
N -2.282243 -2.061764 -1.138167
N -2.235519 -0.986770 -0.885272
S -4.555776 0.355008 0.800599
C -5.190621 -0.589235 -0.620259
H -6.278508 -0.522927 -0.541005
H -4.883708 -0.126336 -1.561204
H -4.905641 -1.641338 -0.583578
H 3.181317 3.715248 -0.085254
C 1.170471 -1.339311 1.997788
H 1.357631 -0.357985 2.451070
H 1.737271 -2.072320 2.585516
S -0.619430 -1.726765 2.037930
C 1.775845 -1.296225 0.584330
H 1.207959 -0.595035 -0.025781
C 3.232951 -0.845933 0.663562
O 4.097789 -1.557809 1.210077
N 1.705831 -2.589854 -0.069124
N 3.514478 0.355015 0.149588
H 2.784596 0.870683 -0.330217
C 4.845894 0.931031 0.196421
H 5.173241 1.071465 1.230776
H 5.568170 0.288929 -0.316572
H 4.816638 1.900338 -0.302186
C 1.051292 -2.817902 -1.219814
O 0.583537 -1.901408 -1.923251
C 0.906057 -4.257202 -1.637317
H -0.158178 -4.515081 -1.617959
H 1.259295 -4.367427 -2.666514
H 1.449955 -4.950215 -0.991004
H 1.989724 -3.390483 0.481902
45
complexB_conf_25 Eopt -1899.559415
C -2.862097 -0.021622 0.382060
C -2.165100 -0.050799 -0.846199
C -1.285351 0.955640 -1.281678
C -1.064747 2.030760 -0.448386
C -1.709123 2.078698 0.799437
C -2.579999 1.083638 1.200520
H -0.788693 0.854167 -2.239673
H -1.524863 2.918648 1.461115
H -3.076912 1.167127 2.161655
C -0.141557 3.136343 -0.830980
O -0.163521 4.235674 -0.305047
O 0.703374 2.787361 -1.791908
C 1.638266 3.791265 -2.227524
H 2.228438 3.311518 -3.006236
H 2.275597 4.091457 -1.393176
N -2.139789 -2.047737 -2.312802

N -2.204298 -1.161926 -1.652765
S -3.984734 -1.193521 1.027849
C -4.742508 -2.029653 -0.393619
H -4.153757 -2.879619 -0.740353
H -5.696196 -2.404888 -0.013959
H -4.949746 -1.324097 -1.201435
H 1.100978 4.653180 -2.628264
C 0.735018 -1.286063 0.185383
H 0.786909 -0.977204 -0.866659
H 0.006225 -0.639834 0.680680
S 0.173371 -3.027007 0.324573
C 2.093717 -0.953543 0.841575
H 2.120545 -1.408630 1.838767
C 3.296932 -1.473135 0.050610
O 4.238026 -0.720904 -0.270778
N 2.251859 0.482984 0.984309
N 3.271482 -2.770986 -0.254935
H 2.385113 -3.262975 -0.083827
C 4.307317 -3.392130 -1.058827
H 5.281037 -3.306582 -0.568340
H 4.062757 -4.448799 -1.173546
H 4.373871 -2.932445 -2.050581
C 1.624236 1.207088 1.923385
O 0.857749 0.689628 2.759740
C 1.913792 2.684384 1.941961
H 2.567250 2.899452 2.794700
H 2.401486 3.033093 1.027986
H 0.978429 3.230096 2.088913
H 2.910765 0.939141 0.364462
45
complexB_conf_26 Eopt -1899.561253
C -1.176150 0.953753 -1.293521
C -1.647786 1.306902 -0.012759
C -2.855978 0.835295 0.517122
C -3.600120 -0.070567 -0.213177
C -3.137562 -0.481213 -1.473605
C -1.965892 0.030028 -1.999221
H -3.175714 1.163970 1.499901
H -3.710463 -1.191260 -2.058878
H -1.653247 -0.275633 -2.992866
C -4.865139 -0.582537 0.383391
O -5.286433 -0.230580 1.473565
O -5.480153 -1.463582 -0.395385
C -6.710525 -2.019190 0.101997
H -6.527622 -2.552111 1.037279
H -7.045367 -2.708044 -0.671602
N -0.216606 2.586805 1.579766
N -0.919657 2.105217 0.847318
S 0.271925 1.494376 -2.114940
C 0.688714 3.118497 -1.427048
H 1.249550 3.045069 -0.491168
H 1.331632 3.579126 -2.181467
H -0.204625 3.736255 -1.310189
H -7.444768 -1.225131 0.252128
C 2.942204 0.788032 0.432381
H 2.380385 0.986233 -0.488833
H 3.958271 1.157477 0.267711
S 2.220411 1.699557 1.841765
C 3.042671 -0.744323 0.613015
H 3.428751 -0.955058 1.616008
C 1.695811 -1.457623 0.458411
O 1.510360 -2.306999 -0.432707
N 3.951843 -1.321506 -0.358427
N 0.754013 -1.129316 1.346104
H 0.935296 -0.312223 1.930717
C -0.583752 -1.689605 1.286629
H -1.207310 -1.167760 2.012955
H -1.017042 -1.577042 0.288656
H -0.566238 -2.754799 1.536913
C 5.287596 -1.293546 -0.227451
O 5.848167 -0.743343 0.740451
C 6.087338 -1.959463 -1.314853
H 5.461408 -2.420388 -2.082567
H 6.735667 -1.211706 -1.782141
H 6.727368 -2.723832 -0.863918
H 3.535437 -1.835085 -1.126426
45
complexB_conf_27 Eopt -1899.561203
C -1.136191 1.073621 -1.300149
C -1.694284 1.191873 -0.011006
C -2.909236 0.597634 0.359665
C -3.568046 -0.201356 -0.555300
C -3.014462 -0.381824 -1.832047
C -1.840158 0.249175 -2.194742
H -3.289829 0.754439 1.362477
H -3.525876 -1.008794 -2.554568
H -1.458136 0.122059 -3.202891
C -4.845865 -0.885900 -0.214958
O -5.459887 -1.588675 -1.001156
O -5.241036 -0.654505 1.031226
C -6.465868 -1.281899 1.450329
H -7.294438 -0.935451 0.829333
H -6.606441 -0.970048 2.483860
N -0.413737 2.238504 1.856723
N -1.052503 1.860920 1.012282
S 0.326082 1.800523 -1.931464
C 0.622616 3.305211 -0.965565
H 1.284981 3.907295 -1.592762
H -0.305242 3.860615 -0.809742
H 1.131436 3.102141 -0.019148
H -6.369340 -2.367678 1.387463
C 2.854214 0.752846 0.631450
H 2.323029 1.071234 -0.274327
H 3.858618 1.181752 0.576222
S 2.030848 1.401822 2.127238
C 3.014128 -0.784233 0.578652
H 3.356681 -1.135525 1.557786
C 1.708142 -1.506528 0.235845

O 1.599864 -2.196638 -0.794562
N 3.994740 -1.170979 -0.417164
N 0.711865 -1.362925 1.113123
H 0.831514 -0.650077 1.833922
C -0.599151 -1.942364 0.883356
H -1.279851 -1.566797 1.647752
H -0.981803 -1.676212 -0.106188
H -0.555595 -3.033486 0.954718
C 5.319554 -1.115656 -0.208188
O 5.804934 -0.694490 0.859858
C 6.202469 -1.586902 -1.332428
H 6.853882 -0.761991 -1.637129
H 6.838058 -2.397877 -0.963871
H 5.638619 -1.937403 -2.200211
H 3.638121 -1.582061 -1.272262
45
complexB_conf_28 Eopt -1899.565201
C -2.778883 -0.549236 0.056036
C -2.047431 -0.538197 -1.158784
C -1.271648 0.535751 -1.610077
C -1.210056 1.665181 -0.819313
C -1.912635 1.691389 0.397141
C -2.673578 0.616148 0.825902
H -0.731051 0.461674 -2.547127
H -1.854183 2.569323 1.030870
H -3.187741 0.687933 1.775922
C -0.354710 2.798066 -1.269702
O 0.430140 2.717813 -2.200718
O -0.541508 3.894153 -0.549789
C 0.279043 5.030699 -0.870595
H 0.053345 5.378155 -1.880966
H 1.334973 4.766949 -0.785356
N -2.107170 -2.545250 -2.611628
N -2.075323 -1.653094 -1.957063
S -3.723424 -1.944797 0.487385
C -4.492851 -1.460181 2.051173
H -5.127481 -0.581459 1.920884
H -5.114503 -2.312945 2.334358
H -3.740337 -1.294633 2.824419
H 0.013812 5.789868 -0.136952
C 0.952149 -1.152606 0.072685
H 1.061474 -0.835766 -0.972283
H 0.129539 -0.578630 0.504423
S 0.534566 -2.935123 0.169880
C 2.214229 -0.693260 0.835803
H 2.195649 -1.130168 1.841368
C 3.523609 -1.111489 0.160995
O 4.424240 -0.283992 -0.082426
N 2.230238 0.754158 0.959150
N 3.633324 -2.407807 -0.132306
H 2.777351 -2.970304 -0.039049
C 4.784166 -2.944038 -0.834473
H 4.903304 -2.480850 -1.819693
H 5.699673 -2.780538 -0.258982
H 4.636129 -4.016952 -0.962965
C 1.457954 1.438265 1.816881
O 0.666243 0.872979 2.596936
C 1.617350 2.935742 1.817701
H 2.171682 3.224597 2.717610
H 2.154300 3.304402 0.940326
H 0.630495 3.402328 1.865194
H 2.894654 1.258662 0.384444
45
complexB_conf_29 Eopt -1899.565389
C -2.869374 -0.176968 0.080479
C -2.218526 -0.324908 -1.170656
C -1.305918 0.589123 -1.707426
C -1.006315 1.711911 -0.962049
C -1.626015 1.895077 0.285645
C -2.536140 0.981673 0.792797
H -0.837852 0.394106 -2.666289
H -1.383427 2.768213 0.881063
H -2.978833 1.171523 1.762518
C 0.027687 2.648204 -1.483099
O 0.608500 2.486408 -2.544182
O 0.257467 3.658158 -0.658269
C 1.297757 4.579668 -1.026231
H 1.029382 5.095116 -1.950817
H 2.242759 4.046343 -1.147173
N -2.673906 -2.355472 -2.516189
N -2.467581 -1.451670 -1.911563
S -3.988842 -1.396104 0.618355
C -4.553574 -0.772225 2.219564
H -3.725743 -0.705201 2.927948
H -5.059884 0.188709 2.110523
H -5.269712 -1.517377 2.574233
H 1.359219 5.284899 -0.199144
C 0.823622 -1.166779 0.029831
H 1.013940 -0.800016 -0.986691
H 0.044140 -0.538914 0.464329
S 0.222163 -2.898632 -0.004534
C 2.086392 -0.888964 0.875598
H 1.972577 -1.382742 1.847987
C 3.377376 -1.400605 0.230926
O 4.363236 -0.654636 0.067496
N 2.247130 0.538506 1.095994
N 3.374052 -2.685275 -0.127283
H 2.463558 -3.162496 -0.102719
C 4.496932 -3.295593 -0.814266
H 4.251717 -4.340559 -1.007625
H 4.702860 -2.795965 -1.766838
H 5.397998 -3.253432 -0.195923
C 1.516456 1.245110 1.971955
O 0.628543 0.721592 2.673639
C 1.838574 2.711359 2.093335
H 0.908693 3.285454 2.070420
H 2.315276 2.881137 3.064951
H 2.505796 3.067894 1.304952

H 3.000985 0.997800 0.598580
45
complexB_conf_3 Eopt -1899.560418
C -1.918299 0.927473 -0.240250
C -2.324426 -0.401367 0.027547
C -3.656519 -0.803039 0.166847
C -4.645692 0.154660 0.031940
C -4.279167 1.484429 -0.226099
C -2.954215 1.864776 -0.356763
H -3.884226 -1.841578 0.376895
H -5.053342 2.237532 -0.329741
H -2.727716 2.904131 -0.558942
C -6.088355 -0.188686 0.160765
O -6.984393 0.635382 0.076582
O -6.294311 -1.482645 0.375953
C -7.661944 -1.907226 0.515065
H -8.123753 -1.406356 1.368285
H -7.610642 -2.981365 0.684686
N -0.568535 -2.152513 0.304581
N -1.370416 -1.380783 0.194821
S -0.218495 1.292781 -0.399541
C -0.203225 3.100989 -0.456397
H -0.669733 3.525291 0.435266
H 0.856196 3.363773 -0.471827
H -0.680622 3.475175 -1.364259
H -8.215094 -1.689670 -0.400850
C 2.827185 -0.809480 -1.707900
H 3.309996 -1.298905 -2.560321
H 2.157957 -0.044276 -2.114280
S 1.879556 -2.060110 -0.762883
C 3.938025 -0.084810 -0.914928
H 4.598897 0.405346 -1.636866
C 3.367727 1.053528 -0.069651
O 2.961558 2.083762 -0.638939
N 4.711969 -1.046015 -0.161151
N 3.321739 0.905516 1.255933
H 3.670681 0.046428 1.659461
C 2.791381 1.934187 2.131834
H 3.336910 2.873198 2.000527
H 1.730632 2.113527 1.929624
H 2.903442 1.598930 3.163045
C 5.963655 -0.826932 0.277800
O 6.568069 0.236783 0.043321
C 6.605848 -1.930974 1.073539
H 5.963316 -2.807824 1.183002
H 7.537072 -2.225627 0.580110
H 6.859703 -1.545015 2.065770
H 4.220781 -1.900332 0.094489
45
complexB_conf_30 Eopt -1899.564769
C -1.075904 2.402978 0.209456
C -0.511501 1.335662 0.950322
C -1.126960 0.098398 1.159063
C -2.365016 -0.110775 0.584950
C -2.949246 0.910916 -0.181664
C -2.328603 2.136862 -0.358748
H -0.628689 -0.664219 1.745059
H -3.915520 0.748556 -0.646076
H -2.829164 2.893242 -0.949917
C -3.012977 -1.435751 0.785314
O -2.572148 -2.292782 1.533686
O -4.109041 -1.585977 0.053422
C -4.785187 -2.851609 0.155267
H -4.118066 -3.656499 -0.160674
H -5.636929 -2.774730 -0.518334
N 1.701142 1.668442 2.016567
N 0.714854 1.517613 1.540395
S -0.217777 3.910612 0.067296
C -1.374062 4.965867 -0.839872
H -2.314104 5.080807 -0.296499
H -0.875876 5.936247 -0.905012
H -1.546094 4.585115 -1.848483
H -5.120748 -3.014595 1.181395
C 3.105536 -0.177656 -1.786767
H 3.049012 -0.371742 -2.863948
H 4.168630 -0.184563 -1.526750
S 2.351942 1.445078 -1.388066
C 2.432154 -1.374252 -1.088752
H 2.897937 -2.294936 -1.458753
C 2.621949 -1.355889 0.435129
O 1.670040 -1.281064 1.227800
N 1.033341 -1.404846 -1.443424
N 3.888491 -1.451428 0.860917
H 4.631998 -1.523777 0.179448
C 4.234930 -1.446138 2.270885
H 3.766137 -2.287028 2.790828
H 5.317998 -1.534538 2.358298
H 3.914585 -0.515350 2.748620
C 0.272762 -2.509333 -1.408045
O 0.730868 -3.621534 -1.078305
C -1.154853 -2.361255 -1.862845
H -1.230991 -2.762795 -2.879688
H -1.804991 -2.959853 -1.220145
H -1.492317 -1.321924 -1.876319
H 0.599861 -0.494217 -1.576876
45
complexB_conf_31 Eopt -1899.564817
C -2.872256 -0.618906 0.275506
C -2.279068 -0.367247 -0.985985
C -1.607380 0.809415 -1.330577
C -1.491280 1.789963 -0.365022
C -2.078606 1.586292 0.894350
C -2.754534 0.417643 1.207763
H -1.191351 0.932301 -2.324632
H -2.003100 2.357721 1.652352
H -3.182612 0.312793 2.196909
C -0.709066 3.012074 -0.698546

O -0.212122 3.211304 -1.794997
O -0.603872 3.847601 0.324990
C 0.179151 5.035126 0.110901
H -0.264935 5.637470 -0.684151
H 1.205275 4.761797 -0.143423
N -2.459735 -2.089236 -2.760291
N -2.364517 -1.332264 -1.958674
S -3.665352 -2.140223 0.566286
C -4.296790 -1.966444 2.252028
H -3.479603 -1.858230 2.967704
H -4.999225 -1.133876 2.325627
H -4.826184 -2.901227 2.451745
H 0.149534 5.570514 1.058264
C 1.294410 -1.172921 -1.492869
H 1.710392 -1.603101 -2.410161
H 0.598915 -0.385345 -1.803896
S 0.425926 -2.479054 -0.538127
C 2.459743 -0.476220 -0.761621
H 3.022856 0.111646 -1.491477
C 1.992766 0.531630 0.298950
O 2.253861 1.744479 0.182013
N 3.337899 -1.487474 -0.201517
N 1.316898 0.032375 1.338249
H 0.982200 -0.928632 1.239925
C 0.825155 0.864686 2.419149
H 0.089083 0.296296 2.989033
H 1.643057 1.155962 3.086569
H 0.356885 1.774341 2.034713
C 4.579544 -1.241301 0.244057
O 5.103801 -0.113792 0.152079
C 5.323261 -2.398462 0.855235
H 6.249994 -2.557845 0.295477
H 5.593233 -2.137685 1.883306
H 4.743102 -3.324288 0.860350
H 2.895836 -2.384697 -0.011757
45
complexB_conf_32 Eopt -1899.558627
C 3.344349 -0.122807 0.051485
C 2.122522 0.390486 -0.452985
C 0.988464 -0.378516 -0.737965
C 1.076356 -1.743959 -0.537282
C 2.267779 -2.292746 -0.039397
C 3.376002 -1.507562 0.247068
H 0.087044 0.096657 -1.112690
H 2.336659 -3.361973 0.128196
H 4.270947 -1.986037 0.624789
C -0.124527 -2.564946 -0.839257
O -1.154692 -2.098119 -1.299497
O 0.039426 -3.849990 -0.555636
C -1.082747 -4.716538 -0.804534
H -1.355654 -4.680918 -1.861034
H -1.927572 -4.417859 -0.180780
N 1.866949 2.835084 -0.742025
N 1.990119 1.741276 -0.619074
S 4.664194 0.968151 0.378038
C 5.942664 -0.110320 1.066454
H 6.276227 -0.844669 0.330877
H 6.773040 0.557251 1.308738
H 5.594146 -0.596484 1.979669
H -0.738905 -5.712690 -0.531359
C -2.089882 1.672297 1.777903
H -2.680005 1.199332 2.570685
H -2.157796 2.753245 1.949221
S -0.347896 1.115566 1.855552
C -2.818987 1.361704 0.453667
H -3.819025 1.806820 0.513895
C -2.125447 1.976712 -0.760074
O -1.645996 1.286220 -1.676554
N -2.996863 -0.058505 0.227061
N -2.087284 3.314105 -0.788934
H -2.503724 3.831802 -0.026679
C -1.487642 4.051224 -1.885977
H -1.550317 5.115421 -1.658445
H -0.436793 3.777911 -2.015288
H -2.016657 3.855202 -2.823732
C -3.986502 -0.763296 0.797922
O -4.874823 -0.226337 1.490972
C -3.966277 -2.253454 0.578128
H -3.473535 -2.530683 -0.355893
H -3.422316 -2.721602 1.406587
H -4.990634 -2.632394 0.583109
H -2.264426 -0.555422 -0.271716
45
complexB_conf_33 Eopt -1899.566929
C 2.002392 -1.271727 0.229932
C 1.395188 -1.294836 -1.047585
C 0.056648 -1.617557 -1.289466
C -0.734211 -1.934268 -0.203534
C -0.175904 -1.905752 1.085505
C 1.156386 -1.590032 1.298741
H -0.330938 -1.615166 -2.302316
H -0.796624 -2.136165 1.943977
H 1.535948 -1.583776 2.313105
C -2.160815 -2.285027 -0.453168
O -2.670334 -2.267723 -1.561700
O -2.802186 -2.636177 0.651980
C -4.197378 -2.955776 0.515034
H -4.739493 -2.093867 0.120424
H -4.536097 -3.192043 1.522310
N 2.792818 -0.853742 -3.047137
N 2.168915 -1.026501 -2.150087
S 3.690153 -0.860815 0.373300
C 4.091534 -1.328262 2.075368
H 3.847432 -2.377286 2.254500
H 5.172372 -1.189509 2.157470
H 3.593616 -0.680314 2.798049
H -4.320619 -3.818476 -0.142994
C 0.020389 2.843290 -1.307404

H 0.180660 3.830864 -0.856372
H -0.670263 2.983646 -2.144212
S 1.601803 2.168816 -1.956344
C -0.713942 1.953046 -0.286401
H -0.745523 0.928312 -0.671487
C -0.064323 1.877663 1.109417
O -0.757969 1.779185 2.139937
N -2.085816 2.413515 -0.162756
N 1.268211 1.906161 1.147957
H 1.764098 1.924287 0.247237
C 2.003152 1.765159 2.388284
H 1.763339 0.818925 2.885187
H 3.070139 1.793435 2.162597
H 1.769188 2.583430 3.076942
C -3.132119 1.618762 0.118415
O -3.017640 0.387294 0.253238
C -4.472707 2.290961 0.251929
H -4.435620 3.363603 0.048020
H -5.176298 1.815816 -0.438082
H -4.842042 2.133141 1.270140
H -2.244183 3.412611 -0.209676
45
complexB_conf_34 Eopt -1899.564374
C -2.651661 -0.846368 0.415151
C -2.247363 -0.474017 -0.889468
C -1.745346 0.780884 -1.240817
C -1.615847 1.725185 -0.240215
C -2.009591 1.399282 1.066592
C -2.519160 0.150349 1.387426
H -1.465113 0.992267 -2.266915
H -1.915811 2.137289 1.855151
H -2.806366 -0.046238 2.412788
C -1.037633 3.049462 -0.597773
O -0.773715 3.379495 -1.742514
O -0.842441 3.825230 0.459597
C -0.235738 5.105995 0.216327
H 0.741837 4.971449 -0.251294
H -0.130149 5.563054 1.198710
N -2.558964 -2.086596 -2.748339
N -2.381459 -1.387602 -1.908993
S -3.243711 -2.458055 0.706891
C -3.708616 -2.419900 2.455075
H -2.836048 -2.254590 3.089995
H -4.479286 -1.669282 2.641079
H -4.116826 -3.412300 2.660633
H -0.883683 5.711904 -0.420419
C 1.355656 -0.964490 -1.750229
H 1.818668 -1.280513 -2.691067
H 0.640365 -0.172370 -2.004898
S 0.511691 -2.389898 -0.968128
C 2.471855 -0.309216 -0.902109
H 3.092310 0.299019 -1.566745
C 1.903968 0.681123 0.117869
O 1.725231 1.867097 -0.217723
N 3.292730 -1.342465 -0.309299
N 1.601207 0.210859 1.331030
H 1.757700 -0.774209 1.502541
C 1.089083 1.028309 2.414662
H 1.899211 1.350477 3.077584
H 0.597590 1.912581 2.008571
H 0.365896 0.449632 2.992426
C 4.474242 -1.105196 0.282147
O 4.982244 0.033283 0.303924
C 5.163554 -2.280833 0.919366
H 6.162846 -2.384050 0.485792
H 5.280805 -2.079836 1.988959
H 4.615634 -3.216920 0.788283
H 2.843174 -2.253741 -0.222472
45
complexB_conf_36 Eopt -1899.556791
C -0.913469 -2.557390 -0.014863
C -0.415916 -1.634490 -0.965856
C -1.062816 -0.455835 -1.346004
C -2.252362 -0.142080 -0.720321
C -2.770339 -1.018506 0.248625
C -2.128878 -2.200082 0.582941
H -0.615849 0.195829 -2.088392
H -3.700548 -0.775173 0.749887
H -2.578506 -2.844995 1.327660
C -2.911869 1.143625 -1.081142
O -2.494832 1.888855 -1.952654
O -3.991068 1.388774 -0.351184
C -4.670497 2.632030 -0.600436
H -3.996853 3.469118 -0.405866
H -5.506958 2.644202 0.096247
N 1.800226 -2.046695 -1.993482
N 0.809397 -1.868596 -1.535490
S -0.021750 -4.011922 0.324359
C -0.981921 -4.794140 1.642631
H -0.424348 -5.698308 1.898642
H -1.037410 -4.146959 2.520348
H -1.978062 -5.074252 1.294357
H -5.029528 2.661869 -1.631115
C 2.507809 0.879478 1.781399
H 2.133382 1.310826 2.716154
H 3.601814 0.910659 1.851434
S 1.921990 -0.845050 1.592322
C 2.098147 1.848218 0.651747
H 2.591259 2.810233 0.833494
C 2.575538 1.353823 -0.715278
O 1.782949 1.019943 -1.611738
N 0.665642 2.067181 0.561390
N 3.901560 1.316083 -0.887378
H 4.501529 1.585919 -0.119343
C 4.510630 0.806124 -2.102624
H 4.190478 1.388521 -2.971274
H 5.593115 0.886251 -2.002162
H 4.244865 -0.243067 -2.264226

C -0.120714 2.768208 1.393755
O -1.361813 2.752070 1.247934
C 0.501486 3.567039 2.506994
H -0.124668 4.440185 2.702212
H 0.518173 2.950317 3.412614
H 1.522673 3.890784 2.296428
H 0.182973 1.500512 -0.127545
45
complexB_conf_39 Eopt -1899.566795
C -2.626811 0.121164 0.712752
C -2.038124 0.960176 -0.263818
C -0.893412 1.738702 -0.059686
C -0.287265 1.678041 1.180615
C -0.843799 0.863641 2.179971
C -1.984282 0.110424 1.958871
H -0.512215 2.355144 -0.865023
H -0.366034 0.819072 3.153061
H -2.372318 -0.501091 2.763884
C 0.963656 2.433088 1.468624
O 1.661621 2.222940 2.446841
O 1.228313 3.350474 0.550540
C 2.467836 4.066741 0.682272
H 3.305599 3.366609 0.662911
H 2.470721 4.641905 1.610405
N -3.144461 1.189384 -2.471166
N -2.630327 1.052664 -1.500656
S -4.044325 -0.806288 0.313979
C -4.473130 -1.606229 1.879346
H -3.691129 -2.300372 2.193086
H -4.679213 -0.867100 2.655944
H -5.385831 -2.168028 1.666559
H 2.505582 4.731911 -0.178768
C 0.161037 -1.715212 -0.754064
H -0.240460 -1.044577 0.013140
H 0.039066 -2.732643 -0.371368
S -0.772893 -1.547794 -2.321889
C 1.685026 -1.471002 -0.837932
H 2.087490 -2.077975 -1.657392
C 2.086543 -0.014764 -1.105484
O 2.887494 0.582679 -0.360586
N 2.337684 -1.874437 0.394952
N 1.563226 0.540553 -2.199049
H 0.775536 0.035322 -2.626690
C 1.841903 1.913384 -2.577480
H 1.609570 2.610776 -1.767425
H 2.896354 2.033356 -2.845060
H 1.229274 2.158109 -3.446367
C 2.637609 -3.146688 0.696854
O 2.341585 -4.097365 -0.053962
C 3.352478 -3.381196 2.001163
H 2.738665 -4.037977 2.625358
H 4.295650 -3.897456 1.797544
H 3.558200 -2.457687 2.547914
H 2.680439 -1.130581 0.991592
45
complexB_conf_4 Eopt -1899.561261
C 2.829199 -0.132607 -0.223031
C 1.664471 -0.028140 -1.021568
C 0.648276 -0.985483 -1.079085
C 0.767007 -2.102363 -0.276363
C 1.899399 -2.239960 0.543642
C 2.907083 -1.288601 0.565017
H -0.210396 -0.819951 -1.722383
H 1.998285 -3.111960 1.180462
H 3.760172 -1.450568 1.211856
C -0.336801 -3.101324 -0.303765
O -1.339892 -2.973017 -0.986691
O -0.113615 -4.135717 0.496103
C -1.136029 -5.147025 0.546021
H -1.273849 -5.587309 -0.443508
H -2.071561 -4.713671 0.905315
N 1.340252 1.975236 -2.446548
N 1.486872 1.090208 -1.799882
S 4.031070 1.124686 -0.276190
C 5.314154 0.526621 0.850556
H 5.731110 -0.421853 0.506374
H 6.093021 1.292517 0.821943
H 4.932927 0.442431 1.870249
H -0.765225 -5.892023 1.247871
C -1.224908 2.625927 0.166124
H -1.926467 3.344362 0.608522
H -1.145562 2.871688 -0.897165
S 0.435053 2.757333 0.942333
C -1.852023 1.215047 0.248591
H -1.066912 0.479952 0.050108
C -2.435668 0.897270 1.627205
O -3.619161 0.530365 1.764773
N -2.911797 1.030416 -0.726379
N -1.592773 1.027084 2.652725
H -0.695315 1.484356 2.446302
C -2.019694 0.840490 4.026789
H -2.373890 -0.181814 4.186489
H -1.163889 1.022006 4.677954
H -2.823390 1.534850 4.293553
C -2.689394 0.831095 -2.034218
O -1.542003 0.833624 -2.522384
C -3.898855 0.606037 -2.901665
H -4.835315 0.606861 -2.339085
H -3.936917 1.391527 -3.662932
H -3.788017 -0.353886 -3.415291
H -3.854574 0.945634 -0.364232
45
complexB_conf_41 Eopt -1899.565980
C 2.524147 -0.260912 0.849610
C 2.101885 -0.992596 -0.287197
C 0.969986 -1.813980 -0.328837
C 0.197583 -1.904571 0.812568

C 0.579504 -1.192751 1.962352
C 1.716732 -0.403145 1.986663
H 0.718610 -2.348819 -1.238422
H -0.023350 -1.262248 2.860938
H 1.970965 0.119397 2.900313
C -1.017843 -2.764988 0.778937
O -1.206614 -3.629004 -0.060623
O -1.863953 -2.486603 1.761002
C -3.081848 -3.251233 1.798956
H -3.631923 -2.868879 2.657007
H -2.854089 -4.311023 1.930299
N 3.484277 -0.915939 -2.344225
N 2.851348 -0.920653 -1.436423
S 3.950970 0.731669 0.761407
C 4.092225 1.408062 2.433298
H 4.993067 2.026005 2.410871
H 3.232940 2.036589 2.675144
H 4.220912 0.613400 3.170848
H -3.647858 -3.093110 0.878595
C -0.035047 1.765126 -0.665439
H 0.317252 1.006928 0.041191
H 0.064918 2.730790 -0.161153
S 0.996027 1.769566 -2.180764
C -1.551923 1.544441 -0.867058
H -1.904353 2.241619 -1.636145
C -1.936090 0.130428 -1.319992
O -2.785545 -0.541208 -0.702445
N -2.275054 1.817050 0.362322
N -1.335142 -0.302859 -2.428446
H -0.521862 0.244664 -2.741661
C -1.572773 -1.627304 -2.970291
H -1.425095 -2.403524 -2.214055
H -2.593871 -1.710147 -3.355907
H -0.873403 -1.790291 -3.791543
C -2.596638 3.052040 0.776705
O -2.270915 4.074077 0.140590
C -3.375402 3.152226 2.061264
H -3.600473 2.177696 2.500805
H -2.797335 3.743835 2.777923
H -4.311478 3.683987 1.865031
H -2.634302 1.017019 0.869622
45
complexB_conf_42 Eopt -1899.563959
C 2.871087 0.092533 -0.221537
C 2.278133 -0.031030 1.059824
C 1.286497 0.817439 1.563576
C 0.848075 1.847230 0.755565
C 1.409847 2.005032 -0.522393
C 2.396245 1.156805 -0.998626
H 0.873688 0.646603 2.551968
H 1.065271 2.808617 -1.163503
H 2.792103 1.323533 -1.992515
C -0.241494 2.728673 1.261426
O -0.767405 2.589148 2.353439
O -0.569958 3.673887 0.393976
C -1.673179 4.526443 0.742594
H -2.569213 3.925670 0.911425
H -1.808475 5.182605 -0.115555
N 3.043766 -1.852442 2.556729
N 2.690727 -1.050291 1.881207
S 4.091379 -1.043210 -0.723525
C 4.542958 -0.469819 -2.378912
H 4.953936 0.541018 -2.346108
H 5.318592 -1.162386 -2.714403
H 3.691151 -0.528318 -3.059349
H -1.429277 5.107385 1.634609
C -0.451635 -1.374439 -0.220899
H -0.691947 -0.721585 0.622576
H 0.331384 -0.878576 -0.801575
S 0.195272 -2.976937 0.398207
C -1.676321 -1.428560 -1.155228
H -1.484774 -2.180897 -1.928448
C -3.012961 -1.835099 -0.511493
O -4.100697 -1.517007 -1.029501
N -1.833873 -0.164268 -1.863525
N -2.936611 -2.584127 0.589995
H -2.003568 -2.906303 0.872385
C -4.116103 -3.128025 1.235204
H -4.776760 -2.326486 1.578276
H -4.678400 -3.776561 0.555130
H -3.794784 -3.712906 2.098083
C -2.194303 0.990729 -1.275793
O -2.500161 1.053100 -0.071481
C -2.185435 2.216958 -2.151177
H -1.211164 2.708372 -2.053200
H -2.346199 1.978889 -3.206079
H -2.954260 2.912808 -1.808222
H -1.594503 -0.143474 -2.845483
45
complexB_conf_44 Eopt -1899.564019
C -2.624654 0.910539 0.164522
C -1.984592 0.335525 -0.960635
C -0.724105 0.711232 -1.436646
C -0.050801 1.710238 -0.762339
C -0.648933 2.305840 0.359563
C -1.899703 1.918682 0.812317
H -0.303429 0.227198 -2.310652
H -0.121853 3.085014 0.898254
H -2.313237 2.409917 1.683875
C 1.312694 2.081701 -1.230602
O 1.899946 1.491894 -2.122249
O 1.812976 3.121289 -0.577302
C 3.157572 3.505364 -0.911511
H 3.840045 2.676040 -0.713466
H 3.383299 4.349466 -0.262097
N -3.182533 -1.393243 -2.273200
N -2.637866 -0.646175 -1.664670
S -4.205801 0.359196 0.640154

C -4.603498 1.404152 2.062504
H -3.902752 1.234071 2.882166
H -4.635700 2.458816 1.782395
H -5.601330 1.085538 2.373560
H 3.213166 3.803734 -1.960391
C -0.009931 -1.670518 1.261926
H -0.142490 -0.635721 0.930249
H -0.118095 -1.670785 2.350643
S -1.313591 -2.737282 0.529739
C 1.444272 -2.111707 0.976983
H 1.496603 -3.192810 1.146423
C 1.944534 -1.902831 -0.466301
O 3.137393 -1.644792 -0.715067
N 2.383286 -1.531419 1.926477
N 1.039605 -2.095986 -1.426784
H 0.085349 -2.338351 -1.129642
C 1.365677 -2.005714 -2.835936
H 1.768512 -1.019799 -3.085984
H 2.101955 -2.764886 -3.120848
H 0.451111 -2.170493 -3.408352
C 2.707227 -0.228857 1.990355
O 2.203113 0.622966 1.237284
C 3.725688 0.162915 3.028541
H 4.065852 -0.679024 3.635885
H 4.586567 0.609061 2.520740
H 3.289124 0.925075 3.681044
H 2.854048 -2.165151 2.557374
45
complexB_conf_45 Eopt -1899.563959
C 2.871087 0.092534 -0.221537
C 2.278133 -0.031029 1.059824
C 1.286497 0.817439 1.563576
C 0.848074 1.847230 0.755565
C 1.409847 2.005033 -0.522393
C 2.396245 1.156806 -0.998626
H 0.873688 0.646602 2.551968
H 1.065271 2.808617 -1.163503
H 2.792103 1.323534 -1.992515
C -0.241495 2.728672 1.261426
O -0.767406 2.589148 2.353439
O -0.569959 3.673886 0.393976
C -1.673180 4.526442 0.742594
H -2.569214 3.925669 0.911425
H -1.808477 5.182604 -0.115555
N 3.043766 -1.852441 2.556729
N 2.690727 -1.050290 1.881207
S 4.091380 -1.043209 -0.723525
C 4.542959 -0.469817 -2.378912
H 4.953936 0.541020 -2.346107
H 5.318592 -1.162384 -2.714403
H 3.691151 -0.528317 -3.059349
H -1.429279 5.107384 1.634609
C -0.451634 -1.374439 -0.220899
H -0.691947 -0.721585 0.622575
H 0.331384 -0.878575 -0.801576
S 0.195273 -2.976937 0.398205
C -1.676321 -1.428560 -1.155229
H -1.484774 -2.180897 -1.928449
C -3.012960 -1.835100 -0.511493
O -4.100697 -1.517008 -1.029500
N -1.833873 -0.164268 -1.863525
N -2.936610 -2.584128 0.589995
H -2.003567 -2.906304 0.872384
C -4.116101 -3.128026 1.235205
H -4.776758 -2.326487 1.578278
H -4.678399 -3.776562 0.555131
H -3.794782 -3.712907 2.098083
C -2.194304 0.990728 -1.275793
O -2.500161 1.053099 -0.071481
C -2.185437 2.216958 -2.151177
H -1.211167 2.708373 -2.053201
H -2.346202 1.978888 -3.206079
H -2.954263 2.912807 -1.808222
H -1.594504 -0.143474 -2.845484
45
complexB_conf_46 Eopt -1899.564867
C -2.226357 -1.294734 0.339221
C -1.364455 -0.487838 1.121337
C 0.005638 -0.708698 1.280958
C 0.570390 -1.777582 0.613476
C -0.244535 -2.589771 -0.191794
C -1.604764 -2.360854 -0.322619
H 0.591199 -0.054362 1.916350
H 0.194035 -3.421362 -0.731578
H -2.186259 -3.022174 -0.952742
C 2.036796 -1.997055 0.746399
O 2.766733 -1.277200 1.407216
O 2.460902 -3.049792 0.060583
C 3.878045 -3.295516 0.065172
H 4.016515 -4.177056 -0.558349
H 4.221901 -3.488199 1.083402
N -2.305613 1.426199 2.384941
N -1.891444 0.583006 1.801537
S -3.924918 -0.929535 0.264734
C -4.594900 -2.269026 -0.749936
H -4.415560 -3.241915 -0.288106
H -5.670808 -2.082004 -0.786452
H -4.192206 -2.233739 -1.764065
H 4.403995 -2.438043 -0.360075
C -0.605065 3.158396 -1.337042
H -0.519601 3.501618 -2.373680
H -0.685730 4.052898 -0.712902
S -2.107132 2.110605 -1.171508
C 0.729646 2.475314 -0.992474
H 1.535200 3.031839 -1.486803
C 1.078050 2.493470 0.510133
O 0.326557 2.961690 1.381592
N 0.736332 1.126236 -1.535972

N 2.295570 2.018960 0.807510
H 2.849079 1.620308 0.053215
C 2.800118 1.969100 2.166242
H 2.157381 1.362028 2.811804
H 3.797194 1.528703 2.146488
H 2.865318 2.975519 2.591902
C 1.813785 0.463013 -1.969553
O 2.973484 0.914521 -1.836745
C 1.573369 -0.854163 -2.655585
H 2.205560 -1.621368 -2.199552
H 0.527977 -1.169480 -2.616611
H 1.873698 -0.752608 -3.704032
H -0.195964 0.713371 -1.610720
45
complexB_conf_47 Eopt -1899.565855
C -2.352026 -1.119123 0.379856
C -1.327709 -0.474845 1.115569
C 0.009921 -0.880667 1.145468
C 0.367354 -1.977359 0.385154
C -0.613952 -2.632415 -0.374627
C -1.936768 -2.222601 -0.376558
H 0.723212 -0.338701 1.754219
H -0.329280 -3.485643 -0.981464
H -2.652056 -2.770276 -0.977141
C 1.775527 -2.455658 0.319145
O 2.126214 -3.414336 -0.349363
O 2.600919 -1.722859 1.051502
C 3.996012 -2.065625 0.996905
H 4.482347 -1.407062 1.713879
H 4.377490 -1.886585 -0.010732
N -1.882619 1.482690 2.531873
N -1.645633 0.619233 1.883036
S -3.986681 -0.530759 0.471437
C -4.906964 -1.694272 -0.564536
H -4.835939 -2.712403 -0.176622
H -5.945840 -1.360451 -0.507912
H -4.572357 -1.643924 -1.602632
H 4.139087 -3.109920 1.281054
C -0.314001 3.235332 -1.163622
H -0.262581 3.632898 -2.182837
H -0.218478 4.086793 -0.484251
S -1.931312 2.391995 -0.925178
C 0.932080 2.351981 -0.974103
H 1.767342 2.817320 -1.511840
C 1.400322 2.236508 0.491158
O 0.801712 2.765027 1.442870
N 0.690966 1.053291 -1.582390
N 2.555114 1.577647 0.660942
H 2.969066 1.123592 -0.150128
C 3.155417 1.387309 1.967019
H 3.305864 2.350610 2.463479
H 2.533195 0.758435 2.612977
H 4.124643 0.907228 1.831767
C 1.612602 0.257190 -2.133859
O 2.839015 0.493578 -2.050129
C 1.105303 -0.936534 -2.896324
H 1.636554 -1.833790 -2.568277
H 0.028918 -1.084526 -2.783210
H 1.330678 -0.782409 -3.957280
H -0.300366 0.802401 -1.616337
45
complexB_conf_48 Eopt -1899.559306
C 2.130943 1.523418 -0.434388
C 2.106601 0.917079 0.845125
C 2.040371 -0.463107 1.072185
C 2.017596 -1.294643 -0.030895
C 2.065581 -0.733297 -1.317371
C 2.115405 0.635107 -1.518317
H 2.002982 -0.840131 2.087526
H 2.048995 -1.389941 -2.180934
H 2.135889 1.014748 -2.531955
C 1.913952 -2.774252 0.105653
O 2.023303 -3.540742 -0.836727
O 1.690890 -3.161184 1.354102
C 1.541214 -4.574481 1.574853
H 2.457983 -5.096787 1.293645
H 0.693766 -4.951743 0.998644
N 2.253483 2.362295 2.854169
N 2.150289 1.721710 1.957510
S 2.166848 3.258014 -0.570126
C 2.245049 3.535624 -2.356303
H 3.150767 3.100767 -2.783285
H 1.350637 3.152482 -2.851348
H 2.279754 4.621761 -2.471123
H 1.354173 -4.678094 2.642282
C -1.147102 0.859960 0.199081
H -0.953765 1.506728 -0.666008
H -0.323030 0.145213 0.259300
S -1.237303 1.835206 1.739782
C -2.383637 0.005460 -0.113769
H -2.585749 -0.654388 0.728985
C -3.593204 0.873271 -0.435912
O -3.574188 1.594540 -1.454145
N -2.132135 -0.835372 -1.280882
N -4.659575 0.826636 0.368930
H -5.426011 1.435273 0.105827
C -4.816442 0.030960 1.580363
H -4.778204 -1.039512 1.361208
H -4.049577 0.281200 2.317346
H -5.795214 0.262633 2.001257
C -1.479902 -2.011319 -1.203444
O -1.203574 -2.548498 -0.115157
C -1.096373 -2.655450 -2.508334
H -0.005734 -2.620386 -2.602786
H -1.397589 -3.706379 -2.491201
H -1.542051 -2.161417 -3.375072
H -2.226922 -0.399331 -2.190646
45

complexB_conf_49 Eopt -1899.565885
C 2.604756 -0.227165 0.908101
C 2.113599 -0.997008 -0.174780
C 0.926881 -1.738085 -0.151371
C 0.159883 -1.688345 0.995552
C 0.607807 -0.933278 2.092238
C 1.803278 -0.234660 2.057863
H 0.625664 -2.312478 -1.020163
H 0.010121 -0.894034 2.996143
H 2.107913 0.322192 2.935054
C -1.137701 -2.420201 1.011054
O -1.395094 -3.351332 0.267481
O -1.976486 -1.931612 1.914076
C -3.277625 -2.540607 1.989072
H -3.181956 -3.595363 2.254963
H -3.793576 -2.432414 1.032934
N 3.426088 -1.094083 -2.275678
N 2.833219 -1.030826 -1.343465
S 4.104242 0.641717 0.746023
C 4.287699 1.458104 2.349782
H 4.372400 0.727779 3.156914
H 5.222106 2.019184 2.274167
H 3.466096 2.154132 2.529469
H -3.802571 -1.994901 2.771131
C -0.071837 1.677213 -0.711772
H 0.285897 0.930626 0.002796
H -0.065563 2.636275 -0.185377
S 1.057292 1.780036 -2.152865
C -1.558706 1.367375 -0.998903
H -1.929754 2.093128 -1.732754
C -1.856861 -0.027884 -1.564856
O -2.790755 -0.719488 -1.114527
N -2.339333 1.515433 0.217989
N -1.096188 -0.423304 -2.585386
H -0.267503 0.154076 -2.788587
C -1.251218 -1.726690 -3.201816
H -2.215556 -1.804116 -3.713797
H -0.452879 -1.854853 -3.934282
H -1.188701 -2.531053 -2.461714
C -2.777400 2.695607 0.681696
O -2.543885 3.770093 0.093255
C -3.569492 2.668162 1.962098
H -4.536912 3.148842 1.788437
H -3.732584 1.657245 2.343420
H -3.034941 3.253055 2.717244
H -2.607362 0.667573 0.703209
45
complexB_conf_5 Eopt -1899.564735
C 2.472403 1.287349 0.293456
C 1.934273 0.850974 -0.936296
C 0.647651 1.157180 -1.387937
C -0.136266 1.971842 -0.592316
C 0.396568 2.505557 0.590119
C 1.674196 2.174560 1.018458
H 0.282569 0.751348 -2.324696
H -0.210173 3.155535 1.210035
H 2.036333 2.558509 1.965956
C -1.558443 2.172807 -0.993927
O -2.036454 1.700247 -2.012441
O -2.248438 2.875805 -0.109447
C -3.654668 3.038287 -0.372103
H -3.800990 3.564872 -1.317059
H -4.032612 3.630123 0.459553
N 3.340534 -0.422139 -2.533949
N 2.719635 0.120798 -1.798143
S 4.036067 0.821370 0.943405
C 4.099284 -0.978284 0.639162
H 4.705555 -1.220700 -0.235360
H 4.564304 -1.416148 1.525130
H 3.090481 -1.388458 0.535067
H -4.140471 2.060010 -0.398808
C -0.721745 -2.789111 -0.993184
H -1.417519 -2.801197 -1.838431
H -0.899808 -3.699938 -0.409613
S 1.013183 -2.743502 -1.585639
C -1.091909 -1.576240 -0.120021
H -1.046167 -0.679237 -0.744361
C -2.517721 -1.734033 0.401077
O -2.810352 -2.560097 1.283528
N -0.140797 -1.467617 0.967549
N -3.440508 -0.982150 -0.212656
H -3.129022 -0.256888 -0.846316
C -4.840955 -1.008905 0.166536
H -4.976033 -0.667118 1.198021
H -5.391374 -0.348015 -0.503373
H -5.244823 -2.020846 0.075808
C -0.247885 -0.599636 1.985281
O -1.251329 0.123664 2.136430
C 0.883734 -0.586666 2.979087
H 1.772825 -1.111517 2.619698
H 1.139946 0.446965 3.224976
H 0.538789 -1.074333 3.897661
H 0.759616 -1.902144 0.773333
45
complexB_conf_50 Eopt -1899.565322
C -0.232848 -2.139707 -0.812120
C 0.821588 -2.154391 0.129665
C 1.968162 -1.356323 0.055986
C 2.080156 -0.494359 -1.017286
C 1.059670 -0.456227 -1.981528
C -0.064459 -1.256833 -1.886906
H 2.721069 -1.420355 0.833249
H 1.151482 0.222914 -2.822522
H -0.825513 -1.185751 -2.654036
C 3.243941 0.423441 -1.160349
O 3.356939 1.222393 -2.075131
O 4.128877 0.288399 -0.182059

C 5.235486 1.206614 -0.179641
H 5.830861 1.079709 -1.085981
H 5.819709 0.946837 0.701444
N 0.727976 -3.759890 2.019031
N 0.741949 -3.015181 1.200956
S -1.625664 -3.166363 -0.584222
C -2.856061 -2.412068 -1.678863
H -2.608187 -2.565708 -2.730383
H -3.791277 -2.930414 -1.453784
H -2.968527 -1.349086 -1.450048
H 4.865335 2.231777 -0.105732
C -1.771922 0.076810 1.339707
H -1.313973 -0.233155 0.395275
H -2.795246 -0.310840 1.336605
S -0.856731 -0.653127 2.749878
C -1.893575 1.614782 1.327665
H -2.225729 1.940077 2.319454
C -0.600444 2.402816 1.047919
O -0.640448 3.569009 0.609920
N -2.936411 2.040526 0.402907
N 0.546575 1.793262 1.347962
H 0.489088 0.872452 1.797479
C 1.833736 2.441556 1.191020
H 2.606882 1.753855 1.535909
H 2.019791 2.694578 0.142421
H 1.888345 3.361201 1.783131
C -2.835275 1.963991 -0.934848
O -1.801498 1.561819 -1.499841
C -4.035432 2.401097 -1.732185
H -4.345856 1.575042 -2.379283
H -4.878671 2.704422 -1.107136
H -3.744258 3.239107 -2.373119
H -3.804938 2.374001 0.798033
45
complexB_conf_51 Eopt -1899.569267
C -1.796426 -1.411439 -0.186169
C -1.056162 -1.541166 1.013895
C 0.326147 -1.747319 1.080326
C 1.025392 -1.791250 -0.109330
C 0.333813 -1.640694 -1.322854
C -1.041481 -1.473431 -1.363355
H 0.821019 -1.837995 2.041159
H 0.882445 -1.658928 -2.257974
H -1.526290 -1.369169 -2.326227
C 2.503774 -1.965495 -0.050203
O 3.086158 -2.435733 0.912451
O 3.106743 -1.546412 -1.154119
C 4.534824 -1.712175 -1.215523
H 4.778427 -2.776270 -1.253707
H 5.008143 -1.246497 -0.350024
N -2.260848 -1.412609 3.176225
N -1.724734 -1.464040 2.210010
S -3.522819 -1.181612 -0.117649
C -4.049490 -1.513966 -1.818005
H -5.141470 -1.503717 -1.781969
H -3.709072 -0.738065 -2.504620
H -3.707183 -2.499644 -2.139891
H 4.840831 -1.215598 -2.134533
C -0.121618 2.664261 1.238420
H -0.310978 3.673269 0.851971
H 0.692582 2.745609 1.963779
S -1.613699 1.986734 2.076149
C 0.392743 1.774132 0.087519
H 0.295819 0.736893 0.416276
C -0.404932 1.910328 -1.210811
O 0.165314 2.019651 -2.315183
N 1.789622 2.009440 -0.228038
N -1.729950 1.889454 -1.083553
H -2.104385 1.793364 -0.131196
C -2.615575 1.913457 -2.229605
H -2.366676 1.109793 -2.930337
H -3.639306 1.778882 -1.877111
H -2.549286 2.868072 -2.762131
C 2.797447 1.512341 0.508478
O 2.598451 0.876698 1.561330
C 4.190891 1.807044 0.020199
H 4.847494 0.971865 0.271384
H 4.226667 1.996002 -1.055670
H 4.560361 2.697660 0.541383
H 1.997126 2.415398 -1.132784
45
complexB_conf_52 Eopt -1899.567459
C -2.029370 1.344818 0.606951
C -1.638613 1.460553 -0.749586
C -0.338697 1.738283 -1.184668
C 0.638905 1.901887 -0.223929
C 0.294324 1.793789 1.134457
C -1.002581 1.525973 1.541534
H -0.118201 1.796079 -2.244678
H 1.060151 1.912900 1.892693
H -1.210789 1.450693 2.601313
C 2.040644 2.119212 -0.677999
O 2.362023 2.194002 -1.852684
O 2.895596 2.207322 0.329875
C 4.285488 2.348773 -0.010583
H 4.607504 1.500320 -0.617862
H 4.813596 2.361046 0.941100
N -3.329183 1.065620 -2.517331
N -2.577304 1.247762 -1.726757
S -3.690629 0.993632 1.003267
C -3.638387 0.693349 2.786775
H -2.950940 -0.121502 3.023173
H -3.378682 1.601725 3.333625
H -4.655609 0.395060 3.051570
H 4.444133 3.285348 -0.549139
C 0.776017 -1.390193 -1.919434
H 1.483246 -2.121801 -2.329671
H 1.192176 -0.397537 -2.128260

S -0.868754 -1.565235 -2.703464
C 0.761645 -1.547527 -0.393724
H 0.041824 -0.849387 0.033660
C 0.390386 -2.979318 0.003718
O 1.163253 -3.921431 -0.262209
N 2.081770 -1.215843 0.129296
N -0.769030 -3.188582 0.638920
H -0.975782 -4.157442 0.851613
C -1.765948 -2.189112 0.998384
H -1.359983 -1.467760 1.713548
H -2.125597 -1.667084 0.108022
H -2.603677 -2.707698 1.465963
C 2.326063 -1.017801 1.435038
O 1.437167 -1.168895 2.294682
C 3.724631 -0.619593 1.822033
H 3.681550 0.319267 2.381896
H 4.132721 -1.388204 2.486148
H 4.390741 -0.498618 0.964737
H 2.826631 -1.049004 -0.536282
45
complexB_conf_53 Eopt -1899.565693
C -3.062547 -0.013871 -0.177464
C -1.987829 -0.333159 -1.042590
C -0.909185 0.511784 -1.322299
C -0.884032 1.746574 -0.704332
C -1.930794 2.103317 0.161667
C -2.993200 1.251341 0.417062
H -0.118934 0.188597 -1.991378
H -1.912416 3.069123 0.653853
H -3.772217 1.576804 1.095095
C 0.280293 2.640040 -0.957677
O 1.231965 2.322851 -1.651184
O 0.169375 3.810006 -0.342752
C 1.271657 4.722398 -0.486350
H 1.002056 5.591659 0.111016
H 1.391978 5.000042 -1.535506
N -2.007201 -2.505291 -2.238406
N -1.996043 -1.548744 -1.682423
S -4.333484 -1.172607 0.087729
C -5.492300 -0.283123 1.155082
H -5.037567 -0.049474 2.119687
H -5.865942 0.618532 0.665777
H -6.321282 -0.977929 1.309929
H 2.187361 4.264410 -0.106134
C 0.947267 -2.430330 0.069501
H 1.437709 -3.379901 -0.167541
H 0.687366 -1.961250 -0.888993
S -0.557523 -2.716116 1.072205
C 2.006058 -1.539210 0.749608
H 2.420160 -2.070872 1.615176
C 3.124845 -1.267208 -0.255889
O 2.994980 -0.442368 -1.177098
N 1.393945 -0.303697 1.190464
N 4.218304 -2.027304 -0.126523
H 4.283456 -2.655969 0.662690
C 5.337103 -1.939837 -1.047210
H 5.021491 -2.179042 -2.066909
H 5.769824 -0.934803 -1.039887
H 6.096925 -2.655714 -0.733584
C 2.074065 0.697936 1.764888
O 3.316151 0.667057 1.880807
C 1.276887 1.867744 2.275504
H 1.455379 1.966131 3.351060
H 1.637007 2.781039 1.792218
H 0.203842 1.763267 2.099688
H 0.377373 -0.306584 1.217832
45
complexB_conf_54 Eopt -1899.563195
C -2.873028 -0.017146 -0.224014
C -2.249242 -0.119274 1.044312
C -1.273289 -1.065853 1.378113
C -0.897132 -1.971845 0.406590
C -1.495732 -1.908376 -0.861654
C -2.455723 -0.960037 -1.173130
H -0.837304 -1.063846 2.370181
H -1.192175 -2.617110 -1.625209
H -2.878103 -0.951216 -2.170022
C 0.145298 -3.002907 0.668047
O 0.379862 -3.924465 -0.095341
O 0.781025 -2.808642 1.815196
C 1.828833 -3.739153 2.139050
H 2.232318 -3.394394 3.089474
H 2.597803 -3.715632 1.364144
N -2.907426 1.462780 2.835860
N -2.604429 0.768043 2.029554
S -4.056992 1.227001 -0.509510
C -4.548457 0.946531 -2.227760
H -3.700328 1.075949 -2.902896
H -5.005135 -0.037543 -2.350205
H -5.294203 1.717253 -2.436277
H 1.417233 -4.745572 2.239362
C 0.466131 1.347138 -0.106249
H 0.721966 0.554760 0.602865
H -0.369574 0.983574 -0.710489
S -0.071298 2.848558 0.801437
C 1.634538 1.503421 -1.098613
H 1.425174 2.365098 -1.742430
C 3.023771 1.772324 -0.497789
O 4.062283 1.520765 -1.139095
N 1.693487 0.349100 -1.987205
N 3.047927 2.335062 0.711607
H 2.148042 2.631716 1.106965
C 4.286107 2.749634 1.342501
H 4.044868 3.200381 2.306058
H 4.944790 1.892267 1.508725
H 4.818252 3.484587 0.729288
C 2.055513 -0.886158 -1.598792
O 2.458478 -1.129754 -0.446434

C 1.968228 -1.972332 -2.637361
H 1.572073 -1.620150 -3.592482
H 2.968443 -2.388276 -2.794563
H 1.329393 -2.773708 -2.254568
H 1.354101 0.468985 -2.931580
45
complexB_conf_55 Eopt -1899.561072
C 2.993376 -0.694336 -0.356946
C 2.244726 -0.785033 0.843293
C 0.984968 -1.385804 0.960815
C 0.426739 -1.919562 -0.184796
C 1.131716 -1.843234 -1.396110
C 2.384969 -1.258254 -1.484517
H 0.480972 -1.392966 1.918453
H 0.683603 -2.255704 -2.294162
H 2.884364 -1.231953 -2.444793
C -0.936681 -2.510902 -0.168101
O -1.522358 -2.873917 -1.177240
O -1.459722 -2.566868 1.047421
C -2.807791 -3.058606 1.156536
H -3.052299 -2.983559 2.214569
H -2.852415 -4.097750 0.824446
N 3.067121 0.333944 2.892380
N 2.722925 -0.178956 1.973384
S 4.543958 0.098194 -0.346218
C 5.008910 0.127449 -2.093934
H 5.164153 -0.882821 -2.476922
H 4.264856 0.665089 -2.685038
H 5.955179 0.672746 -2.124591
H -3.482644 -2.439039 0.562048
C -1.966058 1.041951 1.593249
H -2.321952 0.021840 1.787843
H -2.738835 1.724236 1.967802
S -0.385502 1.346676 2.467241
C -1.905090 1.201225 0.060745
H -1.181973 0.492334 -0.343226
C -3.288007 0.908111 -0.518247
O -4.236802 1.691930 -0.330523
N -1.522904 2.541381 -0.351629
N -3.427567 -0.229992 -1.205171
H -2.635740 -0.854950 -1.281932
C -4.720209 -0.715977 -1.654173
H -5.413025 -0.815045 -0.812764
H -5.156648 -0.036387 -2.391640
H -4.574815 -1.693539 -2.114662
C -0.331494 2.929950 -0.841131
O -0.082067 4.143426 -0.997659
C 0.670511 1.871276 -1.216130
H 1.549544 2.347191 -1.653431
H 0.962270 1.320749 -0.317070
H 0.249427 1.159635 -1.933517
H -2.132264 3.291258 -0.039843
45
complexB_conf_56 Eopt -1899.554987
C 3.672517 -0.342677 -0.110498
C 2.458706 -0.617946 0.568794
C 1.437320 0.314016 0.785058
C 1.632693 1.603341 0.328139
C 2.822564 1.916398 -0.347852
C 3.816676 0.973032 -0.564444
H 0.544715 0.019651 1.319253
H 2.979605 2.923768 -0.716964
H 4.713593 1.272265 -1.092107
C 0.551465 2.598465 0.551433
O -0.516621 2.326612 1.079827
O 0.866289 3.809821 0.118186
C -0.128604 4.837223 0.282989
H 0.319933 5.736375 -0.135641
H -0.348682 4.975435 1.343362
N 1.991501 -2.914347 1.364573
N 2.214005 -1.888197 1.011088
S 4.851139 -1.609177 -0.316524
C 6.163158 -0.809499 -1.271179
H 5.789253 -0.466855 -2.237951
H 6.614442 0.009994 -0.708467
H 6.910492 -1.590437 -1.430416
H -1.034457 4.569090 -0.264260
C -2.094239 -1.284254 -1.540664
H -2.430816 -1.149150 -2.575397
H -2.557705 -2.204619 -1.173890
S -0.270242 -1.418185 -1.481752
C -2.658406 -0.081983 -0.754749
H -2.213109 0.822937 -1.172647
C -4.159894 0.054703 -1.019473
O -4.563174 0.828765 -1.911414
N -2.297077 -0.052497 0.662788
N -4.991580 -0.708360 -0.305681
H -4.578332 -1.369499 0.349356
C -6.426901 -0.700821 -0.519054
H -6.839254 0.298978 -0.354552
H -6.675940 -1.018674 -1.536518
H -6.882332 -1.392200 0.190387
C -2.392482 -1.065527 1.538429
O -3.070795 -2.091318 1.320008
C -1.646955 -0.906352 2.836924
H -0.793003 -1.593478 2.826705
H -1.278653 0.109822 2.997445
H -2.299864 -1.192965 3.665421
H -1.663482 0.701410 0.913929
45
complexB_conf_57 Eopt -1899.562887
C 2.682404 -1.514904 0.061934
C 1.688230 -0.812977 -0.661313
C 1.625064 0.576176 -0.785534
C 2.593689 1.324600 -0.140656
C 3.596882 0.668959 0.589080

C 3.645537 -0.714319 0.684529
H 0.826707 1.031558 -1.362575
H 4.359280 1.249544 1.096165
H 4.441875 -1.169524 1.260241
C 2.515503 2.807468 -0.243789
O 1.643975 3.392695 -0.866184
O 3.490184 3.420123 0.416430
C 3.491911 4.858441 0.381787
H 2.571996 5.243337 0.826499
H 3.593336 5.207066 -0.647885
N 0.034301 -2.105855 -1.982681
N 0.737320 -1.534517 -1.347090
S 2.626815 -3.254290 0.134827
C 4.153282 -3.681072 1.007433
H 5.029105 -3.310593 0.470991
H 4.136230 -3.313229 2.035043
H 4.175470 -4.773480 1.020148
H 4.356084 5.156303 0.973137
C -1.224068 0.322760 1.209026
H -1.305317 0.799392 2.192277
H -0.387728 0.811153 0.696043
S -0.921417 -1.471784 1.389385
C -2.504353 0.674384 0.433842
H -2.632647 1.762340 0.450318
C -2.381825 0.303494 -1.046042
O -1.461959 0.794545 -1.728177
N -3.648333 0.076491 1.090320
N -3.293777 -0.518987 -1.568706
H -4.015323 -0.884828 -0.962729
C -3.311687 -0.870592 -2.976951
H -4.176677 -1.508042 -3.160127
H -3.391303 0.026483 -3.597956
H -2.405381 -1.413040 -3.260429
C -4.876874 0.623098 1.135876
O -5.145934 1.697242 0.565801
C -5.925895 -0.135320 1.903620
H -6.725349 -0.422443 1.213280
H -5.534522 -1.031306 2.391051
H -6.357642 0.527574 2.659402
H -3.468055 -0.809027 1.556809
45
complexB_conf_58 Eopt -1899.564244
C 2.581753 -0.828306 0.648804
C 2.194006 -1.057759 -0.693203
C 1.049388 -1.758081 -1.083959
C 0.238903 -2.269574 -0.091658
C 0.598759 -2.084519 1.255032
C 1.738748 -1.387108 1.618444
H 0.809636 -1.866146 -2.135689
H -0.034800 -2.485568 2.038502
H 1.966223 -1.269415 2.670641
C -1.031382 -2.932590 -0.499343
O -1.307621 -3.202457 -1.656379
O -1.836499 -3.163604 0.528347
C -3.122265 -3.734212 0.226488
H -2.995692 -4.715490 -0.235382
H -3.676575 -3.067998 -0.437665
N 3.616511 -0.140271 -2.503639
N 2.972409 -0.543703 -1.699932
S 4.010862 0.109340 0.986364
C 4.062352 0.162447 2.793753
H 4.945416 0.760205 3.032019
H 3.175354 0.655485 3.196287
H 4.183410 -0.838278 3.212590
H -3.627053 -3.825813 1.186430
C -1.031443 0.965178 -1.516112
H -1.920184 1.466767 -1.912157
H -1.173009 -0.107612 -1.685509
S 0.466489 1.527295 -2.422084
C -0.995695 1.144705 0.016470
H -0.051542 0.721911 0.381347
C -1.027973 2.585788 0.555677
O -1.389032 2.822622 1.724898
N -2.049308 0.366314 0.656277
N -0.603565 3.544554 -0.268485
H -0.186331 3.241071 -1.156878
C -0.470318 4.923180 0.161445
H -0.100399 5.510056 -0.680250
H -1.436849 5.327273 0.475943
H 0.233648 5.014398 0.995598
C -3.361497 0.638004 0.562894
O -3.791064 1.651151 -0.019814
C -4.300354 -0.351603 1.201871
H -4.906870 -0.813332 0.416064
H -3.782670 -1.136670 1.758148
H -4.974904 0.184492 1.875810
H -1.780919 -0.484754 1.130091
45
complexB_conf_59 Eopt -1899.567488
C -1.983999 1.359456 0.642163
C -1.595854 1.522203 -0.710077
C -0.292173 1.789880 -1.138655
C 0.691716 1.896033 -0.176147
C 0.348979 1.746499 1.178595
C -0.951506 1.486529 1.579600
H -0.073703 1.887313 -2.196279
H 1.119023 1.823972 1.937970
H -1.158307 1.374877 2.636479
C 2.095999 2.102273 -0.627645
O 2.419616 2.171474 -1.802090
O 2.949459 2.191601 0.381464
C 4.338696 2.346041 0.043035
H 4.866344 2.350509 0.995055
H 4.490003 3.290535 -0.483739
N -3.324133 1.292154 -2.471900
N -2.549031 1.388540 -1.688734
S -3.650100 1.021997 1.029123
C -3.604095 0.677033 2.804574

H -3.325941 1.566357 3.373240
H -4.627139 0.392908 3.062344
H -2.933420 -0.156951 3.021445
H 4.667987 1.508162 -0.574585
C 0.623394 -1.369472 -1.958657
H 1.301685 -2.094855 -2.425043
H 1.034423 -0.374263 -2.166765
S -1.064125 -1.517239 -2.652107
C 0.692727 -1.562323 -0.438662
H 0.003600 -0.869075 0.043046
C 0.332632 -3.000026 -0.052369
O 1.084800 -3.940337 -0.377503
N 2.042002 -1.252991 0.018599
N -0.792322 -3.216100 0.639736
H -0.993247 -4.187910 0.844388
C -1.762518 -2.218945 1.071763
H -1.315638 -1.513800 1.778816
H -2.165983 -1.676912 0.212647
H -2.577381 -2.742904 1.572587
C 2.361995 -1.096691 1.313597
O 1.517369 -1.249396 2.216436
C 3.790799 -0.748347 1.631940
H 4.398719 -0.573433 0.741211
H 3.805276 0.144971 2.262806
H 4.227673 -1.572367 2.205505
H 2.757308 -1.102074 -0.682081
45
complexB_conf_6 Eopt -1899.564581
C -1.885620 -1.432978 0.053275
C -1.252180 -1.240283 -1.197572
C 0.116474 -1.404983 -1.433514
C 0.914991 -1.747224 -0.361133
C 0.330131 -1.930982 0.902124
C -1.033803 -1.799136 1.101581
H 0.522907 -1.243035 -2.426228
H 0.953990 -2.196614 1.748566
H -1.434274 -1.971841 2.092543
C 2.378625 -1.900610 -0.583246
O 2.874817 -2.132540 -1.673014
O 3.076270 -1.744770 0.534464
C 4.504008 -1.892333 0.436123
H 4.875506 -1.745620 1.448752
H 4.912187 -1.133635 -0.234525
N -2.633560 -0.659749 -3.173494
N -2.019491 -0.883822 -2.280111
S -3.605499 -1.208455 0.201771
C -3.911859 -1.510547 1.959088
H -3.366673 -0.793337 2.576361
H -4.985252 -1.352865 2.090051
H -3.664274 -2.537749 2.234126
H 4.751811 -2.893303 0.076971
C -1.020812 3.198234 -0.313875
H -0.652509 4.126382 -0.763444
H -1.752443 3.480828 0.447303
S -1.810287 2.174154 -1.618840
C 0.176437 2.556399 0.408363
H 0.772375 3.363698 0.853818
C -0.208956 1.653546 1.598667
O -1.374673 1.543817 2.013678
N 1.015885 1.881327 -0.567406
N 0.824102 1.064602 2.216749
H 1.759622 1.263886 1.872712
C 0.673213 0.308237 3.445195
H 1.609012 -0.214969 3.646196
H 0.444945 0.968822 4.289441
H -0.131519 -0.424506 3.356052
C 2.346537 1.755410 -0.510807
O 3.004457 2.079883 0.502559
C 3.028270 1.238444 -1.748146
H 2.344596 0.711621 -2.418611
H 3.447946 2.096694 -2.285684
H 3.854020 0.581503 -1.466290
H 0.502362 1.568085 -1.393435
45
complexB_conf_60 Eopt -1899.561287
C 2.864080 0.116507 -0.179596
C 1.682141 0.127631 -0.958401
C 0.767811 -0.925864 -1.033800
C 1.014616 -2.051359 -0.272970
C 2.170778 -2.101006 0.523689
C 3.075736 -1.052061 0.563549
H -0.112829 -0.828783 -1.661276
H 2.371275 -2.979821 1.126267
H 3.953318 -1.145198 1.191000
C 0.017681 -3.156338 -0.320038
O -1.003336 -3.109466 -0.986788
O 0.356376 -4.187338 0.442734
C -0.556953 -5.299013 0.470423
H -1.526731 -4.974772 0.853209
H -0.102844 -6.023982 1.143688
N 1.142963 2.121405 -2.331337
N 1.383983 1.243886 -1.702963
S 3.926522 1.494642 -0.196241
C 5.309533 0.968515 0.845097
H 6.010643 1.806452 0.824443
H 4.986257 0.800091 1.874255
H 5.797532 0.082044 0.435017
H -0.662940 -5.719660 -0.531501
C -1.434855 2.504708 0.217045
H -2.185480 3.186093 0.636863
H -1.306310 2.783501 -0.833635
S 0.172865 2.674396 1.089895
C -2.014717 1.071329 0.225710
H -1.193523 0.369349 0.053332
C -2.665329 0.690374 1.557999
O -3.831942 0.254018 1.612203
N -3.006757 0.879576 -0.816895
N -1.896371 0.841319 2.637090

H -1.009070 1.341114 2.496665
C -2.389626 0.586403 3.977427
H -2.695715 -0.458019 4.085373
H -1.583171 0.791106 4.682484
H -3.243851 1.227976 4.217898
C -2.695434 0.703032 -2.109960
O -1.518709 0.735780 -2.520782
C -3.840537 0.467127 -3.057915
H -3.671467 -0.477244 -3.584213
H -4.810552 0.431353 -2.556492
H -3.851255 1.269258 -3.802565
H -3.968847 0.767363 -0.519020
45
complexB_conf_61 Eopt -1899.565840
C 0.494507 2.358925 -0.725698
C 1.090313 2.118640 0.536602
C 2.044089 1.125740 0.793502
C 2.448842 0.337282 -0.265662
C 1.901231 0.561009 -1.539791
C 0.948819 1.539217 -1.767556
H 2.429537 0.995557 1.798114
H 2.222707 -0.060742 -2.368922
H 0.547287 1.657139 -2.766241
C 3.429185 -0.769586 -0.084580
O 3.896402 -1.406885 -1.013511
O 3.732732 -0.983192 1.188266
C 4.640180 -2.064671 1.462881
H 5.602011 -1.878741 0.980565
H 4.211283 -3.005505 1.111371
N 0.430594 3.547510 2.451640
N 0.708450 2.895763 1.601543
S -0.725466 3.589291 -0.884784
C -1.162814 3.521181 -2.638445
H -0.304759 3.761841 -3.269124
H -1.584782 2.548293 -2.898337
H -1.928851 4.289836 -2.765601
H 4.751173 -2.076051 2.545723
C -1.290359 -0.225808 0.490059
H -1.668275 0.355819 -0.360957
H -0.201949 -0.231892 0.422852
S -1.776912 0.547334 2.077955
C -1.713223 -1.698388 0.293879
H -1.528553 -2.241627 1.228000
C -3.192003 -1.856261 -0.063054
O -3.556473 -2.505809 -1.062697
N -0.933215 -2.318445 -0.762337
N -4.051572 -1.260724 0.765402
H -3.648388 -0.629352 1.467644
C -5.481445 -1.258690 0.518456
H -5.721947 -0.770787 -0.432039
H -5.871216 -2.280192 0.495390
H -5.967464 -0.714857 1.329234
C 0.345624 -2.700106 -0.610545
O 0.948442 -2.586067 0.473864
C 1.030410 -3.241455 -1.837221
H 0.324495 -3.653911 -2.562968
H 1.578702 -2.422719 -2.316760
H 1.750482 -4.008493 -1.544093
H -1.402187 -2.494899 -1.642670
45
complexB_conf_62 Eopt -1899.564077
C 2.496917 0.721578 -0.524008
C 1.970676 0.876315 0.780280
C 0.749461 1.487425 1.075960
C 0.007658 1.990374 0.024865
C 0.501427 1.873421 -1.284691
C 1.712334 1.257262 -1.553902
H 0.407091 1.554935 2.102865
H -0.076185 2.265412 -2.113932
H 2.042935 1.185961 -2.582570
C -1.303633 2.625836 0.332290
O -1.698744 2.834123 1.467631
O -1.990288 2.937389 -0.758079
C -3.283526 3.534943 -0.559111
H -3.927103 2.850621 -0.002510
H -3.677551 3.700962 -1.560222
N 3.361798 0.210070 2.722378
N 2.717164 0.443375 1.852902
S 4.015557 -0.100079 -0.750774
C 4.332694 0.089673 -2.521848
H 3.579914 -0.433399 -3.114721
H 4.392681 1.143409 -2.801333
H 5.304993 -0.381476 -2.685162
H -3.179768 4.481911 -0.025424
C 0.269922 -2.071679 -0.023104
H 0.317634 -1.019424 -0.325283
H 0.700577 -2.653751 -0.843556
S 1.266071 -2.343708 1.491194
C -1.213938 -2.486460 0.058790
H -1.260152 -3.509880 0.446626
C -2.122840 -1.660276 0.987186
O -3.362733 -1.696679 0.861934
N -1.805844 -2.541286 -1.272229
N -1.522000 -0.957776 1.947296
H -0.512746 -1.095718 2.067394
C -2.264043 -0.186732 2.924749
H -2.854681 -0.836379 3.580694
H -1.551843 0.374893 3.531065
H -2.940539 0.515111 2.429975
C -2.126111 -1.457362 -1.999397
O -1.975992 -0.300542 -1.565484
C -2.697997 -1.702494 -3.370615
H -2.101765 -1.151738 -4.104153
H -2.720554 -2.759721 -3.645476
H -3.717215 -1.304153 -3.399268
H -1.941545 -3.452925 -1.686840
45

complexB_conf_63 Eopt -1899.565418

C	2.326715	1.034749	0.017860
C	1.797632	0.740440	1.297531
C	0.519560	1.107215	1.733508
C	-0.290619	1.792841	0.850926
C	0.195383	2.103511	-0.430931
C	1.466837	1.738232	-0.837006
H	0.180264	0.830210	2.725692
H	-0.439682	2.632972	-1.132231
H	1.789200	1.996980	-1.837618
C	-1.689063	2.093421	1.265039
O	-2.130821	1.848300	2.375346
O	-2.402798	2.627646	0.284142
C	-3.802078	2.837044	0.538595
H	-4.284760	1.878053	0.740044
H	-4.195628	3.280233	-0.374467
N	3.214066	-0.439084	2.956901
N	2.577772	0.054409	2.198032
S	3.940442	0.530253	-0.392398
C	4.115307	1.061172	-2.112662
H	3.372886	0.573689	-2.748023
H	4.059362	2.147782	-2.200661
H	5.113052	0.728248	-2.408654
H	-3.934898	3.517630	1.381929
C	0.918259	-2.068387	-0.891378
H	1.556633	-1.267885	-1.279442
H	0.868891	-2.832925	-1.677330
S	1.647126	-2.752347	0.643782
C	-0.472966	-1.476296	-0.688800
H	-0.441468	-0.806043	0.174477
C	-0.949954	-0.633514	-1.886607
O	-0.320997	-0.566941	-2.957394
N	-1.445152	-2.517352	-0.374395
N	-2.088554	0.040790	-1.682766
H	-2.500279	-0.006062	-0.754327
C	-2.622273	0.985070	-2.644797
H	-1.917499	1.804163	-2.825632
H	-2.836130	0.491450	-3.597342
H	-3.549944	1.395434	-2.244397
C	-2.475407	-2.334816	0.467024
O	-2.776067	-1.203895	0.903266
C	-3.266687	-3.551870	0.857813
H	-2.937884	-4.458419	0.344752
H	-3.167406	-3.697288	1.938358
H	-4.323460	-3.374189	0.638434
H	-1.197681	-3.465623	-0.629677

45

complexB_conf_64 Eopt -1899.567109

C	-1.460907	1.980501	0.602225
C	-0.885966	1.988038	-0.692025
C	0.479600	1.844516	-0.962779
C	1.331146	1.688766	0.112677
C	0.802631	1.693848	1.413629
C	-0.552247	1.836700	1.657800
H	0.826729	1.840715	-1.988579
H	1.475130	1.569837	2.256410
H	-0.902950	1.822704	2.681794
C	2.790777	1.462596	-0.073467
O	3.564825	1.295708	0.854137
O	3.151978	1.452441	-1.349621
C	4.541404	1.203364	-1.627349
H	5.158243	1.984459	-1.178441
H	4.623673	1.227266	-2.712525
N	-2.384524	2.212166	-2.650950
N	-1.713086	2.111827	-1.777252
S	-3.188161	2.124800	0.782122
C	-3.434737	1.997815	2.570393
H	-4.516624	2.060274	2.710853
H	-3.078622	1.036906	2.946468
H	-2.956615	2.828775	3.092402
H	4.826972	0.222083	-1.242660
C	0.631038	-1.478996	-1.667356
H	1.093455	-2.410230	-2.018147
H	1.416222	-0.715049	-1.670529
S	-0.719787	-0.961166	-2.796767
C	0.209541	-1.667383	-0.193162
H	-0.424598	-0.823494	0.100772
C	-0.596950	-2.958223	0.017337
O	-0.159805	-3.919149	0.673343
N	1.389684	-1.662044	0.649058
N	-1.808524	-2.962589	-0.555631
H	-1.946179	-2.254384	-1.284483
C	-2.627377	-4.161333	-0.586032
H	-3.551844	-3.931615	-1.117419
H	-2.117945	-4.986203	-1.096677
H	-2.876208	-4.479919	0.429471
C	1.340875	-1.495460	1.980542
O	0.259789	-1.381552	2.589206
C	2.655607	-1.446392	2.711276
H	2.714734	-0.505382	3.266469
H	2.683510	-2.266246	3.436096
H	3.518206	-1.527039	2.046102
H	2.296124	-1.716843	0.201589

45

complexB_conf_65 Eopt -1899.562086

C	2.412368	1.267420	-0.342074
C	2.118445	1.005118	1.019740
C	1.820947	-0.258596	1.543448
C	1.825636	-1.329578	0.673412
C	2.131539	-1.115772	-0.682039
C	2.417887	0.145065	-1.179760
H	1.585281	-0.370868	2.594645
H	2.135505	-1.955199	-1.368092
H	2.638221	0.251551	-2.234433
C	1.446872	-2.666622	1.207029
O	1.052639	-2.843422	2.348191
O	1.572196	-3.633818	0.309914

C 1.138186 -4.945546 0.710932
H 0.089967 -4.912508 1.016473
H 1.762514 -5.312189 1.528431
N 2.005039 2.910188 2.598465
N 2.055603 2.057644 1.894389
S 2.719123 2.902807 -0.858680
C 2.906643 2.747986 -2.651527
H 1.998325 2.346077 -3.104787
H 3.064404 3.769235 -3.006888
H 3.777707 2.140993 -2.905795
H 1.261098 -5.569012 -0.173047
C -1.275617 1.013049 0.277866
H -1.458945 1.991881 -0.184204
H -0.280621 0.694018 -0.044859
S -1.308121 1.143248 2.107980
C -2.249903 -0.010127 -0.350849
H -2.184871 -0.946128 0.213428
C -3.718351 0.434113 -0.345750
O -4.437952 0.341055 -1.359353
N -1.848996 -0.298507 -1.714442
N -4.174065 0.902958 0.817266
H -3.481402 1.041366 1.564008
C -5.530034 1.391556 0.975912
H -5.733951 2.227803 0.298956
H -6.256705 0.597848 0.777905
H -5.652284 1.732032 2.005000
C -1.104173 -1.357550 -2.073206
O -0.798136 -2.268197 -1.281575
C -0.626146 -1.388567 -3.500794
H 0.454924 -1.208686 -3.502265
H -0.801468 -2.384289 -3.917108
H -1.108489 -0.638180 -4.131581
H -2.044071 0.404753 -2.415796
45
complexB_conf_66 Eopt -1899.566484
C 2.145847 1.204116 -0.604536
C 1.557018 1.486840 0.651789
C 0.216164 1.838793 0.847192
C -0.591251 1.917737 -0.270606
C -0.043128 1.655345 -1.537250
C 1.285088 1.302689 -1.704559
H -0.152153 2.022368 1.849874
H -0.684358 1.713879 -2.410594
H 1.651782 1.097912 -2.702627
C -2.046409 2.217718 -0.168079
O -2.750096 2.447333 -1.137103
O -2.490998 2.186888 1.080173
C -3.902631 2.389210 1.268201
H -4.061378 2.309914 2.342199
H -4.460155 1.612737 0.739651
N 2.977346 1.328548 2.676406
N 2.340158 1.397461 1.774976
S 3.830318 0.764479 -0.692556
C 4.038162 0.266330 -2.419361
H 3.359975 -0.551662 -2.671495
H 3.905569 1.113722 -3.094403
H 5.069504 -0.087701 -2.487805
H -4.191255 3.379135 0.909225
C -0.993917 -1.156680 1.929995
H -1.761515 -1.821816 2.344692
H -1.382156 -0.135001 2.013676
S 0.576131 -1.312075 2.858578
C -0.865548 -1.455821 0.431042
H -0.078156 -0.832032 0.007117
C -0.538081 -2.933181 0.193292
O -1.377889 -3.811348 0.474108
N -2.121276 -1.115782 -0.227199
N 0.657015 -3.251815 -0.318212
H 0.827056 -4.244112 -0.432295
C 1.727635 -2.335508 -0.687801
H 1.423289 -1.684825 -1.513147
H 2.029167 -1.730355 0.170925
H 2.579544 -2.934042 -1.012645
C -2.262332 -1.073727 -1.562567
O -1.328999 -1.386236 -2.325762
C -3.604440 -0.652705 -2.096664
H -4.070909 -1.516482 -2.582139
H -4.273954 -0.277523 -1.319282
H -3.457435 0.121312 -2.854723
H -2.897532 -0.822807 0.353802
45
complexB_conf_67 Eopt -1899.563493
C -2.607094 -0.302484 -0.813876
C -2.113918 -0.945065 0.348278
C -0.949457 -1.719403 0.395530
C -0.206403 -1.836893 -0.762440
C -0.651198 -1.202457 -1.934510
C -1.826049 -0.469121 -1.965755
H -0.649275 -2.194858 1.322837
H -0.070463 -1.290670 -2.845898
H -2.132083 -0.011721 -2.898203
C 1.053106 -2.631680 -0.716746
O 1.295059 -3.457362 0.147118
O 1.872983 -2.336058 -1.715289
C 3.128636 -3.037456 -1.750306
H 2.954056 -4.110337 -1.854151
H 3.696104 -2.828200 -0.841238
N -3.393721 -0.753343 2.462952
N -2.813057 -0.817693 1.523125
S -4.084988 0.613966 -0.737170
C -4.269614 1.248863 -2.421096
H -5.188288 1.839909 -2.399198
H -3.432732 1.897200 -2.687869
H -4.384055 0.434700 -3.139318
H 3.648159 -2.647703 -2.623890
C 0.074820 1.758127 0.499139
H -0.330913 0.949309 -0.115722
H 0.030846 2.662382 -0.116288

S -0.950394 2.005126 1.998018
C 1.580052 1.471092 0.705424
H 1.999712 2.252821 1.346976
C 1.922980 0.134943 1.384869
O 2.822867 -0.604238 0.943728
N 2.266229 1.504641 -0.576483
N 1.236978 -0.148472 2.492843
H 0.423542 0.450609 2.693677
C 1.439015 -1.378470 3.233974
H 2.441035 -1.408751 3.673487
H 0.701433 -1.417714 4.037061
H 1.316648 -2.257997 2.593855
C 2.808823 2.579499 -1.173017
O 3.286688 2.490448 -2.323466
C 2.848587 3.877297 -0.409374
H 3.489352 3.778318 0.473732
H 3.253962 4.656078 -1.056574
H 1.851461 4.173846 -0.069364
H 2.258127 0.648321 -1.121826
45
complexB_conf_68 Eopt -1899.569039
C 2.742290 0.735864 -0.098785
C 2.031441 0.190804 -1.196666
C 0.771395 0.623253 -1.622762
C 0.178806 1.659699 -0.929697
C 0.866783 2.252644 0.142097
C 2.115204 1.809032 0.545118
H 0.282299 0.136809 -2.459091
H 0.408402 3.070500 0.686150
H 2.593064 2.292500 1.387328
C -1.209107 2.050630 -1.300982
O -1.837025 1.516706 -2.201703
O -1.696535 3.011945 -0.530762
C -3.057905 3.409356 -0.777008
H -3.157248 3.793525 -1.794029
H -3.260796 4.193245 -0.049552
N 3.030263 -1.680512 -2.476452
N 2.590272 -0.845200 -1.899416
S 4.274860 0.052194 0.368936
C 4.717492 0.999063 1.845719
H 3.966552 0.872751 2.628423
H 5.663987 0.569474 2.182084
H 4.867228 2.053538 1.606320
H -3.727179 2.560091 -0.621490
C -1.390501 -2.822771 -0.533110
H -2.132490 -2.838472 -1.338029
H -1.689664 -3.578490 0.202630
S 0.279121 -3.204077 -1.189007
C -1.478695 -1.438015 0.134596
H -1.318096 -0.676465 -0.634503
C -2.871002 -1.243773 0.728611
O -3.241238 -1.841703 1.754306
N -0.449320 -1.331639 1.148808
N -3.690119 -0.451712 0.024257
H -3.300783 0.091893 -0.735884
C -5.042470 -0.152192 0.456904
H -5.624858 -1.071270 0.563954
H -5.041168 0.377342 1.415405
H -5.513422 0.477875 -0.297911
C -0.325135 -0.294535 1.992618
O -1.183393 0.605541 2.064342
C 0.878986 -0.311396 2.897549
H 1.208008 0.712789 3.084681
H 0.587513 -0.756724 3.855712
H 1.704332 -0.896613 2.482499
H 0.352985 -1.936624 0.981387
45
complexB_conf_69 Eopt -1899.564866
C 2.226691 1.294356 0.339195
C 1.364776 0.487571 1.121415
C -0.005236 0.708674 1.281278
C -0.569927 1.777581 0.613795
C 0.244980 2.589611 -0.191676
C 1.605161 2.360523 -0.322638
H -0.590774 0.054520 1.916892
H -0.193551 3.421209 -0.731488
H 2.186689 3.021725 -0.952865
C -2.036314 1.997202 0.746882
O -2.766200 1.277558 1.407974
O -2.460481 3.049649 0.060667
C -3.877667 3.295174 0.065020
H -4.221865 3.487312 1.083234
H -4.403336 2.437810 -0.360816
N 2.305939 -1.426723 2.384587
N 1.891730 -0.583380 1.801439
S 3.925223 0.929067 0.264600
C 4.595057 2.268421 -0.750321
H 5.670943 2.081302 -0.787089
H 4.192110 2.233103 -1.764354
H 4.415905 3.241365 -0.288526
H -4.016107 4.176988 -0.558122
C 0.604405 -3.158172 -1.337423
H 0.518869 -3.501138 -2.374143
H 0.684964 -4.052830 -0.713496
S 2.106612 -2.110583 -1.171635
C -0.730249 -2.475036 -0.992699
H -1.535873 -3.031522 -1.486975
C -1.078449 -2.493282 0.509946
O -0.326797 -2.961831 1.381093
N -0.736873 -1.125970 -1.536233
N -2.295754 -2.018462 0.807660
H -2.849336 -1.619600 0.053531
C -2.800000 -1.968700 2.166506
H -2.157181 -1.361574 2.811943
H -3.797142 -1.528437 2.147008
H -2.864972 -2.975133 2.592168
C -1.814319 -0.462503 -1.969409
O -2.974083 -0.913777 -1.836199

C -1.573911 0.854636 -2.655511
H -1.875412 0.753361 -3.703650
H -2.205228 1.622126 -2.198751
H -0.528325 1.169428 -2.617592
H 0.195455 -0.713149 -1.610973
45
complexB_conf_7 Eopt -1899.565164
C 2.225827 -1.051160 0.224736
C 1.541124 -1.314609 -0.986339
C 0.217993 -1.765213 -1.074716
C -0.469232 -1.968551 0.105709
C 0.181432 -1.738961 1.329195
C 1.488322 -1.289011 1.392359
H -0.236484 -1.919504 -2.046684
H -0.358695 -1.909234 2.254481
H 1.935352 -1.119866 2.363666
C -1.908287 -2.349901 0.118338
O -2.558372 -2.462714 1.144626
O -2.404692 -2.534365 -1.096874
C -3.807152 -2.843060 -1.181671
H -4.393337 -2.032613 -0.743760
H -4.014321 -3.784807 -0.669358
N 2.743636 -1.030578 -3.135774
N 2.204111 -1.126408 -2.174712
S 3.871965 -0.485613 0.186425
C 4.223001 -0.122310 1.923774
H 3.520356 0.618157 2.312655
H 4.216388 -1.030026 2.530170
H 5.229937 0.301943 1.924631
H -4.015218 -2.933238 -2.246409
C 1.041289 2.291101 -0.259055
H 1.812583 1.765799 0.313285
H 1.073931 3.338717 0.066973
S 1.396762 2.150645 -2.051390
C -0.301569 1.690285 0.143560
H -0.382966 0.694121 -0.299025
C -0.444891 1.512192 1.665993
O 0.391152 1.947623 2.476683
N -1.411675 2.473342 -0.385764
N -1.531275 0.833474 2.056633
H -2.126521 0.441565 1.331992
C -1.758584 0.460262 3.439488
H -0.914976 -0.117772 3.831913
H -1.896108 1.345971 4.067108
H -2.660843 -0.150208 3.486148
C -2.566267 1.932968 -0.805614
O -2.831501 0.725877 -0.628182
C -3.541647 2.850397 -1.489379
H -3.719395 2.476686 -2.502694
H -4.492517 2.822509 -0.948997
H -3.187982 3.882436 -1.545201
H -1.223237 3.442098 -0.612435
45
complexB_conf_8 Eopt -1899.560252
C 1.686737 1.977219 0.046644
C 1.141671 1.021681 0.931397
C -0.233545 0.870284 1.173828
C -1.119498 1.681194 0.500809
C -0.618950 2.635066 -0.403195
C 0.736882 2.777636 -0.615160
H -0.575094 0.120810 1.878454
H -1.303359 3.273587 -0.949830
H 1.084301 3.531136 -1.315193
C -2.576371 1.474700 0.726587
O -3.028129 0.608375 1.456706
O -3.324438 2.327937 0.040297
C -4.749981 2.169269 0.147776
H -5.040824 1.179871 -0.211636
H -5.171828 2.946136 -0.487569
N 2.432911 -0.640721 2.237827
N 1.921667 0.124478 1.625178
S 3.339814 2.331097 -0.367326
C 4.423561 1.321850 0.669290
H 4.331403 0.260019 0.432707
H 5.429540 1.650662 0.394204
H 4.277308 1.529628 1.731057
H -5.065309 2.307741 1.183849
C 1.462813 -2.838836 -1.322387
H 1.428342 -3.223340 -2.347253
H 1.819452 -3.655192 -0.688463
S 2.621422 -1.411671 -1.256952
C 0.008557 -2.540732 -0.918911
H -0.630876 -3.321450 -1.349839
C -0.245415 -2.601191 0.601390
O 0.640201 -2.877211 1.427509
N -0.400284 -1.272562 -1.502802
N -1.518666 -2.407007 0.971393
H -2.196786 -2.169838 0.251764
C -1.935392 -2.446701 2.359891
H -1.417921 -1.686967 2.955237
H -3.008441 -2.259558 2.402702
H -1.728821 -3.427962 2.798837
C -1.638899 -0.950762 -1.892081
O -2.624245 -1.685704 -1.657279
C -1.801225 0.333999 -2.657534
H -0.883916 0.925833 -2.694854
H -2.102526 0.083440 -3.680511
H -2.603358 0.926306 -2.208339
H 0.376769 -0.626209 -1.655989
45
complexB_conf_9 Eopt -1899.560250
C -1.686126 1.972310 -0.047575
C -1.142009 1.020767 -0.937359
C 0.232984 0.871064 -1.182726
C 1.119765 1.677587 -0.505660
C 0.620175 2.626510 0.404201

C -0.735376 2.768910 0.617681
H 0.573268 0.125302 -1.891896
H 1.305366 3.261694 0.953720
H -1.081949 3.519178 1.321590
C 2.576382 1.471754 -0.733647
O 3.026787 0.618346 -1.479667
O 3.325882 2.309732 -0.030398
C 4.751155 2.149259 -0.138837
H 5.068565 2.307685 -1.171423
H 5.038728 1.152019 0.200991
N -2.435671 -0.646006 -2.235443
N -1.922521 0.124851 -1.631525
S -3.338691 2.325113 0.368854
C -4.423324 1.322681 -0.673355
H -4.336043 0.259937 -0.438943
H -5.428836 1.654819 -0.400575
H -4.273066 1.532317 -1.734218
H 5.174132 2.911961 0.512708
C -1.460719 -2.838961 1.320530
H -1.426548 -3.224231 2.345133
H -1.812462 -3.656371 0.685232
S -2.625335 -1.416903 1.253951
C -0.006968 -2.534667 0.920006
H 0.634684 -3.313228 1.351484
C 0.250227 -2.592905 -0.599876
O -0.632982 -2.870161 -1.428153
N 0.395901 -1.265457 1.505870
N 1.523971 -2.396322 -0.966905
H 2.199964 -2.158238 -0.245529
C 1.944762 -2.438676 -2.354124
H 1.746221 -3.423142 -2.789677
H 1.423544 -1.685043 -2.953863
H 3.016551 -2.243968 -2.394789
C 1.632620 -0.939678 1.897859
O 2.621081 -1.670768 1.663905
C 1.789382 0.344872 2.664795
H 2.583628 0.944500 2.211268
H 0.867650 0.929251 2.709085
H 2.099425 0.094864 3.685262
H -0.383868 -0.622296 1.658601
45
complexC_conf_1 Eopt -1899.561635
C -1.341154 -2.021112 0.176641
C -0.631759 -1.542937 1.307966
C 0.760623 -1.423975 1.387266
C 1.497748 -1.777178 0.276611
C 0.834427 -2.261887 -0.865013
C -0.543720 -2.394210 -0.912334
H 1.226451 -1.040280 2.287233
H 1.410769 -2.542542 -1.739638
H -1.003156 -2.775683 -1.815065
C 2.972253 -1.575660 0.329086
O 3.574506 -1.288842 1.350464
O 3.551844 -1.727929 -0.854421
C 4.979276 -1.550907 -0.902477
H 5.241269 -0.539259 -0.585758
H 5.250548 -1.707578 -1.945007
N -1.931384 -0.746389 3.262301
N -1.348388 -1.111394 2.394275
S -3.077436 -2.112969 0.224780
C -3.515437 -2.508817 -1.484034
H -3.103141 -1.745371 -2.150599
H -3.182092 -3.512196 -1.756199
H -4.607213 -2.471571 -1.513413
H 5.467722 -2.289209 -0.263264
C -0.253307 1.132885 -1.725959
H -0.085565 0.382624 -0.947145
H 0.567811 1.031286 -2.442660
S -1.845478 0.821912 -2.584260
C -0.089187 2.526026 -1.087289
H -0.348046 3.283222 -1.835374
C -0.974917 2.835710 0.130782
O -0.716950 3.797285 0.881019
N 1.311220 2.760834 -0.748062
N -2.041838 2.055079 0.310513
H -2.275682 1.400837 -0.444960
C -2.983746 2.271890 1.390660
H -2.477000 2.231626 2.359339
H -3.482953 3.242822 1.299356
H -3.735748 1.482322 1.352181
C 1.948059 2.160308 0.274831
O 1.340961 1.501080 1.136686
C 3.445700 2.308845 0.318183
H 3.767087 2.449245 1.352931
H 3.890467 1.378007 -0.052722
H 3.809368 3.136225 -0.296154
H 1.876412 3.228824 -1.444111
45
complexC_conf_10 Eopt -1899.555305
C 2.936959 -0.132099 -0.169609
C 2.370161 0.535311 0.944533
C 1.455996 1.597863 0.864385
C 1.060854 2.003140 -0.391237
C 1.580417 1.348450 -1.523738
C 2.498461 0.319634 -1.421561
H 1.071252 2.048687 1.771796
H 1.256300 1.665728 -2.509522
H 2.877168 -0.138508 -2.326180
C 0.035182 3.065897 -0.575425
O -0.581024 3.220624 -1.617024
O -0.128548 3.818713 0.503368
C -1.168584 4.810372 0.447084
H -1.152059 5.296757 1.420870
H -2.133184 4.327041 0.276247
N 2.950838 -0.253570 3.219276
N 2.699164 0.103749 2.200903
S 4.132691 -1.371960 0.078997
C 4.204837 -2.189794 -1.532697

H 3.196998 -2.499906 -1.823600
H 4.663868 -1.547237 -2.286585
H 4.833001 -3.070808 -1.380534
H -0.956591 5.531518 -0.344844
C -0.783880 -1.296494 -0.905161
H -0.233566 -0.395853 -1.203288
H -1.546401 -1.459571 -1.671210
S 0.355496 -2.723104 -0.822163
C -1.462774 -0.953613 0.432304
H -0.677928 -0.824143 1.179690
C -2.189093 0.397922 0.388153
O -1.828670 1.331895 1.129598
N -2.325587 -2.011345 0.965446
N -3.189616 0.529898 -0.487769
H -3.496499 -0.310385 -0.971936
C -3.963158 1.752858 -0.598430
H -3.326804 2.586014 -0.910298
H -4.738942 1.600985 -1.349414
H -4.435342 2.009719 0.355378
C -3.303103 -2.656348 0.305453
O -3.731436 -2.284574 -0.805346
C -3.883822 -3.869435 0.982301
H -3.521164 -4.007422 2.003508
H -4.973979 -3.784746 0.991569
H -3.619132 -4.752051 0.390364
H -2.014869 -2.431164 1.830755
45
complexC_conf_14 Eopt -1899.558320
C -2.556120 1.351140 -0.721825
C -2.310419 0.713240 0.516804
C -2.094317 -0.665711 0.673464
C -2.143693 -1.455936 -0.457482
C -2.394220 -0.864923 -1.702430
C -2.602845 0.500317 -1.829614
H -1.901040 -1.076185 1.657089
H -2.416311 -1.487595 -2.590912
H -2.776202 0.923549 -2.813433
C -1.889059 -2.922729 -0.383834
O -1.486360 -3.574536 -1.332947
O -2.189892 -3.435947 0.800283
C -1.899157 -4.831033 0.988305
H -2.482367 -5.433526 0.288754
H -2.190721 -5.048562 2.014436
N -2.340305 1.954199 2.661244
N -2.320594 1.433140 1.684515
S -2.885800 3.050384 -1.009316
C -1.834877 3.968985 0.157762
H -2.334892 4.161755 1.107678
H -0.880875 3.456924 0.298080
H -1.642094 4.926771 -0.331927
H -0.830407 -5.007606 0.847268
C 1.401255 -1.374595 -0.698470
H 1.822184 -2.138228 -1.362219
H 0.437861 -1.077936 -1.127726
S 1.189360 -2.044906 0.990925
C 2.328014 -0.146354 -0.781039
H 2.489983 0.095659 -1.836658
C 1.647939 1.081839 -0.177625
O 0.627088 1.540244 -0.724030
N 3.606259 -0.461791 -0.180937
N 2.176346 1.629269 0.919164
H 2.984577 1.186992 1.335463
C 1.572347 2.765251 1.589705
H 0.571946 2.515799 1.958452
H 2.202883 3.040023 2.435513
H 1.495358 3.619529 0.911181
C 4.767899 0.127533 -0.512477
O 4.833025 1.018160 -1.381733
C 5.997349 -0.337667 0.219916
H 6.725187 -0.704139 -0.510478
H 6.442556 0.518067 0.736839
H 5.788399 -1.127177 0.945528
H 3.573609 -1.154997 0.563904
45
complexC_conf_18 Eopt -1899.563130
C 2.327712 -1.527449 -0.218451
C 2.158698 -0.660825 0.894094
C 2.098757 0.738959 0.817516
C 2.195831 1.310397 -0.436982
C 2.332689 0.486871 -1.562288
C 2.413866 -0.895010 -1.460593
H 1.979914 1.328685 1.716480
H 2.390817 0.942362 -2.545671
H 2.538488 -1.479818 -2.363191
C 2.076805 2.782766 -0.615628
O 1.718673 3.301523 -1.660500
O 2.416640 3.461596 0.472306
C 2.254489 4.889602 0.419346
H 2.889090 5.310333 -0.363291
H 2.566448 5.249143 1.398416
N 1.888051 -1.655634 3.144792
N 2.019302 -1.210937 2.137777
S 2.424976 -3.250241 0.033932
C 2.377548 -3.912108 -1.649133
H 1.471170 -3.588663 -2.165227
H 3.272603 -3.634656 -2.208992
H 2.355015 -4.997364 -1.525453
H 1.206372 5.137054 0.236277
C -1.324384 1.486991 -0.617123
H -1.705463 2.348793 -1.176072
H -0.396647 1.174810 -1.110726
S -1.016855 1.951941 1.124928
C -2.340886 0.341688 -0.807199
H -2.585013 0.272183 -1.872128
C -1.713263 -1.004268 -0.446896
O -0.836582 -1.482333 -1.191433
N -3.550766 0.640493 -0.073278
N -2.127048 -1.625241 0.659723

H -2.812317 -1.164076 1.242723
C -1.553955 -2.880223 1.108466
H -2.116343 -3.224918 1.976579
H -1.615690 -3.635683 0.320420
H -0.504241 -2.754485 1.391984
C -4.753652 0.107349 -0.346766
O -4.926858 -0.683004 -1.294590
C -5.891711 0.509686 0.551550
H -5.588035 1.206729 1.335860
H -6.676523 0.970506 -0.056119
H -6.309553 -0.390833 1.012281
H -3.423045 1.233102 0.745235
45
complexC_conf_19 Eopt -1899.558319
C -2.556352 1.351036 -0.721829
C -2.310867 0.713201 0.516883
C -2.094525 -0.665731 0.673559
C -2.143444 -1.455955 -0.457391
C -2.393865 -0.864987 -1.702397
C -2.602747 0.500207 -1.829623
H -1.901320 -1.076173 1.657215
H -2.415627 -1.487659 -2.590887
H -2.775966 0.923404 -2.813478
C -1.888637 -2.922730 -0.383781
O -1.485591 -3.574393 -1.332831
O -2.189899 -3.436131 0.800160
C -1.899493 -4.831331 0.987905
H -2.482722 -5.433514 0.288103
H -2.191272 -5.049050 2.013934
N -2.341489 1.954322 2.661160
N -2.321380 1.433146 1.684495
S -2.886001 3.050299 -1.009371
C -1.834946 3.968884 0.157572
H -2.334840 4.161728 1.107536
H -0.880937 3.456826 0.297860
H -1.642147 4.926662 -0.332135
H -0.830760 -5.008111 0.846995
C 1.402033 -1.374891 -0.698687
H 1.823353 -2.138434 -1.362289
H 0.438643 -1.078553 -1.128163
S 1.189976 -2.045195 0.990702
C 2.328428 -0.146359 -0.781062
H 2.490524 0.095679 -1.836659
C 1.647838 1.081599 -0.177762
O 0.626852 1.539596 -0.724207
N 3.606642 -0.461394 -0.180692
N 2.175952 1.629201 0.919099
H 2.984386 1.187276 1.335370
C 1.571365 2.764789 1.589742
H 0.571123 2.514777 1.958550
H 2.201802 3.039880 2.435527
H 1.493906 3.619072 0.911277
C 4.768112 0.128365 -0.511981
O 4.833012 1.019200 -1.381050
C 5.997709 -0.336664 0.220262
H 6.725013 -0.704113 -0.510189
H 6.443562 0.519345 0.736153
H 5.788842 -1.125471 0.946663
H 3.574127 -1.154921 0.563855
45
complexC_conf_2 Eopt -1899.555222
C 2.923047 -0.252282 -0.160762
C 2.478940 0.551102 0.919410
C 1.608393 1.646127 0.800332
C 1.142320 1.953554 -0.458889
C 1.560234 1.181046 -1.557874
C 2.429666 0.114407 -1.419497
H 1.308766 2.197056 1.684481
H 1.188877 1.428679 -2.546981
H 2.719633 -0.444350 -2.299680
C 0.139313 3.032580 -0.672342
O -0.520926 3.133017 -1.693322
O 0.044969 3.862289 0.357287
C -0.968427 4.879428 0.275286
H -0.771883 5.536587 -0.574227
H -0.894709 5.431817 1.210459
N 3.217031 -0.024322 3.212453
N 2.890918 0.233301 2.185115
S 4.027466 -1.565420 0.130991
C 4.004103 -2.468618 -1.436039
H 2.970561 -2.723044 -1.690534
H 4.488505 -1.898497 -2.231123
H 4.575290 -3.381298 -1.249287
H -1.952977 4.415798 0.180607
C -0.940062 -1.268015 -0.999709
H -0.375676 -0.391563 -1.342186
H -1.779888 -1.380922 -1.689985
S 0.122110 -2.754173 -1.040234
C -1.466874 -0.907324 0.400797
H -0.605600 -0.809855 1.064222
C -2.145038 0.469443 0.429516
O -1.675401 1.391180 1.123887
N -2.309148 -1.936068 1.017442
N -3.227951 0.635703 -0.335199
H -3.611717 -0.192791 -0.784030
C -3.961409 1.887116 -0.373314
H -4.312431 2.170107 0.624327
H -3.332832 2.692166 -0.765067
H -4.822375 1.760091 -1.030206
C -3.362705 -2.549904 0.450608
O -3.886064 -2.155782 -0.610671
C -3.907255 -3.756477 1.167982
H -4.992947 -3.660816 1.254297
H -3.693712 -4.641218 0.558488
H -3.474476 -3.900726 2.160521
H -1.925679 -2.372768 1.844628
45

complexC_conf_20 Eopt -1899.558887

C -3.798191 -0.729220 -0.053522
C -3.790171 0.682866 -0.182023
C -2.734636 1.518728 0.211598
C -1.620042 0.922690 0.761810
C -1.591643 -0.473276 0.911926
C -2.644676 -1.280688 0.517923
H -2.813842 2.590121 0.068148
H -0.714317 -0.937802 1.347938
H -2.562743 -2.351159 0.658631
C -0.439330 1.720271 1.194686
O 0.528562 1.230397 1.756139
O -0.574114 3.015514 0.954331
C 0.538488 3.857379 1.302707
H 0.733311 3.798153 2.375352
H 0.232228 4.864411 1.024705
N -5.767602 1.795169 -1.178349
N -4.883831 1.297024 -0.733489
S -5.184146 -1.638638 -0.589356
C -4.704272 -3.356728 -0.288622
H -5.549769 -3.950108 -0.644716
H -3.813700 -3.619355 -0.862993
H -4.557793 -3.543936 0.776746
H 1.422182 3.555793 0.735957
C 2.517563 1.045781 -1.180432
H 3.230811 1.440804 -1.911703
H 2.549954 1.717121 -0.311863
S 0.833944 1.007784 -1.899009
C 3.046907 -0.326684 -0.730674
H 2.956005 -1.033183 -1.563281
C 4.524951 -0.190947 -0.354720
O 4.885637 0.256771 0.748743
N 2.282985 -0.818135 0.397458
N 5.400946 -0.523190 -1.310074
H 5.049525 -0.901058 -2.179881
C 6.834004 -0.363566 -1.142688
H 7.197725 -0.968389 -0.306598
H 7.325652 -0.692366 -2.058341
H 7.090242 0.683662 -0.956633
C 2.482774 -2.030115 0.931259
O 3.334844 -2.817551 0.469933
C 1.615714 -2.413492 2.100631
H 1.161375 -1.547151 2.587627
H 0.819269 -3.077311 1.746128
H 2.217735 -2.964187 2.827729
H 1.577819 -0.205538 0.799726
45
complexC_conf_21 Eopt -1899.560679

C -1.935062 -1.808566 -0.355692
C -1.528581 -1.370044 0.925082
C -0.209173 -1.393395 1.385215
C 0.749793 -1.916252 0.536415
C 0.382320 -2.416451 -0.719287
C -0.944204 -2.357778 -1.154485
H 0.044382 -0.996040 2.362065
H 1.132305 -2.847216 -1.368701
H -1.199464 -2.717896 -2.144895
C 2.180084 -1.804367 0.938113
O 2.576268 -0.979662 1.747157
O 2.961052 -2.671075 0.316135
C 4.374214 -2.567835 0.572884
H 4.730932 -1.578669 0.278130
H 4.832411 -3.340094 -0.042294
N -3.228601 -0.490509 2.501640
N -2.478875 -0.881423 1.788435
S -3.619493 -1.720141 -0.908276
C -3.784621 0.079127 -1.171982
H -2.994285 0.437196 -1.834119
H -4.754638 0.216537 -1.656993
H -3.782064 0.629216 -0.230434
H 4.575149 -2.748929 1.630519
C 0.375329 0.926632 -2.074348
H 0.281932 1.770918 -2.769587
H -0.517618 0.301521 -2.207229
S 1.887963 -0.035873 -2.431465
C 0.311787 1.472963 -0.642933
H 0.320778 0.638599 0.055837
C 1.497924 2.385348 -0.332703
O 1.649394 3.470118 -0.926828
N -0.939616 2.193815 -0.454712
N 2.336163 1.965023 0.618977
H 2.206392 1.033914 1.000837
C 3.563044 2.672609 0.934752
H 3.346097 3.687732 1.278371
H 4.076228 2.131568 1.730112
H 4.219587 2.730260 0.060014
C -1.566500 2.309589 0.728073
O -1.088402 1.843873 1.779754
C -2.873240 3.057290 0.730485
H -3.597646 2.509616 1.339611
H -2.711162 4.034430 1.198466
H -3.278410 3.209211 -0.272928
H -1.395110 2.566770 -1.278571
45
complexC_conf_22 Eopt -1899.559479

C -3.149488 -0.707369 -0.293482
C -1.772305 -0.641388 -0.629717
C -0.995262 0.521719 -0.584708
C -1.623636 1.697574 -0.211835
C -2.985487 1.674739 0.117963
C -3.734256 0.505541 0.078893
H 0.049888 0.488776 -0.863454
H -3.479741 2.592335 0.417403
H -4.782781 0.547627 0.345445
C -0.797443 2.928661 -0.123926
O 0.400066 2.949682 -0.356653
O -1.495455 3.996235 0.245223

C -0.766634 5.229015 0.381158
H 0.012069 5.120515 1.138865
H -1.505829 5.963847 0.695463
N -0.581846 -2.725287 -1.236583
N -1.122471 -1.794519 -0.972891
S -3.979292 -2.240041 -0.363863
C -5.652892 -1.835470 0.190466
H -6.136154 -1.136495 -0.494916
H -6.192457 -2.785404 0.173482
H -5.644018 -1.445698 1.210182
H -0.328616 5.512024 -0.578180
C 2.356642 -0.409579 2.250013
H 2.805677 0.434099 2.786830
H 2.607318 -1.308295 2.823352
S 0.540152 -0.205194 2.136116
C 3.082693 -0.503133 0.896508
H 4.151453 -0.635619 1.099291
C 2.622672 -1.714016 0.070414
O 1.992444 -1.599443 -0.993177
N 2.921967 0.722780 0.148242
N 2.970283 -2.909009 0.566787
H 3.473102 -2.942572 1.443952
C 2.586238 -4.161533 -0.058517
H 2.876859 -4.169494 -1.112438
H 3.100598 -4.973493 0.456220
H 1.506611 -4.326441 0.011631
C 3.692070 1.017399 -0.916403
O 4.681220 0.324615 -1.222442
C 3.297015 2.228861 -1.718317
H 2.453994 1.967929 -2.368058
H 2.984607 3.055075 -1.075218
H 4.136898 2.542592 -2.340727
H 2.035484 1.209845 0.261287
45
complexC_conf_23 Eopt -1899.556468
C -1.263186 2.046899 -0.539512
C -2.388631 1.642689 0.220072
C -2.901218 0.336584 0.252663
C -2.269586 -0.618658 -0.513208
C -1.150059 -0.257825 -1.281411
C -0.660947 1.037236 -1.296837
H -3.762254 0.104289 0.870108
H -0.639320 -1.010298 -1.871763
H 0.216236 1.250773 -1.893489
C -2.782126 -2.017215 -0.476955
O -3.514937 -2.439825 0.402437
O -2.392888 -2.727590 -1.525961
C -2.794319 -4.107423 -1.555250
H -3.883505 -4.180810 -1.583043
H -2.397973 -4.627523 -0.680376
N -3.513799 3.313731 1.661687
N -3.012072 2.568306 1.013636
S -0.702068 3.697387 -0.474616
C 0.927951 3.597811 -1.258959
H 1.555050 2.860917 -0.751470
H 0.844126 3.376719 -2.324470
H 1.363343 4.591956 -1.132097
H -2.361067 -4.512016 -2.468415
C 1.785970 -2.222932 0.179883
H 2.648089 -2.856428 0.421864
H 1.639036 -2.280824 -0.905223
S 0.288033 -2.797867 1.065695
C 2.176401 -0.768695 0.489017
H 1.383289 -0.103044 0.151913
C 2.449451 -0.579302 1.977619
O 3.425763 -1.156555 2.498280
N 3.399806 -0.381097 -0.208840
N 1.660363 0.236446 2.684621
H 1.887079 0.312562 3.669321
C 0.483013 0.945958 2.203212
H -0.228370 0.252429 1.746760
H 0.007462 1.423626 3.060333
H 0.761052 1.722458 1.484857
C 3.427294 0.076575 -1.472891
O 2.391150 0.285948 -2.130760
C 4.786055 0.349388 -2.061771
H 4.892730 -0.230043 -2.983728
H 4.846906 1.410550 -2.323209
H 5.605157 0.100473 -1.383239
H 4.278328 -0.622165 0.234394
45
complexC_conf_24 Eopt -1899.556216
C -0.800395 2.183896 0.313022
C -1.170573 1.332730 1.386317
C -2.167342 0.352822 1.319958
C -2.837521 0.199316 0.123365
C -2.503817 1.023481 -0.963362
C -1.518272 1.995187 -0.872697
H -2.386732 -0.265059 2.183394
H -3.024781 0.903818 -1.906630
H -1.301477 2.600725 -1.743614
C -3.872070 -0.868102 0.034950
O -4.267833 -1.500629 0.999983
O -4.311965 -1.051942 -1.202633
C -5.315192 -2.066628 -1.388086
H -6.207566 -1.819234 -0.809624
H -4.920901 -3.038988 -1.085919
N 0.062830 1.513245 3.528338
N -0.490631 1.436292 2.572201
S 0.470770 3.351284 0.538624
C 0.617821 4.130963 -1.086886
H 0.878960 3.396076 -1.851090
H -0.297871 4.665301 -1.347914
H 1.435064 4.848812 -0.982516
H -5.535409 -2.057874 -2.454129
C 0.512621 -1.215597 -0.139565
H -0.187800 -0.537735 -0.636134
H 0.848433 -0.721107 0.777654

S -0.327791 -2.791913 0.247584
C 1.702720 -1.330787 -1.114222
H 1.316200 -1.727628 -2.054857
C 2.213253 0.070581 -1.461808
O 1.660485 0.708907 -2.379725
N 2.740990 -2.278635 -0.709755
N 3.224801 0.575106 -0.749822
H 3.530724 0.057388 0.069821
C 3.778040 1.890645 -1.009754
H 4.647173 2.029853 -0.366400
H 4.091604 1.975155 -2.053831
H 3.052521 2.681945 -0.798946
C 3.331180 -2.356273 0.495382
O 3.275007 -1.430426 1.330460
C 4.107051 -3.610079 0.796501
H 5.107559 -3.335755 1.142292
H 3.600987 -4.135652 1.613267
H 4.189862 -4.284730 -0.058692
H 2.799267 -3.107513 -1.285553
45
complexC_conf_25 Eopt -1899.560824
C -2.871963 0.011734 -0.620880
C -2.773032 -1.145022 0.194963
C -1.668515 -2.006883 0.230020
C -0.604725 -1.714878 -0.598163
C -0.671786 -0.589689 -1.437920
C -1.770202 0.252279 -1.449661
H -1.670496 -2.859760 0.899093
H 0.178828 -0.331847 -2.067355
H -1.757135 1.115317 -2.104145
C 0.642567 -2.526154 -0.580688
O 1.603491 -2.286579 -1.295587
O 0.610431 -3.512706 0.302500
C 1.796342 -4.322056 0.409642
H 2.646936 -3.699218 0.694277
H 1.991073 -4.825011 -0.539538
N -4.650293 -1.694695 1.716290
N -3.811240 -1.448134 1.036471
S -4.287020 1.021739 -0.527939
C -3.925080 2.353375 -1.697572
H -3.820242 1.966691 -2.713124
H -4.796403 3.010973 -1.651786
H -3.037819 2.911389 -1.392609
H 1.575187 -5.048137 1.189865
C 1.463104 2.270989 -0.760803
H 1.960691 3.212415 -0.496749
H 0.382759 2.448539 -0.688669
S 1.923079 1.761997 -2.459645
C 1.803478 1.247989 0.336184
H 1.226821 0.335508 0.183789
C 3.289726 0.915149 0.361243
O 4.125238 1.778674 0.692483
N 1.456692 1.770231 1.654328
N 3.632088 -0.339473 0.058351
H 2.917233 -0.958775 -0.308004
C 5.018015 -0.761245 -0.032357
H 5.529368 -0.608364 0.921718
H 5.036662 -1.823472 -0.277510
H 5.550498 -0.204372 -0.810576
C 0.250077 1.617225 2.228615
O -0.640822 0.902731 1.733518
C 0.023066 2.350929 3.523770
H -0.786920 3.072710 3.377391
H -0.299750 1.634158 4.284521
H 0.911350 2.878972 3.878290
H 2.100547 2.437596 2.062731
45
complexC_conf_26 Eopt -1899.557260
C 2.833602 0.410763 -0.359673
C 2.225402 0.316818 0.917285
C 1.467652 -0.778841 1.355998
C 1.320765 -1.845376 0.498174
C 1.926630 -1.801393 -0.770027
C 2.660698 -0.703441 -1.189725
H 1.013532 -0.766278 2.340051
H 1.815829 -2.641322 -1.444732
H 3.096877 -0.716372 -2.180376
C 0.472453 -2.988855 0.931084
O -0.288838 -2.935353 1.883791
O 0.657004 -4.073760 0.191122
C -0.162645 -5.213223 0.502602
H 0.130219 -5.980122 -0.212415
H -1.217099 -4.956264 0.378533
N 2.442410 2.222041 2.481720
N 2.345230 1.368056 1.782582
S 3.716105 1.850572 -0.791820
C 4.241991 1.525984 -2.492210
H 4.783641 2.425269 -2.794902
H 4.916273 0.668805 -2.538750
H 3.380381 1.387929 -3.148233
H 0.031130 -5.547675 1.523826
C -1.138629 0.123357 -1.091417
H -0.062433 0.285563 -0.946958
H -1.474479 0.890518 -1.794482
S -1.442636 -1.543971 -1.774025
C -1.787532 0.371854 0.284551
H -1.407377 -0.384001 0.973508
C -1.325982 1.713575 0.864496
O -0.489403 1.746009 1.788548
N -3.242760 0.205249 0.312465
N -1.815683 2.825121 0.310288
H -2.500919 2.715763 -0.435488
C -1.421887 4.148838 0.754596
H -1.959501 4.885635 0.157219
H -1.668993 4.297962 1.810241
H -0.345994 4.300587 0.622802
C -4.131410 0.736063 -0.544365
O -3.841618 1.654492 -1.338069

C -5.529110 0.180149 -0.496392
H -5.692362 -0.504131 0.339453
H -6.241113 1.007679 -0.435040
H -5.715267 -0.356276 -1.433101
H -3.571379 -0.570897 0.869998
45
complexC_conf_27 Eopt -1899.565777
C -2.740823 -0.971836 -0.236862
C -2.016454 -0.661071 0.941278
C -1.456841 0.592513 1.224011
C -1.618315 1.595713 0.294076
C -2.345032 1.334104 -0.879838
C -2.891965 0.088081 -1.140494
H -0.913595 0.751586 2.148715
H -2.477282 2.120905 -1.613712
H -3.435483 -0.059339 -2.065229
C -0.975978 2.911969 0.563486
O -0.229286 3.110404 1.508142
O -1.325932 3.843034 -0.312595
C -0.714067 5.135142 -0.159321
H -0.979319 5.564007 0.809158
H -1.118499 5.740354 -0.968876
N -1.634233 -2.441022 2.621860
N -1.813321 -1.646715 1.870724
S -3.356533 -2.583501 -0.472848
C -4.096865 -2.513264 -2.122349
H -4.456005 -3.527520 -2.312422
H -4.942289 -1.823152 -2.145079
H -3.350104 -2.249592 -2.873791
H 0.370457 5.045029 -0.253485
C 0.942575 -0.203372 -1.614688
H -0.111212 -0.301226 -1.333583
H 1.067294 -0.742140 -2.558766
S 1.385692 1.563477 -1.802618
C 1.756994 -0.959702 -0.548055
H 1.302689 -1.946138 -0.401876
C 3.180063 -1.232239 -1.036615
O 3.355007 -1.932099 -2.053348
N 1.703969 -0.242077 0.707181
N 4.197404 -0.717571 -0.343123
H 3.988172 -0.142463 0.461531
C 5.581632 -0.915251 -0.732054
H 6.218299 -0.410802 -0.005184
H 5.831646 -1.980002 -0.746626
H 5.771372 -0.496094 -1.724864
C 1.738468 -0.824995 1.917445
O 1.872137 -2.056302 2.056570
C 1.628526 0.083140 3.113001
H 1.481957 1.131738 2.842716
H 0.792754 -0.251228 3.735655
H 2.544426 -0.009618 3.705266
H 1.564532 0.763875 0.623133
45
complexC_conf_28 Eopt -1899.562062
C -2.067061 -1.265401 -0.764743
C -2.627324 -0.234891 0.033854
C -2.251140 1.113326 -0.019206
C -1.256424 1.464053 -0.909844
C -0.661714 0.470495 -1.701958
C -1.051897 -0.858920 -1.634791
H -2.733158 1.834578 0.630136
H 0.123732 0.749350 -2.396685
H -0.563466 -1.580085 -2.278073
C -0.778790 2.870657 -1.027214
O 0.209472 3.187573 -1.667998
O -1.562822 3.734585 -0.395788
C -1.152578 5.112251 -0.417555
H -1.119757 5.477698 -1.445951
H -0.173673 5.215075 0.056216
N -4.376082 -0.832744 1.684310
N -3.599560 -0.563618 0.941783
S -2.675607 -2.894203 -0.631303
C -1.435611 -3.864816 -1.523528
H -0.438983 -3.685898 -1.113693
H -1.714868 -4.907311 -1.353127
H -1.464505 -3.656626 -2.594458
H -1.911079 5.645289 0.153248
C 1.975808 1.307868 0.702690
H 1.374841 1.045033 -0.174634
H 2.732778 2.020519 0.361251
S 0.930632 2.109942 1.978750
C 2.748056 0.050909 1.152363
H 3.269481 0.287381 2.086530
C 1.912798 -1.204218 1.461769
O 2.435774 -2.336036 1.462612
N 3.792234 -0.287052 0.192614
N 0.632533 -1.012090 1.779578
H 0.308817 -0.041817 1.872793
C -0.232138 -2.106912 2.170456
H -0.302853 -2.852323 1.372573
H 0.138991 -2.603278 3.073941
H -1.225681 -1.705740 2.374570
C 3.559006 -0.815208 -1.020509
O 2.410069 -1.089687 -1.414723
C 4.760360 -1.078990 -1.889098
H 4.819026 -2.154135 -2.086302
H 4.620724 -0.569080 -2.846952
H 5.698395 -0.747037 -1.438098
H 4.748901 -0.087589 0.450622
45
complexC_conf_29 Eopt -1899.566463
C -0.554041 -2.399002 0.318483
C -0.238122 -1.386615 1.256713
C 0.987168 -0.713646 1.322047
C 1.959349 -1.055827 0.406066
C 1.689614 -2.059630 -0.539003

C	0.470175	-2.716123	-0.583153
H	1.145743	0.055734	2.069604
H	2.449336	-2.330062	-1.263315
H	0.315900	-3.479874	-1.334946
C	3.249300	-0.313014	0.436289
O	3.453090	0.646605	1.162347
O	4.155288	-0.825169	-0.385407
C	5.419171	-0.143892	-0.459074
H	6.002893	-0.702981	-1.188436
H	5.910194	-0.156960	0.516090
N	-1.968059	-0.681672	2.882467
N	-1.195232	-0.998100	2.156052
S	-2.127849	-3.144069	0.348901
C	-2.065433	-4.314411	-1.029030
H	-1.902500	-3.795084	-1.975446
H	-1.304823	-5.079478	-0.862514
H	-3.051341	-4.784968	-1.042747
H	5.267405	0.883877	-0.796541
C	-0.065289	0.947925	-1.961726
H	-0.660345	0.881839	-2.878318
H	-0.123633	-0.029898	-1.467191
S	1.677257	1.349706	-2.350727
C	-0.761302	1.976276	-1.046428
H	-0.850607	2.931315	-1.578543
C	-2.158273	1.455504	-0.710715
O	-2.347450	0.577256	0.147606
N	0.029856	2.157080	0.151125
N	-3.154303	1.948181	-1.456160
H	-2.947969	2.674996	-2.128467
C	-4.521821	1.478851	-1.328045
H	-4.585385	0.405739	-1.531653
H	-4.906019	1.668686	-0.321275
H	-5.137998	2.014822	-2.050097
C	-0.362321	2.863846	1.218988
O	-1.481693	3.412805	1.272921
C	0.602188	2.940107	2.371893
H	0.677312	3.977506	2.709090
H	0.201450	2.344375	3.199339
H	1.596562	2.565631	2.117589
H	0.991405	1.832083	0.083046

45

complexC_conf_30 Eopt -1899.564288

C	0.348964	-2.314852	-0.681344
C	-0.642680	-2.401538	0.329857
C	-1.814525	-1.634467	0.372069
C	-2.009638	-0.713893	-0.636719
C	-1.047869	-0.589994	-1.650970
C	0.095297	-1.372356	-1.683093
H	-2.516314	-1.762433	1.187522
H	-1.201525	0.142662	-2.436660
H	0.800356	-1.233578	-2.492758
C	-3.192322	0.190135	-0.641333
O	-3.279503	1.168906	-1.364325
O	-4.142337	-0.198033	0.198256
C	-5.295790	0.653916	0.304549
H	-5.806208	0.716179	-0.658658
H	-4.992782	1.647795	0.641545
N	-0.300072	-3.982433	2.207087
N	-0.452133	-3.278688	1.365441
S	1.753668	-3.343116	-0.606068
C	2.745903	-2.798283	-2.017105
H	3.018791	-1.745927	-1.918327
H	3.649928	-3.410661	-1.978371
H	2.225420	-2.986345	-2.958170
H	-5.935387	0.177771	1.045812
C	0.236738	1.019732	1.877662
H	0.807129	0.906270	2.805249
H	0.208353	0.034688	1.394270
S	-1.463622	1.596526	2.232241
C	1.045317	1.963582	0.964857
H	1.199627	2.918577	1.481803
C	2.402149	1.318440	0.681160
O	2.536717	0.404543	-0.150309
N	0.309482	2.186128	-0.260901
N	3.418388	1.745232	1.440464
H	3.260433	2.508623	2.084648
C	4.745296	1.163102	1.355169
H	4.715106	0.092647	1.578926
H	5.168022	1.301023	0.355352
H	5.386237	1.660333	2.083333
C	0.790414	2.847365	-1.321669
O	1.948039	3.313430	-1.339729
C	-0.122516	2.973500	-2.511542
H	-0.081787	3.999079	-2.888120
H	0.243984	2.308399	-3.301192
H	-1.157180	2.708941	-2.282018
H	-0.674348	1.932621	-0.228022

45

complexC_conf_4 Eopt -1899.559479

C	-3.149765	-0.709168	-0.292922
C	-1.772399	-0.641738	-0.627973
C	-0.996928	0.522473	-0.583774
C	-1.627252	1.697938	-0.213093
C	-2.989381	1.673701	0.115664
C	-3.736521	0.503472	0.077456
H	0.048614	0.490524	-0.861152
H	-3.485072	2.591059	0.413478
H	-4.785315	0.544452	0.343116
C	-0.802684	2.930177	-0.126410
O	0.395247	2.951868	-0.356988
O	-1.502522	3.997820	0.239015
C	-0.775214	5.231652	0.373512
H	-1.515681	5.966374	0.685064
H	-0.335971	5.513071	-0.585738
N	-0.577478	-2.724501	-1.229591
N	-1.120305	-1.794350	-0.968265
S	-3.977479	-2.242996	-0.362284
C	-5.651619	-1.840454	0.191925

H	-6.136116	-1.142968	-0.494103
H	-6.189684	-2.791256	0.176067
H	-5.643115	-1.449571	1.211222
H	0.002402	5.125577	1.132675
C	2.351827	-0.420835	2.250668
H	2.800344	0.419065	2.793803
H	2.599731	-1.323254	2.819359
S	0.535999	-0.213365	2.132568
C	3.081678	-0.507068	0.898737
H	4.149875	-0.640487	1.103861
C	2.624412	-1.713260	0.064413
O	1.997143	-1.592650	-1.000258
N	2.923104	0.723047	0.157045
N	2.971209	-2.910919	0.554877
H	3.472824	-2.949178	1.432538
C	2.592704	-4.160168	-0.080292
H	1.513442	-4.328984	-0.014431
H	2.886446	-4.159854	-1.133380
H	3.108028	-4.974120	0.430313
C	3.698028	1.025619	-0.901628
O	4.687789	0.334273	-1.209386
C	3.307570	2.242600	-1.697040
H	2.501097	1.971337	-2.387840
H	2.946338	3.049465	-1.055374
H	4.164943	2.587261	-2.278086
H	2.036845	1.210188	0.271089

45

complexC_conf_5 Eopt -1899.563500

C	-1.549440	-2.005527	0.163092
C	-0.701335	-1.423763	1.136648
C	0.698051	-1.396846	1.067251
C	1.292354	-1.983823	-0.030282
C	0.484928	-2.570074	-1.020872
C	-0.894522	-2.591859	-0.926857
H	1.268553	-0.918777	1.855108
H	0.958581	-3.025669	-1.884217
H	-1.465569	-3.057052	-1.720268
C	2.771451	-1.993830	-0.197638
O	3.324689	-2.266075	-1.250502
O	3.414928	-1.679633	0.918227
C	4.848630	-1.598311	0.842497
H	5.143821	-0.862897	0.091106
H	5.264403	-2.578515	0.600013
N	-1.736680	-0.344774	3.111264
N	-1.272445	-0.825173	2.228632
S	-3.276223	-1.956939	0.387850
C	-3.920045	-2.676055	-1.141314
H	-3.626320	-3.722714	-1.240375
H	-5.006873	-2.615352	-1.045663
H	-3.601611	-2.092305	-2.007605
H	5.168937	-1.281783	1.833611
C	-0.695291	2.417384	-2.241971
H	-0.060193	2.821139	-3.039019
H	-1.648853	2.951133	-2.307987
S	-0.936335	0.612587	-2.451834
C	-0.021095	2.789829	-0.908214
H	0.145815	3.872665	-0.889164
C	-0.888437	2.436246	0.310565
O	-0.504771	1.656655	1.196455
N	1.258507	2.126345	-0.824138
N	-2.080054	3.044184	0.380342
H	-2.368507	3.652120	-0.374116
C	-3.028947	2.751902	1.439588
H	-3.347565	1.704800	1.402597
H	-2.588169	2.950469	2.420582
H	-3.901010	3.392664	1.306690
C	2.230976	2.485748	0.028835
O	2.175240	3.535555	0.699457
C	3.408017	1.553963	0.133003
H	3.356454	1.039665	1.098461
H	3.425486	0.807911	-0.665254
H	4.333778	2.135090	0.108504
H	1.272011	1.191056	-1.227059

45

complexC_conf_6 Eopt -1899.561830

C	1.896325	-1.880885	0.458929
C	1.270545	-0.885105	1.248825
C	1.528820	0.486876	1.160646
C	2.465160	0.901084	0.233083
C	3.109830	-0.052449	-0.567195
C	2.843119	-1.408829	-0.455665
H	0.987611	1.182068	1.790302
H	3.841312	0.278306	-1.297197
H	3.373929	-2.101608	-1.096642
C	2.754774	2.346277	0.025770
O	3.373967	2.771768	-0.936169
O	2.278824	3.110691	0.997892
C	2.434998	4.531025	0.839251
H	3.495372	4.791604	0.823305
H	1.946985	4.970906	1.707297
N	-0.516191	-1.566862	2.823297
N	0.287032	-1.265092	2.124440
S	1.451370	-3.554890	0.661922
C	2.226410	-4.357132	-0.763022
H	3.314875	-4.343090	-0.683729
H	1.878762	-5.392324	-0.729365
H	1.892370	-3.894965	-1.694839
H	1.947501	4.858775	-0.081352
C	-2.112168	1.943028	-1.768710
H	-2.922978	2.671908	-1.882426
H	-1.751471	1.721415	-2.778357
S	-0.761898	2.632758	-0.737035
C	-2.749365	0.661825	-1.197554
H	-3.586847	0.374613	-1.843090
C	-1.766366	-0.518611	-1.164573
O	-1.386339	-1.037856	-0.104189
N	-3.259010	0.931796	0.125504
N	-1.362557	-0.968827	-2.360437

H -1.684782 -0.493977 -3.193170
C -0.377700 -2.026579 -2.502370
H 0.601589 -1.699868 -2.138555
H -0.685608 -2.917005 -1.947634
H -0.294383 -2.279862 -3.559561
C -4.120007 0.126691 0.767835
O -4.676182 -0.836091 0.202998
C -4.402153 0.450358 2.210347
H -3.892233 1.354915 2.550226
H -5.481157 0.569917 2.346136
H -4.078012 -0.395264 2.825575
H -2.714796 1.607015 0.657426
45
complexC_conf_7 Eopt -1899.563005
C 2.462497 -1.313418 -0.226549
C 2.214353 -0.621387 0.988619
C 2.058624 0.767654 1.099489
C 2.133758 1.511921 -0.062023
C 2.347364 0.862780 -1.285718
C 2.524811 -0.512182 -1.368328
H 1.892108 1.225118 2.066371
H 2.394839 1.446033 -2.198939
H 2.705910 -0.957352 -2.338569
C 1.944284 2.984561 0.039409
O 2.152444 3.621998 1.058450
O 1.525069 3.520982 -1.099257
C 1.303490 4.942404 -1.101320
H 2.231082 5.467101 -0.863479
H 0.524363 5.196542 -0.379545
N 1.968483 -1.926596 3.078106
N 2.090814 -1.343744 2.143153
S 2.677834 -3.044181 -0.205194
C 2.661622 -3.483166 -1.960133
H 1.737584 -3.143770 -2.432855
H 3.538430 -3.086757 -2.475413
H 2.697706 -4.575037 -1.979215
H 0.978701 5.178837 -2.113097
C -1.395208 1.469754 -0.305822
H -1.828961 2.384662 -0.724346
H -0.436170 1.313363 -0.812927
S -1.160436 1.652319 1.499433
C -2.318414 0.303308 -0.719103
H -2.544899 0.404260 -1.785358
C -1.591887 -1.033871 -0.581900
O -0.674987 -1.311209 -1.378104
N -3.554361 0.379148 0.028005
N -1.963969 -1.862342 0.395782
H -2.685011 -1.555824 1.034704
C -1.289823 -3.122227 0.645273
H -0.253615 -2.956944 0.956902
H -1.820407 -3.646411 1.440313
H -1.289230 -3.744265 -0.254027
C -4.714512 -0.174896 -0.362005
O -4.823286 -0.795027 -1.437657
C -5.887506 -0.010866 0.566531
H -6.697184 0.491159 0.028117
H -6.244091 -1.003470 0.858908
H -5.643233 0.561771 1.464567
H -3.477706 0.829344 0.938772
45
complexC_conf_9 Eopt -1899.560767
C 3.332111 -0.289624 -0.015508
C 2.292043 0.392368 -0.695274
C 1.094822 -0.197370 -1.116737
C 0.897324 -1.530452 -0.826601
C 1.904508 -2.244959 -0.157679
C 3.093118 -1.647480 0.229377
H 0.350394 0.405039 -1.624520
H 1.755054 -3.293779 0.073070
H 3.836481 -2.247148 0.739615
C -0.412498 -2.137171 -1.186061
O -1.275117 -1.541607 -1.814882
O -0.543031 -3.383295 -0.759719
C -1.806283 -4.023427 -1.015523
H -1.705825 -5.026006 -0.603468
H -1.994763 -4.066554 -2.089954
N 2.541656 2.815474 -1.153418
N 2.436670 1.731947 -0.952256
S 4.774766 0.570965 0.448083
C 5.748951 -0.682287 1.315759
H 6.654266 -0.161625 1.636832
H 5.216895 -1.052120 2.194482
H 6.023498 -1.499893 0.646453
H -2.606279 -3.479382 -0.507851
C -1.193177 0.813353 1.745657
H -1.669810 1.395751 2.544129
H -0.216499 1.274171 1.551550
S -0.995429 -0.935353 2.253357
C -2.026466 1.013593 0.469067
H -1.513541 0.561768 -0.379045
C -3.413915 0.400083 0.605552
O -4.238278 0.868797 1.414558
N -2.200915 2.432815 0.176663
N -3.696689 -0.623124 -0.204851
H -2.939311 -1.018036 -0.754108
C -4.923839 -1.388356 -0.083405
H -4.991745 -1.872835 0.896793
H -5.795378 -0.742065 -0.216589
H -4.930182 -2.152966 -0.860868
C -1.341701 3.157576 -0.559417
O -0.369857 2.647296 -1.148881
C -1.612242 4.635599 -0.655595
H -0.748936 5.174354 -0.252445
H -1.714268 4.907099 -1.710637
H -2.510555 4.943981 -0.115767
H -2.920215 2.922466 0.696038
40

cysteine_pep_anion_6sol_conf_10
Eopt -1351.989867
C 1.338210 -1.865524 0.010806
H 0.785786 -2.003099 -0.927472
H 1.167346 -2.757814 0.621691
S 3.124397 -1.689856 -0.345887
C 0.691176 -0.690058 0.768341
H 1.191213 -0.563073 1.732037
C 0.791008 0.601864 -0.040477
O 0.051940 0.786872 -1.037900
N -0.708161 -1.039999 0.991442
N 1.688798 1.495626 0.345300
H 2.338205 1.236160 1.089650
C 1.955442 2.700689 -0.421632
H 1.064139 3.331417 -0.469149
H 2.754361 3.250891 0.075654
H 2.268791 2.452768 -1.440617
C -1.631106 -0.334329 1.663478
O -2.835267 -0.681081 1.632292
C -1.203862 0.878279 2.434415
H -1.952788 1.094908 3.197701
H -0.224226 0.756207 2.902784
H -1.154306 1.733108 1.749033
H -1.071976 -1.790803 0.412991
O -1.815901 2.926514 -0.681840
H -2.600545 2.354676 -0.545368
H -1.097707 2.284988 -0.841474
O -2.172540 -0.562497 -2.050465
H -1.360213 -0.223704 -1.616708
H -2.456660 -1.336264 -1.520475
O 2.141408 -0.140628 -2.982488
H 2.554683 -0.598672 -2.207527
H 1.357313 0.284744 -2.595711
O 3.804479 0.286928 2.037816
H 4.572325 0.808057 1.762694
H 3.686405 -0.385156 1.314015
O -3.470860 -2.480120 -0.410535
H -3.357376 -1.886423 0.364217
H -4.354092 -2.278793 -0.750436
O -3.893690 1.022980 -0.487890
H -3.710847 0.578121 0.359211
H -3.376378 0.488801 -1.131241
40
cysteine_pep_anion_6sol_conf_12
Eopt -1351.985617
C 1.506900 -0.587281 1.675529
H 1.116995 -1.585682 1.444866
H 1.313800 -0.398654 2.736449
S 3.309317 -0.531281 1.361412
C 0.685454 0.447794 0.888908
H 1.084964 1.442806 1.097244
C 0.731162 0.169442 -0.614847
O 0.073292 -0.777931 -1.102831
N -0.695025 0.384679 1.360848
N 1.475763 0.973046 -1.361965
H 2.066373 1.661094 -0.892798
C 1.638505 0.766993 -2.790408
H 2.291955 1.550134 -3.175463
H 2.086575 -0.209333 -2.999062
H 0.671461 0.826884 -3.297518
C -1.688609 1.234586 1.064428
O -2.860025 0.987063 1.436983
C -1.388509 2.467175 0.264358
H -1.351876 2.195678 -0.797717
H -2.195471 3.187187 0.407111
H -0.433946 2.925648 0.532689
H -0.983043 -0.483789 1.803383
O -1.279510 -2.827141 0.298893
H -2.120719 -2.415298 0.591330
H -0.781100 -2.096445 -0.121116
O -2.557937 -0.725377 -2.080256
H -1.645398 -0.597168 -1.750420
H -2.841584 -1.550160 -1.658247
O 3.129269 -2.401988 -1.191708
H 3.218659 -1.804876 -0.402418
H 3.731261 -3.136605 -1.009601
O 3.482845 2.450066 0.261097
H 4.236808 2.477778 -0.345233
H 3.515167 1.538961 0.657692
O -4.403664 1.078270 -0.971638
H -4.034459 1.201375 -0.077632
H -3.755275 0.474883 -1.394071
O -3.696392 -1.644234 1.139530
H -3.390443 -0.723684 1.294913
H -4.227376 -1.586415 0.332501
40
cysteine_pep_anion_6sol_conf_15
Eopt -1351.983751
C -0.840668 -0.154354 1.792919
H -0.902673 0.940030 1.764868
H -0.272587 -0.424928 2.688206
S -2.516965 -0.880523 1.905739
C -0.007727 -0.627201 0.588499
H -0.013684 -1.718271 0.560076
C -0.567614 -0.067741 -0.720616
O -0.365282 1.128487 -1.035432
N 1.365874 -0.173967 0.768050
N -1.258398 -0.896678 -1.491509
H -1.461228 -1.832198 -1.135097
C -1.919458 -0.458478 -2.708800
H -1.198758 -0.000404 -3.391084
H -2.359890 -1.332163 -3.189845
H -2.709475 0.266026 -2.489334
C 2.471934 -0.721952 0.241286
O 3.573858 -0.140652 0.367689
C 2.358225 -2.038547 -0.471128
H 1.946752 -2.803956 0.194793
H 1.693050 -1.952245 -1.336045
H 3.345969 -2.350889 -0.810542
H 1.488795 0.739522 1.194854

O -2.699619 2.741971 -1.091842
H -3.317579 2.086607 -0.699038
H -1.865899 2.240464 -1.170378
O 0.636793 3.169580 0.552432
H 0.278284 2.427187 0.016338
H 0.510906 3.955349 0.002452
O -4.380850 0.800837 0.010460
H -3.762951 0.295026 0.608531
H -4.955951 1.293112 0.613706
O -2.151178 -3.336580 -0.072664
H -3.010835 -3.484339 -0.491757
H -2.321575 -2.634029 0.609682
O 5.935039 -1.188974 -0.682985
H 5.099091 -0.827760 -0.315885
H 6.605804 -0.527133 -0.467746
O 3.421740 2.706387 0.510561
H 3.519525 1.733819 0.492432
H 2.451843 2.855257 0.524501
40
cysteine_pep_anion_6sol_conf_17
Eopt -1351.981921
C -0.500373 -1.433635 1.389612
H -0.600502 -0.636025 2.135477
H 0.327590 -2.074461 1.710039
S -2.047991 -2.411102 1.293525
C -0.068391 -0.793599 0.058872
H 0.033326 -1.566099 -0.703665
C -1.072524 0.251373 -0.412991
O -1.169392 1.339304 0.203588
N 1.216209 -0.116845 0.201949
N -1.807982 -0.041071 -1.475096
H -1.775326 -0.998374 -1.829874
C -2.856716 0.843094 -1.954060
H -2.435673 1.804174 -2.259123
H -3.331022 0.372914 -2.815439
H -3.609180 1.013595 -1.177924
C 2.424169 -0.653459 -0.011580
O 3.456422 0.015545 0.233336
C 2.513702 -2.046293 -0.566033
H 2.101807 -2.073842 -1.580570
H 3.559339 -2.354299 -0.598818
H 1.947131 -2.752942 0.047806
H 1.201200 0.803401 0.632536
O 0.722289 3.447970 -0.359921
H 1.589754 3.154768 -0.006664
H 0.104624 2.722822 -0.155772
O -2.719757 3.688534 0.068377
H -2.248476 2.831183 0.040499
H -2.011854 4.346784 0.034593
O -1.952948 -2.977872 -1.840552
H -2.025394 -2.895654 -0.852303
H -2.864287 -3.093433 -2.144657
O -3.612608 0.339283 1.773128
H -2.985774 0.943824 1.343634
H -3.212286 -0.554368 1.617139
O 6.025089 -0.971090 -0.163435
H 5.104872 -0.653658 -0.034362
H 5.932335 -1.764598 -0.708089
O 3.283669 2.764323 0.599153
H 3.359588 1.791265 0.478887
H 3.847148 3.143200 -0.090029
40
cysteine_pep_anion_6sol_conf_18
Eopt -1351.982758
C -0.646741 1.373373 1.221598
H -0.002462 1.669473 2.055642
H -1.668732 1.319159 1.606156
S -0.542277 2.611247 -0.127215
C -0.226162 -0.052393 0.816750
H -0.275069 -0.677310 1.713496
C -1.227726 -0.651947 -0.166406
O -2.415063 -0.807067 0.204258
N 1.134546 -0.040747 0.316529
N -0.822858 -0.985338 -1.383121
H 0.146709 -0.829664 -1.627268
C -1.722596 -1.537738 -2.381769
H -1.153249 -1.710589 -3.294676
H -2.144836 -2.485144 -2.036824
H -2.537301 -0.839453 -2.592535
C 2.057269 -1.006249 0.444711
O 3.197136 -0.850776 -0.049739
C 1.702135 -2.255221 1.198130
H 1.558870 -2.019645 2.258511
H 0.774118 -2.698681 0.825023
H 2.514365 -2.975992 1.100491
H 1.401599 0.794140 -0.203521
O -4.852174 -1.747227 -0.800597
H -5.124126 -1.045569 -1.408004
H -3.952515 -1.483116 -0.519645
O -3.276317 -0.651207 2.841689
H -2.945183 -0.664788 1.917844
H -4.073056 -1.198485 2.823842
O -3.518416 1.736601 -0.930378
H -2.608169 2.087359 -0.766094
H -3.463674 0.826910 -0.593519
O 2.538386 3.393781 0.379524
H 3.036664 2.749229 -0.166487
H 1.589432 3.187945 0.202334
O 5.238403 -2.745123 0.025450
H 4.485300 -2.115856 0.014200
H 5.265569 -3.109694 -0.869470
O 4.012815 1.548650 -1.180933
H 3.735990 0.695241 -0.778191
H 4.919138 1.687176 -0.871985
40
cysteine_pep_anion_6sol_conf_21
Eopt -1351.988291
C 1.104058 0.283062 1.805478
H 0.737560 0.679290 2.757453
H 2.117247 0.670330 1.663199

S 1.132946 -1.549619 1.895469
C 0.219048 0.893070 0.696904
H 0.128599 1.961535 0.912745
C 0.920079 0.815019 -0.658056
O 1.822667 1.648539 -0.912503
N -1.098792 0.291884 0.724281
N 0.569223 -0.128080 -1.521453
H -0.064809 -0.869768 -1.223382
C 1.260354 -0.304493 -2.787882
H 0.858171 -1.194397 -3.272594
H 1.098646 0.560216 -3.437699
H 2.336191 -0.431770 -2.634885
C -2.231089 0.822833 0.242241
O -3.295411 0.162104 0.270539
C -2.198754 2.221664 -0.301172
H -1.950976 2.928352 0.498302
H -1.441694 2.325793 -1.084950
H -3.177546 2.472414 -0.710693
H -1.141484 -0.664751 1.073734
O 4.336576 0.293082 -0.666746
H 3.964208 -0.612547 -0.585272
H 3.545285 0.851949 -0.779005
O 2.572428 3.470610 1.072038
H 2.313002 2.825287 0.378872
H 2.226006 3.086178 1.889466
O 3.174867 -2.241378 -0.391351
H 2.510221 -2.016001 0.316918
H 2.645187 -2.343538 -1.195277
O -0.516899 -2.738654 -0.649265
H -1.454185 -2.713225 -0.355683
H -0.006516 -2.464850 0.147656
O -3.185664 -2.603802 0.201514
H -3.250271 -1.629055 0.311954
H -3.151104 -2.955117 1.102149
O -5.776314 1.143956 -0.521636
H -4.878353 0.830272 -0.278428
H -5.883717 0.888106 -1.447664
40
cysteine_pep_anion_6sol_conf_22
Eopt -1351.981290
C -0.750158 -1.411522 -1.800265
H 0.203829 -1.499726 -2.330358
H -1.537730 -1.450182 -2.561115
S -0.936625 -2.841259 -0.655604
C -0.772652 0.001773 -1.199502
H -0.633158 0.700550 -2.029112
C -2.078552 0.467086 -0.547322
O -2.279659 1.694727 -0.385584
N 0.296200 0.240096 -0.236646
N -2.961501 -0.445380 -0.172094
H -2.668488 -1.426274 -0.221537
C -4.210759 -0.093251 0.479656
H -4.810222 0.553800 -0.166110
H -4.763209 -1.012097 0.676529
H -4.025606 0.424678 1.425423
C 1.497223 0.766018 -0.500918
O 2.351244 0.864667 0.414240
C 1.802457 1.240567 -1.893247
H 1.141337 2.067019 -2.172253
H 2.836548 1.584224 -1.934582
H 1.661521 0.432756 -2.618054
H 0.167064 -0.144060 0.700628
O -0.407550 3.745567 -0.579403
H 0.376014 3.467674 -0.085500
H -0.999455 2.962938 -0.561568
O -1.884217 2.460329 2.321131
H -2.101861 2.171238 1.413613
H -1.073682 2.977124 2.212717
O -0.297463 -1.508317 2.211580
H -0.489720 -2.008314 1.381632
H 0.669976 -1.567926 2.325323
O 2.243105 -2.856474 -0.175110
H 2.372038 -2.337683 0.645003
H 1.263520 -2.882039 -0.294276
O 5.013784 1.480711 -0.072827
H 4.064551 1.271733 0.068439
H 5.109835 1.519104 -1.034227
O 2.516733 -1.255348 2.152959
H 2.510730 -0.420275 1.633254
H 3.396092 -1.318073 2.550698
40
cysteine_pep_anion_6sol_conf_25
Eopt -1351.981584
C 0.432109 -1.408561 -1.403175
H 0.548287 -0.602909 -2.137692
H -0.398784 -2.037303 -1.739484
S 1.966843 -2.404168 -1.303319
C -0.003297 -0.782993 -0.067294
H -0.104702 -1.563499 0.687182
C 0.996687 0.261231 0.414418
O 1.075168 1.358353 -0.187348
N -1.287261 -0.103360 -0.202995
N 1.733303 -0.035326 1.475443
H 1.710897 -0.997714 1.817909
C 2.769255 0.853364 1.973187
H 3.576545 0.964695 1.242137
H 2.349757 1.837244 2.195830
H 3.174938 0.423671 2.889173
C -2.497091 -0.639307 0.007752
O -3.527777 0.037654 -0.218116
C -2.586352 -2.041860 0.537691
H -3.631283 -2.352446 0.558122
H -2.013764 -2.737222 -0.083365
H -2.181640 -2.084839 1.554678
H -1.268051 0.827694 -0.609835
O 2.927716 3.450270 -0.287984
H 3.655134 3.244193 0.314805
H 2.306353 2.700591 -0.181719
O -0.690345 3.484227 0.406199
H -0.092616 2.738570 0.199845

H -0.381828 4.204781 -0.161274
O 1.891505 -2.971223 1.830236
H 1.953028 -2.889688 0.841071
H 2.806271 -3.086114 2.124088
O 3.874420 0.123516 -1.552044
H 3.376402 0.845274 -1.140102
H 3.289638 -0.672452 -1.442559
O -6.101439 -0.914823 0.252906
H -5.186567 -0.597132 0.089729
H -6.670182 -0.243701 -0.147846
O -3.245611 2.828123 -0.614427
H -3.354919 1.866240 -0.470065
H -2.351667 3.029522 -0.265508
40
cysteine_pep_anion_6sol_conf_26
Eopt -1351.984890
C -0.922155 -1.327340 -1.526320
H -0.376001 -1.410545 -2.471675
H -1.988469 -1.333781 -1.768401
S -0.526581 -2.757761 -0.445211
C -0.599054 0.063336 -0.947529
H -0.845170 0.799743 -1.718734
C -1.513811 0.382650 0.233860
O -2.707840 0.677655 -0.005345
N 0.814865 0.161214 -0.642725
N -1.023754 0.341685 1.465798
H -0.101536 -0.066780 1.615638
C -1.862392 0.551274 2.634050
H -2.340789 1.532618 2.588012
H -2.635485 -0.219947 2.707881
H -1.228157 0.507050 3.519588
C 1.538554 1.285891 -0.539527
O 2.738477 1.229405 -0.184756
C 0.889595 2.593988 -0.889032
H -0.033957 2.748145 -0.322599
H 1.581189 3.409941 -0.677026
H 0.633725 2.607182 -1.954154
H 1.281263 -0.705935 -0.391526
O -3.594063 0.903187 -2.640252
H -2.972128 0.374460 -3.159304
H -3.272210 0.811442 -1.717035
O -5.079048 0.963673 1.448103
H -4.212474 0.853542 1.003843
H -5.729309 0.860914 0.740231
O 1.086560 -1.639508 2.148500
H 0.596551 -2.054943 1.399479
H 1.987710 -1.492497 1.792462
O 2.645483 -2.988651 -1.018902
H 3.025799 -2.384342 -0.350846
H 1.670661 -2.951979 -0.857472
O 4.360529 3.479376 0.043925
H 3.758035 2.710437 -0.054100
H 3.819895 4.241848 -0.203761
O 3.582111 -1.148939 0.942304
H 3.407633 -0.260674 0.557205
H 4.418629 -1.080394 1.422529
40
cysteine_pep_anion_6sol_conf_28
Eopt -1351.985741
C 1.595150 -1.657201 -1.371053
H 1.698047 -1.412845 -2.432719
H 1.570289 -2.749327 -1.295073
S 3.061849 -1.017178 -0.462469
C 0.215016 -1.146728 -0.938680
H -0.509269 -1.552973 -1.647394
C -0.281564 -1.593025 0.443970
O -1.514662 -1.708501 0.646666
N 0.141835 0.308625 -0.990885
N 0.609637 -1.792763 1.403515
H 1.593758 -1.623264 1.171784
C 0.229935 -2.150434 2.758988
H 1.140595 -2.292825 3.341066
H -0.369266 -1.358225 3.217261
H -0.347996 -3.078566 2.764578
C -0.907180 1.066810 -1.351722
O -0.892698 2.297903 -1.118398
C -2.083632 0.435017 -2.038837
H -2.695916 -0.094514 -1.302147
H -2.685715 1.222937 -2.493147
H -1.777830 -0.278304 -2.808801
H 0.889468 0.800568 -0.507238
O -3.098103 -2.878127 -1.332620
H -3.017092 -2.361516 -2.146066
H -2.539159 -2.399634 -0.683592
O -2.235532 0.725161 2.006643
H -2.068072 -0.110715 1.531379
H -1.396473 1.233870 1.927734
O 2.526803 1.603748 1.484861
H 2.558483 0.680722 1.154499
H 2.796168 2.104402 0.689220
O 3.291617 2.097427 -1.236608
H 4.235752 2.307833 -1.264415
H 3.258274 1.111355 -1.151252
O -0.114414 2.472815 1.605430
H -0.332702 2.569698 0.656516
H 0.801715 2.114822 1.604592
O -3.443693 2.580539 0.270849
H -2.686645 2.692606 -0.332209
H -3.127193 1.883354 0.885943
40
cysteine_pep_anion_6sol_conf_5
Eopt -1351.986530
C 1.339335 -1.841565 -0.250278
H 0.881442 -1.809640 -1.246788
H 1.115444 -2.823311 0.179086
S 3.152186 -1.634830 -0.385565
C 0.626505 -0.805209 0.638128
H 1.091570 -0.806026 1.627135
C 0.706106 0.595199 0.028833
O -0.040619 0.915111 -0.925285

N -0.767273 -1.219373 0.763903
N 1.580692 1.439635 0.558372
H 2.218048 1.090416 1.275954
C 1.819128 2.758213 -0.003059
H 0.912467 3.367224 0.041063
H 2.600454 3.242084 0.583682
H 2.146993 2.682347 -1.043971
C -1.720933 -0.646317 1.514832
O -2.913128 -1.017447 1.412469
C -1.341782 0.455683 2.459407
H -2.112136 0.546550 3.226635
H -0.369738 0.291324 2.930704
H -1.298110 1.399511 1.902584
H -1.102640 -1.872714 0.062031
O -2.276832 -0.267633 -2.162829
H -2.964613 0.210292 -1.650914
H -1.444665 -0.003016 -1.718081
O -1.940410 2.905371 -0.347654
H -1.220029 2.277130 -0.565287
H -2.095178 3.391839 -1.169816
O 2.976798 0.475683 -2.746746
H 3.051737 -0.178451 -2.003687
H 2.871599 -0.065206 -3.540804
O 3.621393 0.090261 2.233940
H 4.403730 0.637362 2.075289
H 3.565442 -0.504335 1.439336
O -4.004344 1.030038 -0.336404
H -3.768947 0.370949 0.342814
H -3.325493 1.735614 -0.242568
O -3.367222 -2.581559 -0.927113
H -3.356401 -2.167504 -0.041206
H -2.945122 -1.891617 -1.480280
40
cysteine_pep_anion_6sol_conf_6
Eopt -1351.990023
C -1.633640 -0.469955 -1.672459
H -1.263219 -1.495546 -1.560896
H -1.519159 -0.195762 -2.725911
S -3.400877 -0.397257 -1.197178
C -0.710885 0.466251 -0.874622
H -1.044706 1.495758 -1.018753
C -0.717440 0.122404 0.615478
O -0.130320 -0.906733 1.030462
N 0.645299 0.330162 -1.398309
N -1.352717 0.945842 1.435761
H -1.916057 1.694317 1.028490
C -1.483843 0.664120 2.855015
H -2.030196 -0.269891 3.020045
H -0.497683 0.583262 3.319709
H -2.030136 1.486462 3.317230
C 1.678303 1.157139 -1.175272
O 2.836119 0.821381 -1.519696
C 1.431268 2.472724 -0.497338
H 2.283158 3.130069 -0.676135
H 0.514877 2.956069 -0.844105
H 1.340285 2.304885 0.582257
H 0.897552 -0.590521 -1.748157
O 0.869727 -3.008790 -0.585033
H 1.780436 -2.667854 -0.709692
H 0.434731 -2.298582 -0.070571
O 2.606413 -0.735869 1.843186
H 1.676205 -0.652326 1.557503
H 3.049673 0.097394 1.579196
O -2.673715 -2.519837 1.101884
H -2.993570 -1.869716 0.426019
H -1.770749 -2.214959 1.293078
O -3.352312 2.560384 -0.030377
H -4.070136 2.630354 0.615000
H -3.466568 1.659353 -0.435877
O 3.431971 -1.811177 -0.631851
H 3.269822 -0.976409 -1.115577
H 3.252437 -1.555078 0.298746
O 4.294231 1.315864 0.847759
H 3.940733 1.211211 -0.059673
H 5.088751 0.763712 0.865281
40
cysteine_pep_anion_6sol_conf_7
Eopt -1351.984969
C -0.830238 -1.342848 -1.523237
H -0.263647 -1.401906 -2.458212
H -1.890624 -1.396398 -1.784462
S -0.389577 -2.754012 -0.433574
C -0.577430 0.058775 -0.936130
H -0.840235 0.787217 -1.709327
C -1.527729 0.332807 0.228037
O -2.733291 0.553477 -0.032185
N 0.825336 0.210033 -0.604596
N -1.056645 0.332304 1.467853
H -0.111834 -0.010804 1.634555
C -1.923889 0.501951 2.621503
H -2.660184 -0.305520 2.682259
H -1.303350 0.487642 3.517645
H -2.448164 1.459217 2.567085
C 1.511515 1.357833 -0.490882
O 2.706368 1.337474 -0.117904
C 0.824889 2.643860 -0.849825
H -0.118482 2.759703 -0.307571
H 1.480557 3.482338 -0.613840
H 0.597704 2.655058 -1.921475
H 1.315416 -0.640059 -0.341311
O -5.135039 0.824503 1.369751
H -5.771131 0.606998 0.675134
H -4.258648 0.717651 0.944325
O -3.693403 0.863258 -2.625972
H -3.322509 0.742803 -1.724756
H -4.004089 1.778391 -2.642275
O 2.726569 -2.880157 -1.065726
H 1.749866 -2.897025 -0.873967
H 3.041521 -3.762117 -0.821377
O 1.138849 -1.552768 2.207665

H 2.033624 -1.379947 1.840936
H 0.661560 -1.987966 1.463043
O 4.313855 3.613709 -0.019277
H 3.724253 2.829642 -0.053778
H 5.035466 3.352104 0.568395
O 3.620730 -1.125879 0.931215
H 3.410087 -0.241956 0.564389
H 3.375434 -1.750439 0.211638
40
cysteine_pep_anion_6sol_conf_8
Eopt -1351.980212
C 1.603083 0.947419 0.103949
H 1.850340 0.491875 1.070708
H 1.279556 1.973043 0.303613
S 3.085632 0.999751 -0.980352
C 0.376233 0.220396 -0.484535
H 0.228214 0.557794 -1.515376
C 0.484722 -1.308183 -0.560480
O -0.511786 -2.038582 -0.356032
N -0.814317 0.553283 0.280950
N 1.665845 -1.813413 -0.887710
H 2.435007 -1.154540 -1.045075
C 1.886550 -3.240831 -1.035253
H 1.686571 -3.764962 -0.096393
H 1.241061 -3.655255 -1.815041
H 2.928956 -3.395608 -1.314460
C -1.686301 1.531804 0.012476
O -2.637388 1.758508 0.799470
C -1.517760 2.334748 -1.245982
H -2.248521 3.144026 -1.250341
H -0.510165 2.755765 -1.317919
H -1.684148 1.698005 -2.121803
H -0.976723 0.040919 1.142641
O -1.623354 -2.256028 2.277758
H -2.413487 -1.693705 2.123920
H -1.176494 -2.255600 1.410876
O -3.125057 -1.375229 -1.057879
H -2.189599 -1.548911 -0.811408
H -3.484058 -2.242619 -1.291354
O 4.443034 2.758150 1.326891
H 4.073406 2.230989 0.577126
H 4.573113 2.109399 2.032792
O 4.801329 -0.825684 1.031431
H 5.104189 -0.202026 1.705993
H 4.311655 -0.264195 0.381153
O -5.080166 1.799433 -0.607677
H -4.238990 1.867984 -0.111344
H -5.229504 0.846271 -0.677262
O -3.840154 -0.678605 1.545853
H -3.445173 0.215966 1.517286
H -3.736849 -0.977541 0.615039
40
cysteine_pep_anion_6sol_conf_9
Eopt -1351.979506
C 1.659981 -1.483588 -0.909458
H 1.530130 -1.050464 -1.908438
H 1.391940 -2.541710 -0.976809
S 3.413490 -1.353582 -0.386780
C 0.636365 -0.836333 0.050277
H 0.898528 -1.122730 1.072323
C 0.642041 0.698000 -0.025084
O -0.348523 1.347314 -0.420042
N -0.680830 -1.364226 -0.252275
N 1.762764 1.294433 0.373928
H 2.573234 0.687592 0.510172
C 1.978915 2.723669 0.223527
H 1.808339 3.041085 -0.809575
H 1.310920 3.286474 0.880510
H 3.010956 2.941422 0.499688
C -1.732546 -1.408155 0.576819
O -2.833820 -1.850825 0.173105
C -1.557039 -0.939164 1.992532
H -0.804678 -1.547815 2.505691
H -1.224424 0.103377 2.022876
H -2.508075 -1.028570 2.518390
H -0.866398 -1.616380 -1.218426
O -3.039952 1.399924 -1.224116
H -3.616730 1.074773 -0.500424
H -2.127697 1.265696 -0.900126
O -1.502136 3.106687 1.493246
H -1.069418 2.541870 0.823115
H -0.769010 3.548848 1.942680
O 3.081059 -1.387498 2.806737
H 3.183513 -1.377007 1.822921
H 2.966170 -0.454587 3.043075
O 4.038072 1.204608 -2.184597
H 4.411596 0.855980 -3.005939
H 3.877440 0.403568 -1.623979
O -4.732758 0.141321 0.658159
H -4.175808 -0.661745 0.578200
H -5.498641 -0.034710 0.093763
O -3.381975 -1.084630 -2.502250
H -3.232325 -1.559851 -1.661441
H -3.264992 -0.151026 -2.224955
40
cysteine_pep_anion_6sol_ox_conf_25
Eopt -1351.800134
C 0.362898 -1.410192 -1.524785
H 0.575134 -0.592703 -2.226487
H -0.515990 -1.934458 -1.923396
S 1.733287 -2.570760 -1.572302
C 0.013183 -0.849007 -0.134763
H -0.153052 -1.672550 0.559865
C 1.129542 0.049858 0.385382
O 1.321980 1.163800 -0.164620
N -1.196188 -0.046707 -0.212499
N 1.861391 -0.400224 1.386195
H 1.660889 -1.328770 1.772053
C 2.992578 0.343949 1.916087
H 3.731752 0.528726 1.131788

H 2.662443 1.299483 2.331763
H 3.448929 -0.251741 2.706466
C -2.452289 -0.482412 -0.015897
O -3.412993 0.297447 -0.193291
C -2.665900 -1.898187 0.435489
H -3.736007 -2.102706 0.475546
H -2.186925 -2.606909 -0.247315
H -2.236714 -2.045150 1.432538
H -1.092823 0.900230 -0.567022
O 3.143356 3.319135 0.222679
H 3.833486 3.057300 0.847365
H 2.567399 2.532520 0.154598
O -0.317212 3.334804 0.746381
H 0.244806 2.599107 0.436972
H 0.085440 4.125421 0.360031
O 1.302783 -3.026938 2.505589
H 0.787918 -3.451190 1.803778
H 2.162882 -3.469601 2.471672
O 3.259608 0.697801 -2.260925
H 2.602113 0.900414 -1.570216
H 2.731681 0.409588 -3.018439
O -6.089221 -0.423862 0.130024
H -5.137968 -0.204408 0.030026
H -6.546295 0.420079 0.013315
O -2.848311 3.060083 -0.477848
H -3.076141 2.111519 -0.401967
H -1.961965 3.134259 -0.064968
40
cysteine_pep_anion_6sol_ox_conf_7
Eopt -1351.800749
C -0.983514 -1.035918 -1.805859
H -0.446558 -0.943562 -2.759351
H -2.049842 -1.035915 -2.056541
S -0.556852 -2.648619 -1.132279
C -0.666332 0.185857 -0.931178
H -0.959664 1.054824 -1.525513
C -1.571544 0.211335 0.301707
O -2.787321 0.455676 0.132011
N 0.748949 0.280282 -0.640384
N -1.046432 -0.035437 1.491555
H -0.071082 -0.334915 1.573772
C -1.862485 -0.074364 2.694139
H -2.624627 -0.855847 2.623843
H -1.208998 -0.286856 3.540184
H -2.353364 0.888861 2.855272
C 1.439730 1.426777 -0.481707
O 2.648269 1.388505 -0.171672
C 0.736731 2.733222 -0.707931
H -0.159912 2.813808 -0.085344
H 1.417061 3.549841 -0.466004
H 0.430797 2.822166 -1.755919
H 1.251165 -0.574340 -0.411377
O -5.117902 0.571975 1.680337
H -5.775955 0.659187 0.977461
H -4.261934 0.511549 1.208172
O -3.873535 1.183280 -2.332444
H -3.459162 0.922762 -1.482012
H -4.109606 2.113271 -2.214878
O 3.134260 -3.082253 -0.732628
H 2.431080 -3.437454 -0.169119
H 3.849112 -3.731556 -0.662147
O 1.386249 -1.430146 2.208967
H 2.249782 -1.217836 1.784920
H 1.105924 -2.250793 1.778925
O 4.260268 3.666043 0.024976
H 3.672370 2.883536 -0.036658
H 4.976621 3.389042 0.612041
O 3.779399 -0.906234 0.903655
H 3.452542 -0.099990 0.450405
H 3.643247 -1.632012 0.257492
43
cysteine_pep_anion_7sol_conf_1
Eopt -1428.407622
C 1.339379 -1.574352 -0.374656
H 1.103911 -1.385979 -1.428366
H 0.877015 -2.527985 -0.104095
S 3.153279 -1.709458 -0.144478
C 0.656075 -0.494221 0.494233
H 1.077266 -0.553305 1.500723
C 0.907688 0.913680 -0.057550
O 0.017416 1.567482 -0.642835
N -0.766675 -0.773937 0.568734
N 2.131842 1.403540 0.121317
H 2.845847 0.743472 0.422289
C 2.554137 2.658256 -0.479234
H 2.394476 2.652504 -1.561267
H 1.997419 3.493098 -0.045910
H 3.615730 2.794683 -0.272822
C -1.598906 -0.341999 1.522743
O -2.823139 -0.615826 1.479177
C -1.034746 0.451421 2.667978
H -0.421639 1.289024 2.322341
H -1.855918 0.832899 3.275449
H -0.400803 -0.192659 3.288080
H -1.182556 -1.259719 -0.228724
O -2.102382 3.011090 0.498622
H -2.143095 3.782287 -0.084368
H -1.345652 2.489122 0.161563
O -2.044534 0.668020 -2.329281
H -1.283374 0.909509 -1.761751
H -2.817067 0.813619 -1.739182
O 4.049379 0.261227 -2.474235
H 3.784121 -0.360323 -1.748410
H 3.939420 -0.251994 -3.286370
O 3.465815 -0.746096 2.917469
H 4.007526 0.053769 2.864025
H 3.370095 -1.041435 1.978658
O -4.010919 1.189932 -0.398075
H -3.773696 0.455174 0.197658
H -3.445714 1.923396 -0.066849

O -3.981812 -2.507585 -0.197233
H -3.563278 -1.866903 0.420080
H -4.735608 -2.028709 -0.569715
H -2.667071 -2.293802 -1.454827
H -1.956124 -1.133760 -2.207743
O -1.866275 -2.086816 -1.982390
43
cysteine_pep_anion_7sol_conf_10
Eopt -1428.407893
C 0.901898 -0.123923 1.600366
H 0.286360 -0.788317 2.217369
H 0.702463 0.900103 1.934931
S 2.678401 -0.517649 1.817455
C 0.401551 -0.215918 0.147765
H 1.041044 0.381264 -0.505217
C 0.365674 -1.662041 -0.338729
O -0.521483 -2.439475 0.088235
N -0.969386 0.269536 0.040688
N 1.271112 -2.040579 -1.228460
H 2.039638 -1.399476 -1.442298
C 1.354890 -3.414006 -1.695241
H 0.427315 -3.705361 -2.194890
H 2.179171 -3.482610 -2.405238
H 1.538804 -4.100559 -0.863029
C -1.384488 1.528486 -0.144647
O -2.610562 1.788470 -0.087334
C -0.366374 2.586650 -0.452858
H 0.085250 2.389651 -1.430898
H -0.851664 3.562889 -0.474124
H 0.435060 2.595606 0.291554
H -1.689338 -0.401335 0.283422
O -2.968869 -2.371702 -1.374245
H -3.424039 -3.204022 -1.182525
H -2.114686 -2.445634 -0.903293
O -2.522298 -2.165682 2.147812
H -1.770586 -2.275947 1.533829
H -3.136704 -1.577956 1.661521
O 3.625919 -0.310129 -1.252110
H 3.424898 -0.363376 -0.284074
H 3.385803 0.612159 -1.486519
O 3.153080 2.530408 1.145103
H 4.079488 2.755141 1.313683
H 3.085640 1.564066 1.379469
O -4.190306 -0.579261 0.420173
H -3.755978 0.285322 0.272004
H -3.846469 -1.155326 -0.297723
O -3.640769 4.317785 -0.623652
H -3.247574 3.437095 -0.437720
H -4.595531 4.166965 -0.612884
H 3.012752 2.523525 -0.640553
H 3.775015 2.814194 -1.955509
O 2.966345 2.404103 -1.618494
43
cysteine_pep_anion_7sol_conf_13
Eopt -1428.411852
C 0.272417 -1.218433 -1.284942
H 1.202610 -1.700827 -1.595862
H 0.080153 -0.403190 -1.994416
S -1.094270 -2.434239 -1.338645
C 0.522496 -0.596817 0.099311
H 0.713125 -1.399074 0.817473
C 1.797635 0.245094 0.028318
O 2.903286 -0.343263 0.004009
N -0.629991 0.172072 0.523866
N 1.692046 1.565762 -0.049381
H 0.773255 2.010396 -0.007967
C 2.858068 2.425744 -0.160407
H 3.521071 2.293633 0.699271
H 3.415284 2.207692 -1.075607
H 2.514600 3.459893 -0.189731
C -0.911557 0.570585 1.774723
O -1.841406 1.385272 1.975996
C -0.093538 0.018088 2.905803
H -0.462021 0.429108 3.846022
H -0.167935 -1.074097 2.931871
H 0.964014 0.278462 2.790468
H -1.229709 0.546837 -0.205665
O 3.111258 -3.107319 0.261210
H 3.867765 -3.220527 0.852242
H 3.005056 -2.134798 0.176738
O 5.616154 0.255967 -0.244542
H 4.646056 0.137076 -0.172238
H 5.909425 -0.480096 -0.798580
O -3.101405 -0.055006 -1.935599
H -2.512781 -0.842792 -1.790130
H -3.479951 0.122299 -1.044883
O -2.810046 -2.133484 1.322390
H -3.425495 -2.876703 1.247743
H -2.227750 -2.205711 0.519162
O -0.810231 3.265458 -0.037217
H -1.326321 2.803689 0.647823
H -1.113243 2.869088 -0.885883
O -4.073956 0.256604 0.669487
H -3.414328 0.863438 1.059122
H -3.724225 -0.627606 0.927072
H -2.070059 1.320411 -2.283239
H -2.061365 2.724049 -2.943092
O -1.493208 2.102867 -2.466686
43
cysteine_pep_anion_7sol_conf_17
Eopt -1428.408208
C -1.477765 -0.991722 0.460911
H -2.166911 -0.230537 0.839499
H -1.028167 -1.467000 1.340709
S -2.416974 -2.207820 -0.539250
C -0.365772 -0.279182 -0.333725
H -0.709126 -0.149222 -1.362176
C -0.115237 1.113526 0.250227
O 0.816205 1.352296 1.048487
N 0.842310 -1.079528 -0.348196

N -0.973429 2.047597 -0.148769
H -1.751015 1.753084 -0.744201
C -0.954551 3.398540 0.383793
H 0.003419 3.881553 0.172724
H -1.751885 3.965970 -0.096238
H -1.118329 3.395903 1.465816
C 1.858722 -0.961832 -1.214109
O 2.901650 -1.642037 -1.063802
C 1.713440 -0.019134 -2.374006
H 2.645324 -0.005397 -2.939939
H 0.898718 -0.349924 -3.027774
H 1.481472 0.996367 -2.038596
H 1.013758 -1.667497 0.461750
O 3.061523 2.634556 -0.217993
H 3.679660 1.879776 -0.247825
H 2.267675 2.270916 0.216392
O 3.044038 0.072025 2.156995
H 2.207586 0.391520 1.759416
H 3.708411 0.221615 1.452878
O -4.902214 -1.436332 1.223875
H -4.165456 -1.754705 0.631178
H -5.712196 -1.754756 0.800166
O -3.305741 0.794205 -1.607671
H -3.863304 1.024683 -0.831919
H -3.093795 -0.156162 -1.462872
O 4.699378 0.306979 -0.128020
H 4.263310 -0.452503 -0.564286
H 5.630254 0.063548 -0.028111
O 3.190986 -2.668175 1.557425
H 3.141576 -2.503774 0.594252
H 3.095014 -1.761237 1.919750
H -5.695175 1.532960 0.404163
H -4.890515 0.321800 0.935433
O -4.812564 1.279045 0.707955
43
cysteine_pep_anion_7sol_conf_20
Eopt -1428.407503
C 1.280795 -0.055878 -1.349290
H 2.159261 0.558767 -1.131505
H 0.711367 0.472539 -2.123968
S 1.819717 -1.697641 -1.961392
C 0.427688 -0.103003 -0.070783
H 0.922103 -0.746928 0.658398
C 0.334934 1.306233 0.521341
O -0.621974 2.065587 0.254838
N -0.886365 -0.640991 -0.361254
N 1.345775 1.661031 1.306180
H 2.114386 0.992992 1.434696
C 1.466622 2.997144 1.862326
H 2.332173 3.013803 2.525034
H 1.607776 3.741484 1.072712
H 0.573332 3.255938 2.437142
C -1.769405 -1.102772 0.533467
O -2.930727 -1.407427 0.171145
C -1.331030 -1.262316 1.960846
H -2.149087 -1.683212 2.545838
H -0.461365 -1.923825 2.027934
H -1.049357 -0.294374 2.389005
H -1.238580 -0.496882 -1.302782
O -0.983576 4.824846 0.083283
H -1.724441 4.827488 -0.538392
H -0.778900 3.874659 0.207743
O -2.227228 2.046011 -2.057332
H -1.677478 1.963710 -1.252053
H -2.762342 1.225315 -2.087452
O 4.644024 -0.590910 -1.047249
H 3.834553 -1.045420 -1.401987
H 5.265722 -1.309781 -0.864651
O 2.408865 -2.851175 0.947579
H 1.541950 -2.973955 1.360726
H 2.202397 -2.556738 0.019589
O -3.805753 -0.303262 -2.224301
H -3.535362 -0.751875 -1.392251
H -3.352693 -0.794673 -2.923799
O -4.731240 -2.692138 1.865813
H -4.079443 -2.231591 1.293917
H -4.329683 -2.679707 2.745370
H 3.950713 -0.252403 0.654575
H 3.061724 -1.172867 1.488571
O 3.506647 -0.299898 1.528467
43
cysteine_pep_anion_7sol_conf_21
Eopt -1428.404675
C -1.389776 -0.335888 1.623193
H -1.303240 0.565792 2.239852
H -0.795923 -1.117046 2.109252
S -3.140621 -0.869393 1.514605
C -0.733121 -0.067692 0.259992
H -0.935307 -0.911613 -0.401094
C -1.260376 1.224138 -0.366558
O -0.915202 2.337831 0.093455
N 0.710384 0.035374 0.438154
N -2.073914 1.113619 -1.407992
H -2.398210 0.182072 -1.673521
C -2.683301 2.272889 -2.036503
H -1.915082 2.942072 -2.433258
H -3.311121 1.924869 -2.857059
H -3.299670 2.827070 -1.321905
C 1.655280 -0.300533 -0.448610
O 2.863855 -0.080891 -0.188607
C 1.245887 -0.947322 -1.740184
H 0.671456 -1.861950 -1.562955
H 0.617396 -0.262638 -2.319136
H 2.138786 -1.188044 -2.317610
H 1.033778 0.522118 1.269662
O 0.698225 2.811819 2.360163
H 1.593033 2.709513 1.983786
H 0.098676 2.636709 1.605531
O 1.532261 3.242914 -1.081747
H 0.666193 2.959135 -0.734105

H 2.159509 3.004861 -0.369127
O -1.626900 -3.620320 0.830547
H -2.192116 -2.835245 1.037263
H -0.837548 -3.240630 0.418568
O -3.288945 -1.567419 -1.612040
H -4.173768 -1.331675 -1.924929
H -3.318549 -1.413470 -0.632172
O 3.189563 2.367667 1.051393
H 3.220172 1.450724 0.699829
H 4.099178 2.695493 1.030296
O 4.430053 -2.278070 -0.839190
H 3.969215 -1.427832 -0.667263
H 5.076147 -2.357653 -0.123276
H 3.025125 -3.311785 -0.239581
H 1.557945 -3.229199 0.227962
O 2.284718 -3.855763 0.106299
43
cysteine_pep_anion_7sol_conf_26
Eopt -1428.417687
C -1.178457 0.220409 -1.789998
H -0.793832 0.747187 -2.668493
H -2.267420 0.198234 -1.879212
S -0.536023 -1.496498 -1.773399
C -0.833509 1.079798 -0.563057
H -1.288831 2.061246 -0.727017
C -1.487528 0.521494 0.700688
O -2.739016 0.458517 0.755108
N 0.600695 1.249111 -0.442245
N -0.721683 0.121837 1.706375
H 0.296290 0.178062 1.645745
C -1.289776 -0.450817 2.914419
H -1.877846 0.294703 3.458566
H -1.934234 -1.300387 2.673198
H -0.469970 -0.790738 3.547765
C 1.240846 2.317085 0.062425
O 2.482749 2.286854 0.218208
C 0.443049 3.533274 0.433238
H -0.029867 3.964413 -0.455722
H -0.348101 3.282528 1.146947
H 1.110563 4.272029 0.877526
H 1.170910 0.427046 -0.631192
O -3.434954 -2.101581 -0.405329
H -2.564796 -2.103880 -0.863604
H -3.401878 -1.268117 0.098703
O -4.287612 1.936120 -1.021131
H -3.723787 1.392257 -0.428214
H -3.763623 2.026244 -1.828933
O 2.683512 -1.701709 -1.726845
H 1.715446 -1.531303 -1.810063
H 3.106451 -0.817308 -1.648754
O -0.219389 -3.030963 1.046499
H -0.258006 -3.946197 0.734025
H -0.426873 -2.486524 0.247845
O 2.242602 0.086772 2.149410
H 2.573952 0.720658 1.490329
H 2.334079 -0.799127 1.728243
O 4.075629 0.683875 -1.378293
H 3.506065 1.217797 -0.780745
H 4.792414 0.362879 -0.813055
H 2.629189 -2.170716 0.017539
H 1.542682 -2.622077 1.024668
O 2.499800 -2.404015 0.965260
43
cysteine_pep_anion_7sol_conf_27
Eopt -1428.412272
C 0.531227 0.781193 1.392263
H 1.523493 1.147044 1.671762
H 0.361265 -0.151553 1.943347
S -0.731758 2.023815 1.856793
C 0.578331 0.449903 -0.112052
H 0.803650 1.362816 -0.672002
C 1.696700 -0.569836 -0.336560
O 1.481094 -1.798926 -0.245919
N -0.704878 -0.077087 -0.533934
N 2.898077 -0.065594 -0.588131
H 3.008150 0.953892 -0.583222
C 4.079958 -0.900279 -0.715060
H 3.921733 -1.674320 -1.470452
H 4.912614 -0.267532 -1.023150
H 4.329546 -1.380085 0.236103
C -1.065501 -0.467206 -1.760859
O -2.199660 -0.972785 -1.954191
C -0.097420 -0.295591 -2.894196
H -0.648660 -0.303624 -3.835695
H 0.475901 0.631714 -2.813307
H 0.607505 -1.135089 -2.898492
H -1.392491 -0.222480 0.197724
O 2.835491 -3.451201 1.575148
H 2.065265 -3.840172 2.012141
H 2.450404 -2.851901 0.903951
O -0.607672 -2.948949 1.268402
H 0.027151 -2.468851 0.699283
H -1.494532 -2.639806 0.983506
O -3.523122 0.441387 1.540849
H -2.663070 0.915301 1.666711
H -3.810568 0.695296 0.636615
O 0.682205 3.993932 -0.153918
H 0.844708 4.828174 0.309553
H 0.177129 3.435348 0.498612
O -3.184989 -2.145673 0.466688
H -2.939906 -1.844798 -0.432139
H -3.300632 -1.304404 0.961164
O -4.235781 0.827440 -1.151986
H -3.578430 0.177776 -1.475736
H -5.083796 0.364474 -1.204561
H 3.535918 2.821876 0.572625
H 2.295693 3.208760 -0.261239
O 3.200925 2.826121 -0.334890
43

cysteine_pep_anion_7sol_conf_30
Eopt -1428.403704

C	0.508725	-1.068885	1.370565
H	-0.177023	-1.071500	2.223889
H	1.510047	-1.270063	1.760474
S	0.024693	-2.368365	0.170196
C	0.509004	0.364006	0.803170
H	0.749644	1.042292	1.627090
C	1.623633	0.540135	-0.224086
O	2.813044	0.400561	0.147589
N	-0.805933	0.687164	0.285530
N	1.307653	0.825209	-1.478527
H	0.328757	0.915100	-1.717895
C	2.306174	0.982924	-2.522670
H	2.980300	1.810727	-2.287945
H	2.891405	0.066187	-2.635704
H	1.789246	1.195963	-3.458005
C	-1.410751	1.884600	0.290586
O	-2.556822	2.008599	-0.198021
C	-0.694706	3.053314	0.903347
H	-1.259399	3.965398	0.707874
H	-0.611885	2.909979	1.986407
H	0.316640	3.158957	0.500066
H	-1.308709	-0.089144	-0.142472
O	5.386895	0.496093	-0.958500
H	5.408869	-0.269370	-1.549294
H	4.460308	0.527023	-0.644354
O	3.629120	0.240485	2.801799
H	3.304633	0.269396	1.876067
H	4.435785	0.773184	2.794567
O	-3.107946	-2.152734	0.697276
H	-2.138745	-2.242547	0.512569
H	-3.416389	-1.455155	0.076670
O	3.127685	-2.449737	-0.675588
H	3.325833	-1.531466	-0.425647
H	2.158991	-2.523244	-0.494626
O	-4.038474	-0.175371	-1.062704
H	-3.531277	0.611467	-0.761558
H	-4.943151	-0.020201	-0.756810
O	-3.988054	4.395838	-0.048377
H	-3.458521	3.570622	-0.093988
H	-3.428033	5.012987	0.441967
H	-3.882662	-5.185139	0.194214
H	-3.747096	-3.660116	-0.019049
O	-4.105096	-4.481317	-0.429991

43
cysteine_pep_anion_7sol_conf_5
Eopt -1428.411335

C	1.946371	0.572584	-1.666701
H	1.829379	1.637643	-1.434431
H	1.769164	0.450485	-2.739792
S	3.642429	0.017644	-1.250838
C	0.846951	-0.213078	-0.941881
H	0.971922	-1.274290	-1.158885
C	0.890029	0.018758	0.569815
O	0.561951	1.129118	1.049586
N	-0.461732	0.202894	-1.437037
N	1.245254	-1.003811	1.337102
H	1.617068	-1.838098	0.882138
C	1.351186	-0.884694	2.781083
H	0.382079	-0.623235	3.214788
H	1.671636	-1.847205	3.180145
H	2.081411	-0.118670	3.059421
C	-1.558019	-0.561864	-1.527452
O	-2.658597	-0.041868	-1.833366
C	-1.437869	-2.041231	-1.298923
H	-2.427095	-2.494721	-1.366538
H	-0.786950	-2.486179	-2.059463
H	-1.005136	-2.258422	-0.318534
H	-0.609953	1.205551	-1.533067
O	-2.091532	0.989036	2.058395
H	-2.333901	0.041493	1.950695
H	-1.154621	1.041118	1.786320
O	-0.516780	3.261833	-0.453072
H	-0.062107	2.582095	0.085882
H	-1.462915	3.008212	-0.396528
O	2.886378	-2.922726	-0.251727
H	3.200931	-2.045075	-0.594655
H	3.544365	-3.175992	0.410829
O	3.920197	1.730583	1.406026
H	4.739626	2.232425	1.296789
H	3.851673	1.180905	0.580689
O	-4.737979	-1.017648	-0.284964
H	-4.053844	-0.700354	-0.915427
H	-5.137643	-0.208298	0.064355
O	-3.135873	2.219247	-0.200083
H	-3.027947	1.523487	-0.879092
H	-2.853871	1.770853	0.630522
H	-3.612719	-1.477776	1.044726
H	-3.448111	-1.804394	2.549098
O	-2.937499	-1.640112	1.743870

43
cysteine_pep_anion_7sol_conf_6
Eopt -1428.410266

C	1.523810	-0.565656	-1.514476
H	1.081724	-0.009374	-2.349525
H	1.345579	-1.627225	-1.712720
S	3.334964	-0.264226	-1.433390
C	0.744153	-0.238629	-0.232225
H	1.201313	-0.768227	0.610096
C	0.690343	1.232125	0.201015
O	-0.039066	1.566626	1.163060
N	-0.637168	-0.683393	-0.366027
N	1.413413	2.120570	-0.467203
H	2.130871	1.756885	-1.094923
C	1.485632	3.511550	-0.053835
H	2.142567	4.039003	-0.745505
H	0.493634	3.968845	-0.083628
H	1.885884	3.597664	0.961513
C	-1.149288	-1.839016	0.068111

O -2.348985 -2.128305 -0.168405
C -0.271784 -2.783526 0.839179
H 0.097907 -2.316176 1.757462
H -0.849400 -3.671330 1.098570
H 0.593180 -3.078944 0.236462
H -1.244003 -0.093985 -0.942875
O -1.380352 -0.066870 2.943402
H -1.533977 0.550196 3.672473
H -0.868566 0.452021 2.281906
O -2.670446 2.295769 0.379973
H -1.747180 2.175744 0.675930
H -3.117605 1.480464 0.701172
O 3.442413 -2.831067 0.475525
H 3.479130 -2.037490 -0.114493
H 2.757562 -2.609769 1.123089
O 3.656440 0.537153 1.693404
H 2.791750 0.339488 2.080376
H 3.549029 0.304014 0.739125
O -3.696461 -0.103637 1.405014
H -3.443938 -0.773021 0.742621
H -2.945215 -0.132875 2.040774
O -3.821639 -0.959860 -2.218246
H -3.300345 -1.385897 -1.501140
H -4.628958 -0.652381 -1.782487
H -2.780514 0.555579 -2.277286
H -2.364544 1.715516 -1.333163
O -2.098932 1.251137 -2.156675
43
cysteine_pep_anion_7sol_conf_7
Eopt -1428.408251
C -1.472293 0.554376 -1.483390
H -1.409385 -0.344010 -2.108598
H -1.058083 1.382676 -2.065618
S -3.219695 0.926855 -1.066400
C -0.546511 0.375782 -0.254938
H -0.788422 1.148604 0.477352
C -0.736765 -0.983362 0.418847
O 0.161820 -1.852975 0.419035
N 0.853362 0.508214 -0.612322
N -1.914955 -1.183529 1.001582
H -2.648445 -0.506206 0.782231
C -2.289849 -2.476296 1.549632
H -2.230413 -3.260954 0.788382
H -1.633168 -2.743425 2.381413
H -3.314209 -2.405629 1.916204
C 1.569174 1.638388 -0.625088
O 2.784136 1.607615 -0.943362
C 0.904139 2.927477 -0.236307
H -0.043311 3.073089 -0.762928
H 0.696569 2.921070 0.840212
H 1.576628 3.756124 -0.460403
H 1.352334 -0.341546 -0.856260
O 2.098603 -1.169111 2.393203
H 2.841744 -0.864734 1.831735
H 1.440951 -1.495746 1.749476
O 2.663650 -2.417605 -0.887144
H 1.774263 -2.334948 -0.495857
H 3.227122 -1.849517 -0.320907
O -2.605533 2.952544 1.368055
H -2.803798 2.342647 0.615116
H -1.678611 3.199155 1.236556
O -4.274583 -2.050848 -1.486374
H -4.113110 -2.509895 -0.650173
H -3.970562 -1.122766 -1.320996
O 4.033555 -0.420331 0.527165
H 3.709860 0.333050 -0.012272
H 4.993169 -0.317255 0.588843
O 3.193673 -0.469841 -2.912413
H 3.133799 0.304429 -2.320269
H 2.958641 -1.217331 -2.325593
H 0.041170 1.247510 2.602675
H 1.271470 0.357251 2.877423
O 0.826267 1.186514 3.163999
43
cysteine_pep_anion_7sol_conf_8
Eopt -1428.412514
C -1.476894 -0.203075 1.404483
H -2.154140 0.656719 1.419968
H -0.866885 -0.140327 2.313287
S -2.453223 -1.754114 1.398953
C -0.553009 -0.036211 0.186166
H -1.124200 -0.259626 -0.715047
C -0.084716 1.418579 0.107005
O 0.955564 1.811576 0.684890
N 0.569999 -0.949475 0.263402
N -0.875301 2.236920 -0.575380
H -1.741473 1.855335 -0.973457
C -0.626569 3.665571 -0.656562
H -0.630242 4.120580 0.338487
H 0.338740 3.862982 -1.130898
H -1.416536 4.115911 -1.257947
C 1.298939 -1.405725 -0.764153
O 2.317243 -2.106198 -0.553008
C 0.867468 -1.064296 -2.162247
H 0.912378 0.018649 -2.321833
H 1.532028 -1.557660 -2.871936
H -0.161586 -1.390107 -2.344621
H 0.936472 -1.147950 1.189693
O 1.975851 0.474180 2.979195
H 2.659076 -0.078321 2.543619
H 1.567203 0.952892 2.230304
O 3.558526 1.380277 -0.221326
H 2.624395 1.510118 0.049009
H 4.030322 2.159297 0.104743
O -4.834161 0.217439 0.715350
H -4.173046 -0.485381 0.956326
H -5.511874 -0.256190 0.212030
O -3.035598 -1.744933 -1.741317
H -3.980687 -1.937768 -1.825116
H -2.857693 -1.806493 -0.764796

O 3.919041 -0.874423 1.429099
H 3.381578 -1.475424 0.874780
H 3.959452 -0.062978 0.879579
O 4.338150 -0.682864 -2.014390
H 3.723711 -1.343228 -1.641693
H 4.136805 0.113996 -1.487674
H -3.876075 0.946860 -0.721958
H -3.141956 0.134526 -1.779526
O -3.360666 1.062225 -1.548852
43
cysteine_pep_anion_7sol_conf_9
Eopt -1428.406984
C -0.867643 0.605282 1.557937
H -0.858307 1.545554 0.995221
H -0.252489 0.755290 2.450394
S -2.573879 0.196319 2.078921
C -0.166968 -0.479243 0.718087
H -0.288525 -1.442336 1.220533
C -0.769699 -0.548929 -0.686091
O -0.472910 0.308668 -1.550725
N 1.255338 -0.157691 0.646322
N -1.609266 -1.544519 -0.935579
H -1.864066 -2.168331 -0.167960
C -2.311701 -1.672415 -2.200474
H -1.600603 -1.728462 -3.029171
H -2.898602 -2.590803 -2.172134
H -2.982007 -0.823893 -2.366488
C 2.222462 -0.946988 0.165081
O 3.405772 -0.533933 0.100335
C 1.877292 -2.326595 -0.315223
H 2.784180 -2.932131 -0.345080
H 1.131873 -2.816937 0.315356
H 1.472956 -2.258380 -1.332090
H 1.523440 0.798149 0.898037
O -2.699486 1.975244 -2.232163
H -3.335479 1.635174 -1.564355
H -1.918755 1.402451 -2.113028
O 0.475283 2.879658 -1.142759
H 0.205064 1.937166 -1.216924
H -0.350848 3.347075 -0.953827
O -2.569329 -2.927501 1.499591
H -2.624419 -1.973993 1.775300
H -3.472555 -3.159166 1.240520
O -4.458874 0.951943 -0.317822
H -4.948796 1.725948 -0.004842
H -3.835869 0.728902 0.429416
O 5.558807 -2.025676 -0.824273
H 4.770269 -1.536595 -0.501993
H 6.303909 -1.443967 -0.621435
O 4.427787 1.988423 0.544803
H 4.063187 1.090521 0.373154
H 4.563095 2.372839 -0.332476
H 1.372141 2.889876 0.426640
H 2.775846 2.620822 1.041543
O 1.835759 2.759789 1.283242
43
cysteine_pep_anion_7sol_ox_conf_17
Eopt -1428.229663
C 1.558113 -0.886098 -0.306282
H 2.417418 -0.221126 -0.452386
H 1.253741 -1.210547 -1.312107
S 2.140657 -2.341688 0.572536
C 0.417142 -0.126400 0.382983
H 0.732620 0.132772 1.396012
C 0.145574 1.172992 -0.392333
O -0.776084 1.247091 -1.231894
N -0.767528 -0.951462 0.457162
N 0.953089 2.188441 -0.119546
H 1.713186 2.044903 0.554505
C 0.857805 3.456626 -0.822217
H -0.122835 3.913262 -0.662233
H 1.628542 4.120894 -0.431257
H 1.012882 3.319419 -1.896373
C -1.771604 -0.808982 1.340894
O -2.796069 -1.519988 1.246073
C -1.626104 0.199682 2.443449
H -2.545014 0.216945 3.029931
H -0.787607 -0.069086 3.095494
H -1.434271 1.200645 2.043726
H -0.958960 -1.558145 -0.335189
O -3.054907 2.569639 -0.038568
H -3.667995 1.815240 0.048903
H -2.268213 2.185885 -0.467481
O -2.975358 -0.224074 -2.166666
H -2.151613 0.169470 -1.813173
H -3.647855 -0.009714 -1.487258
O 4.835186 -1.732091 -1.403026
H 4.136442 -2.180662 -0.894809
H 5.658358 -2.132141 -1.086878
O 3.166361 1.596308 1.675896
H 3.768597 1.253968 0.975781
H 2.846038 0.809334 2.138270
O -4.659508 0.224271 0.062097
H -4.207152 -0.458793 0.595626
H -5.584871 -0.050458 -0.001848
O -3.053962 -2.876740 -1.241334
H -3.014053 -2.602906 -0.304020
H -2.978448 -2.014951 -1.704709
H 5.777830 0.965664 -0.015912
H 4.848557 -0.058625 -0.708339
O 4.881802 0.863081 -0.365970
43
cysteine_pep_anion_7sol_ox_conf_21
Eopt -1428.226774
C 1.306235 0.693713 1.800489
H 1.394889 -0.224711 2.398061
H 0.586511 1.333623 2.325049
S 2.908071 1.504281 1.837956
C 0.774368 0.373049 0.400202
H 0.660484 1.301662 -0.159220

C 1.712589 -0.574670 -0.350991
O 1.953039 -1.716390 0.103952
N -0.533728 -0.247869 0.539301
N 2.224191 -0.136461 -1.491397
H 1.984603 0.805157 -1.815268
C 3.118821 -0.951586 -2.295622
H 2.620518 -1.870686 -2.616698
H 3.407351 -0.374792 -3.174306
H 4.015646 -1.215407 -1.727863
C -1.569339 -0.135620 -0.309766
O -2.581298 -0.854120 -0.147139
C -1.492617 0.865437 -1.424787
H -1.323911 1.875204 -1.035023
H -0.664106 0.618679 -2.096537
H -2.426165 0.848246 -1.987200
H -0.612713 -0.986991 1.232989
O 0.560233 -3.062720 2.165182
H -0.287573 -3.258091 1.722655
H 1.077409 -2.574737 1.492488
O 0.056901 -3.359418 -1.360273
H 0.788480 -2.880400 -0.931447
H -0.630768 -3.411748 -0.664417
O -0.096387 3.857414 0.741463
H 0.571413 3.619092 0.082693
H -0.578828 3.032938 0.897687
O 1.600127 2.582855 -2.362956
H 2.066720 3.121821 -1.708150
H 0.663749 2.688804 -2.140041
O -1.872822 -3.398929 0.712644
H -2.276810 -2.533651 0.484069
H -2.550871 -4.066843 0.540414
O -4.945796 0.473246 -0.731623
H -4.159943 -0.099620 -0.593639
H -5.619689 0.119374 -0.134439
H -4.212098 1.869959 0.214514
H -2.877416 2.344964 0.822264
O -3.808947 2.592819 0.742699
43
cysteine_pep_anion_7sol_ox_conf_26
Eopt -1428.228920
C -1.363733 0.600993 -1.743002
H -1.139025 1.305995 -2.555641
H -2.451992 0.497983 -1.719181
S -0.652516 -0.968343 -2.260686
C -0.866626 1.201335 -0.418831
H -1.341974 2.183473 -0.352118
C -1.406288 0.406392 0.774582
O -2.647294 0.266858 0.885682
N 0.568553 1.380144 -0.413608
N -0.555927 -0.109308 1.646808
H 0.451865 0.052779 1.568298
C -1.011762 -0.879897 2.792438
H -1.507092 -0.234122 3.524142
H -1.713424 -1.656699 2.479151
H -0.141416 -1.345715 3.255054
C 1.241196 2.397045 0.166906
O 2.486555 2.367594 0.216109
C 0.464419 3.545854 0.740413
H -0.106399 4.053993 -0.043912
H -0.241580 3.198224 1.501713
H 1.161972 4.252267 1.190804
H 1.143287 0.599050 -0.720976
O -3.568440 -2.114878 -0.341655
H -3.826573 -1.818786 -1.225505
H -3.255475 -1.303526 0.102988
O -4.375926 2.022819 -0.445690
H -3.772666 1.391404 0.000455
H -3.854554 2.365139 -1.185135
O 2.803774 -1.642425 -1.714158
H 1.885164 -1.374732 -1.874363
H 3.283262 -0.798346 -1.538456
O -0.228283 -3.612912 0.710911
H -0.229761 -4.045679 -0.154950
H -0.886121 -2.907496 0.627098
O 2.320776 -0.067673 2.009264
H 2.703657 0.557594 1.373415
H 2.400381 -0.957004 1.590013
O 4.199436 0.669917 -1.159883
H 3.559418 1.257384 -0.701246
H 4.812919 0.390270 -0.465657
H 2.567716 -2.305024 -0.064661
H 1.464417 -2.901085 0.852028
O 2.389732 -2.575553 0.864445
43
cysteine_pep_anion_7sol_ox_conf_30
Eopt -1428.221504
C -0.484105 1.505110 0.083213
H 0.323221 2.068893 0.567521
H -1.409608 1.805383 0.584909
S -0.552799 2.061168 -1.626186
C -0.236292 0.005204 0.302866
H -0.182409 -0.136596 1.385935
C -1.439528 -0.813975 -0.163317
O -2.550104 -0.599722 0.375770
N 1.017783 -0.405965 -0.290808
N -1.272032 -1.711010 -1.120269
H -0.341159 -1.849803 -1.493499
C -2.361429 -2.532956 -1.624104
H -2.731702 -3.201026 -0.842063
H -3.180206 -1.901282 -1.976757
H -1.981771 -3.127139 -2.454472
C 1.898524 -1.287453 0.223676
O 2.973403 -1.500877 -0.374337
C 1.561525 -1.973969 1.514346
H 2.297925 -2.753953 1.708484
H 1.591170 -1.246611 2.334052
H 0.560306 -2.414210 1.488098
H 1.292167 0.031395 -1.167097
O -5.041854 -1.907749 0.454719
H -5.084174 -2.508304 -0.302209

H -4.158870 -1.493045 0.382529
O -2.757428 0.791145 2.811344
H -2.661790 0.309169 1.964283
H -2.753949 1.724168 2.556960
O 3.069801 2.011150 1.096489
H 2.399052 1.400933 1.434873
H 3.337079 1.633631 0.227383
O -4.059442 1.666647 -0.501318
H -3.554789 0.885628 -0.207182
H -3.721630 2.387515 0.048353
O 3.911797 0.950835 -1.347917
H 3.699915 0.017166 -1.138197
H 4.876548 1.006687 -1.298785
O 4.746537 -3.507309 0.436389
H 4.109812 -2.806607 0.181117
H 4.309088 -3.978186 1.158840
H 0.886420 4.351259 0.723479
H 2.211228 3.556982 0.684556
O 1.814852 4.429170 0.464526
43
cysteine_pep_anion_7sol_ox_conf_6
Eopt -1428.231989
C 1.569840 0.173928 -1.980638
H 1.112433 0.906302 -2.658037
H 1.567982 -0.771632 -2.539688
S 3.293205 0.615151 -1.713945
C 0.759910 -0.025889 -0.695434
H 1.238562 -0.808884 -0.104592
C 0.682903 1.172574 0.255271
O 0.605169 0.960297 1.488762
N -0.612625 -0.425095 -0.982436
N 0.659978 2.400903 -0.240562
H 0.613575 2.533733 -1.242792
C 0.485925 3.573522 0.603686
H 0.561830 4.458721 -0.026939
H -0.493023 3.558633 1.091006
H 1.266191 3.605993 1.367619
C -1.072887 -1.687045 -1.000723
O -2.294270 -1.910680 -1.159780
C -0.106949 -2.822286 -0.819637
H 0.252737 -2.843838 0.214839
H -0.621508 -3.759876 -1.031885
H 0.758590 -2.724790 -1.482135
H -1.305798 0.326068 -1.088314
O -0.309839 -1.516655 2.467740
H -0.338527 -1.373739 3.424345
H 0.039720 -0.676867 2.101628
O -2.277277 1.524332 1.843272
H -1.309243 1.446279 1.911061
H -2.588412 0.591723 1.798674
O 3.405480 -2.587121 0.575955
H 3.223167 -1.766233 1.083254
H 2.675473 -2.660760 -0.053849
O 3.123169 -0.277413 2.142728
H 2.239568 0.109963 1.994427
H 3.725402 0.322874 1.679656
O -2.951292 -1.182309 1.679045
H -2.977210 -1.394016 0.730323
H -2.034076 -1.415524 1.952341
O -4.356042 -0.105102 -1.582497
H -3.627382 -0.753105 -1.456312
H -4.905816 -0.198362 -0.791859
H -3.245878 1.286635 -1.168164
H -2.435693 1.842357 0.034223
O -2.486082 1.866708 -0.946457
43
cysteine_pep_anion_7sol_ox_conf_8
Eopt -1428.229897
C -1.377559 -0.627701 1.488840
H -2.203327 0.075720 1.657685
H -0.752289 -0.581915 2.390281
S -2.106050 -2.266464 1.388063
C -0.576325 -0.194524 0.255131
H -1.217425 -0.276405 -0.624459
C -0.156511 1.268953 0.444293
O 0.853514 1.561426 1.124015
N 0.580456 -1.052281 0.085714
N -0.938974 2.179308 -0.112964
H -1.765545 1.865667 -0.638258
C -0.705054 3.603090 0.057232
H -0.729728 3.876333 1.116083
H 0.265602 3.887964 -0.358322
H -1.491435 4.142781 -0.470504
C 1.287580 -1.214220 -1.048276
O 2.364301 -1.848467 -1.025220
C 0.758960 -0.622691 -2.322379
H 0.771210 0.471048 -2.259132
H 1.393025 -0.937898 -3.151317
H -0.270635 -0.937815 -2.514592
H 1.048357 -1.359332 0.934678
O 2.059420 -0.221066 2.984517
H 2.771707 -0.562951 2.402115
H 1.582214 0.406685 2.405926
O 3.417479 1.591214 -0.000166
H 2.504485 1.615134 0.355584
H 3.883179 2.320056 0.432909
O -4.933526 0.489795 0.690872
H -5.069270 -0.434599 0.435089
H -5.793931 0.908299 0.544100
O -3.172866 -1.401234 -2.337277
H -3.889715 -1.707278 -1.762463
H -2.376431 -1.808527 -1.966463
O 4.015305 -0.935277 1.088865
H 3.487121 -1.446239 0.443466
H 3.961854 -0.022799 0.732827
O 4.212109 0.015823 -2.232967
H 3.675970 -0.762774 -1.994365
H 3.979566 0.658771 -1.535984
H -3.865734 1.084281 -0.675046
H -3.154815 0.389848 -1.845759

O -3.306252 1.276878 -1.457814
43
cysteine_pep_anion_7sol_ox_conf_9
Eopt -1428.224610
C 0.719878 0.864673 -1.577076
H 0.669915 1.805696 -1.014726
H 0.134342 1.023795 -2.492166
S 2.421680 0.610788 -2.087085
C 0.096892 -0.287171 -0.774284
H 0.229542 -1.212522 -1.338991
C 0.779396 -0.408139 0.591030
O 0.627559 0.481717 1.459592
N -1.325203 -0.013717 -0.621794
N 1.549629 -1.467166 0.786286
H 1.614555 -2.178126 0.052057
C 2.264411 -1.679375 2.033253
H 1.565038 -1.880397 2.850848
H 2.924049 -2.538195 1.907687
H 2.860012 -0.799829 2.290701
C -2.250710 -0.885170 -0.194209
O -3.440111 -0.518913 -0.049432
C -1.845480 -2.293194 0.131522
H -2.732340 -2.928116 0.134271
H -1.111892 -2.696852 -0.570328
H -1.403286 -2.310992 1.134567
H -1.631847 0.955486 -0.766099
O 3.123506 1.970723 1.336907
H 3.793566 1.287737 1.124847
H 2.290645 1.477175 1.444794
O -0.259762 3.089436 1.170288
H -0.000685 2.142861 1.224679
H 0.524874 3.530933 0.815196
O 1.770891 -3.465784 -1.330171
H 1.728387 -2.894374 -2.110929
H 2.694918 -3.750820 -1.290024
O 5.162827 0.100830 0.760457
H 5.359425 0.177386 -0.183876
H 4.807860 -0.793693 0.863538
O -5.456783 -2.261672 0.770362
H -4.738893 -1.657153 0.482660
H -6.270018 -1.751610 0.656772
O -4.449963 2.052680 0.072366
H -4.097345 1.134832 0.053233
H -4.446052 2.300765 1.007264
H -1.404989 3.032807 -0.234077
H -2.887511 2.726451 -0.602984
O -2.000222 2.866056 -0.997668
46
cysteine_pep_anion_8sol_conf_10
Eopt -1504.833529
C -0.988441 -0.166867 1.741332
H -0.901039 0.886471 1.450281
H -0.523945 -0.270483 2.726785
S -2.750521 -0.643814 1.865333
C -0.145945 -1.014102 0.768258
H -0.315133 -2.072483 0.982928
C -0.531759 -0.712933 -0.680378
O -0.124094 0.330544 -1.243177
N 1.258912 -0.694029 0.996366
N -1.307292 -1.594129 -1.296005
H -1.669654 -2.382801 -0.756764
C -1.815673 -1.380479 -2.639870
H -2.346926 -2.280865 -2.949485
H -2.503740 -0.530279 -2.670949
H -0.991941 -1.194954 -3.334053
C 2.318730 -1.287175 0.436600
O 3.470498 -0.828313 0.639837
C 2.116611 -2.486878 -0.441427
H 3.048711 -3.051605 -0.493565
H 1.314520 -3.137488 -0.084975
H 1.861187 -2.148767 -1.452811
H 1.458141 0.155646 1.532711
O -2.184661 2.155494 -1.881137
H -2.918030 1.727123 -1.381072
H -1.442273 1.526446 -1.780316
O 1.022715 2.580437 -0.090550
H 0.636997 1.738724 -0.422067
H 0.275656 3.220852 -0.107725
O -2.627495 -3.511612 0.521077
H -2.735461 -2.656015 1.015900
H -3.475302 -3.645127 0.073905
O -4.197675 0.886081 -0.464891
H -4.679983 1.601624 -0.026386
H -3.713488 0.418764 0.272760
O 5.390707 -1.198301 -1.344872
H 4.714419 -1.125349 -0.637204
H 5.461872 -0.301060 -1.698126
O 3.767773 1.860812 -0.261219
H 3.666327 0.960629 0.105492
H 2.861414 2.222651 -0.254584
O -1.260176 4.158504 -0.254109
H -1.050581 4.913693 -0.821232
H -1.661377 3.488375 -0.860539
H 2.718545 1.878352 2.411589
H 1.456420 2.239914 1.602969
O 1.752472 1.869698 2.466740
46
cysteine_pep_anion_8sol_conf_12
Eopt -1504.835970
C 1.048110 -2.113113 0.334121
H 0.552804 -2.287619 -0.628002
H 0.633662 -2.831625 1.048183
S 2.852141 -2.378168 0.160931
C 0.651659 -0.713631 0.833910
H 1.143031 -0.529483 1.792418
C 1.072258 0.354008 -0.175059
O 0.469334 0.464651 -1.272613
N -0.796188 -0.687520 1.023375
N 2.086600 1.140026 0.147341
H 2.616848 0.919229 0.992161

C 2.629489 2.119812 -0.777914
H 3.355615 2.731610 -0.242096
H 3.124443 1.627727 -1.621691
H 1.833932 2.762183 -1.161995
C -1.509996 0.285853 1.602165
O -2.764471 0.231851 1.621177
C -0.799264 1.461244 2.205290
H -0.546257 2.170540 1.408536
H -1.471065 1.959697 2.905520
H 0.124035 1.181319 2.717978
H -1.328060 -1.430445 0.563176
O -1.762486 -0.864308 -2.293497
H -2.441967 -0.255530 -1.927944
H -0.928482 -0.516189 -1.911873
O -1.206479 2.679081 -1.337201
H -0.571709 1.931538 -1.387629
H -1.212330 3.062195 -2.226371
O 2.513287 -1.120850 -2.772574
H 2.729625 -1.523287 -1.893431
H 1.794215 -0.501984 -2.560352
O 3.784679 -0.120866 2.210025
H 4.673059 0.160214 1.949226
H 3.575584 -0.884145 1.608689
O -3.451406 1.021650 -1.073492
H -3.368924 0.715350 -0.151044
H -2.742567 1.696216 -1.158065
O -4.400511 -1.897961 0.938470
H -3.816314 -1.153199 1.205648
H -4.987992 -1.518306 0.269993
O 0.134603 4.549220 0.279159
H -0.320261 3.878702 -0.274487
H 0.891845 4.078527 0.653842
H -2.102085 -2.276853 -1.213235
H -3.065248 -2.663432 -0.053617
O -2.237313 -2.940573 -0.500519
46
cysteine_pep_anion_8sol_conf_13
Eopt -1504.835879
C -1.705398 -0.827908 -1.729819
H -1.145839 -1.771085 -1.727728
H -1.683232 -0.438222 -2.752277
S -3.434305 -1.137542 -1.213931
C -0.947481 0.181160 -0.856572
H -1.455307 1.147014 -0.904088
C -0.865656 -0.297832 0.591893
O -0.194057 -1.314044 0.890107
N 0.407060 0.330133 -1.380712
N -1.508233 0.403775 1.513899
H -2.078956 1.182861 1.212179
C -1.566508 -0.004107 2.907288
H -2.021860 -0.994465 3.000981
H -0.562673 -0.032223 3.339463
H -2.170557 0.722913 3.449969
C 1.223999 1.382683 -1.212292
O 2.430927 1.298120 -1.535842
C 0.661502 2.657596 -0.654620
H -0.180588 3.006067 -1.261229
H 0.302393 2.503028 0.368289
H 1.442235 3.418694 -0.647801
H 0.859255 -0.525938 -1.691577
O 2.472260 -0.678860 1.642677
H 2.861382 -0.983689 0.796813
H 1.513579 -0.809353 1.513036
O 1.398253 -2.945609 -0.784967
H 0.788214 -2.409793 -0.238785
H 2.210014 -2.406572 -0.833692
O -4.520954 1.367992 0.373585
H -4.121819 0.599106 -0.117573
H -4.467649 1.120384 1.308072
O -2.355359 -3.295705 0.891589
H -1.517563 -2.844064 1.092559
H -2.790373 -2.668488 0.259410
O 3.639794 -1.163759 -0.929806
H 3.239769 -0.327447 -1.251877
H 4.499997 -0.888417 -0.542321
O 3.731352 1.737183 1.006112
H 3.423193 1.814672 0.083603
H 3.187235 0.996616 1.353988
O -2.919128 3.616561 0.057395
H -2.015795 3.367901 0.297202
H -3.445689 2.792810 0.169076
H 5.941180 -0.571333 1.183584
H 5.179142 0.672641 0.676312
O 5.824994 -0.016861 0.399116
46
cysteine_pep_anion_8sol_conf_17
Eopt -1504.833372
C -1.824076 0.039036 -1.628778
H -1.497047 -0.872688 -2.141263
H -1.841834 0.844902 -2.369658
S -3.499095 -0.204859 -0.925667
C -0.776528 0.416694 -0.579089
H -1.066378 1.355933 -0.109759
C -0.628970 -0.668112 0.487861
O -0.205438 -1.806272 0.182739
N 0.524414 0.609651 -1.212760
N -0.918733 -0.333712 1.739761
H -1.337115 0.575295 1.908287
C -0.780496 -1.265618 2.845396
H -1.004860 -0.734495 3.770443
H -1.471550 -2.107042 2.736187
H 0.241594 -1.650673 2.892115
C 1.481244 1.465413 -0.822164
O 2.624225 1.400458 -1.333654
C 1.145130 2.524663 0.188093
H 0.404245 3.213481 -0.232708
H 0.718750 2.097808 1.099874
H 2.049602 3.079856 0.437382
H 0.815676 -0.115666 -1.865174
O 2.510642 -2.135171 0.838628

H 2.895175 -1.826838 -0.011936
H 1.546135 -2.129440 0.678926
O 0.983034 -2.403498 -2.323384
H 0.512854 -2.270104 -1.475321
H 1.899409 -2.124835 -2.139206
O -3.098092 1.918924 1.369129
H -3.325314 1.225039 0.688649
H -3.948815 2.217571 1.721514
O -3.275114 -3.096924 0.374874
H -3.991109 -3.591488 -0.047133
H -3.325223 -2.192727 -0.033345
O 3.481170 -1.221840 -1.617926
H 3.264102 -0.263284 -1.593347
H 4.447066 -1.275774 -1.633666
O 4.509077 1.480683 0.805958
H 3.973862 1.563591 -0.006232
H 3.900905 1.019770 1.423854
O 2.711104 0.078842 2.451074
H 1.855224 0.523865 2.375854
H 2.621960 -0.722960 1.881545
H -2.593627 3.152314 0.123957
H -1.835473 3.258473 -1.211294
O -2.348143 3.787778 -0.584287
46
cysteine_pep_anion_8sol_conf_20
Eopt -1504.836531
C 2.278454 -0.786997 -0.717765
H 2.840944 0.040155 -1.161338
H 1.676261 -1.217525 -1.526543
S 3.458497 -2.015533 -0.040658
C 1.328309 -0.205362 0.348554
H 1.867144 -0.156212 1.296971
C 0.934302 1.221688 -0.035628
O -0.122975 1.483700 -0.647578
N 0.170198 -1.061567 0.518762
N 1.817333 2.154502 0.307170
H 2.696322 1.842064 0.725818
C 1.667245 3.546203 -0.080764
H 1.643984 3.649733 -1.169977
H 0.744785 3.962242 0.332915
H 2.516637 4.104122 0.313503
C -0.714680 -1.003081 1.523544
O -1.735351 -1.731057 1.508712
C -0.457878 -0.055228 2.658756
H 0.538962 -0.206439 3.084219
H -0.520479 0.981454 2.309057
H -1.210177 -0.212239 3.432221
H -0.088969 -1.650225 -0.267191
O -1.520183 -0.235911 -2.351447
H -1.898819 -0.998701 -1.857763
H -1.001851 0.270275 -1.690653
O -2.724302 2.073544 0.316337
H -1.795141 1.831516 0.138223
H -3.183132 1.858324 -0.527613
O 5.847457 -0.752757 -1.704225
H 5.127785 -1.188112 -1.176547
H 6.200550 -0.072496 -1.113893
O 4.397001 0.850914 1.203353
H 4.933730 1.199162 0.476922
H 4.207232 -0.081126 0.935419
O -2.828515 -2.291045 -0.993419
H -2.424029 -2.183672 -0.105560
H -3.698008 -1.845058 -0.911624
O -3.934213 0.043683 1.766169
H -3.249324 -0.652553 1.816323
H -3.469102 0.792461 1.323779
O -3.902185 1.192017 -2.064869
H -3.071573 0.710788 -2.283293
H -4.463212 0.513330 -1.637502
H -4.846115 -0.509222 0.355490
H -6.037863 -1.187946 -0.400750
O -5.164198 -0.795520 -0.535756
46
cysteine_pep_anion_8sol_conf_21
Eopt -1504.829939
C -1.387567 -1.349216 1.338099
H -1.124842 -0.551136 2.042582
H -0.896326 -2.263515 1.686704
S -3.201600 -1.596777 1.300031
C -0.770979 -1.004751 -0.029157
H -1.056993 -1.757461 -0.765831
C -1.212155 0.375820 -0.502286
O -0.761115 1.399122 0.062643
N 0.685496 -0.957372 0.051122
N -2.049304 0.439923 -1.528554
H -2.480642 -0.427428 -1.851885
C -2.594607 1.703007 -1.997345
H -3.135790 2.216446 -1.196919
H -1.795561 2.354825 -2.359051
H -3.280898 1.493206 -2.817784
C 1.534867 -1.989868 -0.045160
O 2.748820 -1.822339 0.217577
C 1.008895 -3.319614 -0.502686
H 0.608313 -3.236015 -1.518573
H 1.818321 -4.049956 -0.495418
H 0.201770 -3.668842 0.148516
H 1.076611 -0.091709 0.405764
O 0.896351 3.126547 -1.440600
H 1.694422 2.559152 -1.546110
H 0.248422 2.551562 -0.986246
O 1.253556 2.028660 1.973102
H 0.520822 1.738536 1.393276
H 1.451119 2.938559 1.658311
O -3.565749 -2.094071 -1.824958
H -3.508731 -2.006973 -0.836379
H -4.442427 -1.750677 -2.048849
O -3.894049 1.490102 1.563843
H -4.640301 1.530398 2.177976
H -3.704572 0.519201 1.467522
O 4.651046 -3.842713 -0.035131

H 3.963761 -3.145857 0.043792
H 5.489938 -3.368773 0.043379
O 3.566474 0.791684 0.962267
H 3.329922 -0.146019 0.800722
H 2.763302 1.193014 1.365564
O 1.736694 4.520295 0.781643
H 1.014561 5.097426 1.066816
H 1.421802 4.118094 -0.063330
H 3.362639 1.321272 -0.724459
H 3.882212 2.194523 -1.894605
O 3.183056 1.567271 -1.663229
46
cysteine_pep_anion_8sol_conf_23
Eopt -1504.829427
C 0.915245 -1.494283 -1.356192
H 0.726967 -0.595883 -1.955874
H 0.259407 -2.282742 -1.739705
S 2.666580 -2.006750 -1.512347
C 0.469518 -1.206503 0.088239
H 0.659618 -2.077940 0.717058
C 1.190378 0.011818 0.653363
O 0.890418 1.148816 0.225400
N -0.954304 -0.888833 0.138661
N 2.093242 -0.185879 1.603869
H 2.376874 -1.146540 1.800752
C 2.888387 0.900982 2.149317
H 2.248288 1.624564 2.660684
H 3.592462 0.480069 2.867198
H 3.443980 1.414479 1.358976
C -1.975826 -1.753864 0.195287
O -3.148475 -1.339572 0.036292
C -1.684971 -3.197644 0.486317
H -2.616656 -3.763744 0.475877
H -0.999270 -3.616473 -0.256699
H -1.214055 -3.295592 1.470122
H -1.195223 0.069259 -0.091992
O 1.485521 3.811917 0.682501
H 2.054043 4.084272 -0.051465
H 1.394741 2.839655 0.577825
O -0.846388 2.103727 -1.830176
H -0.208950 1.650388 -1.243424
H -0.955807 2.979947 -1.395443
O 3.150105 -2.964301 1.490937
H 3.045650 -2.740095 0.528149
H 4.084896 -2.798965 1.679028
O 3.676472 1.007049 -1.591970
H 4.421439 1.018003 -2.208949
H 3.401885 0.052866 -1.552658
O -3.425152 1.318851 -0.972804
H -3.384434 0.397270 -0.641950
H -2.534951 1.495778 -1.352037
O -5.409381 -2.954007 0.212462
H -4.588485 -2.417798 0.158998
H -6.105508 -2.314447 0.414830
O -1.092297 4.401505 -0.258180
H -1.700930 3.928375 0.351552
H -0.196757 4.267215 0.123176
H -3.634723 3.410558 1.455308
H -3.144483 2.289346 0.503041
O -2.866355 2.855528 1.261721
46
cysteine_pep_anion_8sol_conf_24
Eopt -1504.829543
C 1.481006 -1.653360 -0.983705
H 1.759849 -1.330393 -1.993708
H 0.911779 -2.580848 -1.089025
S 2.982572 -1.994056 0.018065
C 0.511412 -0.618183 -0.376840
H 0.386944 -0.832184 0.689089
C 0.976506 0.840683 -0.432439
O 0.148581 1.782773 -0.491589
N -0.797930 -0.716882 -1.001285
N 2.280751 1.057467 -0.372302
H 2.884819 0.238940 -0.248066
C 2.873093 2.382591 -0.422355
H 2.497536 2.935882 -1.286730
H 2.653961 2.950859 0.485866
H 3.953313 2.264220 -0.515327
C -1.799546 -1.501122 -0.583262
O -2.870201 -1.554263 -1.234795
C -1.624429 -2.295656 0.680530
H -2.480482 -2.959702 0.803010
H -0.706710 -2.890747 0.660673
H -1.570864 -1.619494 1.541471
H -0.967746 -0.188682 -1.851650
O 0.421984 3.128998 2.041831
H -0.420135 3.562974 2.240024
H 0.336412 2.845610 1.110935
O -2.215072 1.405566 1.049155
H -1.482951 1.534657 0.413024
H -1.773066 0.927086 1.779917
O 1.675989 -1.355877 2.883476
H 2.105278 -1.539591 2.009623
H 2.366136 -0.925326 3.407893
O 5.115439 -0.554366 -1.917755
H 4.934123 0.386369 -1.779498
H 4.486794 -1.015233 -1.310624
O -5.190208 -1.760875 0.341343
H -4.374073 -1.775916 -0.201175
H -5.407363 -0.820291 0.404294
O -3.933994 1.054910 -1.134213
H -3.676000 0.149000 -1.393000
H -3.505430 1.150477 -0.258732
O -0.362756 0.586971 3.034835
H 0.331035 -0.091791 2.890666
H 0.041367 1.434270 2.753645
H -2.597193 1.937849 -2.059629
H -1.089409 2.163191 -1.862545
O -1.815648 2.351151 -2.485529
46

cysteine_pep_anion_8sol_conf_26
Eopt -1504.839732

C	-1.683438	-0.437713	1.346694
H	-0.940974	-0.042919	2.049014
H	-1.673922	-1.528054	1.448339
S	-3.349835	0.213842	1.746043
C	-1.216236	-0.115152	-0.083390
H	-1.976290	-0.429083	-0.801344
C	-0.931653	1.373821	-0.245173
O	0.074401	1.875562	0.309855
N	0.034014	-0.798219	-0.402402
N	-1.753369	2.086254	-1.001758
H	-2.620855	1.648080	-1.316661
C	-1.596916	3.522252	-1.159307
H	-0.625719	3.753666	-1.604136
H	-2.386500	3.880748	-1.819594
H	-1.674964	4.032757	-0.194433
C	0.179804	-2.058921	-0.831026
O	1.328178	-2.562046	-0.896158
C	-1.036996	-2.836210	-1.239193
H	-0.725604	-3.801427	-1.639659
H	-1.687838	-3.000677	-0.374211
H	-1.612395	-2.295977	-1.997672
H	0.888231	-0.337535	-0.101015
O	1.884621	0.808084	2.229919
H	2.082030	-0.112777	1.945322
H	1.208636	1.133305	1.601043
O	2.476856	1.869742	-1.230613
H	1.611412	1.880708	-0.777973
H	3.125738	1.937482	-0.493497
O	-4.477394	-2.409960	0.315936
H	-4.189472	-1.588669	0.790853
H	-3.972770	-2.395731	-0.509906
O	-4.376474	0.777501	-1.233919
H	-4.262805	-0.068048	-1.691449
H	-4.132850	0.578945	-0.292103
O	2.780881	-1.736797	1.454429
H	2.298541	-2.065102	0.669691
H	3.640436	-1.435029	1.090502
O	3.242584	-0.707442	-1.940108
H	2.599329	-1.352242	-1.583348
H	2.870848	0.177088	-1.716015
O	4.176468	1.799216	0.985553
H	3.404363	1.509921	1.523362
H	4.625711	0.962925	0.748485
H	5.827995	-1.260271	-0.022403
H	4.511677	-0.768370	-0.716954
O	5.031562	-0.731347	0.123427

46
cysteine_pep_anion_8sol_conf_27
Eopt -1504.829824

C	0.771664	0.103334	-1.483967
H	0.882694	1.191220	-1.419168
H	0.078422	-0.102523	-2.305061
S	2.383821	-0.676863	-1.876190
C	0.108449	-0.416639	-0.195015
H	0.216531	-1.502483	-0.158453
C	0.764924	0.203114	1.041030
O	0.531435	1.391916	1.362639
N	-1.313410	-0.093731	-0.227192
N	1.585590	-0.567224	1.742231
H	1.796158	-1.504039	1.393408
C	2.321868	-0.073721	2.893319
H	2.920893	0.802025	2.630255
H	1.635755	0.198359	3.700918
H	2.982597	-0.868749	3.239940
C	-2.279065	-0.740310	0.436462
O	-3.471595	-0.359175	0.351770
C	-1.915023	-1.932204	1.275222
H	-2.820916	-2.355138	1.710168
H	-1.413704	-2.696084	0.671918
H	-1.234631	-1.640868	2.081671
H	-1.596682	0.704821	-0.803294
O	2.974411	2.841543	0.768561
H	3.532019	2.052327	0.593458
H	2.128992	2.456665	1.063264
O	-0.296529	3.482875	-0.246489
H	-0.062699	2.685319	0.279443
H	0.507471	3.680273	-0.747933
O	4.440502	0.509562	0.219249
H	3.786992	0.130059	-0.425401
H	5.178785	0.808156	-0.330997
O	2.487669	-3.004754	0.352257
H	3.402136	-3.036917	0.667627
H	2.506172	-2.358059	-0.395884
O	-4.435851	1.988727	-0.732066
H	-4.089902	1.164827	-0.319608
H	-4.406599	2.649986	-0.026641
O	-5.647405	-1.718344	1.417681
H	-4.852167	-1.271599	1.053455
H	-6.057829	-2.147413	0.654835
O	0.376857	-3.111021	-2.643636
H	-0.321473	-2.909926	-2.004190
H	1.078400	-2.448025	-2.446729
H	-2.873918	2.260073	-1.648792
H	-1.406037	2.744071	-1.458110
O	-1.982090	2.217258	-2.054798

46
cysteine_pep_anion_8sol_conf_30
Eopt -1504.826896

C	1.189673	-1.049025	-1.037352
H	0.656668	-0.859779	-1.975896
H	0.820345	-2.001261	-0.644771
S	2.995473	-1.193714	-1.353399
C	0.781386	0.023253	-0.009416
H	1.414190	-0.075481	0.876995
C	0.934435	1.484153	-0.453669
O	0.219540	2.380120	0.050745
N	-0.600168	-0.177686	0.389949
N	1.870454	1.761590	-1.350642

H 2.479269 0.993715 -1.649234
C 2.156880 3.120204 -1.775913
H 2.453969 3.741318 -0.925570
H 2.974298 3.085270 -2.496508
H 1.280555 3.569181 -2.251216
C -1.024401 -0.784170 1.500534
O -2.253121 -0.980367 1.686435
C -0.018178 -1.236778 2.521420
H 0.645723 -1.991731 2.086836
H 0.599363 -0.403735 2.871604
H -0.547034 -1.671919 3.369978
H -1.302636 0.046347 -0.320183
O -0.231207 2.388641 2.828192
H -0.830326 1.674259 3.084518
H -0.117005 2.281471 1.861496
O -2.496989 3.064604 0.048147
H -1.578596 2.725776 0.040463
H -2.679499 3.286825 -0.875868
O 4.058576 0.206208 1.390035
H 3.750696 -0.191450 0.540405
H 3.468056 -0.176536 2.054914
O 3.107242 -3.146339 1.167731
H 2.915943 -2.544516 1.901052
H 3.134381 -2.555319 0.374428
O -3.506446 -2.731484 -0.106174
H -3.064158 -2.124982 0.524136
H -4.301350 -2.251521 -0.380125
O -4.009507 0.727247 0.247238
H -3.451698 0.134379 0.791880
H -3.527943 1.584252 0.257767
O -2.016801 -2.454284 -2.421870
H -2.526970 -2.640413 -1.599544
H -2.609220 -2.710134 -3.142619
H -2.234578 -0.649273 -2.224107
H -3.077769 0.343186 -1.367136
O -2.331662 0.301697 -2.000242
46
cysteine_pep_anion_8sol_conf_4
Eopt -1504.838980
C 1.516216 -1.753971 0.479244
H 0.932727 -2.054998 -0.398134
H 1.268578 -2.449166 1.287557
S 3.304352 -1.857637 0.099116
C 1.029312 -0.365512 0.930319
H 1.612806 -0.055938 1.801078
C 1.193920 0.659423 -0.191001
O 0.425468 0.642946 -1.184784
N -0.377696 -0.477297 1.307082
N 2.168882 1.546912 -0.076433
H 2.830432 1.440616 0.694780
C 2.475004 2.497377 -1.131889
H 1.630506 3.170942 -1.298984
H 3.341694 3.081732 -0.822254
H 2.705620 1.978395 -2.067471
C -1.126183 0.471778 1.880951
O -2.354334 0.289979 2.070470
C -0.494516 1.775784 2.272361
H -0.459631 2.430576 1.393175
H -1.115874 2.255369 3.030106
H 0.523144 1.658534 2.651908
H -0.869159 -1.317404 0.989333
O -1.372959 -1.308444 -2.038212
H -2.260860 -0.910704 -1.912383
H -0.744090 -0.653813 -1.666550
O -1.305932 2.819860 -1.394457
H -0.673593 2.071248 -1.359004
H -1.632308 2.822719 -2.305430
O 4.276292 0.657504 1.797156
H 4.053302 -0.177760 1.306086
H 5.068255 0.994699 1.354929
O 2.473488 -0.879015 -2.849850
H 1.713014 -0.323977 -2.610211
H 2.826390 -1.177131 -1.973374
O -3.988026 -1.667124 0.940217
H -3.419117 -0.999540 1.382662
H -4.132547 -1.288216 0.048600
O -3.524810 1.910869 0.004499
H -3.196915 1.433204 0.790795
H -2.713691 2.257726 -0.433122
O -1.655728 -3.003109 0.166199
H -1.505624 -2.514886 -0.673773
H -2.533592 -2.679722 0.462867
H -4.631075 -0.192937 -2.186504
H -3.851772 0.549633 -1.051117
O -3.940595 -0.316416 -1.520720
46
cysteine_pep_anion_8sol_conf_6
Eopt -1504.838435
C 2.193744 -1.161613 1.297783
H 1.860067 -2.076214 0.792546
H 2.169472 -1.356866 2.374477
S 3.900749 -0.746941 0.774636
C 1.163447 -0.056565 1.025801
H 1.460419 0.846973 1.560785
C 1.047730 0.234984 -0.469875
O 0.539175 -0.615736 -1.242577
N -0.138504 -0.485838 1.523789
N 1.494047 1.401640 -0.908006
H 2.006981 1.992254 -0.253445
C 1.512647 1.742008 -2.320818
H 0.494976 1.804474 -2.715164
H 1.998129 2.711730 -2.431895
H 2.069138 0.991875 -2.890948
C -1.144946 0.307973 1.919207
O -2.278289 -0.178958 2.143378
C -0.879958 1.772350 2.119028
H -0.114181 1.915134 2.888903
H -0.518680 2.235855 1.196801
H -1.802139 2.263004 2.431720
H -0.382026 -1.457668 1.351624

O -1.862479 0.263536 -2.445555
H -2.609857 -0.208846 -2.010629
H -1.046261 -0.096243 -2.045484
O -0.545881 -3.122483 -0.610134
H -0.166238 -2.246439 -0.829487
H -1.403091 -2.914197 -0.185074
O 3.073723 -1.850689 -2.123263
H 3.436776 -1.501024 -1.270236
H 2.141972 -1.571552 -2.088951
O 3.584370 2.428697 0.950389
H 4.168652 2.814031 0.282040
H 3.771441 1.453496 0.915997
O -4.095863 1.377982 0.573327
H -3.565708 0.883954 1.229598
H -3.428725 1.883770 0.056161
O -2.946869 -2.457107 0.713495
H -2.723758 -1.714604 1.320383
H -3.465056 -3.083835 1.237004
O -2.217350 2.627744 -1.083154
H -2.738757 3.204671 -1.658434
H -2.044967 1.819329 -1.624421
H -4.214038 -0.013131 -0.611969
H -3.755015 -1.483785 -0.590796
O -4.107575 -0.796802 -1.196220
46
cysteine_pep_anion_8sol_conf_7
Eopt -1504.832883
C -0.759135 0.286219 1.607335
H -0.844170 1.243801 1.081754
H -0.148016 0.458277 2.498595
S -2.412432 -0.297950 2.133695
C 0.022179 -0.698960 0.718443
H -0.016093 -1.688413 1.180837
C -0.587656 -0.760931 -0.683610
O -0.380562 0.157017 -1.511917
N 1.413179 -0.261587 0.651066
N -1.338468 -1.816478 -0.967756
H -1.527288 -2.492112 -0.225764
C -2.029954 -1.965929 -2.236274
H -2.576497 -2.909098 -2.215581
H -2.735462 -1.146951 -2.400575
H -1.313769 -1.986184 -3.062835
C 2.437022 -0.957758 0.144045
O 3.585778 -0.454755 0.096783
C 2.195745 -2.342422 -0.383198
H 3.149848 -2.865076 -0.463699
H 1.516987 -2.918211 0.250675
H 1.753035 -2.273274 -1.383706
H 1.610182 0.699501 0.945154
O -2.833286 1.541765 -1.973574
H -3.417261 0.968614 -1.424613
H -1.969554 1.085134 -1.940754
O 0.385676 2.797496 -0.936715
H 0.198638 1.845620 -1.083577
H -0.482415 3.165692 -0.661588
O -2.184812 -3.379138 1.410506
H -2.295862 -2.443935 1.728886
H -3.070768 -3.646462 1.127365
O -4.372360 -0.049779 -0.305689
H -5.059309 0.554247 0.011160
H -3.723322 -0.111569 0.450471
O 5.800317 -1.670084 -1.060590
H 4.997025 -1.278475 -0.653051
H 6.513928 -1.457996 -0.443838
O 4.423720 2.127961 0.577539
H 4.123467 1.209769 0.389863
H 4.496866 2.546988 -0.291269
O -2.217722 3.615014 -0.284365
H -2.367655 4.445317 -0.757670
H -2.532718 2.908841 -0.899365
H 2.742095 2.599347 1.152465
H 1.297118 2.779406 0.600455
O 1.804215 2.646946 1.433956
46
cysteine_pep_anion_8sol_conf_8
Eopt -1504.835273
C 1.963756 -0.263780 -1.557683
H 1.575465 0.389571 -2.348067
H 1.890874 -1.289356 -1.933103
S 3.725887 0.127910 -1.210483
C 1.025214 -0.189144 -0.344927
H 1.425205 -0.816011 0.458672
C 0.814259 1.187852 0.295425
O -0.010643 1.310278 1.230919
N -0.299956 -0.683814 -0.701853
N 1.498952 2.223705 -0.169129
H 2.287696 2.022954 -0.782137
C 1.412387 3.535541 0.449860
H 2.060905 4.216226 -0.101569
H 0.386170 3.907882 0.408920
H 1.734902 3.496007 1.495262
C -0.743859 -1.935999 -0.552744
O -1.893389 -2.254308 -0.947853
C 0.145164 -2.955658 0.101158
H -0.367850 -3.917799 0.109881
H 1.087782 -3.052687 -0.447010
H 0.380316 -2.667082 1.130779
H -0.897865 -0.029094 -1.215825
O -2.365686 2.587911 0.362690
H -3.080066 1.929278 0.505287
H -1.572028 2.192434 0.773490
O -0.969368 -0.771811 2.754659
H -0.632480 -0.063113 2.160043
H -1.239045 -0.310064 3.560939
O 3.824363 -2.677798 0.302785
H 3.860762 -1.803074 -0.159889
H 3.037118 -2.611650 0.862245
O 3.612623 0.442299 2.021707
H 2.737761 0.099314 2.253717
H 3.647115 0.361531 1.037413

O -3.752729 -0.434651 -1.979021
H -3.096052 -1.084488 -1.643493
H -4.114479 -0.027104 -1.164376
O -3.295414 -1.643279 1.530544
H -2.979653 -1.993089 0.677250
H -2.471974 -1.340561 1.980062
O -1.740217 1.499728 -2.161823
H -2.501043 0.884326 -2.247788
H -1.931865 1.985610 -1.330265
H -5.243181 0.839341 0.900171
H -4.035787 -0.152042 0.995001
O -4.366714 0.658390 0.533200
46
cysteine_pep_anion_8sol_ox_conf_26
Eopt -1504.657689
C -1.493874 -0.359612 1.600762
H -0.671935 0.028520 2.211080
H -1.543542 -1.441499 1.796552
S -3.060977 0.297397 2.184271
C -1.214004 -0.136513 0.103092
H -2.050642 -0.511360 -0.490096
C -0.989682 1.347607 -0.175977
O 0.028656 1.911834 0.285264
N 0.006430 -0.820315 -0.293941
N -1.895297 1.978705 -0.903905
H -2.706562 1.463644 -1.258642
C -1.784219 3.397830 -1.200371
H -0.889531 3.598122 -1.796425
H -2.667085 3.696258 -1.765255
H -1.732437 3.980904 -0.277032
C 0.129259 -2.109538 -0.654035
O 1.271377 -2.601113 -0.796113
C -1.110435 -2.916913 -0.897704
H -0.826579 -3.918980 -1.219921
H -1.708718 -2.988463 0.016576
H -1.728423 -2.447889 -1.671056
H 0.878969 -0.334365 -0.102076
O 1.960933 0.921318 2.142603
H 2.146440 -0.009831 1.885665
H 1.249580 1.219184 1.540769
O 2.352468 1.816184 -1.393795
H 1.509833 1.851552 -0.902497
H 3.036597 1.920397 -0.693683
O -4.555216 -2.142374 0.082431
H -4.243689 -1.247392 0.285921
H -3.995012 -2.423525 -0.655127
O -4.199958 0.515612 -1.911090
H -3.913235 -0.408391 -1.951301
H -4.793829 0.546582 -1.147165
O 2.852675 -1.650011 1.443469
H 2.342489 -2.037986 0.706359
H 3.686382 -1.355439 1.017910
O 3.112762 -0.786707 -2.026265
H 2.498229 -1.418036 -1.601851
H 2.744325 0.104265 -1.824802
O 4.172922 1.867505 0.730068
H 3.440258 1.595153 1.328146
H 4.618102 1.026001 0.503475
H 5.804746 -1.213980 -0.232559
H 4.443208 -0.770879 -0.869785
O 5.009318 -0.690600 -0.062972
46
cysteine_pep_anion_8sol_ox_conf_6
Eopt -1504.657968
C 2.206904 0.854117 -1.609466
H 1.924298 1.873228 -1.310407
H 2.164137 0.831686 -2.705604
S 3.918050 0.598480 -1.129917
C 1.212916 -0.168787 -1.045632
H 1.527958 -1.170275 -1.341245
C 1.136918 -0.062003 0.477811
O 0.671074 0.977990 1.007508
N -0.096397 0.101282 -1.617414
N 1.586689 -1.080493 1.186409
H 1.938482 -1.907262 0.692850
C 1.590280 -1.072414 2.640368
H 0.569442 -1.032356 3.029870
H 2.068962 -1.989673 2.983129
H 2.148873 -0.211275 3.017364
C -1.087452 -0.791112 -1.792043
O -2.229121 -0.395991 -2.117863
C -0.790220 -2.251644 -1.618361
H -0.013527 -2.567968 -2.322633
H -0.430952 -2.460475 -0.606413
H -1.699921 -2.823495 -1.802848
H -0.361928 1.079155 -1.691311
O -1.725653 0.457385 2.422950
H -2.481279 0.783214 1.880465
H -0.916855 0.697862 1.930530
O -0.538450 3.260944 -0.142342
H -0.097309 2.467933 0.221441
H -1.342340 2.918100 -0.582526
O 2.791624 2.620255 1.956524
H 3.342819 1.976358 2.422675
H 2.030052 2.096029 1.642913
O 2.638938 -3.392607 -0.236235
H 3.373805 -3.691552 0.318143
H 3.066070 -2.951180 -0.984831
O -4.003350 -1.470046 -0.131583
H -3.488767 -1.185242 -0.911362
H -3.322708 -1.805704 0.495269
O -2.860218 2.193381 -1.355142
H -2.668537 1.309720 -1.743902
H -3.401416 2.660942 -2.006310
O -2.069893 -2.192624 1.746058
H -2.543501 -2.614526 2.476447
H -1.899861 -1.269386 2.052797
H -4.106959 0.203980 0.610251
H -3.645875 1.609385 0.176794
O -3.983713 1.116642 0.954885

46
cysteine_pep_anion_8sol_ox_conf_7
Eopt -1504.650269
C -0.647949 0.465856 1.599480
H -0.732450 1.432606 1.086338
H -0.085615 0.658663 2.522089
S -2.300204 -0.040322 2.079960
C 0.124324 -0.543660 0.736980
H 0.146319 -1.501210 1.260909
C -0.567260 -0.708237 -0.619480
O -0.576662 0.232576 -1.446861
N 1.484730 -0.051351 0.579130
N -1.173821 -1.863276 -0.847124
H -1.117968 -2.603688 -0.142067
C -1.903172 -2.126422 -2.076243
H -2.390205 -3.097180 -1.981930
H -2.661449 -1.358326 -2.248416
H -1.223301 -2.147146 -2.933467
C 2.549190 -0.783425 0.219190
O 3.669201 -0.238195 0.084755
C 2.380708 -2.254222 -0.029137
H 3.364250 -2.711340 -0.141915
H 1.843585 -2.746574 0.786267
H 1.812200 -2.411312 -0.952128
H 1.637730 0.954750 0.716863
O -3.232115 1.306299 -1.487517
H -3.816674 0.591783 -1.153414
H -2.345474 0.901786 -1.554650
O -0.111754 2.966329 -1.003764
H -0.192913 1.997982 -1.136963
H -0.943947 3.207546 -0.541701
O -1.038766 -3.961043 1.183119
H -1.001330 -3.431466 1.992800
H -1.932101 -4.333343 1.181240
O -4.984093 -0.716301 -0.652305
H -5.229788 -0.567222 0.271812
H -4.454251 -1.526410 -0.636267
O 5.947320 -1.525490 -0.864209
H 5.132698 -1.110266 -0.506816
H 6.640694 -1.288048 -0.233848
O 4.248809 2.449874 -0.204466
H 4.046436 1.490820 -0.124034
H 4.132178 2.649188 -1.143767
O -2.618319 3.362203 0.221149
H -3.003919 4.161535 -0.163820
H -2.950968 2.623078 -0.342127
H 2.640172 2.883165 0.574008
H 1.108032 2.994393 0.307278
O 1.770118 2.878053 1.026298
22
cysteine_pep_anion_conf_10
Eopt -893.444211
C 0.190150 1.269000 0.733974
H 0.483889 0.912950 1.729584
H -0.750958 1.813011 0.853487
S 1.461666 2.404229 0.046285
C -0.121026 0.037805 -0.149293
H -0.182713 0.369809 -1.192308
C 0.955008 -1.048937 -0.061835
O 0.665252 -2.231289 0.209652
N -1.387192 -0.574906 0.210716
N 2.203754 -0.647192 -0.301516
H 2.350613 0.368612 -0.367707
C 3.337898 -1.543692 -0.179714
H 3.423697 -1.944154 0.835985
H 3.247263 -2.380180 -0.878448
H 4.243081 -0.983533 -0.417215
C -2.577352 -0.084439 -0.168263
O -2.682740 0.964356 -0.834976
C -3.797530 -0.856877 0.257517
H -3.556603 -1.768585 0.809221
H -4.419168 -0.211832 0.886324
H -4.377919 -1.117224 -0.632790
H -1.346751 -1.473547 0.677198
22
cysteine_pep_anion_conf_11
Eopt -893.439002
C -0.446027 -1.524134 0.265212
H -0.283508 -1.223449 1.304836
H 0.195679 -2.390339 0.078885
S -2.205149 -2.012027 0.030758
C 0.051645 -0.410070 -0.683337
H -0.336909 -0.643890 -1.680565
C -0.459706 1.012008 -0.375635
O 0.244506 2.017561 -0.587177
N 1.501228 -0.412466 -0.842908
N -1.708606 1.094331 0.084297
H -2.229820 0.213294 0.182906
C -2.356428 2.365541 0.343569
H -3.371070 2.167246 0.691427
H -1.821775 2.928816 1.114526
H -2.404949 2.976212 -0.563733
C 2.454683 -0.142937 0.067967
O 3.657265 -0.153175 -0.269009
C 2.075403 0.121110 1.500434
H 1.098467 0.590732 1.623689
H 2.065344 -0.833391 2.038446
H 2.839571 0.759386 1.948446
H 1.851416 -0.569426 -1.780479
22
cysteine_pep_anion_conf_2
Eopt -893.441673
C -0.150067 1.240487 -0.822707
H -0.510175 0.887890 -1.797751
H 0.806604 1.741191 -1.000655
S -1.331892 2.438467 -0.084137
C 0.159919 0.005414 0.057807
H 0.279666 0.340663 1.092943
C -0.967251 -1.036095 0.049429
O -0.750249 -2.236991 -0.202822

N 1.384862 -0.656547 -0.359483
N -2.182563 -0.568898 0.341045
H -2.279626 0.453616 0.394256
C -3.360377 -1.415193 0.310765
H -4.225973 -0.804907 0.571523
H -3.516667 -1.847641 -0.683119
H -3.271761 -2.229521 1.035646
C 2.634931 -0.348002 0.026530
O 3.613867 -0.953260 -0.458224
C 2.817702 0.719797 1.072857
H 2.319442 1.652469 0.791727
H 2.392261 0.387663 2.026518
H 3.883984 0.907014 1.205975
H 1.298341 -1.402241 -1.041529
22
cysteine_pep_anion_conf_3
Eopt -893.441992
C 0.861960 -1.333147 1.212080
H 0.291724 -2.054528 1.806822
H 1.634264 -0.915282 1.867110
S 1.627027 -2.193011 -0.225461
C -0.109169 -0.184583 0.860333
H -0.655075 0.083914 1.767761
C 0.620161 1.094720 0.425660
O 0.438700 2.167131 1.036578
N -1.053057 -0.648875 -0.144618
N 1.438413 0.991071 -0.624923
H 1.660323 0.037894 -0.930924
C 2.240895 2.105383 -1.093648
H 2.817902 1.773411 -1.957538
H 1.601543 2.939535 -1.395673
H 2.929931 2.456322 -0.317924
C -2.293007 -0.191644 -0.390913
O -2.963733 -0.663653 -1.332578
C -2.849283 0.882614 0.504995
H -2.226796 1.781941 0.467427
H -3.860536 1.130571 0.179900
H -2.879295 0.541638 1.545267
H -0.672985 -1.350491 -0.781124
22
cysteine_pep_anion_conf_5
Eopt -893.441234
C -0.073862 1.212200 -0.754906
H -0.410453 0.865903 -1.740414
H 0.906283 1.674329 -0.901285
S -1.231890 2.462865 -0.068837
C 0.140814 -0.025335 0.147959
H 0.211465 0.311108 1.188072
C -1.027432 -1.017701 0.074608
O -0.843516 -2.221818 -0.189637
N 1.344752 -0.764211 -0.202899
N -2.234316 -0.507656 0.321692
H -2.293515 0.517395 0.379481
C -3.441646 -1.305437 0.215955
H -3.414318 -2.143519 0.918036
H -4.293438 -0.669488 0.459911
H -3.571439 -1.701608 -0.796698
C 2.621156 -0.425798 0.044191
O 3.552013 -1.178784 -0.315180
C 2.925023 0.877330 0.733040
H 2.106955 1.251616 1.351690
H 3.816298 0.744520 1.350206
H 3.149938 1.631341 -0.029550
H 1.197105 -1.671111 -0.636151
22
cysteine_pep_anion_conf_6
Eopt -893.444632
C 0.971988 1.399299 -1.156345
H 0.474439 2.117533 -1.816699
H 1.847967 1.022282 -1.695147
S 1.476346 2.242846 0.400002
C -0.001809 0.213169 -0.974907
H -0.417384 -0.049525 -1.951532
C 0.714994 -1.049277 -0.481773
O 0.813901 -2.052647 -1.214192
N -1.085140 0.618777 -0.099344
N 1.230489 -0.998250 0.751702
H 1.308236 -0.064109 1.160959
C 2.034772 -2.077994 1.293904
H 2.938446 -2.244877 0.697350
H 2.324150 -1.813942 2.311651
H 1.457792 -3.005996 1.321057
C -2.217402 -0.081571 0.065360
O -2.453624 -1.118819 -0.585612
C -3.202746 0.447909 1.072465
H -3.360500 -0.314073 1.842220
H -2.870848 1.374186 1.547648
H -4.160227 0.622985 0.572409
H -0.862059 1.388824 0.529747
22
cysteine_pep_anion_conf_8
Eopt -893.441466
C 0.832419 -1.536783 -0.628498
H 1.054495 -1.235948 -1.660481
H 0.245293 -2.458454 -0.682986
S 2.386262 -1.872287 0.294692
C -0.091758 -0.473038 0.002748
H -0.141444 -0.668102 1.078029
C 0.415956 0.971314 -0.184141
O -0.315555 1.870974 -0.638273
N -1.427288 -0.614473 -0.550415
N 1.674393 1.193845 0.202649
H 2.237346 0.363382 0.428534
C 2.309952 2.488876 0.051261
H 1.772228 3.253409 0.619494
H 3.328668 2.417135 0.434640
H 2.346906 2.796804 -0.999113
C -2.579852 -0.228761 0.030554
O -3.651676 -0.260745 -0.606849
C -2.541218 0.192626 1.475838

H -2.200626 -0.636322 2.105696
H -1.855724 1.032116 1.626711
H -3.544329 0.487745 1.786236
H -1.487885 -0.818028 -1.543062
22
cysteine_pep_anion_ox_conf_1
Eopt -893.273912
C 1.069265 -1.525095 0.836362
H 0.549353 -2.445970 1.131319
H 1.777782 -1.304160 1.641704
S 2.012388 -1.907049 -0.649551
C 0.050679 -0.384004 0.715125
H -0.420425 -0.294849 1.697787
C 0.768545 0.951267 0.477935
O 1.638719 1.321348 1.285766
N -0.968466 -0.696585 -0.264645
N 0.426484 1.665866 -0.594079
H -0.299678 1.314892 -1.203984
C 1.039899 2.947893 -0.894307
H 2.118328 2.835468 -1.036743
H 0.596662 3.332643 -1.812497
H 0.862996 3.661240 -0.084340
C -2.287130 -0.392046 -0.194296
O -3.039562 -0.676944 -1.140522
C -2.797035 0.278115 1.051838
H -3.850472 0.523803 0.914051
H -2.695886 -0.392572 1.912204
H -2.236225 1.192627 1.269976
H -0.665362 -1.107304 -1.143485
22
cysteine_pep_anion_ox_conf_10
Eopt -893.275521
C 0.049805 1.433770 0.606445
H 0.136335 1.260971 1.687005
H -0.862879 2.028064 0.463501
S 1.419139 2.469570 0.078723
C -0.105718 0.095783 -0.140838
H -0.150827 0.282899 -1.215858
C 1.047205 -0.847010 0.192350
O 1.095958 -1.406691 1.301663
N -1.336448 -0.558773 0.262617
N 1.977578 -1.015719 -0.747540
H 1.882533 -0.518086 -1.622973
C 3.159525 -1.831902 -0.527331
H 3.740829 -1.451555 0.317570
H 2.878664 -2.869186 -0.325219
H 3.771864 -1.796928 -1.427997
C -2.536266 -0.252206 -0.268647
O -2.654175 0.566203 -1.197518
C -3.730883 -0.958894 0.311006
H -3.469766 -1.652725 1.113202
H -4.426783 -0.207995 0.697448
H -4.237655 -1.506328 -0.489440
H -1.292729 -1.174149 1.066593
22
cysteine_pep_anion_ox_conf_11
Eopt -893.266078
C -0.237320 -1.505574 0.241758
H -0.131694 -1.270123 1.306851
H 0.513888 -2.274909 0.023565
S -1.848260 -2.281685 0.051580
C 0.022965 -0.277383 -0.659579
H -0.419297 -0.492626 -1.635375
C -0.646955 0.996655 -0.112275
O -0.004430 1.872063 0.485186
N 1.432632 -0.059928 -0.939724
N -1.966111 1.088527 -0.315708
H -2.436588 0.357135 -0.832896
C -2.752923 2.194951 0.201973
H -3.794834 2.031563 -0.073191
H -2.674933 2.249840 1.291569
H -2.415124 3.143832 -0.224523
C 2.487635 0.094423 -0.105131
O 3.589658 0.425211 -0.581811
C 2.350461 -0.196552 1.364559
H 1.447591 0.237026 1.797038
H 2.327053 -1.280535 1.520034
H 3.224335 0.208153 1.877435
H 1.641113 0.180093 -1.902590
22
cysteine_pep_anion_ox_conf_2
Eopt -893.273125
C -0.010964 1.560279 -0.505293
H -0.108816 1.518185 -1.598742
H 0.902475 2.133282 -0.301174
S -1.408274 2.500063 0.123293
C 0.140139 0.144328 0.069035
H 0.265962 0.200285 1.151459
C -1.076272 -0.718357 -0.268679
O -1.397161 -0.906890 -1.454748
N 1.307764 -0.507991 -0.505146
N -1.740075 -1.253805 0.756404
H -1.428755 -1.058633 1.698520
C -2.910396 -2.093187 0.564730
H -3.271584 -2.405113 1.544325
H -3.701608 -1.541087 0.049528
H -2.657234 -2.979713 -0.023309
C 2.566883 -0.471338 -0.013777
O 3.506516 -0.950221 -0.673014
C 2.782795 0.132379 1.347415
H 2.413577 1.162147 1.386419
H 2.251788 -0.446243 2.111039
H 3.849977 0.123789 1.571572
H 1.214822 -0.843077 -1.459629
22
cysteine_pep_anion_ox_conf_6
Eopt -893.276109
C 1.021206 1.593137 -0.803534
H 0.447403 2.468320 -1.135120
H 1.802708 1.440681 -1.555403

S 1.819006 2.043172 0.747930
C 0.101793 0.368507 -0.756088
H -0.296532 0.230577 -1.766214
C 0.906631 -0.901441 -0.454626
O 1.892813 -1.175979 -1.161078
N -1.021794 0.583846 0.128834
N 0.506031 -1.671537 0.556982
H -0.308434 -1.394400 1.088679
C 1.184228 -2.909199 0.900287
H 2.227250 -2.717063 1.167048
H 0.673488 -3.354187 1.753892
H 1.157873 -3.610396 0.061274
C -2.274333 0.145683 -0.136328
O -2.545201 -0.479232 -1.173608
C -3.324456 0.453050 0.894827
H -3.752735 -0.489801 1.248250
H -2.935522 1.013326 1.747913
H -4.124436 1.028383 0.419227
H -0.856139 1.050909 1.013141
22
cysteine_pep_anion_ox_conf_8
Eopt -893.273048
C 0.835175 -1.725208 -0.256507
H 0.951067 -1.822585 -1.344670
H 0.318282 -2.631592 0.081240
S 2.486380 -1.726913 0.453558
C -0.008294 -0.495570 0.088294
H -0.169081 -0.469878 1.166575
C 0.681494 0.793633 -0.373018
O 1.105881 0.901889 -1.536383
N -1.300368 -0.605654 -0.571317
N 0.769106 1.777405 0.524633
H 0.373311 1.634632 1.444394
C 1.348428 3.070262 0.202393
H 0.783585 3.560973 -0.595625
H 1.318428 3.692105 1.096753
H 2.387328 2.956138 -0.119189
C -2.512192 -0.303775 -0.052113
O -3.525139 -0.360124 -0.771099
C -2.597754 0.073432 1.402215
H -2.228925 -0.740075 2.035907
H -1.998861 0.964887 1.613550
H -3.640114 0.275348 1.651009
H -1.285346 -0.802363 -1.567820
23
cysteine_pep_neu_conf_1 Eopt
-893.918120
C 1.030929 -1.542441 0.871301
H 0.474525 -2.428476 1.185879
H 1.799767 -1.344553 1.619134
S 1.825191 -1.991307 -0.714040
H 2.610439 -0.903128 -0.818808
C 0.070720 -0.350970 0.799821
H -0.336810 -0.210982 1.806163
C 0.837155 0.938842 0.483528
O 1.772597 1.284042 1.227240
N -1.035492 -0.620714 -0.092687
N 0.470604 1.640049 -0.589088
H -0.302854 1.305973 -1.148544
C 1.130487 2.878316 -0.964460
H 0.655972 3.258541 -1.868832
H 1.035920 3.623684 -0.169651
H 2.192120 2.705378 -1.162152
C -2.290536 -0.165139 0.118989
O -2.573160 0.546311 1.096393
C -3.328775 -0.560159 -0.894287
H -4.132302 -1.096923 -0.380927
H -3.756181 0.348256 -1.329540
H -2.929047 -1.188488 -1.693338
H -0.855761 -1.177233 -0.920740
23
cysteine_pep_neu_conf_10
Eopt -893.913582
C 1.264038 -0.963854 1.003853
H 0.968888 -1.389096 1.966488
H 2.218370 -0.453570 1.146166
S 1.503723 -2.400632 -0.099653
H 1.933640 -1.719693 -1.177160
C 0.196285 0.052396 0.572088
H 0.099097 0.763948 1.396456
C 0.623156 0.808777 -0.694909
O 0.287399 0.403480 -1.820813
N -1.083042 -0.591139 0.383329
N 1.399323 1.889462 -0.536465
H 1.673022 2.350774 -1.396208
C 1.769132 2.535266 0.717721
H 2.420124 3.375084 0.475347
H 0.889178 2.917975 1.243196
H 2.317258 1.856177 1.374873
C -2.212074 0.125346 0.210292
O -2.218271 1.362632 0.333339
C -3.459436 -0.641666 -0.128647
H -3.783630 -0.350304 -1.132955
H -3.313083 -1.723866 -0.101262
H -4.250030 -0.363585 0.574030
H -1.086003 -1.568478 0.114831
23
cysteine_pep_neu_conf_12
Eopt -893.915648
C 1.272801 -1.531658 0.204817
H 1.027181 -2.483294 -0.272000
H 1.292225 -1.696251 1.284896
S 2.949690 -1.112708 -0.381440
H 3.144791 -0.042185 0.408540
C 0.218707 -0.498854 -0.173779
H 0.273007 -0.302075 -1.248059
C -1.197476 -1.007840 0.146685
O -1.402234 -1.958923 0.918363
N 0.459203 0.762775 0.512019
N -2.184346 -0.339530 -0.460074

H -1.934949 0.461858 -1.030068
C -3.585552 -0.622975 -0.204775
H -3.828126 -1.652082 -0.484040
H -3.828757 -0.478265 0.852409
H -4.187721 0.058558 -0.805546
C 0.171689 1.964849 -0.025078
O -0.366984 2.069861 -1.142230
C 0.522955 3.176046 0.792685
H 1.183817 3.817036 0.201530
H -0.393921 3.737079 0.998040
H 1.011915 2.928532 1.737533
H 0.856509 0.726788 1.443801
23
cysteine_pep_neu_conf_13
Eopt -893.913367
C -0.645382 -1.381160 -0.557742
H -0.148327 -2.348331 -0.666297
H -0.510803 -0.828271 -1.488994
S -2.441732 -1.641756 -0.393807
H -2.388395 -2.476126 0.660322
C -0.003101 -0.637813 0.626250
H -0.168479 -1.230390 1.527244
C 1.522740 -0.630342 0.441681
O 2.210497 -1.495204 1.014730
N -0.589419 0.667054 0.914725
N 2.046379 0.292416 -0.366776
H 1.406892 0.918609 -0.851264
C 3.470762 0.360130 -0.639532
H 3.650733 1.200045 -1.310571
H 4.034923 0.515394 0.284546
H 3.822940 -0.560037 -1.115840
C -0.828949 1.659390 0.035894
O -0.384442 1.640168 -1.127744
C -1.654935 2.814574 0.529692
H -2.589392 2.835740 -0.040593
H -1.890191 2.751905 1.594345
H -1.117180 3.745350 0.328860
H -1.008972 0.759522 1.830152
23
cysteine_pep_neu_conf_14
Eopt -893.914740
C 0.708890 -1.681544 0.408828
H 0.051458 -2.509459 0.138127
H 0.758273 -1.612052 1.499173
S 2.383474 -2.007600 -0.266679
H 2.558882 -3.180903 0.364403
C 0.142053 -0.386677 -0.173310
H 0.156182 -0.438861 -1.267173
C -1.315837 -0.241774 0.295930
O -1.592237 0.247028 1.402483
N 0.933603 0.750115 0.247790
N -2.243801 -0.707481 -0.543835
H -1.949721 -1.082178 -1.436413
C -3.657815 -0.715062 -0.211958
H -3.841066 -1.302741 0.692178
H -4.022979 0.303426 -0.051458
H -4.202131 -1.161312 -1.044018
C 0.707904 1.989633 -0.230432
O -0.137661 2.194435 -1.118768
C 1.526170 3.106575 0.354718
H 2.269643 2.758087 1.075143
H 2.030249 3.637816 -0.457836
H 0.850020 3.811190 0.849176
H 1.547986 0.641528 1.045501
23
cysteine_pep_neu_conf_15
Eopt -893.914610
C -0.600036 -1.425797 -0.530682
H -0.122741 -2.408381 -0.582276
H -0.430569 -0.920924 -1.482191
S -2.381399 -1.752999 -0.329479
H -2.804709 -0.489678 -0.516251
C 0.028210 -0.654604 0.641865
H -0.116950 -1.243638 1.547938
C 1.551024 -0.595026 0.446909
O 2.273183 -1.426108 1.027495
N -0.603599 0.632588 0.919526
N 2.035708 0.333696 -0.378914
H 1.370694 0.927445 -0.869670
C 3.454230 0.445194 -0.667400
H 4.021745 0.633838 0.248346
H 3.832682 -0.469910 -1.133214
H 3.599154 1.280229 -1.352904
C -0.877376 1.612628 0.036010
O -0.450360 1.591815 -1.133882
C -1.727039 2.749942 0.530977
H -1.961122 2.681096 1.595398
H -1.207768 3.691570 0.331646
H -2.661133 2.753123 -0.040133
H -0.998319 0.729551 1.845718
23
cysteine_pep_neu_conf_16
Eopt -893.912878
C 0.634460 -1.475410 -0.371520
H 0.322395 -1.142902 -1.361296
H 0.208372 -2.466860 -0.197400
S 2.443914 -1.712332 -0.359884
H 2.780061 -0.493171 -0.819557
C 0.133970 -0.533412 0.734870
H 0.500689 -0.909449 1.690156
C 0.711872 0.886515 0.617297
O 1.512172 1.305083 1.473460
N -1.325093 -0.553288 0.850577
N 0.339265 1.612863 -0.438445
H -0.358661 1.215166 -1.063720
C 0.812085 2.969530 -0.647061
H 0.525203 3.616814 0.187420
H 1.901088 2.988677 -0.747951
H 0.363415 3.351201 -1.564270
C -2.222967 -0.338168 -0.131074

O -1.890905 0.012485 -1.279005
C -3.670773 -0.535177 0.226098
H -4.081819 -1.317220 -0.419914
H -3.825735 -0.815785 1.270086
H -4.211845 0.392205 0.016089
H -1.687015 -0.822214 1.756263
23
cysteine_pep_neu_conf_17
Eopt -893.916999
C -0.086340 1.532621 -0.496493
H -0.113339 1.456518 -1.586399
H 0.758311 2.168974 -0.220402
S -1.577141 2.401369 0.095087
H -2.481402 1.643434 -0.552455
C 0.126616 0.145888 0.125821
H 0.204000 0.238798 1.210729
C -1.005124 -0.806330 -0.254018
O -1.222651 -1.072980 -1.449206
N 1.369299 -0.428486 -0.362776
N -1.724155 -1.320040 0.744780
H -1.499676 -1.057913 1.695518
C -2.839179 -2.221376 0.509593
H -3.605462 -1.735988 -0.101627
H -2.501542 -3.127652 -0.000843
H -3.269128 -2.492707 1.473389
C 2.558435 -0.225540 0.240933
O 2.656064 0.384689 1.319433
C 3.769162 -0.787233 -0.453098
H 4.445134 0.038826 -0.695006
H 4.290684 -1.457036 0.237060
H 3.525092 -1.330669 -1.368706
H 1.356856 -0.848667 -1.284912
23
cysteine_pep_neu_conf_18
Eopt -893.916680
C -0.863183 -1.705265 0.292547
H -0.924825 -1.759086 1.383401
H -0.397887 -2.624764 -0.068476
S -2.586624 -1.616837 -0.295896
H -2.308946 -1.694153 -1.610099
C -0.010030 -0.509701 -0.131983
H 0.062577 -0.483417 -1.221637
C -0.594854 0.807137 0.391092
O -0.812061 0.965005 1.605274
N 1.334711 -0.680443 0.392130
N -0.827977 1.759209 -0.514308
H -0.609367 1.574197 -1.484272
C -1.363000 3.059614 -0.150025
H -1.475888 3.650725 -1.058560
H -2.339043 2.953406 0.331897
H -0.686123 3.579440 0.534166
C 2.442082 -0.250387 -0.247910
O 2.392849 0.276095 -1.372573
C 3.751218 -0.452126 0.464976
H 4.403111 -1.064065 -0.165788
H 4.230113 0.522906 0.596760
H 3.639692 -0.932036 1.439822
H 1.425739 -1.029554 1.338581
23
cysteine_pep_neu_conf_19
Eopt -893.917270
C -0.078813 1.282694 0.923953
H 0.850302 1.577857 1.415205
H -0.893777 1.455313 1.631216
S -0.398771 2.392223 -0.489276
H 0.735590 2.124485 -1.160941
C 0.001040 -0.212076 0.568878
H 0.116144 -0.756749 1.512619
C -1.266128 -0.711505 -0.128978
O -1.252128 -1.067932 -1.318262
N 1.131342 -0.515804 -0.280278
N -2.372701 -0.726623 0.617876
H -2.313998 -0.441199 1.587033
C -3.662786 -1.126440 0.082135
H -3.636498 -2.165139 -0.259495
H -4.405743 -1.030291 0.873610
H -3.948595 -0.485468 -0.756745
C 2.393056 -0.617091 0.174702
O 2.674691 -0.463354 1.377171
C 3.453658 -0.933133 -0.844317
H 4.208576 -0.141400 -0.821864
H 3.942082 -1.871161 -0.562862
H 3.057609 -1.023811 -1.858323
H 0.937481 -0.665423 -1.263724
23
cysteine_pep_neu_conf_2 Eopt
-893.916888
C -0.858603 -1.713583 0.248708
H -0.889795 -1.816359 1.336577
H -0.411301 -2.620303 -0.165606
S -2.560736 -1.655189 -0.403523
H -3.019883 -0.688358 0.412148
C -0.001738 -0.513391 -0.156306
H 0.081449 -0.475272 -1.244133
C -0.598105 0.794477 0.376156
O -0.861360 0.922348 1.585150
N 1.336304 -0.685064 0.384882
N -0.803717 1.765822 -0.514832
H -0.548008 1.604137 -1.480013
C -1.348508 3.058877 -0.138251
H -1.425458 3.672850 -1.035230
H -2.342169 2.943779 0.303888
H -0.696291 3.558478 0.583843
C 2.451370 -0.246684 -0.235840
O 2.416159 0.293010 -1.354757
C 3.751262 -0.455079 0.492048
H 4.221030 0.520183 0.652395
H 3.628800 -0.957161 1.454398
H 4.416916 -1.048248 -0.142202
H 1.415912 -1.046567 1.327732

23
cysteine_pep_neu_conf_20
Eopt -893.911409
C -0.602352 -1.428878 -0.527952
H -0.148717 -2.422306 -0.551823
H -0.415505 -0.931757 -1.479962
S -2.409915 -1.603589 -0.273095
H -2.581573 -2.573729 -1.186827
C 0.024148 -0.656464 0.639820
H -0.122951 -1.239751 1.549896
C 1.548360 -0.603860 0.448105
O 2.266531 -1.430063 1.039738
N -0.605253 0.634504 0.911501
N 2.036420 0.317568 -0.383454
H 1.373474 0.908600 -0.880453
C 3.455963 0.425452 -0.668311
H 3.835190 -0.494591 -1.123621
H 3.603427 1.253332 -1.361873
H 4.020593 0.623061 0.247320
C -0.869009 1.615210 0.024624
O -0.441598 1.587991 -1.144360
C -1.705082 2.763090 0.518113
H -1.175060 3.698129 0.316086
H -2.639791 2.775884 -0.051802
H -1.938719 2.699302 1.582955
H -0.981522 0.746012 1.843869
23
cysteine_pep_neu_conf_21
Eopt -893.912977
C 0.626156 -1.485168 -0.372636
H 0.313915 -1.147544 -1.361944
H 0.197051 -2.474972 -0.201135
S 2.443409 -1.608409 -0.452887
H 2.627575 -2.115896 0.779677
C 0.133502 -0.536720 0.730631
H 0.498868 -0.913320 1.687066
C 0.709087 0.884289 0.619385
O 1.455352 1.325579 1.512081
N -1.324500 -0.552707 0.850543
N 0.380032 1.592905 -0.462435
H -0.297757 1.187436 -1.103679
C 0.841665 2.954529 -0.662509
H 1.934325 2.990683 -0.695099
H 0.446159 3.314121 -1.612533
H 0.492388 3.610229 0.141296
C -2.219680 -0.333498 -0.133191
O -1.882501 0.008417 -1.281876
C -3.669573 -0.514871 0.224162
H -4.091059 -1.288785 -0.424804
H -3.826821 -0.797830 1.267221
H -4.199431 0.420136 0.019141
H -1.688229 -0.814449 1.757523
23
cysteine_pep_neu_conf_23
Eopt -893.912754
C 0.494084 -1.499755 -0.356002
H 0.486015 -1.667040 -1.436383
H -0.185768 -2.218050 0.107655
S 2.204616 -1.825859 0.189316
H 1.985299 -1.656839 1.506526
C 0.007623 -0.080938 -0.039004
H -0.002883 0.059905 1.041453
C 0.877977 0.968290 -0.730293
O 0.971597 0.957072 -1.971804
N -1.354976 0.100446 -0.509018
N 1.496843 1.883037 0.021490
H 2.058305 2.554213 -0.490358
C 1.461869 2.004551 1.474811
H 0.442940 2.165410 1.835545
H 1.886711 1.122220 1.960938
H 2.064892 2.871126 1.745627
C -2.435451 -0.131411 0.265511
O -2.335379 -0.432500 1.467126
C -3.777678 0.001624 -0.400108
H -3.709350 0.344525 -1.434893
H -4.275421 -0.973000 -0.377366
H -4.387790 0.704298 0.174749
H -1.490154 0.251863 -1.501922
23
cysteine_pep_neu_conf_24
Eopt -893.914047
C -1.320397 -1.056954 0.608154
H -2.326094 -0.640679 0.508902
H -1.130270 -1.209555 1.674693
S -1.380893 -2.668288 -0.244878
H -0.186673 -3.127196 0.168705
C -0.304979 -0.070613 0.022628
H -0.459234 0.009407 -1.055372
C -0.524282 1.300773 0.681165
O 0.043135 1.587702 1.749434
N 1.046892 -0.533598 0.247310
N -1.375904 2.141028 0.080995
H -1.522014 3.023500 0.557110
C -2.058243 1.938978 -1.191449
H -2.664383 1.029613 -1.180348
H -2.720674 2.789974 -1.349167
H -1.347667 1.891889 -2.021820
C 2.100754 0.045367 -0.362916
O 1.945029 0.935956 -1.216442
C 3.467951 -0.444640 0.025054
H 3.979323 -0.806067 -0.872216
H 4.041393 0.398498 0.422350
H 3.439422 -1.243173 0.770042
H 1.224120 -1.142338 1.037288
23
cysteine_pep_neu_conf_25
Eopt -893.912605
C -1.013573 -1.561067 -0.126776
H -1.004011 -2.047975 0.852844
H -0.667368 -2.282190 -0.870012

S -2.758099 -1.135856 -0.447825
H -2.579890 -0.670224 -1.697977
C -0.073435 -0.352826 -0.126291
H -0.048967 0.083987 -1.124714
C -0.511476 0.678821 0.921246
O -0.525988 0.355957 2.123657
N 1.272014 -0.805225 0.182775
N -0.848090 1.906866 0.514211
H -1.143652 2.536996 1.251272
C -0.912261 2.401814 -0.856307
H -1.220963 3.446204 -0.812804
H 0.064210 2.349481 -1.343265
H -1.646746 1.850156 -1.449439
C 2.384446 -0.231662 -0.322168
O 2.335528 0.684303 -1.160315
C 3.699301 -0.768084 0.174265
H 4.234422 0.040464 0.682079
H 3.586772 -1.609724 0.861461
H 4.299098 -1.079877 -0.685794
H 1.374885 -1.479145 0.932101
23
cysteine_pep_neu_conf_26
Eopt -893.915840
C 1.066366 -1.479837 0.896436
H 0.558541 -2.407445 1.170642
H 1.758158 -1.222900 1.699531
S 2.010652 -1.860484 -0.622110
H 2.731227 -0.724247 -0.658474
C 0.019876 -0.368720 0.747296
H -0.464771 -0.276559 1.723289
C 0.705877 0.980746 0.496534
O 1.539810 1.397003 1.320135
N -0.977395 -0.724162 -0.240933
N 0.384739 1.652951 -0.608979
H -0.312669 1.266218 -1.230712
C 0.980902 2.937824 -0.931030
H 0.561208 3.282652 -1.875793
H 0.761937 3.673027 -0.151480
H 2.065697 2.844230 -1.033625
C -2.298950 -0.430913 -0.204715
O -3.034796 -0.761775 -1.149636
C -2.833943 0.285466 1.004842
H -2.743523 -0.349915 1.892834
H -2.281554 1.211136 1.195793
H -3.886226 0.519447 0.840328
H -0.650732 -1.178644 -1.089357
23
cysteine_pep_neu_conf_3 Eopt
-893.916757
C 0.766763 -1.661194 0.399498
H 0.126773 -2.508691 0.146843
H 0.815099 -1.592108 1.490807
S 2.470487 -2.012532 -0.154339
H 2.184471 -2.143369 -1.462384
C 0.158312 -0.380986 -0.182902
H 0.167885 -0.440200 -1.276727
C -1.298896 -0.287279 0.298358
O -1.579108 0.147323 1.426850
N 0.910643 0.782214 0.232951
N -2.222637 -0.746702 -0.549746
H -1.931348 -1.060698 -1.466207
C -3.633432 -0.793755 -0.207642
H -3.796747 -1.416248 0.676711
H -4.017641 0.210852 -0.008024
H -4.176174 -1.221767 -1.050238
C 0.632673 2.016613 -0.224037
O -0.263785 2.207552 -1.065626
C 1.454365 3.150095 0.324245
H 1.941229 3.666343 -0.508556
H 0.783927 3.861948 0.815530
H 2.213467 2.821247 1.037531
H 1.609665 0.674660 0.956795
23
cysteine_pep_neu_conf_4 Eopt
-893.916899
C -0.042064 1.660922 0.398827
H 0.719054 2.330502 -0.011668
H 0.038176 1.686439 1.489033
S -1.642878 2.364320 -0.115760
H -2.424532 1.463445 0.507167
C 0.201578 0.234990 -0.116039
H 0.079501 0.209231 -1.199966
C 1.622817 -0.178982 0.270030
O 1.892551 -0.505372 1.438610
N -0.742769 -0.702153 0.455529
N 2.539695 -0.131216 -0.699147
H 2.251688 0.129831 -1.633294
C 3.940513 -0.427827 -0.452276
H 4.061733 -1.455170 -0.097326
H 4.486332 -0.306876 -1.387686
H 4.354349 0.255154 0.295114
C -1.681557 -1.364966 -0.250695
O -1.787411 -1.264059 -1.485038
C -2.610147 -2.244688 0.541873
H -2.552613 -3.263287 0.146892
H -2.380003 -2.257533 1.609561
H -3.634355 -1.886177 0.399460
H -0.719722 -0.822339 1.461308
23
cysteine_pep_neu_conf_5 Eopt
-893.916805
C 0.089109 1.538225 0.517628
H 0.153318 1.446678 1.605456
H -0.772669 2.165063 0.277750
S 1.623878 2.373893 -0.005526
H 1.309170 2.481276 -1.309299
C -0.115036 0.153167 -0.109086
H -0.192377 0.249346 -1.194308
C 1.016392 -0.801747 0.265524
O 1.244149 -1.064346 1.459370

N -1.359481 -0.425427 0.370393
N 1.711603 -1.337401 -0.738864
H 1.482167 -1.075502 -1.688359
C 2.814564 -2.255274 -0.512112
H 2.470875 -3.147521 0.018590
H 3.219602 -2.549302 -1.480080
H 3.602444 -1.775760 0.075947
C -2.545003 -0.229619 -0.242839
O -2.637121 0.374967 -1.325002
C -3.758641 -0.791541 0.445909
H -3.518403 -1.334462 1.362878
H -4.435636 0.034584 0.684858
H -4.277200 -1.461568 -0.246183
H -1.352716 -0.839553 1.295369
23
cysteine_pep_neu_conf_6 Eopt
-893.916757
C 0.766448 -1.660949 0.400258
H 0.125931 -2.508309 0.148490
H 0.815284 -1.591176 1.491503
S 2.469739 -2.013551 -0.154116
H 2.182972 -2.145504 -1.461883
C 0.158447 -0.380839 -0.182762
H 0.168146 -0.440552 -1.276558
C -1.298829 -0.286470 0.298182
O -1.579221 0.149968 1.425915
N 0.911072 0.782289 0.232659
N -2.222396 -0.747297 -0.549344
H -1.930832 -1.063339 -1.465013
C -3.633207 -0.794269 -0.207297
H -4.017712 0.210519 -0.009190
H -4.175746 -1.223645 -1.049329
H -3.796453 -1.415523 0.677945
C 0.633175 2.016632 -0.224583
O -0.263113 2.207414 -1.066375
C 1.454451 3.150172 0.324185
H 1.937153 3.670181 -0.508655
H 0.784203 3.858924 0.820258
H 2.216742 2.820667 1.033766
H 1.609518 0.674908 0.957092
23
cysteine_pep_neu_conf_7 Eopt
-893.916899
C -0.042063 1.660893 0.398720
H 0.718931 2.330513 -0.011952
H 0.038354 1.686502 1.488912
S -1.643020 2.364077 -0.115659
H -2.424476 1.463137 0.507432
C 0.201659 0.234944 -0.116082
H 0.079620 0.209141 -1.200007
C 1.622908 -0.178935 0.270029
O 1.892696 -0.505173 1.438627
N -0.742653 -0.702238 0.455500
N 2.539774 -0.131210 -0.699186
H 2.251696 0.129510 -1.633401
C 3.940580 -0.427814 -0.452315
H 4.354464 0.255289 0.294937
H 4.061802 -1.455098 -0.097184
H 4.486380 -0.307044 -1.387762
C -1.681630 -1.364845 -0.250697
O -1.787700 -1.263678 -1.484989
C -2.610130 -2.244661 0.541871
H -2.552540 -3.263223 0.146788
H -2.379896 -2.257625 1.609536
H -3.634368 -1.886207 0.399562
H -0.719412 -0.822670 1.461244
23
cysteine_pep_neu_conf_8 Eopt
-893.913885
C -0.436933 1.433656 -0.686437
H -1.392351 1.897505 -0.425251
H -0.384409 1.363942 -1.776657
S 0.844327 2.579313 -0.080988
H 1.906170 1.888454 -0.534651
C -0.354996 0.028153 -0.068153
H -0.361456 0.102936 1.019362
C -1.558260 -0.776908 -0.564902
O -1.494405 -1.393750 -1.643449
N 0.866193 -0.645785 -0.448914
N -2.672058 -0.745611 0.175023
H -3.460776 -1.256233 -0.204860
C -2.849608 -0.084749 1.462837
H -2.630626 0.983964 1.397438
H -3.893403 -0.203568 1.752822
H -2.220629 -0.538231 2.233944
C 1.846992 -0.985880 0.412016
O 1.768973 -0.774044 1.634458
C 3.061640 -1.640841 -0.188495
H 2.971354 -1.808356 -1.264151
H 3.930376 -1.003315 0.003506
H 3.229876 -2.597682 0.314342
H 0.988138 -0.856897 -1.432074
23
cysteine_pep_neu_ox_conf_1
Eopt -893.683508
C 0.026366 -1.478691 0.983954
H -0.968120 -1.903226 1.141724
H 0.634496 -1.615847 1.879185
S 0.798708 -2.377812 -0.393168
H 1.936417 -2.754343 0.222682
C -0.094672 0.008730 0.632300
H -0.348975 0.542924 1.551311
C 1.251286 0.483479 0.093550
O 1.939310 -0.312394 -0.604486
N -1.111719 0.298258 -0.354476
N 1.648355 1.709877 0.344165
H 1.044569 2.301763 0.904018
C 2.891629 2.257116 -0.183463
H 2.997979 3.273862 0.190689
H 3.738540 1.654099 0.151905

H 2.865665 2.269951 -1.275690
C -2.400612 0.535802 -0.000039
O -2.754525 0.533030 1.185842
C -3.366788 0.795447 -1.120552
H -4.107349 -0.010516 -1.132132
H -3.892515 1.732836 -0.917702
H -2.884850 0.853322 -2.099002
H -0.873175 0.266830 -1.339465
23
cysteine_pep_neu_ox_conf_10
Eopt -893.680909
C -1.106828 -1.027832 -1.068213
H -0.557793 -1.933556 -1.336997
H -1.629429 -0.638655 -1.942897
S -2.339533 -1.471556 0.190356
H -3.442808 -1.024450 -0.440111
C -0.134571 0.019902 -0.517096
H 0.398506 0.434177 -1.375910
C -0.929485 1.112162 0.194485
O -2.035601 0.807480 0.725540
N 0.855413 -0.500920 0.395934
N -0.439461 2.330324 0.272481
H -0.996615 3.006234 0.785776
C 0.824751 2.783407 -0.305635
H 0.954771 3.826833 -0.023044
H 1.662928 2.203905 0.086471
H 0.800086 2.710526 -1.394977
C 2.129248 -0.762001 -0.000633
O 2.489586 -0.595981 -1.171931
C 3.069625 -1.254210 1.061756
H 3.859882 -0.508415 1.194490
H 2.577676 -1.429207 2.020775
H 3.535657 -2.181148 0.716330
H 0.609693 -0.647739 1.368636
23
cysteine_pep_neu_ox_conf_12
Eopt -893.684208
C -0.026234 -1.378260 0.516546
H -0.726342 -2.150460 0.190371
H -0.118423 -1.225217 1.594052
S 1.625407 -2.031928 0.157843
H 2.280209 -1.519589 1.218705
C -0.306576 -0.073039 -0.260106
H -0.227200 -0.275303 -1.332684
C -1.729852 0.400481 0.072522
O -1.908430 1.340252 0.859125
N 0.595839 0.998832 0.086917
N -2.714051 -0.267087 -0.524132
H -2.481536 -1.015941 -1.165106
C -4.113514 0.034801 -0.269296
H -4.348990 -0.097282 0.790250
H -4.343462 1.063565 -0.559523
H -4.722168 -0.648685 -0.860253
C 1.879575 1.008001 -0.242348
O 2.411565 0.007083 -0.812030
C 2.710573 2.210262 0.062719
H 3.505750 1.917488 0.754575
H 3.176696 2.556515 -0.864179
H 2.117540 3.016691 0.498367
H 0.199988 1.774684 0.613471
23
cysteine_pep_neu_ox_conf_13
Eopt -893.662257
C -0.502923 -1.554867 -0.672131
H -0.239320 -2.613515 -0.719282
H -0.294405 -1.075412 -1.629292
S -2.300082 -1.361668 -0.391877
H -2.363253 -2.017734 0.790183
C 0.087678 -0.834133 0.534952
H -0.008808 -1.485043 1.404270
C 1.594654 -0.524377 0.455590
O 2.349057 -1.054470 1.284924
N -0.815592 0.300715 0.823036
N 2.025113 0.272475 -0.520477
H 1.341672 0.695328 -1.139820
C 3.430814 0.609565 -0.667209
H 3.532843 1.279143 -1.520782
H 3.804181 1.110606 0.230567
H 4.025683 -0.290993 -0.842892
C -0.994156 1.436549 0.019408
O -0.531882 1.470820 -1.111442
C -1.787531 2.533789 0.651860
H -2.048273 3.273596 -0.104928
H -2.691996 2.139281 1.124449
H -1.177768 3.006059 1.430754
H -1.054511 0.423085 1.806211
23
cysteine_pep_neu_ox_conf_14
Eopt -893.685773
C -0.000885 -1.313093 0.614766
H -0.754711 -2.085009 0.435614
H 0.014476 -1.063795 1.678292
S 1.604876 -2.048880 0.232850
H 1.356759 -2.359242 -1.056613
C -0.295773 -0.068548 -0.245067
H -0.220249 -0.333633 -1.304850
C -1.721398 0.422512 0.052560
O -1.905016 1.419693 0.763605
N 0.601969 1.026521 0.040135
N -2.702447 -0.292620 -0.491909
H -2.467159 -1.085322 -1.076497
C -4.103220 0.022388 -0.261421
H -4.335887 -0.018896 0.806069
H -4.339943 1.020814 -0.639207
H -4.708725 -0.713568 -0.789299
C 1.901923 1.005928 -0.217235
O 2.462708 -0.029979 -0.686709
C 2.720585 2.226842 0.047699
H 2.104695 3.071964 0.361516
H 3.452082 1.992048 0.826639

H 3.265799 2.487684 -0.863718
H 0.189267 1.837846 0.495884
23
cysteine_pep_neu_ox_conf_15
Eopt -893.662257
C -0.628062 -1.344400 -0.585119
H -0.063232 -2.243392 -0.844085
H -0.614471 -0.649387 -1.445334
S -2.311555 -1.837288 -0.345434
H -2.761426 -0.673089 0.193544
C -0.024876 -0.611875 0.653344
H -0.178469 -1.224963 1.540772
C 1.516578 -0.577931 0.474475
O 2.203307 -1.323885 1.183971
N -0.653252 0.659368 0.917688
N 2.015958 0.241917 -0.446112
H 1.371114 0.816726 -0.982779
C 3.446232 0.342670 -0.684757
H 3.967341 0.665057 0.221013
H 3.852600 -0.621105 -1.003933
H 3.607718 1.078542 -1.471953
C -0.863849 1.653879 0.014168
O -0.427705 1.573064 -1.143639
C -1.662377 2.832607 0.482918
H -1.869342 2.808591 1.554390
H -1.115602 3.745967 0.234037
H -2.609419 2.844857 -0.066188
H -1.047575 0.776659 1.843595
23
cysteine_pep_neu_ox_conf_16
Eopt -893.686314
C 0.797787 -1.638497 0.109772
H 0.344297 -1.823434 -0.863348
H 0.718802 -2.529736 0.735927
S 2.560997 -1.296081 -0.117340
H 2.580339 -1.183192 -1.459289
C 0.125914 -0.457142 0.836197
H 0.467313 -0.439769 1.876125
C 0.608019 0.866777 0.239434
O 1.764196 0.917766 -0.263954
N -1.304799 -0.592343 0.872491
N -0.144590 1.937738 0.342255
H -1.073994 1.832629 0.733525
C 0.276612 3.243744 -0.148526
H 1.182084 3.567528 0.370041
H 0.470298 3.199621 -1.223101
H -0.527005 3.951886 0.046677
C -2.080005 -0.579447 -0.245318
O -1.583727 -0.380574 -1.360638
C -3.550780 -0.803222 -0.048167
H -3.838785 -1.708618 -0.591411
H -3.829853 -0.909629 1.002007
H -4.093685 0.039406 -0.485980
H -1.739058 -0.767972 1.769434
23
cysteine_pep_neu_ox_conf_17
Eopt -893.664338
C 0.023319 1.472465 0.580342
H 0.068337 1.241022 1.645731
H -0.868138 2.039811 0.305990
S 1.435693 2.337640 0.021875
H 2.347788 1.759370 0.834053
C -0.170843 0.009639 -0.194560
H -0.234281 0.233748 -1.259010
C 1.020319 -0.868929 0.200795
O 1.112456 -1.301021 1.354124
N -1.361828 -0.548128 0.264373
N 1.912007 -1.081597 -0.763181
H 1.737188 -0.719646 -1.692662
C 3.133801 -1.833848 -0.518363
H 3.714566 -1.364357 0.279815
H 2.898869 -2.862768 -0.233408
H 3.719518 -1.837935 -1.436686
C -2.602235 -0.246756 -0.291807
O -2.667558 0.478206 -1.275329
C -3.776271 -0.847550 0.411537
H -3.947949 -0.294789 1.342614
H -4.660097 -0.768288 -0.221814
H -3.587961 -1.893472 0.670180
H -1.337906 -1.054471 1.148822
23
cysteine_pep_neu_ox_conf_18
Eopt -893.688920
C -0.863394 -1.585640 0.579497
H -0.919142 -1.457809 1.663665
H -0.445104 -2.569548 0.350680
S -2.561624 -1.526106 -0.024550
H -2.290407 -1.634494 -1.343437
C -0.016434 -0.492384 -0.078373
H 0.125659 -0.725539 -1.140507
C -0.794249 0.823683 -0.045862
O -2.048340 0.784159 -0.186598
N 1.282038 -0.408740 0.520502
N -0.143554 1.956025 0.079614
H 0.865415 1.914681 0.176186
C -0.804908 3.254532 0.054229
H -0.048761 4.020568 0.217586
H -1.286089 3.416755 -0.913083
H -1.555775 3.307822 0.845964
C 2.437687 -0.466697 -0.197072
O 2.434207 -0.595312 -1.426344
C 3.711244 -0.354092 0.590607
H 4.347690 -1.210494 0.350232
H 4.234308 0.553609 0.273768
H 3.545433 -0.315209 1.669179
H 1.336750 -0.300821 1.527130
23
cysteine_pep_neu_ox_conf_19
Eopt -893.685728
C -0.003077 1.483293 0.987636

H 0.998326 1.894879 1.144773
H -0.602251 1.641374 1.887282
S -0.817999 2.423530 -0.322747
H -0.021240 2.055446 -1.350923
C 0.081978 -0.005470 0.644046
H 0.327430 -0.543472 1.563445
C -1.275809 -0.455535 0.111186
O -1.964343 0.359343 -0.564189
N 1.089142 -0.317957 -0.346427
N -1.678467 -1.684828 0.337919
H -1.072980 -2.293299 0.877874
C -2.927831 -2.213011 -0.194788
H -2.908900 -2.199410 -1.287169
H -3.037052 -3.238018 0.155222
H -3.769419 -1.613925 0.160421
C 2.377174 -0.569738 0.002468
O 2.738724 -0.557116 1.185694
C 3.330172 -0.865165 -1.120534
H 4.130987 -0.119769 -1.101583
H 3.780117 -1.846832 -0.944583
H 2.853300 -0.857555 -2.103120
H 0.844249 -0.300454 -1.330246
23
cysteine_pep_neu_ox_conf_2
Eopt -893.687034
C -0.865459 -1.590222 0.564449
H -0.932770 -1.454436 1.645304
H -0.437859 -2.568834 0.335823
S -2.528661 -1.539782 -0.152512
H -3.219920 -1.184943 0.948069
C -0.009856 -0.496658 -0.091788
H 0.138953 -0.726619 -1.153080
C -0.787158 0.819533 -0.060757
O -2.037759 0.780098 -0.226659
N 1.283664 -0.419219 0.516307
N -0.139981 1.950377 0.092198
H 0.866460 1.907305 0.211288
C -0.801215 3.249020 0.070238
H -0.046474 4.013967 0.244744
H -1.273931 3.417267 -0.900207
H -1.558842 3.297583 0.855814
C 2.444659 -0.463930 -0.194558
O 2.449191 -0.570573 -1.425682
C 3.712986 -0.367807 0.603555
H 4.254456 0.528742 0.286428
H 3.539157 -0.321507 1.680545
H 4.336447 -1.236544 0.372767
H 1.331926 -0.329390 1.525068
23
cysteine_pep_neu_ox_conf_20
Eopt -893.661082
C -0.635743 -1.371097 -0.533597
H -0.170743 -2.345622 -0.739856
H -0.562441 -0.778211 -1.463210
S -2.377953 -1.633608 -0.249029
H -2.608021 -2.404336 -1.341817
C 0.004757 -0.641795 0.653363
H -0.130024 -1.243062 1.553220
C 1.537311 -0.604399 0.450667
O 2.238182 -1.411961 1.078385
N -0.639990 0.633417 0.921895
N 2.028176 0.285047 -0.410287
H 1.375468 0.875656 -0.919317
C 3.452423 0.385416 -0.679663
H 3.836221 -0.550970 -1.094938
H 3.606351 1.187153 -1.401450
H 4.002322 0.617528 0.236572
C -0.863023 1.622326 0.020314
O -0.458919 1.532861 -1.150016
C -1.633647 2.815248 0.502423
H -1.044203 3.714589 0.302747
H -2.559189 2.882614 -0.077818
H -1.878250 2.766202 1.565094
H -0.944116 0.785545 1.876003
23
cysteine_pep_neu_ox_conf_21
Eopt -893.688221
C 0.808937 -1.637720 0.095456
H 0.344089 -1.838352 -0.871087
H 0.752240 -2.533272 0.720225
S 2.543143 -1.293721 -0.249456
H 2.944276 -1.045266 1.016122
C 0.137500 -0.462070 0.820276
H 0.491636 -0.440116 1.856351
C 0.602242 0.865166 0.215390
O 1.739292 0.920464 -0.330347
N -1.293146 -0.603623 0.871316
N -0.143791 1.936148 0.358465
H -1.057570 1.827851 0.784250
C 0.261633 3.247371 -0.131488
H 0.431725 3.211704 -1.210319
H -0.540256 3.950818 0.086512
H 1.177087 3.571357 0.369226
C -2.078116 -0.583589 -0.239084
O -1.592593 -0.370834 -1.356710
C -3.546197 -0.815602 -0.031448
H -3.840202 -1.703331 -0.599833
H -3.812420 -0.955139 1.018245
H -4.095013 0.040164 -0.435432
H -1.716905 -0.792917 1.770416
23
cysteine_pep_neu_ox_conf_23
Eopt -893.682878
C -1.224831 -1.348100 0.372848
H -1.229634 -1.413914 1.463953
H -0.955638 -2.321852 -0.045704
S -2.918775 -0.958446 -0.099339
H -2.711040 -0.786803 -1.423583
C -0.233752 -0.284940 -0.114874
H -0.073220 -0.393929 -1.194560

C -0.830294 1.110257 0.093298
O -2.080812 1.205089 0.267489
N 1.031493 -0.473871 0.535737
N -0.085238 2.195890 0.057296
H -0.593441 3.063584 0.196859
C 1.332933 2.315358 -0.281893
H 1.968720 2.066723 0.569336
H 1.581273 1.683861 -1.135925
H 1.502209 3.355156 -0.560502
C 2.159233 -0.846514 -0.122101
O 2.158997 -1.076594 -1.337546
C 3.408837 -0.927616 0.706495
H 3.212388 -0.888169 1.780096
H 3.939695 -1.851292 0.462733
H 4.052184 -0.084233 0.432740
H 1.084882 -0.286760 1.530575
23
cysteine_pep_neu_ox_conf_24
Eopt -893.680793
C 0.240512 1.166495 0.836541
H 1.082466 1.853045 0.723464
H 0.140379 0.877736 1.884618
S -1.230126 2.098944 0.335681
H -2.094125 1.614638 1.250044
C 0.448360 -0.074056 -0.061903
H 0.503160 0.242363 -1.106604
C 1.748491 -0.764724 0.369429
O 1.701760 -1.661094 1.225901
N -0.618642 -1.040126 0.070991
N 2.883544 -0.343685 -0.188867
H 3.719999 -0.812417 0.140755
C 3.034107 0.625379 -1.270020
H 2.627753 1.601339 -0.994524
H 4.100816 0.739385 -1.460015
H 2.551818 0.273646 -2.185859
C -1.842397 -0.832414 -0.396140
O -2.157164 0.279428 -0.918210
C -2.858813 -1.921834 -0.305717
H -3.671723 -1.586843 0.345417
H -3.271863 -2.098180 -1.302832
H -2.428391 -2.846339 0.082862
H -0.410918 -1.894539 0.582240
23
cysteine_pep_neu_ox_conf_26
Eopt -893.680452
C -0.034363 -1.454604 0.988140
H -1.033572 -1.879793 1.111254
H 0.528617 -1.552833 1.917411
S 0.811914 -2.396096 -0.314157
H 1.910437 -2.756515 0.378165
C -0.146196 0.018953 0.575577
H -0.441683 0.584855 1.461413
C 1.231516 0.484779 0.105311
O 1.966315 -0.332490 -0.514695
N -1.090361 0.265435 -0.493199
N 1.600683 1.725020 0.328109
H 0.956510 2.333890 0.820387
C 2.866847 2.266334 -0.149083
H 2.932852 3.303800 0.173886
H 3.699911 1.699121 0.272268
H 2.910649 2.219006 -1.239743
C -2.413950 0.543864 -0.340325
O -3.128240 0.671523 -1.343364
C -2.951591 0.701300 1.053633
H -2.730614 -0.177348 1.667914
H -2.501036 1.574891 1.537537
H -4.031339 0.842161 0.999297
H -0.781788 0.137092 -1.452680
23
cysteine_pep_neu_ox_conf_3
Eopt -893.670459
C -0.041486 -1.861902 0.026979
H -0.444748 -2.548076 -0.719148
H -0.414321 -2.124682 1.020474
S 1.778734 -1.996593 0.124961
H 2.044268 -1.630254 -1.150238
C -0.236415 -0.381190 -0.303279
H -0.144748 -0.219882 -1.379337
C -1.562086 0.167914 0.227487
O -1.616211 0.683425 1.353201
N 0.895957 0.285265 0.346433
N -2.614860 0.011855 -0.572013
H -2.479600 -0.400346 -1.486748
C -3.954051 0.409273 -0.168163
H -4.258588 -0.128804 0.733685
H -3.993554 1.484020 0.028669
H -4.640282 0.168029 -0.979104
C 1.612739 1.322853 -0.288190
O 1.486979 1.497082 -1.486947
C 2.507149 2.104816 0.616959
H 3.102337 2.804337 0.030349
H 1.896182 2.653248 1.342726
H 3.162244 1.427702 1.175149
H 0.822137 0.365715 1.362419
23
cysteine_pep_neu_ox_conf_4
Eopt -893.684208
C -0.026165 1.378331 -0.516672
H -0.726426 2.150433 -0.190591
H -0.118222 1.225310 -1.594204
S 1.625342 2.032050 -0.157640
H 2.280359 1.519921 -1.218465
C -0.306482 0.073042 0.259804
H -0.226921 0.275097 1.332411
C -1.729827 -0.400346 -0.072701
O -1.908506 -1.340038 -0.859402
N 0.595927 -0.998747 -0.087586
N -2.713939 0.267001 0.524295
H -2.481374 1.015588 1.165550
C -4.113441 -0.034956 0.269711

H -4.344169 -1.062691 0.563012
H -4.722044 0.650764 0.858130
H -4.348195 0.093934 -0.790373
C 1.879576 -1.008008 0.242046
O 2.411415 -0.007125 0.811910
C 2.710483 -2.210547 -0.062229
H 3.171067 -2.560129 0.866215
H 2.118546 -3.014959 -0.503072
H 3.509782 -1.917080 -0.748969
H 0.200080 -1.774556 -0.614189
23
cysteine_pep_neu_ox_conf_5
Eopt -893.673670
C 0.158826 1.261998 0.679850
H 0.129011 1.011021 1.743008
H -0.488396 2.121075 0.488634
S 1.878444 1.738787 0.325870
H 1.692552 2.160470 -0.944217
C -0.276638 0.052261 -0.179709
H -0.355167 0.361401 -1.226582
C 0.815450 -0.990863 -0.041831
O 0.741312 -1.986484 0.644834
N -1.542813 -0.465607 0.250720
N 2.012264 -0.591193 -0.651713
H 1.912551 -0.237189 -1.603334
C 3.252707 -1.300009 -0.367631
H 3.209164 -2.305943 -0.797776
H 4.072491 -0.743052 -0.820568
H 3.393949 -1.367814 0.711341
C -2.716437 0.007908 -0.231315
O -2.751520 0.884683 -1.106602
C -3.966902 -0.585910 0.351740
H -3.766886 -1.403969 1.046980
H -4.514767 0.204318 0.874670
H -4.597200 -0.948249 -0.465312
H -1.548932 -1.132959 1.013701
23
cysteine_pep_neu_ox_conf_6
Eopt -893.670480
C -0.125248 -1.840374 0.094297
H -0.571265 -2.531532 -0.622420
H -0.499587 -2.047401 1.100023
S 1.686592 -2.063930 0.177141
H 1.953547 -1.752433 -1.112392
C -0.241841 -0.365093 -0.287254
H -0.124561 -0.249176 -1.367886
C -1.545236 0.296430 0.164496
O -1.536407 1.163733 1.048349
N 0.915065 0.265633 0.351453
N -2.648720 -0.135856 -0.444442
H -2.566330 -0.834389 -1.173111
C -3.967058 0.373093 -0.101310
H -4.027330 1.447916 -0.293208
H -4.702413 -0.142436 -0.718144
H -4.188323 0.187191 0.953132
C 1.673893 1.258791 -0.303134
O 1.542489 1.425198 -1.502591
C 2.615276 2.007554 0.581494
H 3.261156 2.643074 -0.024059
H 2.037780 2.624470 1.279332
H 3.217346 1.309191 1.171791
H 0.852719 0.366113 1.366020
20
maleimide Eopt -590.316008
C 2.986419 -0.644899 0.165521
C 2.986354 0.645095 -0.165549
H 3.821899 -1.310467 0.338225
H 3.821915 1.310563 -0.338252
N 0.765004 -0.000043 0.000025
C 1.569248 -1.102061 0.288167
O 1.163380 -2.208193 0.588775
C 1.569183 1.102107 -0.288181
O 1.163151 2.208150 -0.588809
C -0.661176 -0.000038 0.000004
C -1.346218 0.828332 0.886719
C -1.346220 -0.828416 -0.886684
C -2.739323 0.831303 0.876888
H -0.793660 1.461157 1.574570
C -2.739327 -0.831332 -0.876858
H -0.793689 -1.461297 -1.574506
C -3.436209 0.000023 -0.000019
H -3.278924 1.478531 1.561710
H -3.278947 -1.478560 -1.561665
H -4.522251 0.000030 -0.000037
2
n2 Eopt -109.489915
N 0.000000 0.000000 0.550499
N 0.000000 0.000000 -0.550499
23
s2_cation_trunc_conf_1 Eopt -1006.104141
C -1.582933 -0.218847 0.000625
C -0.725029 0.910767 -0.000033
C 0.675855 0.853596 -0.000011
C 1.264889 -0.394478 0.000264
C 0.449841 -1.538917 0.000816
C -0.932090 -1.459193 0.001162
H 1.254172 1.769976 -0.000545
H 0.916402 -2.518392 0.001051
H -1.507752 -2.375978 0.001744
C 2.746239 -0.560426 -0.000787
O 3.294820 -1.649321 -0.005625
O 3.392985 0.597093 0.004016
C 4.831175 0.536223 0.001519
H 5.180609 0.023710 -0.896951
H 5.183167 0.021026 0.897484
N -1.728153 3.177807 -0.002456
N -1.284626 2.163130 -0.001273

S -3.311010 -0.001284 0.000723
C -3.946401 -1.695158 0.000115
H -3.639618 -2.229709 0.901254
H -5.033401 -1.585060 -0.000082
H -3.639216 -2.229157 -0.901216
H 5.159537 1.574000 0.002629
23
s2_cation_trunc_conf_2 Eopt
-1006.104280
C -1.538269 -0.286428 -0.000117
C -0.893617 0.976998 0.000015
C 0.494147 1.164995 0.000042
C 1.295170 0.042069 -0.000033
C 0.696043 -1.230027 -0.000143
C -0.679508 -1.392737 -0.000196
H 0.908358 2.167473 0.000155
H 1.321485 -2.115682 -0.000233
H -1.086016 -2.396160 -0.000303
C 2.774008 0.230878 -0.000069
O 3.309259 1.326821 -0.000414
O 3.434500 -0.918341 0.000382
C 4.871890 -0.840497 0.000137
H 5.216522 -0.322736 0.897461
H 5.216290 -0.322129 -0.896919
N -2.285411 3.028666 0.000315
N -1.665724 2.111016 0.000164
S -3.277078 -0.373610 -0.000266
C -3.607729 -2.152180 0.000335
H -4.697346 -2.235307 0.000844
H -3.212146 -2.623970 -0.901006
H -3.211311 -2.623439 0.901592
H 5.212460 -1.874323 -0.000253
23
s2_cation_trunc_conf_3 Eopt
-1006.098158
C 1.442549 -0.692356 0.029625
C 0.961497 0.637158 0.018704
C -0.403111 0.983819 -0.000457
C -1.339439 -0.023973 0.003154
C -0.903468 -1.362279 0.030856
C 0.439112 -1.679698 0.041397
H -0.692641 2.029053 -0.012197
H -1.629107 -2.167733 0.037452
H 0.733738 -2.724287 0.050788
C -2.781475 0.354194 -0.019393
O -3.170582 1.509755 -0.048227
O -3.585188 -0.699561 -0.004878
C -4.999966 -0.435016 -0.025874
H -5.472826 -1.415329 -0.010376
H -5.263980 0.104640 -0.937512
N 2.380737 2.666089 0.100057
N 1.800308 1.724728 0.055084
S 3.068822 -1.318929 0.045125
C 4.219991 0.057142 -0.186630
H 5.190974 -0.432833 -0.299483
H 4.012580 0.609376 -1.105051
H 4.268374 0.706360 0.688667
H -5.283550 0.142396 0.856261
23
s2_cation_trunc_conf_4 Eopt
-1006.098057
C 1.519357 -0.663179 0.001272
C 0.835848 0.574890 0.000556
C -0.568451 0.705032 -0.000345
C -1.333901 -0.438162 -0.000060
C -0.692024 -1.690426 0.001322
C 0.682210 -1.796147 0.001935
H -1.010840 1.694642 -0.001138
H -1.290630 -2.595025 0.001689
H 1.134399 -2.783119 0.002550
C -2.823526 -0.385383 -0.001096
O -3.523976 -1.383351 -0.003580
O -3.296236 0.853497 0.000989
C -4.728114 1.000954 -0.000030
H -5.150350 0.541612 0.895932
H -5.148829 0.544484 -0.898159
N 1.914368 2.806060 0.002684
N 1.494575 1.781738 0.001602
S 3.221683 -1.037027 0.002091
C 4.162606 0.507140 -0.006717
H 3.992806 1.085803 -0.916124
H 4.001165 1.091545 0.900511
H 5.204041 0.173166 -0.010568
H -4.903618 2.075246 0.001556
23
s2_reduced_trunc_conf_1 Eopt
-1006.275715
C -1.608605 -0.182314 0.000105
C -0.759634 0.936881 0.000224
C 0.623431 0.817419 0.000160
C 1.200074 -0.450186 -0.000072
C 0.370706 -1.577069 -0.000304
C -1.010404 -1.449846 -0.000239
H 1.235914 1.712712 0.000369
H 0.811427 -2.568655 -0.000468
H -1.616351 -2.347682 -0.000415
C 2.673207 -0.639728 0.000001
O 3.217551 -1.734399 0.000048
O 3.347018 0.508190 0.000000
C 4.781460 0.418828 0.000117
H 5.125223 -0.101307 -0.896486
H 5.125084 -0.101115 0.896886
N -0.846991 3.279681 -0.001130
N -1.395681 2.235161 0.000839
S -3.351568 0.082224 0.000225
C -4.014135 -1.602344 -0.000175
H -3.720870 -2.150934 0.898008
H -5.100570 -1.481974 -0.000110
H -3.720954 -2.150468 -0.898669
H 5.131728 1.449772 0.000029

23
s2_reduced_trunc_conf_2 Eopt
-1006.275672
C -1.565290 -0.263749 0.000016
C -0.935404 0.992194 -0.000090
C 0.445673 1.125247 0.000035
C 1.245093 -0.014256 0.000201
C 0.636668 -1.275176 0.000240
C -0.745395 -1.400294 0.000179
H 0.892671 2.114324 0.000015
H 1.244274 -2.173286 0.000386
H -1.176239 -2.394079 0.000280
C 2.720796 0.155260 0.000503
O 3.280739 1.242065 0.002245
O 3.378316 -1.001939 -0.001269
C 4.813961 -0.931735 -0.000920
H 5.164451 -0.418124 0.896828
H 5.164733 -0.414556 -0.896504
N -1.452840 3.279171 -0.001054
N -1.799231 2.151322 -0.000222
S -3.327289 -0.318893 -0.000121
C -3.676995 -2.094875 0.000407
H -4.767460 -2.172577 0.000548
H -3.289498 -2.581395 -0.897709
H -3.289298 -2.580877 0.898718
H 5.150388 -1.967290 -0.002938
23
s2_reduced_trunc_conf_3 Eopt
-1006.270653
C 1.471697 -0.631543 0.239465
C 1.003640 0.688455 0.204461
C -0.350289 0.980712 0.097051
C -1.273729 -0.060500 0.042211
C -0.828570 -1.384750 0.091409
C 0.530388 -1.662895 0.179013
H -0.682592 2.013813 0.078384
H -1.537403 -2.204056 0.051382
H 0.866789 -2.694380 0.195038
C -2.719467 0.286114 -0.060976
O -3.137982 1.431903 -0.122171
O -3.509724 -0.782402 -0.078055
C -4.924109 -0.541348 -0.173075
H -5.382317 -1.529002 -0.173050
H -5.152674 -0.013595 -1.101358
N 1.788771 2.887355 0.063161
N 1.970149 1.758663 0.351708
S 3.196351 -1.043307 0.397599
C 3.882749 -0.339280 -1.134738
H 4.916163 -0.691417 -1.194399
H 3.329924 -0.706328 -2.002143
H 3.879374 0.751853 -1.114609
H -5.263531 0.038114 0.687970
23
s2_reduced_trunc_conf_4 Eopt
-1006.270616
C 1.546361 -0.594280 -0.225785
C 0.866659 0.630454 -0.222251
C -0.517926 0.700610 -0.119738
C -1.255496 -0.478589 -0.035465
C -0.596729 -1.710688 -0.049839
C 0.788743 -1.765393 -0.134731
H -1.008832 1.667682 -0.128706
H -1.169247 -2.629918 0.014558
H 1.291324 -2.726970 -0.124329
C -2.742157 -0.462395 0.064574
O -3.425368 -1.472138 0.135323
O -3.250576 0.765721 0.070484
C -4.681109 0.874599 0.166832
H -5.028528 0.426581 1.100097
H -5.152291 0.386118 -0.688584
N 1.289337 2.928569 -0.113353
N 1.644531 1.841158 -0.398727
S 3.314959 -0.720120 -0.382557
C 3.880319 0.120879 1.130146
H 3.702621 1.196698 1.085253
H 3.394502 -0.309834 2.008300
H 4.956849 -0.060006 1.193149
H -4.887251 1.943626 0.156813
