# Supplementary material for: TagC-RED: An Infrared-Triggered Retro-Ene Reaction for Deep-Tissue Bioconjugation
Source: J Am Chem Soc. 2026 May 11;148(19):19738–49. doi: 10.1021/jacs.6c01581 (PMC13195677; doi:10.1021/jacs.6c01581)
Supplement: Supplementary file 2 [file ja6c01581_si_002.pdf]

Supplementary Materials for

TagC-RED: An Infrared-Triggered Retro-Ene Reaction for Deep Tissue Bioconjugation

Sang Mi Suh,[1,§] Benjamin Ben-Zvi,[1,§] John M. Talbott,[2] Niket Manoj,[3] Brock M. Nelson,[1] Riley R. Hughes,[2] Graham C. Haug,[3] Shohei Koide,[4] Robert S. Paton,[3*] Monika Raj,[2*] and Tianning Diao[1*]

[1]Department of Chemistry, New York University, 100 Washington Square East, New York, NY 10003, United States. [2]Department of Chemistry, Emory University, 1515 Dickey Dr, Atlanta, GA 30322, United States. [3]Department of Chemistry, Colorado State University, 1301 Center Avenue Ft. Collins, CO 80523-1872, United States. [4]Department of Biochemistry and Molecular Pharmacology, New York University Grossman School of Medicine, and Perlmutter Cancer Center, New York University Langone Health, 522 1st Ave, Smilow Research Center, New York, NY 10016, United States.

[§]Authors contributed equally

*e-mail: diao@nyu.edu, monika.raj@emory.edu, and Robert.Paton@colstate.edu

Table of Contents

1. General Considerations....4
2. Synthetic Procedures....6
3. Synthetic Procedure for Peptide 2....18
4. Peptide Modification and Product Characterization....19
   4.1. General Procedures and Characterization Methods....19
   4.2. Stability Studies of 1 in Buffers....26
   4.3. Control Experiments at Elevated Temperatures in the Dark....29
   4.4. Reduction of Modified Peptide 3 with TCEP....31
   4.5. Biotinylation of Peptides 2 and 3....32
   4.6. Characterization of 3....33
5. Reaction and Stability of 1 with Amino Acids Containing Nucleophilic Side Chains....34
   5.1. Reaction with Oxytocin....38
6. Proteins Used in This Study....40
   6.1. Ub-K63C....40
   6.2. Trastuzumab-LALAPG-S239C....42
7. Procedures for Protein Modifications....44
   7.1. Reaction and Characterization of Ub-K63C-Biotin Conjugate....44
   7.2. Reaction and Characterization of BSA Conjugates....44
      7.2.1. General Procedure A for Irradiation Reactions....44
      7.2.2. General Western Blot Procedure B with BSA-Biotin Conjugates....45
      7.2.3. SDS-PAGE Fluorescence Procedure with BSA-Fluorescein Conjugate....46
      7.2.4. BSA Reactions with 660 nm LED Irradiation....48
      7.2.5. BSA Reactions with 1040 nm Laser Irradiation....50
      7.2.6. BSA Reactions with 1040 nm Laser Irradiation through Bacon Layer....52
   7.3. Reaction and Characterization of Traustuzumab-LALAPG-S239C-Biotin Conjugate....54
8. Mechanistic Studies....55
   8.1. UV-Vis Experiment....55
   8.2. Quantum Yield Experiment....55
      8.2.1. Determination of the Light Intensity at 660 nm....55
      8.2.2. Determination of the Quantum Yield....56
   8.3. Isolation and Irradiation of Theoretical Intermediate....57
   8.4. Reaction of 13 in Organic Solvent....58
9. Stability Test of 14 in Buffers and Glutathione....59

10. Cell Data 60
10.1. General Cell Culture 60
10.2. Photolabeling of HeLa Cells for Imaging Via Confocal Microscopy 60
10.3. Western Blot Procedure of HeLa Cell Lysate..61
10.4. Photolabeling of MCF 10A and MDA-MB-231 cells 63
11. Animal Studies 64
11.1 *Ex vivo* Brain Labelling 65
11.2 *In vivo* Liver Labelling...65
12. Computational Studies...65
12.1 Computational Methods...65
12.2 Model system for computational mechanistic studies 67
12.3 Explicit water solvation 68
12.4 Marcus barriers for outer-sphere electron transfer...69
12.5 Donor-acceptor associative complexes...72
12.6 State-specific modeling of excited state energies using ΔSCF 73
12.7 Post-electron transfer radical relay mechanism 75
12.8 Density Functional Benchmarking 77
13. NMR Spectra 78
14. References 108

1. General Considerations

NMR Spectroscopy

NMR spectra were recorded on a Bruker Avance 400 spectrometer (400 MHz for ^{1}H, 162 MHz for ^{31}P and 101 MHz for ^{13}C). and ^{19}F NMR spectra were recorded on Bruker 500 (471 MHz). Chemical shifts of the ^{1}H resonances are reported in ppm relative to tetramethylsilane, with the residual solvent resonance ($CDCl_3$, δ = 7.26 ppm, Acetonitrile-*d₃*, δ = 1.94 ppm, and DMSO-*d₆*, δ = 2.50 ppm) as the internal reference. Spectra are reported as the following: chemical shift (δ ppm), multiplicity (s = singlet, b s = broad singlet, d = doublet, t = triplet, q = quartet, m = multiplet), coupling constant (Hz), and integration. Chemical shifts of the ^{13}C were reported in ppm relative to tetramethylsilane with the solvent resonance used as the internal reference ($CDCl_3$, δ = 77.2 ppm, Acetonitrile-d_3, δ = 1.32 ppm and 118.26 ppm, and DMSO-d_6, δ = 39.52 ppm).

Mass Spectra

High resolution mass spectra (HRMS) were collected on an Agilent 6224 TOF LC/MS. LC-MS was performed on a Thermo Scientific TSQ Fortis Plus Triple Quadrupole mass spectrometer coupled to a Zorbax RRHD Eclipse Plus C18 column (95 Å, 1.8 µm, 3.0 x 100 mm). Bruker Matrix assisted laser desorption/ionization (MALDI-TOF/TOF) instrument was used to find exact $[M+H]^+$ (m/z). Reported $[M+H]^+$ (m/z) are observed in reflector polarization (positive) mode. High resolution mass spectra (HRMS) were recorded on an Agilent 6224 TOF LC/MS (APCI source).

Reagents and Solvents

All chemical reagents were purchased from commercial suppliers (Ambeed, Oakwood Chemical, Sigma-Aldrich, TCI, Alfa, or Thermo Fisher Scientific). Rink amide resin (0.3 – 0.8 meq/g, 200-400 mesh) was purchased from Chem-Impex. $[(Ir[dF(CF_3)ppy]_2(dtbpy)) PF_6]$ were prepared according to literature procedures. [1] Dichloromethane (DCM), diethyl ether (Et_2O), tetrahydrofuran (THF), methanol (MeOH), ethyl acetate (EtOAc), and dimethylformamide (DMF) were purchased from Fisher Scientific.

Chromatography

Reactions were monitored by thin-layer chromatography (TLC) on Merck TLC silica gel 60 F254 plates and compounds were visualized by UV light (254 nm) or staining with $KMnO_4$. Flash column chromatography was performed using SiliaFlash® Irregular Silica Gel, F60 40 - 63 µm, 60 Å, purchased from Silicycle®.

Photochemical Equipment and Setup:

The light source used for the photoredox reactions: Kessil PR160L 660 nm (35 W), Kessil PR160L 740 nm (35 W), and Kessil PR160L 525 nm (44 W). (For more details about the lamp, please go to https://www.kessil.com/products/science_PR160L.php). Over a stir plate equipped with a fan, the sealed vials were positioned in between two lamps, approximately 4.5 cm away from each. Samples irradiated with the 1040 nm laser were photoexcited via 1040 nm light from an Innolas SpitLight Evo S OPO Laser system, pulsed at 50 Hz (pulse duration 10 ns), with a ThorLabs Hard-Coated Longpass Filters (FELH0750) with a cut-on wavelength of 750 nm placed in front of the laser. The beam provided excitation energies between 0.1 to 1 mJ. Samples irradiated with the 1064 nm laser were photoexcited via 1064 nm light from a ThorLabs 1064 nm, 450 mW, Ø9 mm laser diode (Part Number: L1064H2). The ThorLabs diode was mounted on ThorLabs TE-Cooled Mount for Ø9.0 mm Laser Diode (Part Number: LDM90). The ThorLabs diode was connected to a LDC205C Benchtop LD Current Controller as well as a TED200C Benchtop Temperature Controller.

UV-Vis Equipment

UV-Vis spectrum was recorded on Multicell Peltier Module G9889A for Agilent Cary 3500 UV-Vis Spectrophotometer.

2. Synthetic Procedures

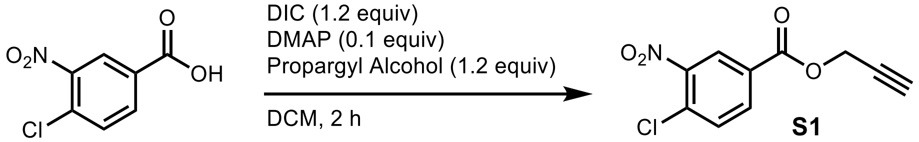

4-chloro-3-nitrobenzoic acid (11.0 g, 54.6 mmol, 1 equiv) was dissolved in DCM in a flame-dried 500 mL flask equipped with a stir bar. Propargyl alcohol (3.78 mL, 65.6 mmol, 1.2 equiv) and DMAP (DMAP = 4-dimethylaminopyridine) (667 mg, 5.5 mmol, 0.1 equiv) were added to the reaction mixture and the reaction flask was sealed with a septum. Then, DIC (DIC = N,N'-Diisopropylcarbodiimide) (10.3 mL, 65.6 mmol, 1.2 equiv) was added dropwise via syringe. After stirring at room temperature for 2 hours, 100 mL of Et_2O was added. The reaction mixture was filtered through a pad of Celite® and washed with DCM/Et_2O. After solvent removal in vacuo, the residue was purified by flash column chromatography (*n*-hexane/EtOAc 3:1) to afford **S1** (11.8 g, 49.2 mmol) as a pale yellow solid in 90% yield.

^{1}H NMR (400 MHz, $CDCl_3$) δ: 8.51 (d, J = 2.1 Hz, 1H), 8.18 (dd, J = 8.4, 2.0 Hz, 1H), 7.66 (d, J = 8.4 Hz, 1H), 4.95 (d, J = 2.5 Hz, 2H), 2.56 (t, J = 2.5 Hz, 1H).

^{13}C{^{1}H} NMR (101 MHz, $CDCl_3$) δ:163.2, 133.9, 132.5, 132.3, 129.5, 126.9, 76.0, 53.6, 42.4, 23.6.

ESI$^+$-HRMS: calculated for $C_{10}H_6ClNO_4$ ($[M]^+$) 238.9980, found 238.9973.

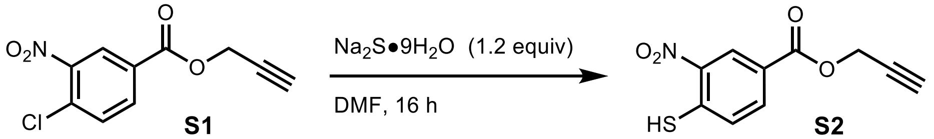

S1 (11.8 g, 49.2 mmol, 1 equiv) was dissolved in DMF in a flame-dried 500 mL flask equipped with a stir bar. $Na_2S \bullet 9H_2O$ (14.2 g, 59.1 mmol, 1.2 equiv) was added to the reaction mixture and the flask was sealed with a septum. After stirring at room temperature for 16 h, the reaction was cooled to 0° C. The reaction mixture was diluted with 200 mL of H_2O and 150 mL of 2 M HCl solution was added to precipitate out yellow solids. The aqueous phase was extracted with DCM (2 x 200 mL) and the combined organic phase was washed with water (4 x 600 mL), dried over Na_2SO_4, filtered, and evaporated *in vacuo* to obtain **S2** (10.2 g, 43 mmol) as a yellow solid in 87% yield.

^{1}H NMR (400 MHz, $CDCl_3$) δ: 8.92 (d, J = 1.9 Hz, 1H), 8.08 (dd, J = 8.3, 1.9 Hz, 1H), 7.53 (d, J = 8.3 Hz, 1H), 4.96 (d, J = 2.5 Hz, 2H), 4.23 (s, 1H), 2.56 (t, J = 2.5 Hz, 1H).

^{13}C{^{1}H} NMR (101 MHz, $CDCl_3$) δ: 163.6, 145.0, 140.3, 133.9, 132.4, 127.8, 127.5, 75.8, 53.3.

ESI$^+$-HRMS: calculated for $C_{10}H_7NNaO_4S$ ($[M+Na]^+$) 259.9988, found 259.9989.

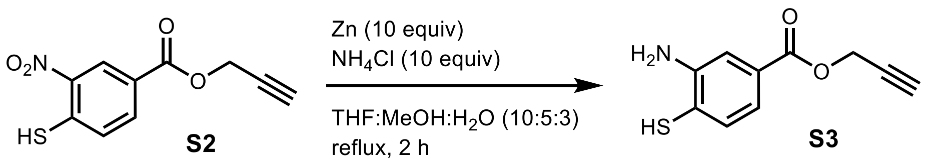

S2 (10.0 g, 42.2 mmol, 1 equiv) was dissolved in a solution mixture of THF:MeOH:H_2O (10:5:3) in a flame-dried 500 mL flask equipped with a stir bar. Zinc powder (27.6 g, 421.5 mmol, 10 equiv) and ammonium chloride (22.6 g, 421.5 mmol, 10 equiv) was added to the reaction mixture. After stirring under reflux using an oil bath for 2 hours, the reaction mixture was filtered through a pad of Celite® and washed with 200 mL of MeOH. The filtrate was evaporated *in vacuo* to obtain **S3** (4.8 g, 23.3 mmol) as a pale yellow solid in 55% yield.

^{1}H NMR (400 MHz, DMSO-*d*$_6$) δ: 7.54 (d, *J* = 2.0 Hz, 1H), 7.42 (d, *J* = 8.1 Hz, 1H), 7.30 (dd, *J* = 8.1, 2.0 Hz, 1H), 5.88 (s, 2H), 4.88 (d, *J* = 2.5 Hz, 2H), 3.57 (t, *J* = 2.4 Hz, 1H).

^{13}C{^{1}H} NMR (101 MHz, DMSO-*d*$_6$) δ: 165.1, 142.0, 132.3, 122.5, 78.7, 77.7, 51.9

ESI$^+$-HRMS: calculated for $C_{10}H_{10}NO_2S$ ($[M+H]^+$) 208.0427, found 208.0444.

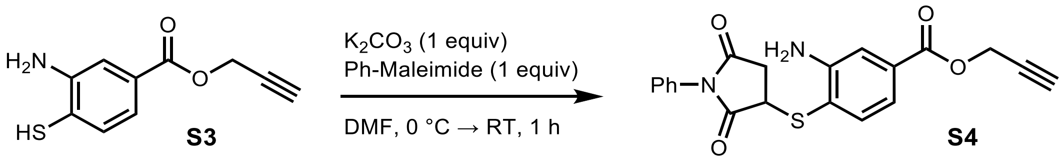

S3 (1.00g, 4.83 mmol, 1 equiv) was dissolved in DMF in a flame-dried 100 mL flask equipped with a stir bar. The reaction mixture was cooled to 0 °C and K_2CO_3 (67 mg, 4.8 mmol, 1 equiv) was added dropwise and phenyl maleimide (836 mg, 4.8 mmol, 1 equiv) was added to the reaction mixture. After stirring at room temperature for 1 hour, the reaction mixture was poured into 20 mL of H_2O and extracted with EtOAc (3 x 10 mL). The combined organic phases were dried over Na_2SO_4, filtered, evaporated *in vacuo*, and the crude product was purified by flash column chromatography (5% MeOH in DCM) to afford **S4** (279 mg, 0.73 mmol) as a yellow solid in 15% yield.

^{1}H NMR (400 MHz, Acetonitrile-*d*$_3$) δ: 7.53 – 7.38 (m, 5H), 7.22 (dd, *J* = 8.0, 1.9 Hz, 1H), 7.17 – 7.12 (m, 2H), 5.08 (s, 2H), 4.88 (d, *J* = 2.5 Hz, 2H), 4.21 (dd, *J* = 9.3, 4.0 Hz, 1H), 3.26 (dd, *J* = 18.7, 9.3 Hz, 1H), 2.85 – 2.76 (m, 2H).

^{13}C{^{1}H} NMR (101 MHz, Acetonitrile-*d*$_3$) δ: 176.4, 175.0, 166.1, 151.5, 138.6, 133.4, 133.0, 130.0, 129.6, 127.8, 118.9, 118.6, 116.3, 78.8, 76.4, 53.4, 44.4, 36.7, 30.8.

ESI$^+$-HRMS: calculated for $C_{20}H_{17}N_2O_4S$ ($[M+H]^+$) 381.0909, found 381.0912.

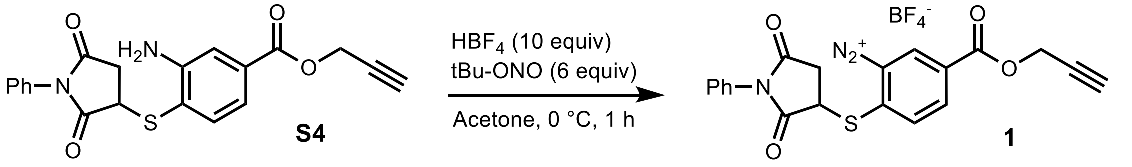

S4 (100 mg, 0.26 mmol, 1 equiv) was dissolved in acetone in a flame-dried 50 mL flask equipped with a stir bar. Fluoroboric acid (0.35 mL, 2.6 mmol, 10 equiv) was added to the reaction mixture dropwise. Then, the reaction mixture was cooled to 0 °C and tert-Butyl nitrite (0.19 mL, 1.6 mmol, 6 equiv) was added dropwise *via* syringe. After stirring at 0 °C for 1 hour, 6 mL of Et_2O was added to precipitate out yellow solids. The reaction was filtered using a Buchner funnel to obtain **1** (63 mg, 0.13 mmol) as a yellow solid in 50% yield.

^{1}H NMR (400 MHz, DMSO-d_6) δ: 9.39 (d, J = 1.9 Hz, 1H), 8.65 (dd, J = 8.5, 2.0 Hz, 1H), 8.40 (d, J = 8.6 Hz, 1H), 7.59 – 7.49 (m, 3H), 7.49 – 7.42 (m, 1H), 7.35 – 7.27 (m, 2H), 5.19 (dd, J = 9.4, 5.5 Hz, 1H), 5.07 (d, J = 2.5 Hz, 2H), 3.75 (t, J = 2.4 Hz, 1H), 3.46 (dd, J = 18.1, 9.4 Hz, 1H), 3.12 (dd, J = 18.1, 5.6 Hz, 1H).

^{13}C{^{1}H} NMR (101 MHz, DMSO-d_6) δ: 174.3, 173.4, 161.6, 146.9, 139.6, 134.6, 133.6, 132.1, 129.6, 129.0, 128.7, 126.9, 118.2, 79.0, 77.7, 54.0, 44.9, 35.7.

^{19}F{^{1}H} NMR (471 MHz, DMSO-d_6) δ: -148.19, -148.24.

ESI$^+$-HRMS: calculated for $C_{20}H_{14}N_3NaO_4S$ ([M+Na]$^+$) 415.0597, found 415.0594.

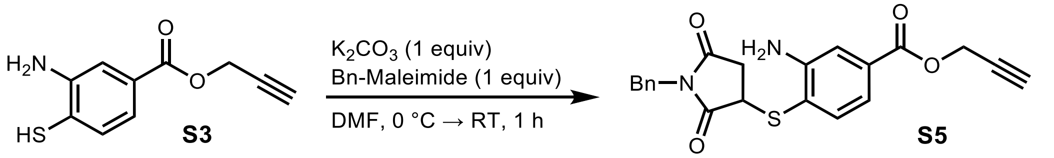

S3 (3.00g, 14.5 mmol, 1 equiv) was dissolved in DMF in a flame-dried 250 mL flask equipped with a stir bar. The reaction mixture was cooled to 0° C and K_2CO_3 (2.0 g, 14.5 mmol, 1 equiv) was added dropwise and benzyl maleimide (2.7 g, 14.5 mmol, 1 equiv) was added to the reaction mixture. After stirring at room temperature for 1 hour, the reaction mixture was poured into 20 mL of H_2O and extracted with EtOAc (3 x 20 mL). The combined organic phases were dried over Na_2SO_4, filtered, evaporated *in vacuo*, and the crude product was purified by flash column chromatography (5% MeOH in DCM) to afford **S5** (4.9 g, 12.4 mmol) as a red solid in 86% yield.

^{1}H NMR (400 MHz, DMSO-d_6) δ: 7.36 (d, J = 1.9 Hz, 1H), 7.29 – 7.24 (m, 4H), 7.13 (dd, J = 7.4, 2.1 Hz, 2H), 6.94 (dd, J = 8.1, 1.9 Hz, 1H), 5.82 (s, 2H), 5.75 (s, 1H), 4.96 – 4.89 (m, 2H), 4.57 – 4.45 (m, 2H), 4.35 (dd, J = 9.0, 4.0 Hz, 1H), 3.63 (t, J = 2.5 Hz, 1H), 3.22 (dd, J = 18.5, 9.1 Hz, 1H), 2.63 (dd, J = 18.5, 4.0 Hz, 1H).
^{13}C{^{1}H} NMR (101 MHz, Acetonitrile-d_3) δ: 176.9, 175.6, 166.1, 151.3, 138.4, 136.8, 132.7, 129.4, 128.9, 128.6, 128.5, 118.7, 118.6, 118.3, 116.3, 78.8, 76.4, 53.3, 44.1, 43.1, 36.3, 1.9, 1.7, 1.5, 1.3, 1.1, 0.9, 0.7.

ESI$^+$-HRMS: calculated for $C_{21}H_{18}N_2NaO_4S$ ([M+Na]$^+$) 417.0879, found 417.0881.

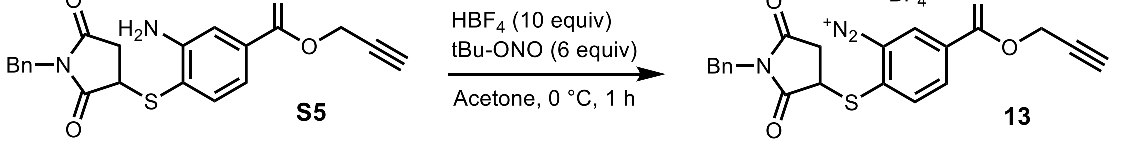

S5 (170 mg, 0.43 mmol, 1 equiv) was dissolved in acetone in a flame-dried 50 mL flask equipped with a stir bar. Fluoroboric acid (0.58 mL, 4.3 mmol, 10 equiv) was added to the reaction mixture dropwise. Then, the reaction mixture was cooled to 0 °C and tert-Butyl nitrite (0.31 mL, 2.6 mmol, 6 equiv) was added dropwise *via* syringe. After stirring at 0 °C for 1 hour, 6 mL of Et_2O was added to precipitate out yellow solids. The reaction was filtered using a Buchner funnel to obtain **13** (148 mg, 0.30 mmol) as a yellow solid in 70% yield.

^{1}H NMR (400 MHz, Acetonitrile-*d*$_3$) δ: 9.01 (d, *J* = 1.8 Hz, 1H), 8.58 (dd, *J* = 8.5, 1.9 Hz, 1H), 8.29 (d, *J* = 8.5 Hz, 1H), 7.37 – 7.24 (m, 5H), 5.02 (d, *J* = 2.5 Hz, 2H), 4.70 – 4.64 (m, 1H), 4.62 (d, *J* = 3.3 Hz, 2H), 3.39 (dd, *J* = 18.7, 9.5 Hz, 1H), 2.94 (t, *J* = 2.5 Hz, 1H), 2.86 (dd, *J* = 18.7, 5.1 Hz, 1H).

^{13}C{^{1}H} NMR (101 MHz, Acetonitrile-*d*$_3$) δ: 175.79, 174.38, 162.28, 146.55, 141.98, 136.87, 136.49, 135.42, 132.77, 129.56, 129.19, 128.85, 77.77, 77.44, 55.00, 45.65, 43.61, 36.07.

^{19}F{^{1}H} NMR (471 MHz, Acetonitrile-*d*$_3$) δ: -148.19, -148.24.

ESI$^+$-HRMS: calculated for $C_{21}H_{16}N_3NaO_4S$ ($[M+Na]^+$) 429.0748, found 429.0811.

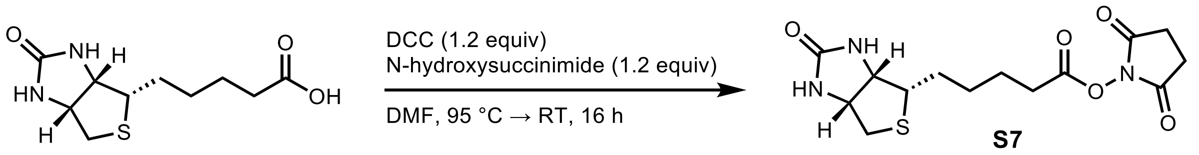

Biotin (8.00g, 33 mmol, 1 equiv) was dissolved in DMF in a flame-dried 250 mL flask equipped with a stir bar. DCC (8.1 g, 39 mmol, 1.2 equiv) and N-hydroxysuccinimide (4.5 g, 39 mmol, 1.2 equiv) was added to the reaction mixture. After stirring at room temperature for 16 hours, the reaction mixture was filtered through Celite® to remove the urea. 500 mL of Et_2O was added to the filtrate to crash out white solids. Filtration of the white solids afforded **S7** (10.8 g, 32 mmol) in 97% yield. The NMR spectra are consistent with the reported literature.[2]

^{1}H NMR (400 MHz, DMSO-*d*$_6$) δ: 6.42 (d, *J* = 1.9 Hz, 1H), 6.36 (s, 1H), 4.31 (ddt, *J* = 7.6, 5.2, 1.2 Hz, 1H), 4.15 (ddd, *J* = 7.7, 4.4, 1.8 Hz, 1H), 3.11 (ddd, *J* = 8.2, 6.4, 4.2 Hz, 1H), 2.89 (s, 1H), 2.86 – 2.82 (m, 1H), 2.67 (t, *J* = 7.4 Hz, 2H), 2.58 (d, *J* = 12.5 Hz, 1H), 1.74 – 1.31 (m, 6H).

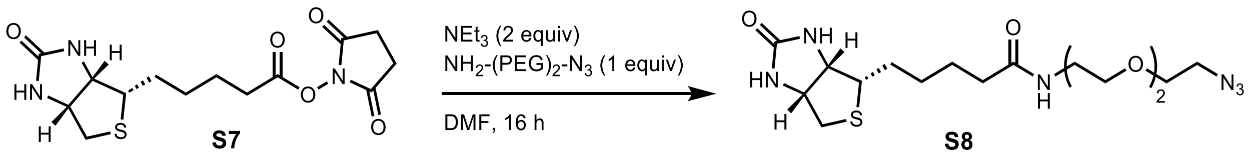

S7 (1.8 g, 5.4 mmol, 1 equiv) was dissolved in DMF in a flame-dried 100 mL flask equipped with a stir bar. Triethylamine (1.5 mL, 10.7 mmol, 2 equiv) and NH_2-$(PEG)_2$-N_3 (0.93 g, 5.4 mmol, 1 equiv) were added to the reaction flask and stirred at room temperature. After 16 h, the reaction was concentrated *in vacuo*, and the crude product was purified by flash column chromatography (10% MeOH in DCM) to afford **S8** (1.4 g, 3.6 mmol) as a white solid in 66% yield. The NMR spectra are consistent with the reported literature.[3]

^{1}H NMR (400 MHz, DMSO-d_6) δ: 7.82 (t, *J* = 5.6 Hz, 1H), 6.42 (t, *J* = 1.8 Hz, 1H), 6.36 (s, 1H), 4.31 (dd, *J* = 7.7, 5.1 Hz, 1H), 4.13 (ddd, *J* = 7.7, 4.4, 1.9 Hz, 2H), 3.67 – 3.46 (m, 7H), 3.40 (q, *J* = 5.6, 4.8 Hz, 7H), 3.23 – 3.14 (m, 4H), 3.10 (ddd, *J* = 8.6, 6.1, 4.3 Hz, 1H), 2.82 (dd, *J* = 12.4, 5.0 Hz, 1H), 2.60 (s, 2H), 2.07 (t, *J* = 7.4 Hz, 2H), 1.68 – 1.22 (m, 7H).

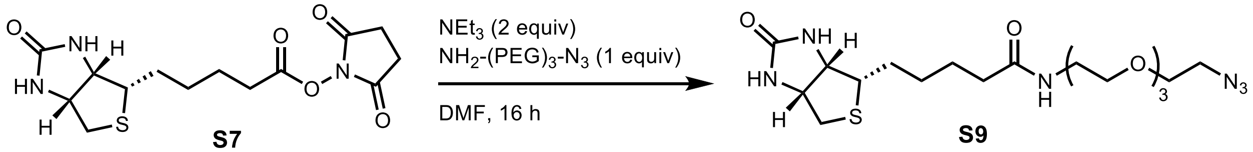

S7 (4.00 g, 11.7 mmol, 1 equiv) was dissolved in DMF in a flame-dried 100 mL flask equipped with a stir bar. Triethylamine (3.27 mL, 23.4 mmol, 2 equiv) and NH_2-$(PEG)_3$-N_3 (2.56 g, 11.7 mmol, 1 equiv) was added to the reaction flask and stirred at room temperature. After 16 h, the reaction was concentrated *in vacuo*, and the crude product was purified by flash column chromatography (10% MeOH in DCM) to afford **S9** (3.56 g, 8.01 mmol) as a white solid in 68% yield. The NMR spectra are consistent with the reported literature.[3]

^{1}H NMR (400 MHz, DMSO-d_6) δ: 7.82 (t, *J* = 5.7 Hz, 1H), 6.41 (t, *J* = 1.8 Hz, 1H), 6.35 (t, *J* = 1.4 Hz, 1H), 4.30 (ddt, *J* = 7.6, 5.2, 1.1 Hz, 1H), 4.14 – 4.12 (m, 1H), 3.63 – 3.58 (m, 2H), 3.58 – 3.47 (m, 9H), 3.39 (dd, *J* = 5.6, 4.2 Hz, 4H), 3.17 (d, *J* = 5.2 Hz, 6H), 3.09 (ddd, *J* = 8.6, 6.2, 4.4 Hz, 1H), 2.82 (dd, *J* = 12.4, 5.1 Hz, 1H), 2.57 (d, *J* = 12.9 Hz, 1H), 2.06 (t, *J* = 7.4 Hz, 2H), 1.65 – 1.22 (m, 7H).

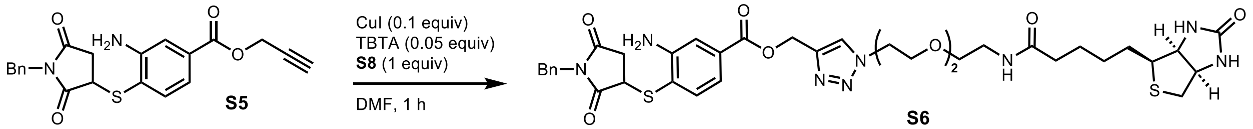

S5 (120 mg, 0.3 mmol, 1 equiv) was dissolved in DMF in a flame-dried 50 mL flask equipped with a stir bar. CuI (6 mg, 0.03 mmol, 0.1 equiv), TBTA (TBTA = Tris(benzyltriazolylmethyl)amine) (8 mg, 0.015 mmol, 0.05 equiv), and Biotin-$(PEG)_2$-N_3 **S8** (118 mg, 0.03 mmol, 1 equiv) was added to the reaction mixture and the flask was sealed with a septum. After stirring for 1 hour, the reaction mixture was concentrated *in vacuo* and purified by flash column chromatography (5% MeOH in DCM) to afford **S6** (125 mg, 0.16 mmol) as a yellow oil in 53% yield.

^{1}H NMR (400 MHz, Acetonitrile-d_3) δ: 7.97 (s, 1H), 7.35 – 7.21 (m, 5H), 7.19 – 7.14 (m, 2H), 7.03 (dd, *J* = 8.1, 1.9 Hz, 1H), 6.52 (d, *J* = 5.9 Hz, 1H), 5.45 (d, *J* = 5.3 Hz, 1H), 5.38 (d, *J* = 1.6 Hz, 2H), 5.14 (s, 1H), 5.05 (s, 2H), 4.56 – 4.50 (m, 4H), 4.38 (ddt, *J* = 7.6, 5.0, 1.2 Hz, 1H), 4.20 (ddd, *J* = 7.8, 4.5, 1.9 Hz, 1H), 4.10 (dd, *J* = 9.1, 4.0 Hz, 1H), 3.85 (dd, *J* = 5.6, 4.6 Hz, 2H), 3.56 – 3.52 (m, 2H), 3.51 – 3.46 (m, 2H), 3.39 (t, *J* = 5.6 Hz, 2H), 3.30 – 3.21 (m, 8H), 3.16 – 3.07 (m, 2H), 2.85 (dd, *J* = 12.7, 5.0 Hz, 1H), 2.67 – 2.58 (m, 2H), 2.11 (t, *J* = 7.4 Hz, 2H), 1.69 – 1.45 (m, 5H), 1.41 – 1.25 (m, 3H).

^{13}C{^{1}H} NMR (101 MHz, Acetonitrile-d_3) δ: 177.0, 175.6, 173.8, 166.7, 163.9, 151.4, 143.3, 138.4, 136.9, 133.2, 129.5, 128.8, 128.6, 126.0, 118.6, 116.3, 71.0, 70.7, 70.3, 69.8, 62.3, 60.7, 59.1, 56.3, 51.0, 49.8, 44.1, 43.1, 41.1, 39.7, 36.3, 29.0, 28.9, 26.3.

ESI$^+$-HRMS: calculated for $C_{37}H_{47}N_8O_8S_2$ ($[M+H]^+$) 795.2958, found 795.2967.

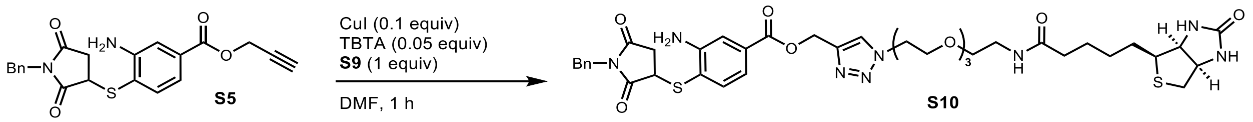

S5 (956 mg, 2.42 mmol, 1 equiv) was dissolved in DMF in a flame-dried 100 mL flask equipped with a stir bar. CuI (46.2 mg, 0.2 mmol, 0.1 equiv), TBTA (TBTA = Tris(benzyltriazolylmethyl)amine) (64.3 mg, 0.12 mmol, 0.05 equiv), and Biotin-(PEG)$_3$-N$_3$ **S9** (1.08 g, 2.42 mmol, 1 equiv) was added to the reaction mixture and the flask was sealed with a septum. After stirring for 1 hour, the reaction mixture was concentrated *in vacuo* and purified by flash column chromatography (5% MeOH in DCM) to afford **S10** (1.30 g, 1.50 mmol) as a yellow oil in 64% yield.

^{1}H NMR (400 MHz, DMSO-*d*$_6$) δ: 8.20 (s, 1H), 7.80 (t, *J* = 5.7 Hz, 1H), 7.33 (d, *J* = 1.9 Hz, 1H), 7.28 – 7.19 (m, 4H), 7.14 – 7.09 (m, 2H), 6.91 (dd, *J* = 8.0, 1.9 Hz, 1H), 6.40 (d, *J* = 1.9 Hz, 1H), 6.35 (s, 1H), 5.78 (s, 2H), 5.36 (d, *J* = 1.6 Hz, 2H), 4.58 – 4.51 (m, 2H), 4.49 (d, *J* = 3.4 Hz, 2H), 4.35 – 4.26 (m, 2H), 3.85 – 3.79 (m, 2H), 3.53 – 3.46 (m, 4H), 3.38 (s, 2H), 3.10 – 3.05 (m, 1H), 2.84 – 2.77 (m, 1H), 2.05 (t, *J* = 7.4 Hz, 2H), 1.67 – 1.21 (m, 7H).

^{13}C{^{1}H} NMR (101 MHz, DMSO-*d*$_6$) δ: 176.04, 174.97, 172.64, 172.30, 168.57, 166.00, 165.65, 165.10, 163.14, 150.82, 141.90, 141.84, 139.30, 137.20, 136.93, 135.87, 131.48, 128.58, 128.45, 128.22, 128.16, 127.62, 127.34, 126.99, 125.52, 124.99, 123.75, 117.55, 117.18, 116.76, 115.30, 69.94, 69.91, 69.84, 69.77, 69.75, 69.33, 68.85, 68.83, 61.34, 59.52, 58.18, 58.02, 55.72, 55.66, 49.68, 48.80, 43.09, 42.43, 41.94, 35.56, 35.40, 35.33, 28.41, 28.35, 28.24, 25.48.

ESI$^+$-HRMS: calculated for $C_{39}H_{50}N_8O_9S_2$ ([M+H]$^+$) 839.3220, found 839.3215.

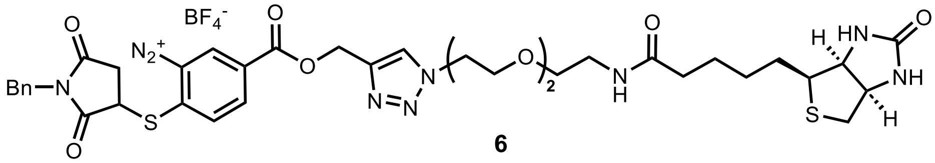

6

S6 (100 mg, 0.13 mmol, 1 equiv) was dissolved in acetone in a flame-dried 25 mL flask equipped with a stir bar. Fluoroboric acid (81 μL, 1.3 mmol, 10 equiv) was added to the reaction mixture dropwise. Then, the reaction mixture was cooled to 0 °C and tert-Butyl nitrite (90 μL, 0.76 mmol, 6 equiv) was added dropwise *via* syringe. After stirring at 0 °C for 1 hour, 6 mL of Et$_2$O was added to precipitate out yellow solids. The reaction was filtered using a Buchner funnel to obtain **6** (56 mg, 58 μmol) as a yellow solid in 46% yield.

^{1}H NMR (400 MHz, DMSO-*d*$_6$) δ: 9.31 (d, *J* = 1.9 Hz, 1H), 8.79 (s, 1H), 8.53 (dd, *J* = 8.5, 1.9 Hz, 1H), 8.34 – 8.23 (m, 2H), 7.93 – 7.74 (m, 1H), 7.41 – 7.17 (m, 6H), 5.11 (dd, *J* = 9.3, 5.0 Hz, 1H), 4.82 (t, *J* = 6.4 Hz, 1H), 4.64 – 4.49 (m, 5H), 4.35 – 4.20 (m, 1H), 4.12 (dt, *J* = 7.8, 4.0 Hz, 1H), 3.83 (t, *J* = 5.2 Hz, 3H), 3.57 – 3.44 (m, 7H), 3.37 (dtd, *J* = 15.7, 5.8, 3.1 Hz, 6H), 3.24 – 3.11 (m, 4H), 3.10 – 3.01 (m, 1H), 3.00 – 2.90 (m, 1H), 2.66 (d, *J* = 13.2 Hz, 1H), 2.07 (d, *J* = 7.6 Hz, 4H), 1.75 – 1.20 (m, 9H).

$^{13}C\{^1H\}$ NMR (101 MHz, DMSO-d_6) δ: 174.8, 174.1, 172.1, 162.7, 161.9, 153.7, 145.8, 140.8, 139.6, 135.6, 134.6, 134.0, 130.2, 128.5, 127.8, 127.6, 125.7, 118.6, 69.5, 69.4, 69.1, 68.6, 61.0, 59.5, 59.2, 58.6, 55.4, 54.0, 49.5, 44.4, 42.1, 38.4, 35.4, 35.1, 35.0, 34.5, 28.2, 28.0, 27.6, 25.3, 25.1.

$^{19}F\{^1H\}$ NMR (471 MHz, DMSO-d_6) δ: -148.19, -148.25.

ESI$^+$-HRMS: calculated for $C_{37}H_{44}N_9O_8S_2$ ($[M]^+$) 806.2749, found 806.2698.

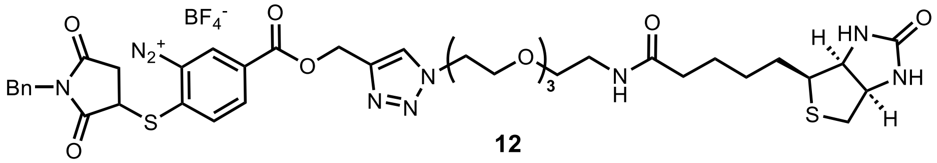

12

S10 (400 mg, 0.48 mmol, 1 equiv) was dissolved in acetone in a flame-dried 25 mL flask equipped with a stir bar. Fluoroboric acid (299 μL, 4.77 mmol, 10 equiv) was added to the reaction mixture dropwise. Then, the reaction mixture was cooled to 0 °C and tert-Butyl nitrite (340 μL, 2.86mmol, 6 equiv) was added dropwise *via* syringe. After stirring at 0 °C for 1 hour, 6 mL of Et_2O was added to precipitate out yellow solids. The reaction was filtered using a Buchner funnel to obtain **12** (32 mg, 34 μmol) as a yellow solid in 7% yield.

^1H NMR (400 MHz, DMSO-d_6) δ: 9.30 (d, J = 1.9 Hz, 1H), 8.81 – 8.77 (m, 1H), 8.53 (dd, J = 8.5, 1.9 Hz, 1H), 8.29 (d, J = 8.6 Hz, 1H), 8.27 (s, 1H), 7.83 (t, J = 5.7 Hz, 1H), 7.34 – 7.22 (m, 6H), 5.50 (s, 2H), 5.11 (dd, J = 9.3, 5.1 Hz, 1H), 4.86 – 4.79 (m, 1H), 4.60 – 4.53 (m, 4H), 4.25 (ddd, J = 7.1, 4.4, 1.4 Hz, 1H), 3.83 (t, J = 5.2 Hz, 3H), 3.42 – 3.31 (m, 9H), 3.05 (dd, J = 18.3, 5.0 Hz, 1H), 2.98 – 2.92 (m, 1H), 2.67 (d, J = 13.3 Hz, 1H), 2.05 (d, J = 7.4 Hz, 2H), 1.73 – 1.24 (m, 7H).

$^{13}C\{^1H\}$ NMR (101 MHz, DMSO-d_6) δ: 174.8, 174.1, 172.1, 162.7, 161.9, 153.7, 145.7, 140.8, 139.6, 135.6, 134.7, 134.6, 134.0, 130.2, 128.5, 127.8, 127.6, 125.7, 118.6, 69.7, 69.6, 69.5, 69.1, 68.6, 64.9, 61.0, 59.5, 59.2, 58.6, 55.4, 54.0, 49.5, 44.3, 42.1, 35.4, 35.1, 35.0, 34.5, 28.2, 28.0, 27.6, 25.2, 25.1, 15.2.

$^{19}F\{^1H\}$ NMR (471 MHz, DMSO-d_6) δ: -148.19, -148.25.

ESI$^+$-HRMS: calculated for $C_{39}H_{48}N_9O_9S_2$ ($[M]^+$) 850.3011, found 850.2997.

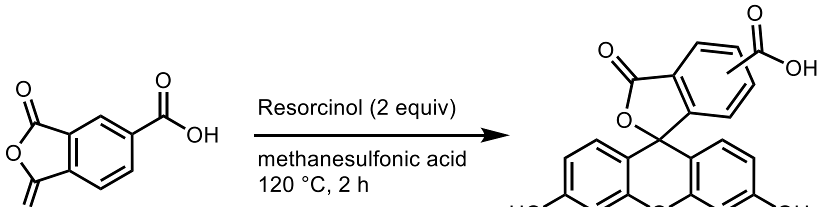

S11

Resorcinol (5.73 g, 52 mmol, 2 equiv) and trimellitic anhydride (5.0 g, 26 mmol, 1 equiv) were dissolved in concentrated methanesulfonic acid (40.0 mL) in a flame-dried 250 mL flask equipped

with a stir bar. The reaction mixture was brough to reflux at 120 °C. After 2 hours, the reaction mixture was poured into 500 mL of cold water and the orange solids were filtered and washed with 100 mL of water. The orange solid was dissolved in 4 M NaOH (50 mL) and was acidified with concentrated H_2SO_4 (60 mL). The dark orange precipitate was filtered and washed with water (3 x 100 mL) to obtain **S11** (8.76 g, 23.3 mmol) as a dark orange solid in 89% yield. The NMR spectra are consistent with the reported literature.[4]

^{1}H NMR (400 MHz, DMSO-*d*$_6$) δ: 8.39 (d, *J* = 1.4 Hz, 1H), 8.29 (dd, *J* = 8.0, 1.5 Hz, 1H), 8.22 (dd, *J* = 8.0, 1.3 Hz, 1H), 8.13 – 8.09 (m, 1H), 7.64 (t, *J* = 1.1 Hz, 1H), 7.39 (d, *J* = 7.9 Hz, 1H), 6.70 (t, *J* = 2.1 Hz, 4H), 6.61 (dd, *J* = 8.7, 3.0 Hz, 4H), 6.55 (dt, *J* = 8.7, 2.1 Hz, 4H).

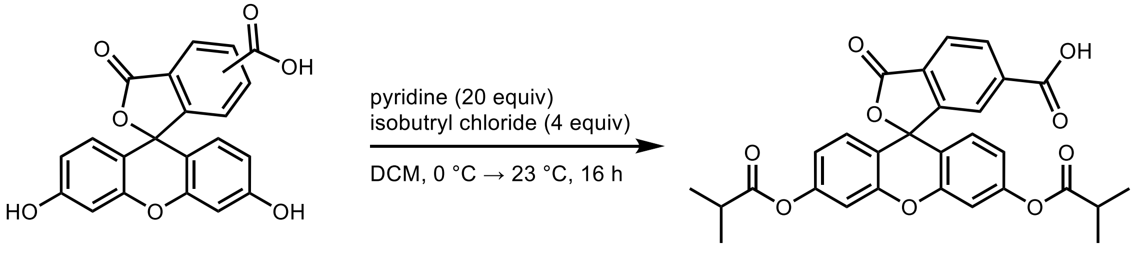

S11 **S12**

S11 (3.16 g, 8.4 mmol, 1 equiv) was suspended in DCM (20 mL) followed by the addition of pyridine (13.6 mL, 168 mmol, 20 equiv) in a flame-dried 250 mL flask equipped with a stir bar. The reaction mixture was cooled to 0 °C and isobutyryl chloride (3.52 mL, 33.6 mmol, 4 equiv) was added dropwise. After stirring at 16 hours while slowly warming up to room temperature, 1 M HCl (30 mL) was added to the reaction flask. The organic phase was separated with EtOAc (3 x 20 mL) and the combined organic phases were dried over Na_2SO_4, filtered, evaporated *in vacuo*, and the crude product was purified by flash column chromatography (1% MeOH in DCM + 1% AcOH). 6-isomer was separated by adding DIPA (DIPA = diisopropylamine) (10 mL) to a cooled solution of the purified mixture in EtOAc (5 mL). The diisopropylammonium salt was acidified with 1 M HCl (10 mL) and the aqueous layer was extracted with EtOAc (3 x 20 mL). The combined organic phases were dried over Na_2SO_4, filtered, evaporated *in vacuo* to obtain **S12** (1.11 g, 2.15 mmol) as an orange solid in 26% yield. The NMR spectra are consistent with the reported literature.[5]

^{1}H NMR (400 MHz, DMSO-*d*$_6$) δ: 8.27 (dd, *J* = 8.1, 1.3 Hz, 1H), 8.18 (dd, *J* = 8.0, 0.8 Hz, 1H), 7.83 (dd, *J* = 1.3, 0.8 Hz, 1H), 7.29 (dd, *J* = 2.0, 0.7 Hz, 2H), 7.01 – 6.88 (m, 5H), 2.84 (hept, *J* = 7.0 Hz, 2H), 1.24 (d, *J* = 7.0 Hz, 12H).

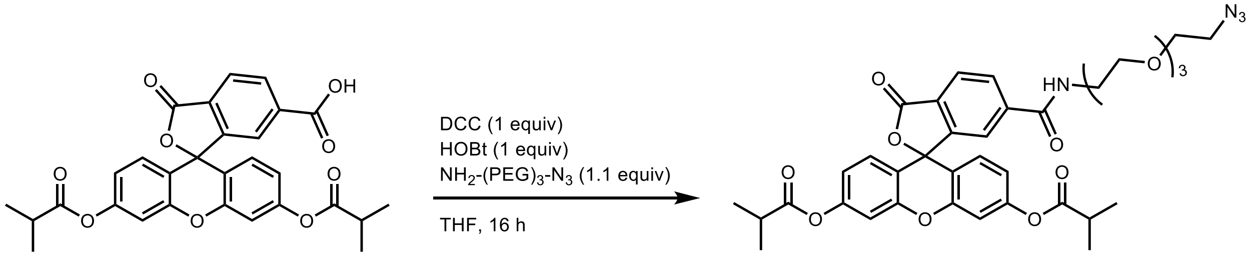

S12 **S13**

S12 (1.11 g, 2.15 mmol, 1 equiv) was dissolved in THF (20 mL) in a flame-dried 100 mL flask equipped with a stir bar. DCC (DCC = N,N'-Dicyclohexylcarbodiimide) (443 mg, 2.15 mmol, 1 equiv) and HOBt*H_2O (HOBt = 1-hydroxybenzotriazole) (290 mg, 2.15 mmol, 1 equiv) was added

to the reaction mixture and stirred for pre-activation. After 20 minutes, NH_2-$(PEG)_3$-N_3 (412 mg, 2.36 mmol, 1.1 equiv) was added to the reaction mixture and stirred at room temperature. After 16 hours, DCU (DCU = Dicyclohexylurea) was filtered through Celite® and the filtrate was concentrated *in vacuo*. The residue was dissolved in EtOAc and the organic layer was washed with 5% $NaHCO_3$ (2 x 15 mL) and brine. The combined organic phases were dried over Na_2SO_4, filtered, evaporated *in vacuo*, and the crude product was purified by flash column chromatography (1% MeOH in DCM + 1% AcOH) to afford **S13** (1.31 g, 1.95 mmol) as an orange oil in 91% yield.

^{1}H NMR (400 MHz, DMSO-*d*$_6$) δ: 8.76 (t, *J* = 5.6 Hz, 1H), 8.22 (dd, *J* = 8.0, 1.4 Hz, 1H), 8.15 (dd, *J* = 8.1, 0.7 Hz, 1H), 7.82 (dd, *J* = 1.4, 0.8 Hz, 1H), 7.30 (t, *J* = 1.3 Hz, 2H), 6.95 (d, *J* = 1.3 Hz, 4H), 3.57 – 3.51 (m, 3H), 3.52 – 3.41 (m, 10H), 3.35 (p, *J* = 5.1, 4.6 Hz, 5H), 2.83 (hept, *J* = 7.0 Hz, 2H), 1.24 (d, *J* = 7.0 Hz, 13H).

^{13}C{^{1}H} NMR (101 MHz, DMSO-*d*$_6$) δ: 174.64, 172.05, 167.80, 164.47, 152.41, 152.33, 150.87, 141.01, 129.98, 129.41, 129.29, 127.57, 125.38, 122.34, 118.64, 118.59, 115.77, 115.73, 110.44, 81.23, 69.77, 69.75, 69.70, 69.57, 69.24, 68.68, 50.00, 33.38, 21.06, 18.57.

ESI$^+$-HRMS: calculated for $C_{37}H_{41}N_4O_{11}$ ($[M+H]^+$) 717.2766, found 717.2775.

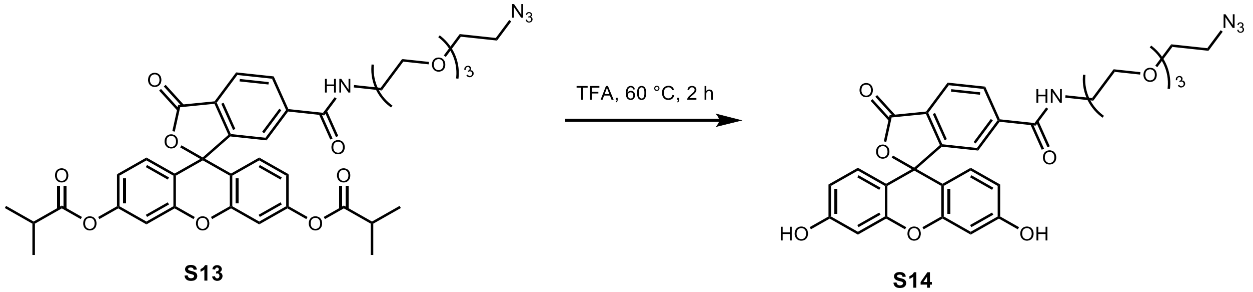

S13 (1.31 g, 1.95 mmol, 1 equiv) was dissolved in TFA (TFA = Trifluoroacetic acid) (20 mL) in a flame-dried 100 mL flask equipped with a stir bar. The reaction mixture was heated at 60 °C. After 2 hours, TFA was removed via N_2 and the orange oil was purified by flash column chromatography (5% MeOH in DCM + 1% AcOH) to afford **S14** (920 mg, 1.73 mmol) as an orange oil in 89% yield.

^{1}H NMR (400 MHz, DMSO-*d*$_6$) δ: 10.15 (s, 1H), 8.75 (t, *J* = 5.7 Hz, 1H), 8.17 (dd, *J* = 8.0, 1.4 Hz, 1H), 8.10 – 8.04 (m, 1H), 7.69 (t, *J* = 1.1 Hz, 1H), 6.69 (d, *J* = 2.3 Hz, 2H), 6.65 – 6.48 (m, 5H), 3.57 – 3.51 (m, 3H), 3.50 – 3.43 (m, 10H), 3.35 (q, *J* = 5.3, 4.7 Hz, 4H).

^{13}C{^{1}H} NMR (101 MHz, DMSO-*d*$_6$) δ: 172.3, 164.8, 159.7, 152.0, 140.7, 129.4, 125.1, 112.9, 109.3, 102.4, 69.8, 69.6, 69.3, 68.7, 50.1, 40.1, 39.9, 39.7, 39.5, 39.3, 39.1, 39.0, 38.9, 21.2.

ESI$^+$-HRMS: calculated for $C_{29}H_{29}N_4O_9$ ($[M+H]^+$) 577.1929, found 577.1930.

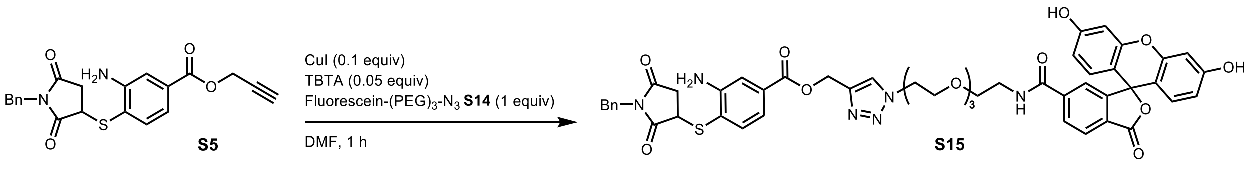

S14 (288 mg, 0.5 mmol, 1 equiv) was dissolved in DMF in a flame-dried 50 mL flask equipped with a stir bar. CuI (9.5 mg, 0.05 mmol, 0.1 equiv), TBTA (TBTA = Tris(benzyltriazolylmethyl)amine) (13 mg, 0.025 mmol, 0.05 equiv), and **S5** (197 mg, 0.5 mmol, 1 equiv) was added to the reaction mixture and the flask was sealed with a septum. After stirring for 1 hour, the reaction mixture was concentrated *in vacuo* and purified by flash column chromatography (5% MeOH in DCM) to afford **S15** (125 mg, 0.16 mmol) as a yellow oil in 88% yield.

^{1}H NMR (400 MHz, DMSO-d_6) δ: 8.73 (t, J = 5.5 Hz, 1H), 8.18 (s, 1H), 8.15 (dd, J = 8.0, 1.4 Hz, 1H), 8.06 (dd, J = 7.9, 0.7 Hz, 1H), 7.69 – 7.67 (m, 1H), 7.38 – 7.05 (m, 8H), 6.90 (dd, J = 8.0, 1.9 Hz, 1H), 6.69 (d, J = 2.2 Hz, 2H), 6.64 – 6.51 (m, 5H), 5.76 (d, J = 8.3 Hz, 2H), 5.41 – 5.27 (m, 2H), 4.56 – 4.41 (m, 4H), 4.32 (dd, J = 9.0, 3.9 Hz, 1H), 3.78 (t, J = 5.2 Hz, 2H), 3.46 – 3.38 (m, 14H), 3.39 – 3.31 (m, 15H), 3.20 (dd, J = 18.5, 9.1 Hz, 2H), 2.59 (dd, J = 18.5, 4.0 Hz, 1H).

^{13}C{^{1}H} NMR (101 MHz, DMSO-d_6) δ: 176.3, 175.2, 172.5, 168.5, 165.8, 165.1, 160.1, 152.3, 151.0, 142.1, 137.1, 129.7, 128.8, 128.7, 127.9, 125.8, 122.7, 122.0, 117.5, 116.9, 115.5, 113.23, 111.9, 109.6, 102.7, 70.1, 70.0, 69.9, 69.1, 58.3, 49.8, 43.3, 42.2, 21.6.

ESI$^+$-HRMS: calculated for $C_{50}H_{50}N_7O_{13}S$ ([M+NH$_4$]$^+$) 988.3182, found 998.3202.

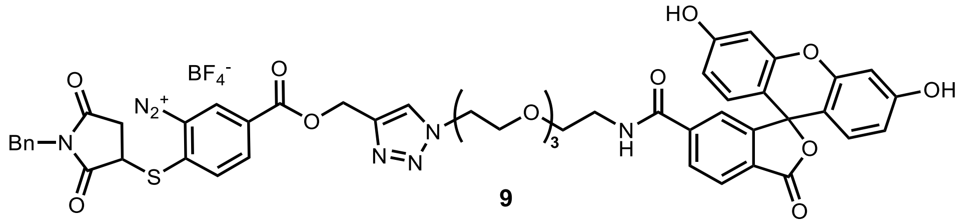

9

S15 (100 mg, 0.1 mmol, 1 equiv) was dissolved in acetone in a flame-dried 25 mL flask equipped with a stir bar. Fluoroboric acid (66 µL, 1.03 mmol, 10 equiv) was added to the reaction mixture dropwise. Then, the reaction mixture was cooled to 0 °C and tert-Butyl nitrite (74 µL, 0.62 mmol, 6 equiv) was added dropwise *via* syringe. After stirring at 0 °C for 1 hour, 6 mL of Et_2O was added to precipitate out yellow solids. The reaction was filtered using a Buchner funnel to obtain **9** (36 mg, 34 µmol) as a yellow solid in 33% yield.

^{1}H NMR (400 MHz, DMSO-d_6) δ: 9.31 (d, J = 2.0 Hz, 1H), 8.74 (d, J = 5.7 Hz, 1H), 8.52 (dd, J = 8.5, 2.0 Hz, 1H), 8.29 (d, J = 8.6 Hz, 1H), 8.25 (s, 1H), 8.16 (dd, J = 8.1, 1.4 Hz, 1H), 8.08 (d, J = 8.0 Hz, 1H), 7.69 (s, 2H), 7.37 – 7.21 (m, 9H), 6.70 (d, J = 2.2 Hz, 4H), 6.62 – 6.51 (m, 8H), 5.49 (d, J = 4.9 Hz, 2H), 5.11 (dd, J = 9.3, 5.0 Hz, 1H), 4.59 (s, 3H), 4.53 (h, J = 5.1 Hz, 4H), 3.79 (s, 18H), 3.43 (ddt, J = 15.3, 12.1, 6.3 Hz, 20H), 3.35 (dd, J = 7.4, 4.1 Hz, 4H), 3.05 (dd, J = 18.3, 5.0 Hz, 1H).

^{13}C{^{1}H} NMR (101 MHz, DMSO-d_6) δ: 168.08, 164.63, 159.66, 151.87, 140.57, 129.28, 128.54, 127.81, 122.31, 112.79, 109.19, 102.27, 69.51, 68.61, 49.45.

^{19}F{^{1}H} NMR (471 MHz, DMSO-d_6) δ: -148.21, -148.27.

ESI$^+$-HRMS: calculated for $C_{51}H_{46}N_7O_{13}S$ (M$^+$) 996.2869, found 996.2761.

Synthesis of Mesodiphenylhelianthrene (MDH):

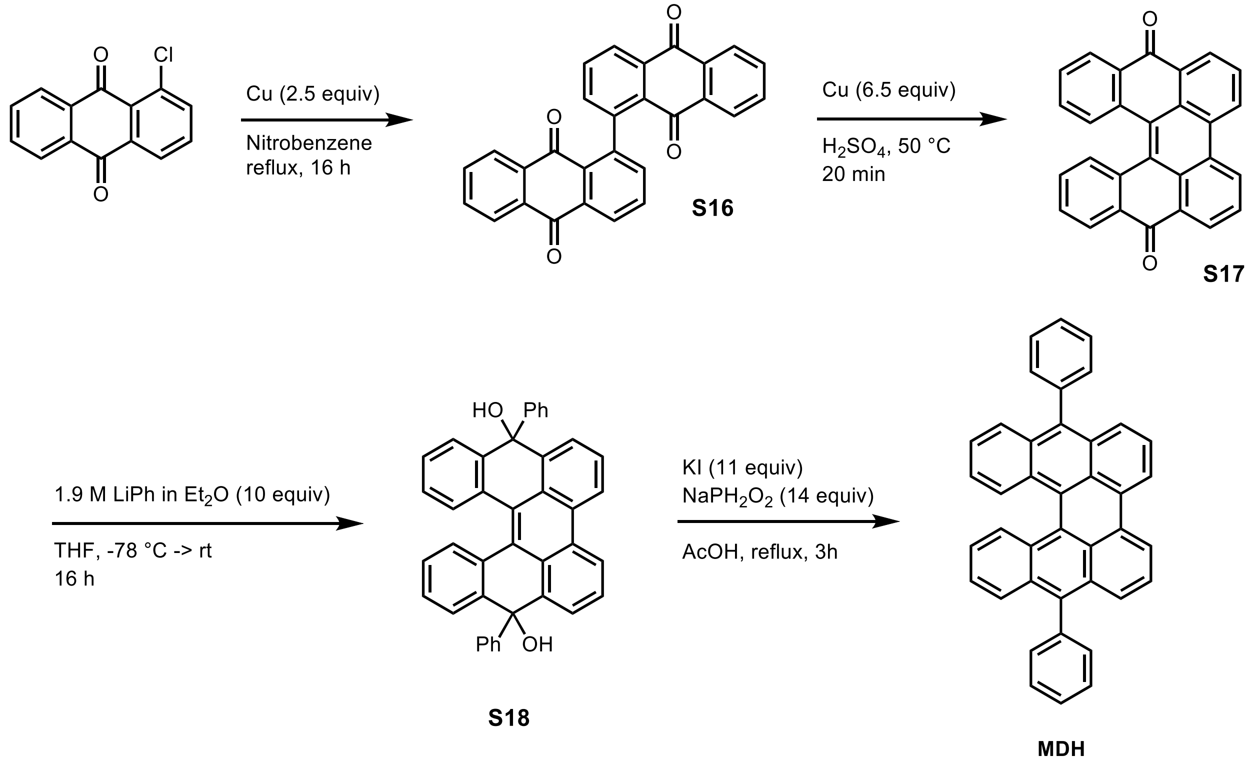

1-Chloroanthraquinone was dissolved in nitrobenzene (25 mL) in a flame-dried 100 mL flask equipped with a stir bar. The yellow solution was heated to 210 °C for 15 minutes to remove any water inside the solution. Copper (2.5 equiv) was added to the solution and the reaction was refluxed for 3 hours. After cooling the reaction to room temperature, the brown solution was added to 20 mL of toluene. This suspension was filtered and washed with additional 100 mL of toluene. The yellow-green powder was then added to 500 mL of 0.1 M HNO_3 and stirred until there was no gas evolution. This suspension was then filtered, washed with 300 mL of H2O and 300 mL of MeOH to give the crude product **S16**, which was forwarded to the next step without purification. Crude [1,1'-Bianthracene]-9,9',10,10'-tetrone **S16** was added to a 50 °C solution of concentrated H_2SO_4 along with copper (6.5 equiv). After 20 minutes, the resulting blue solution was poured into 200 mL of ice water to give a brown precipitate. The brown precipitate was filtered and then suspended in 150 mL of xylene. The suspension was heated to a boil and was filtered through Celite to remove copper. The filtrate was concentrated to obtain crude orange powder **S17**, which was forwarded to the next step without purification. Helianthrone **S17** was dissolved in THF and the reaction was cooled to -78 °C. Phenyl lithium solution (1.9 M in n-butyl ether, 10 equiv) was added to the reaction dropwise under N_2 and darkness. After 16 hours, the reaction was brought to room temperature and NH_4Cl was added to quench the reaction solution. After adding additional H_2O, the THF was mostly concentrated and the solution was extracted with DCM, washed with water and brine, dried with Na_2SO_4 and concentrated to afford a crude brown powder. This crude product was purified via FCC (100% DCM) to afford the product **S18** as a yellow powder. The diol **S18** was then suspended in acetic acid in a flame-dried 25 mL flask under dark and N_2. To this suspension, KI (11 equiv) and $NaPH_2O_2$ (14 equiv) were added, and the reaction was refluxed for 3 hours. After cooling to room temperature, the dark purple solution was filtered and washed with water and methanol to give pure **MDH** as a dark purple powder. The NMR spectra are consistent with the reported literature.[6]

^{1}H NMR (400 MHz, Benzene-d6) δ: 7.98 (d, J = 7.1 Hz, 2H), 7.85 (dd, J = 8.6, 2.5 Hz, 4H), 7.81 (d, J = 8.8 Hz, 2H), 7.46 (d, J = 7.2 Hz, 2H), 7.40 – 7.33 (m, 6H), 7.33 – 7.26 (m, 4H), 7.11 – 7.06 (m, 2H), 6.95 (ddd, J = 8.9, 6.4, 1.3 Hz, 2H).

3. Synthetic Procedure for Peptide 2

The peptide used in this study was synthesized through Fmoc-solid phase peptide synthesis method. Peptides were synthesized on rink-amide resins using DMF as solvents. Piperidine (25% by volume) was used to deprotect the Fmoc group followed by amino acid coupling using HATU as the coupling reagents in the presence of DIPEA (DIPEA = Diisopropylethylamine). HATU (HATU = Hexafluorophosphate Azabenzotriazole Tetramethyl Uronium) and Fmoc (Fmoc = Fluorenylmethyloxycarbonyl) protected amino acids were added in 5 equivalents and DIPEA in 10 equivalents to the amino groups on the resin. Cocktail solution containing trifluoroacetic acid (TFA/triisopropylsilane/water/ethanedithiol (91/3/3/3) by volume) was prepared to cleave all peptides from the rink-amide resin. After 2 hrs, solid resin was removed by gravity filtration and TFA was evaporated. Cold diethyl ether was added to the concentrated TFA solution to precipitate the peptides which were centrifuged and thoroughly washed with diethyl ether four times. Residual diethyl ether was removed under vacuum. Purification was performed by reversed phase HPLC using Luna® C18 column (250×21.2mm, 10 μm) flow rate = 15.0 mL/min, with an isocratic eluent 40:60 v/v (0.1% v/v FA in MeCN : 0.1% FA TFA in H_2O) over 10 min, followed by 50:50 v/v (0.1% v/v TFA in MeCN : 0.1% v/v TFA in H_2O) over 2 minutes, followed by 65:35 v/v (0.1% v/v TFA in MeCN : 0.1% v/v TFA in H_2O) over 5 minutes, then 100:0 v/v (0.1% v/v TFA in MeCN : 0.1% v/v TFA in H_2O) over 5 minutes. The fractions containing the desired peptide were collected and lyophilized to afford the title compound **2** as a colorless powder (120 mg, 0.114 mmol, 19% yield).

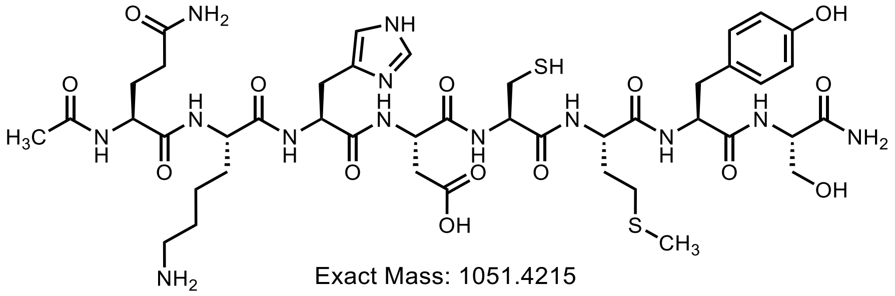

Gln-Lys-His-Asp-Cys-Met-Tyr-Ser (2).

ESI$^+$-HRMS: calculated for: $C_{43}H_{65}N_{13}O_{14}S_2$ ($[M+H]^+$), 1052.4288; found, 1052.4180.

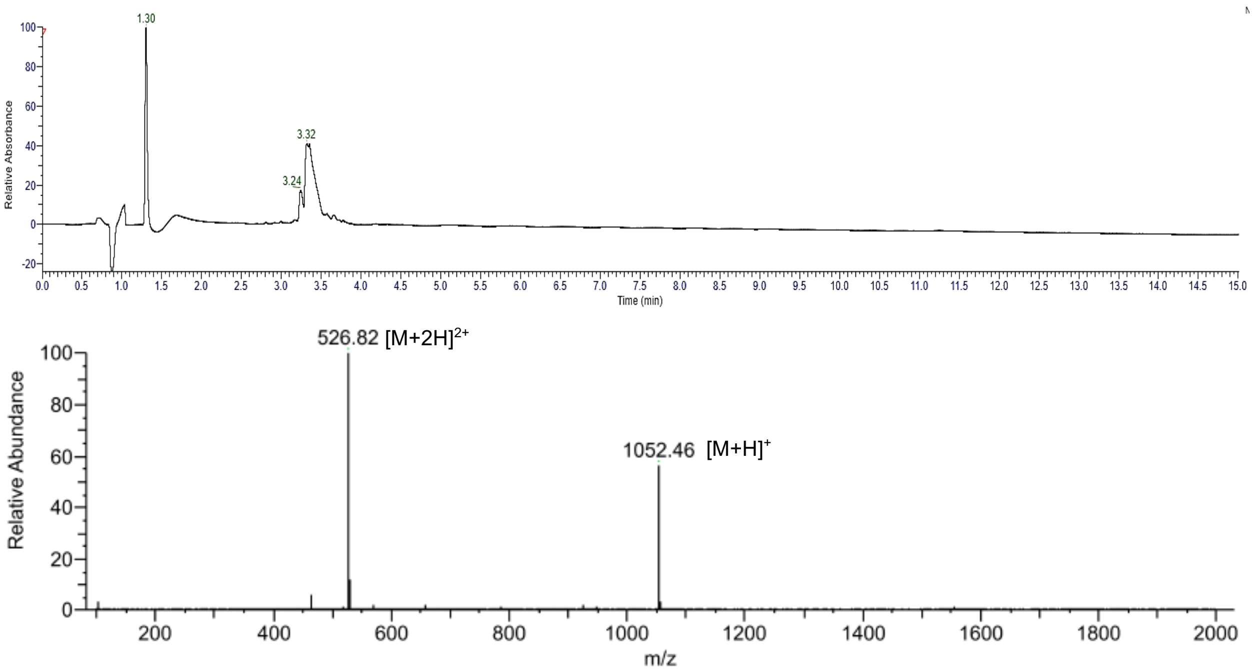

Figure S1. LCMS and ESI-TOF for Peptide **2**.

4. Peptide Modification and Product Characterization

4.1. General Procedures and Characterization Methods

<u>LC-MS method for analysis of peptide conjugation:</u>
LC-MS was performed on a Thermo Scientific TSQ Fortis Plus Triple Quadrupole mass spectrometer coupled to a Zorbax RRHD Eclipse Plus C18 column (95 Å, 1.8 µm, 3.0 x 100 mm). Solvents A, water with 0.1% formic acid and B, acetonitrile with 0.1% formic acid were used as the mobile phase with a flow rate of 0.60 mL/min. The gradient was programmed as follows: 95% A for 13 minutes, then 60% A for 2 minutes. *m/z* values of positively and negatively charged ions were measured in the mass analyzer, which was scanned between *m/z* 100–2000 for the generation of mass spectra. The major peak(s) were selected for integration and analyzed using Freestyle software (v. 1.8 SP2 from Thermo Scientific).

<u>General Procedure 1 for the reaction with peptides (GP1):</u>
1 (3.0 mg, 6 µmol, 3 equiv), peptide **2** (2 mg, 2 µmol, 1 equiv), and **PC** (5 mol%), where applicable, were dissolved in water (2 mL) in a 4-mL clear glass vial equipped with a stir bar. The reaction was then irradiated in between either two 525 nm Kessil lamps (44 W), two 660 nm Kessil lamps (35 W), or in ambient light, at room temperature for 10-30 minutes. After the allotted irradiation time, the samples were filtered through glass filter paper and were subjected to analysis via LC-MS.

<u>General Procedure 2 for the reaction with peptides (GP2):</u>
1 (3-10 equiv) and peptide **2** (2 mg, 2 µmol, 1 equiv) were dissolved in 1X PBS (2 mL) in a 4-mL clear glass vial equipped with a stir bar. The reaction was then irradiated at room temperature in

between two 660 nm Kessil lamps (35 W) equipped with fans. After 30 minutes, the samples were filtered through glass filter paper and were subjected to analysis via LC-MS.

General Procedure 3 for the reaction with peptides (GP3):
1 (3.0 mg, 6 µmol, 3 equiv), and peptide **2** (2 mg, 2 µmol, 1 equiv) were dissolved in water (2 mL) in a 4-mL clear glass vial equipped with a stir bar. The vial was wrapped with aluminum foil to protect from light. The vial was placed in a heating block and was stirred at either 50 °C or 60 °C. After the allotted reaction time, the samples were filtered through glass filter paper and were subjected to analysis via LC-MS.

Table S1. Evaluation of Photocatalysts and Irradiation for Disulfide Formation.

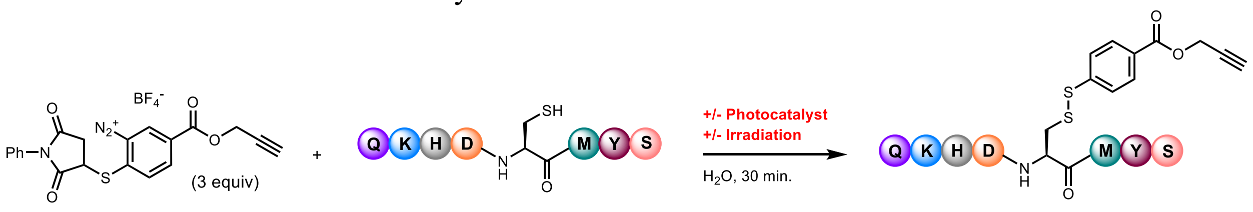

Entry	Photocatalyst (5 mol%)	Wavelength	Yield of **3** (%)
1	None	525 nm	>99
2	Eosin Y	525 nm	>99
3	(Ir[dF(CF$_3$)ppy]$_2$(dtbpy))PF$_6$	525 nm	>99
4	New Methylene Blue	525 nm	32
5	New Methylene Blue	660 nm	>99
6	None	660 nm	>99
7	None	1040 nm	>99
8	None	None	<0

Reactions were carried out *via* GP1 for 30 minutes.

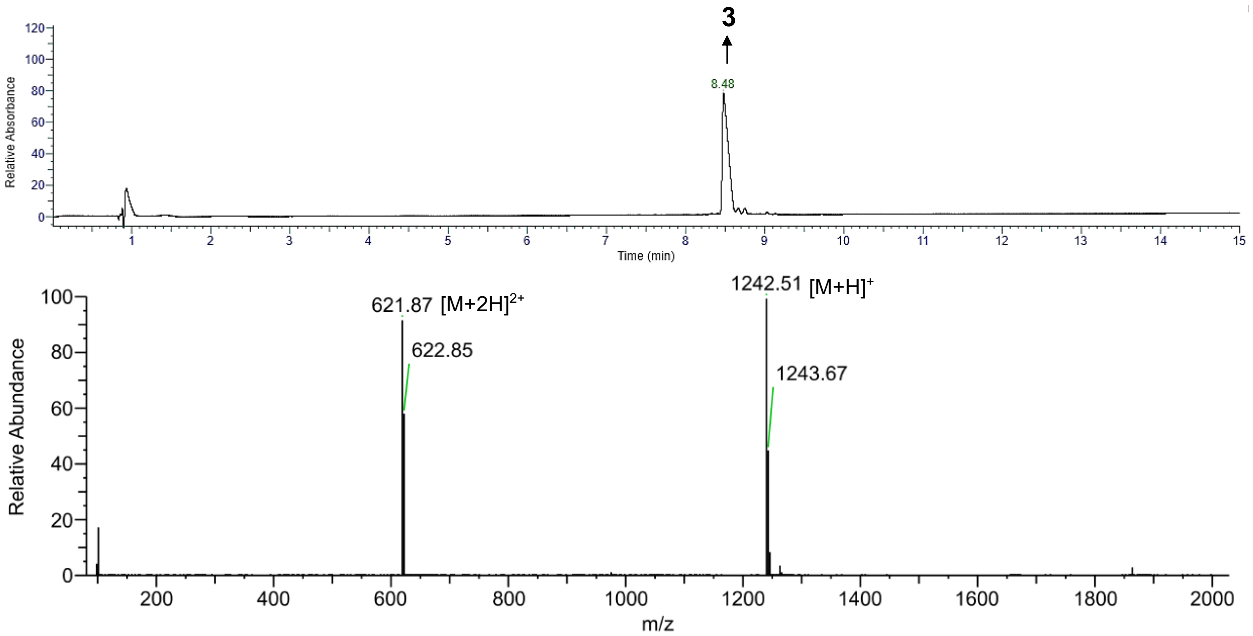

Figure S2. LCMS and ESI-TOF for Entry 1 in **Table S1**.

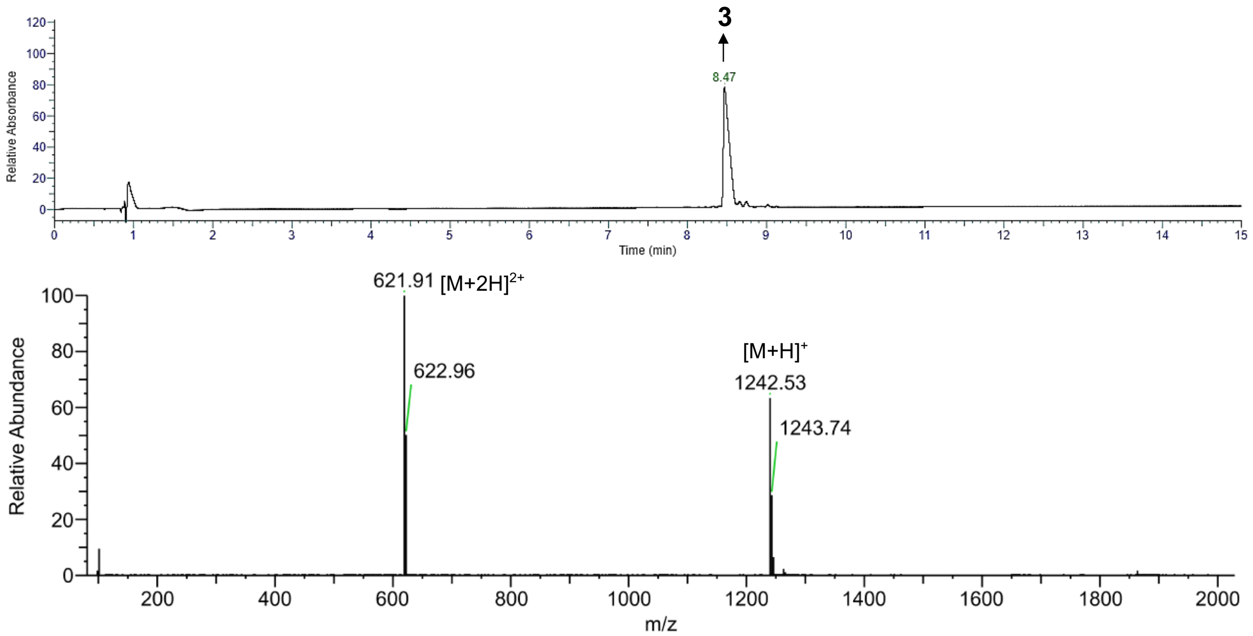

Figure S3. LCMS and ESI-TOF for Entry 2 in **Table S1**.

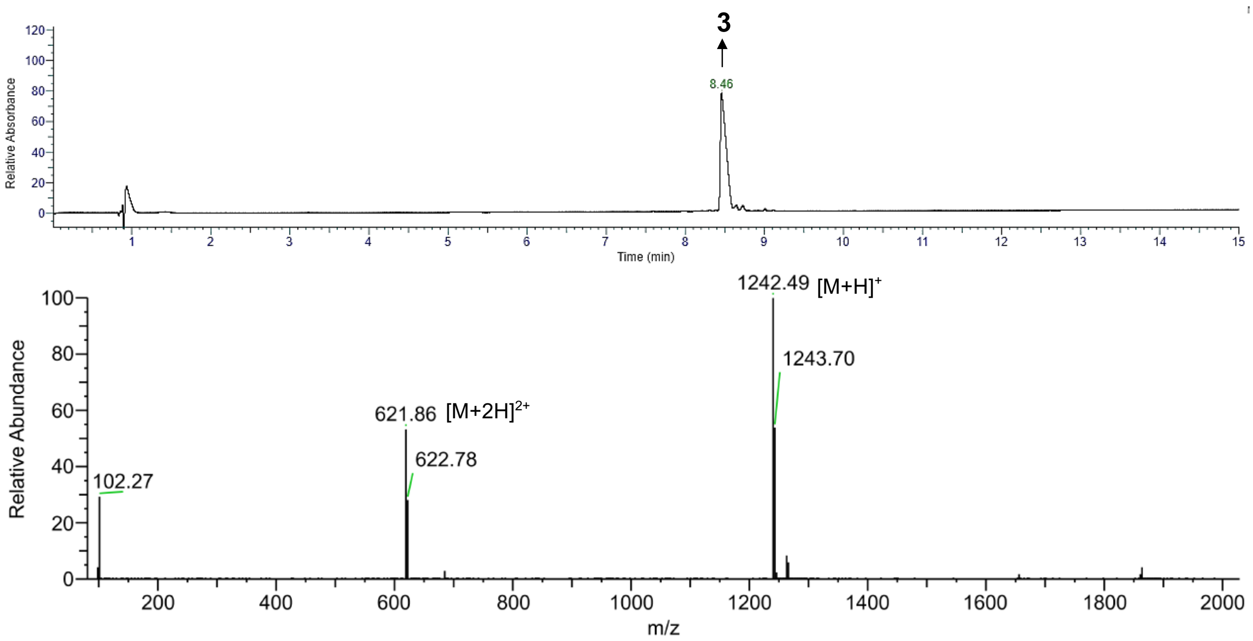

Figure S4. LCMS and ESI-TOF for Entry 3 in **Table S1**.

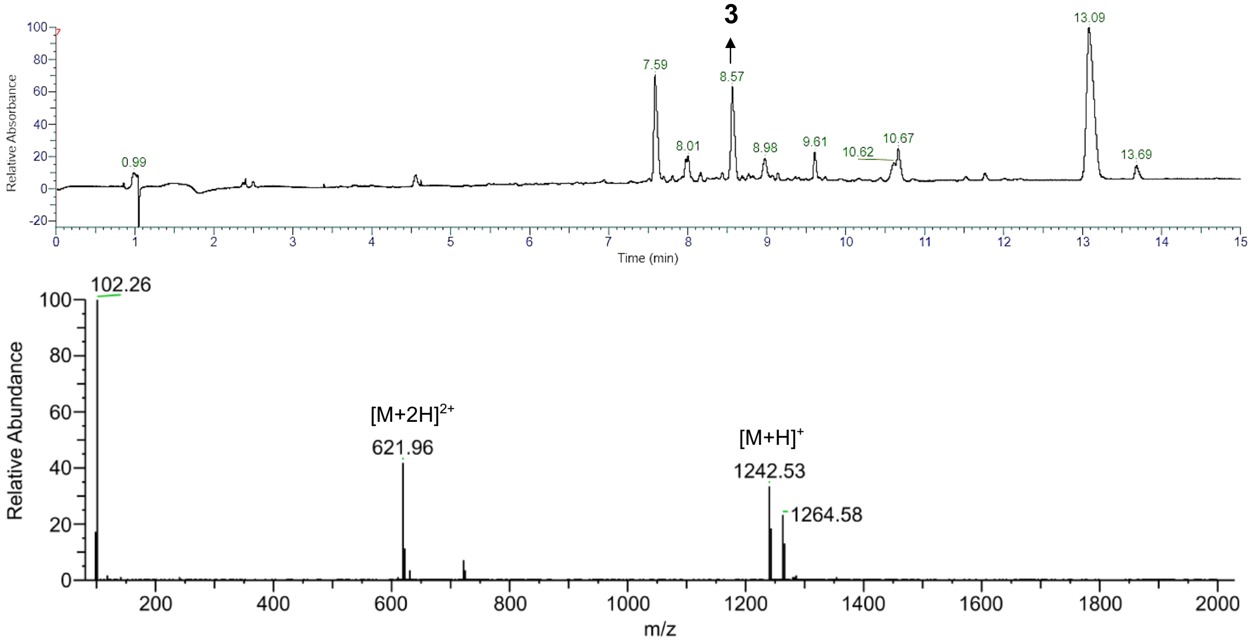

Figure S5. LCMS and ESI-TOF for Entry 4 in **Table S1**.

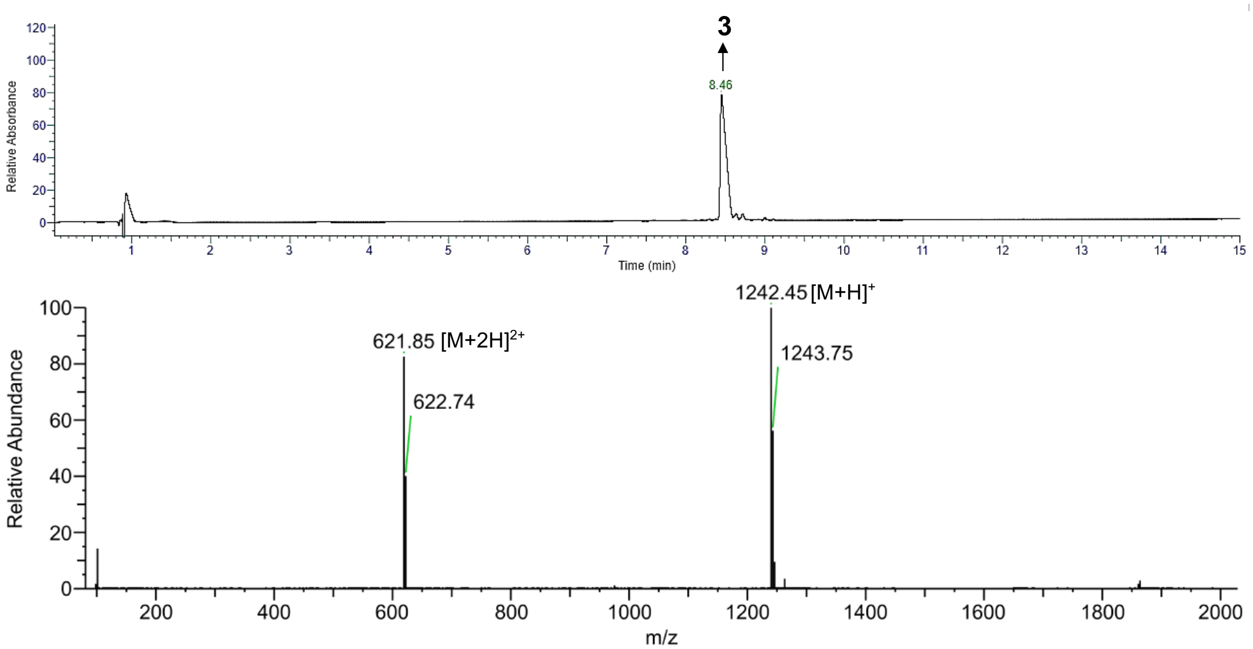

Figure S6. LCMS and ESI-TOF for Entry 5 in **Table S1**.

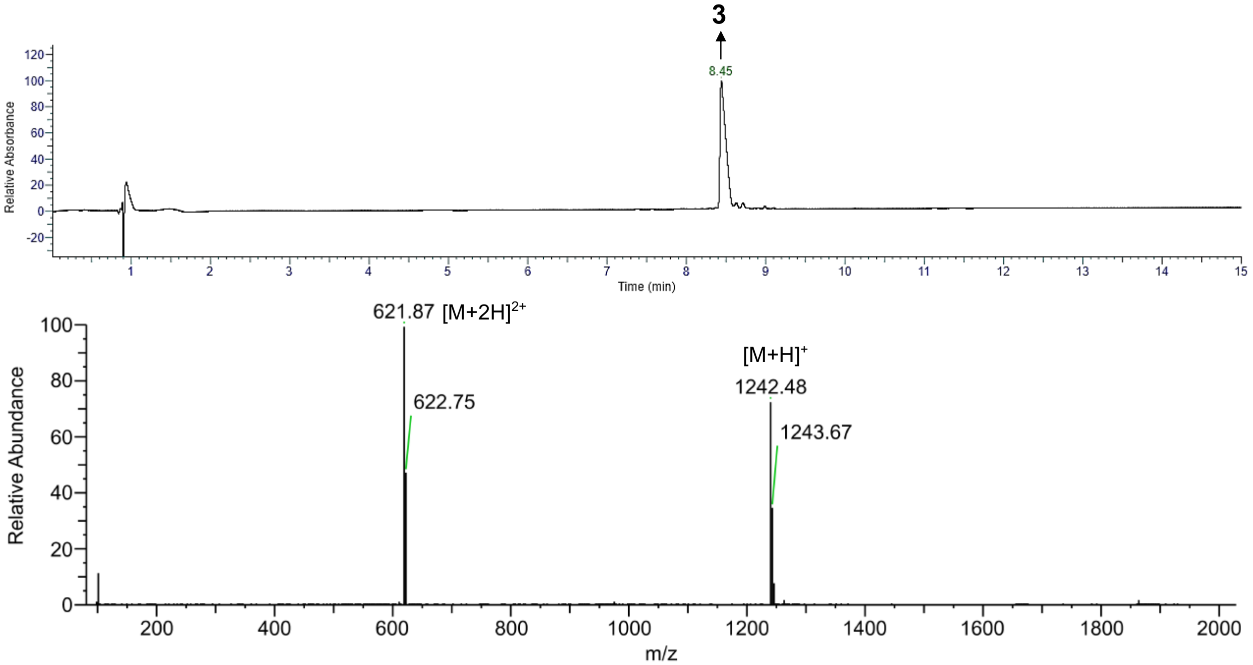

Figure S7. LCMS and ESI-TOF for Entry 6 in **Table S1**.

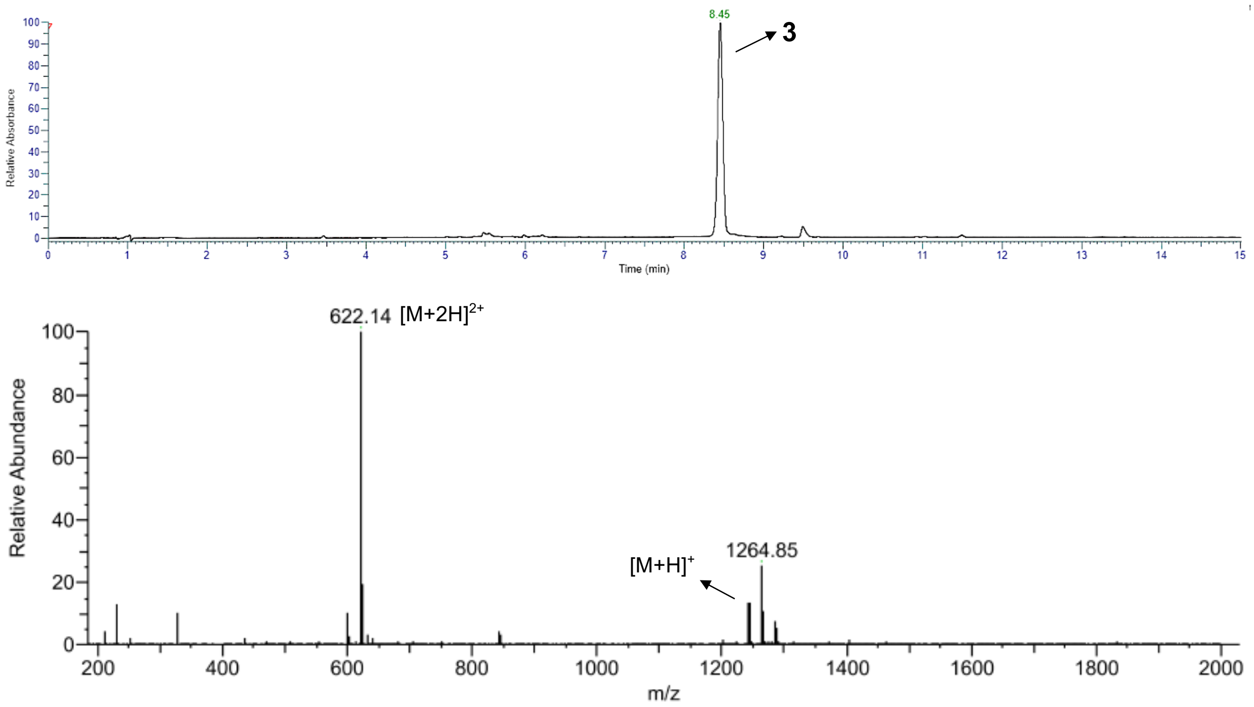

Figure S8. LCMS and ESI-TOF for Entry 7 in **Table S1**.

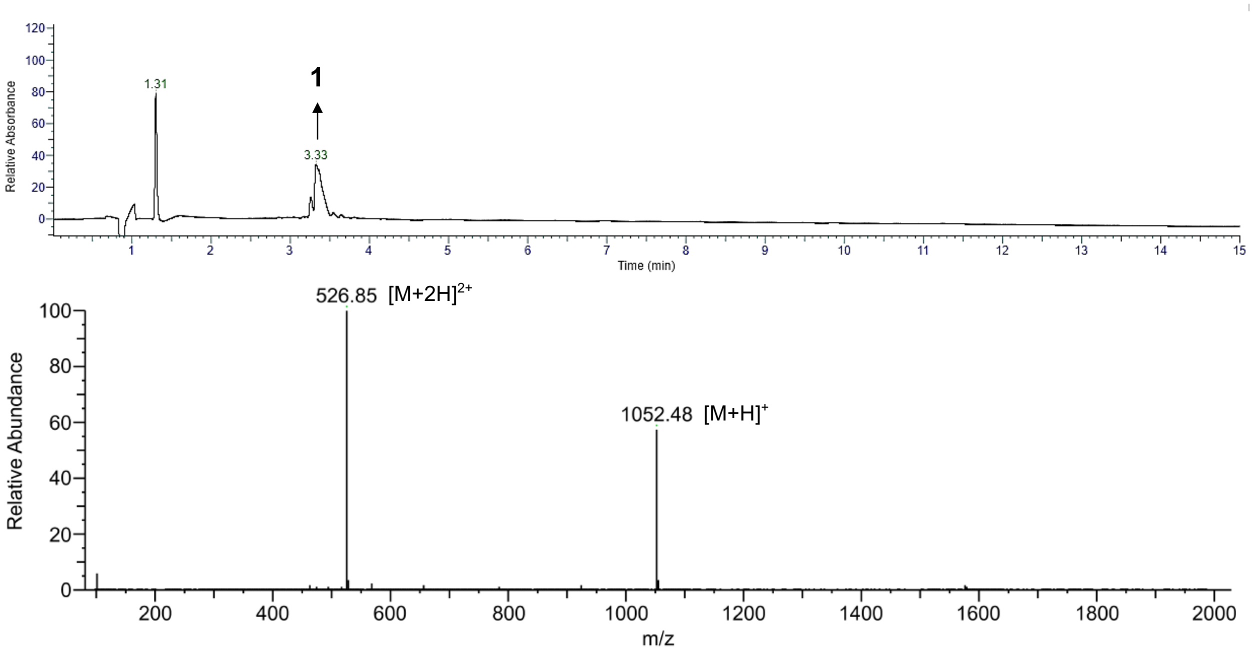

Figure S9. LCMS and ESI-TOF for Entry 8 in **Table S1**.

Table S2. Evaluation of Reaction Time.

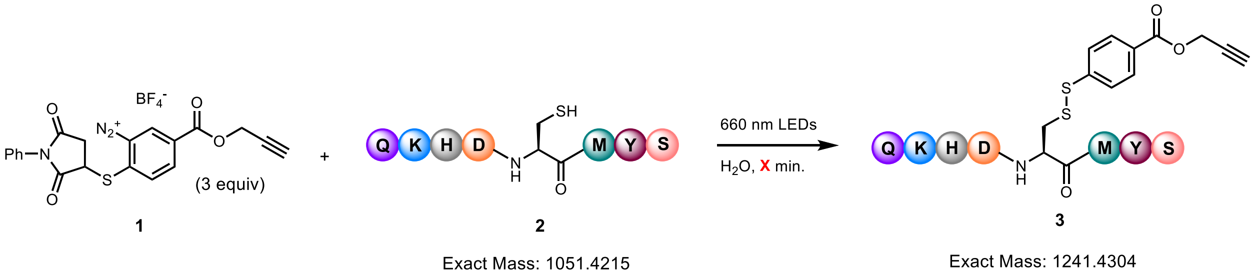

Entry	Reaction Time	Yield of **3** (%)
1	10 minutes	68
2	20 minutes	>99
Reactions were carried out *via* GP1.		

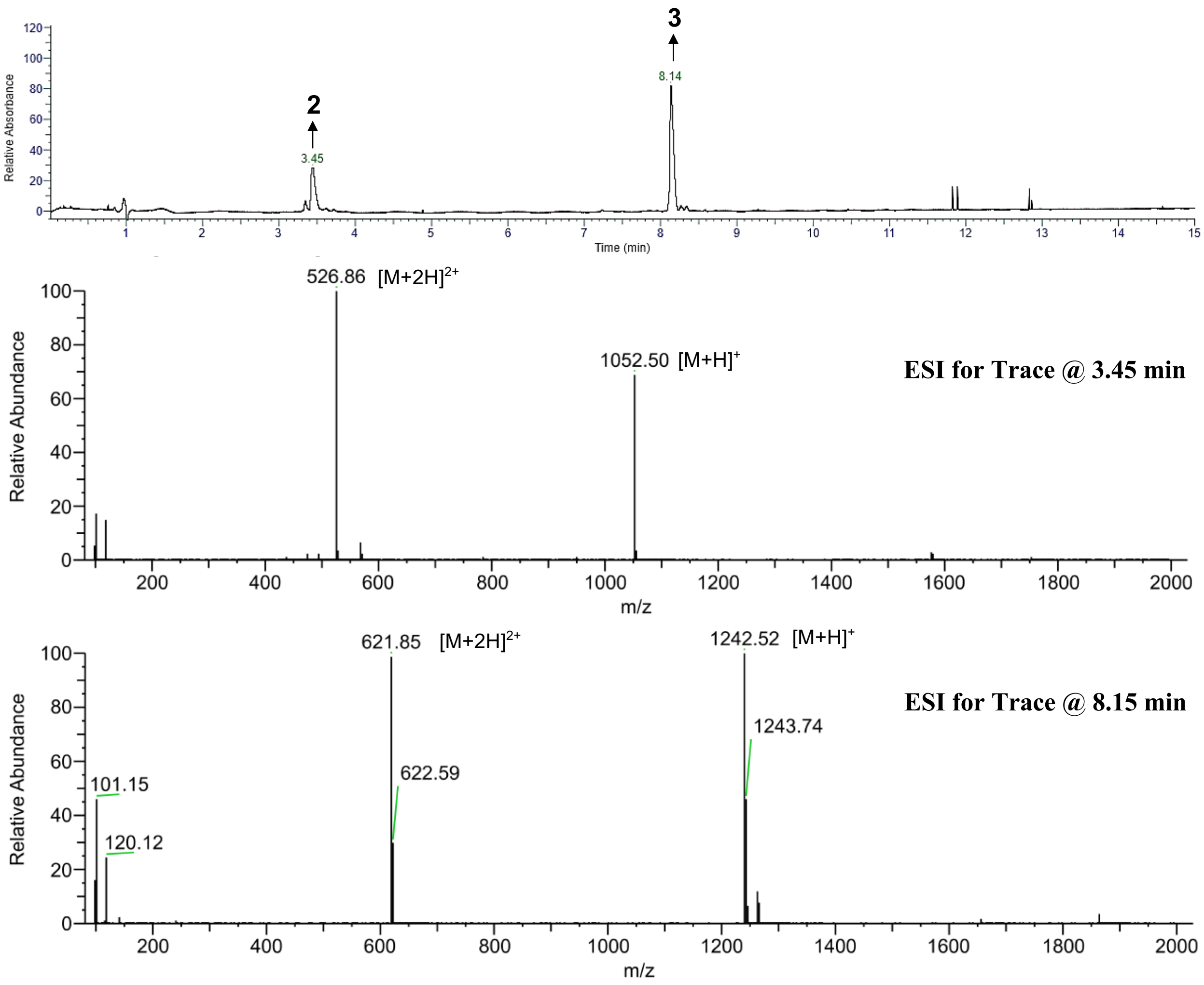

Figure S10. LCMS and ESI-TOF (at 3.45 and 8.15 minutes) for Entry 1 in **Table S2**.

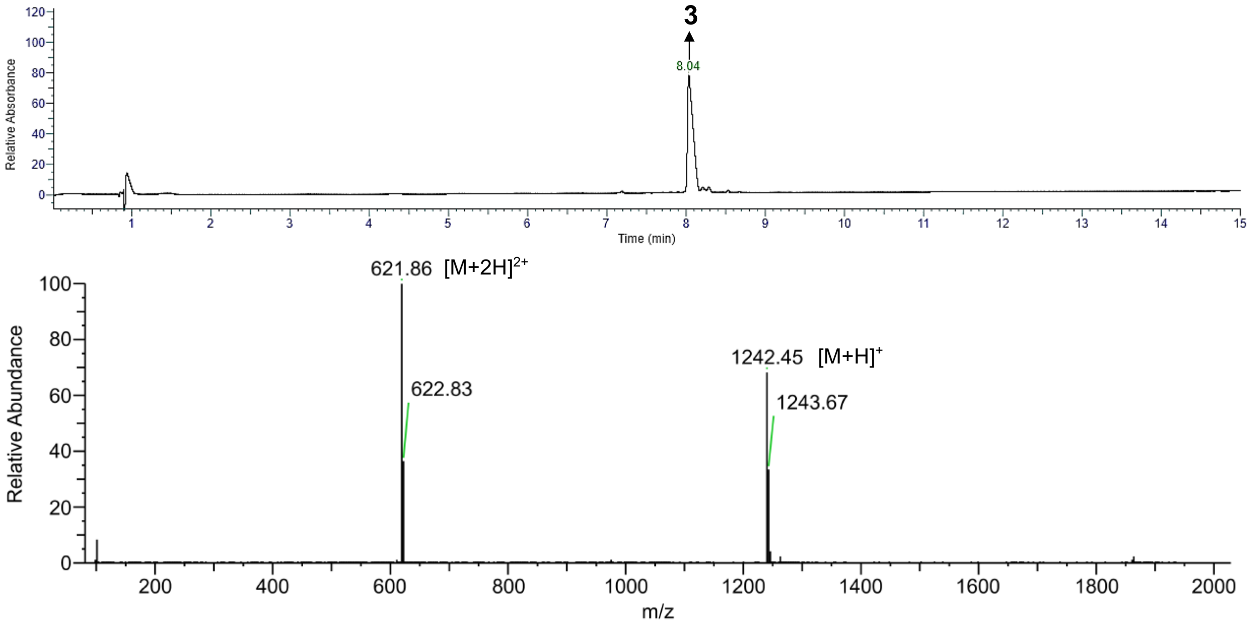

Figure S11. LCMS and ESI-TOF for Entry 2 in **Table S2**.

4.2. Stability Studies of 1 in Buffers

Table S3. Evaluation of Buffer pH.

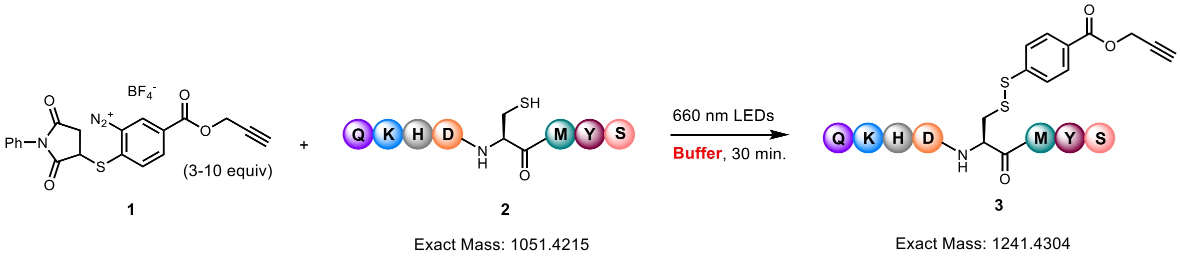

Entry	Buffer	pH	Yield of **3** (%)
1	1X PBS	6.2	>99
2	1X PBS	6.7	>99
3	1X PBS	6.9	>99
Reactions were carried out *via* GP2 listed above.			

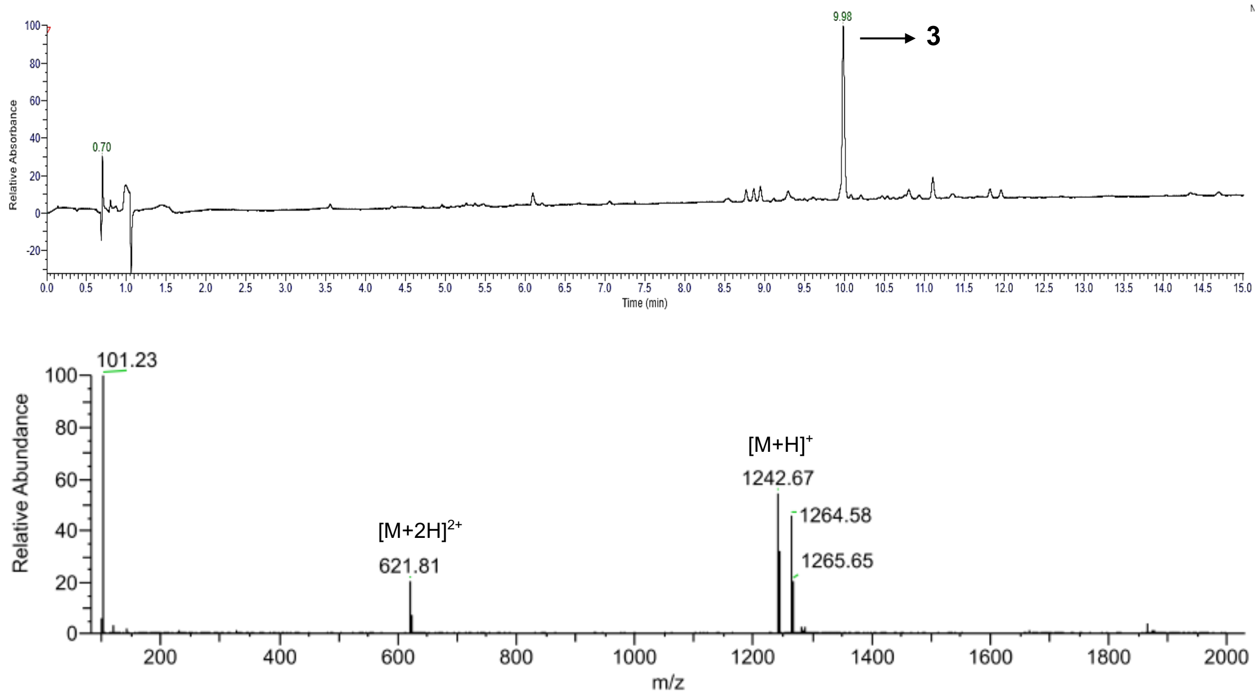

Figure S12. LCMS and ESI-TOF for Entry 1 in **Table S3**.

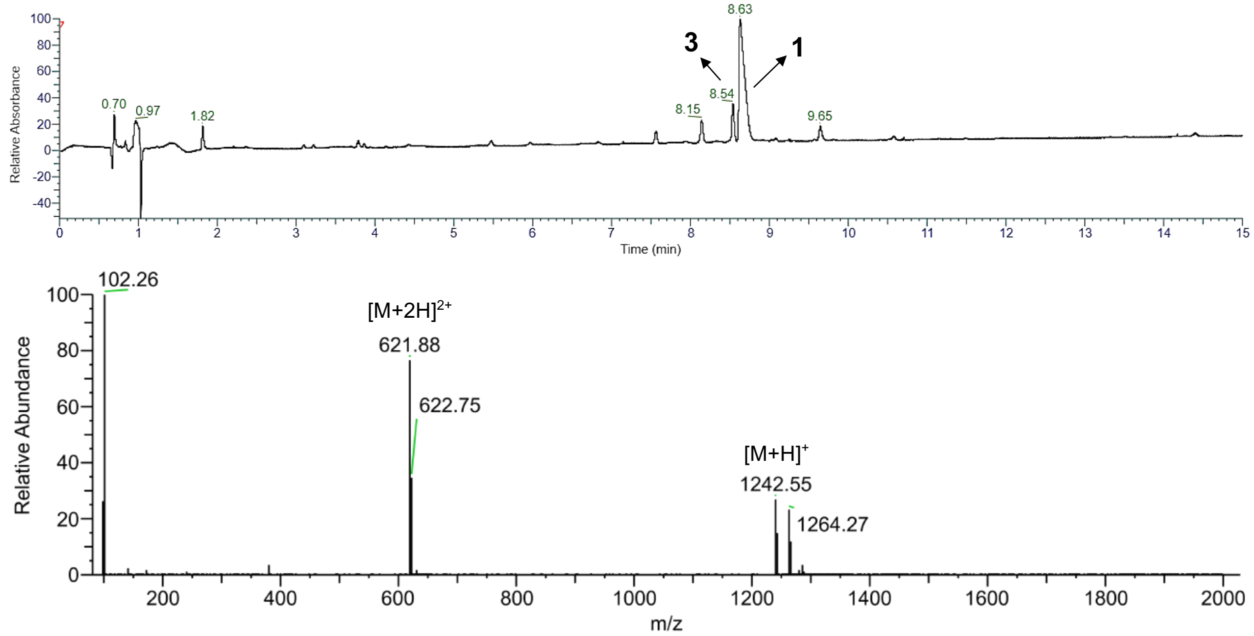

Figure S13. LCMS and ESI-TOF for Entry 2 in **Table S3**.

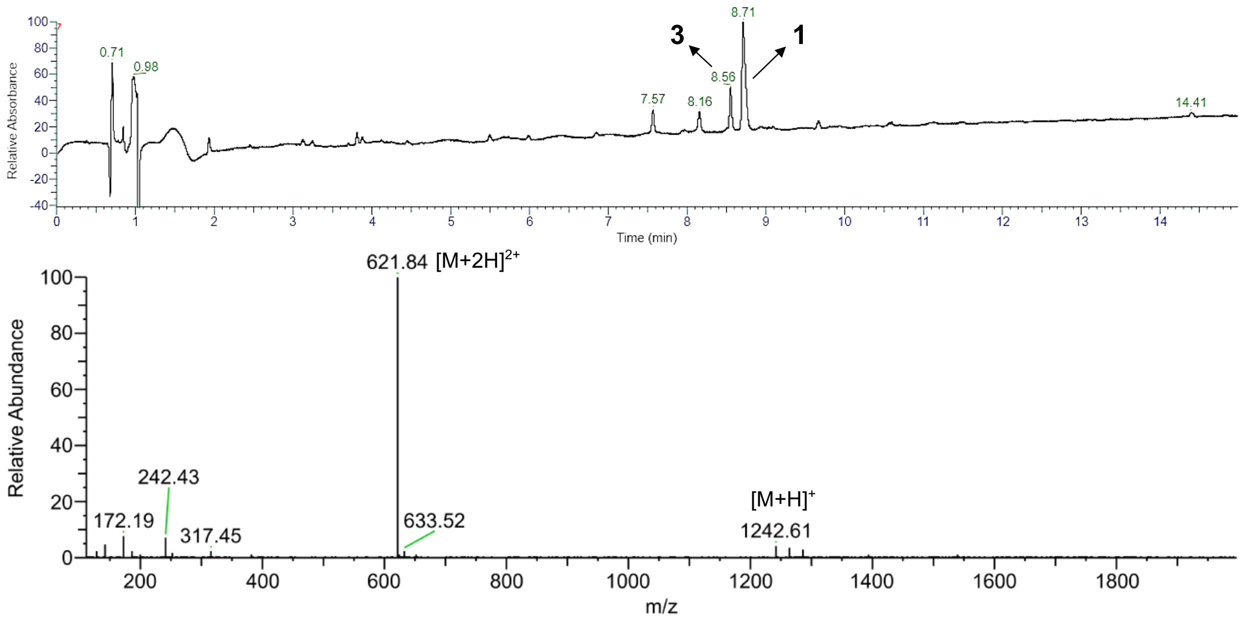

Figure S14. LCMS and ESI-TOF for Entry 3 in **Table S3**.

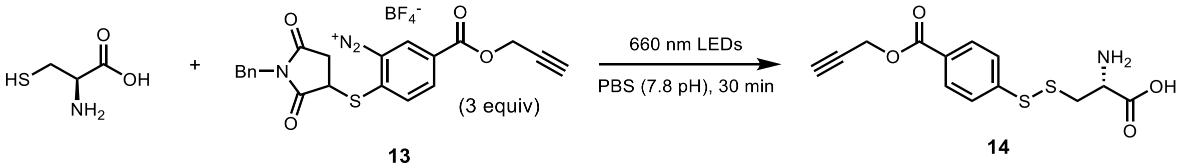

13

14

Exact Mass: 311.03

To a 4-mL clear glass vial, was added 2.0 mL of a 1 mM stock solution of *L*-cysteine in 7.8 pH PBS (1 equiv), **13** (3.0 mg, 6 µmol, 3 equiv), 1.0 mL of PBS (7.8 pH), and a stir bar. The reaction mixture was irradiated with two 660 nm Kessil lamps (35 W) while stirring at room temperature for 30 minutes. The samples were then filtered through glass filter paper and were subjected to analysis via LC/MS.

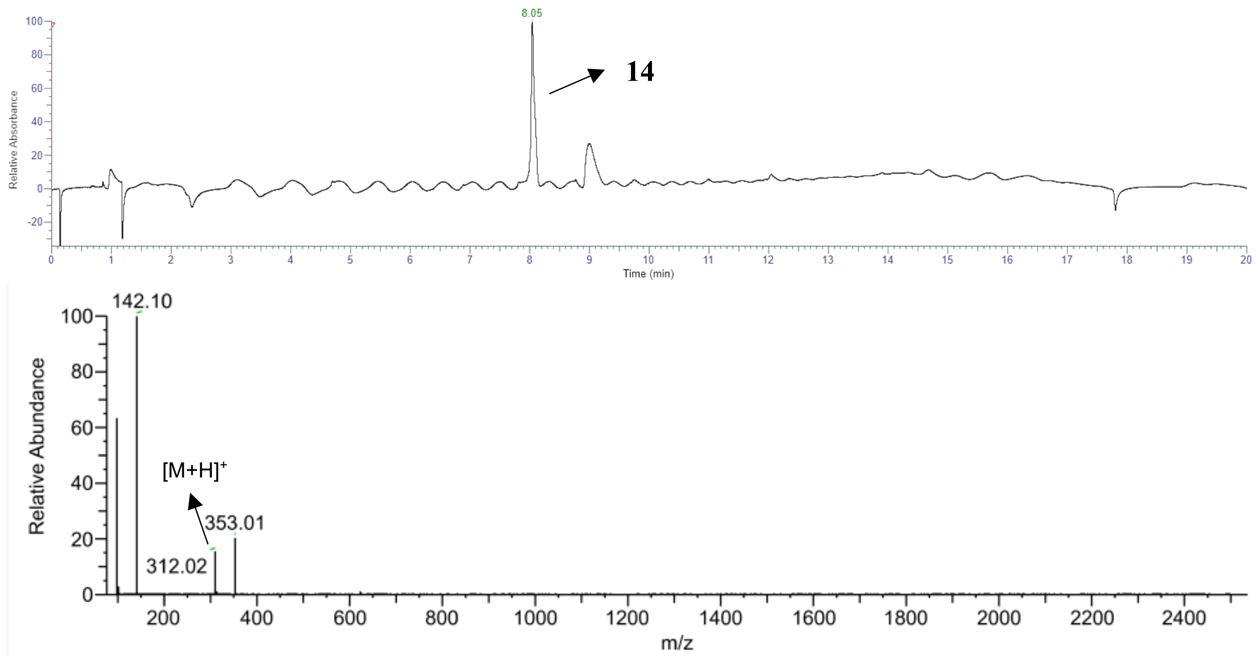

Figure S15. LCMS and ESI-TOF for the reaction between *L*-cysteine and **13** at 7.8 pH PBS buffer.

4.3. Control Experiments at Elevated Temperatures in the Dark

Table S4. Reactions at Different Temperatures

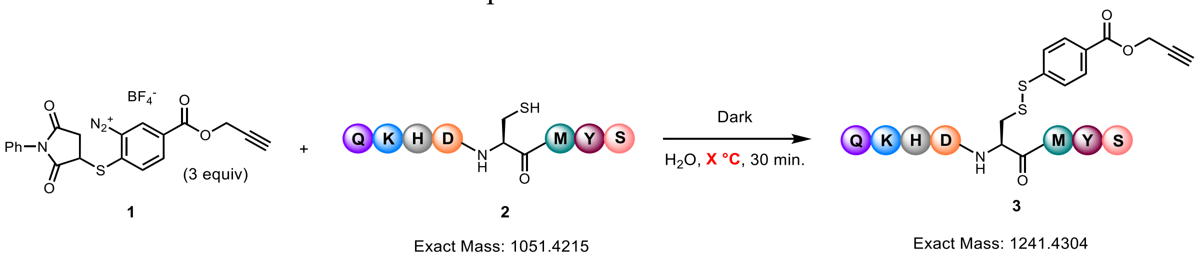

Entry	Temperature (°C)	Yield of **3** (%)
1	50	n.r.
2	60	n.r.
Reactions were carried out via GP3 listed above.		

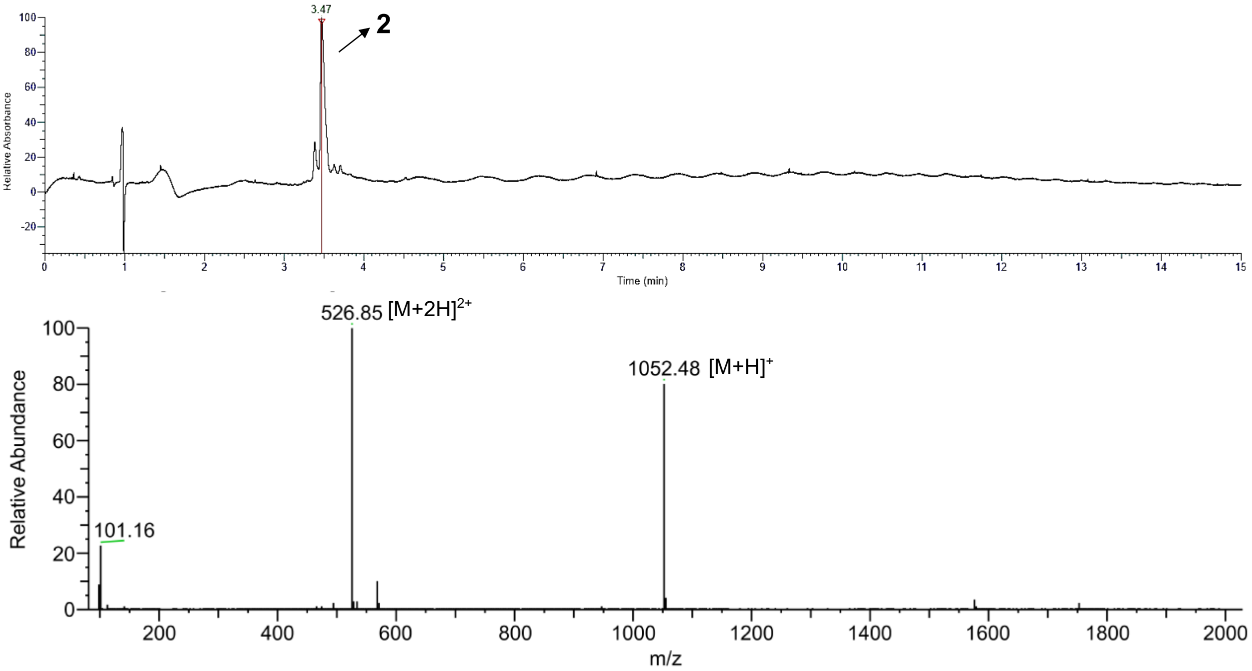

Figure S16. LCMS and ESI-TOF for Entry 1 in **Table S4**.

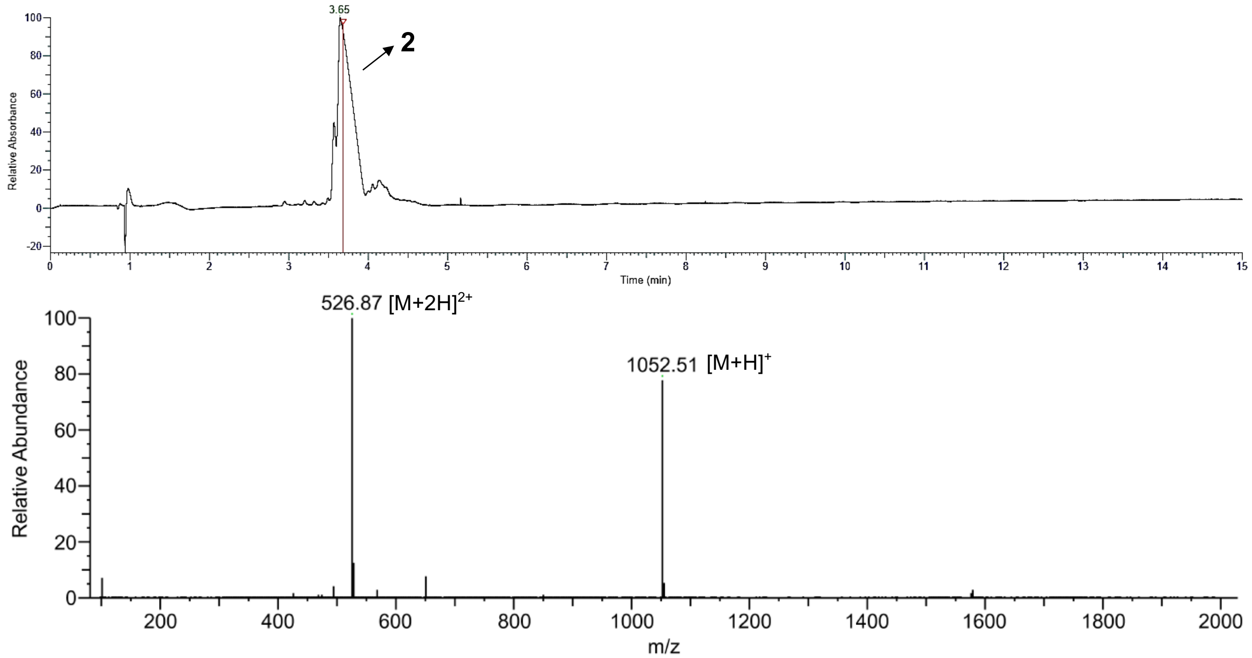

Figure S17. LCMS and ESI-TOF for Entry 2 in **Table S4**.

4.4. Reduction of Modified Peptide 3 with TCEP

1 (3.0 mg, 6 µmol, 3 equiv) and peptide **2** (2 mg, 2 µmol, 1 equiv) were dissolved in water (2 mL) in a 4-mL vial equipped with a stir bar. The reaction was then irradiated with two 660 nm Kessil lamps (35 W) at room temperature. After 30 minutes, the reactions were filtered through glass filter paper to obtain a crude mixture of **3**. The crude mixture was charged with a stir bar and 40 µL of TCEP (0.5 M in H_2O). Afte 10 minutes, 50 µL of crude reaction was pipetted into a 2 mL vial and diluted with 1 mL of H_2O for LC-MS analysis.

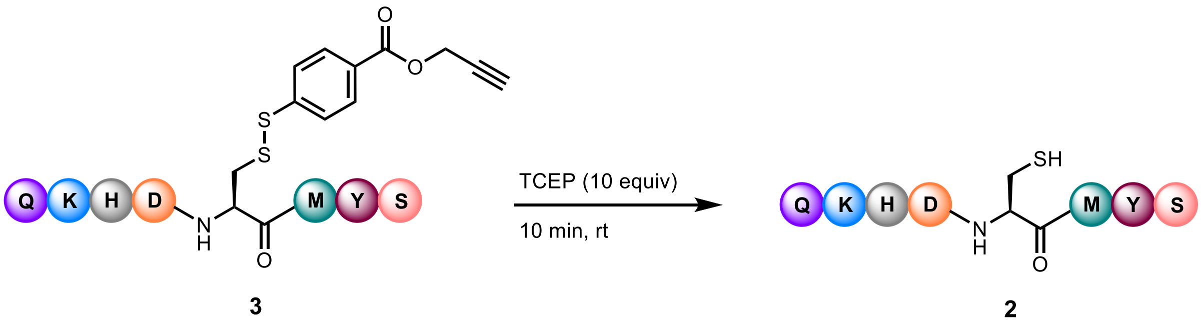

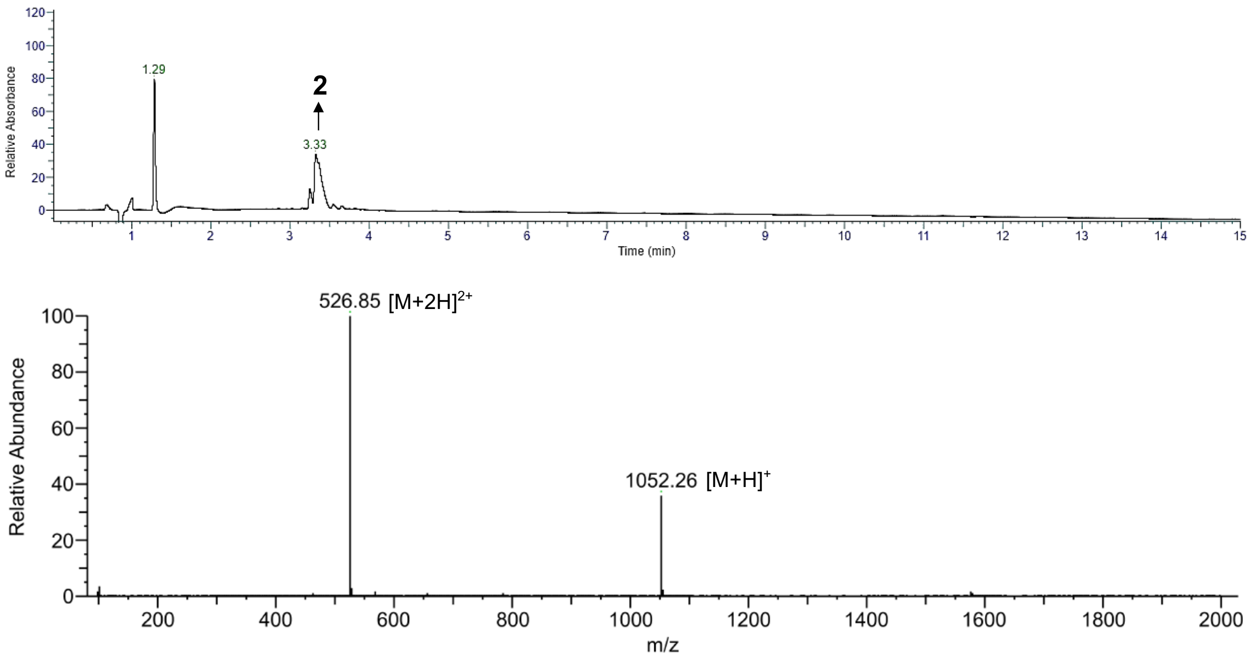

Figure S18. LCMS and ESI-TOF for the reaction of **3** and TCEP.

4.5. Biotinylation of Peptides 2 and 3

<u>Addition of Biotin to TagC-Red-modified-peptide **3** via Click Chemistry with Biotin-(PEG)$_2$-N$_3$</u>: **1** (3.0 mg, 6 µmol, 3 equiv) and peptide **2** (2 mg, 2 µmol, 1 equiv) were dissolved in water (2 mL) in a 4-mL vial equipped with a stir bar. The reaction was then irradiated with two 660 nm Kessil lamps (35 W) at room temperature. After 30 minutes, the reactions were filtered through glass filter paper to obtain a crude mixture of **3**. The crude mixture was then charged with $CuSO_4 \cdot 5H_2O$ (0.1 equiv), sodium ascorbate (0.2 equiv), TBTA (0.05 equiv), and Biotin-(PEG)$_2$-N$_3$ **S8** (1 equiv). After 2 hours, the reaction mixture was filtered through glass filter paper to obtain crude mixture of **5**. 50 µL of crude reaction was pipetted into a 2 mL vial and diluted with 1 mL of H_2O for LC-MS analysis (Figure S18A).

<u>Direct addition of Biotin to peptide **2** via TagC-RED with biotin-attached-diazonium reagent **6**</u>: **6** (2.5 mg, 2.8 µmol, 3 equiv) and peptide **2** (1 mg, 0.95 µmol, 1 equiv) were dissolved in water (2 mL) in a 4-mL vial equipped with a stir bar. The reaction was then irradiated with two 660 nm Kessil lamps (35 W) at room temperature. After 30 minutes, the reactions were filtered through glass filter paper. 50 µL of the filtered sample was pipetted into a 2 mL vial and diluted with 1 mL of H_2O for LC-MS analysis (Figure S18B).

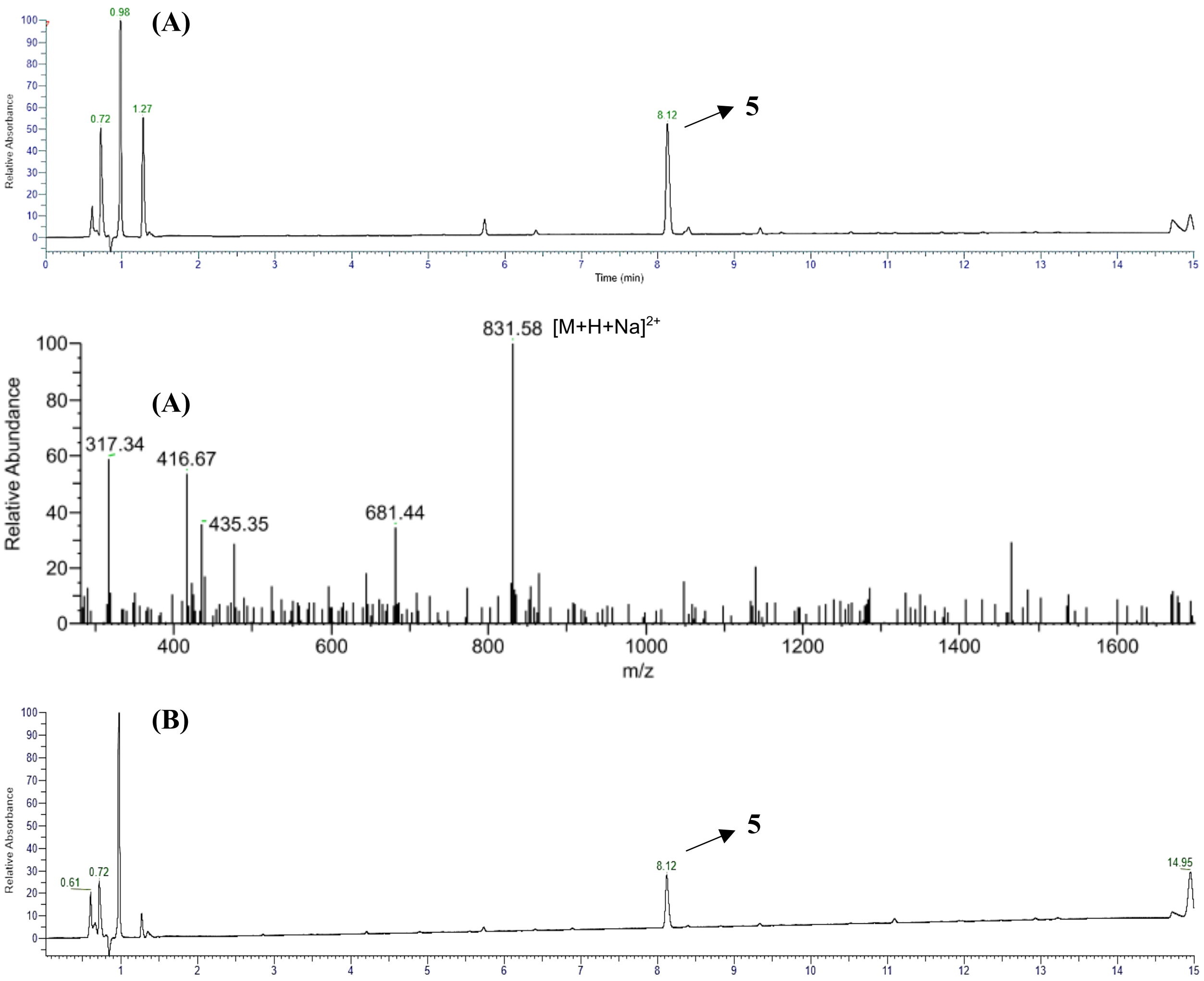

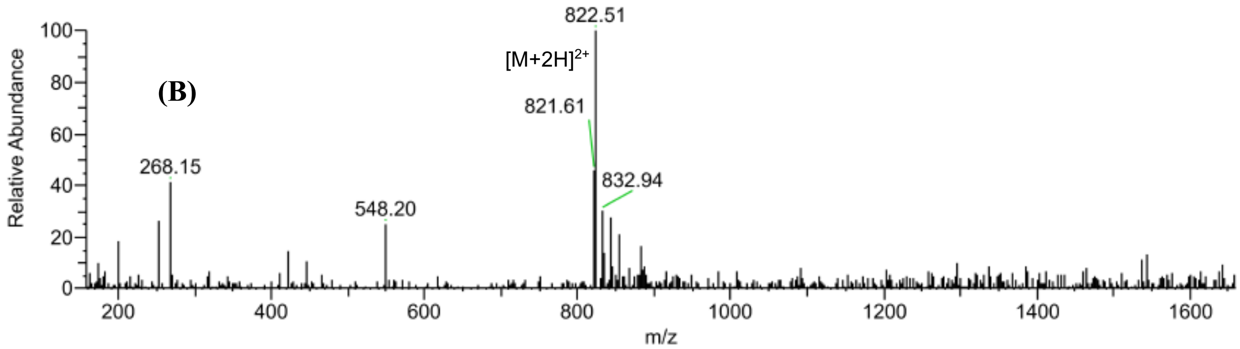

Figure S19. (A) LCMS and ESI-TOF of the reaction with modified peptide **3** "clicked" with biotin to form **5**. (B) LCMS and ESI-TOF of the TagC-RED reaction with peptide **2** and diazonium **6** to form **5**.

4.6. Characterization of 3

Purification was performed by reversed phase HPLC using Luna® C18 column (250×21.2mm, 10 µm) flow rate = 15.0 mL/min, with an isocratic eluent 40:60 v/v (0.1% v/v FA in MeCN : 0.1% FA TFA in H_2O) over 10 min, followed by 50:50 v/v (0.1% v/v TFA in MeCN : 0.1% v/v TFA in H_2O) over 2 minutes, followed by 65:35 v/v (0.1% v/v TFA in MeCN : 0.1% v/v TFA in H_2O) over 5 minutes, then 100:0 v/v (0.1% v/v TFA in MeCN : 0.1% v/v TFA in H_2O) over 5 minutes. The fractions containing the desired peptide were collected and lyophilized to afford the title compound **3** as a colorless powder (2.4 mg, 2 µmol, 98% yield).

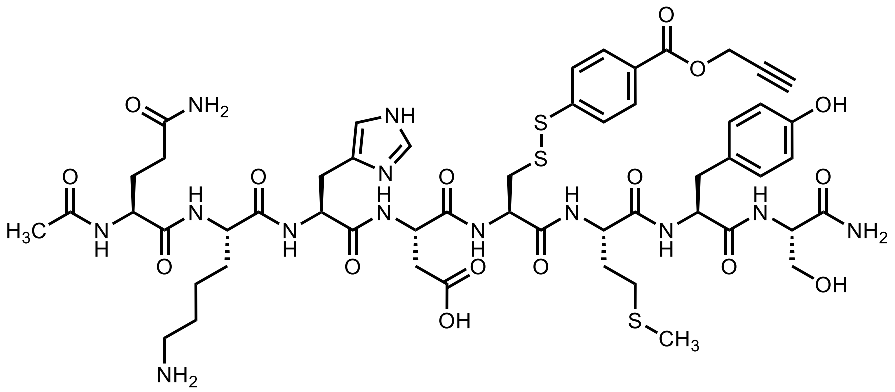

Modified Gln-Lys-His-Asp-Cys-Met-Tyr-Ser (3).

ESI⁺-HRMS: calculated for: $C_{53}H_{72}N_{13}O_{16}S_3$ ($[M+H]^+$), 1242.4377; found, 1242.4390.

^{1}H NMR (400 MHz, MeOD) δ: 8.42 (s, 4H), 8.02 – 7.97 (m, 2H), 7.81 (s, 1H), 7.69 – 7.64 (m, 2H), 7.08 (d, *J* = 8.5 Hz, 2H), 7.01 (s, 1H), 6.73 – 6.67 (m, 2H), 4.37 – 4.25 (m, 4H), 3.84 – 3.72 (m, 2H), 3.23 – 3.05 (m, 5H), 2.98 (t, *J* = 2.5 Hz, 1H), 2.90 (dt, *J* = 13.8, 8.4 Hz, 3H), 2.79 (dd, *J* = 15.8, 6.3 Hz, 1H), 2.67 (dd, *J* = 15.8, 5.3 Hz, 1H), 2.45 – 2.34 (m, 2H), 2.31 (t, *J* = 7.4 Hz, 2H), 2.01 (s, 3H), 1.99 (s, 3H), 1.92 (q, *J* = 9.3, 8.0 Hz, 3H), 1.75 – 1.60 (m, 3H), 1.47 (s, 2H).

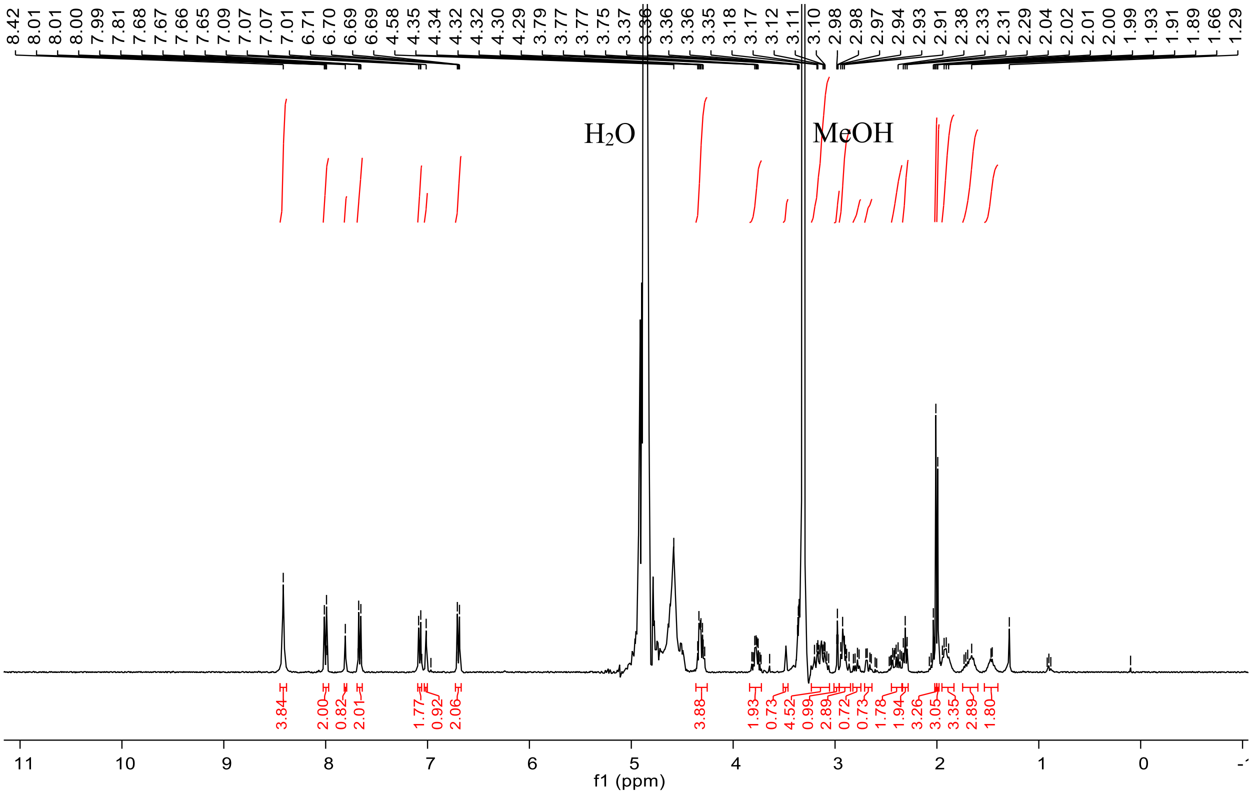

Figure S20. ^{1}H NMR (400 MHz, MeOD) spectrum of **3**.

5. Reaction and Stability of 1 with Amino Acids Containing Nucleophilic Side Chains

General Procedure 4 for selectivity studies with amino acids (GP4):

To a 4-mL clear glass vial, was added 0.5 mL of a 4 mM stock solution of the corresponding amino acid (1 equiv), **1** (3.0 mg, 6 µmol, 3 equiv), 1.5 mL of water, and a stir bar. The reaction mixture was irradiated with two 660 nm Kessil lamps (35 W) while stirring at room temperature for 30 minutes. The samples were then filtered through glass filter paper and were subjected to analysis via LC/MS.

LC-MS method for analysis of amino acid conjugation:

LC-MS was performed on a Thermo Scientific TSQ Fortis Plus Triple Quadrupole mass spectrometer coupled to a Zorbax RRHD Eclipse Plus C18 column (95 Å, 1.8 µm, 3.0 x 100 mm). Solvents A, water with 0.1% formic acid and B, acetonitrile with 0.1% formic acid were used as the mobile phase with a flow rate of 0.60 mL/min. The gradient was programmed as follows: 95% A for 13 minutes, then 60% A for 2 minutes. *m/z* values of positively and negatively charged ions were measured in the mass analyzer, which was scanned between *m/z* 100–2000 for the generation of mass spectra. The major peak(s) were selected for integration and analyzed using Freestyle software (v. 1.8 SP2 from Thermo Scientific).

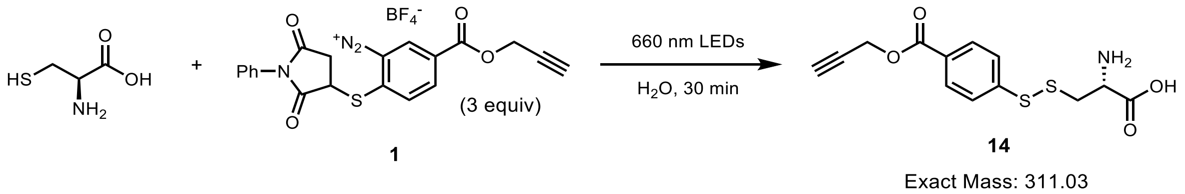

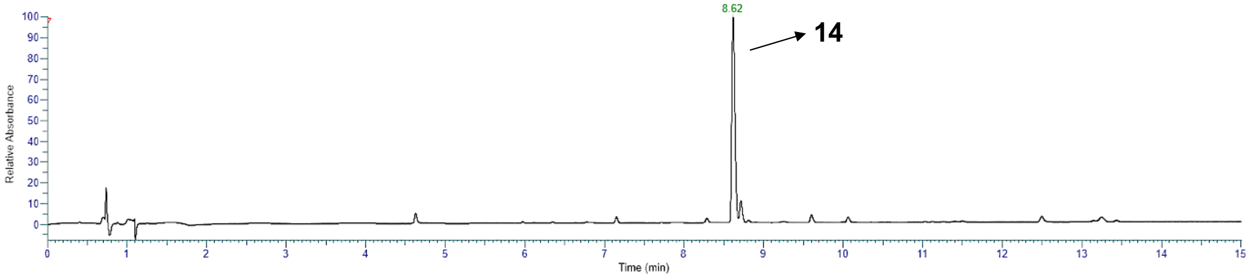

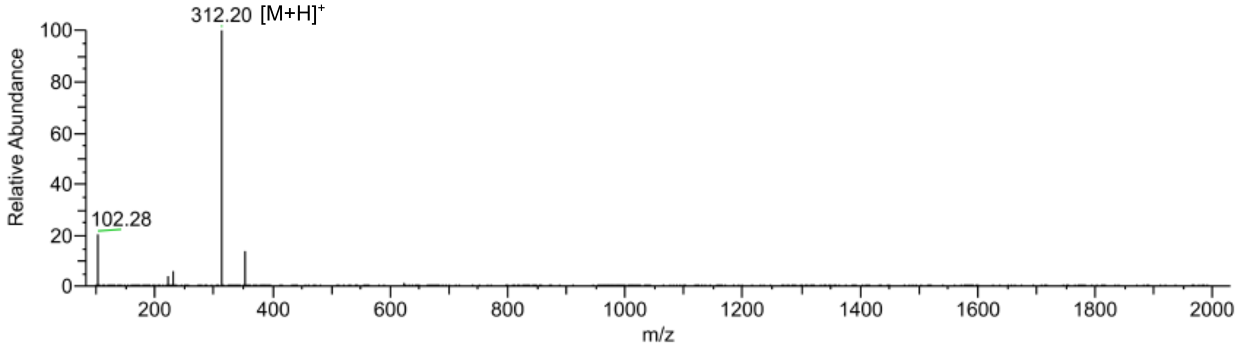

Figure S21. LCMS and ESI-TOF for the reaction between *L*-cysteine and **1**.

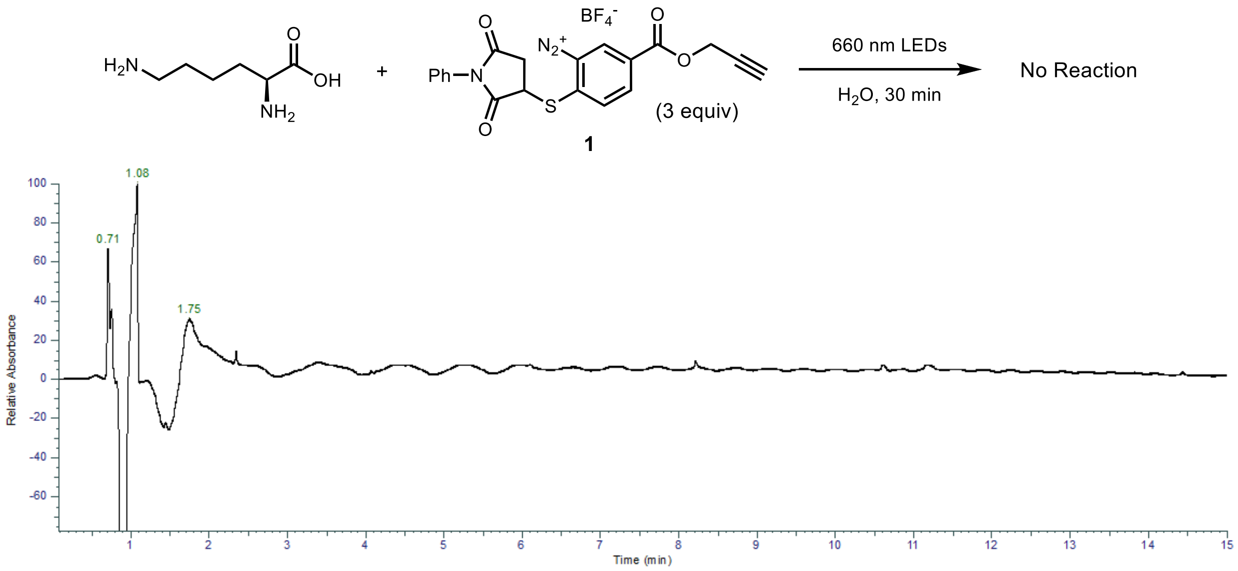

Figure S22. LCMS for the reaction between *L*-lysine and **1**.

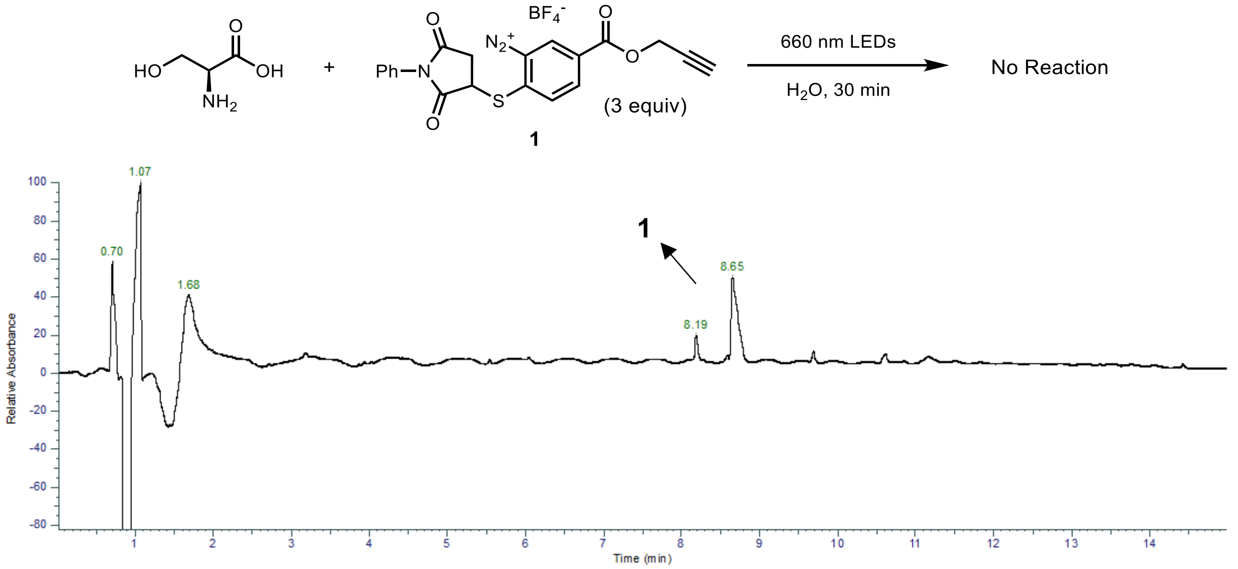

Figure S23. LCMS for the reaction between *L*-serine and **1**.

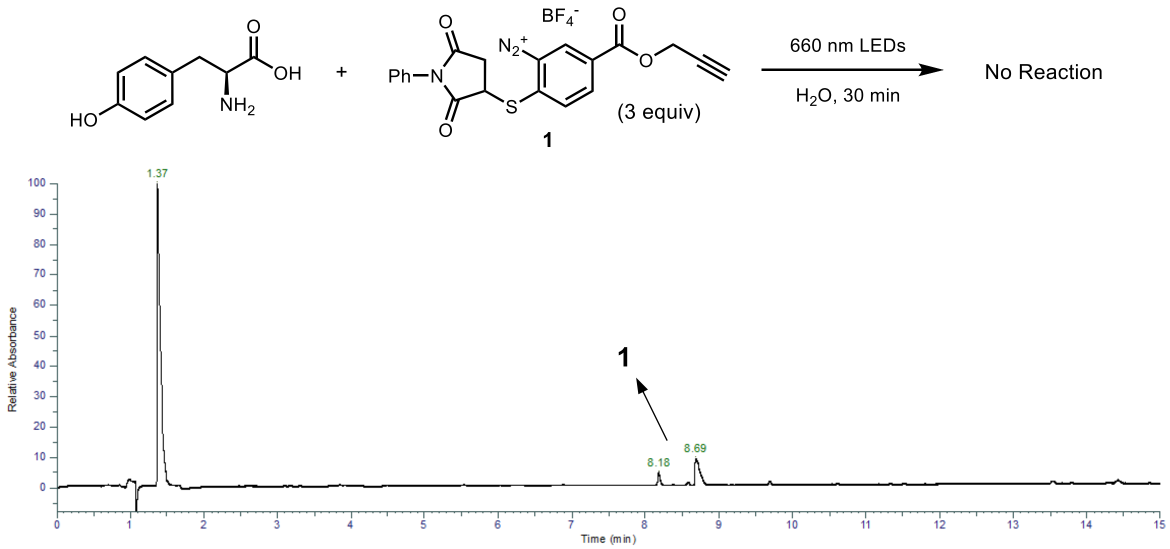

Figure S24. LCMS for the reaction between *L*-tyrosine and **1**.

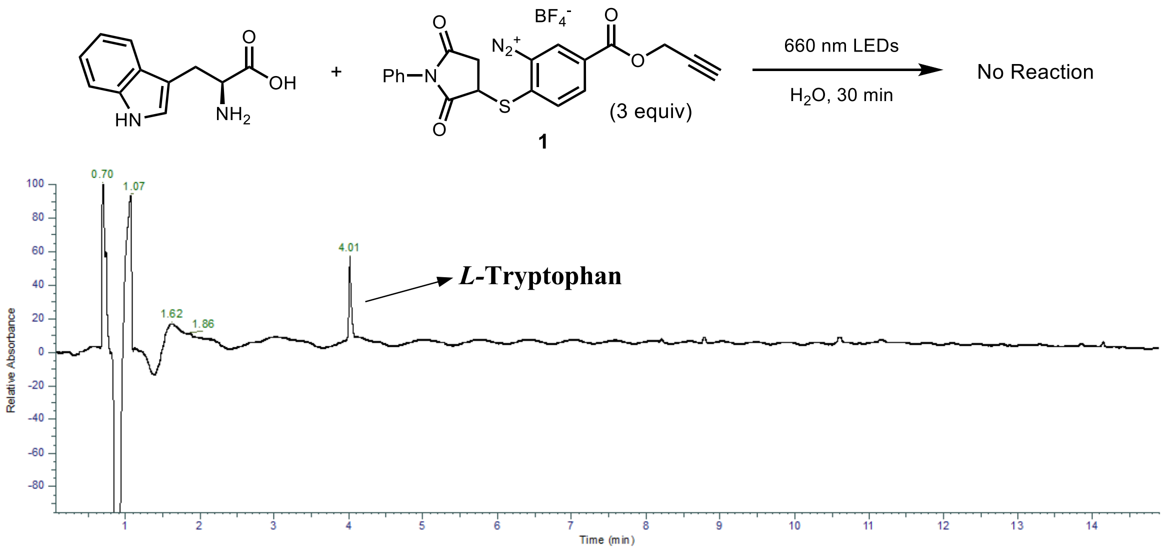

Figure S25. LCMS for the reaction between *L*-tryptophan and **1**.

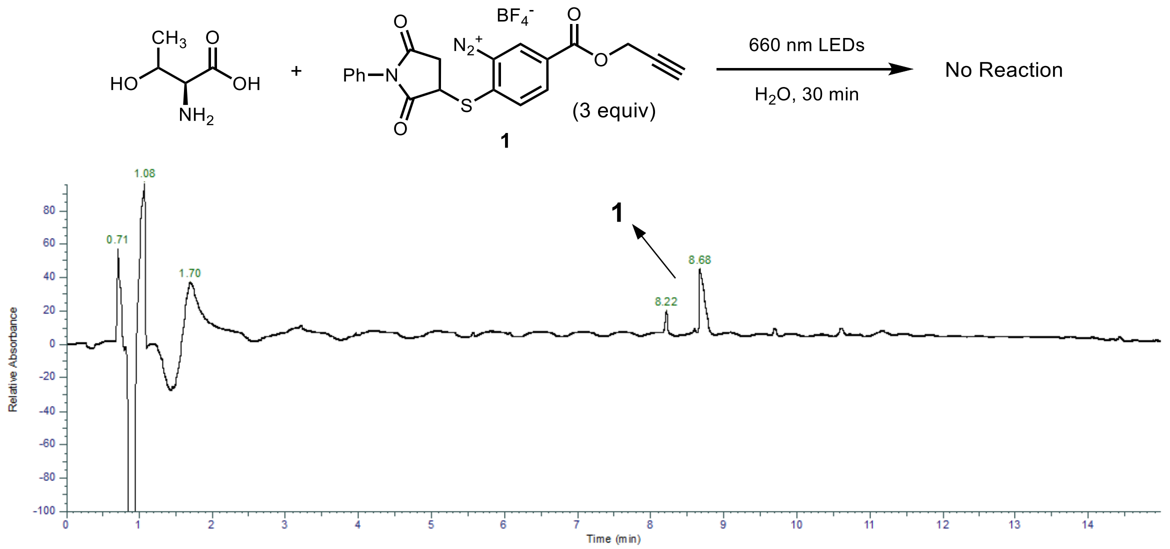

Figure S26. LCMS for the reaction between *L*-threonine and **1**.

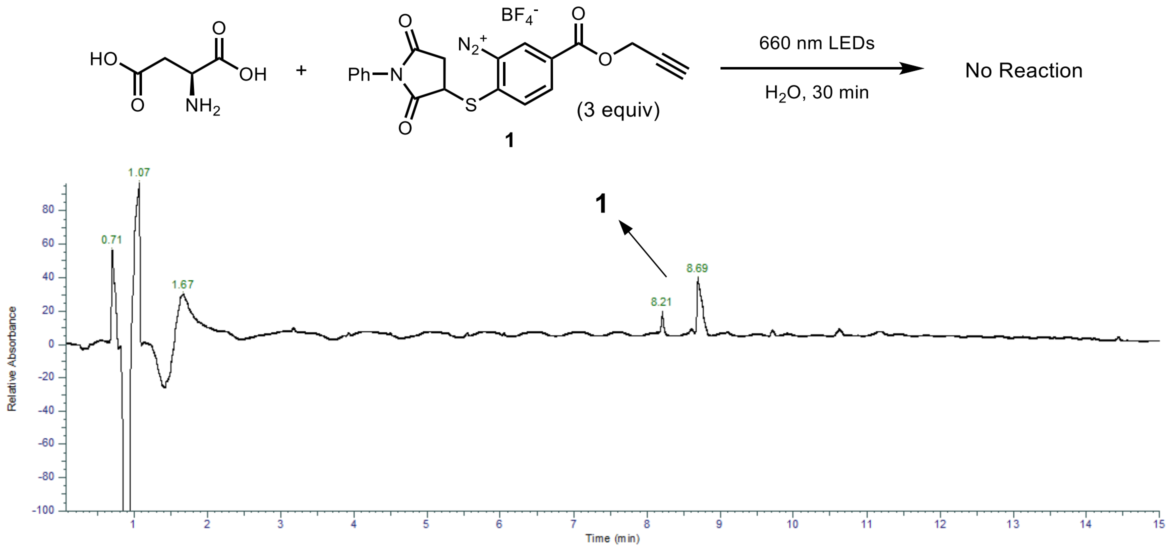

Figure S27. LCMS for the reaction between *L*-aspartic acid and **1**.

5.1. Reaction with Oxytocin

1 (3.0 mg, 6 µmol, 3 equiv) and oxytocin (2 mg, 2 µmol, 1 equiv) were dissolved in water (2 mL) in a 4-mL vial equipped with a stir bar. The reaction was then irradiated with two 660 nm Kessil lamps (35 W) while stirring at room temperature. After 30 minutes, the reactions were filtered through glass filter paper and were subjected to analysis via LC-MS.

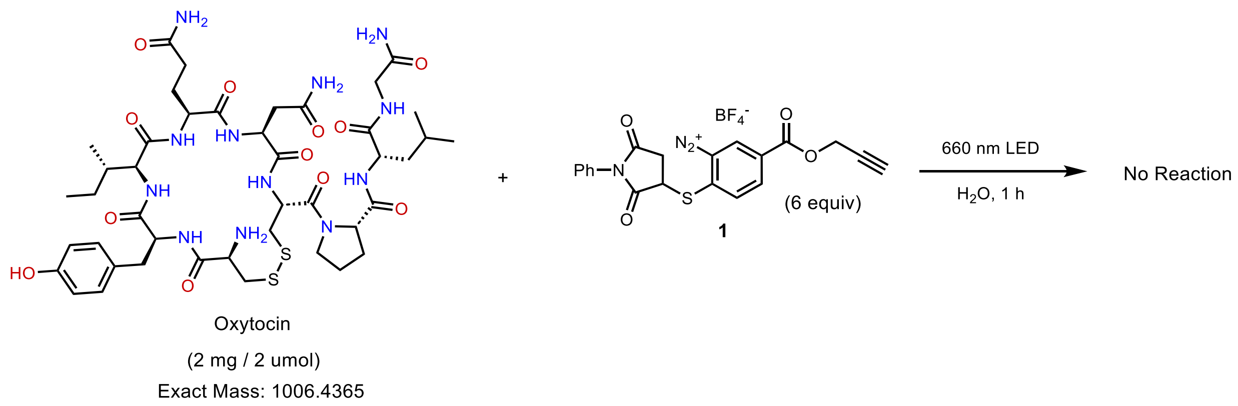

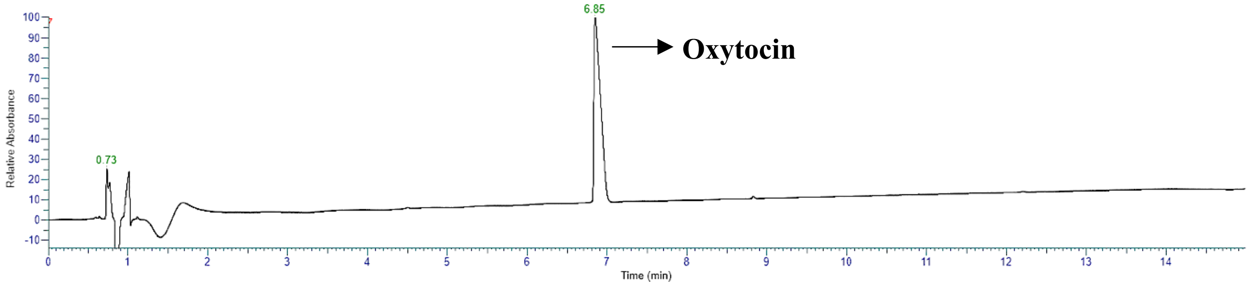

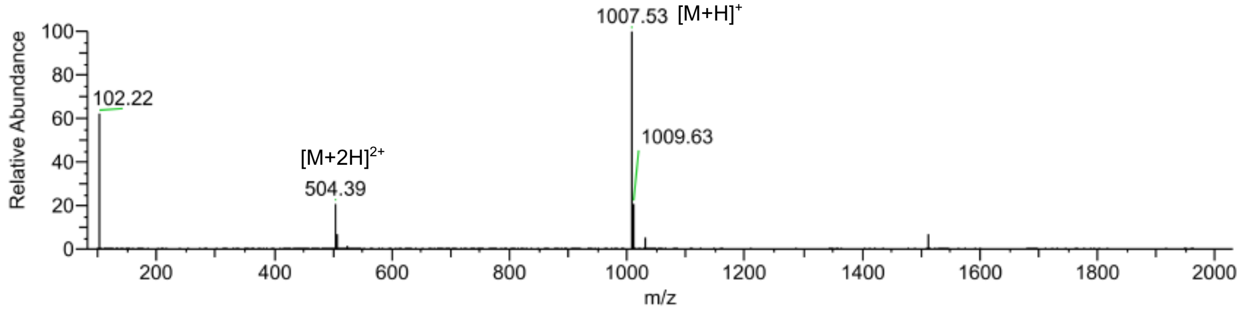

Figure S28. LCMS and ESI-TOF for reaction between oxytocin and **1**.

6. Proteins Used in This Study

6.1. Ub-K63C

The overexpression and purification protocol was carried out as previously described[7] with some modifications. His-MBP-Ub K63C was overexpressed in BL21 (DE3) competent cells. Overnight cultures (5 mL of LB with ampicillin (100 µg/mL)) were used to inoculate 500 mL of LB supplemented with ampicillin (100 µg/mL) and grown to log-phase (OD_{600} = 0.5) with shaking (37 ºC, 200 RPM). Cultures continued to grow at a lower temperature of 18 °C for 1 hr. Cultures were then induced with 1 mM isopropyl β-D-1-thiogalactopyranoside (IPTG) for 18-20 hrs (18 °C, 200 RPM). Cells were harvested by centrifugation and then stored at -80 °C.

For purification, the His-MBP-Ub K63C cell pellet was resuspended in 15 mL of amylose buffer (50 mM Sodium Phosphate, pH 7.5, 200 mM NaCl) supplemented with lysozyme (100 µg/mL), DNaseI (6.6 µg/mL) and SIGMAFAST EDTA-free protease inhibitor cocktail (Sigma-Aldrich, 1 tablet per 100 mL of buffer). Cells were rocked for 30 minutes at 4 °C before lysing using a cell disruptor (Emulsiflex-C5, 10K psi, 10 min). Lysate was then centrifuged at 20,000 x g for 1 hr. Supernatant was incubated with a washed, equilibrated amylose resin column for 0.5 hrs. The protein was washed with amylose buffer, then eluted with amylose buffer containing 20 mM maltose. The elution was incubated with TEV protease (1:20 molar ratio) overnight at 4 °C while rocking. Following cleavage, samples were incubated with a washed, equilibrated Ni-NTA resin column for 1 hr while rocking at 4 °C. The flow through was then collected and the resin was washed with amylose buffer. The flowthrough and wash samples were concentrated then further purified via gel filtration on a Superdex 200 Increase 10/300 GL column. Ubiquitin K63C was collected at the 18-mL retention volume peak, concentrated, and flash frozen to be stored at -80 °C.

Amino acid-sequence Ub-K63C:
SAQIFVKTLTGKTITLEVEPSDTIENVKAKIQDKEGIPPDQQRLIFAGKQLEDGRTLSDYNI
QCESTLHLVLRLRGG

Isotopically Averaged Molecular Weight = 8566.77 Da

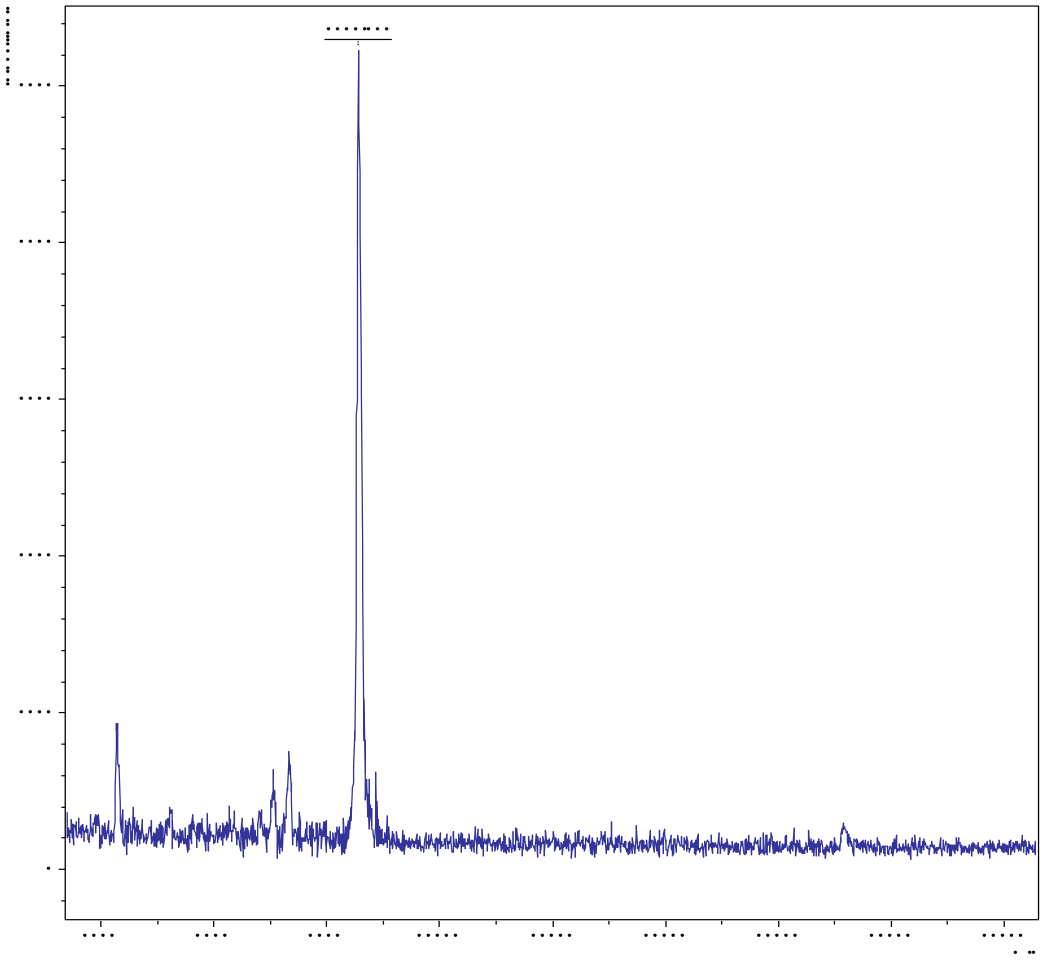

Figure S29. MALDI-TOF of Ub-K63C.

6.2. Trastuzumab-LALAPG-S239C

The sequences were designed using published sequences of trastuzumab (PDB: 1N8Z).

The protein was produced in CHO cells using synthetic genes encoding the designed amino sequences, and purified to apparent homogeneity as judged with SDS-PAGE and size-exclusion chromatography by Biointron, Inc.

Amino acid-sequence of Traustuzumab-LALAPG-S239C:

>Heavy chain:
EVQLVESGGGLVQPGGSLRLSCAASGFNIKDTYIHWVRQAPGKGLEWVARIYPTNGYT
RYADSVKGRFTISADTSKNTAYLQMNSLRAEDTAVYYCSRWGGDGFYAMDYWGQGT
LVTVSSASTKGPSVFPLAPSSKSTSGGTAALGCLVKDYFPEPVTVSWNSGALTSGVHTFP
AVLQSSGLYSLSSVVTVPSSSLGTQTYICNVNHKPSNTKVDKKVEPKSCDKTHTCPPCPA
PEAAGGPCVFLFPPKPKDTLMISRTPEVTCVVVDVSHEDPEVKFNWYVDGVEVHNAKT
KPREEQYNSTYRVVSVLTVLHQDWLNGKEYKCKVSNKALGAPIEKTISKAKGQPREPQ
VYTLPPSREEMTKNQVSLTCLVKGFYPSDIAVEWESNGQPENNYKTTPPVLDSDGSFFL
YSKLTVDKSRWQQGNVFSCSVMHEALHNHYTQKSLSLSPG

>Light chain:
DIQMTQSPSSLSASVGDRVTITCRASQDVNTAVAWYQQKPGKAPKLLIYSASFLYSGVP
SRFSGSRSGTDFTLTISSLQPEDFATYYCQQHYTTPPTFGQGTKVEIKRTVAAPSVFIFPPS
DEQLKSGTASVVCLLNNFYPREAKVQWKVDNALQSGNSQESVTEQDSKDSTYSLSSTL
TLSKADYEKHKVYACEVTHQGLSSPVTKSFNRGEC

Isotopically Averaged Molecular Weight = 146834 KDa

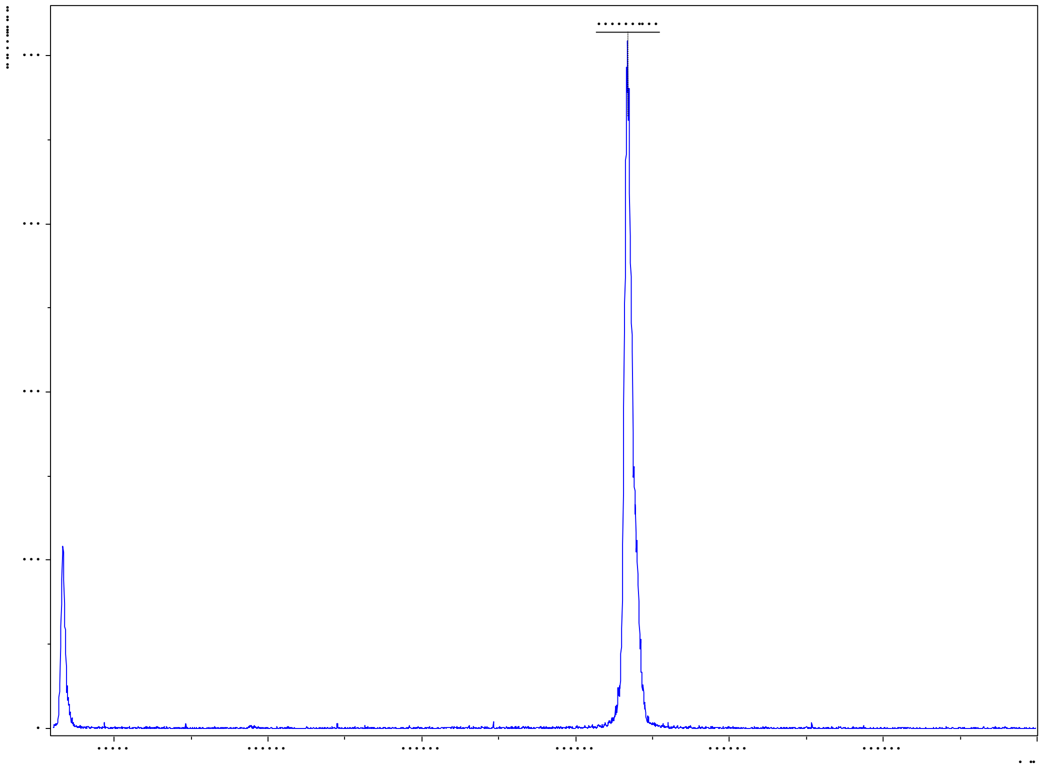

Figure S30. MALDI-TOF of Trastuzumab-LALAPG-S239C.

7. Procedures for Protein Modifications

7.1. Reaction and Characterization of Ub-K63C-Biotin Conjugate

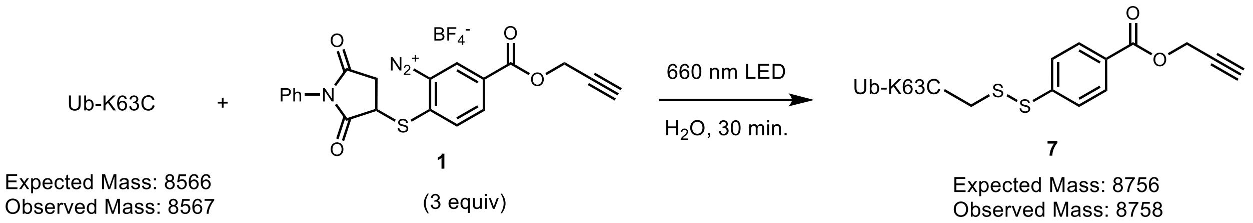

To a 0.2 mL PCR tube containing 22 µL of stock solution of Ub-K63C (150 µM) was added a 5 µL of a 2 mM stock of **1** in H_2O. The resulting mixture was then irradiated with two 660 nm Kessil lamps (35 W). After 30 minutes, a 3 µL aliquot was analyzed by MALDI-TOF, and complete conversion to the desired product was observed (Figure S30).

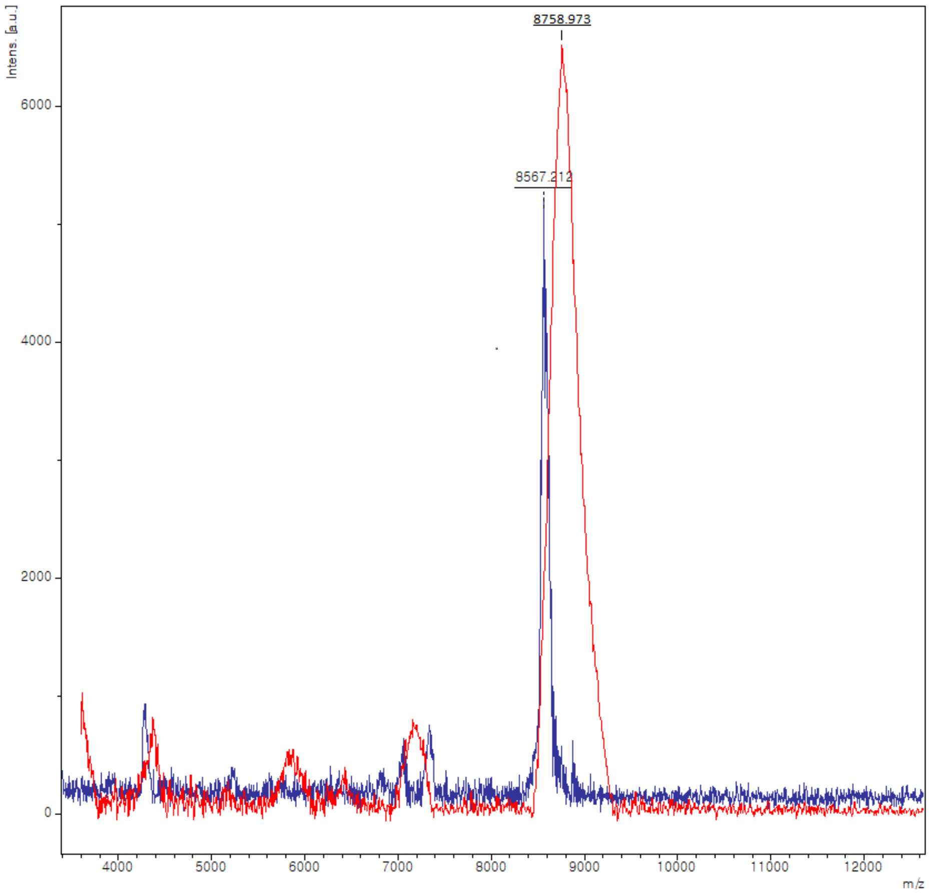

Figure S31. MALDI-TOF of the reaction between Ub-K63C and **1** after 660 nm light irradiation for 30 minutes. Blue trace represents Ub-K63C before red light irradiation. Red trace represents **7**.

7.2. Reaction and Characterization of BSA Conjugates

7.2.1. General Procedure A for Irradiation Reactions

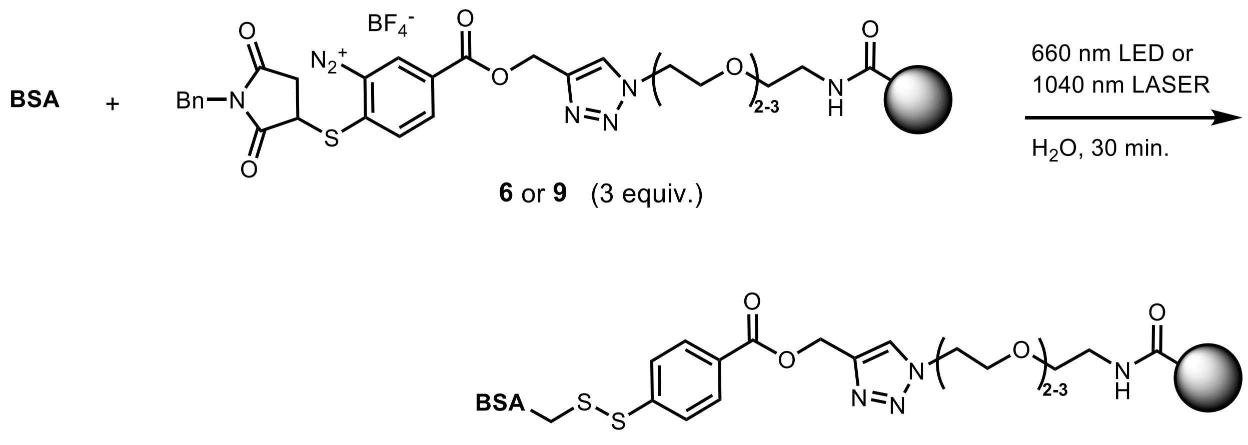

To 4-mL clear glass vials containing BSA (3 mg/0.05 µmol) was added 0.5 mL of a 0.3 mM stock solution of either **6** or **9** in water (1.5 µmol, 3 equiv). The mixture was further diluted with 1.5 mL water (2 mL total volume). The mixture was sonicated for 1 minute and subsequently irradiated with either Kessil 660 nm red LED lights (35W), irradiated with 1040 nm laser, kept under ambient light, or kept in the dark wrapped in aluminum foil for 20-30 minutes at room temperature. 3 µL aliquots were analyzed by MALDI-TOF using a super-DHB matrix to determine the success of the reaction.

7.2.2. General Western Blot Procedure B with BSA-Biotin Conjugates

Samples were prepared according to General Procedure A using diazonium **6**. Samples were either irradiated with Kessil 660 nm red LED lights (35W), irradiated with 1040 nm laser, kept under ambient light, or kept in the dark wrapped in aluminum foil for 20-30 minutes. From each sample, 20 µL (15 µg of protein) was diluted with 2X non-reducing Laemmli SDS buffer and loaded in a pre-made 4% stacking 9% resolving SDS-PAGE polyacrylamide gels in Criterion gel cassettes. Gel electrophoresis was performed using Mini-PROTEAN Tetra Vertical Electrophoresis Cell tanks and a BioRad PowerPac Basic Power Supply. Gels were run at 80 volts for 20 minutes and then 180 volts for 60-70 minutes using Tris-glycine-SDS running buffer (25 mM Tris, 192 mM glycine, 0.1% SDS). The proteins were transferred from the gel to a PVDF membrane (pre-activated with methanol). For the transfer, the membrane and gel were placed between two extra thick western blotting filter papers, pre-wetted with transfer buffer (25 mM Tris, 192 mM glycine), and then placed in a chamber in a Bio-Rad Trans-Blot Turbo System, set to a standard condition of 25V, 1.0A for 30 minutes. The membrane was removed from the chamber and was then blocked with a 5% w/v milk TBST blocking buffer (5% w/v dry milk powder, 1X TBS, 0.1% Tween-20, pH 7.8) on a rotating platform for 60 minutes. The blocking buffer was removed, and the membrane was immersed in a 1:1000 α-biotin HRP antibody (Cell Signaling 7075S) solution in blocking buffer and allowed to incubate on a rotating platform at room temperature for 1 hour. The antibody solution was decanted, and the membrane was washed with TBST buffer on a rotating platform at room temperature for 20 minutes. The solution was decanted, and the TBST wash step was repeated two additional times. The final wash solution was decanted, and the membrane was layered with 1 mL Bio-Rad Clarity™ Western ECL Substrate Peroxide Solution and 1 mL Bio-Rad Clarity™ Western ECL Substrate Luminol/Enhancer Solution and incubated for 1 minute.

The membrane was immediately imaged using a UV tray in a Bio-Rad ChemiDoc Imaging System. For total protein, gels were incubated in Coomassie solution (5:4:1 methanol:ddH_2O:acetic acid with 0.05% Coomassie) for 1 hour, decanted, and then incubated in a destain solution (5:4:1 methanol:ddH_2O:acetic acid) overnight. The gels were imaged using a white tray in the Bio-Rad ChemiDoc Imaging System.

7.2.3. SDS-PAGE Fluorescence Procedure with BSA-Fluorescein Conjugate

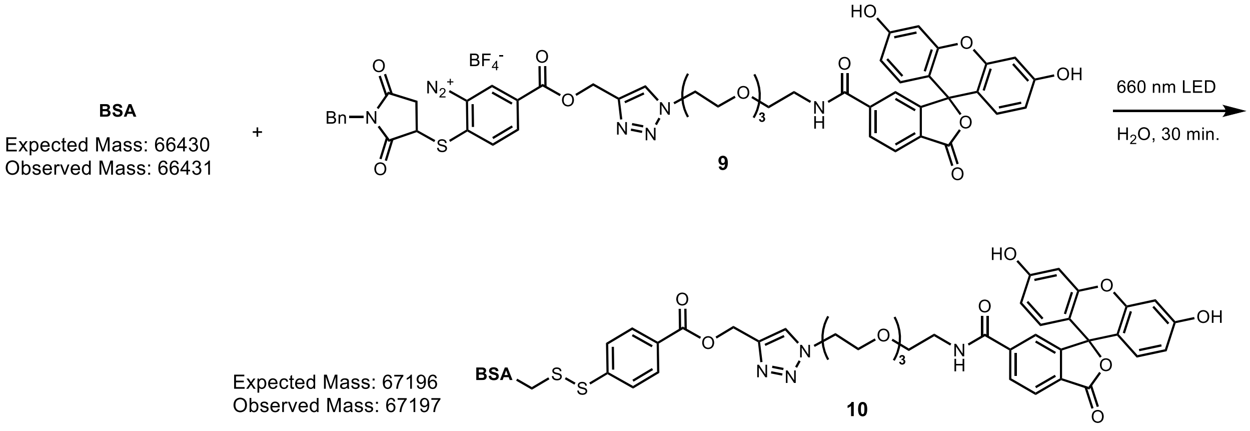

Samples were prepared according to General Procedure A using diazonium **9**. Samples were either irradiated with Kessil 660 nm red LED lights (35W) or kept in the dark wrapped in aluminum foil for 20 minutes. 3 µL aliquots were analyzed by MALDI-TOF using a super-DHB matrix to determine success of the reaction (Figure S31). From each sample, 20 µL (15 µg of protein) was diluted with 2X non-reducing Laemmli SDS buffer and loaded in a SDS-PAGE polyacrylamide gel. Gel electrophoresis was performed using Mini-PROTEAN Tetra Vertical Electrophoresis Cell tanks, BioRad PowerPac Basic Power Supply, and pre-made 4% stacking 9% resolving SDS-PAGE polyacrylamide gels in Criterion gel cassettes. Gels were run at 80 volts for 20 minutes and then 180 volts for 1 hour using Tris-Glycine-SDS running buffer (25 mM Tris, 200 mM glycine, 35 mM SDS). The gel was removed from the cassette and the fluorescence was measured using Amersham Typhoon Biomolecular Imager with a FITC filter tray (Figure S32). For total protein, the gel was incubated in Coomassie solution (5:4:1 methanol:ddH_2O:acetic acid with 0.05% Coomassie) for 1 hour, decanted, and then incubated in a destain solution (5:4:1 methanol:ddH_2O:acetic acid) overnight. The gel was imaged using a white tray in the Bio-Rad ChemiDoc Imaging System.

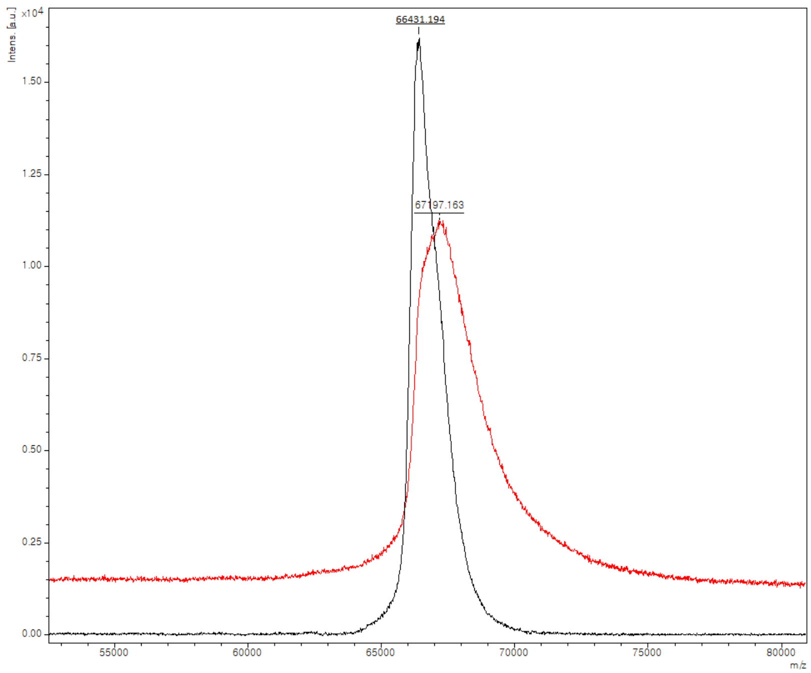

Figure S32. MALDI-TOF of the reaction between BSA and **9** after 660 nm light irradiation for 30 minutes. Black trace represents BSA before light irradiation. Red trace represents **10** from the 660 nm irradiation reaction.

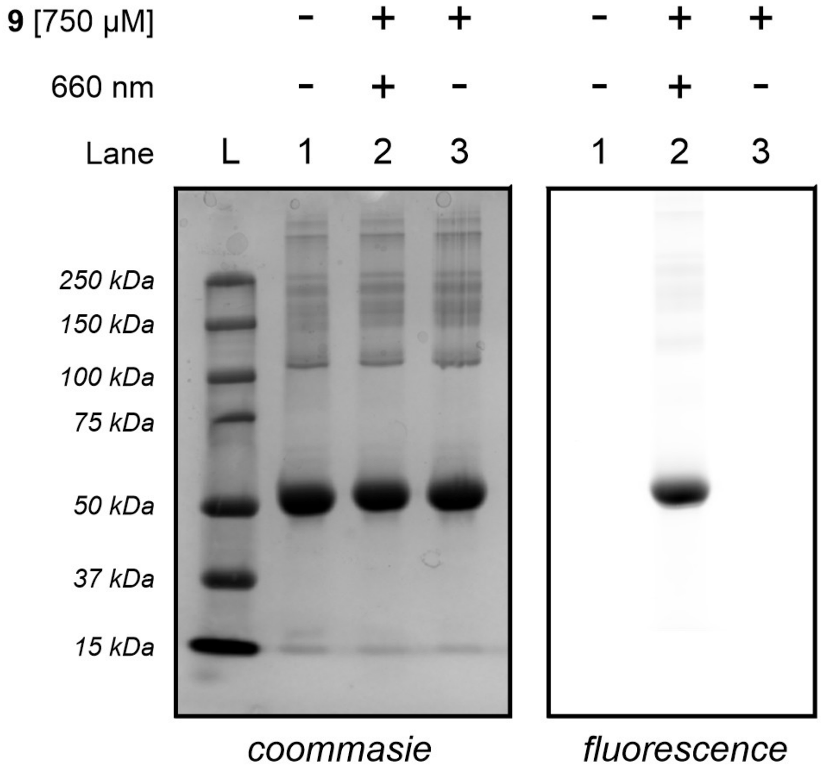

Figure S33. SDS-PAGE Gel and Fluorescence Assay of BSA under reaction conditions with **9**. Lane L: Precision Plus Protein™ All Blue Prestained Protein Standards; Lane 1: BSA alone in the absence of **9** and red light; Lane 2: BSA in the presence of **9** with 660 nm red light LED irradiation; Lane 3: BSA in the presence of diazonium **9** in the Dark.

7.2.4. BSA Reactions with 660 nm LED Irradiation

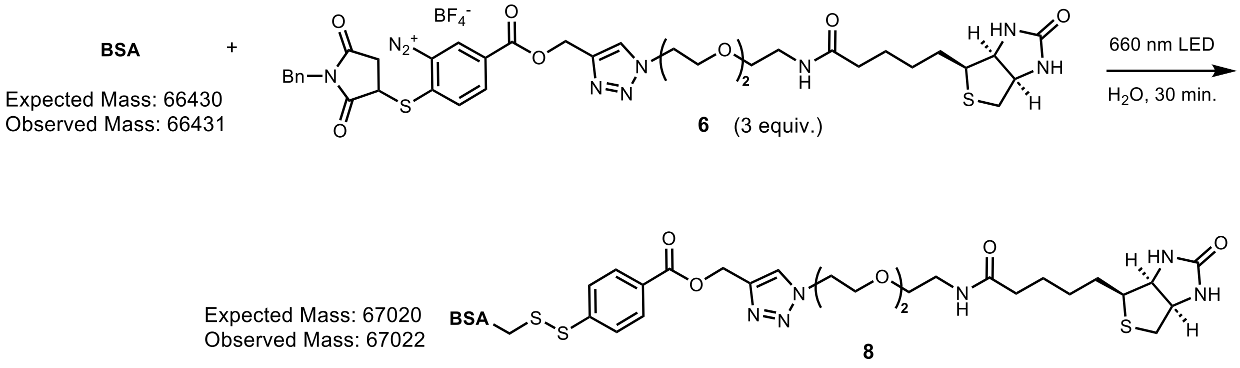

Samples were prepared according to General Procedure A using diazonium **6**. BSA samples were either irradiated with Kessil 660 nm red LED lights (35W) or kept in the dark wrapped in aluminum foil for 20 minutes. 3 μL aliquots were analyzed by MALDI-TOF using a super-DHB matrix to determine success of the reaction (Figure S33). The sample was also analyzed by western blot analysis following General Western Blot Procedure B (Figure S34). Antibody staining with anti-biotin-HRP demonstrated biotin labeling of BSA only in the presence of **6** and light irradiation (Lane 1).

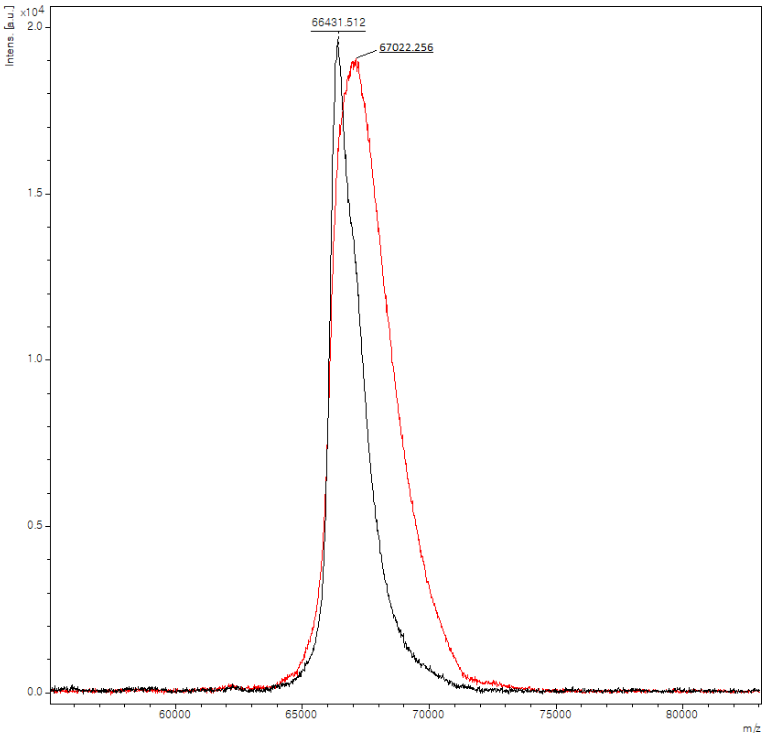

Figure S34. MALDI-TOF of the reaction between BSA and **6** after 660 nm light irradiation for 30 minutes. Black trace presents BSA before red light irradiation. Red trace represents **8** from the 660 nm irradiation reaction.

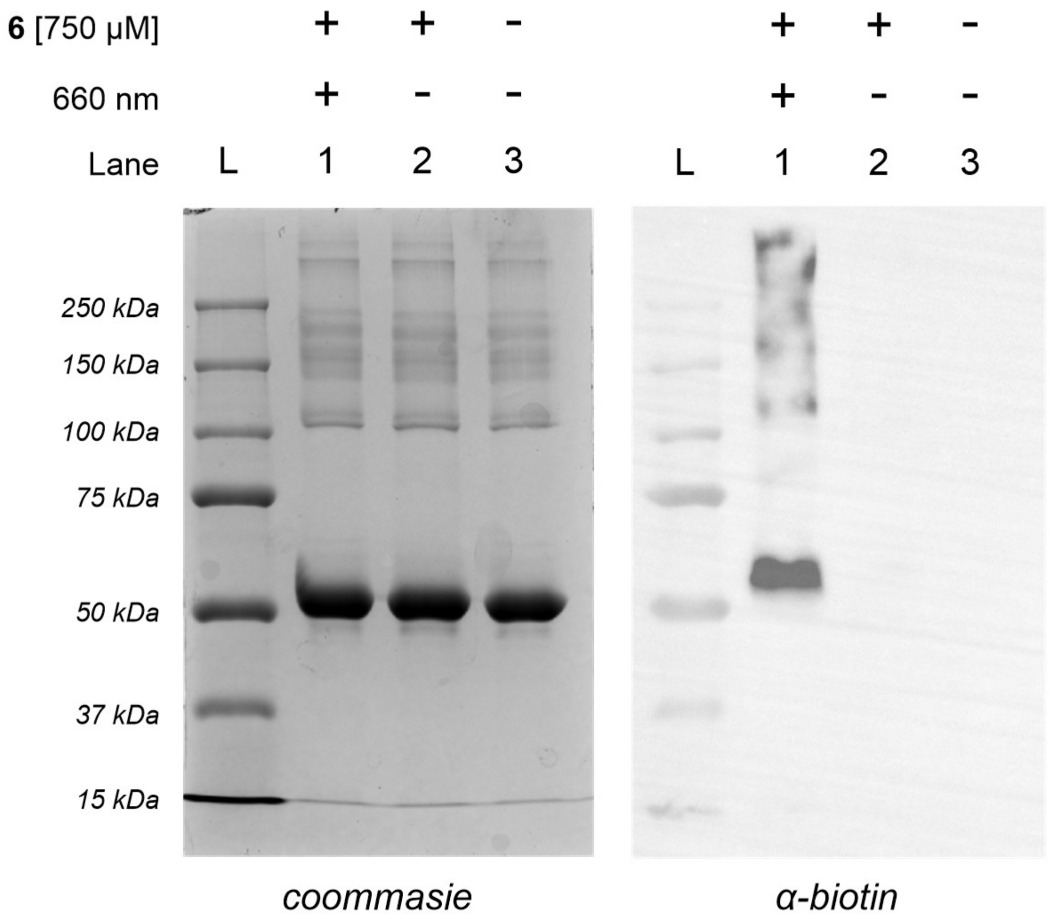

Figure S35. Western Blot Assay of Red Light-Mediated Biotinylating of BSA with **6**. Lane L: Precision Plus Protein™ All Blue Prestained Protein Standards; Lane 1: BSA in the presence of **6** with 660 nm red light LED irradiation; Lane 2: BSA in the presence of diazonium **6** in the Dark; Lane 3: BSA alone in the absence of **6** and red light.

7.2.5. BSA Reactions with 1040 nm Laser Irradiation

Samples were prepared according to General Procedure A using diazonium **6** in 2-mL glass quartz vials. The resulting mixture was irradiated with 1040 nm laser at room temperature. After 30 minutes, a 3 µL aliquot was analyzed by MALDI-TOF and complete conversion to the desired product was observed (Figure S37). Western Blot analysis was also performed using General Western Blot Procedure B (Figure S41).

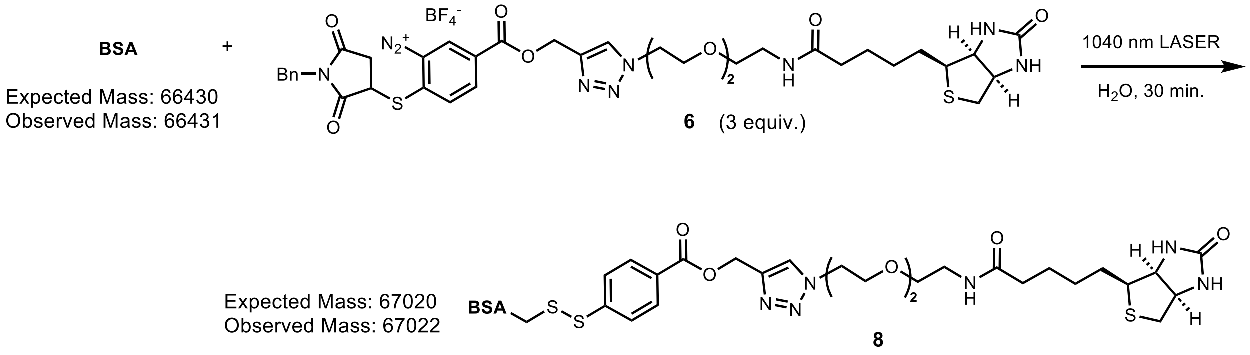

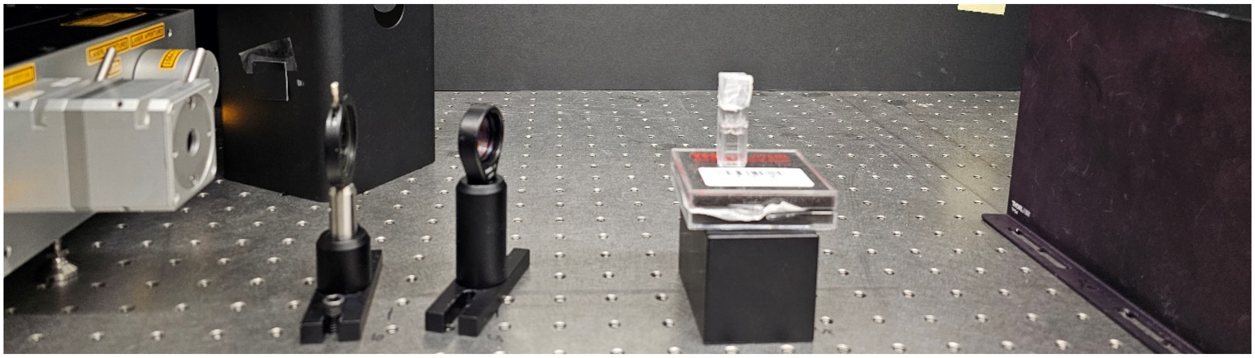

Figure S36. Photo before 1040 nm laser irradiation w/ ThorLabs Hard-Coated Longpass Filter.

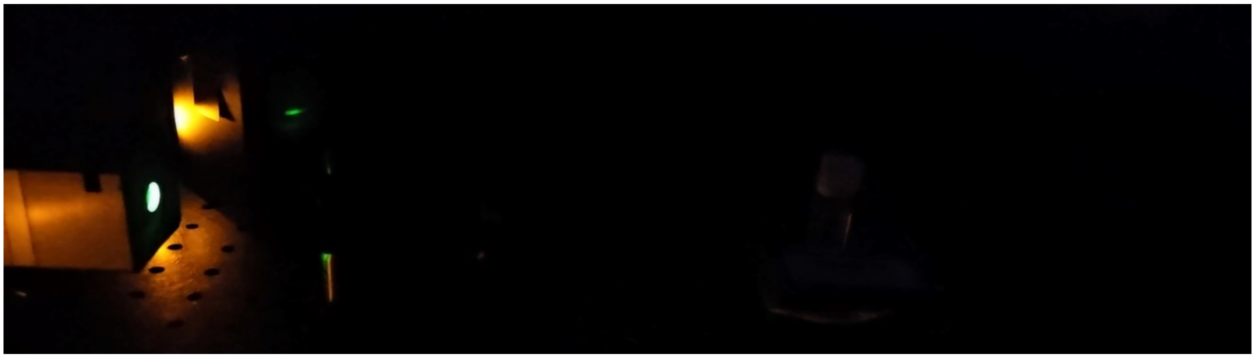

Figure S37. Photo during 1040 nm laser irradiation w/ ThorLabs Hard-Coated Longpass Filter.

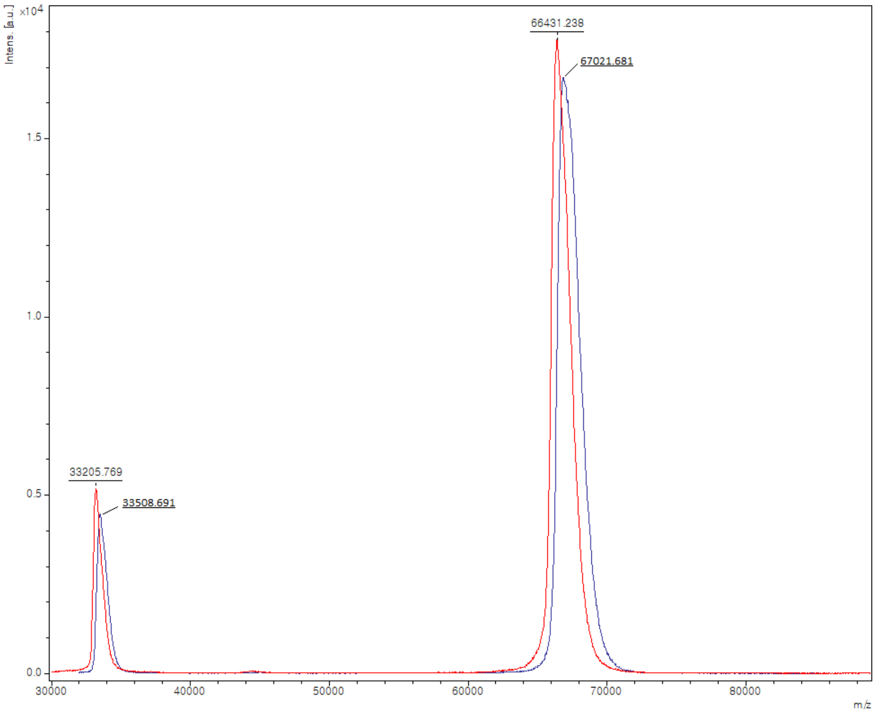

Figure S38. MALDI-TOF of the reaction between BSA and **6** after 1040 nm laser irradiation for 30 minutes. Red trace represents BSA before laser irradiation. Blue trace represents **8** from the 1040 nm irradiation reaction.

7.2.6. BSA Reactions with 1040 nm Laser Irradiation through Bacon Layer

Samples were prepared according to General Procedure A using diazonium **6** in 2-mL glass quartz vials, wrapped with a 4-mm thick bacon layer. The resulting mixture was irradiated with 1040 nm laser at room temperature. After 30 minutes, a 3 μL aliquot was analyzed by MALDI-TOF and complete conversion to the desired product was observed (Figure S40). Western Blot analysis was also performed using General Western Blot Procedure B (Figure S41).

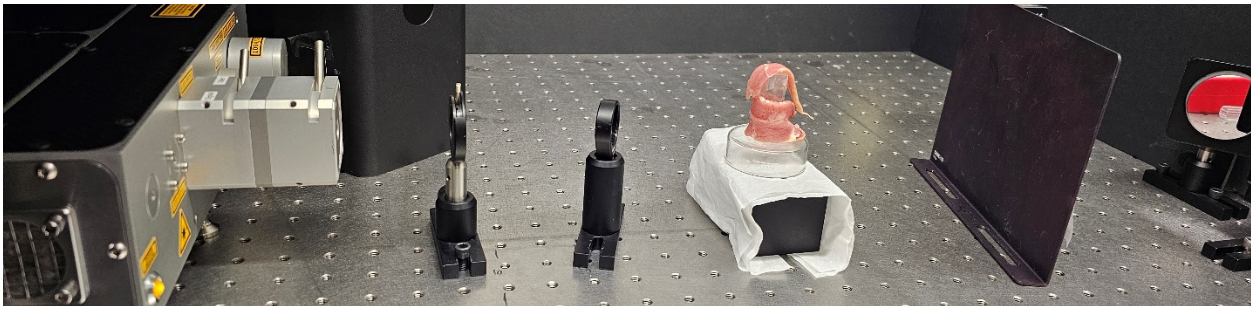

Figure S39. Photo of bacon-wrapped sample before 1040 nm laser irradiation equipped with ThorLabs Hard-Coated Longpass Filter.

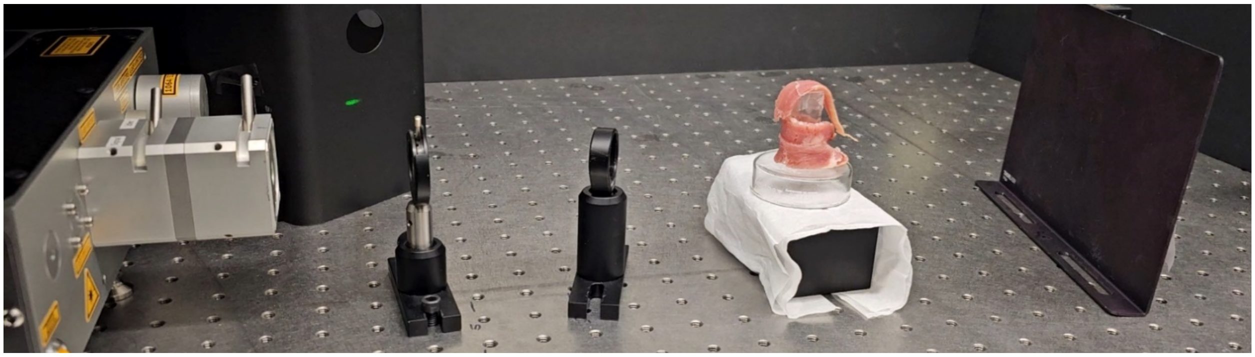

Figure S40. Photo of bacon-wrapped sample during 1040 nm laser irradiation equipped with ThorLabs Hard-Coated Longpass Filter.

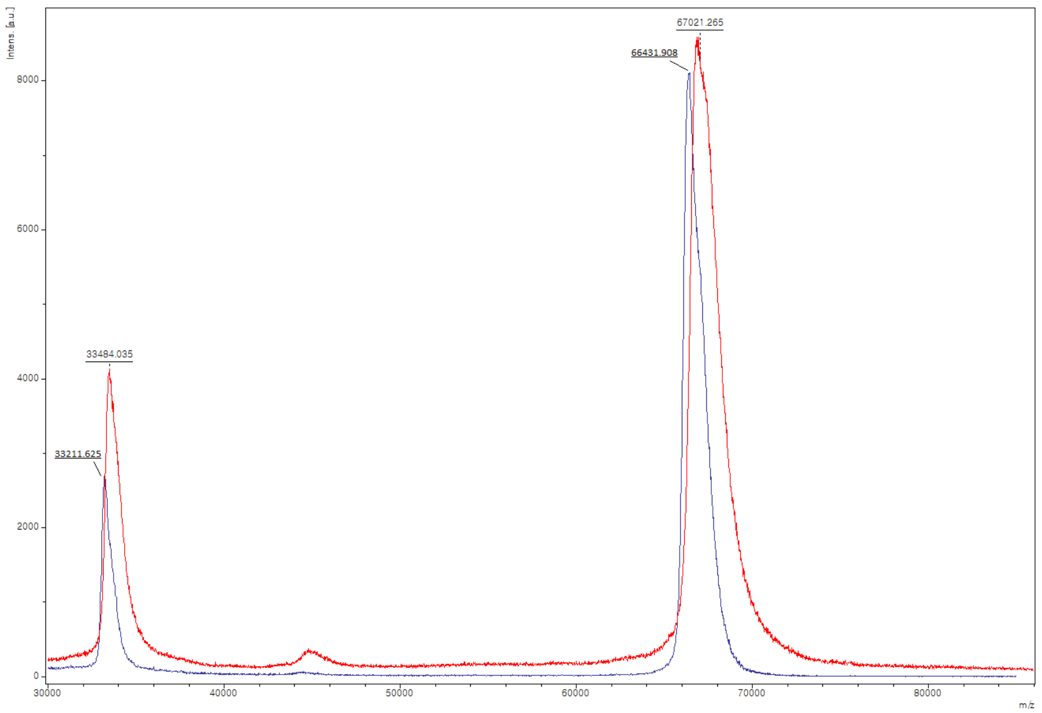

Figure S41. MALDI-TOF of the reaction between BSA and **6** after 1040 nm laser irradiation for 20 minutes. Blue trace represents BSA before laser irradiation. Red trace represents **8** from the bacon-wrapped 1040 nm irradiation reaction.

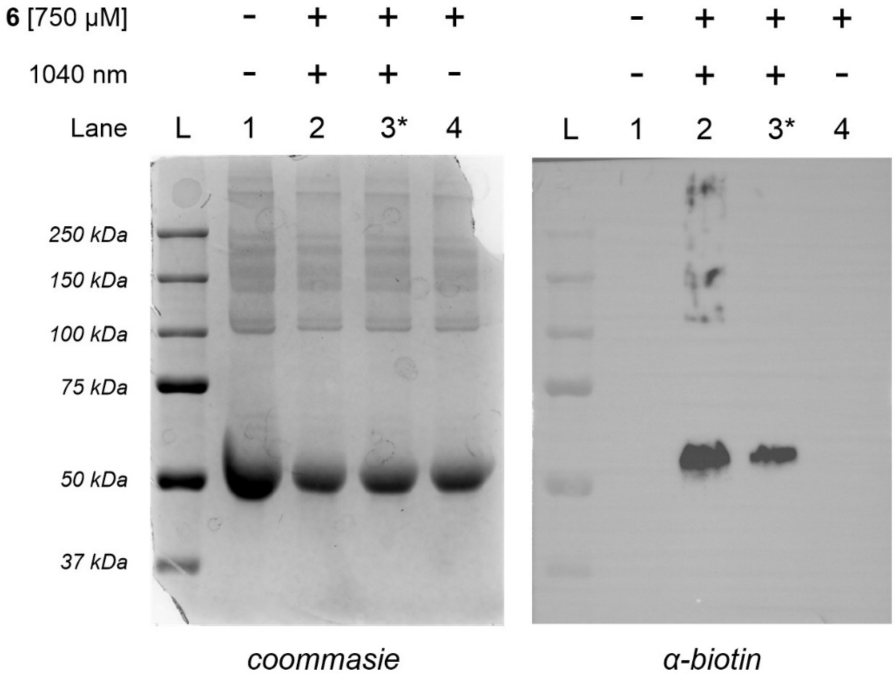

Figure S42. Western Blot Assay of 1040 nm Laser-Mediated Biotinylating of BSA with **6**. Lane L: Precision Plus Protein™ All Blue Prestained Protein Standards; Lane 1: BSA alone in the absence of **6** and no irradiation; Lane 2: BSA in the presence of **6** with 1040 nm irradiation; Lane 3: Bacon (4 mm)-wrapped sample of BSA in the presence of **6** with 1040 nm irradiation; Lane 4: BSA in the presence of **6** with no irradiation under ambient light.

7.3. Reaction and Characterization of Traustuzumab-LALAPG-S239C-Biotin Conjugate

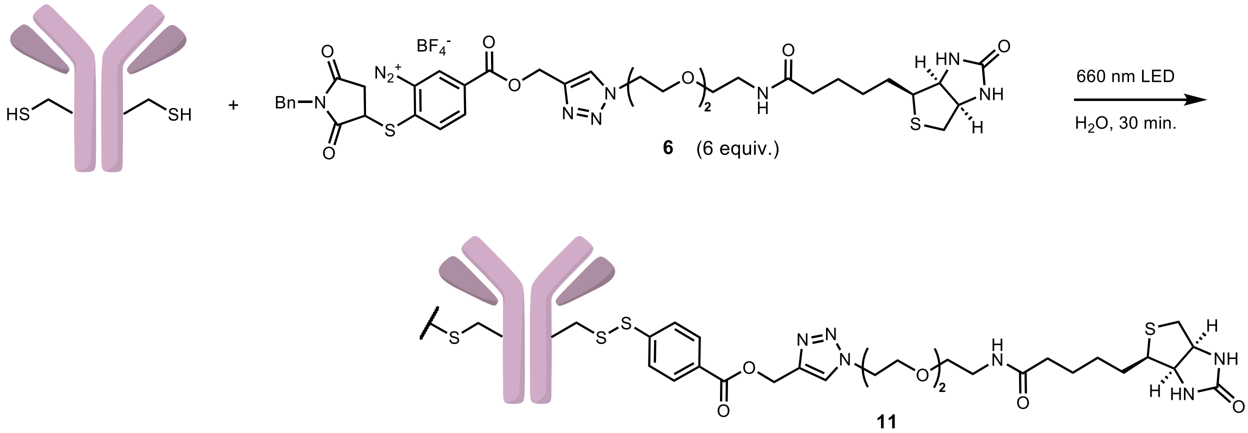

To an Eppendorf tube containing 50 µL of Traustuzumab-LALAPG-S239C (1 mg/mL in PBS pH 6.5), 21 µL of a 100 µM stock solution of **6** in H_2O (6 equiv) was added. The resulting mixture was irradiated with 660 nm LED light at room temperature. After 30 minutes, a 3 µL aliquot was analyzed by MALDI-TOF and complete conversion to the desired product was observed (Figure S42).

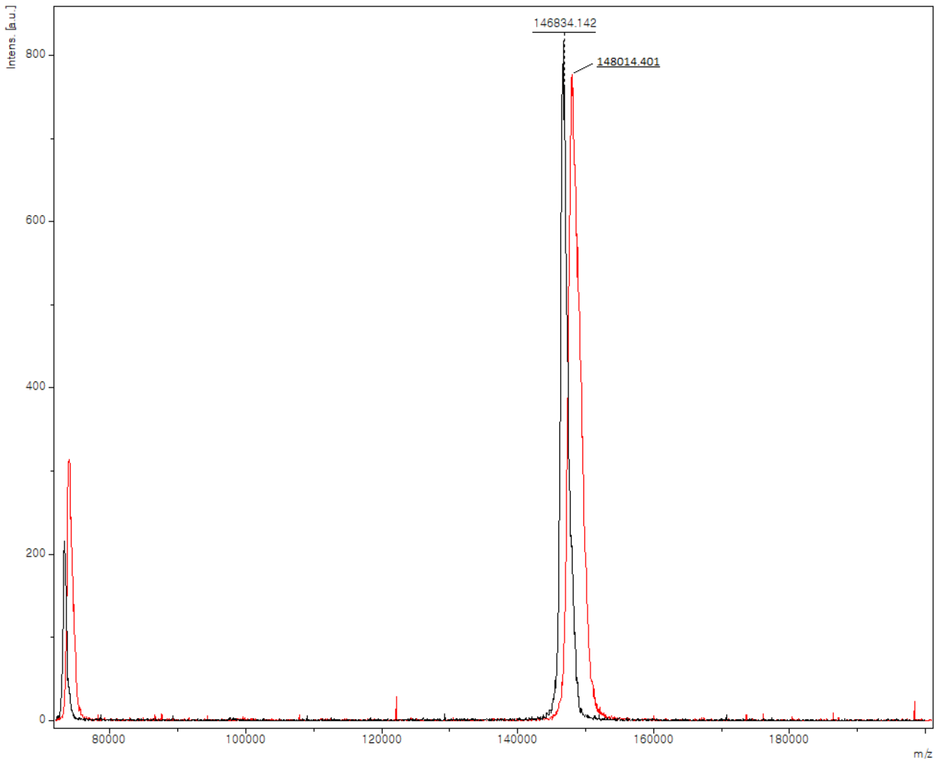

Figure S43. MALDI-TOF of the reaction between Traustuzumab-LALAPG-S239C and **6** with 660 nm light irradiation for 30 minutes. Black trace represents Traustuzumab-LALAPG-S239C before light irradiation. Red trace represents **11** from the 660 nm irradiation reaction.

8. Mechanistic Studies

8.1. UV-Vis Experiment

A 2 mM stock solution of **1** in water was prepared, and its absorbance was measured by adding 1 mL of the stock solution to 1 mL of water to make a 1 mM solution. A 1 mM stock solution of *L*-cysteine was prepared, and its absorbance was measured. To measure the UV-Vis absorption shift of **1**, a 10 mM stock solution of *L*-cysteine was prepared. To a 1 mL sample of the 2 mM stock solution of **1**, a corresponding volume of the 10 mM stock solution of *L*-cysteine was added along with water to prepare a 2 mL solution (Table S5).

Table S5. Solution Preparations for UV-Vis Study.

Sample	Diazonium Standard	1 mM Cys	2 mM Cys	3 mM Cys	4 mM Cys	5 mM Cys	Cys Standard
Diazonium **1** (mL)	1.00	1.00	1.00	1.00	1.00	1.00	0
Cys (mL)	0.00	0.20	0.40	0.60	0.80	1.00	0.20
Water (mL)	1.00	0.80	0.60	0.40	0.20	0	1.80

8.2. Quantum Yield Experiment

8.2.1. Determination of the Light Intensity at 660 nm

The photon flux of the 660 nm Kessil lamps was determined by the oxygenation of MDH.[8] A 188 μM stock solution of methylene blue (MB) was prepared by dissolving 3 mg of MB in 50 mL of $CHCl_3$ to be used as a sensitizer to generate singlet oxygen from triple oxygen. A 174 μM stock solution of MDH was prepared by dissolving 4.4 mg of MDH in 50 mL of $CHCl_3$. Both solutions were stored in the dark. To determine the photon flux, 1.0 mL of MB stock solution was added to a 4-mL vial along with 1.35 mL of MDH stock solution, to obtain 80 μM of MB and 100 μM of MDH, respectively. This solution was irradiated with two 660 nm LED lamps for 8 minutes. The absorbance of the oxygenated MDH was measured at 405 nm. A non-irradiated sample was also prepared and the absorbance at 405 nm was measured. Conversion was calculated using Eq 1.

$$mol\ MDH = \frac{V \bullet \Delta A}{l \bullet \varepsilon} \qquad \text{(Eq 1)}$$

Where V is the total volume (0.00235 L) of the solution after the addition of MDH stock solution, and ΔA is the change in absorbance at 405 nm between the irradiated and non-irradiated samples, l is the path length (1.00 cm) and ε is the molar absorptivity at 405 nm (18,090 L mol^{-1} cm^{-1}). The photon flux can be calculated using Eq 2.

$$photon\ flux = \frac{mol\ MDH}{\Phi\ (MDH) \bullet t \bullet f} \qquad \text{(Eq 2)}$$

Where Φ is the quantum yield for the photo-oxygenated MDH (0.106 for a 100 µM solution at λ= 670 nm), t is the time of irradiation (480 s) and f is the fraction of light absorbed at λ = 660 nm (0.99833, *vide infra*). The photon flux was calculated to be 3.66 x 10^{-9} einsteins s^{-1}.

Sample calculation:

$$mol\ MDH = \frac{0.00235\ L \bullet 1.428}{1.00\ cm \bullet 18{,}090\ L\ mol^{-1}cm^{-1}} = 1.86\ x\ 10^{-7}\ mol \qquad \text{(Eq 3)}$$

$$photon\ flux = \frac{1.86 \bullet 10^{-7}}{0.106 \bullet 480 \bullet 0.99833} = 3.66\ x\ 10^{-9}\ mol \qquad \text{(Eq 4)}$$

8.2.2. Determination of the Quantum Yield

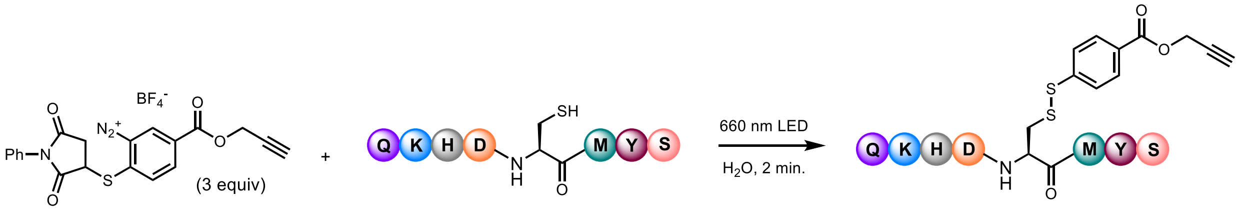

1

2

3

Exact Mass: 1051.4215

Exact Mass: 1241.4304

1 (3.0 mg, 6 µmol, 3 equiv) and peptide **2** (2 mg, 2 µmol, 1 equiv) were dissolved in water (2 mL) in a 4-mL clear glass vial equipped with a stir bar. The reaction was then irradiated with two 660 nm Kessil lamps (35 W) at room temperature. After 2 minutes, the reactions were filtered through glass filter paper and were subjected to analysis via LC-MS. The product yield was determined by dividing the corresponding area of the product peak by the total area of peptide containing peaks. The quantum yield was determined by Eq 5.

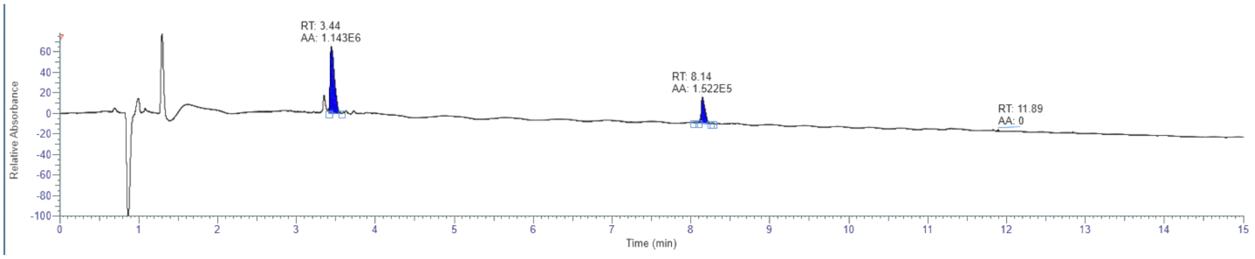

Figure S44. LCMS of 2-minute irradiation reaction and the area integration to determine the yield.

Sample quantum yield calculation:

$$\Phi = \frac{mol\ product}{flux \bullet t \bullet f} \qquad \text{(Eq 5)}$$

(Eq 6)

$$\Phi = \frac{2.4 \bullet 10^{-7} mol}{3.66 \bullet 10^{-9}\ einstein\ s^{-1} \bullet 120 \bullet 1} = 0.55$$

8.3. Isolation and Irradiation of Theoretical Intermediate

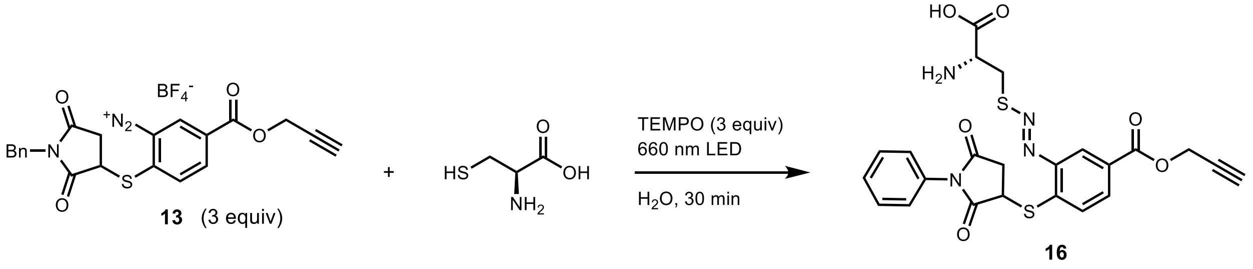

Exact Mass: 479.0734

Exact Mass: 512.08

To a 4-mL clear glass vial, was added 0.5 mL of a 2 µM stock of *L*-Cysteine in water, **13** (3.0 mg, 6 µmol, 3 equiv), TEMPO (3 equiv), and a stir bar. The mixture was further diluted with 1.5 mL water. The reaction was irradiated with two 660 nm Kessil lamps (35 W) at room temperature. After 30 minutes, the reaction was filtered through glass filter paper and was subjected to analysis via LC-MS (Figure S44).

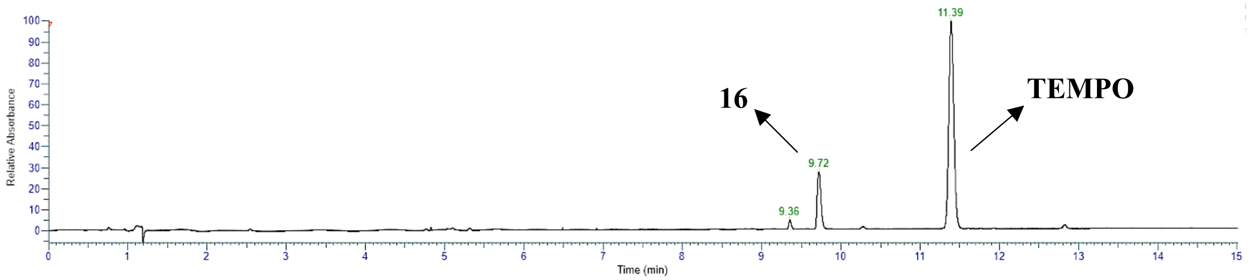

Figure S45. LCMS of crude reaction between **13** and *L*-Cysteine in the presence of 3 equivalence of TEMPO.

Purification of the crude reaction mixture was performed by reversed phase HPLC using Luna® C18 column (250×21.2mm, 10 µm) flow rate = 15.0 mL/min, with an isocratic eluent 40:60 v/v (0.1% v/v FA in MeCN : 0.1% FA TFA in H_2O) over 10 min, followed by 50:50 v/v (0.1% v/v TFA in MeCN : 0.1% v/v TFA in H_2O) over 2 minutes, followed by 65:35 v/v (0.1% v/v TFA in MeCN : 0.1% v/v TFA in H_2O) over 5 minutes, then 100:0 v/v (0.1% v/v TFA in MeCN : 0.1% v/v TFA in H_2O) over 5 minutes. The fractions containing the desired product were collected and lyophilized to afford the title compound **16** as a colorless powder (1 mg, 2 µM, 98% yield) (Figure S45).

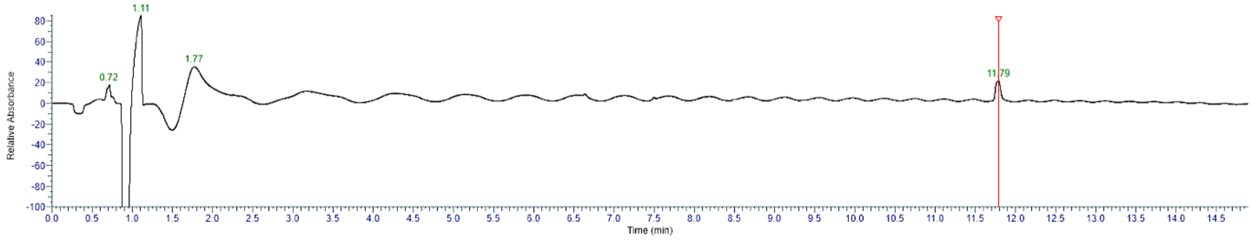

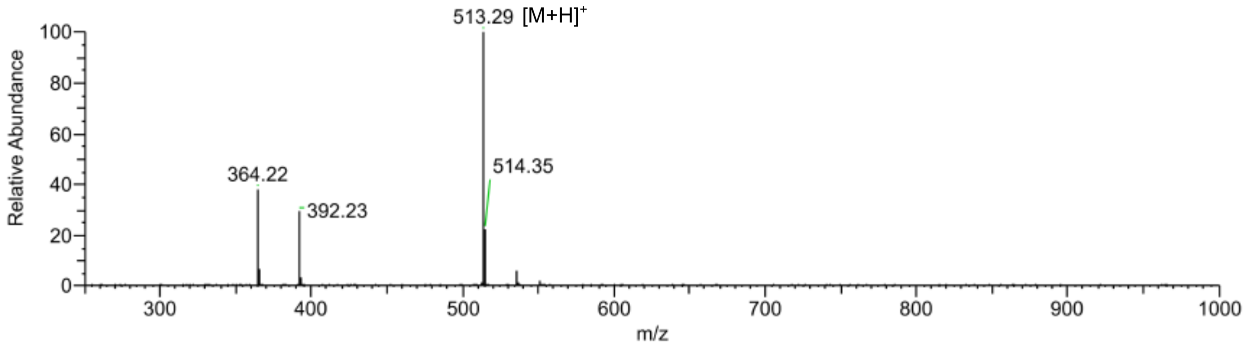

Figure S46. LCMS and ESI-TOF of pure **16**.

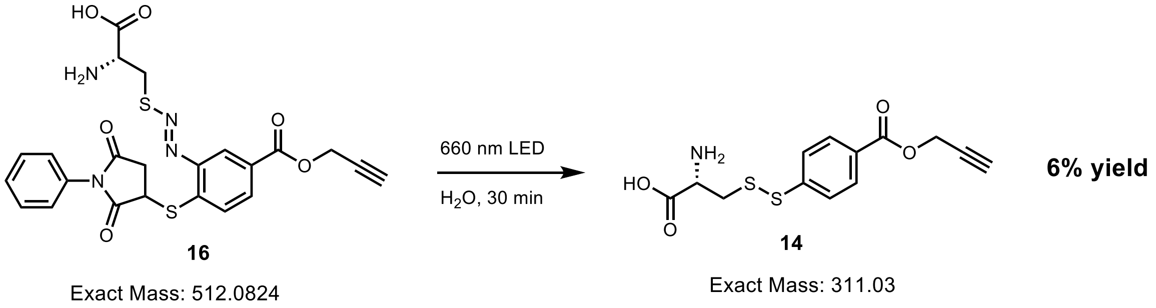

16 (1.0 mg, 1.95 μmol) was dissolved in water (1.9 mL) in a 4-mL clear glass vial equipped with a stir bar. The reaction was irradiated with two 660 nm Kessil lamps (35 W) at room temperature. After 30 minutes, the reaction was filtered through glass filter paper and subjected to analysis via LC-MS (Figure S46).

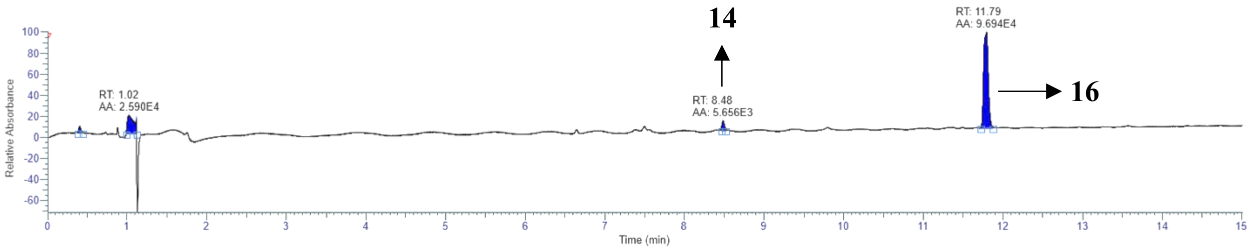

Figure S47. LCMS of **16** after irradiation. The conversion to **14** was calculated by dividing the area under the peak of **14** in the trace by the total area under the peaks (total of **14** and **16**).

8.4. Reaction of 13 in Organic Solvent

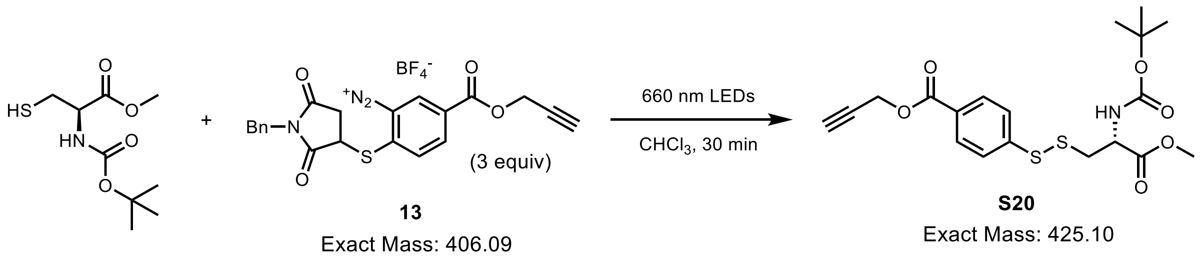

To a 4-mL clear glass vial, was added 2.0 mL of a 2 μM stock of methyl (tert-butoxycarbonyl)-L-cysteinate in chloroform, **13** (3.0 mg, 6 μmol, 3 equiv) dissolved in 3 drops of acetonitrile, and a stir bar. The reaction was irradiated with two 660 nm Kessil lamps (35 W) at room temperature. After 30 minutes, the reaction was subjected to analysis via LC-MS.

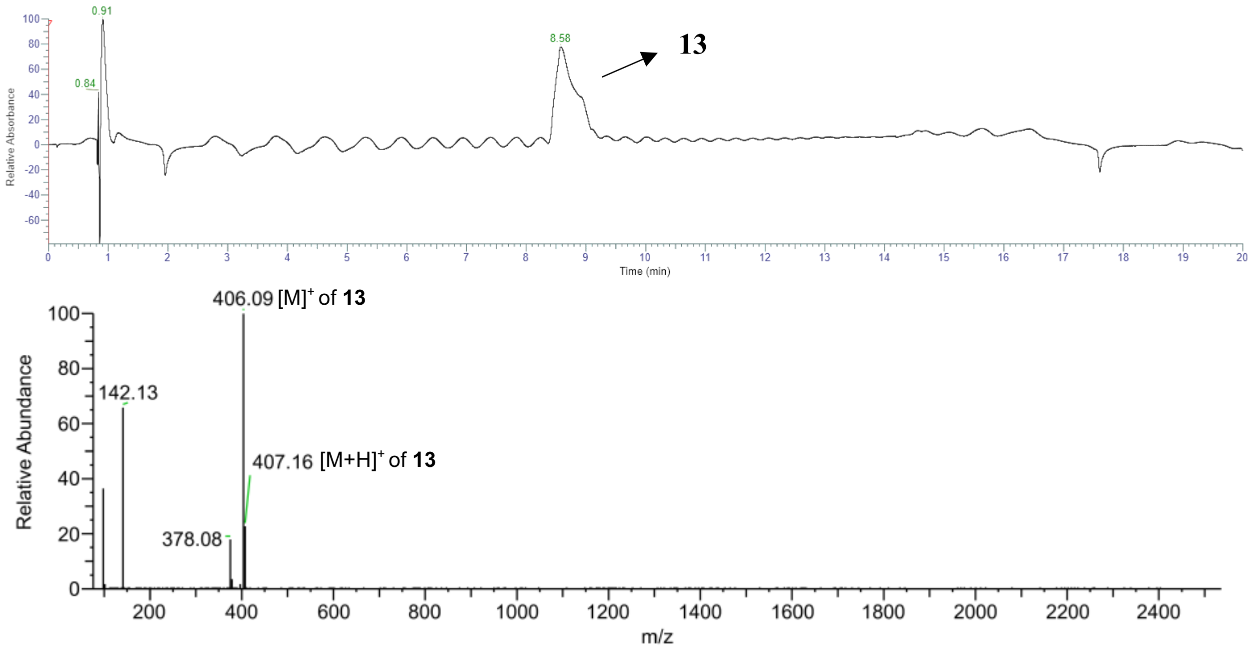

Figure S48. LCMS and ESI-TOF of crude reaction between **13** and Boc-Cys-OMe. The mass of 406.09 m/z represents the mass of **13** without the BF_4 anion.

9. Stability Test of 14 in Buffers and Glutathione

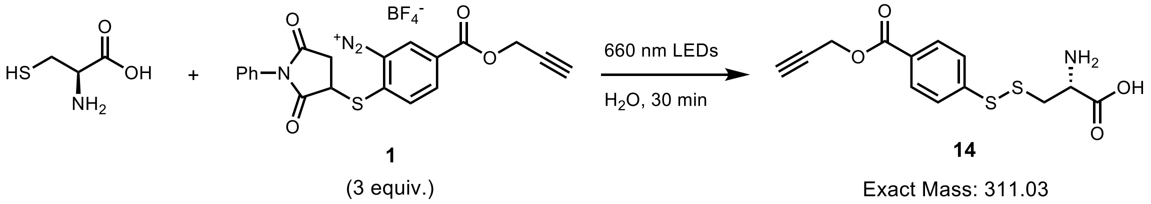

To a 4-mL clear glass vial, was added 0.5 mL of a 4 mM stock solution of *L*-Cysteine in water (1 equiv), was added **1** (3.0 mg, 6 μmol, 3 equiv), 1.5 mL of water, and a stir bar. The reaction was irradiated with two 660 nm Kessil lamps (35 W) at room temperature. After 30 minutes, the sample was filtered through glass filter paper and was subjected to purification via HPLC. Purification of the crude reaction mixture was performed by reversed phase HPLC using Luna® C18 column (250×21.2mm, 10 μm) flow rate = 15.0 mL/min, with an isocratic eluent 40:60 v/v (0.1% v/v FA in MeCN : 0.1% FA TFA in H_2O) over 10 min, followed by 50:50 v/v (0.1% v/v TFA in MeCN : 0.1% v/v TFA in H_2O) over 2 minutes, followed by 65:35 v/v (0.1% v/v TFA in MeCN : 0.1% v/v TFA in H_2O) over 5 minutes, then 100:0 v/v (0.1% v/v TFA in MeCN : 0.1% v/v TFA in H_2O) over 5 minutes. The fractions containing the desired product were collected and lyophilized to afford the title compound **14** as a colorless powder (0.61 mg, 2 μmol, 98% yield).

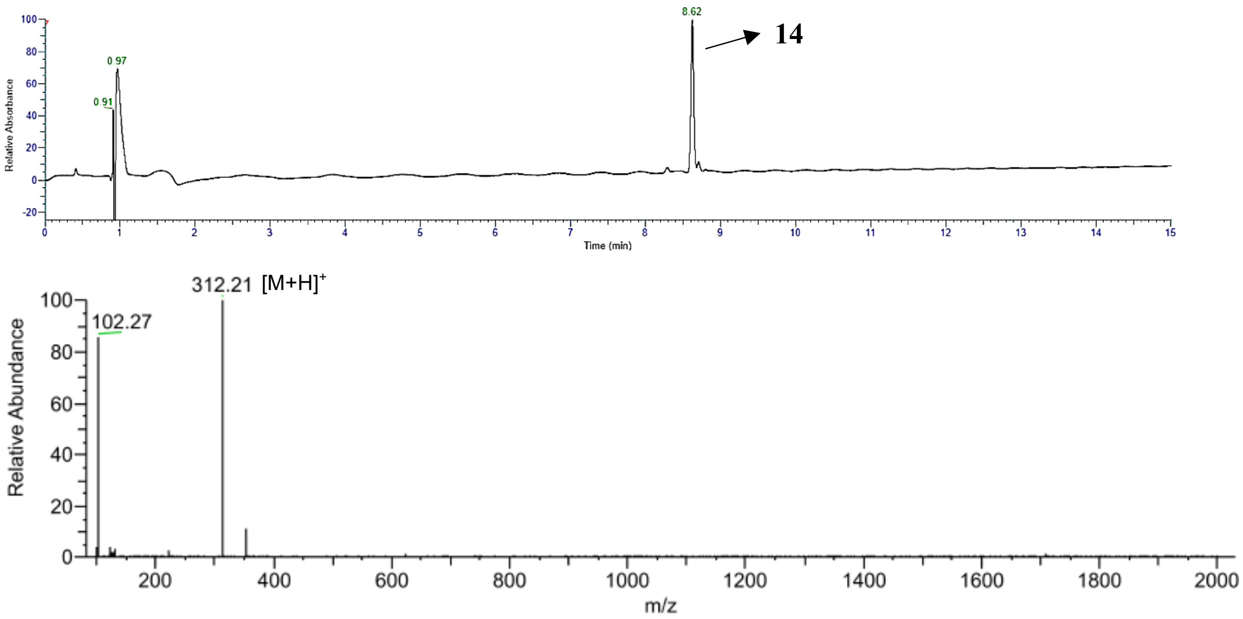

Figure S49. LCMS and ESI of the purified **14**.

14 (2 mg) was dissolved in either water, 1X PBS, or 1X MEM to prepare a 6 mM solution. Glutathione (5 equiv) was added to the prepared stock solution along with an internal standard (1 equiv of *L*-Tryptophan). 20 µL of this was added to a 2 mL vial with 1 mL of water and was subjected to LCMS analysis. This was repeated every 2 hours until the 8-hour timepoint. The last 24 h timepoint was taken the following day. For each timepoint, a product-to-internal standard (IS) ratio was determined by dividing the area under the product peak by the area under the IS peak. The values were then converted to percent of **14** remaining by dividing the area ratio at each timepoint by the area ratio at the 0 h timepoint.

10. Cell Data

10.1. General Cell Culture

HeLa cells were grown in DMEM growth media (GIBCO 11995065) with 10% FBS (GIBCO A5670401) in 75 cm^2 surface treated culture dishes (Fisherbrand FB012937) and were incubated in a cell culture chamber (37 °C with 5% CO_2). Cells were kept at a maximum confluency of 80% and passaged up to 20 times. For each passage, the cells were washed with 1X PBS (GIBCO 20012027), detached from the plate by incubation with 0.25% Trypsin-EDTA (GIBCO 25200072) for 3-4 minutes in the cell culture chamber and then diluted with growth media. The cells were spun down at 300 xg at 4 °C for 5 minutes, resuspended with fresh growth media, and replated in a new flask. After 20 passages, cells were discarded.

10.2. Photolabeling of HeLa Cells for Imaging Via Confocal Microscopy

HeLa cells were washed with 1X PBS and detached from the cell culture dishes by 4-minute incubation with 0.25% Trypsin-EDTA in the cell culture chamber and then diluted with DMEM.

Cells were spun down at 300 xg at 4 °C for 5 minutes. The media was aspirated, and the cell pellet was resuspended in fresh DMEM. The cells were counted and seeded on 35-mm^3 cell culture plates with #1.5 cover glass (Fisher NC0409658) at 300,000 cells/plate in 2 mL DMEM and incubated overnight. 24 hours after the initial seeding, the media in the plates were aspirated and the cells were washed with 2 mL 1X PBS. The buffer was aspirated and 2.5 mL of a 150 μM solution of diazonium **9** in MEM (Gibco 51200038) at pH 6.6 was added to the plates (diazonium solution was prepared from a 4 mM stock in acetone and added to pre-acidified MEM, pH 6.6). Plates were incubated for 20 minutes in a cell culture chamber (37 °C with 5% CO_2) with or without irradiation by a 660 nm Kessil lamp (35 W). The plates were removed from the incubators and the cells were washed with 1X PBS. The buffer was aspirated, and the plates washed again with 1X PBS. The buffer was aspirated and to the cell plates was added 2 mL of membrane and nuclear stain solution (In 1X PBS was added 1:2000 Hoechst 33342 Stain (ThermoFisher Scientific 62249) and 1X CytoPainter Cell Plasma Membrane Deep Red Dye Stain (Abcam ab219942)). The cells were incubated with the stain solution in the cell culture chamber for 10-15 minutes. The cell plates were removed from the incubator and the cells were washed with 1X PBS. The cells were finally layered with 2 mL 1X MEM and immediately imaged by confocal microscopy. Confocal images were acquired in between lines using a Leica SP8 confocal microscope equipped with a 63x glycerol immersion objective (NA = 1.3). Excitation at 405 nm was used to visualize Hoechst 33342 fluorescence, detected by a HyD. Excitation at 495 nm was used to visualize fluorescein fluorescence, detected by a PMT. Excitation at 649 nm was used to visualize CellPainter Cell Plasma Membrane Deep Red Dye fluorescence, detected by a HyD.

10.3. Western Blot Procedure of HeLa Cell Lysate

HeLa cells were washed with 1X PBS and detached from the cell culture dishes by 4-minute incubation with 0.25% Trypsin-EDTA in the cell culture chamber and then diluted with DMEM. Cells were spun down at 300 xg at 4 °C for 5 minutes (all spin downs followed these specifications, unless otherwise stated). The media was aspirated, and the cell pellet was resuspended in fresh DMEM. The cells were counted and 4 million cells were spun down and further washed with 1X PBS and spun down again. The buffer was aspirated and 2 mL of a 200 μM solution of diazonium **12** in 1X PBS pH 6.6 was added to the cell pellet (diazonium working solution was prepared from a 4.5 mM stock of **12** in DMSO and added to a pre-acidified 1X PBS, pH 6.6). The cell-diazonium mixture (2 million cells / mL) was resuspended and into two 1.5 mL Eppendorf tubes was added 1 mL of the cell-diazonium mixture. One of the tubes was irradiated with a 1040 nm laser at room temperature and the other without irradiation in the dark. Additionally, a 1 mL cell-diazonium mixture (2 million cells in a 200 μM solution of diazonium) wrapped in a 4-mm thick bacon layer was also irradiated with the 1040 nm laser. Alongside these samples, a 2 million cells / mL sample in 1 mL 1X PBS pH 6.6 without diazonium was also incubated. After the incubation of all samples, the cells were spun down, and the liquid was aspirated. The cell pellets were resuspended and washed with 1X PBS and subsequently spun down. The pellets were washed again with 1X PBS and spun down. The pellets were then resuspended with RIPA lysis buffer (+ proteases, ThermoFisher Scientific A32961) with a volume of 50 μL buffer / 1 million cells. The samples were incubated on a rotating platform in a 4 °C cold room for 30-45 minutes. The samples were then spun down at 12,000 xg at 4 °C for 20 minutes.

The cell lysate solutions were transferred to pre-chilled Eppendorf tubes and their protein concentrations were measured by a standard BCA Assay (ThermoFisher Scientific 23225). For each sample, 4 μg of cellular protein was diluted with 5X Pierce™ Lane Marker Non-Reducing

Sample Buffer (ThermoFisher Scientific 39001) and loaded in a pre-made 4% stacking 9% resolving SDS-PAGE polyacrylamide gels in Criterion gel cassette. Gel electrophoresis was performed using Mini-PROTEAN Tetra Vertical Electrophoresis Cell tanks and a BioRad PowerPac Basic Power Supply. Gels were run at 80 volts for 20 minutes and then 180 volts for 70-90 minutes using Tris-glycine-SDS running buffer (25 mM Tris, 192 mM glycine, 0.1% SDS). The proteins were transferred from the gel to a PVDF membrane (pre-activated with methanol). For the transfer, the membrane and gel were placed between two extra thick western blotting filter papers, pre-soaked in transfer buffer (25 mM Tris, 192 mM glycine, 20% methanol), sealed in a Mini Gel Holder Cassette and then placed in a Bio-Rad Mini Trans-Blot Electrophoretic Transfer Cell filled with transfer buffer and set to a standard condition of 100V for 120 minutes in a 4 °C cold room. The membrane was removed from the chamber and was then blocked with a 5% w/v milk TBST blocking buffer (5% w/v dry milk powder, 1X TBS, 0.1% Tween-20, pH 7.8) on a rotating platform for 1 hour. The blocking buffer was removed, and the membrane was immersed in a 1:1500 α-biotin HRP antibody (Cell Signaling 7075S) solution in blocking buffer and allowed to incubate on a rotating platform at room temperature for 1 hour. The antibody solution was decanted, and the membrane was washed with TBST buffer on a rotating platform at room temperature for 20 minutes. The solution was decanted, and the TBST wash step was repeated two additional times. The final wash solution was decanted, and the membrane was layered with 1 mL Bio-Rad Clarity™ Western ECL Substrate Peroxide Solution and 1 mL Bio-Rad Clarity™ Western ECL Substrate Luminol/Enhancer Solution and incubated for 30-45 seconds. The membrane was immediately imaged using a UV tray in a Bio-Rad ChemiDoc Imaging System. For total cellular protein, gels were incubated in Coomassie solution (5:4:1 methanol:ddH2O:acetic acid with 0.05% Coomassie) for 1 hour, decanted, and then incubated in a destain solution (5:4:1 methanol:ddH2O:acetic acid) overnight. The gels were imaged using a white tray in the Bio-Rad ChemiDoc Imaging System.

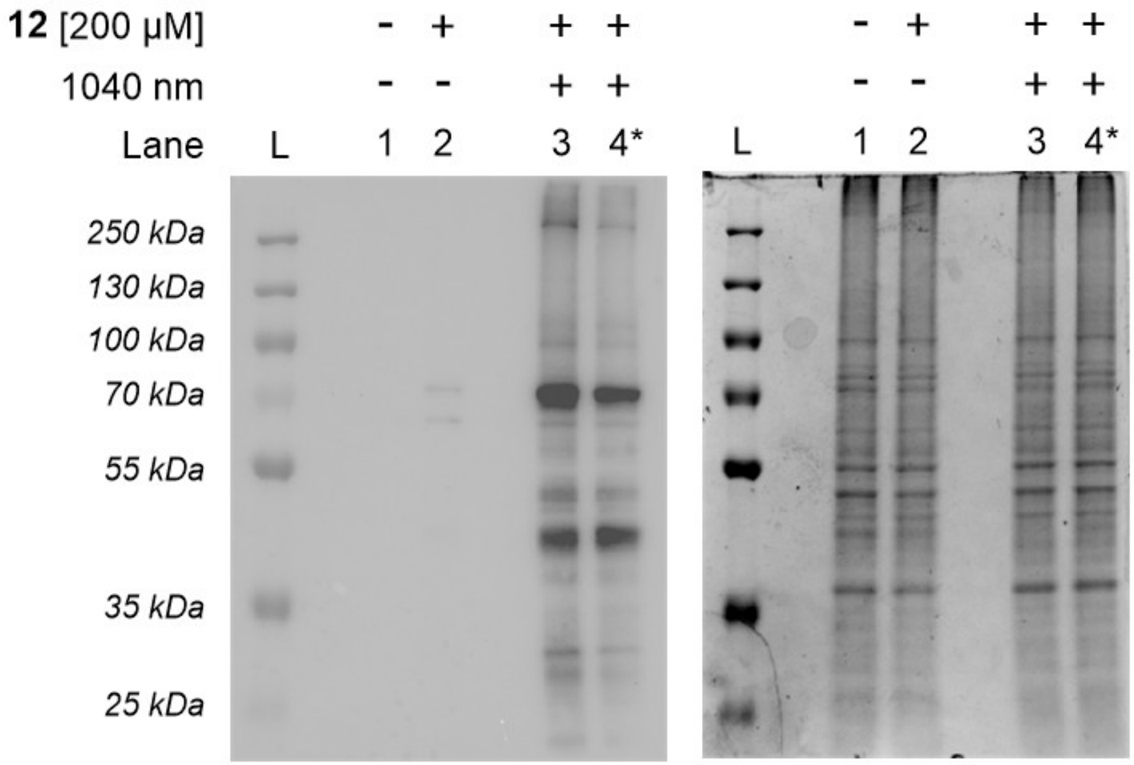

Figure S50. Western Blot Assay of Total Protein from cells irradiated with 1040 nm IR laser in the presence of **12**. Lane L: PageRuler™ Plus Prestained Protein Ladder (ThemoFisher Scientific 26619); Lane 1: HeLa cells in the absence of **12** and with no irradiation; Lane 2: HeLa cells in the presence of **12** with no light irradiation in the dark; Lane 3: HeLa cells irradiated in the presence of **12** and with 1040 nm IR laser irradiation. Lane 4: HeLa cells wrapped with 4 mm bacon layer and irradiated in the presence of **12** and with 1040 nm IR laser irradiation.

10.4. Photolabeling of MCF 10A and MDA-MB-231 cells

MCF 10A and MDA-MB-231 cells were washed with 1X PBS and detached from the cell culture dishes by 4-minute incubation with 0.25% Trypsin-EDTA in the cell culture chamber and then diluted with DMEM. Cells were spun down at 300 xg at 4 °C for 5 minutes. The media was aspirated, and the cell pellet was resuspended in fresh DMEM. The cells were counted and seeded in a 6 well plate with cover glass in each well at 25,000 cells per well and incubated at 37 °C, 5% CO_2. 24 hours after the initial seeding, the media in the plates was aspirated and the cells were washed with 2 mL 1X PBS. The buffer was aspirated and 2.5 mL of a 150 µM solution of diazonium **1** in MEM (Gibco 51200038) at pH 6.6 was added to the plates (diazonium solution was prepared from a 4 mM stock in acetone and added to pre-acidified MEM, pH 6.6). Cells were incubated for 15 minutes with or without irradiation by a 1064nm laser (Thorlabs L1064H2).. Following irradiation, dosage media was removed, and the cells were washed with 1X PBS for five minutes, three times total. A 4% PFA solution in PBS was freshly prepared and 2 mL was added to each well and incubated for 10 minutes. The PFA solution was removed, and cells were washed with PBS for 5 minutes, three times. A 0.1% Triton X-100 solution was freshly prepared, and 2 mL was added to each well and incubated for 10 minutes. The Triton X-100 solution was

removed, and cells were washed with PBS for 5 minutes, three times. To a freshly prepared cocktail of 2 mM $Cu(SO_4)_2$, 10 mM THPTA, and 30 mM sodium ascorbate in 10 mL PBS was added 20 µL of a 10 mM stock solution of Cy5 azide. Cells were incubated in this solution for 2 hours. The dosage media was removed, and cells were washed with PBS for five minutes, three times total. Cells were mounted onto slides with Flouroshield with DAPI. Cells were imaged on a Stellaris® 8 Leica DMi8 microscope (20x objective) with fast lifetime contrast (FALCON) module. Samples were excited using a 40 MHz pulsed white light laser tuned to 405 and 633 nm with sequential acquisition. Emitted photons were detected using HyD® S and HyD® X (GaAsP hybrid photocathode). Images were processed and analyzed using ImageJ software. Scale bar = 25 µm.

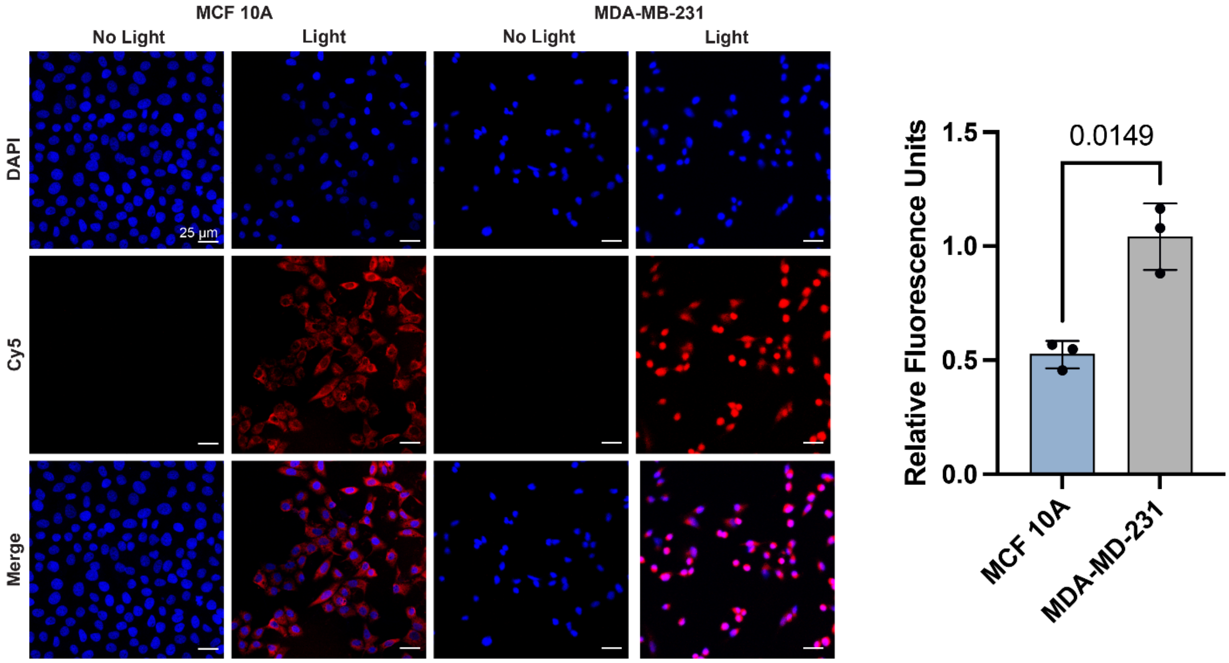

Figure S51. Confocal images of MCF 10A and MDA-MB-231 cells treated with TagC-RED alkyne probe **1** with and without 1064 nm laser, followed by fixation and Click labelling with Cy5 azide. Depicted scale bar is 25 µm. Error bars represent mean ± S.D.

11. Animal Studies

Adult male and female TH-Cre+ (B6.Cg-*7630403G23Rik*$^{Tg(Th\text{-}cre)1Tmd}$/J, The Jackson Laboratory, #:008601) were used in this experiment. These mice had previously received surgery for a bilateral infusion of AAV5-Ef1a-DIO-hTyr into the locus coeruleus (LC) as part of an ongoing study. During surgery, mice were anesthetized with isoflurane and injected with 0.5 mg/kg ketoprofen to minimize pain. Animals were maintained on a 12:12 light:dark cycle (lights on at 0700). Food and water were *ad Libitum*. All experiments were conducted at Emory University in accordance with the National Institutes of Health Guideline for the Care and Use of Laboratory Animals and approved by the Emory Institutional Animal Care and Use Committee.

11.1 *Ex vivo* Brain Labelling

Mice were sacrificed via rapid decapitation under anesthesia via isoflurane. Brains were isolated, dipped into cold isopentane before being fixed by 4% paraformaldehyde solution in PBS for 24 h followed by soaking for 48 h in 30% sucrose solution in a 4 °C refrigerator. Brains were then incubated in 2 mL of 150 μM of **9** (4% DMSO in 1X PBS (10 mM, pH 6.6)) for 5 minutes, then exposed to two Kessil pro160 740 nm LED lights (35W) for 30 minutes. Control brains received the same dosage of probe, but were covered in aluminium foil and not exposed to light. After irradiation, the probe solution was removed and 2 mL of 1X PBS (10 mM, pH 6.6) was added to each brain sample. Brains were rocked for 1 h to remove any unreacted probe. Wash solution was removed and this washing process was repeated two more times. After washing, brains were embedded in OT medium (Tissue-Tek) and sectioned by cryostat into 60 μM thick coronal sections at the level of the locus coeruleus (LC), substantia nigra (SN), and prefrontal cortex (PFC). Sections were immediately transferred to glass Superfrost Plus slides which were then cover slipped with Fluoromount-G (Southern Biotech, Birmingham, AL) and allowed to dry. Slides were wiped with EtOH wipes before imaging.

Tissue slices were imaged on a Stellaris® 8 Leica DMi8 microscope (5x objective). Samples were excited using an 80 MHz pulsed white light laser tuned to 490 nm. Emitted photons were detected using HyD® X (GaAsP hybrid photocathode).

11.2 *In vivo* Liver Labelling

Mice were placed under anesthesia via isoflurane. Mouse hair was removed via Nair treatment, then transcardially perfused with PBS (20 mL, 10 mM pH 6.6) to remove blood, then perfused with 20 mL of 150 μM of **1** (4% DMSO in 1X PBS (10 mM, pH 6.6)). Mice were exposed to 1064 nm laser (Thorlabs L1064H2) for 15 minutes or stored under aluminum foil for 15 minutes for no light treatment. Livers were excised and stored in 4% PFA in PBS solution in a 4 °C refrigerator for 24 h. Livers were then dosed with 1.5 mM THPTA, 1 mM $CuSO_4$, 500 μM Cy5-Azide (BroadPharm, Catalog #BP-23908), and 5 mM sodium ascorbate in 10 mL PBS (10 mM, pH 7.4) and rocked overnight in the dark. Click reagents were removed, and livers were washed with PBS (10 mM, pH 7.4) containing 1 mM EDTA and 0.1% Triton X-100 for ~4 hours, repeated 5 times over ~36 hours. Livers were then imaged via IVIS® Spectrum Series Imaging System. Excitation was set to 640 nm and emission was 680 nm with FOV = C, height = 1.0 cm, and exposure set to auto. Images were analyzed via Living Image™ software to determine radiant efficiency. Data was analyzed and plotted in GraphPad Prism. This was repeated in triplicate.

12. Computational Studies

12.1 Computational Methods

Gaussian 16 revision C.01 was employed for the optimization of all stationary points, calculation of single-point energy corrections and TD-DFT calculations.[9,10] Harmonic vibrational frequency calculations were performed to obtain thermal corrections to Gibbs free energies (G) as well as to verify the nature of the stationary points. For numerical integration of the exchange-correlation functionals in all Gaussian calculations, the default “ultrafine” pruned (99,590) grid was utilized. Geometry optimizations of stationary points and TD-DFT were performed using the range-

separated hybrid ωB97X-D functional and the Pople split-valence double-ζ basis set 6-31+G(d).[11,12] These optimizations were conducted with the SMD solvation model to simulate an aqueous solvation environment.[13] In case of an imaginary frequency, Intrinsic Reaction Coordinate (IRC) calculations were performed to verify that the TSs connect the reactants to the products.[14]

Single-point energy corrections were calculated with the ωB97X-D functional (a range-separated hybrid with D2 empirical nonlocal correlation) and the triple-ζ def2-TZVPD basis set, maintaining the same SMD solvation model.[15,16] The selection of density functional approximations (DFAs) was informed by the good accuracy of the range-separated ωB97 family for ground-state geometries and energies, as benchmarked against the GMTKN55 database.[17] Additionally, these functionals include explicit long-range dispersion corrections necessary for the accurate description of non-covalent interactions. The computed thermochemistry and kinetics of all computed elementary steps were compared using multiple density functionals for single-point corrections (SPC). The results were consistent across the selected functionals (refer to Table S6 and Figure S50).

Orca 6.0 was used for conformational sampling using the Global Optimizer Algorithm (GOAT) in combination with the semiempirical GFN2-xTB level of theory. Conformers were clustered based on geometry using the CREGEN module of CREST. This workflow was used alongside manual conformer searching to generate the conformational space for the non-covalent EDA complexes, as well as the structures for the post electron transfer mechanism. The Δ-SCF approach implemented in Orca 6.0 was used to calculate absorption wavelengths and perform excited state optimizations for the lowest excited state using TD-DFT transition orbitals for all the donor-acceptor associative complexes. In addition, the Resolution of Identity (RI) approximation was applied to Coulomb integrals using an auxiliary basis set and a numerical chain-of-sphere integration applied to Hartree Fock exchange integrals (RIJCOSX) to significantly speed up hybrid DFT calculations.[18]

GoodVibes[19] was used to implement Grimme's quasi-harmonic approximation using a cutoff frequency of 100 cm^{-1} to correct for errors in vibrational entropy calculations due to low frequencies.[20] The concentrations of all species were adjusted from 1 atm to a 1 M standard state in solution. Boltzmann-weighted Gibbs energies (G_{conf}) were obtained to account for the presence of multiple thermally accessible low-lying conformers and include terms for the entropy of mixing. Reported Gibbs energies (G) correspond to Boltzmann-weighted values.

Input files for Orca single-point energy calculations were generated automatically using the *qprep* function in AQME.[21] 3D molecular graphics were rendered using the Pymol software.[22]

a) Gaussian 16 optimization (keywords):

```
# opt freq wb97xd/6-31+g(d) scrf=(smd,solvent=water)
```

*For TS optimization the keyword "opt" was replaced with "opt=(ts,calcfc,noeigen).

b) Gaussian 16 single point (example file):

```
# wb97xd/gen scrf=(smd,solvent=water)
```

```
Title

0 1
 C             -1.77836800   -1.04131400   -0.00006100
 C             -0.40692700   -1.28118800   -0.00001000
 C              0.51073700   -0.22423300    0.00006400
 C              0.01583100    1.08760300    0.00008600
 C             -1.35216900    1.32849900    0.00002600
 C             -2.25617800    0.26552100   -0.00004600
 H             -2.47141000   -1.87569300   -0.00011000
 H             -0.03783400   -2.30241700   -0.00001200
 H              0.70135200    1.92822800    0.00016700
 H             -1.71693400    2.35003400    0.00005300
 H             -3.32347600    0.45788700   -0.00009900
 C              1.95338500   -0.53674800    0.00009800
 H              2.18951900   -1.59906000    0.00034900
 C              2.95842700    0.33997200   -0.00013800
 H              2.80276100    1.41435800   -0.00039100
 H              3.98759600   -0.00201800   -0.00007500

User-defined basis set corresponding to def2-tzvpd for elements C and H
```

c) User-defined basis set for def2-TZVPD (with H atom as example):

```
H     0
S    3   1.00
    34.0613410            0.60251978D-02
     5.1235746            0.45021094D-01
     1.1646626            0.20189726
S    1   1.00
     0.32723041           1.0000000
S    1   1.00
     0.10307241           1.0000000
P    1   1.00
     0.8000000            1.0000000
P    1   1.00
     0.95774129632D-01      1.0000000
****
```

*Basis sets for all the elements in the calculation are appended in this way. These user-defined basis sets are obtained from the Basis Set Exchange.

d) TD-DFT keyword:

```
# wb97xd/6-31+g(d) scrf=(smd,solvent=water) td=(nstates=5)
```

e) GoodVibes (terminal command):

```
python -m goodvibes *log --spc mv -c 1 --pes pes_vpd.yaml --graph pes.yaml
```

f) AQME (terminal command for generating SPC input files):

```
python -m aqme --qprep --program "gaussian" --files "*log" --qm_input " wb97xd/gen scrf=(smd,solvent=acetonitrile)" --
mem "64GB" --nprocs "32" –suffix sp_xd
```

12.2 Model system for computational mechanistic studies

In this study, a key challenge is the extensive conformational sampling required to accurately describe highly flexible EDA complex. We employed a truncated model system in computational studies that preserves the electronic features central to EDA formation and photoexcitation, while reducing the conformational complexity. For the donor species, we modeled a truncated cysteine

peptide capped with *N*-acetyl groups. We considered the potential involvement of neutral and deprotonated thiol sidechains (Figure S50).

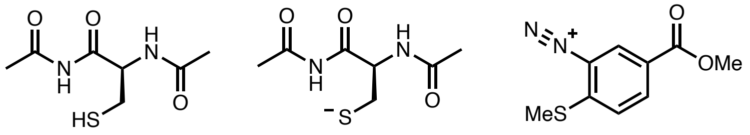

17 **18**

Figure S52: Structures used in computational modeling studies.

12.3 Explicit water solvation

We examined the extent to which the inclusion of explicit water molecules, as opposed to an implicit solvation model, influenced the computed electronic properties of the system. Specific intermolecular interactions between a protic solvent and solute, such as H-bonding interactions, are expected to stabilize charged species and thus we envisaged that predictions of redox potentials might be particularly susceptible to the description of solvent. We used the free energy of reduction (ΔG°_{red}) of the donor and acceptor—computed both with and without explicit water molecules—as a metric to quantify the influence of explicit solvation.

An anionic cysteine donor contains three basic functional groups capable of engaging in hydrogen bonding, which can, in principle, accept up to two hydrogen bonds, suggesting that a minimal first hydration shell would involve approximately six water molecules. We therefore began by adding six explicit water molecules around the anionic cysteine and then systematically varied this number to probe whether additional solvent molecules further influence electronic and redox properties (Table S6).

n H_2O	*0*	*6*	*7*	*8*
ΔG_{red}	-103	-115	-114	-113

Table S6. Variation in the reduction free energy (ΔG°_{red}) of cysteine as a function of the number of explicit water molecules included in the model.

We observe that the reduction free energy (ΔG°_{red}) changes substantially, by more than 10 kcal mol^{-1}, when moving from an implicit SMD treatment to explicit solvation. This shift confirms that specific solute–solvent hydrogen-bonding interactions play a critical role in stabilizing the cysteine anion, and therefore directly influence its electronic and photophysical properties. The predicted value of ΔG°_{red} has effectively converged by six water molecules, which is therefore sufficient to capture the dominant effects of explicit solvation on the redox behaviour of cysteine.

We carried out the same analysis for the diazonium acceptor (Table S7). In this case, introducing explicit water molecules produces only minimal changes in ΔG°_{red}. In contrast to cysteine, where strong, localized solute–solvent hydrogen bonds significantly stabilize the anionic donor, solvation of the acceptor can be adequately captured by the implicit continuum model.

$n\ H_2O$	*0*	*2*	*3*	*4*
ΔG_{red}	-105	-104	-104	-104

Table S7. Variation in the reduction free energy ($\Delta G°_{red}$) of the diazonium acceptor as a function of the number of explicit water molecules.

Consequently, for all subsequent modelling of EDA complex formation and their photophysics, our explicit solvation protocol incorporates six water molecules on the cysteine donor.

12.4 Marcus barriers for outer-sphere electron transfer

Thermal activation barriers for all outer-sphere electron-transfer (ET) processes were evaluated using the Marcus–Hush model.[23] In this framework, the activation free energy depends on two key quantities: (a) the thermodynamic driving force ($\Delta G°$) between the reactant (pre-SET) and product (post-SET) states, and (b) the overall reorganization energy ($\boldsymbol{\lambda}$), which comprises both the internal ($\boldsymbol{\lambda_i}$) and solvent ($\boldsymbol{\lambda_o}$) components. These terms are related through the standard Marcus expression:

$$\Delta G^{\ddagger} = \frac{(\lambda + \Delta G°)^2}{4\lambda}$$

DFT calculations were performed on the infinitely separated donor and acceptor species to evaluate both ΔG^o and λ. The free energy difference is obtained directly from the difference between the optimized geometries of the reactant and product states. The reorganization energy is computed by performing a single-point calculation on the optimized product geometry and solvation environment, while constraining the electronic configuration (i.e., charge and spin multiplicity) to that of the reactant state. This procedure yields the total $\lambda = \lambda_i + \lambda_o$. Specifically, computing the product geometry with the reactant electronic state accounts for the internal contribution (λ_i), representing the energetic penalty of distorting the molecular geometry from the reactant's optimized structure toward that of the product while maintaining the reactant's electronic state. Performing this calculation in the solvation environment of the product accounts for the solvent contribution (λ_o), which accounts for the energetic cost of reorganizing the surrounding medium. Computationally, this is achieved using 'Non-Equilibrium' solvation, where the solvation environment of the product is first saved in a checkpoint file using the keyword 'NonEq=Save', and subsequently employed in the single-point calculation with the reactant electronic state using 'NonEq=Read'.

We illustrate the workflow for λ calculation using the example of a thermal single-electron transfer (SET) from the cysteine anion (Cys^-) to the diazonium acceptor (A^+).

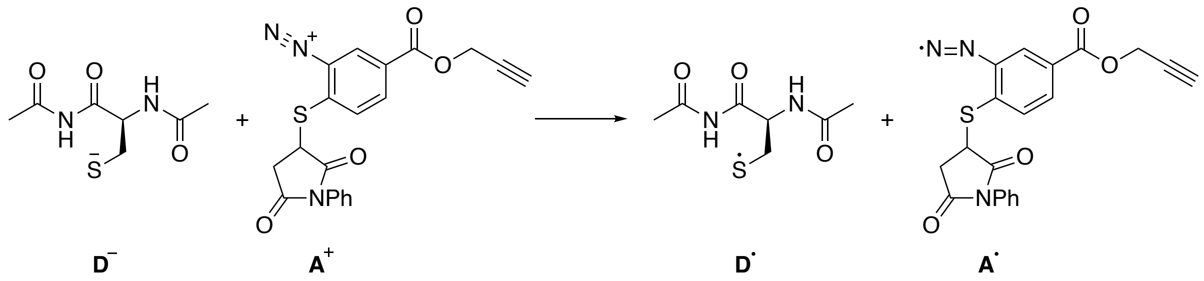

Figure S53: Structures used in computational modeling studies.

To compute the reorganization energy λ, four single-point calculations are carried out as follows:

Reactant-state electronic energies (before SET)

1) **E_1 : Cys**$^-$ at its optimized geometry and solvation with charge = -1 and multiplicity =1

2) **E_2 : A**$^+$ in its own optimized geometry and solvation with charge = +1 and multiplicity =1

Product-state electronic energies (after SET), with reactant electronic configuration

E_3 : Cys$^-$ computed at the optimized geometry of neutral Cys (post-SET), but with the reactant-state electronic configuration (charge = –1, multiplicity = 1). Non-equilibrium solvation is applied using the solvent response saved from the charge = 0, multiplicity = 2 calculation via NonEq=Save, and read into the new calculation using NonEq=Read

3) **E_4 : A**$^+$ computed at the optimized geometry of neutral **A**$^\cdot$ (post-SET), but with the reactant-state electronic configuration (charge = +1, multiplicity = 1). Non-equilibrium solvation is applied using the solvent response saved from the charge = 0, multiplicity = 2 calculation via NonEq=Save, and read into the new calculation using NonEq=Read

With these four single point energies, λ is calculated as follows:

$\lambda = ((\mathbf{E_3} - \mathbf{E_1}) + (\mathbf{E_4} - \mathbf{E_2}))$;

where ($\mathbf{E_3} - \mathbf{E_1}$) and ($\mathbf{E_4} - \mathbf{E_2}$) are the reorganization energies of **Cys**$^-$ and **A**$^+$.

A corresponding set of calculations can be carried out for the post–electron-transfer species. Under the Marcus assumption that the reactant and product free-energy surfaces possess identical parabolic curvature, the reorganization energies obtained from either side should, in principle, be equivalent. In practice, however, the forward and reverse evaluations of λ generally differ. Following Nelsen's four-point protocol, we therefore compute both values and use their geometric mean as the final estimate of the total reorganization energy.[24] The resulting ΔG° and λ parameters

are then used to compute the Marcus activation barrier. However, because this protocol evaluates the barrier for electron transfer within the pre-assembled donor–acceptor complex, we must also account for the thermodynamic cost of bringing the two species together from infinite separation to form the complex ($\Delta G^{\circ}_{complex}$). Inclusion of this term yields the total activation barrier for the thermal electron-transfer process.

Before examining photochemical pathways, we first evaluated the feasibility of thermal electron-transfer between the donor and acceptor species to determine whether any component of the reaction could occur in the absence of light. In implicit solvent, we assessed electron transfer from both the neutral and anionic forms of cysteine. With neutral cysteine as the donor, the thermodynamics are highly unfavourable (ΔG° = +36.5 kcal/mol), leading to an insurmountable Marcus activation barrier ($\Delta G^{\ddagger}$ = 54.0 kcal/mol). In contrast, electron transfer from anionic cysteine is nearly thermoneutral (ΔG° = –2.4 kcal/mol), with a correspondingly lower activation barrier of 24.2 kcal/mol (Table S8).

In both cases, the reorganization energy is large, consistent with the substantial geometry change of the diazonium group as it changes from linear to bent upon reduction. We next evaluated the barrier in the presence of explicit water molecules (six H_2O molecules hydrogen-bonded to cysteine, as identified in our solvation study). Consistent with our explicit-solvent results, the thermodynamics become markedly more uphill (ΔG° = +7.7 kcal/mol), yielding a higher activation barrier of 29.5 kcal/mol. As anticipated, this demonstrates the significant stabilizing effect that hydrogen-bonding interactions have on the cysteine anion, which suppress thermal electron transfer. Together, these results confirm that in aqueous environments, the chemistry cannot proceed via a thermal electron transfer pathway and must instead rely on photoinduced pathways. More importantly, we see the crucial role explicit solute-solvent interactions play on the redox properties of the system.

electron donor	ΔG°	λ	$\Delta G_{complex}$	$\Delta G^{\ddagger}$
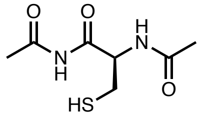	36.5	100.4	7.3	54.0
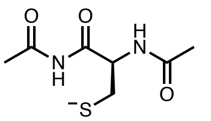	-2.4	82.0	4.9	24.2
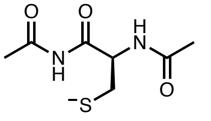 explicit solvent	7.7	84.0	4.5	29.5

Table S8. Marcus activation barriers for neutral and anionic cysteine under implicit solvation, and for anionic cysteine with explicit aqueous solvation.

12.5 Donor-acceptor associative complexes

Based on the results of the thermal electron-transfer analysis, we performed DFT screening (with implicit solvation) to identify possible donor–acceptor assemblies between an anionic cysteine donor and diazonium acceptor. This analysis revealed three distinct classes of complexes (Figure S52).

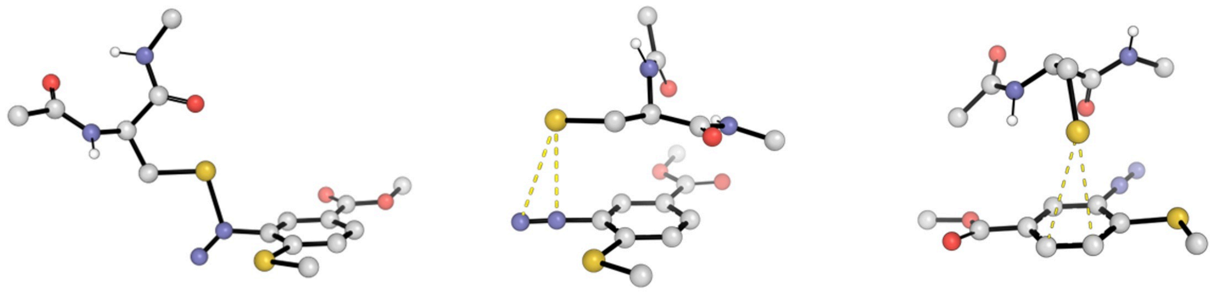

Figure S54. Representative geometries of distinct types of donor-acceptor complexes and their ensemble averaged free energies (kcal/mol).

The first type of structure found (A) resulted from collapse of the charged donor and acceptor to form a covalently bound structure. The second (B) is a charge-separated noncovalent complex, in which the S lone pair lies close in space with the diazonium group, interacting through a charge-transfer interaction with the π_{NN}* orbital. Owing to the cylindrical symmetry of the diazonium group, the cysteine sulfur can approach from a wide range of orientations, resulting in a large ensemble of possible coordination geometries and conformers. The third (C) is also a charge-separated noncovalent complex, in which the S atom lies above/below the aryl ring, engaging in a charge-transfer interaction with the aryl π* LUMO.

Conformational ensembles of these complexes were obtained using the GOAT algorithm with the semi-empirical GFN2-xTB method prior to DFT optimization. At the GFN2-xTB level of theory, covalent adducts (type A) are much more stable, and in the absence of restraints all optimizations of type B and C complexes collapse to these adducts. To circumvent this issue during conformational sampling of these types of complex, we inhibited pyramidalization at any of the electrophilic centers, which prevents bond formation with the cysteine sulfur, while still preserving the flexibility to generate a representative conformational ensemble for the non-covalent complexes. DFT optimizations subsequently were used to optimize these noncovalent complexes in the absence of any restraints.

We initially proceeded using implicit solvation for these studies, finding complex **19** is the most thermodynamically favorable, indicating that charge recombination is favorable for this theoretical model. This covalent adduct is not expected to absorb in the near IR region (which was confirmed

by TD-DFT calculations) and so, we proceeded to examine the EDA complexes with explicit water molecules to investigate whether the same energetic preference is observed. Given the highly flexible nature of these systems and the slow convergence associated with explicit-solvent optimizations, we restricted our analysis to the two lowest-energy families: the ion-collapse complex **19** and the charge-separated complex **20**. Using the conformer-searching protocol detailed above, we generated the conformational ensembles for both complexes in the presence of six explicit water molecules. Geometry optimizations were performed using the 'Opt=Loose' keyword to obtain stable convergence. The inclusion of explicit water molecules proves to be critical: complex **20** is more stable than adduct/complex **19** by 3.2 kcal/mol (Fig S52), due to stabilization of the charge-separated complex.

12.6 State-specific modeling of excited state energies using ΔSCF

Linear Response TD-DFT systematically overestimates the energies of charge-transfer and charge-separated states. Previous studies and benchmarks have demonstrated that state-specific excited state methods, such as ΔSCF, generally provide a more reliable description and energetics for charge transfer excitations.[25] Accordingly, we used ORCA's implementation of the ΔSCF approach to compute onset excitation energies for the explicitly solvated donor-acceptor complexes.[26] In this protocol, TD-DFT is first employed to identify the orbital transitions that constitute the first excited state (S_1). These are listed in the output under 'Excited State 1' which provides the dominant one-electron excitations contributing to the S_1 configuration. From this analysis, we determine the electronic configuration for the S_1 state. This excited state configuration is then used to variationally optimize the targeted excited-state and determine the corresponding SCF energy. This procedure yields a fully relaxed excited-state wavefunction and energy, incorporating orbital relaxation effects essential for accurately describing charge-separated excited states.

We demonstrate this protocol using the S_1 excited states of complexes **19** and **20**. For complex **19**, TD-DFT analysis shows that the onset excitation is dominated by a (HOMO–1) → LUMO transition. This corresponds to an electronic configuration in which the α-spin (HOMO–1) orbital becomes unoccupied while an α-electron is promoted into the LUMO. Inspection of the frontier α orbitals gives us the following configuration: (HOMO–1) has an occupancy of 0, whereas HOMO and (HOMO+1) each have occupancies of one. In ORCA notation, this is encoded as a **0,1,1** excited-state configuration.

Accordingly, the ΔSCF calculation for complex **19** is invoked using the following keywords:

```
!pal16 wB97X-D4 def2-svpd DELTASCF UKS
%scf
ALPHACONF 0,1,1
end
```

Meanwhile, the S_1 excited state of complex **20** is dominated by a HOMO → LUMO transition. In this case, a single electron is promoted from the α -HOMO into the α -LUMO, leaving the α-

HOMO with zero occupancy and the α-LUMO with occupancy=1 in the excited state. Hence, the corresponding ΔSCF configuration is specified as **0,1**, which instructs ORCA to variationally optimise the excited state with an empty HOMO and singly occupied LUMO.

The ΔSCF calculation for complex **19** is invoked using the following keywords:

```
!pal16 wB97X-D4 def2-svpd DELTASCF UKS
%scf
ALPHACONF 0,1,
end
```

The convergence of these excited-state calculations is often challenging, as the SCF procedure can readily collapse back to the ground-state wavefunction. To mitigate this, we employ the NoSOSCF convergence algorithm and supply the ground-state wavefunction as an initial guess, while increasing the maximum number of SCF iterations to 400.

From this analysis, we obtain the onset excitation energies (and corresponding wavelengths) for the conformers of complexes **19** and **20**. As expected, complex **19** shows consistently high-energy onsets, with all absorptions lying below 550 nm, in line with its established behavior (Figure S53). Due to the excited state configuration of **19**, only a small fraction of its ΔSCF calculations converged, leading to a lower number of data points. Complex **20** exhibits dramatically lower excitation energies (i.e., longer wavelengths), with absorption features extending deep into the near-IR and reaching wavelengths of 1300 nm. Importantly, the onset wavelengths predicted for complex **20** align strongly with the experimentally observed reactivity at 1040 nm. State specific ΔSCF treatment of the charge separated excited state shows the near-IR photoactivity of the EDA complex which is otherwise missed with the linear response TD-DFT analysis.

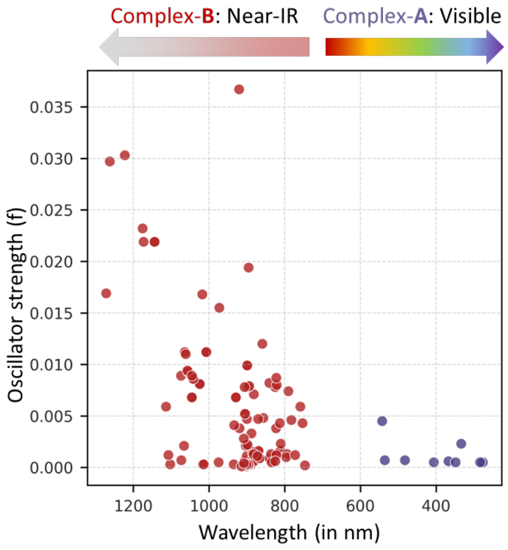

Figure S55. ΔSCF calculated onset absorption wavelength of complexes **19** and **20** with explicit solvation.

We examined whether this photoexcitation corresponds to electron transfer from the donor to the acceptor.
The S_1 excitation in complex **20** is described by a HOMO → LUMO transition: the HOMO is localized almost entirely on the S atom of the cysteine donor, whereas the LUMO resides predominantly on the diazonium acceptor. Given that the $S_0 \rightarrow S_1$ excitation promotes an electron from the HOMO into the LUMO, this orbital picture clearly indicates that the photoexcitation corresponds to charge transfer from the donor to the acceptor (Figure S54).

After the qualitative frontier-orbital analysis, we next examined the spin and charge distribution of the S_1 state, followed by excited-state geometry optimization. The Löwdin charge and spin analyses reveal that the vertically excited S_1 state corresponds to a biradical species: the acceptor bears a spin density of –0.97 (β), while the donor carries +0.97 (α), and both fragments are essentially neutral in charge. Moreover, upon optimization, the S_1 geometry relaxes to a structure in which the diazonium unit adopts a bent geometry of 126°, consistent with the expected structure of the reduced diazonium species.

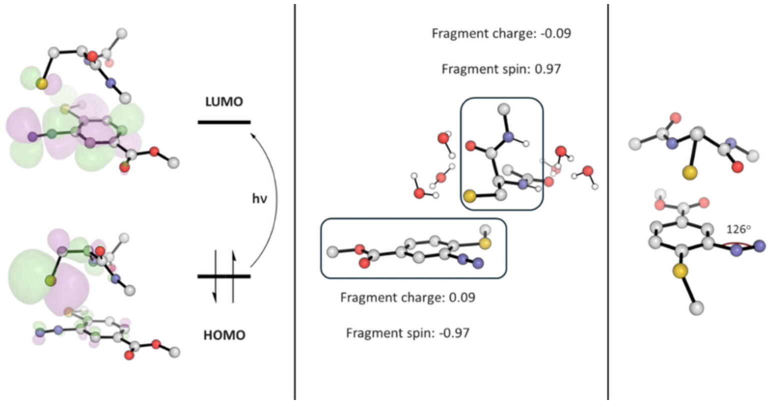

Figure S56. (a) The HOMO→LUMO excitation corresponds to electron transfer from the donor to the acceptor; **(b)** Spin and charge densities for the vertically excited S_1 state confirm donor-to-acceptor charge transfer. (c) Optimization of the S_1 state yields a bent diazonium geometry, consistent with the hybridization change expected upon reduction.

12.7 Post-electron transfer radical relay mechanism

We computed the mechanistic steps following electron-transfer to the aryldiazonium (Figure S55). The proposed mechanism begins with the formation of the reduced aryldiazonium radical (**A**). The reduced diazo group is bent as opposed to its cationic geometry which is linear. Nitrogen expulsion from **A** occurs via transition state **TS-1**, with a low activation barrier ($\Delta G^{\ddagger}$) of 7.4 kcal/mol, yielding aryl radical **B**. Subsequently, an intramolecular HAT reaction proceeds through **TS-2** which has a low activation barrier of 7.0 kcal/mol. While we had initially hypothesized a concerted retro-ene elementary step, analysis of the Intrinsic Reaction Coordinate (IRC) confirmed an overall stepwise loss of maleimide via the intermediacy of radical intermediate **C**.

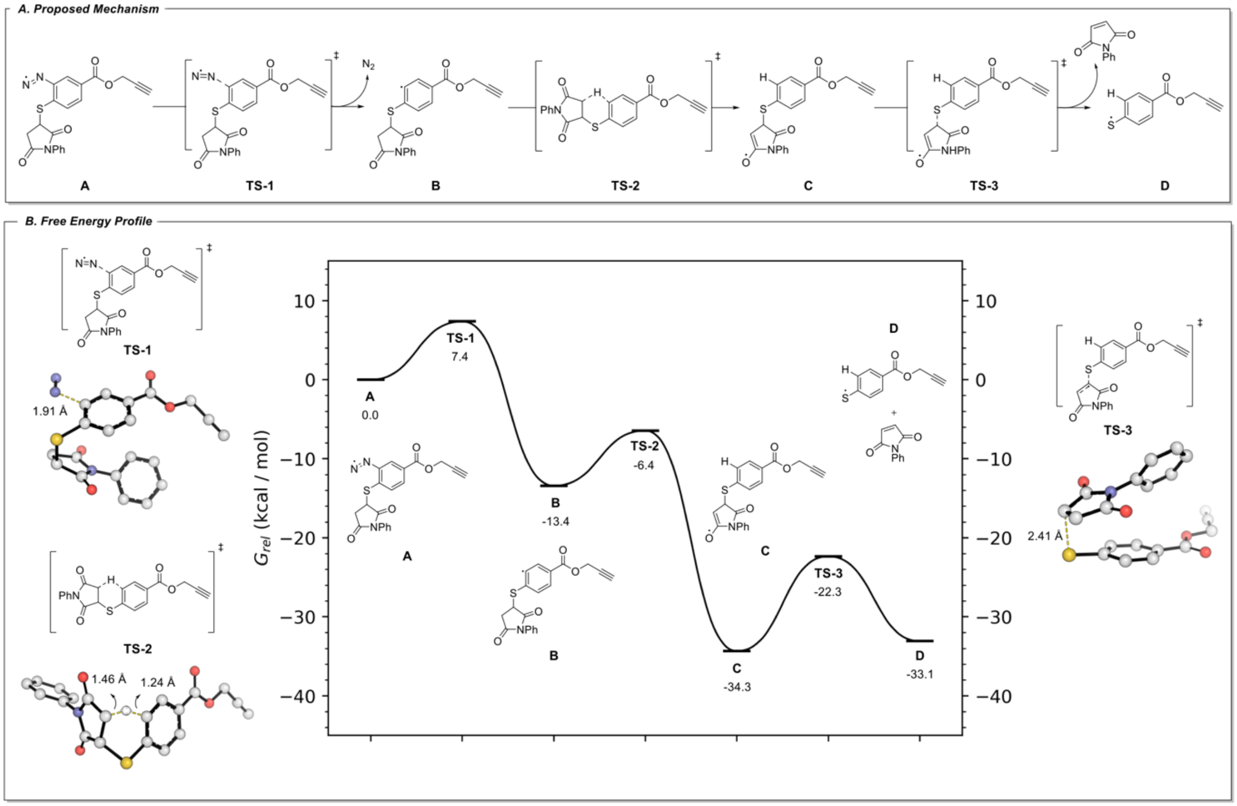

Figure S57. A) Proposed mechanism based on DFT modelling; B) Free energy profile of pathway (kcal/mol) with key bond breaking/ forming TS distances shown in Å.

The HAT step is highly exergonic (ΔG° = -20.9 kcal/mol), indicating significantly greater stability of the Csp^3-centered radical C compared to its aryl counterpart B. This enhanced stability likely arises from two factors: the formation of a stronger Csp^2-H bond (BDE = 110.5 kcal/mol) at the expense of the Csp^3-H bond (BDE =92.4 kcal/mol), and resonance stabilization of the newly-formed radical through delocalization with the adjacent carbonyl group. NBO spin population calculations support this, with significant delocalization of spin density onto the carbonyl oxygen in **C,** while **B** has a highly localized spin density. Following the formation of **C**, the cleavage of the C-S bond occurs via **TS-3**, yielding the thiophene radical (**D**) and releasing a maleimide molecule. This step is identified as the rate-determining step of the mechanism, with an activation barrier of 11.9 kcal/mol, which is readily overcome at 298 K.

Intriguingly, this step is nearly thermoneutral, with a Gibbs free energy change (ΔG° = 1.2 kcal/mol), suggesting that the two species, **C** and **D**, may exist in a reversible equilibrium. At low concentrations the reverse process would be disfavored relative to these standard state values. Examining the mechanism with other range-separated hybrid functionals show qualitatively similar results for all steps involved, especially for the last step. Reaction of thiyl radical **D** with a cysteine radical results in the formation of the observed bioconjugated disulfide product.

12.8 Density Functional Benchmarking

We compared the performance of several different levels of theory in describing the thermochemistry and kinetics of the three elementary steps shown in Figure S55. The results were consistent across the levels examined.

Level of theory	ΔG_1°	$\Delta G_1^{\ddagger}$	ΔG_2°	$\Delta G_2^{\ddagger}$	ΔG_3°	$\Delta G_3^{\ddagger}$
ωB97M-V/def2-TZVPD (SMD, water)	-13.4	7.4	-20.9	7.0	1.2	11.9
ωB97X-D3BJ/def2-TZVPD (SMD, water)	-11.7	8.7	-20.6	6.3	3.2	14.0
ωB97M-D3BJ/def2-TZVPD (SMD, water)	-14.2	7.2	-21.1	7.1	0.1	11.9
CAM-B3LYP-D3BJ/def2-TZVPD (SMD, water)	-11.1	8.9	-20.9	6.0	-0.7	10.8
M062X-D3/def2-TZVPD (SMD, water)	-15.8	7.6	-18.9	9.0	1.0	11.3

Table S9. Benchmarking for single point corrections across different DFAs12.9. Spin Density Isosurfaces

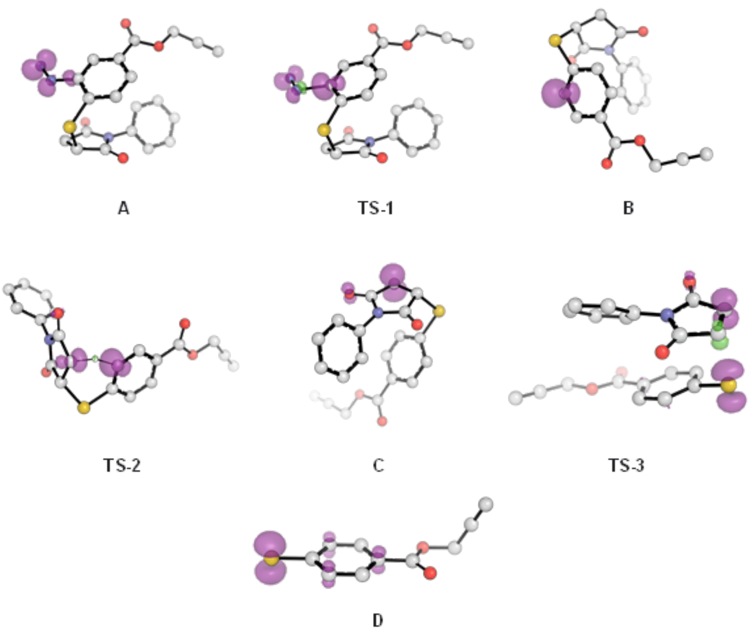

Figure S58. Spin Density Isosurfaces (Isovalue=0.015) of all stationary points with purple and green representing alpha and beta spin densities.

13. NMR Spectra

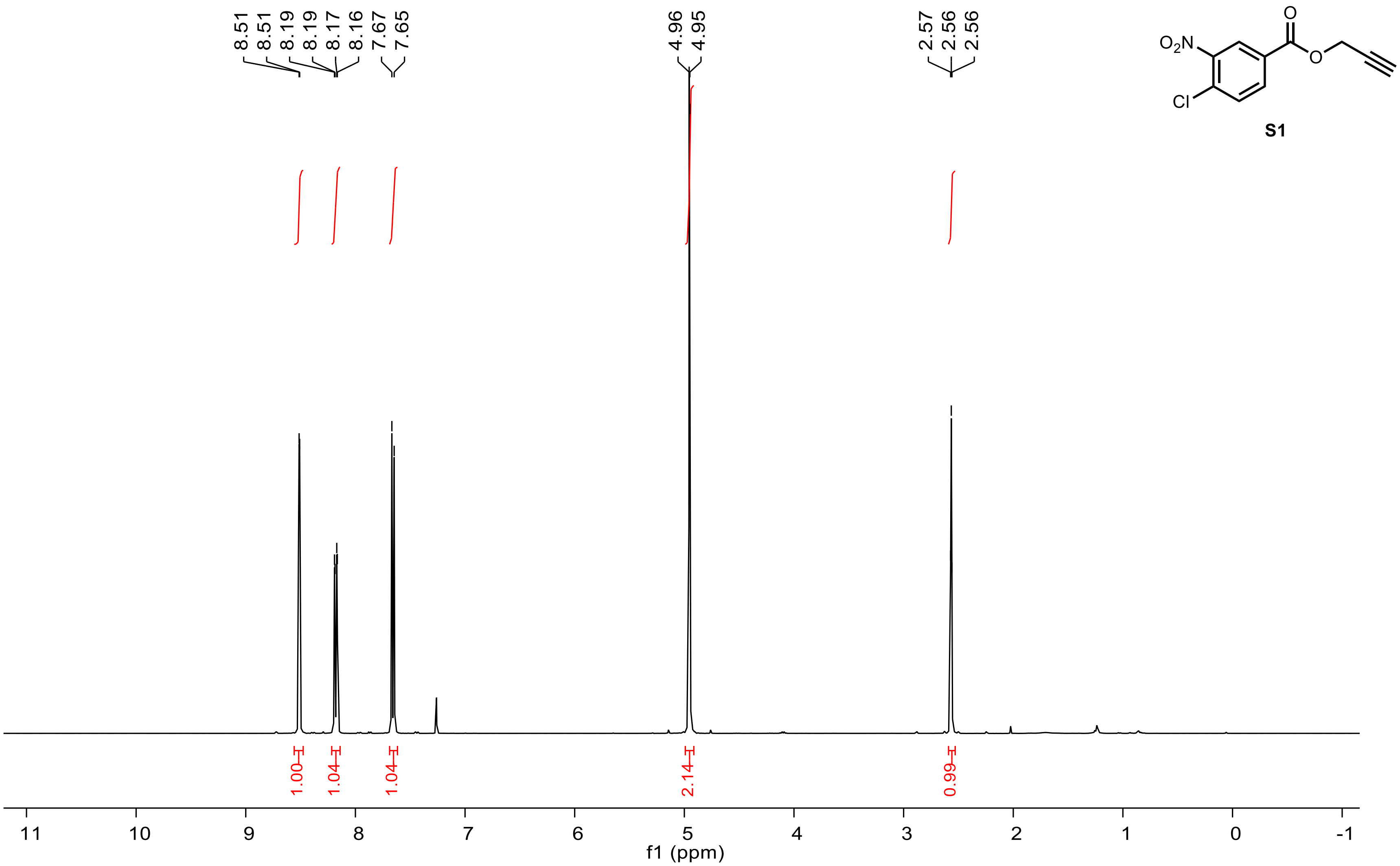

Figure S59. ^{1}H NMR (400 MHz, $CDCl_3$) spectrum of **S1**.

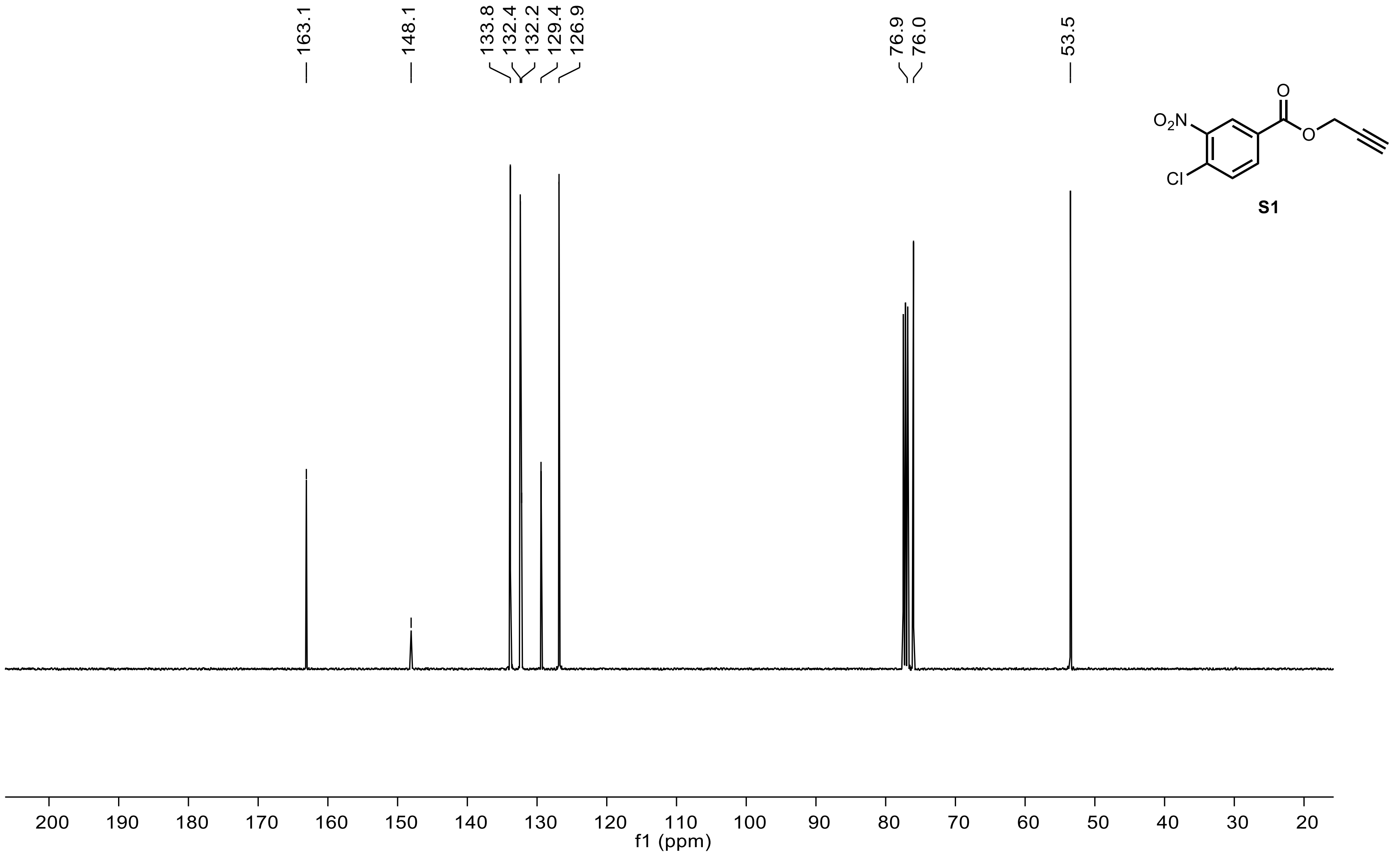

Figure S60. ^{13}C NMR (101 MHz, $CDCl_3$) spectrum of **S1**.

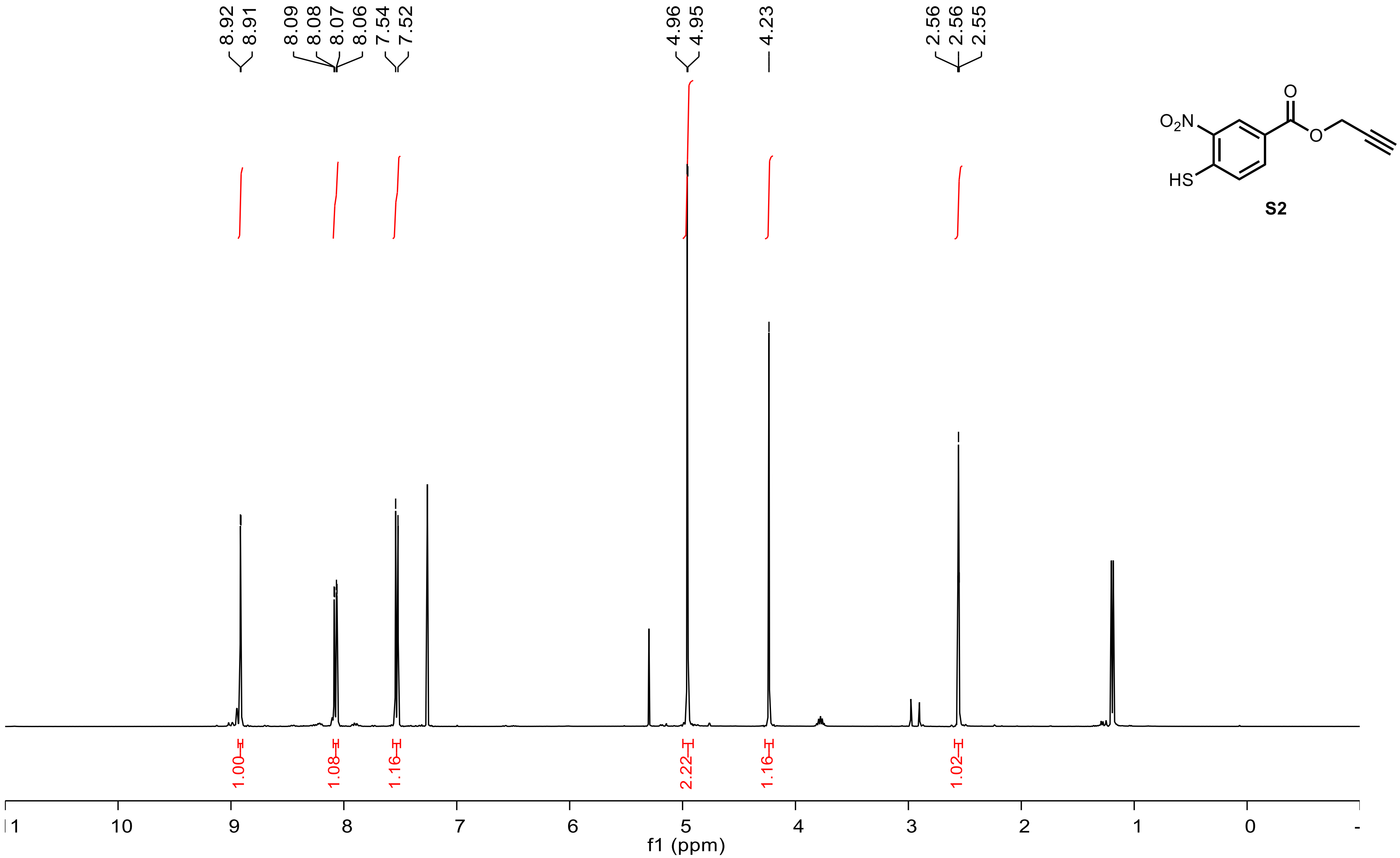

Figure S61. ^{1}H NMR (400 MHz, $CDCl_3$) spectrum of **S2.**

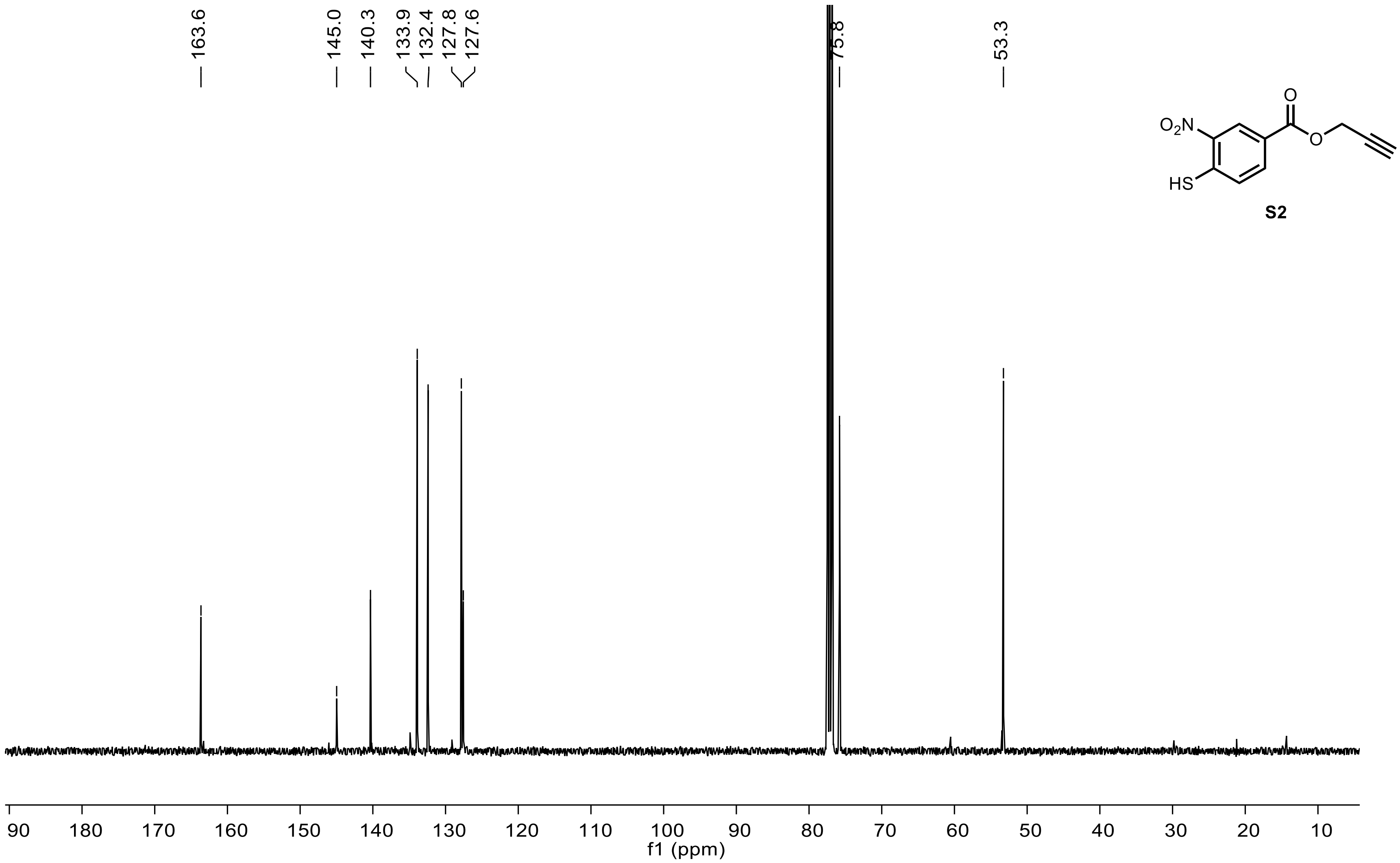

Figure S62. ^{13}C NMR (101 MHz, $CDCl_3$) spectrum of **S2**.

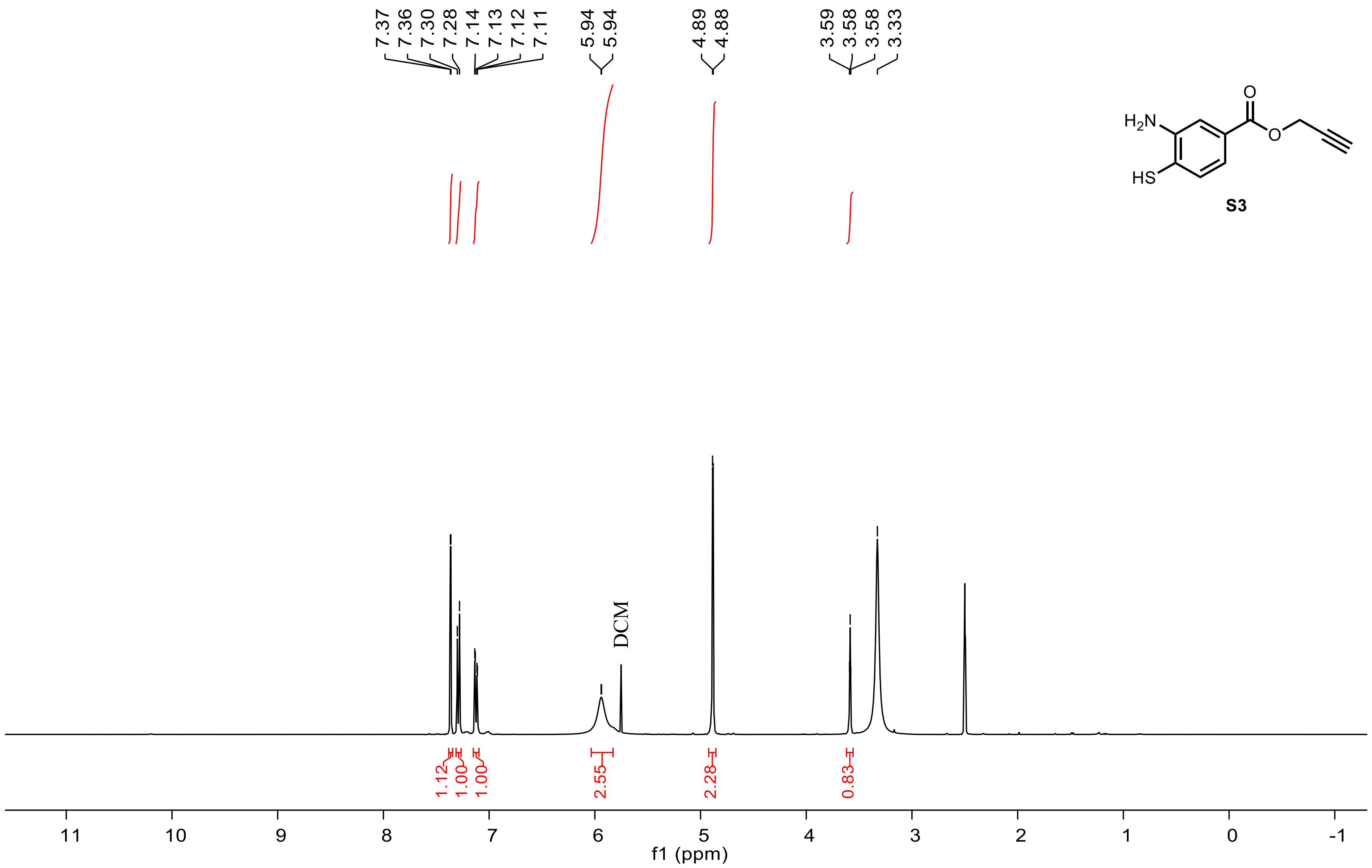

Figure S63. ^{1}H NMR (400 MHz, DMSO-d_6) spectrum of **S3**.

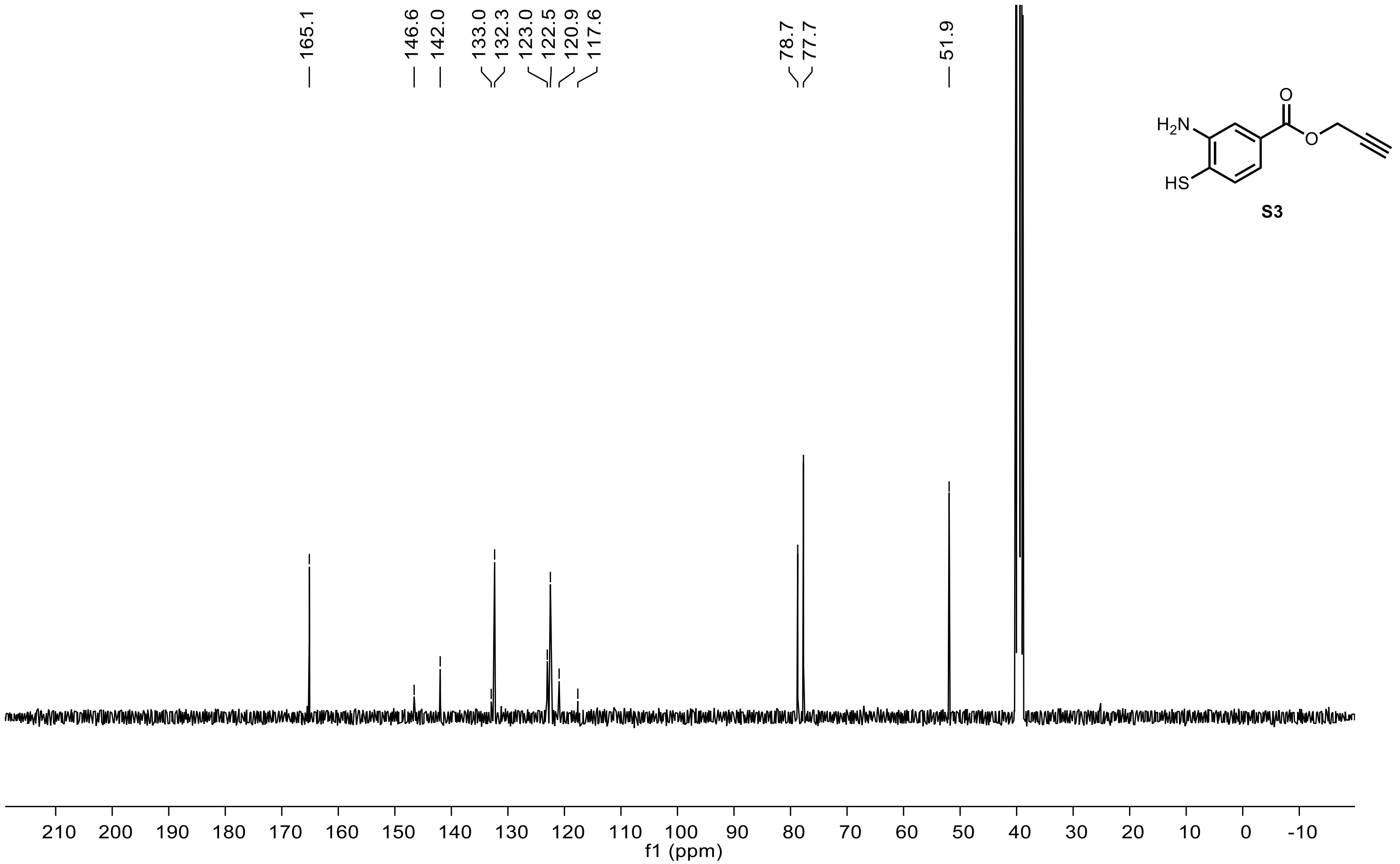

Figure S64. ^{13}C NMR (101 MHz, DMSO-*d*$_6$) spectrum of **S3**.

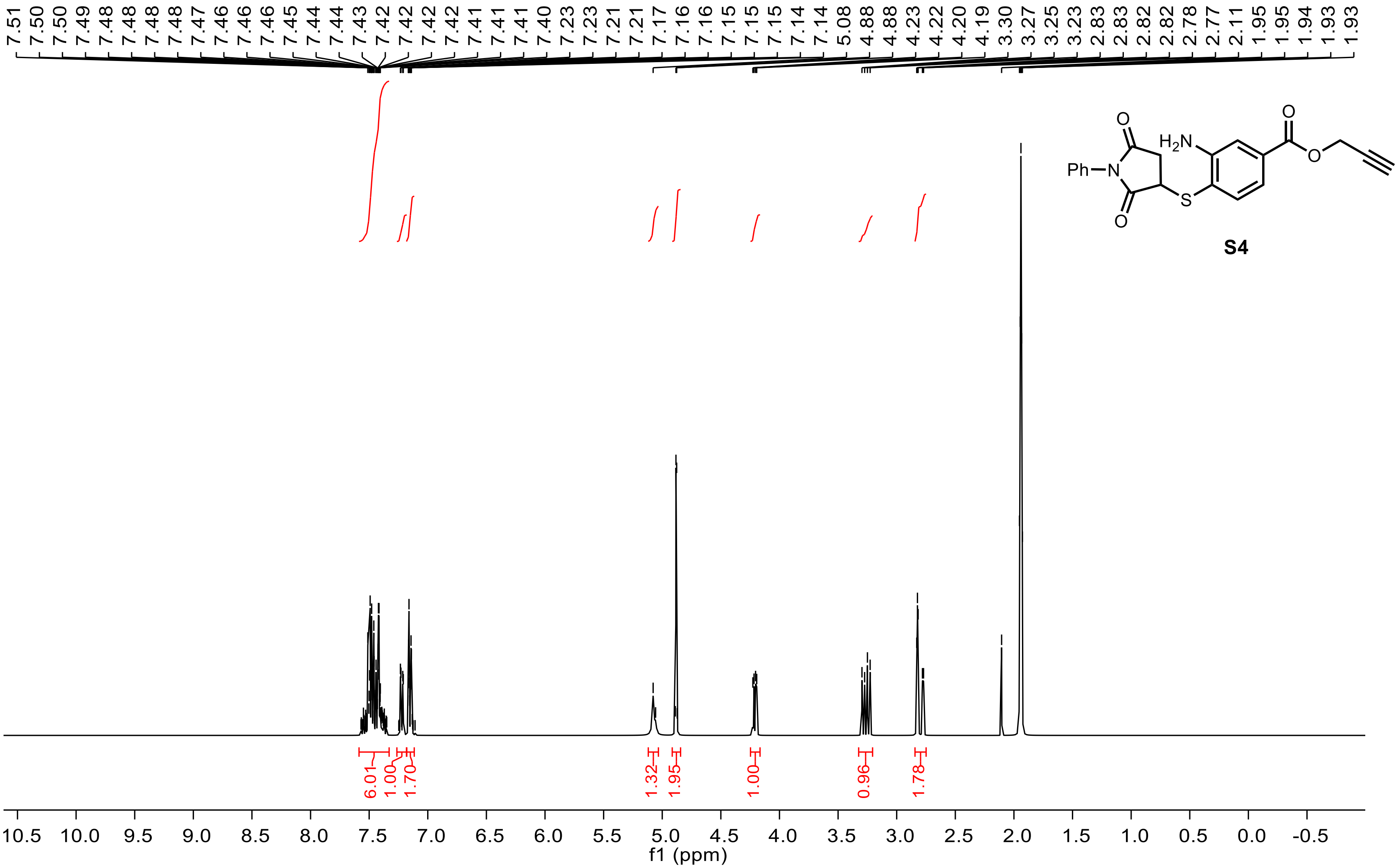

Figure S65. ^{1}H NMR (400 MHz, Acetonitrile-d_3) spectrum of **S4**.

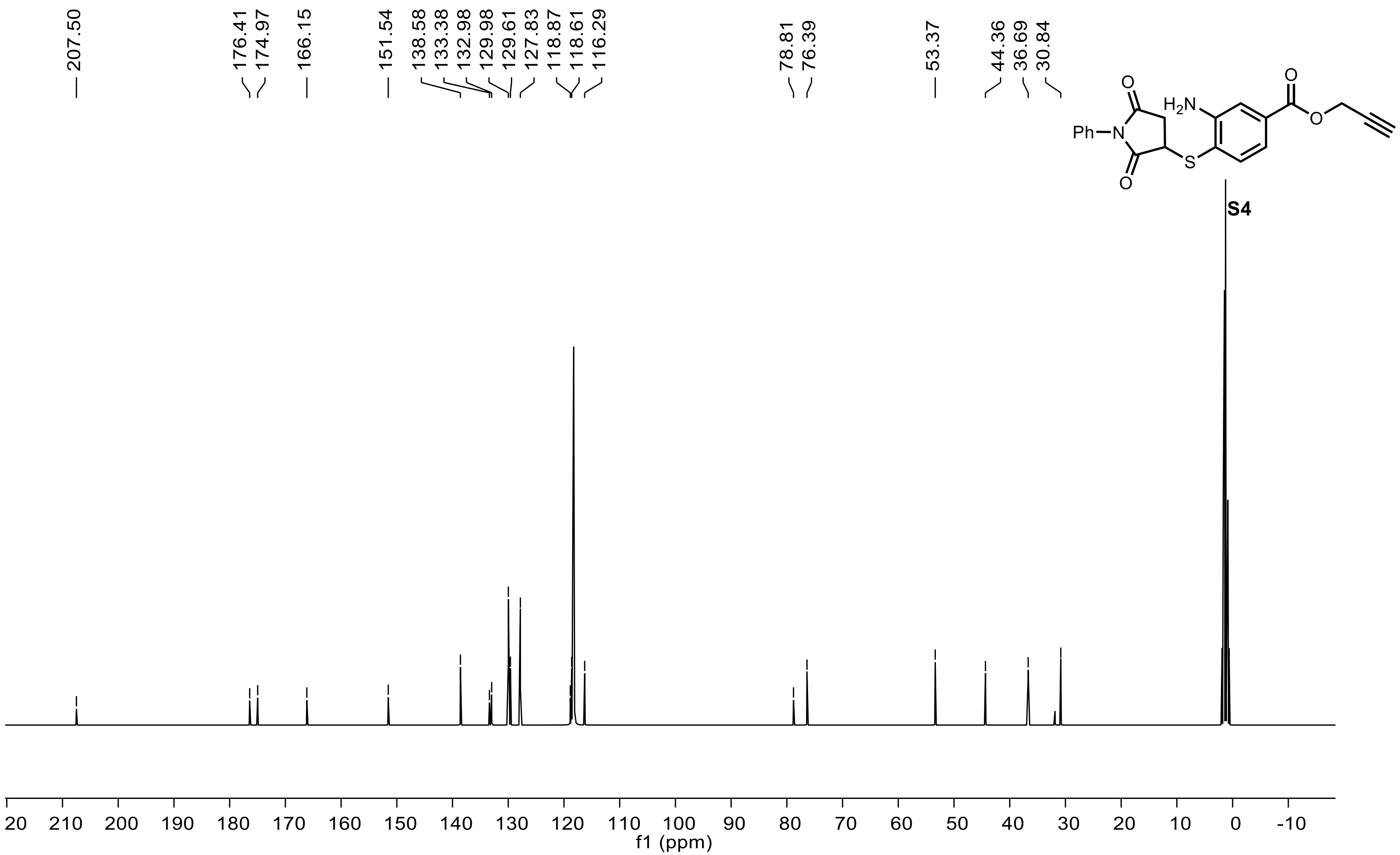

Figure S66. ^{13}C NMR (101 MHz, Acetonitrile-*d*$_3$) spectrum of **S4**.

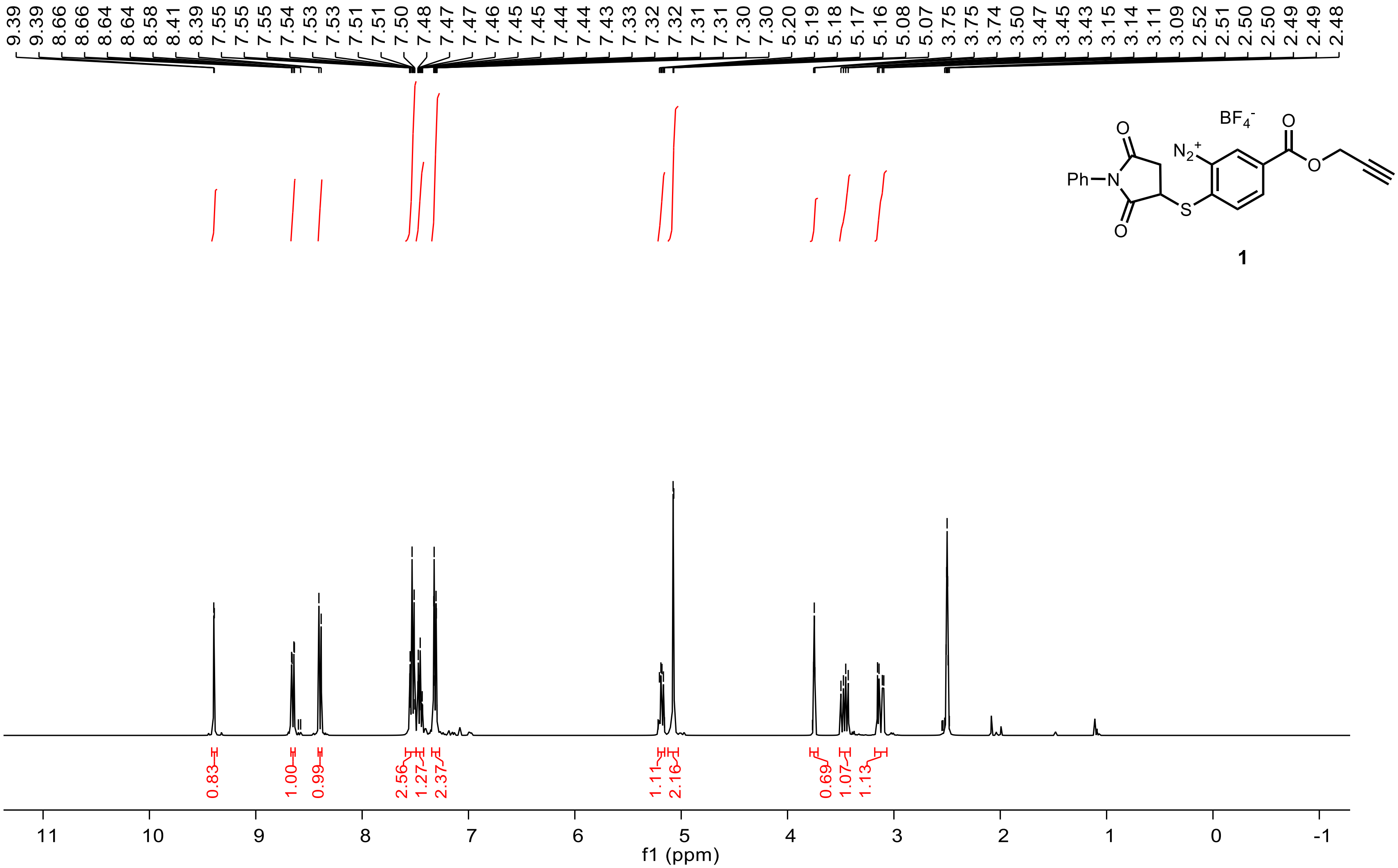

Figure S67. ^{1}H NMR (400 MHz, DMSO-d_6) spectrum of **1**.

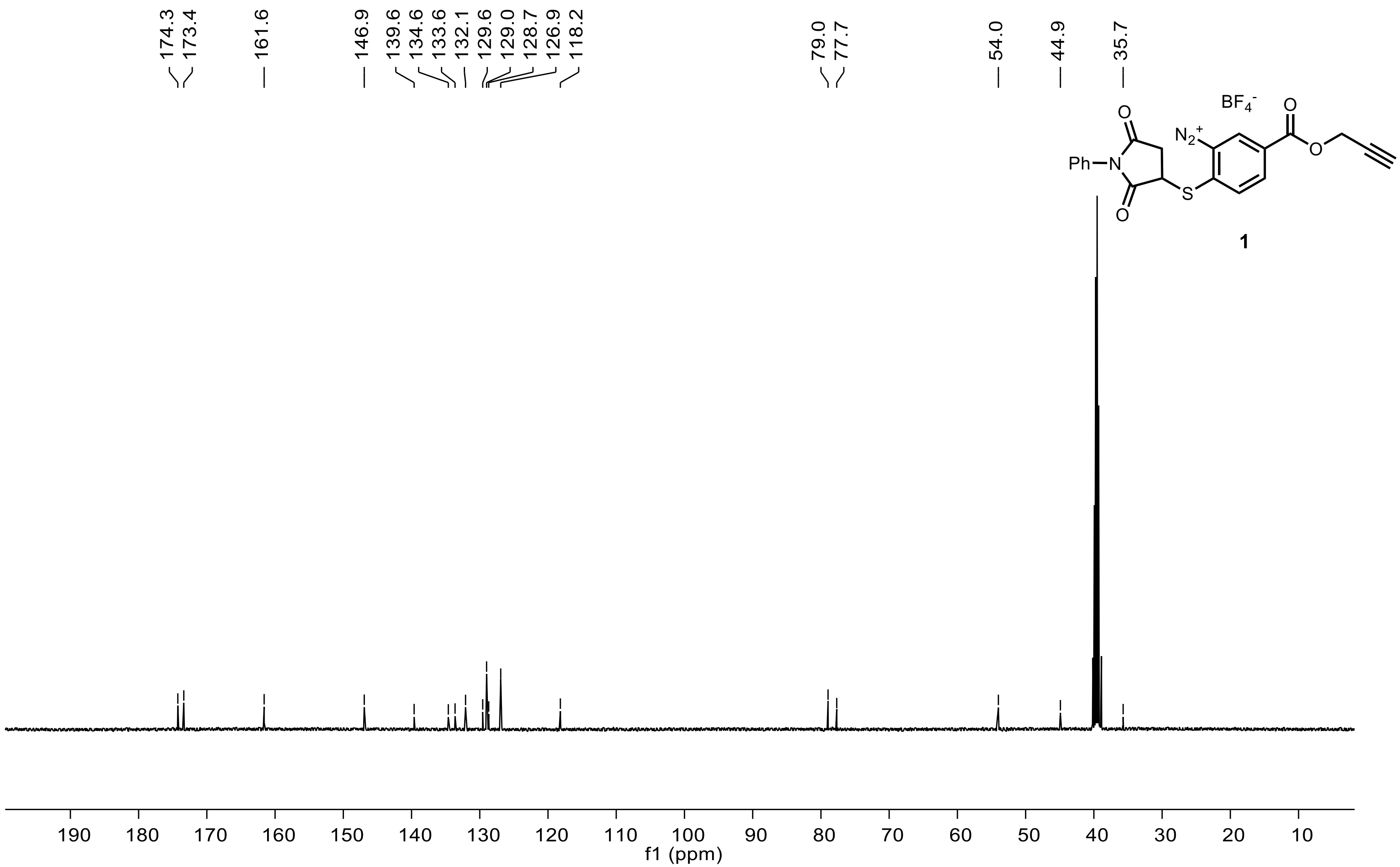

Figure S68. ^{13}C NMR (101 MHz, DMSO-d_6) spectrum of **1**.

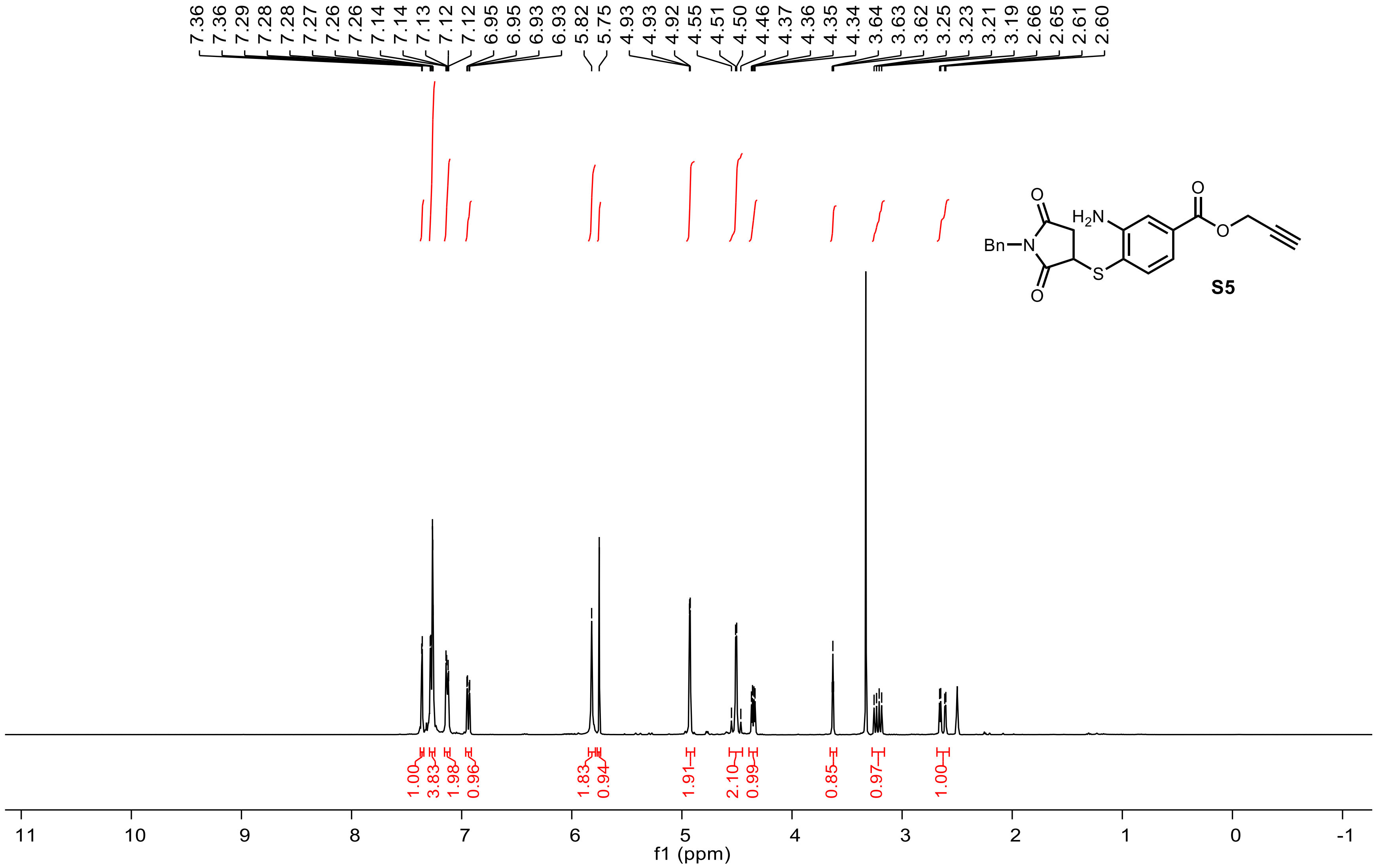

Figure S69. ¹H NMR (400 MHz, DMSO-d_6) spectrum of **S5**.

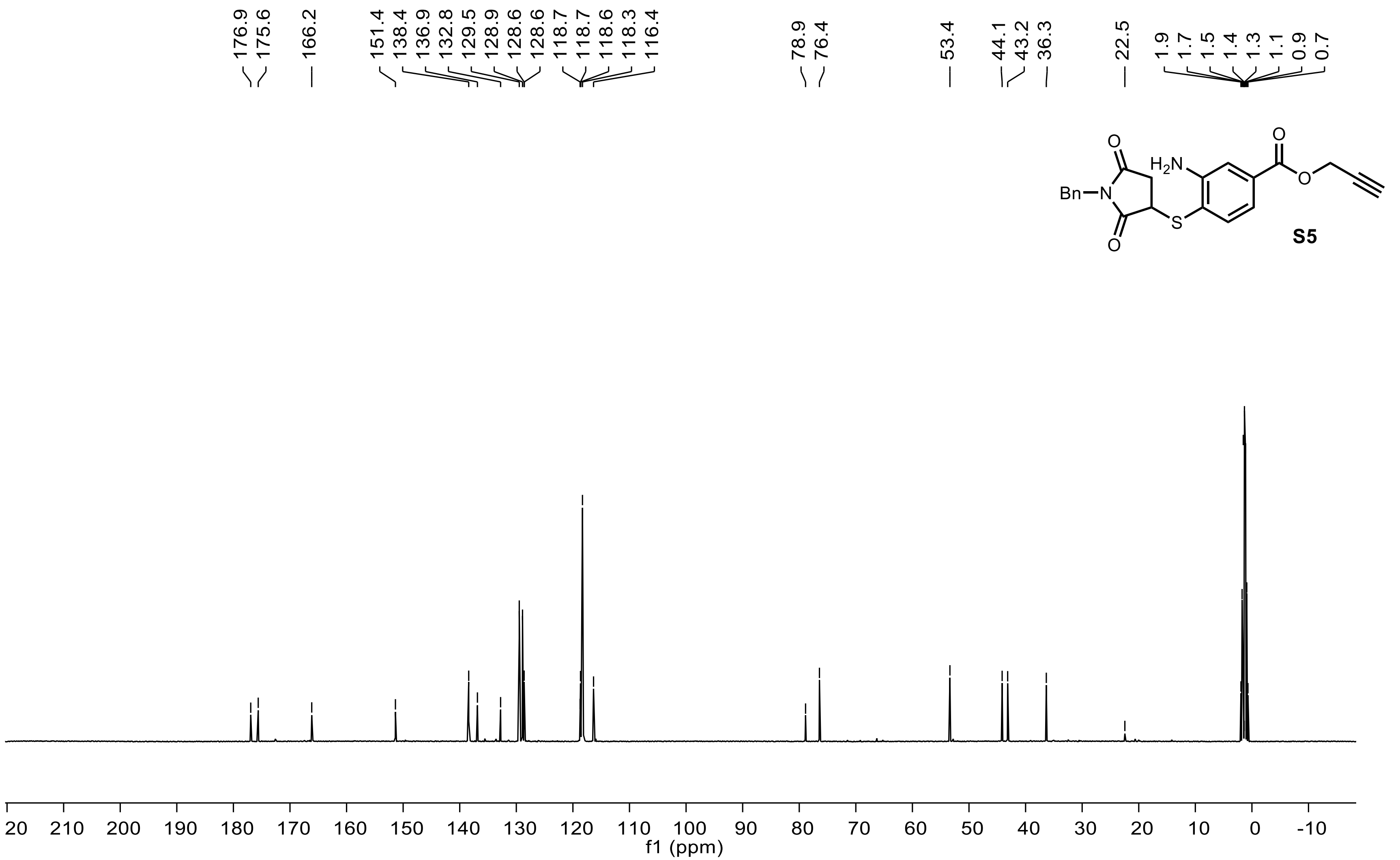

Figure S70. ^{13}C NMR (101 MHz, Acetonitrile-d_3) spectrum of **S5**.

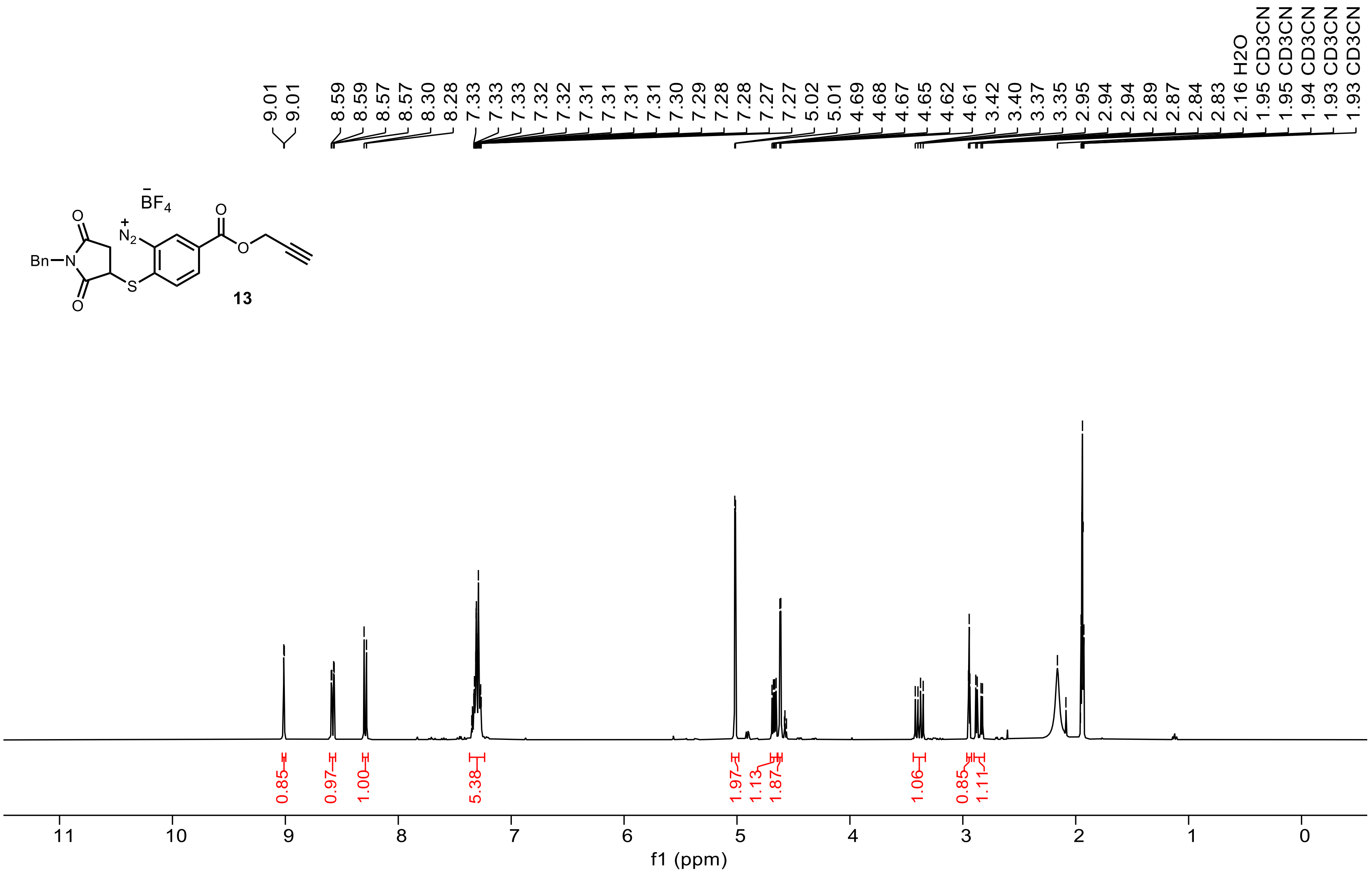

Figure S71. ^{1}H NMR (400 MHz, Acetonitrile-d_3) spectrum of **13**.

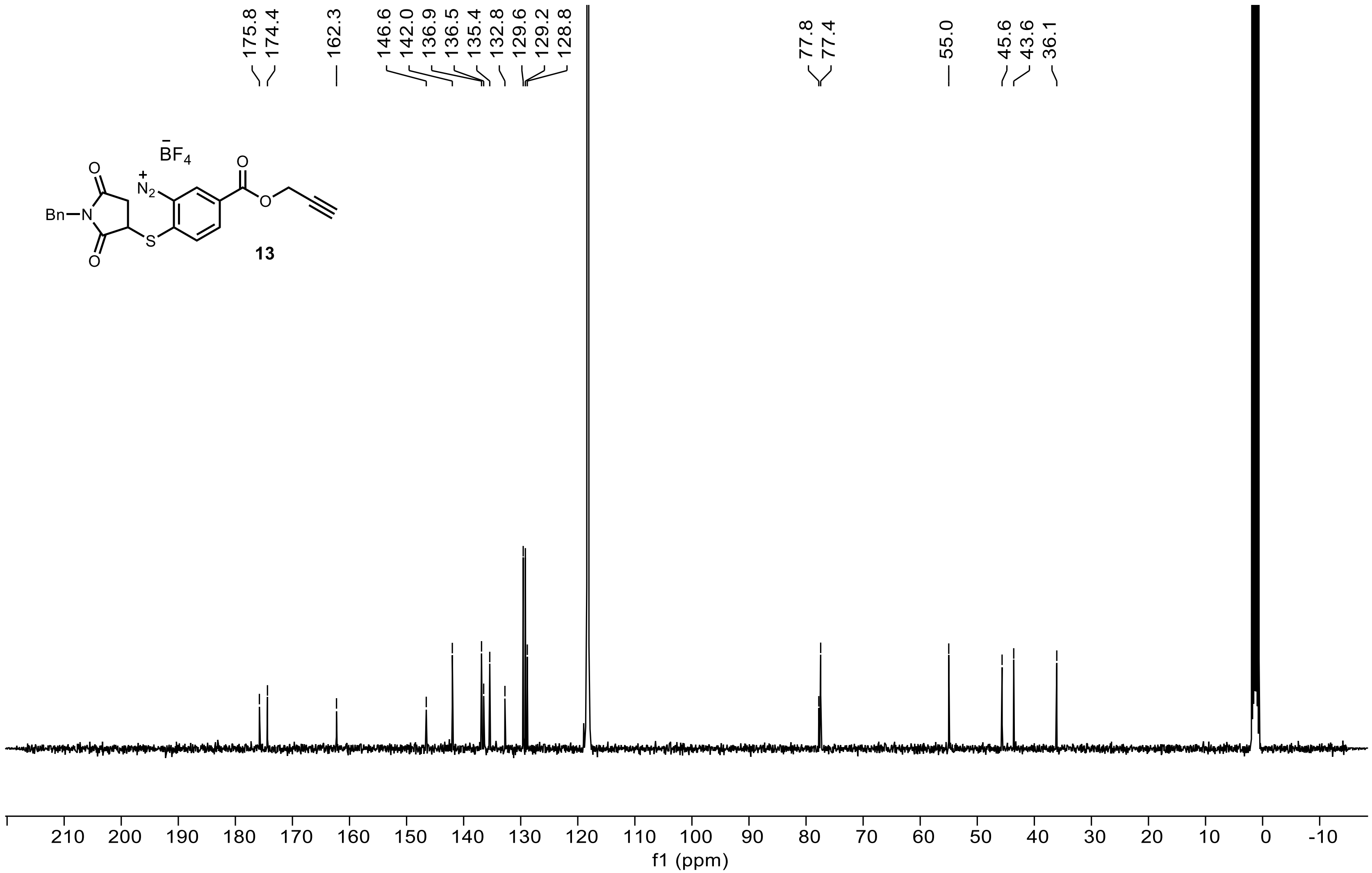

Figure S72. ^{13}C NMR (101 MHz, Acetonitrile-d_3) spectrum of **13**.

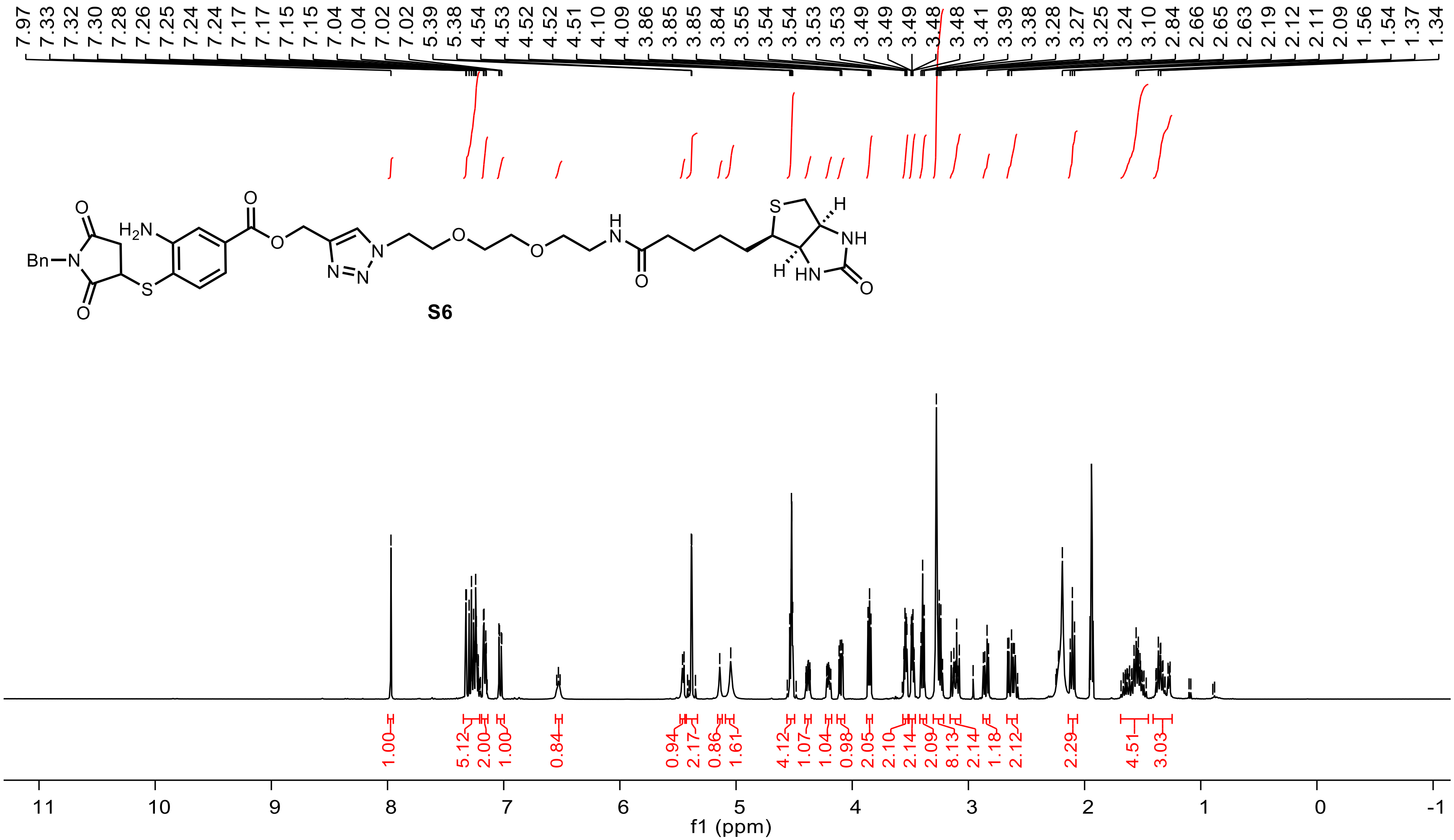

Figure S73. ^{1}H NMR (400 MHz, Acetonitrile-d_3) spectrum of **S6**.

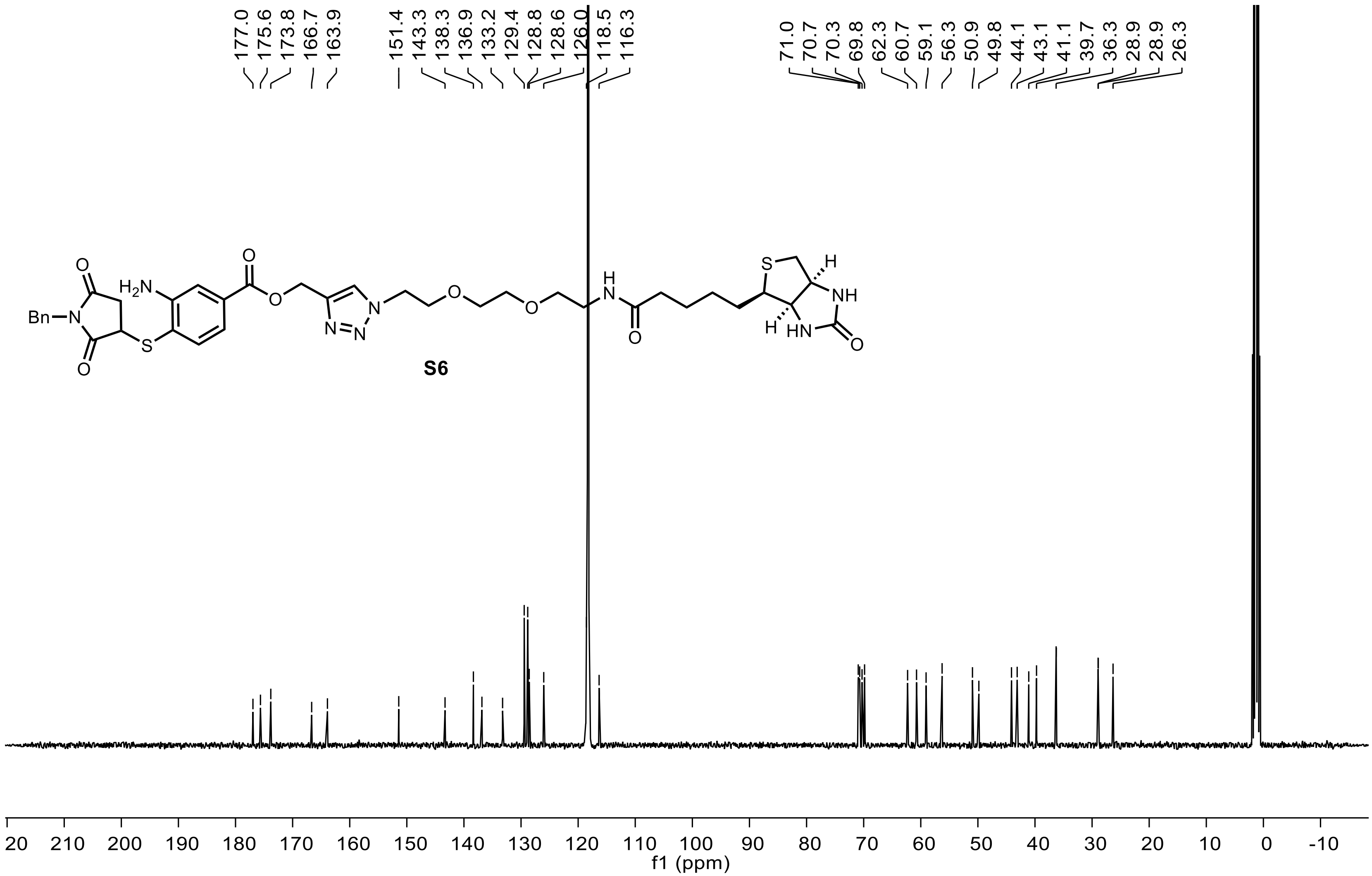

Figure S74. ^{13}C NMR (101 MHz, Acetonitrile-d_3) spectrum of **S6**.

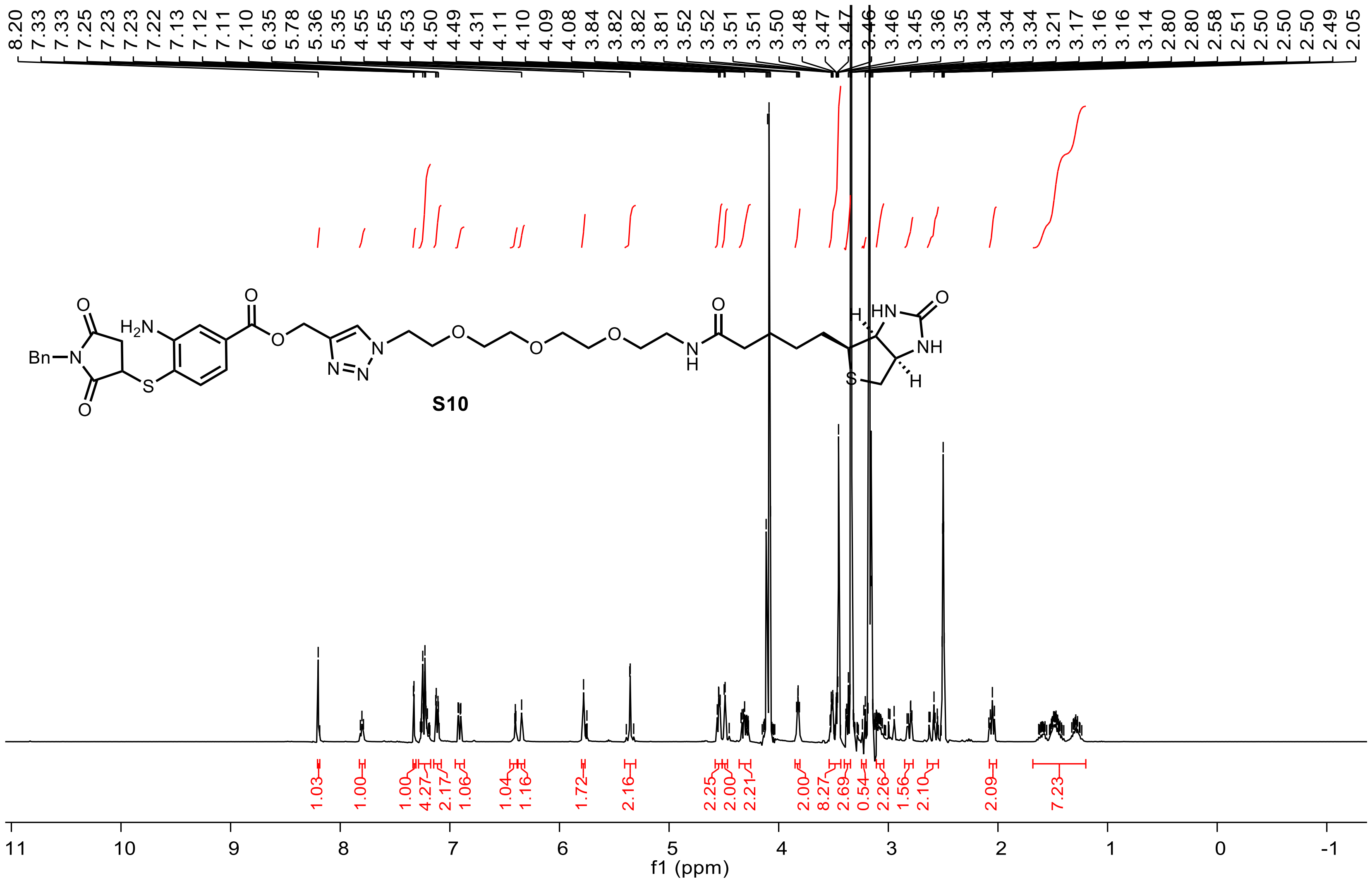

Figure S75. ^{1}H NMR (400 MHz, DMSO-d_6) spectrum of **S10**.

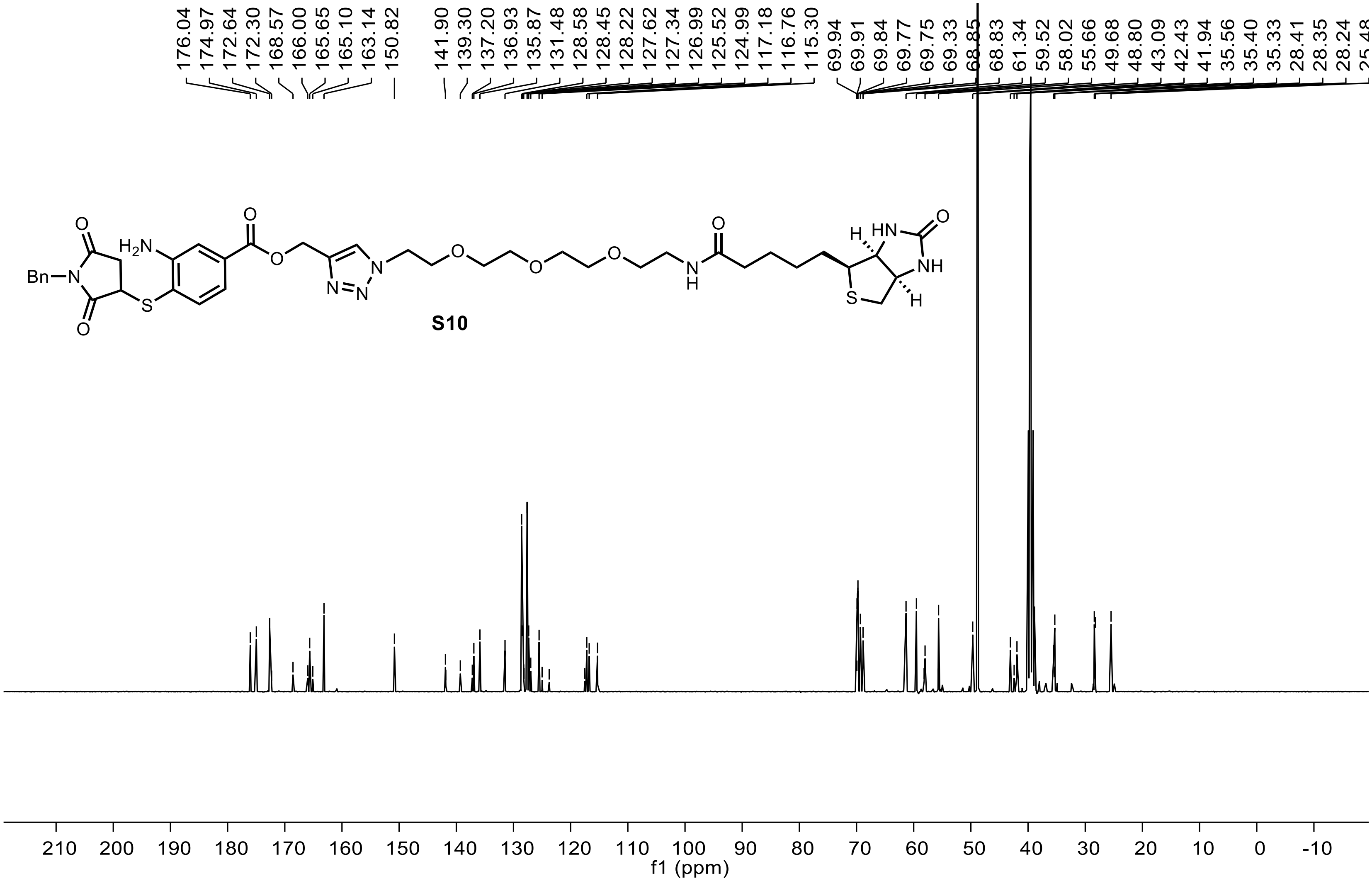

Figure S76. ^{13}C NMR (101 MHz, DMSO-d_6) spectrum of **S10**.

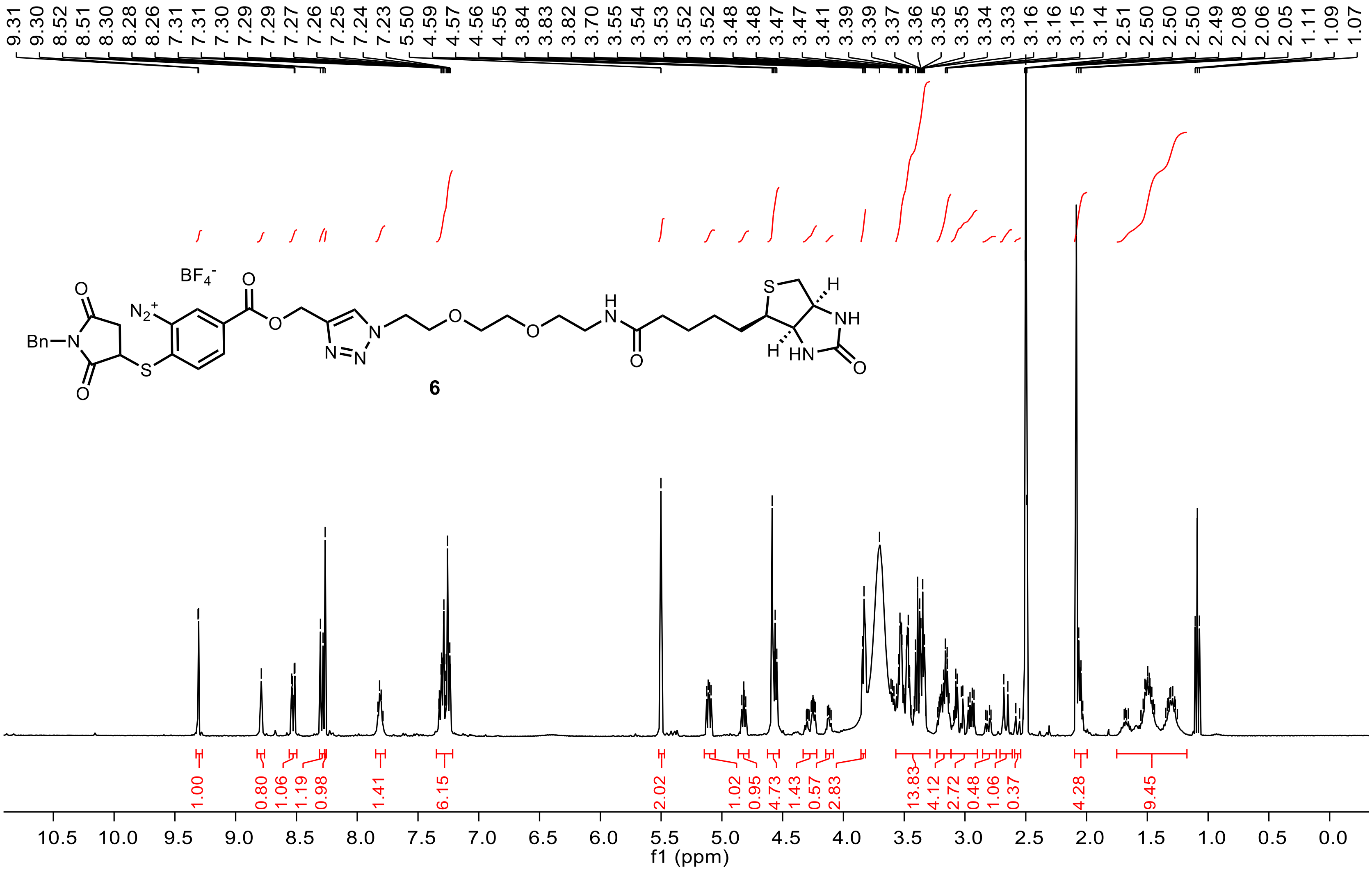

Figure S77. ^{1}H NMR (400 MHz, DMSO-d_6) spectrum of **6**.

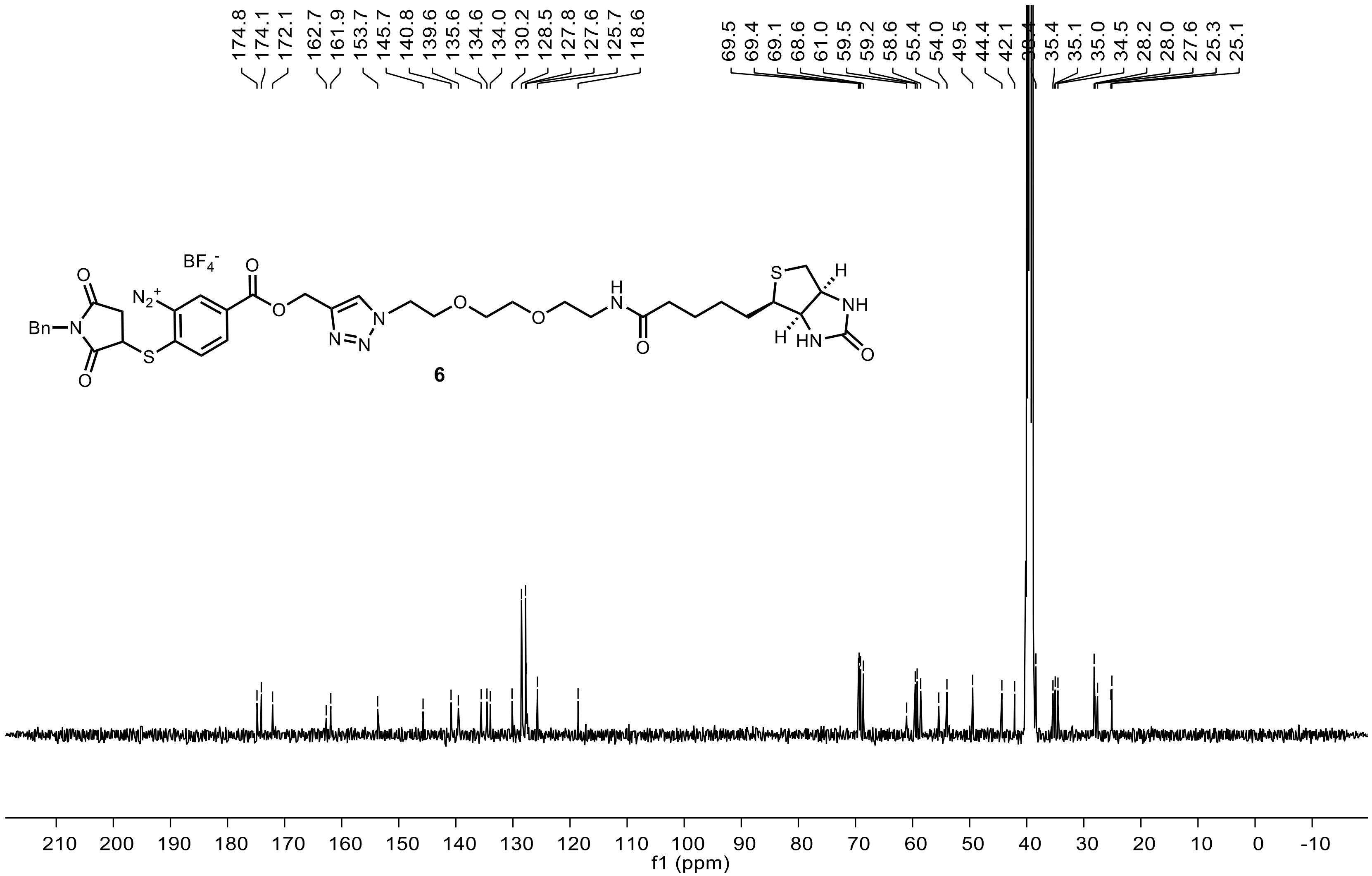

Figure S78. ^{13}C NMR (101 MHz, DMSO-d_6) spectrum of **6**.

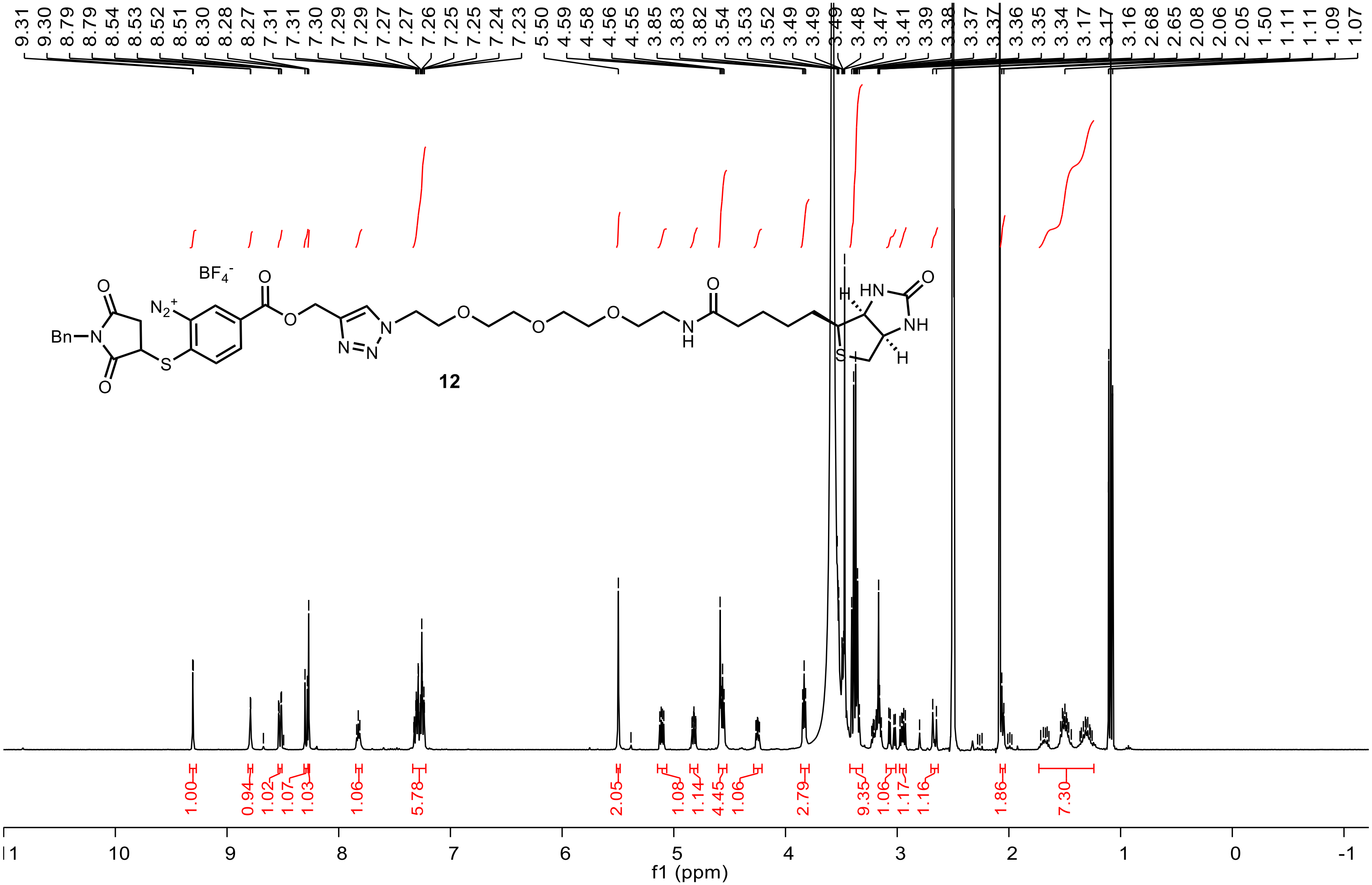

Figure S79. ^{1}H NMR (400 MHz, DMSO-d_6) spectrum of **12**.

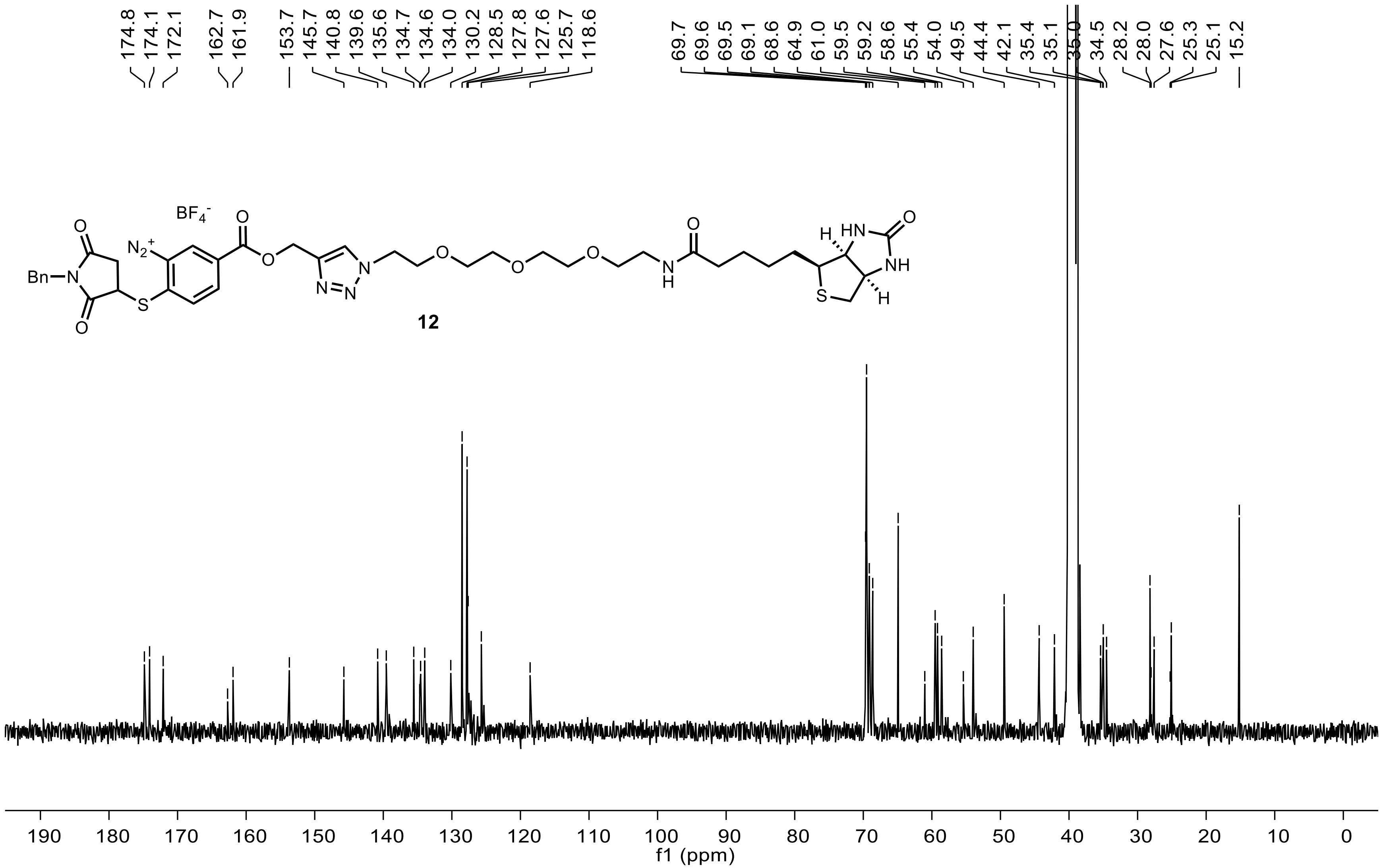

Figure S80. ^{13}C NMR (101 MHz, DMSO-d_6) spectrum of **12**.

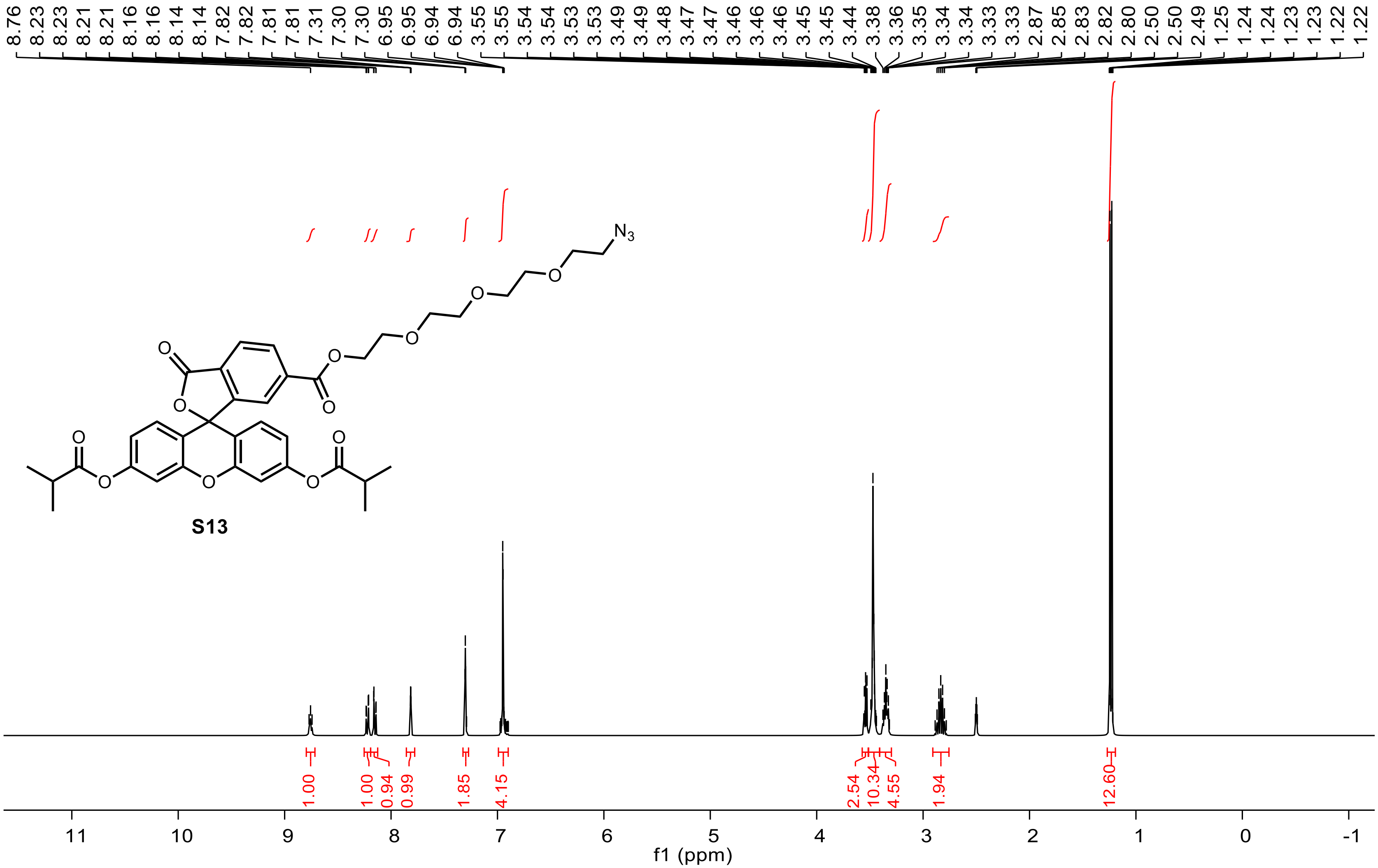

Figure S81. ^{1}H NMR (400 MHz, DMSO-d_6) spectrum of **S13**.

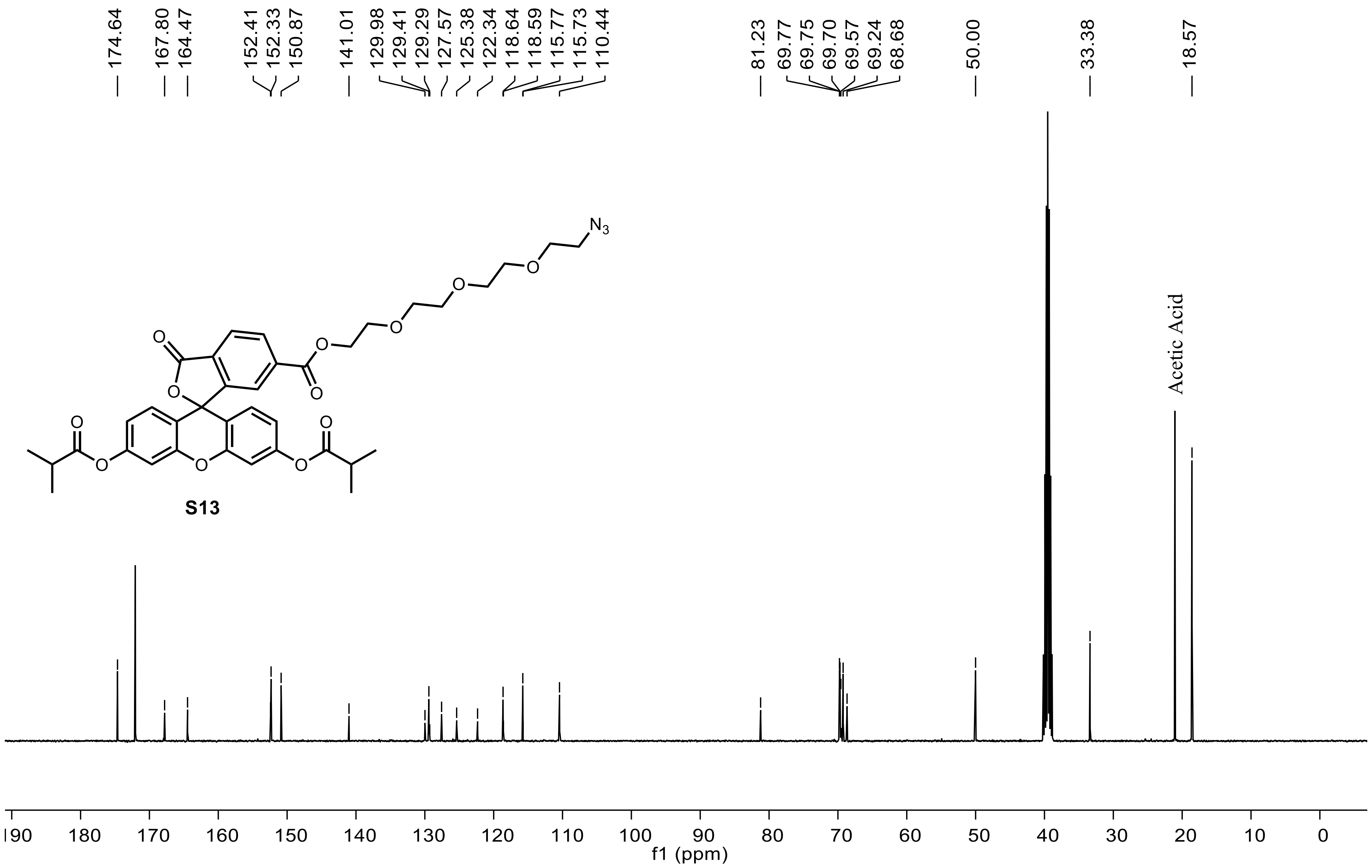

Figure S82. ^{13}C NMR (101 MHz, DMSO-d_6) spectrum of **S13**.

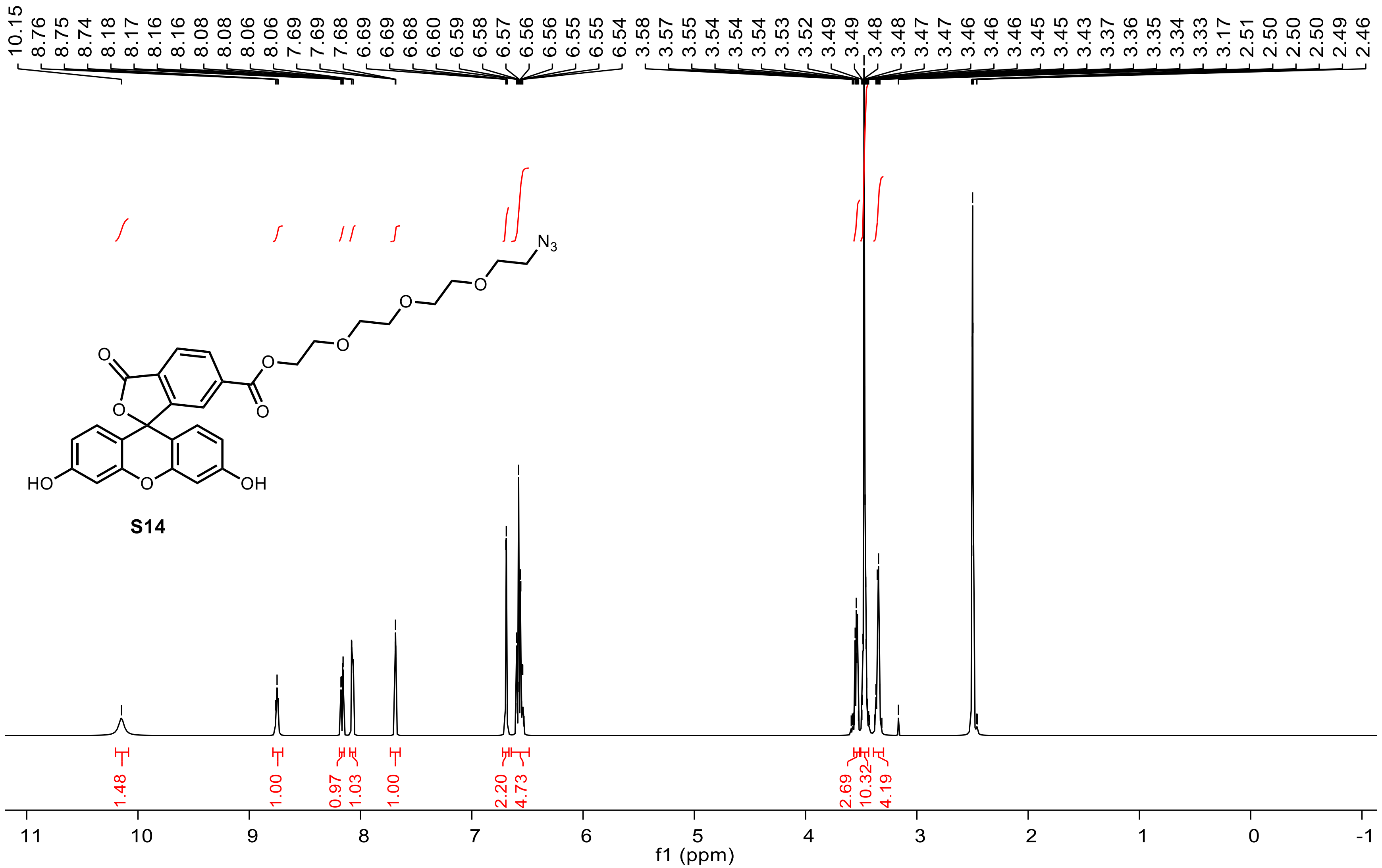

Figure S83. ^{1}H NMR (400 MHz, DMSO-d_6) spectrum of **S14**.

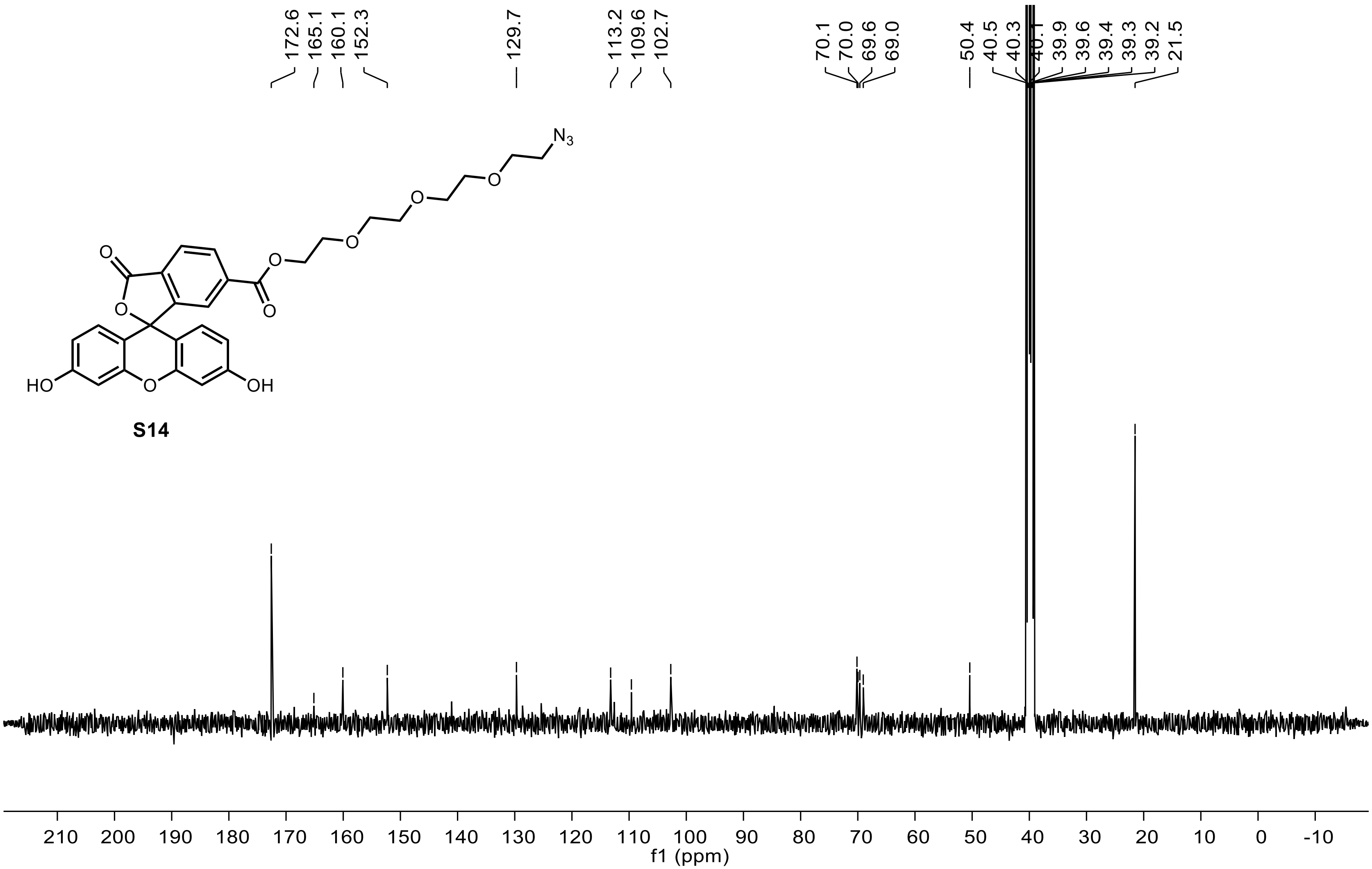

Figure S84. ^{13}C NMR (101 MHz, DMSO-d_6) spectrum of **S14**.

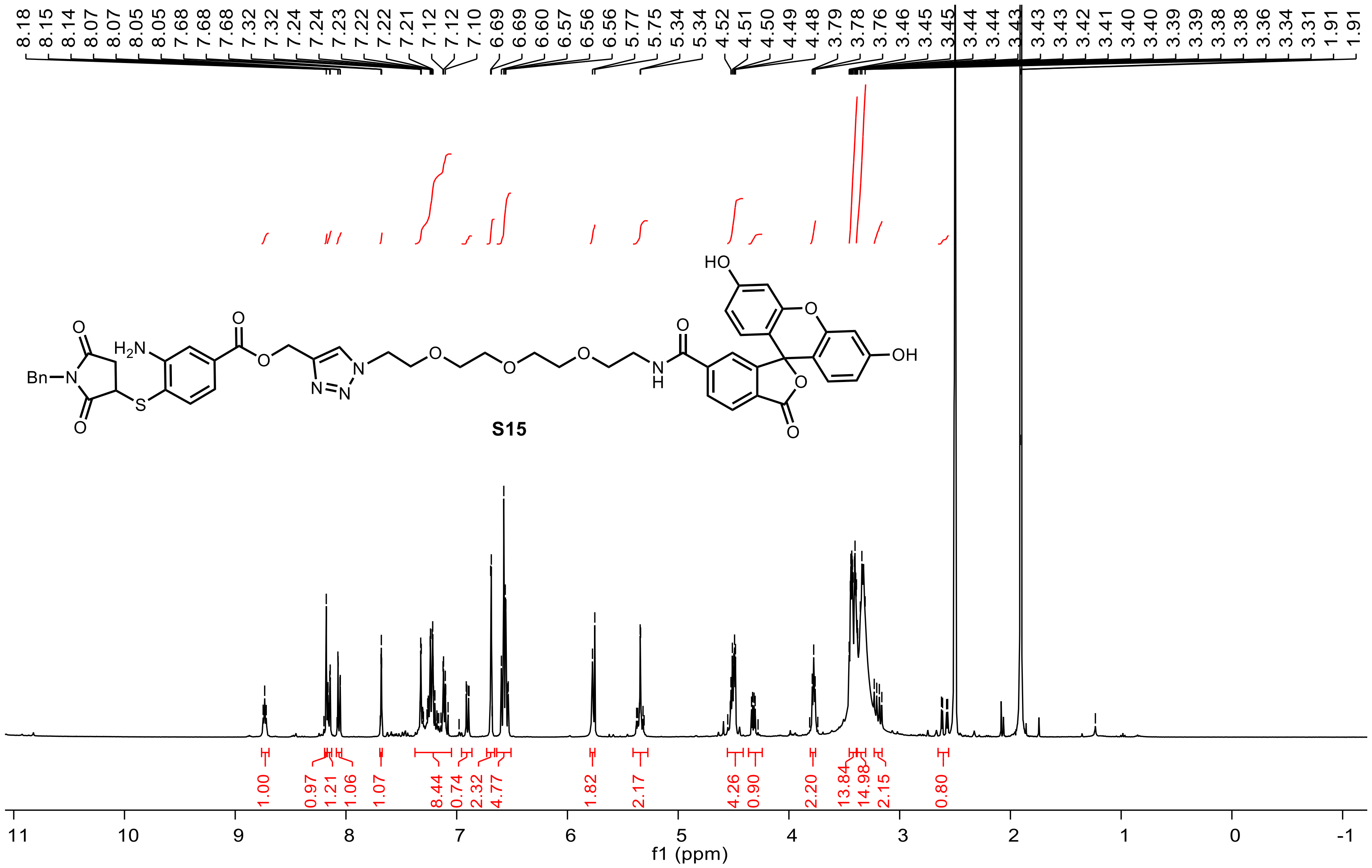

Figure S85. ^{1}H NMR (400 MHz, DMSO-d_6) spectrum of **S15**.

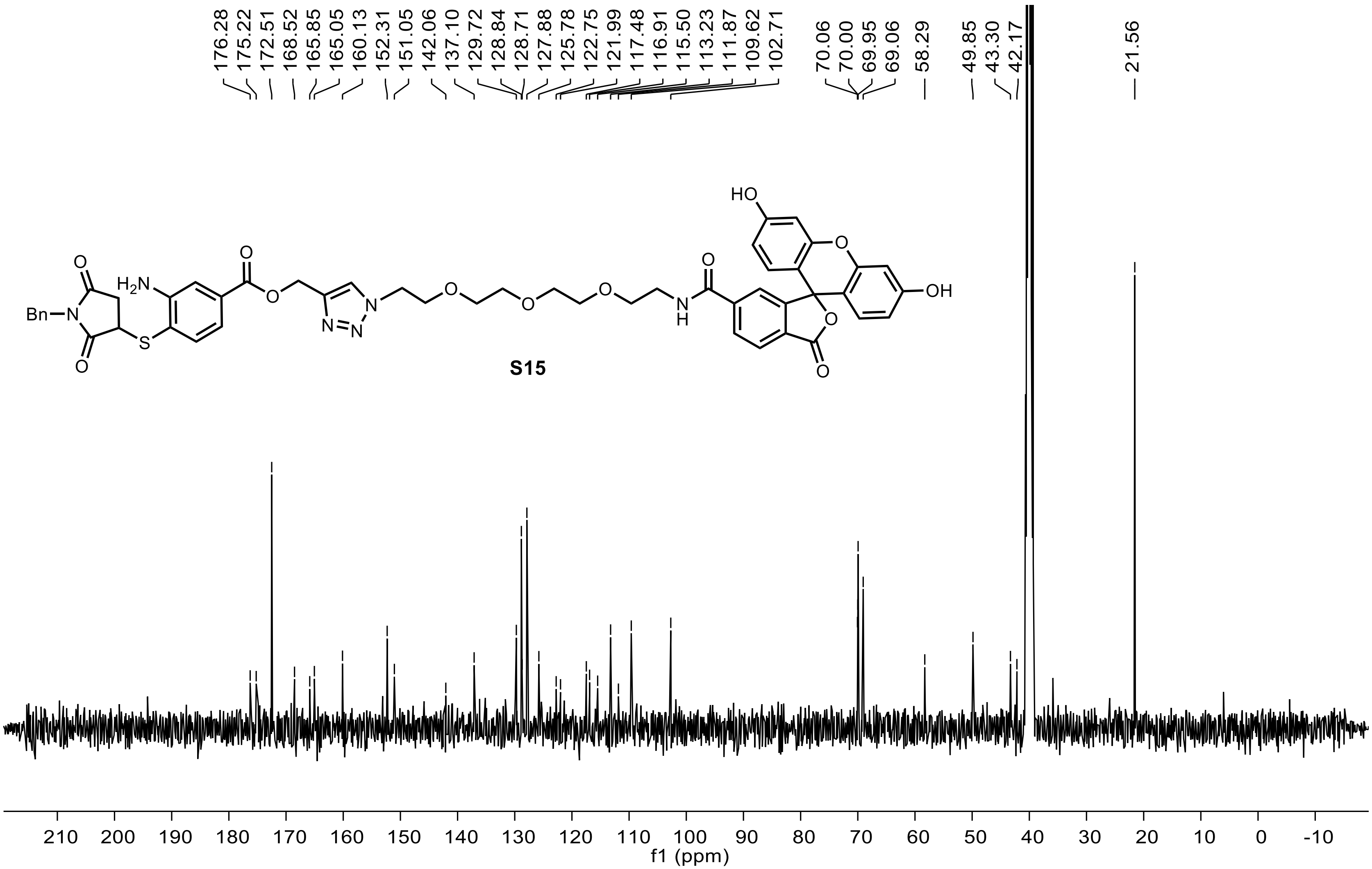

Figure S86. ^{13}C NMR (101 MHz, DMSO-d_6) spectrum of **S15**.

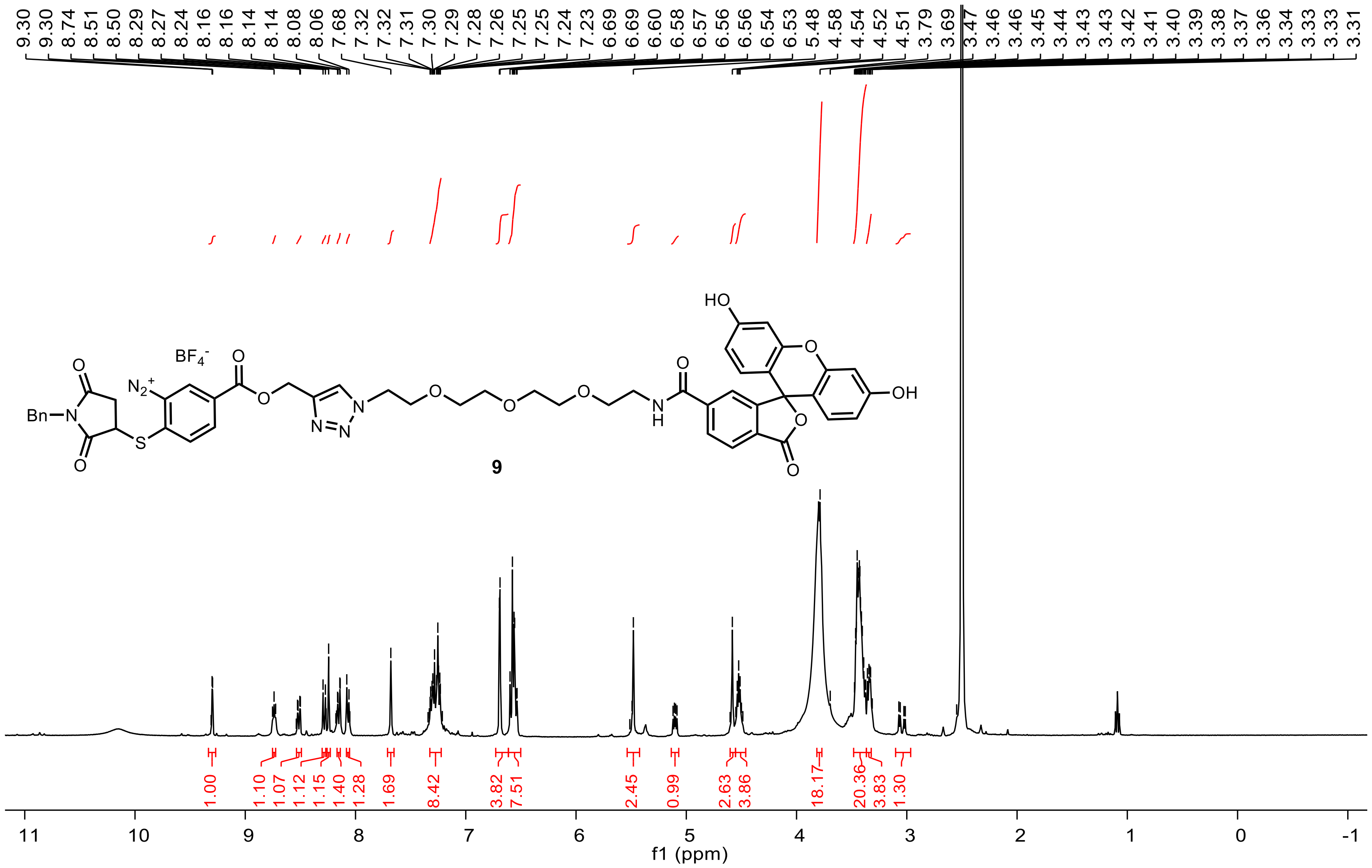

Figure S87. ^{1}H NMR (400 MHz, DMSO-d_6) spectrum of **9**.

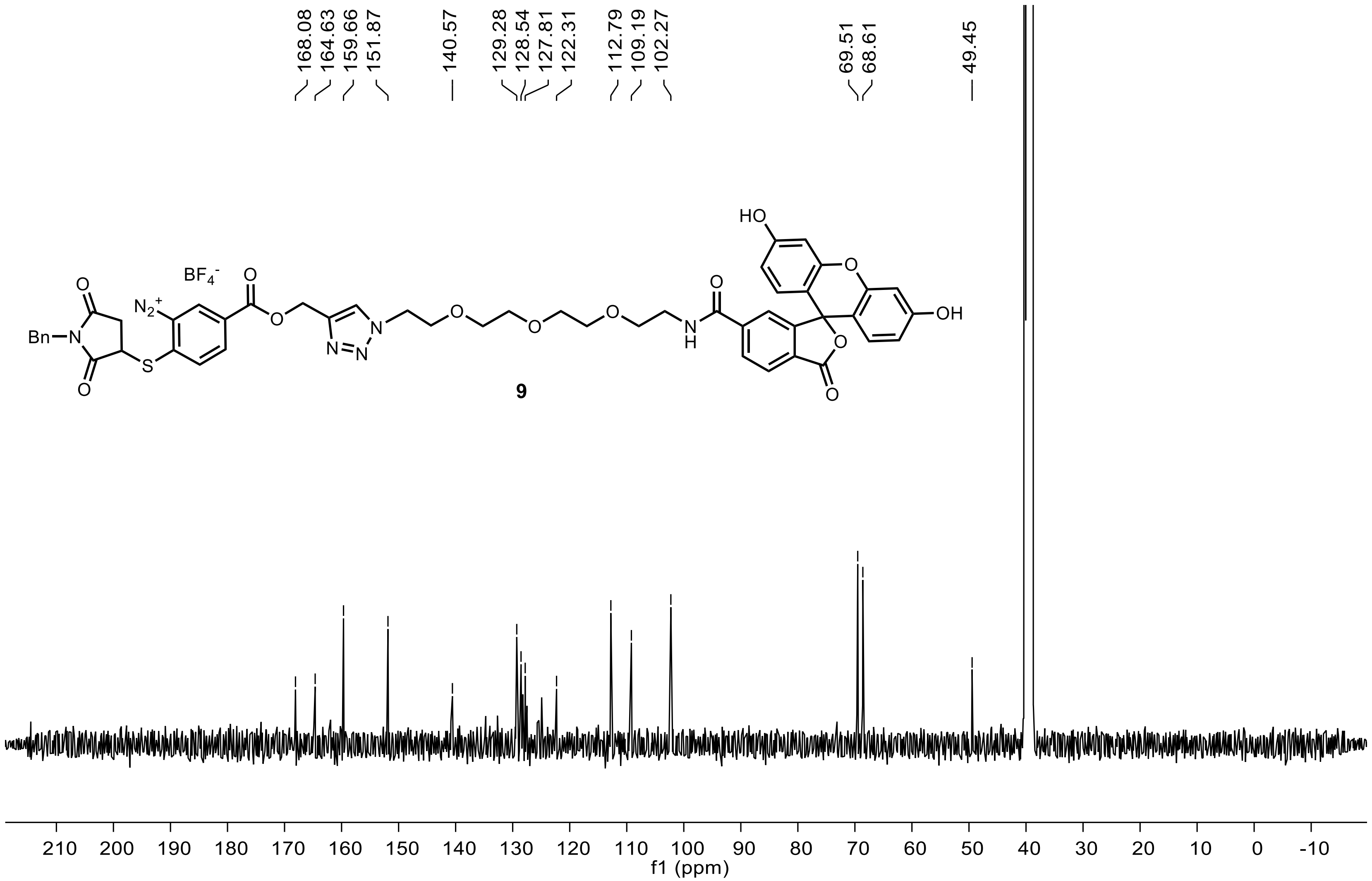

Figure S88. ^{13}C NMR (101 MHz, DMSO-d_6) spectrum of **9**.

14. References

(1) Choi, G. J.; Zhu, Q.; Miller, D. C.; Gu, C. J.; Knowles, R. R. Catalytic alkylation of remote C–H bonds enabled by proton-coupled electron transfer. *Nature* **2016**, *539* (7628), 268-271.
(2) Hartmann, D.; Chowdhry, R.; Smith, J. M.; Booth, M. J. Orthogonal Light-Activated DNA for Patterned Biocomputing within Synthetic Cells. . *J. Am. Chem. Soc.* **2023**, *145* (17), 9471-9480.
(3) Bhuniya, S.; Lee, M. H.; Jeon, H. M.; Han, J. H.; Lee, J. H.; Park, N.; Maiti, S.; Kang, C.; Kim, J. S. A fluorescence off–on reporter for real time monitoring of gemcitabine delivery to the cancer cells. *Chem. Comm.* **2013**, *49* (64), 7141-7143.
(4) Lee, M.; Grissom, C. B. Design, Synthesis, and Characterization of Fluorescent Cobalamin Analogues with High Quantum Efficiencies. *Org. Lett.* **2009**, *11* (12), 2499-2502.
(5) Horatscheck, A.; Wagner, S.; Ortwein, J.; Kim, B. G.; Lisurek, M.; Beligny, S.; Schütz, A.; Rademann, J. Benzoylphosphonate-Based Photoactive Phosphopeptide Mimetics for Modulation of Protein Tyrosine Phosphatases and Highly Specific Labeling of SH2 Domains. *Angew. Chem. Int. Ed.* **2012**, *51* (37), 9441-9447.
(6) Feng, S.-J.; Schumer, F.; Vandaele, E.; Meola, G.; Kradolfer, F.; Luber, S.; Spingler, B. Synthesis of Mesodiphenylhelianthrene from 1-Aminoanthraquinone and the Structural Elucidation of Its Endoperoxide Species after Irradiation. *Org. Lett.* **2022**, *24* (29), 5266-5270.
(7) Liu, J.; Chen, Q.; Rozovsky, S. Utilizing Selenocysteine for Expressed Protein Ligation and Bioconjugations. *J. Am. Chem. Soc.* **2017**, *139* (9), 3430-3437.
(8) Adick, H. J.; Schmidt, R.; Brauer, H. D. A chemical actinometer for the wavelength range 610 – 670 nm. *J. Photochem. Photobiol. A* **1989**, *49* (3), 311-316.
(9) Gaussian 16, Revision C.01, Frisch, M. J.; Trucks, G. W.; Schlegel, H. B.; Scuseria, G. E.; Robb, M. A.; Cheeseman, J. R.; Scalmani, G.; Barone, V.; Petersson, G. A.; Nakatsuji, H.; Li, X.; Caricato, M.; Marenich, A. V.; Bloino, J.; Janesko, B. G.; Gomperts, R.; Mennucci, B.; Hratchian, H. P.; Ortiz, J. V.; Izmaylov, A. F.; Sonnenberg, J. L.; Williams-Young, D.; Ding, F.; Lipparini, F.; Egidi, F.; Goings, J.; Peng, B.; Petrone, A.; Henderson, T.; Ranasinghe, D.; Zakrzewski, V. G.; Gao, J.; Rega, N.; Zheng, G.; Liang, W.; Hada, M.; Ehara, M.; Toyota, K.; Fukuda, R.; Hasegawa, J.; Ishida, M.; Nakajima, T.; Honda, Y.; Kitao, O.; Nakai, H.; Vreven, T.; Throssell, K.; Montgomery, J. A., Jr.; Peralta, J. E.; Ogliaro, F.; Bearpark, M. J.; Heyd, J. J.; Brothers, E. N.; Kudin, K. N.; Staroverov, V. N.; Keith, T. A.; Kobayashi, R.; Normand, J.; Raghavachari, K.; Rendell, A. P.; Burant, J. C.; Iyengar, S. S.; Tomasi, J.; Cossi, M.; Millam, J. M.; Klene, M.; Adamo, C.; Cammi, R.; Ochterski, J. W.; Martin, R. L.; Morokuma, K.; Farkas, O.; Foresman, J. B.; Fox, D. J. Gaussian, Inc., Wallingford CT, 2016.
(10) Neese, F. *Wiley Interdiscip. Rev. Comput. Mol. Sci.* **2012**, *2*, 73–78.
(11) Chai, J.-D.; Head-Gordon, M. *Phys. Chem. Chem. Phys.* **2008**, *10*, 6615–6620.
(12) (a) Hehre, W. J.; Ditchfield, R.; Pople, J. A. *J. Chem. Phys.* **1972**, *56*, 2257–2261; (b) Hariharan, P. C.; Pople, J. A. *Theor. Chim. Acta* **1973**, *28*, 213–222; (c) Krishnan, R.; Binkley, J. S.; Seeger, R.; Pople, J. A. *J. Chem. Phys.* **1980**, *72*, 650–654; (d) McLean, A. D.; Chandler, G. S. *J. Chem. Phys.* **1980**, *72*, 5639–5648; (e) Francl, M. M.; Pietro, W. J.; Hehre, W. J.; Binkley, J. S.; Gordon, M. S.; DeFrees, D. J.; Pople, J. A. *J. Chem. Phys.* **1982**, *77*, 3654–3665.
(13) Marenich, A. V.; Cramer, C. J.; Truhlar, D. G. *J. Phys. Chem. B* **2009**, *113*, 6378–6396.
(14) Fukui, K. *Acc. Chem. Res.* **1981**, *14*, 363–368.
(15) Mardirossian, N.; Head-Gordon, M. *J. Chem. Phys.* **2016**, *144*, 214110.

(16) Neese, F.; Wennmohs, F.; Hansen, A.; Becker, U. *Chem. Phys.* **2009**, *356*, 98–109.
(17) Goerigk, L.; Grimme, S. *J. Chem. Theory Comput.* **2011**, *7*, 291–309.
(18) (a) Weigend, F.; Ahlrichs, R. *Phys. Chem. Chem. Phys.* **2005**, *7*, 3297–3305; (b) Weigend, F. *Phys. Chem. Chem. Phys.* **2006**, *8*, 1057–1065.
(19) Luchini, G.; Alegre-Requena, J. V.; Funes-Ardoiz, I.; Paton, R. S. *F1000Research* **2020**, *9*, 291.
(20) Grimme, S. *Chem. Eur. J.* **2012**, *18*, 9955–9964.
(21) Alegre-Requena, J. V.; Sowndarya, S.; Alturaifi, T.; Perez-Soto, R.; Paton, R. S. *WIREs Comput. Mol. Sci.* **2023**, DOI: 10.1002/wcms.1663.
(22) The PyMOL Molecular Graphics System, version 2.0.7, Schrodinger, LLC.
(23) Marcus, R. A. *J. Chem. Phys.* **1965,** *43*, 679–701.
(24) Nelsen, S. F.; Blackstock, S. C.; Kim, Y. *J. Am. Chem. Soc.* **1987,** *109*, 677–682.
(25) (a) Froitzheim, T.; Kunze, L.; Grimme, S.; Herbert, J. M.; Mewes, J.-M. *J. Phys. Chem. A* **2024,** *128*, 6324–6335. b) Hait, D.; Head-Gordon, M. *J. Phys. Chem. Lett.* **2021,** *12*, 4517–4529.
(26) Gilbert, A. T. B.; Besley, N. A.; Gill, P. M. W. *J. Phys. Chem. A* **2008,** *112*, 13164–13171.
